# Influence of the Lennard-Jones Combination Rules on the Simulated Properties of Organic Liquids at Optimal Force-Field Parametrization

**DOI:** 10.1021/acs.jctc.2c01170

**Published:** 2023-03-15

**Authors:** Marina P. Oliveira, Philippe H. Hünenberger

**Affiliations:** Laboratorium für Physikalische Chemie, ETH Zürich, CH-8093 Zürich, Switzerland

## Abstract

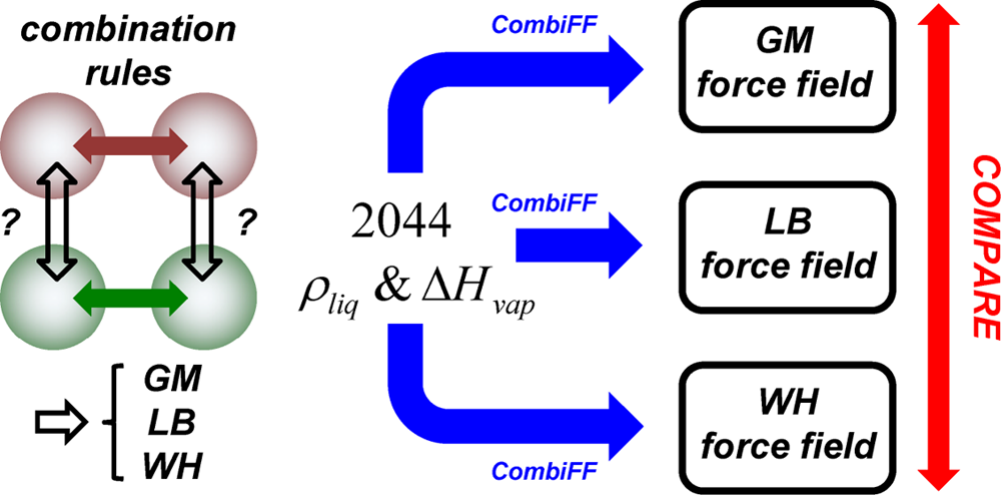

We recently introduced the CombiFF scheme [Oliveira et al., *J. Chem. Theory Comput*. **2020**, *16*, 7525], an approach for the automated refinement of force-field parameters against experimental condensed-phase data for large compound families. Using this scheme, once the time-consuming task of target-data selection and curation has been performed, the force-field optimization itself is both straightforward and fast. As a result, CombiFF provides an ideal framework for evaluating the influence of functional-form decisions on the accuracy of a force field at an optimal level of parametrization. We already used this approach to assess the effect of using an all-atom representation compared to united-atom representations in the force field [Oliveira et al., *J. Chem. Theory Comput*. **2022**, *18*, 6757]. Here, CombiFF is applied to assess the effect of three Lennard-Jones combination rules, geometric-mean (GM), Lorentz–Berthelot (LB), or Waldman–Hagler (WH), on the simulated properties of organic liquids. The comparison is performed in terms of the experimental liquid density ρ_liq_, vaporization enthalpy Δ*H*_vap_, surface-tension coefficient γ, static relative dielectric permittivity ϵ, and self-diffusion coefficient *D*. The calibrations of the three force-field variants are carried out independently against 2044 experimental values for ρ_liq_, and Δ*H*_vap_ concerning 1516 compounds. The resulting root-mean-square deviations from experiment are 30.0, 26.9, and 36.7 kg m^–3^ for ρ_liq_ and 2.8, 2.8, and 2.9 kJ mol^–1^ for Δ*H*_vap_, when applying the GM, LB, and WH combination rules, respectively. In terms of these (and the other) properties, the three combination rules perform comparatively well, with the GM and LB results being more similar to each other and slightly more accurate compared to experiment. In contrast, the use of distinct combination rules for the parameter calibration and property calculation leads to much larger errors.

## 1 Introduction

Classical atomistic simulation^1−3^ and, in particular, molecular dynamics^4−8^ (MD) has become an established tool complementary to experiment for investigating condensed-phase systems. Although classical models represent an approximation to quantum mechanics (QM), they can provide a realistic description of atom-based systems at a much lower computational cost. However, the accuracy of classical MD simulations depends crucially on the quality of the underlying potential-energy function or force field.^9−15^

The automatic optimization of force-field parameters^16−22^ has a long history in the context of target QM data^23−27^ (see also refs (28−33).). However, until recently, the refinement against experimental data has mainly relied on manual (thus laborious and time-consuming) procedures, with only a few attempts at automation, all in the context of atomic liquids^34^ or water.^35−37^ Recent attempts at automating the fitting against condensed-phase observables include the POP^38,39^ and the ForceBalance schemes^29−31,40−45^ (see also refs (46−51)).

Along these lines, the CombiFF^22,52−54^ approach developed in our group is an integrated scheme for the automated refinement of force-field parameters against experimental condensed-phase data, considering entire classes of organic molecules constructed using a fragment library via combinatorial isomer enumeration. The scheme is designed to achieve: (i) a comprehensive (although not exhaustive) coverage of the chemical space; (ii) an appropriate representation of induction effects; and (iii) a complete automation of the topology construction and parameter optimization. As initial applications, CombiFF was used to design GROMOS-compatible united-atom force fields for saturated acyclic compounds with halogen substitution^22^ or including common functional groups of oxygen and nitrogen.^52^

Using CombiFF, once the time-consuming task of target-data selection and curation has been performed, the force-field optimization itself is both straightforward and fast. As a result, CombiFF provides an ideal framework for assessing the influence of functional-form decisions on the accuracy of a force field at an optimal level of parametrization. The goal of the present study is to perform such an assessment considering the choice of a specific combination rule for the Lennard-Jones^55^ (LJ) potential. Despite a probably too steep short-range repulsion,^29,56−58^ the latter potential remains the most common function for representing the van der Waals interactions in condensed-phase (bio)molecular force fields (e.g., OPLS, AMBER, CHARMM, and GROMOS).

The application of combination rules is a widely used strategy to infer the parameters appropriate for LJ interactions between unlike atoms from the knowledge of those between like atoms, thereby reducing the number of parameters required in the definition of a force field. The combination is usually performed in terms of the LJ collision diameter σ (zero of the LJ curve) and well depth ϵ (energy drop at the minimum of the LJ curve). Three common rules are the geometric-mean (GM) rule,^59,60^ involving a geometric averaging of both σ and ϵ,
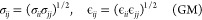
1the Lorentz–Berthelot (LB) rule,^61,62^ involving an arithmetic averaging for σ and a geometric averaging for ϵ,
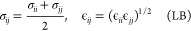
2and the Waldman–Hagler (WH) rule,^63^ involving a sixth-power mean for σ and a geometric mean for ϵσ^6^,
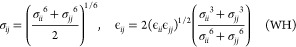
3

There is extensive literature discussing the accuracy and limitations of commonly used combination rules,^64−68^ including comparisons with results from using equations of state^69,70^ or molecular simulations^71−73^ in terms of the thermodynamic properties of fluid mixtures. It is well-known that the GM and LB rules lead to significant deviations from experimental data for rare gases, resulting in overly attractive unlike-pair potentials, while other rules, such as WH, provide a better description^63^ (see Section S.1 in the Supporting Information for a summary). In the condensed phase, the choice of a combination rule also significantly impacts the calculated thermodynamic properties of binary mixtures.^71^ For pure systems, their effect is expected to be more limited and, in large part, compensated for by the effective force-field parameters selected during the calibration.

In a recent study,^54^ we used the CombiFF approach to assess the effect of using an all-atom compared to a united-atom representation in the force field. Here, CombiFF is used to investigate the effect of the combination rule (GM, LB, or WH) on the accuracy of a force field in terms of condensed-phase observables for pure liquids at an optimal level of force-field parametrization.

## 2 Methodology

The CombiFF workflow for calibrating the parameters of a force field based on experimental data concerning a given compound family is described in our previous article.^22^ This section only provides information on its application to the present systematic comparison of combination rules. For ease of reference, a few key numbers (symbols and values) are summarized in Table 1.

**Table 1:** Key Numbers (Symbols and Values) Pertaining to the CombiFF Force-Field Calibration^a^

parameter	value	description
*N*_iso_^cal^	1516	compounds included in the calibration set
*N*_exp_^cal^	2044	experimental data points for the calibration set 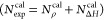
*N*_ρ_^cal^	1440	experimental ρ_liq_ data points for the calibration set
*N*_Δ*H*_^cal^	604	experimental Δ*H*_vap_ data points for the calibration set
		
*N*_sim_^cal^	1607	distinct compounds and *P*, *T*-points (i.e., simulations) for the calibration set
*N*_att_^EE^, *N*_att_	56	number of EE types (or, equivalently, atom types)
*N*_att_^LJ^	17	number of LJ types
*N*_prm_^tot^	289	total number of force-field parameters 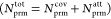
*N*_prm_^cov^	104	number of covalent parameters
*N*_prm_^nbd^	185	number of nonbonded parameters
*N*_prm_^cal^	137	number of parameters that are optimized

a The structures of the *N*_iso_^cal^ representative molecules considered in the calibration are shown in Section S.2 in the Supporting Information. The experimental data concerning these molecules is provided in Section S.3. The information concerning the *N*_prm_^cov^ covalent parameters is summarized in Section S.4. The optimized values of the *N*_prm_^nbd^ nonbonded parameters are provided in Section S.5. Note that the number *N*_prm_^cal^ of parameters optimized is smaller than the total number *N*_prm_^tot^ of force-field parameters, because only non-bonded parameters are optimized, and solely a subset thereof.

The set of compounds considered for the comparison is defined as the union of 11 subfamilies listed in Table 2. Besides alkanes, it includes homofunctional noncyclic aliphatic molecules with up to 10 carbon atoms representative of nine chemical functional groups, namely, halogen, ether, aldehyde, ketone, ester, alcohol, carboxylic acid, amine, and amide (with up to four occurrences of the given functional groups in the molecule), along with heterobifunctional molecules.

**Table 2:** Family of Compounds Used for the Force-Field Calibration^a^

function	acronym	char	*n*	*m*	*N*^sim^	subfamily description
alkanes	ALK	A	1–10	–	150	C_1_–C_10_ alkanes
		F	1–10	1–4	27	C_1_–C_10_ fluoroalkanes
haloalkanes	HAL	C	1–10	1–4	33	C_1_–C_10_ chloroalkanes
		B	1–10	1–4	39	C_1_–C_10_ bromoalkanes
		I	1–10	1–4	28	C_1_–C_10_ iodoalkanes
ethers	ROR	O	1–10	1–3	123	C_1_–C_10_ ethers
aldehydes	RCOH	A	1–10	1–2	33	C_1_–C_10_ aldehyde
ketones	RCOR	K	1–10	1–2	85	C_1_–C_10_ ketones
esters	RCOOR	E	1–10	1–2	183	C_1_–C_10_ esters (only formates)
alcohols	ROH	L	1–10	1–3	358	C_1_–C_10_ alcohols
carboxylic acids	RCOOH	D	1–10	1	48	C_1_–C_10_ carboxylic acids
amines	RN	N	1–10	1–2	117	C_1_–C_10_ amines
amides	RCON	M	1–10	1	19	C_1_–C_10_ amides
mixed subfamilies	MIX	S	1–10	1–4	273	C_1_–C_10_ heteropolyfunctional molecules
						
total	–	–	–	–	1516	total over the 11 subfamilies

a The family is defined as the union of 11 non-overlapping subfamilies, representative for alkanes, for nine chemical functional groups, and for hetero-bifunctional compounds of these groups. The acronyms retained for the different subfamilies (also distinguishing halogen types in the HAL subfamily) are further used in the text, tables, and figures. The one-character variant (Char) is used as a first letter in the acronyms of the corresponding molecules. For each subfamily, *n* stands for the number of carbon atoms, *m* for the number of occurrences of the functional group in the molecule (or total for the two types of group in the MIX subfamily), and *N*^sim^ for the number of isomers considered in the simulations (i.e., for which experimental data could be found). The structures of the *N*_iso_^cal^ = 1516 representative molecules considered in the calibration are shown in Section S.2 in the Supporting Information (Figure S.2).

For the experimental data collection, the database (DBS) maintained in our group was queried for the liquid density ρ_liq_, the vaporization enthalpy Δ*H*_vap_, the surface-tension coefficient γ, the static relative dielectric permittivity ϵ, and the self-diffusion coefficient *D*. The data sources accessed were refs (74−87). This resulted in values concerning *N*_iso_^cal^ = 1516 compounds. The structures of these compounds are shown in Section S.2 in the Supporting Information (Figure S.2). The acronyms employed for the individual molecules involve one letter and four digits. The letter represents the chemical functional group (see Table 2). The first digit stands for the number of carbon atoms, with the number 10 mapped to the digit zero. The last three digits form a sequential index to further distinguish compounds for which the first two symbols are identical.

The experimental-data vector **X**^exp^ used to calibrate the force-field parameters has the dimension *N*_exp_^cal^ = 2044. It encompasses *N*_ρ_^cal^ = 1440 values for ρ_liq_ and *N*_Δ*H*_^cal^ = 604 values for Δ*H*_vap_, and requires *N*_sim_^cal^ = 1607 independent simulations (i.e., distinct compounds and *P*,*T*-points) for its evaluation. The experimental reference values retained for ρ_liq_ and Δ*H*_vap_, along with the associated *P*,*T*-points, are listed in Section 3 in the Supporting Information (see Table S.2). After calibration based on ρ_liq_ and Δ*H*_vap_, experimental values of γ, ϵ, and *D* for 66 compounds were used to further test the accuracy of the optimized force fields. These values are listed in Section S.3 in the Supporting Information (See Table S.3). All the experimental data can also be freely downloaded from the Internet using ref (113), where the present data are labeled as version 1.0.

The force-field representation employed is compatible with the GROMOS force field^88−93^ in its 2016H66 variant,^94^ except for one important difference. The atomic partial charges are determined for each molecule based on an electronegativity equalization (EE) scheme.^95^ Similar to our previous work^22^ (see Appendix A.4 therein), charge flows are only allowed within overall neutral charge groups, and intramolecular Coulombic effects (*J*-terms in the EE scheme) are only included for first and second covalent neighbors.

The covalent interaction parameters relevant for the molecules considered here were taken or ported by analogy from the 2016H66 parameter set^94^ and kept unaltered. The corresponding information is summarized in Section S.4 in the Supporting Information (see Table S.4). Only the nonbonded interaction parameters were subjected to refinement and solely a subset thereof.

**Figure 1:**
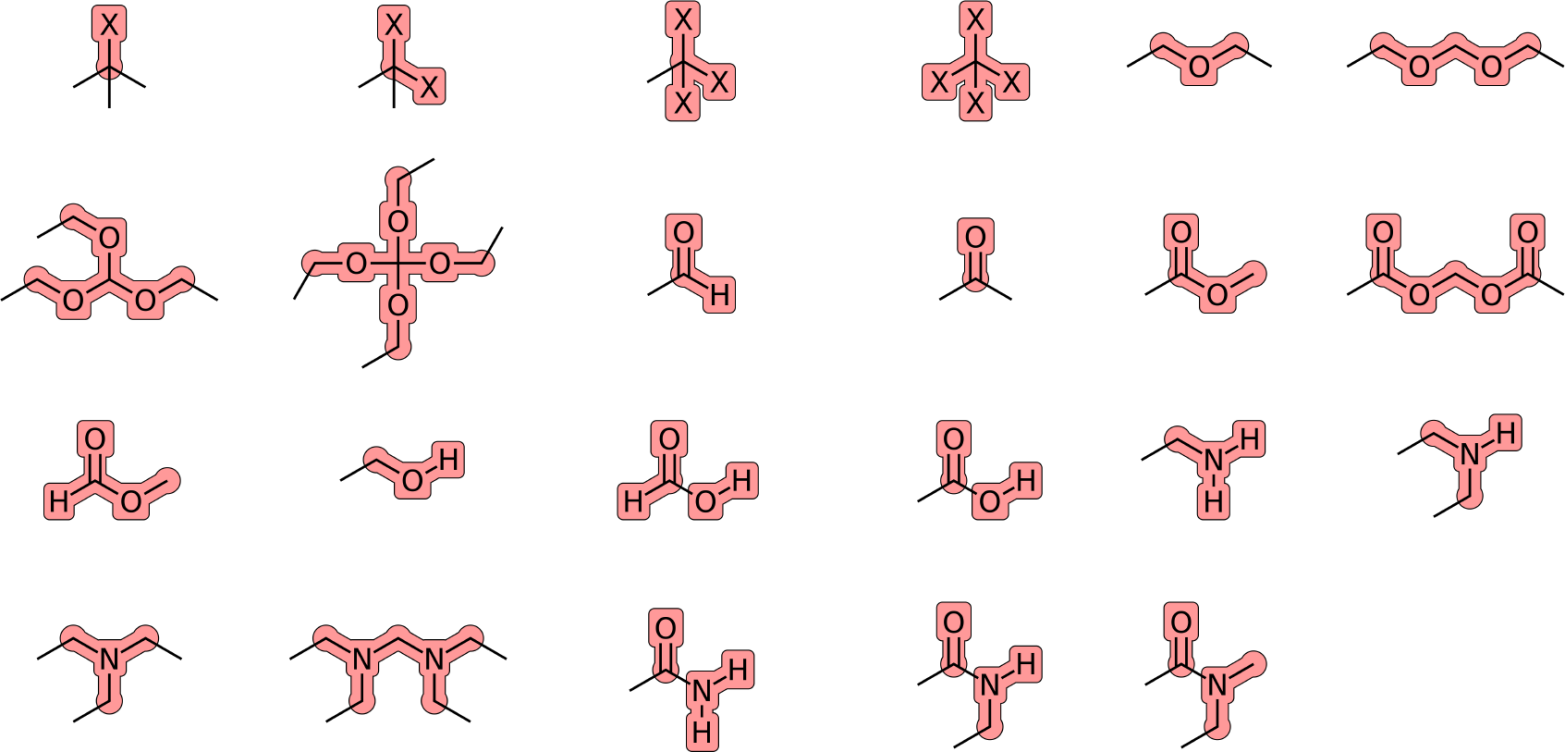
Charge groups relevant for the compounds considered here. The symbol X denotes a halogen atom. Charge flows in the EE scheme are only permitted between atoms belonging to the same overall neutral charge group. All the aliphatic (united) atoms of the molecule (atom types CH0, CH1, CH2, CH3, and CH4 in Table 3) that are not explicitly included in these groups define separate one-particle charge groups with a charge of zero.

In GROMOS, charge groups are used for the application of the nonbonded interaction cutoff, which performs a group-based truncation in terms of the centers of geometry of the two charge groups. The relevant charge groups are illustrated in Figure 1. All the aliphatic (united) atoms of the molecule that are not explicitly included in one of these groups define separate one-particle charge groups with zero charge. Intramolecular Coulombic effects between first and second covalent neighbors within a charge group in the EE scheme are described using Gaussian-cloud interactions. The corresponding effective interatomic distances *r̅* are calculated based on the reference bond lengths and angles of the covalent force field, along with effective radii set to the van der Waals radii of the involved (united) atoms. These radii are listed in Section S.4 (see Table S.5).

**Figure 2:**
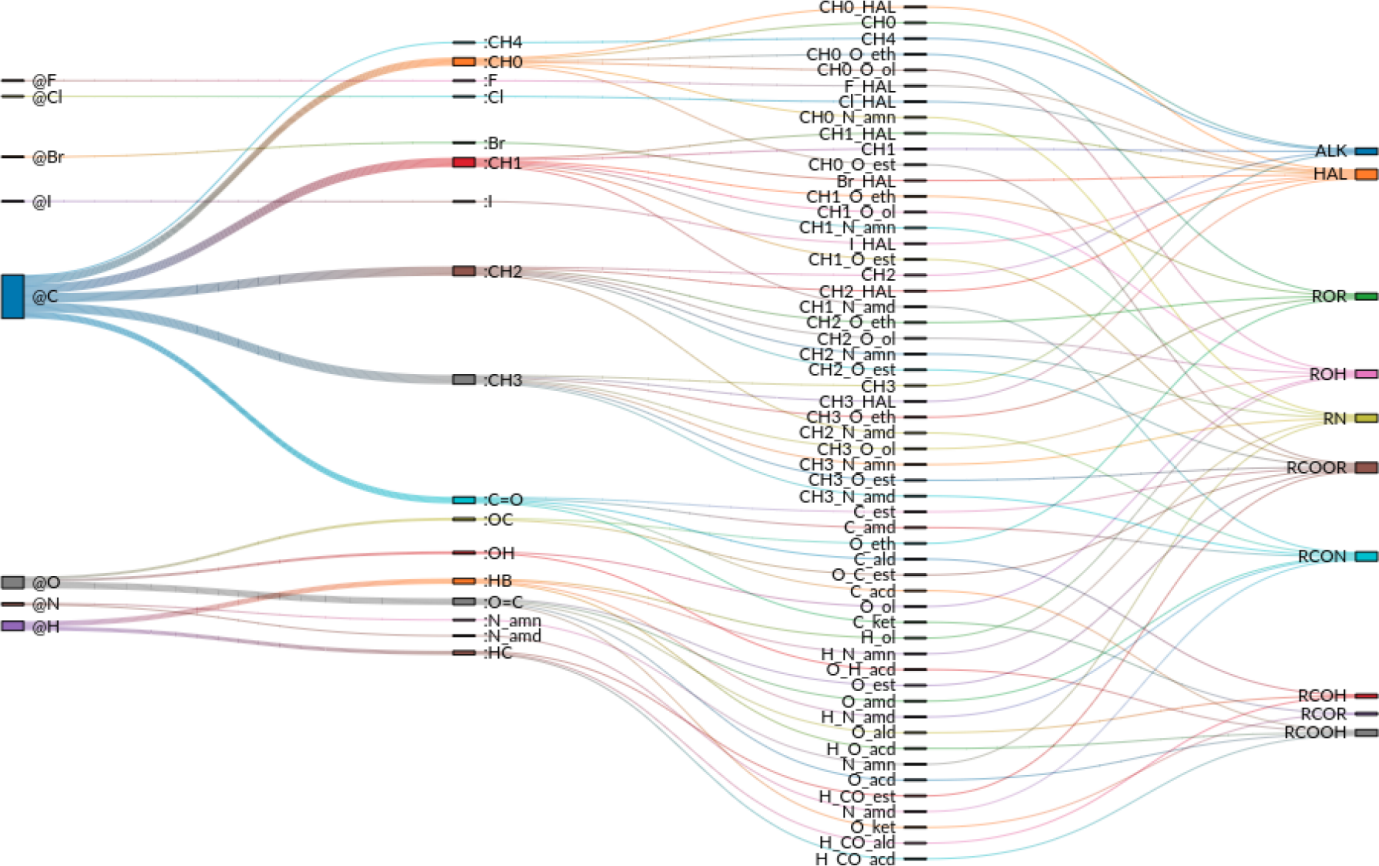
Correspondence between the 8 elements, the 17 LJ types, the 56 atom types (EE types), and the 10 chemical functional groups. The first column refers to the elements, the second to the LJ types (see Table 4), the third to the atom or EE types (see Table 3), and the fourth to the chemical functional groups (see Table 2).

The atomic partial charges are determined indirectly via the EE scheme based on two types of atomic parameters: the hardness η and the electronegativity χ. Because of the use of a combination rule (GM, LB, or WH), the pairwise LJ coefficients are also constructed based on two types of atomic parameters, namely, the collision diameter σ and the well depth ϵ. Following the GROMOS design principles, the values σ and ϵ are only used in the combination rule for non-hydrogen-bonding LJ-type pairs (corresponding to the LJ parameters *C*_6_ and *C*_12,*I*_ in GROMOS). For hydrogen-bonding LJ-type pairs, GROMOS relies on a modified set of LJ parameters with slightly enhanced repulsion. In this case, alternative values σ̃ and ϵ̃ are used instead (corresponding to the LJ parameters *C*_6_ and *C*_12,*II*_ in GROMOS). For simplicity, the value of the dispersion coefficient *C*_6_ is kept identical in the two sets, so that only σ̃ needs to be specified, while ϵ̃ can be deduced as ϵ̃ = . Finally, for third covalent neighbors, yet another pair of values σ* and ϵ* is used in the combination rule. Each atom type of the force field is thus associated with a unique selection for six (non-hydrogen-bonding type) or seven (potentially hydrogen-bonding type) parameters. However, the same σ and ϵ parameters are often used for different atom types of the same element. As a result, the present force field relies on a number *N*_att_ = 56 of atom types, which are equivalent to EE types , but involve a smaller number *N*_att_^LJ^ = 17 of LJ types. The 56 atom types (or EE types) are listed in Table 3 along with a LJ type. The latter refers to the entries of Table 4. The correspondence between elements, LJ types, atom-types (EE types), and chemical functional groups is illustrated schematically in Figure 2. The final (optimized) values of the EE and LJ parameters are reported in Section S.5 in the Supporting Information (Tables S.6 and S.7) for the three choices of combination rules.

**Table 3:** Atom Types (or, Equivalently, EE Types) of the Force Field^a^

atom type (EE type)	LJ type	usage
**Aliphatic Carbon (United) Atoms**		
CH0	CH0	CH_0_ carbon atom (methanetetryl group)
CH1	CH1	CH_1_ carbon united atom (methanetriyl group)
CH2	CH2	CH_2_ carbon united atom (methylene group)
CH3	CH3	CH_3_ carbon united atom (methyl group)
CH4	CH4	CH_4_ carbon united atom (methane group)
**Halogen**		
F_hal	F	fluorine atom
Cl_hal	Cl	chlorine atom
Br_hal	Br	bromine atom
I_hal	I	iodine atom
CH0_hal	CH0	halogenated CH_0_ atom
CH1_hal	CH1	halogenated CH_1_ united atom
CH2_hal	CH2	halogenated CH_2_ united atom
CH3_hal	CH3	halogenated CH_3_ united atom
**Ether**		
O_eth	OC	ether oxygen atom
CH0_O_eth	CH0	alkoxylated CH_0_ atom
CH1_O_eth	CH1	alkoxylated CH_1_ united atom
CH2_O_eth	CH2	alkoxylated CH_2_ united atom
CH3_O_eth	CH3	alkoxylated CH_3_ united atom
**Aldehyde**		
H_CO_ald	HC	aldehyde hydrogen atom
C_ald	C=O	aldehyde carbonyl carbon atom
O_ald	O=C	aldehyde carbonyl oxygen atom
**Ketone**		
C_ket	C=O	ketone carbonyl carbon atom
O_ket	O=C	ketone carbonyl oxygen atom
**Ester**		
H_CO_est	HC	formate ester hydrogen atom
C_est	C=O	ester carbonyl carbon atom
O_est	O=C	ester carbonyl oxygen atom
O_C_est	OC	ester acylated oxygen atom
CH0_O_est	CH0	ester oxygen-linked CH_0_ atom
CH1_O_est	CH1	ester oxygen-linked CH_1_ united atom
CH2_O_est	CH2	ester oxygen-linked CH_2_ united atom
CH3_O_est	CH3	ester oxygen-linked CH_3_ united atom
**Alcohol**		
H_ol	HB	hydroxyl hydrogen atom
O_ol	OH	hydoxyl oxygen atom
CH0_O_ol	CH0	hydroxylated CH_0_ atom
CH1_O_ol	CH1	hydroxylated CH_1_ united atom
CH2_O_ol	CH2	hydroxylated CH_2_ united atom
CH3_O_ol	CH3	hydroxylated CH_3_ united atom
**Carboxylic Acid**		
H_CO_acd	HC	formic acid hydrogen atom
C_acd	C = O	carboxylic acid carbonyl carbon atom
O_acd	O = C	carboxylic acid carbonyl oxygen atom
H_O_acd	HB	carboxylic acid hydroxyl hydrogen atom
O_H_acd	OH	carboxylic acid hydroxyl oxygen atom
**Amine**		
H_N_amn	HB	amine hydrogen atom
N_amn	N_amn	amine nitrogen atom
CH0_N_amn	CH0	aminated CH_0_ atom
CH1_N_amn	CH1	aminated CH_1_ united atom
CH2_N_amn	CH2	aminated CH_2_ united atom
CH3_N_amn	CH3	aminated CH_3_ united atom
**Amide**		
H_N_amd	HB	amide nitrogen-linked hydrogen atom
C_amd	C = O	amide carbonyl carbon atom
O_amd	O = C	amide carbonyl oxygen atom
N_amd	N_amd	amide acylated nitrogen atom
CH0_N_amd	CH0	amide nitrogen-linked CH_0_ atom (estimated)
CH1_N_amd	CH1	amide nitrogen-linked CH_1_ united atom
CH2_N_amd	CH2	amide nitrogen-linked CH_2_ united atom
CH3_N_amd	CH3	amide nitrogen-linked CH_3_ united atom

a The *N*_att_ = 56 atom types (or, equivalently, *N*_att_^EE^ = 56 EE types) are listed, along with their usage and the associated LJ type (referring to the entries of Table 4). Initial (optimization start) and final (after optimization) values for these parameters can be found in Tables S.9 and S.7, respectively, in the Supporting Information.

**Table 4:** LJ Types of the Force Field^a^

LJ type	usage
**Carbon**	
CH0	CH_0_ carbon atom (methanetetryl group)
CH1	CH_1_ carbon united-atom (methanetriyl group)
CH2	CH_2_ carbon united-atom (methylene group)
CH3	CH_3_ carbon united-atom (methyl group)
CH4	CH_4_ carbon united-atom (methane group)
C=O	carbonyl carbon atom
**Halogen**	
F	fluorine atom
Cl	chlorine atom
Br	bromine atom
I	iodine atom
**Oxygen**	
OC	ether oxygen atom
O=C	carbonyl oxygen atom
OH	hydoxyl oxygen atom
**Nitrogen**	
N_amn	amine nitrogen atom
N_amd	amide nitrogen atom
**Hydrogen**	
HC	carbonyl-linked hydrogen atom
HB	oxygen- or nitrogen-linked hydrogen atom

a The *N*_att_^LJ^ = 17 LJ types are listed along with their usage. These LJ types are invoked in the specification of the *N*_att_ = 56 atom types of Table 3. Initial (optimization start) and final (after optimization) values for these parameters can be found in Tables S.8 and S.6, respectively, in the Supporting Information.

The five aliphatic atom types (CH0 to CH4) have no EE parameters, as their charge is always zero. The LJ parameters of the polar hydrogen atom type (HB) were also kept at zero and excluded from the optimization. The third-neighbor LJ interaction parameters were kept equal to those of the 2016H66 set^94^ for the GM combination rule, or made equal to the corresponding normal LJ parameters multiplied by 0.8 (σ* = 0.8σ and ϵ* = 0.8ϵ) for the LB and WH combination rules. This modification is inspired by the scaling applied to third-neighbor interactions in the AMBER force field.^96^ Note also that, in the absence of a parametrization target, the η and χ values of the EE-type CH0_N_amd could not be calibrated. The initial parameter values selected to start the optimization are reported in Section S.6 in the Supporting Information (Tables S.8 and S.9). They were taken from the previous application of the CombiFF scheme to the HAL and O+N families^22,52^, complemented when necessary by η and χ, as estimated from ref (95).

Following from the above choices, the present force field involves *N*_prm_^tot^ = 289 parameters, namely, *N*_prm_^cov^ = 104 covalent parameters and *N*_prm_^nbd^ = 185 nonbonded parameters (2 × 56 EE-types + 4 × 12 non-hydrogen-bonding LJ types + 5 × 5 potentially hydrogen-bonding LJ types), among which *N*_prm_^cal^ = 137 are subject to optimization (omitted are 2 × 17 third-neighbor LJ parameters, 2 × 1 LJ parameters for HB, and 2 × 6 EE parameters for aliphatic carbons and CH0_N_amd). Optimizing these parameters against *N*_exp_^cal^ = 2044 experimental data points leads to an observable-to-parameter ratio of ∼15. This ratio is further analyzed for each EE and LJ type separately in Section S.7 in the Supporting Information (Tables S.10 and S.11). A favorable observable-to-parameter ratio is achieved in most cases, although three EE types (CH3_O_ol, H_CO_acd, and CH1_N_amd) only occur in a single representative molecule, and one (CH0_N_amd) is not represented at all.

The search for optimal parameters was performed as in our previous work^22^ (see Appendix A.7 therein), by minimizing an objective function *Q*(**P**; **X**^exp^) of the parameter vector **P** which accounts for the deviation between the simulated-data vector **X**^sim^(**P**) and the reference-data vector **X**^exp^. This function is
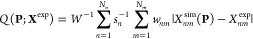
4with
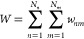
where the index *n* corresponds to the *N*_*n*_ observable types and the index *m* to the *N*_*m*_ molecules in the family. The *s*_*n*_ coefficients eliminate the dependence on a unit system and adjust the relative weights of different observables in terms of the perceived (i.e., subjective) extent of “badness”. They are set here to 20 kg m^–3^ for ρ_liq_ and 1 kJ mol^–1^ for Δ*H*_vap_, i.e., we “decided” that it is equally bad for a force field to be off by 20 kg m^–3^ in terms of ρ_liq_, or to be off by 1 kJ mol^–1^ in terms of Δ*H*_vap_. The coefficients *w*_*nm*_ are set to one for all the combinations included (also considering observables at multiple state points).

The iterative parameter adjustments are is performed by assuming that **X**^sim^(**P**) is approximately linear in parameter changes within a small trust region around a reference point **P**° in parameter space, i.e., using the local first-order approximation  to *Q*(**P**; **X**^exp^), defined by
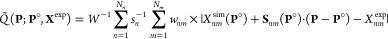
5where **S**(**P**°) is the sensitivity matrix of the different molecule/observable combinations, with respect to variations of the *N*_*k*_ parameters around the point **P**°, i.e.,
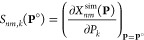
6This matrix is calculated next to the observables themselves during the MD simulations at **P**° using appropriate statistical-mechanical expressions.^22,30,38−40,97^ The trust region is defined here in terms of maximal allowed relative changes in each of the parameters over an iteration, set to 5% for all parameters optimized. Note that the MD simulations and the parameter changes are performed sequentially, not simultaneously. During the simulations, ρ_liq_ and Δ*H*_vap_ are calculated and averaged, as well as the first derivatives of these observables with respect to all force-field parameters (with the parameters remaining fixed). Only after the simlations is a parameter adjustments undertaken.

In practice, the optimization algorithm involves the following steps over iterations *i* starting from zero: (1) select an initial guess **P**_0_^°^ for the parameters; (2) run *N*_sim_^cal^ simulations to get the vector **X**_*i*_^sim,°^ and the matrix; (3) calculate the real value  of the objective function at this point in parameter space using eq 4; (4) minimize  in eq 5 with respect to **P** starting from **P**_*i*_^°^ and staying within the trust region, leading to **P**_i_^*^; (5) calculate the predicted value  of the objective function; (6) set **P**_*i*+1_^°^ to **P**_i_^*^, increment *i*, and iterate to step (2) until convergence.

Convergence can be defined by an objective function that stops varying significantly upon further iteration, or by force-field parameters that also stop varying significantly upon further iteration. The two options will not differ much in terms of the accuracy reached for ρ_liq_ and Δ*H*_vap_ (because it is what is measured by the objective function), but they may differ when other properties are calculated based on the optimized force fields (because their accuracy depends on the parameters retained). In this work, we took the first definition. Previous work involving multiple optimizations initiated from different starting parameters^22^ (see Section S.8 in the Supporting Information of this article) have shown that (i) different solutions of similar accuracies are obtained; (ii) the corresponding EE parameters evidence significant variations, while the LJ parameters and EE-derived partial charges are more similar. The force-field variants derived in this work are thus probably close to optimality, but not unique.

The full parameter optimization was performed twice for GM and LB, or three times for WH. The second optimization (and third for WH) were carried out with randomly perturbed parameters from the final parameters of the first optimization (changes in the range 20%, or 50% for the third run with WH). These repeats served to assess the robustness of the calibration with respect to variations of the initial parameters. Only the results for the three runs (one for each combination rule) leading to the minimum value of the target function are discussed in the main article. The results for the other runs are reported in Section S.13 in the Supporting Information.

The optimization against ρ_liq_ and Δ*H*_vap_ was performed as in our previous work^22,52^ using an in-house GROMOS-compatible simulation engine in C++ called SAMOS. The GROMOS program^92,98^ was used for the calculation of all other properties. The pure-liquid MD simulations were carried out under periodic boundary conditions based on cubic computational boxes containing 512 molecules. They were performed in the isothermal–isobaric ensemble at the reference pressures *P* and temperatures *T* listed in Tables S.2 and S.3 in the Supporting Information.

The equations of motion were integrated using the leapfrog scheme^99,100^ with a time step of 2 fs. Constraints on all bond lengths were enforced using the SHAKE procedure^101^ with a relative geometric tolerance of 10^–5^. The nonbonded interactions were calculated using a twin-range scheme^102^ based on charge-group distances, with short- and long-range cutoff radii set to 0.8 and 1.4 nm, respectively, and an update frequency of 5 timesteps for the short-range pairlist and intermediate-range interactions. The mean effect of the omitted electrostatic interactions beyond the long-range cutoff was reintroduced by means of a reaction-field correction.^103,104^ The corresponding static relative dielectric permittivities were set to the experimental permittivity ϵ. The temperature was maintained close to its reference value using a Nosé–Hoover thermostat^105^ with a coupling time of 0.1 ps, and the pressure was maintained close to its reference value using a weak-coupling barostat^106^ with a coupling time of 0.5 ps and an isothermal compressibility set to 4.575 · 10^–4^ kJ^–1^ mol nm^3^. The ideal-gas simulations (required for Δ*H*_vap_) involved a single molecule and relied on stochastic dynamics^100,107−110^ (SD) with a friction coefficient set to 2 ps^–1^.

For the calculation of the five monitored properties (ρ_liq_, Δ*H*_vap_, γ, ϵ, and *D*), three independent repeats were carried out each time, involving different initial coordinates and velocities. The average value is reported, along with an uncertainty estimate corresponding to the error on the mean over the three repeats with a 95% confidence interval.

The pure-liquid density ρ_liq_ and vaporization enthalpy Δ*H*_vap_ were calculated based on pure-liquid and ideal-gas simulations at *P*,*T*-points specified in Table S.2. For ρ_liq_, this required only a pure-liquid simulation at the indicated *P*,*T*-point. For Δ*H*_vap_, this also required an ideal-gas simulation at the same temperature *T*. For each repeat, the former simulation involved 0.6 ns equilibration followed by 0.6 ns production. The value of ρ_liq_ was calculated from the pure-liquid simulation as the ratio of the mass of the computational box to the corresponding average volume. The value of Δ*H*_vap_ was calculated from the pure-liquid and gas-phase simulations as the difference between the average potential energies per molecule in the two phases (gas minus liquid), expressed on a per-mole basis and increased by *RT*, where *R* is the gas constant.

The surface-tension coefficient was calculated at the *P*,*T*-points listed in Table S.3. For each of the three repeats, the system was first equilibrated for 5 ns at constant pressure. The box was then extended by a factor of 5 in the *z*-direction, generating a system with two liquid-vacuum interfaces. The value of γ was calculated from a subsequent 5 ns constant-volume simulation as
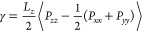
7where *L*_*z*_ is the box length in the *z*-direction and *P*_αα_ (α = *x*, *y*, *z*) are the diagonal elements of the pressure tensor.

The static relative dielectric permittivity ϵ was calculated at the *P*,*T*-points listed in Table S.3. For each of the three repeats, ϵ was obtained from a 50 ns constant-pressure simulation using the Neuman relation,^111^
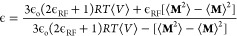
8where **M** is the box dipole moment vector, ϵ_o_ is the permittivity of vacuum, and ϵ_RF_ is the reaction-field permittivity (Table S.2).

The self-diffusion coefficient *D* was calculated at the *P*,*T*-points listed in Table S.3. For each of the three repeats, it was obtained based on a 5.0 ns constant-pressure simulation from the mean-square displacement of the molecules, using the Einstein relation^112^
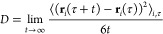
9where **r**_*i*_ is the instantaneous position of the center of geometry of molecule *i*, following molecules across periodic boundaries. The estimation of *D* relied in practice on a least-squares fit over the interval from 0 to 3 ns.

Additional details about the simulation protocols can be found in refs (22 and 52). The GROMOS-compatible molecular topologies and equilibrated liquid configurations for the *N*_iso_^cal^ = 1516 molecules considered here can be downloaded from the Internet under ref (113) (version 1.0).

## 3 Results

**Figure 3:**
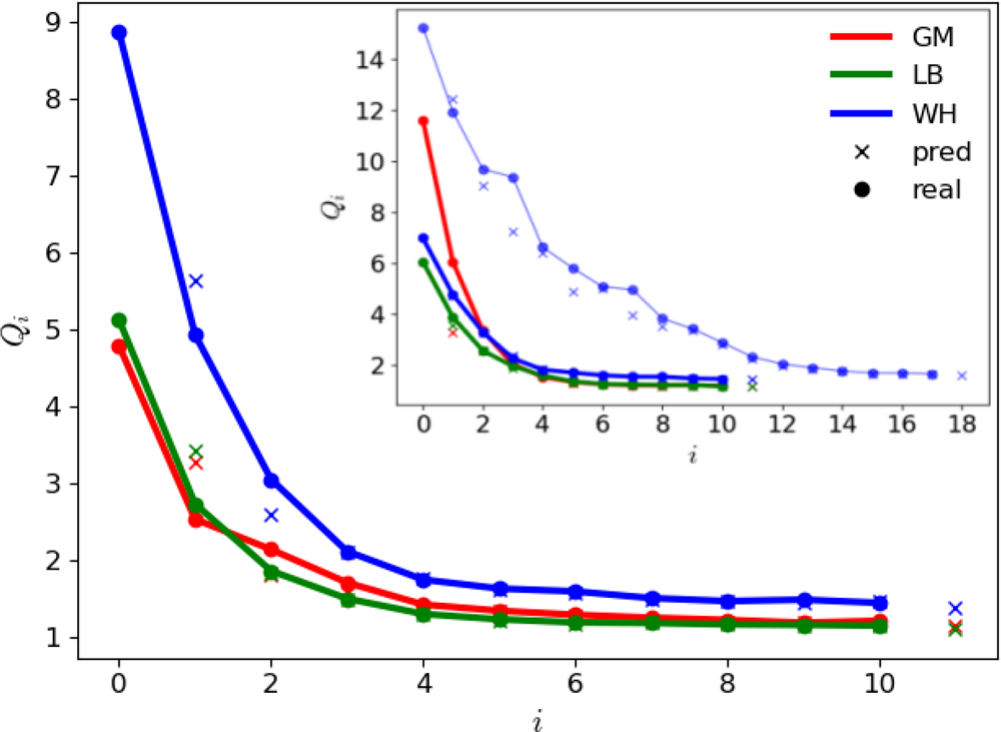
Evolution of the predicted (pred) and real (real) values of the objective function *Q* against the iteration number *i* along the force-field parameter optimization for geometric-mean (GM), Lorentz–Berthelot (LB), and Waldman–Hagler (WH) combination rules. The main graph shows the optimizations that led to the lowest final *Q* values. The inset shows the other optimizations (one for GM and LB, two for WH). For the main runs, 34, 7, and 17 molecules vaporized when optimizing with the GM, LB, and WH combinations rules, respectively. These are displayed in Section S.8 in the Supporting Information. The results discussed in the text exclude the set of 69 molecules that vaporized in any of these runs. Note that the lines have no physical meaning and are intended only as a guide to the eye.

The evolution of the objective function *Q* against the iteration number *i* is illustrated in Figure 3 for the three combination rules. The main graph shows the optimizations that led to the lowest final *Q* values. The inset shows the other optimizations (one for GM and LB, two for WH). The real values *Q*_*i*_^real^ at iteration *i* as well as their predicted values *Q*_*i*_^pred^ from iteration *i* – 1 are both shown. The objective function drops sharply during the first two iterations, converges after about four iterations (where *Q*^pred^ and *Q*^real^ become almost identical), and the additional iterations bring only little further improvement. The three final force-field variants corresponds to iteration *i* = 10 for the main replica, with final values of 1.18, 1.15, and 1.45 for the objective function in the GM, LB, and WH cases, respectively. The final values for the alternative replicas are 1.21, 1.16, and 1.45 for the GM, LB, and WH combination rules. The third run using WH, which involved a more pronounced randomization of the initial parameters, gives a final value of 1.64. The value of *Q*_*i*_^real^ using WH is always the highest, whereas the values for GM and LB are lower and closer together. For the sake of conciseness, only the three force-field variants with the lowest final *Q* value are further discussed here. The results for the other replicas evidence the same qualitative features, and are reported in Section S.13 in the Supporting Information for completeness.

**Figure 4:**
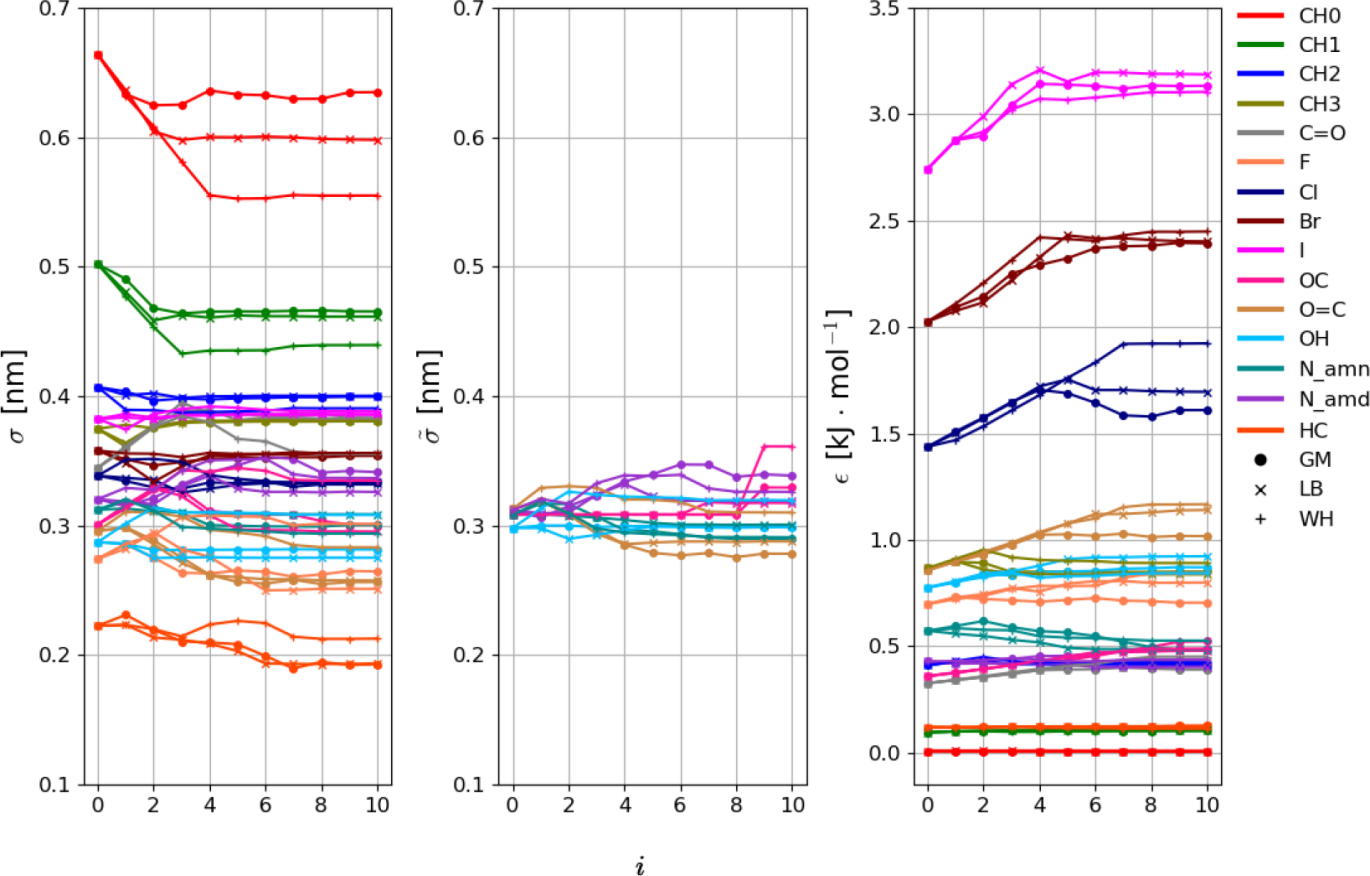
Evolution of the 37 LJ interaction parameters against the iteration number *i* along the force-field parameter optimization for the geometric-mean (GM), Lorentz–Berthelot (LB), and Waldman–Hagler (WH) combination rules. The parameters considered are the collision diameter σ or σ̃ (the latter is applicable for hydrogen-bonding types) and the well depth ϵ. The *N*_att_^LJ^ = 17 LJ types are listed in Table 4. The final parameter values are reported numerically in Table S.6 in the Supporting Information. Note that the parameters σ̃ are only relevant for potentially hydrogen-bonding LJ types (5 types), and that the LJ-type HB is omitted from the graph (σ and ϵ set to zero). The results with the alternative replicas are shown in Section S.13 in the Supporting Information. Note that the lines have no physical meaning and are intended only as a guide to the eye.

**Figure 5:**
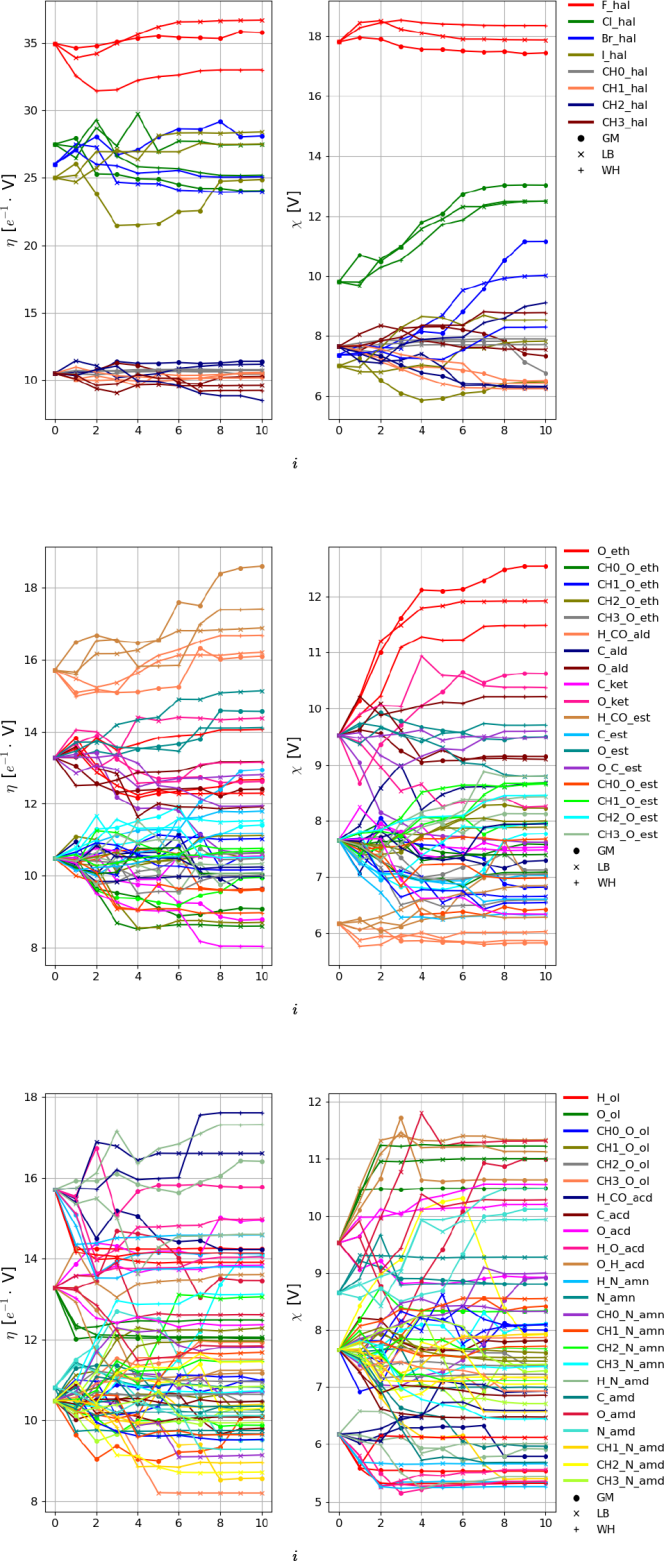
Evolution of the 100 EE interaction parameters against the iteration number *i* along the force-field parameter optimization for geometric-mean (GM), Lorentz–Berthelot (LB), and Waldman–Hagler (WH) combination rules. The parameters considered are the electrostatic hardness η and electronegativity χ. The *N*_att_^EE^ = 56 EE types are listed in Table 3. The final parameter values are reported numerically in Table S.7 in the Supporting Information. Note that the aliphatic united-atom EE types are omitted from the graph (5 types with zero charge) as well as the EE type CH0_N_amd (no representative molecule for calibration). The results with the alternative replicas are shown in Section S.13 in the Supporting Information. Note that the lines have no physical meaning and are intended only as a guide to the eye.

**Figure 6:**
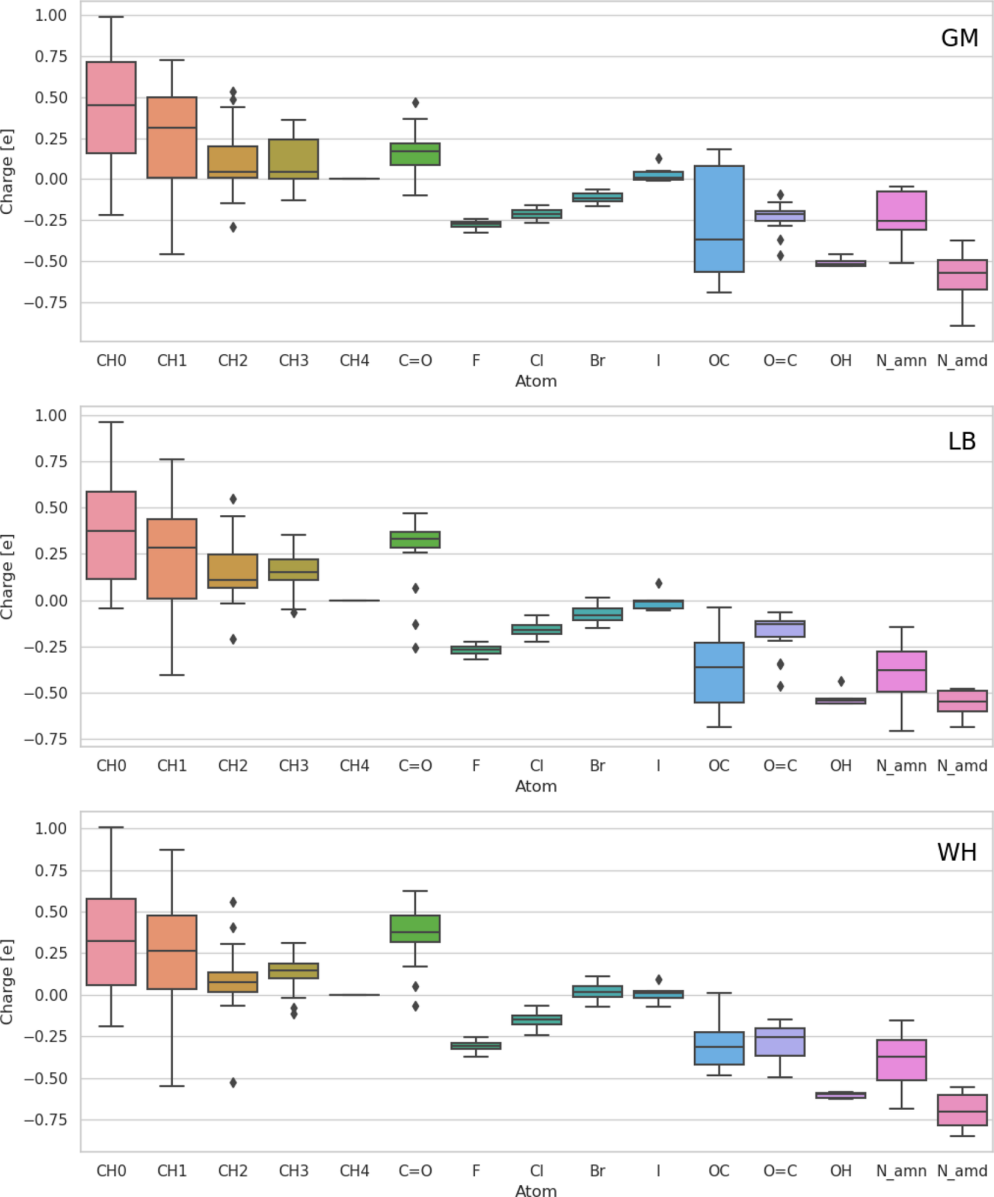
Distribution of the EE-derived atomic partial charges for the different LJ types using the GM (top), LB (middle), and WH (bottom) combination rules. The boxes show the minimum, first quartile, median, third quartile, maximum, and outlier values of the distribution. Each possible value of the charge is only counted once in the distribution (irrespective of the number of molecules in which the particular charge occurs). The *N*_att_^LJ^ = 17 LJ types are listed in Table 4.

The evolution of the *N*_prm_^cal^ = 137 nonbonded interaction parameters subject to calibration against the iteration number *i* is shown in Figures 4 and 5 for the LJ and EE parameters, respectively. The LJ interaction parameters σ and ϵ tend to converge to similar final values for the three combination rules, i.e., within relatively narrow ranges. The exceptions are the atom types CH0, CH1, and Cl. Note that CH0 is a special case, as it is a buried atom type. Compared to the LJ interaction parameters, the electrostatic parameters η and χ evidence more variations across the three optimizations. However, as shown in Figure 6, the atomic partial charges remain qualitatively consistent across the optimizations with the different combination rules. The charges tend to be positive for hydrogen and carbon atom types, and predominantly negative for oxygen and nitrogen types. Exceptionally, the alkoxyl-oxygen atom type (OC) in ester groups may present positive charges when using GM. This is because the electronegativity of the oxygen type O_C_est (7.6 V) is lower than of alkoxyl-carbon atom types (8.0 V for CH1_O_est, 7.8 V for CH2_O_est, and 8.1 V for CH3_O_est). Only when bound to CH0_O_est (6.4 V) is the alkoxyl-oxygen in ester groups negative. The corresponding atomic partial charges are shown in Section S.9 in the Supporting Information (Figure S.7) and compared to QM-derived charges.^114−118^

**Figure 7:**
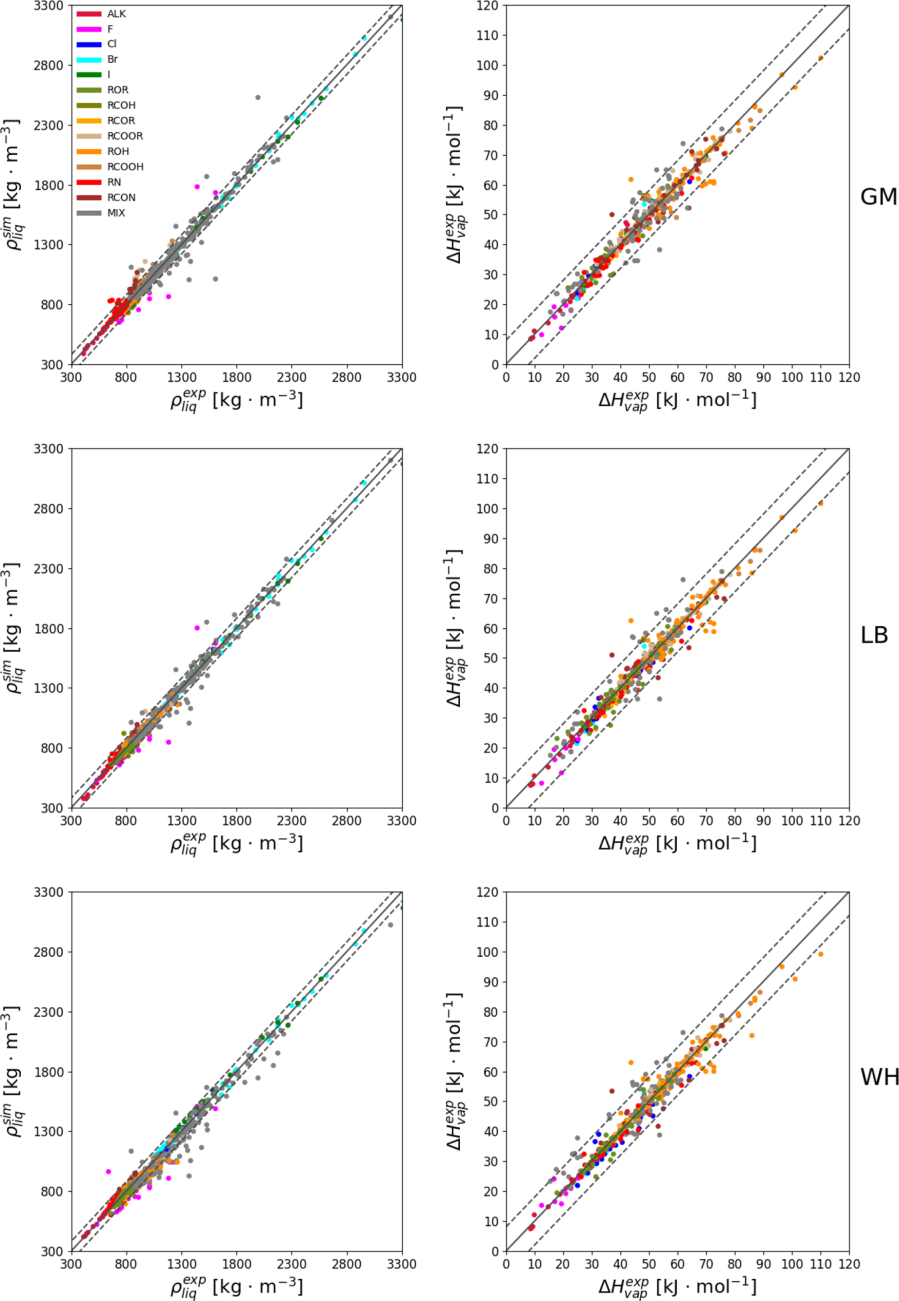
Comparison of simulated and experimental properties based on the optimized force field for the GM (top), LB (middle), and WH (bottom) combination rules. The diagonal solid lines and the two parallel dashed lines indicate perfect agreement within ±80 kg m^–3^ for ρ_liq_ (left) or ±8 kJ mol^–1^ for Δ*H*_vap_ (right). The corresponding numerical values are reported in Tables S.12–S.14 in the Supporting Information.

**Figure 8:**
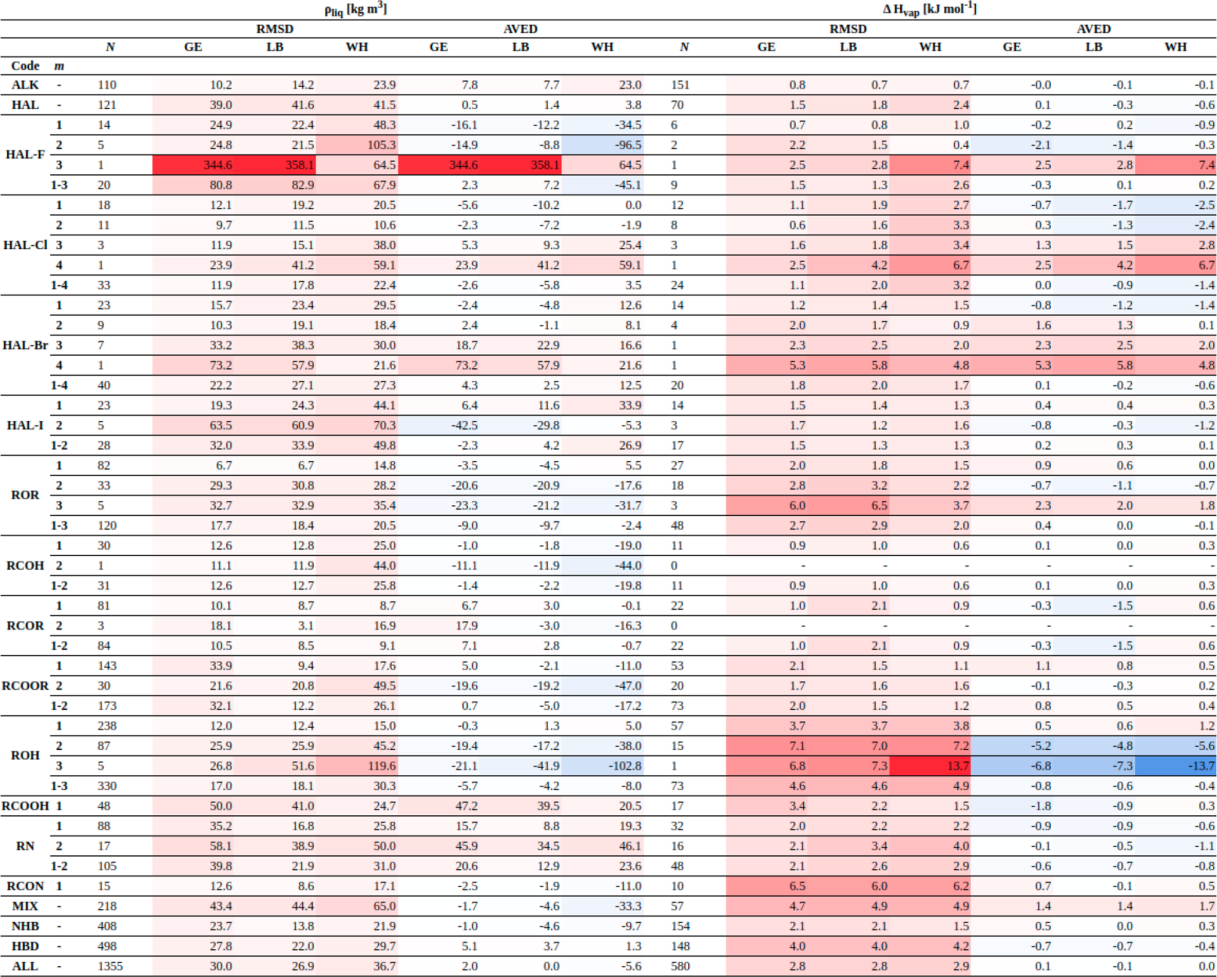
Statistics concerning the discrepancies between simulated and experimental properties with the GM, LB, and WH combination rules using a set of 1447 molecules. The results are reported separately for the different chemical functions as listed in Table 2. Four classes of compounds are also considered separately, namely, the compounds with two different functional groups (MIX), the halogenated (HAL) ones, the non-hydrogen-bonding (NHB) ones (including ethers, ketones, aldehydes, and esters), and the hydrogen-bonding (HBD) ones (including alcohols, carboxylic acids, amines, and amides), along with the entire set of molecules (ALL). The root-mean-square deviation (RMSD) and average deviation (AVED) values are reported in terms of ρ_liq_ (left) and Δ*H*_vap_ (right). The value of *m* indicates to the number of functional groups and *N* refers to the number of data points considered. The color coding underlines the sign and magnitude of the discrepancies.

The level of agreement between the optimized force fields and experiment in terms of ρ_liq_ and Δ*H*_vap_ for each combination rule is illustrated in Figure 7. The corresponding numerical values can be found in Section S.10 in the Supporting Information (Tables S.15–S.17). The statistics per compound types are provided in Figure 8 for the three combination rules. In these statistics, four classes of compounds are also considered separately, namely, the compounds with two different functional groups (MIX), the halogen (HAL) ones, the non-hydrogen-bonding (NHB) ones (including ethers, ketones, aldehydes, and esters), and the hydrogen-bonding (HBD) ones (including alcohols, carboxylic acids, amines, and amides).

Considering ρ_liq_, the overall agreement with experiment in terms of root-mean-square deviation (RMSD) is good for the three combination rules, with values of 30.0, 26.9, and 36.7 kg m^–3^ for GM, LB, and WH, respectively. Overall, the results with LB are the most accurate, but this is not always true when considering the compound families separately. For example, the GM combination rule is generally more accurate for the halohydrocarbons. On the other hand, the results with WH are slightly less accurate compared to GM and LB, not only overall but also in terms of the different families. In particular, for the alkanes with WH, the RMSD is significantly higher (23.9 kg·m^–3^) than with the other combination rules (10.2 and 14.2 kg m^–3^). The only exceptions are trifluoromethane, tetrabromomethane, and carboxylic acids, for which WH performs slightly better.

In terms of Δ*H*_vap_, the three combination rules perform comparably well with overall RMSD values of 2.8, 2.8, and 2.9 kJ mol^–1^ for GM, LB, and WH, respectively. A similar observation also applies to the results for the individual families, where there is no significant difference between the combination rules, irrespective of the chemical functional group considered.

Although the agreement with experiment is good for most molecules (Figure 7), there are outliers for the three combination rules. The corresponding structures (with deviations higher than 80.0 kg m^–3^ for ρ_liq_ and/or higher than 8.0 kJ mol^–1^ for Δ*H*_vap_) are depicted in Section S.11 in the Supporting Information (Figures S.8–S.10). These molecules are predominantly tri- and tetrafluoromethane, diamines, diols, and small compounds with two distinct functional groups. Note that the number of such outliers is lowest when using the LB combination rule (61), compared to that observed for the GM rule (105) and the WH rule (91).

**Figure 9:**
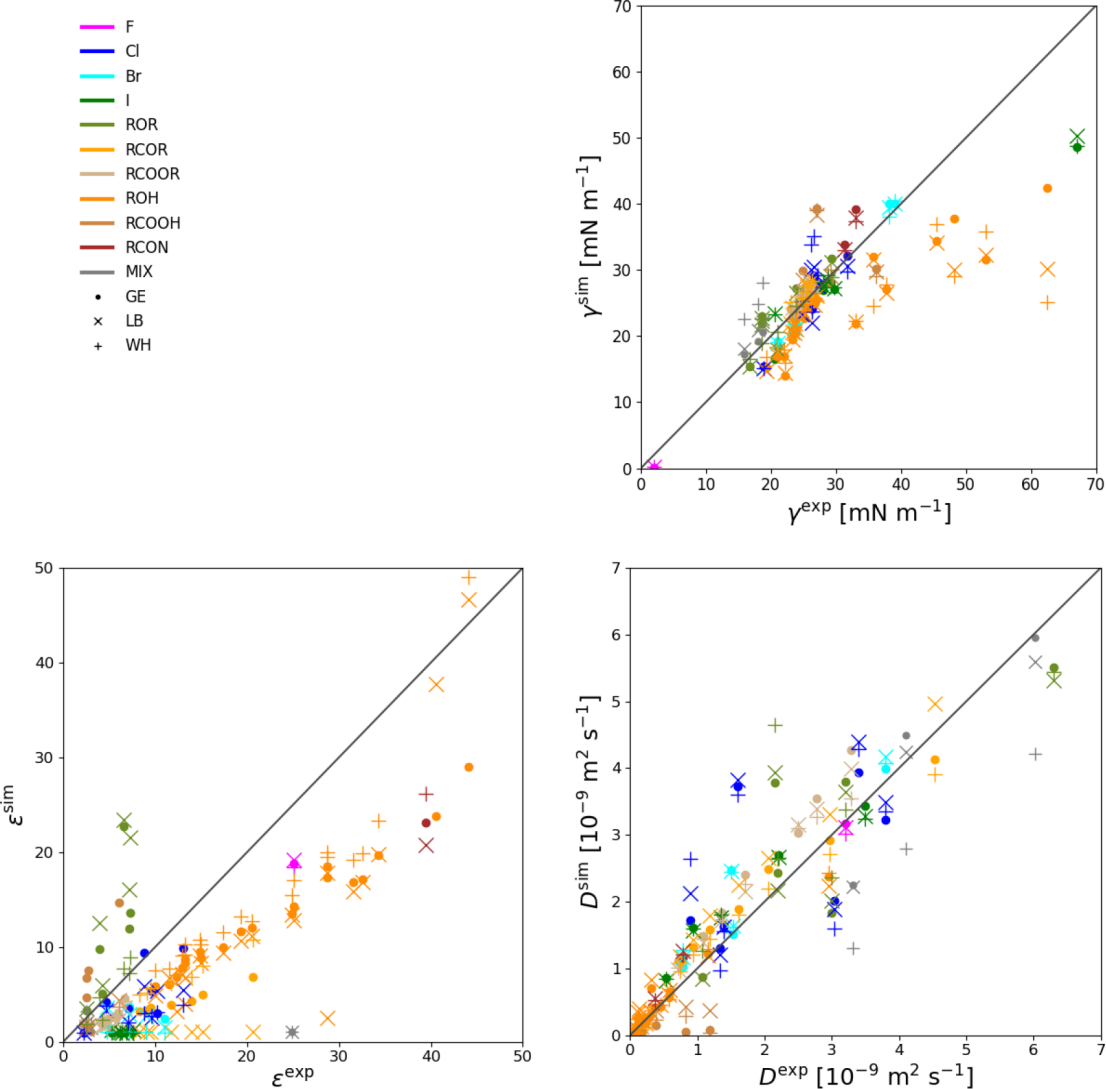
Comparison of the simulated and experimental surface-tension coefficient (γ), static relative dielectric permittivity (ϵ), and self-diffusion coefficient (*D*), based on the force-field variants optimized using the GM, LB, and WH combination rules. The corresponding numerical values are reported in Tables S.15–S.17.

The level of agreement with experiment in terms of γ, ϵ, and *D* for each combination rule is illustrated in Figure 9. The corresponding numerical values are reported in Section S.10 (Tables S.15–S.17 in the Supporting Information). The statistics for the set of 66 compounds is provided in Table 5 for the three combination rules. The results do not evidence any pronounced systematic effect of the combination rule on these three properties. In terms of the comparison with experiment, however, significant deviations are observed. The errors concerning *D* are significant, but largely nonsystematic. They probably result from an interplay between different effects, such as the use of united atoms (expected to enhance diffusion, depending on their count in a given molecule), and the application of a cutoff with a reaction-field correction for the long-range electrostatic interactions in the absence of a correction for the long-range Lennard-Jones interactions (which may affect diffusion in different ways for different molecules). The errors concerning ϵ and γ are also significant, and now somewhat systematic. The deviations observed for ϵ, which are predominantly negative (especially for the most polar compounds), likely result from the use of a nonpolarizable force field, i.e., with an implicit treatment of the electronic-polarization effects. The deviations observed for γ, which predominantly occur at high γ and are then negative, likely result from the use of a mean-field treatment of the electrostatic interactions beyond the cutoff (reaction-field correction), i.e., that may not be very accurate/adequate in a heterogeneous environment like a liquid/vacuum interface.

**Table 5:** Statistics Concerning the Discrepancies between Simulated and Experimental Properties with the GM, LB, and WH Combination Rules for 66 Molecules in Terms of Surface-Tension Coefficient (γ), Static Relative Dielectric Permittivity (ϵ), and Self-Diffusion Coefficient (*D*)^a^

	ρ_liq_ [kg m^–3^]		Δ*H*_vap_ [kJ mol^–1^]		γ [mN m^–1^]		ϵ		*D* [ 10^–9^ m^2^ s^–1^]	
combination rule	RMSD	AVED	RMSD	AVED	RMSD	AVED	RMSD	AVED	RMSD	AVED
GM	30.0	2.0	2.8	0.1	5.9	–1.9	19.0	–7.0	0.6	0.1
LB	26.9	0.0	2.8	–0.1	6.8	–2.0	20.9	–7.9	0.6	0.2
WH	36.7	–5.6	2.9	0.0	7.3	–1.5	19.0	–6.5	0.7	0.1

a The results for the 1447 molecules in terms of the experimental liquid densities ρ_liq_, and vaporization enthalpies Δ*H*_vap_ are also shown. The root-mean-square (RMSD) and average (AVED) deviations are reported.

**Figure 10:**
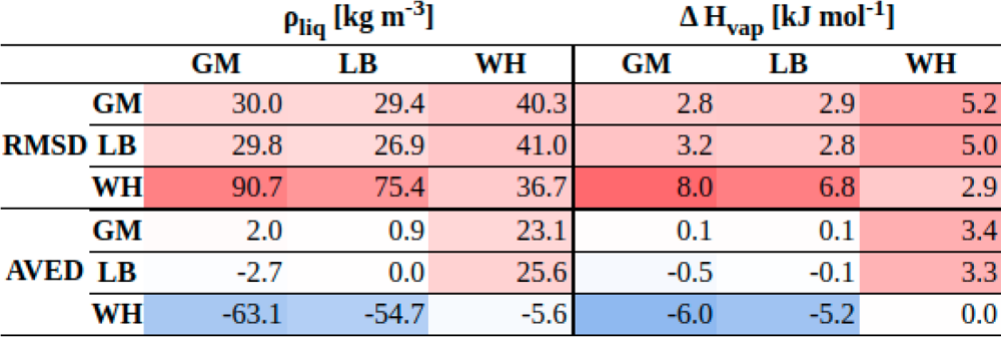
Statistics concerning the discrepancies between simulated and experimental properties considering the interchange of combination rules. Each entry corresponds to the results obtained with the combination rule specified in the given row and the parameters optimized using the combination rule specified in the given column. The root-mean-square deviation (RMSD) and the average deviation (AVED) are reported in terms of ρ_liq_ (left) and Δ*H*_vap_ (right) for the common set of 1447 molecules. The same analysis separated per family of compounds is shown in Figure S.11 in the Supporting Information.

The force-field parameters obtained from the optimization with a given combination rule were also used to carry out simulations with the two other rules, to investigate the effect of a possible mismatch in this choice between force-field calibration and property calculation. The matrix with the statistics concerning ρ_liq_ and Δ*H*_vap_ is shown in Figure 10. The corresponding data sorted by compound families can be found in Section S.12 (Figure S.11 in the Supporting Information). Expectedly, the best agreement with experiment is normally obtained when the force field is used together with the combination rule that was employed in the calibration (diagonal elements of the matrices). However, the exchange between the GM and LB combination rules has a limited effect. For the NHB and HBD groups, using the combination rule with parameters optimized for LB even leads to slightly more accurate results than with the GM parameters optimized for GM. On the other hand, replacing the combination rules GM or LB with WH significantly decreases the accuracy for both ρ_liq_ and Δ*H*_vap_.

## 4 Conclusions

To assess in a fair way the effect of a specific functional-form choice on the intrinsic accuracy of the classical force-field representation, the comparison must ideally be performed at an optimal level of parametrization relative to a given set of molecules, observables, and target values.

Here, we performed such a comparison considering the choice different combination rules for the LJ interactions, namely geometric-mean (GM), Lorentz–Berthelot (LB), and Waldmann–Hagler (WH). This assessment was performed using 2044 experimental liquid densities ρ_liq_ and vaporization enthalpies Δ*H*_vap_ concerning 1516 organic liquids. Three force-field variants (implementing the three alternative combination rules) were optimized independently and automatically against these target data using the CombiFF workflow.

The resulting RMSD values from the experiment are 30.0, 26.9, and 36.7 kg m^–3^ for ρ_liq_ and 2.8, 2.8, and 2.9 kJ mol^–1^ for Δ*H*_vap_, when using the GM, LB, and WH combination rules, respectively. Repeats of the optimizations were also performed, leading to similar deviations, which suggests that the calibrated parameters are close to optimal with respect to the target data. The comparison was then extended to three other properties that were not included as parametrization targets, namely, the surface-tension coefficient (γ), the static relative dielectric permittivity (ϵ), and the self-diffusion coefficient (*D*).

The main observation is that, provided that the parameters are optimized specifically for a given combination rule, the difference between the three rules is rather small. The simulation results with GM and LB are closer together and have a slightly higher accuracy compared to WH, but the effect is not very pronounced.

The slightly lower accuracy of the WH rule may come as a surprise, considering that this rule is demonstrably more accurate than the GM and LB rules for rare gases.^63^ Clearly, the presence of a slight suboptimality in the CombiFF optimization (convergence not entirely reached and/or reaching a local minimum) cannot be excluded. This could lead to a residual dependence of the optimized force field on the initial parameters of the optimization. Since these parameters are taken from GROMOS, which relies on the GM rule, this could penalize WH (and, to a lesser extent, LB). However, it is also possible that the WH rule is more accurate in the context of rare gases, but less accurate in the context of effective interaction functions for condensed-phase systems. In practice, the latter involve numerous approximations (united atoms, atomic partial charges, implicit electronic polarization, cutoff and mean-field corrections) leading to atom–atom interactions differing significantly from those corresponding to isolated pairs of neutral closed-shell noble-gas atoms in the gas phase.

Expectedly, the discrepancies relative to experiment are typically larger when the properties are calculated using a combination rule that differs from the one used in the parameter calibration. The discrepancies are in particular relatively large when the GM or LB rules are substituted by the WH rule.

## Supporting Information Available

Detailed information concerning: (i) the influence of combination rules for rare gases; (ii) the compounds in the calibration set; (iii) the reference experimental data; (iv) the covalent interaction parameters; (v) the final values of the nonbonded parameters; (vi) the initial values of the nonbonded parameters; (vii) the observable-to-parameter ratio; (viii) the list of compounds that vaporized; (ix) the partial charges on oxygen and carbon for esters; (x) the comparison with experiment (experimental vs simulated properties); (xi) the outliers; (xii) the statistics concerning the discrepancies between simulated and experimental properties considering the interchange of combination rules; and (xiii) the results of the alternative replica sets (PDF)
