# Supplementary material for: Influence of the Lennard-Jones Combination Rules on the Simulated Properties of Organic Liquids at Optimal Force-Field Parametrization
Source: J Chem Theory Comput. 2023 Mar 15;19(7):2048–63. doi: 10.1021/acs.jctc.2c01170 (PMC10100539; doi:10.1021/acs.jctc.2c01170)
Supplement: Supplementary file 1 — ct2c01170_si_001.pdf [file ct2c01170_si_001.pdf]

Supplementary Material to

Influence of the Lennard-Jones combination rules on the simulated properties of organic liquids at optimal force-field parametrization

Marina P. Oliveira[†] and Philippe H. Hünenberger[†,*]

Affiliations:

†: Laboratorium für Physikalische Chemie, ETH Zürich, CH-8093 Zürich, Switzerland

S.1 Influence of the Combination Rule for Rare Gases

The present section summarizes the results of applying the different combination rules (GM, LB or WH) in the context of rare gases, based on the data from Ref. 63. The results are reported numerically in Tab. S.1 and graphically in Fig. S.1.

Atoms	σ [Å]							ϵ [° K]				
	Exp	GM	Err	LB	Err	WH	Err	Exp	GM/LB	Err	WH	Err
He-He	2.610	2.610	0.0	2.610	0.0	2.610	0.0	10.440	10.440	0.0	10.440	0.0
He-Ne	2.691	2.682	-0.4	2.683	-0.3	2.687	-0.1	19.440	20.940	7.7	20.667	6.3
He-Ar	3.084	2.957	-4.1	2.980	-3.4	3.087	0.1	30.010	38.435	28.1	29.709	-1.0
He-Kr	3.267	3.053	-6.6	3.091	-5.4	3.258	-0.3	31.050	45.443	46.4	30.791	-0.8
He-Xe	3.533	3.184	-9.9	3.247	-8.1	3.512	-0.6	29.770	53.484	79.7	29.703	-0.2
Ne-Ne	2.755	2.755	0.0	2.755	0.0	2.755	0.0	42.000	42.000	0.0	42.000	0.0
Ne-Ar	3.119	3.038	-2.6	3.053	-2.1	3.122	0.1	64.170	77.091	20.1	65.495	2.1
Ne-Kr	3.264	3.137	-3.9	3.163	-3.1	3.284	0.6	67.320	91.146	35.4	69.131	2.7
Ne-Xe	3.488	3.272	-6.2	3.320	-4.8	3.531	1.2	67.250	107.275	59.5	67.879	0.9
Ar-Ar	3.350	3.350	0.0	3.350	0.0	3.350	0.0	141.500	141.500	0.0	141.500	0.0
Ar-Kr	3.464	3.459	-0.2	3.461	-0.1	3.469	0.2	165.800	167.298	0.9	164.272	-0.9
Ar-Xe	3.660	3.608	-1.4	3.617	-1.2	3.666	0.2	182.600	196.904	7.8	178.935	-2.0
Kr-Kr	3.571	3.571	0.0	3.571	0.0	3.571	0.0	197.800	197.800	0.0	197.800	0.0
Kr-Xe	3.753	3.725	-0.8	3.728	-0.7	3.744	-0.2	225.400	232.803	3.3	225.555	0.1
Xe-Xe	3.885	3.885	0.0	3.885	0.0	3.885	-0.0	274.000	274.000	0.0	274.000	-0.0

Table S.1: Comparison of combination rules in terms of the LJ parameters σ and ϵ for rare gases. The codes refer to experimental values[63] (Exp), percentage error (Err), geometric-mean (GM), Lorentz-Berthelot (LB), and Waldman-Hagler (WH). The results are illustrated graphically in Fig. S.1.

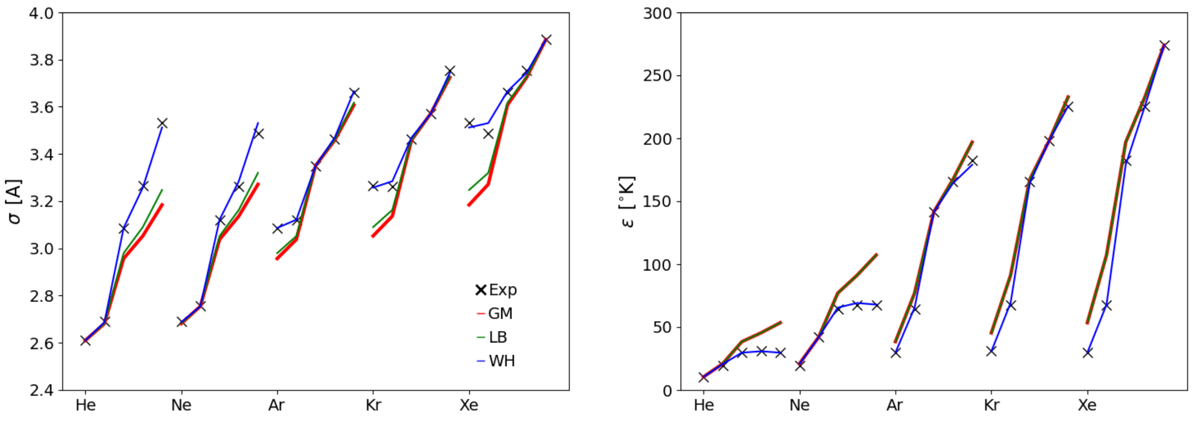

Figure S.1: Graphical representation of the experimental and calculated values using the GM, LB, or WH combination rules for rare gases. The numerical values are reported in Tab. S.1.

S.2 Compounds in the Calibration Set

Figure S.2: Chemical structures of the $N_{\text{iso}}^{\text{cal}} = 1516$ molecules in the calibration set.

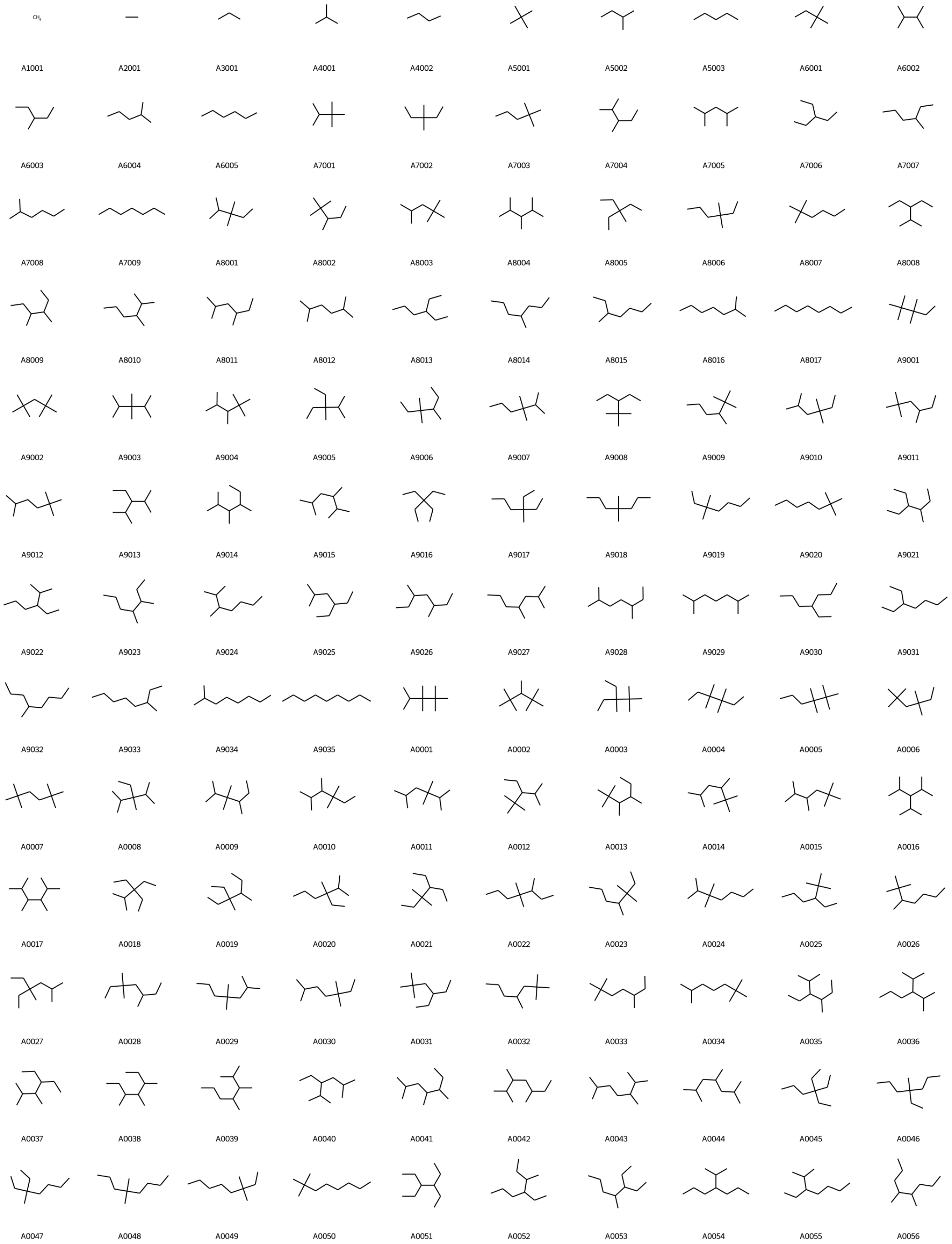

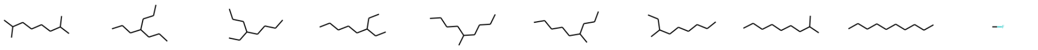

A0067 A0068 A0069 A0070 A0071 A0072 A0073 A0074 A0075 F1101

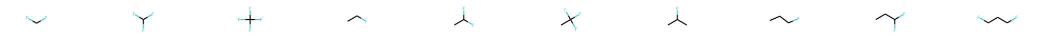

F1201 F1301 F1401 F2101 F2201 F2301 F3101 F3102 F3201 F3202

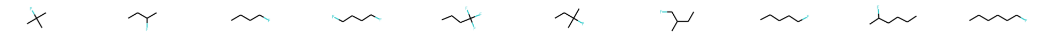

F4101 F4102 F4103 F4201 F4301 F5101 F5102 F5103 F6101 F6102

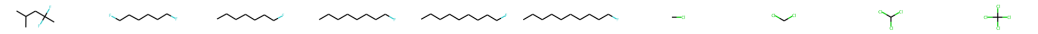

F6201 F6202 F7101 F8101 F9101 F0101 C1101 C1201 C1301 C1401

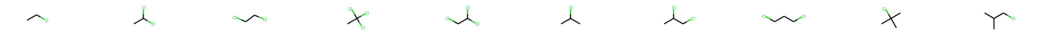

C2101 C2201 C2202 C2301 C2302 C3101 C3201 C3202 C4101 C4102

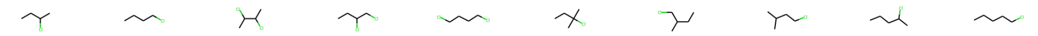

C4103 C4104 C4201 C4202 C4203 C5101 C5102 C5103 C5104 C5105

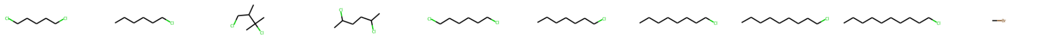

C5201 C6101 C6201 C6202 C6203 C7101 C8101 C9101 C0101 B1101

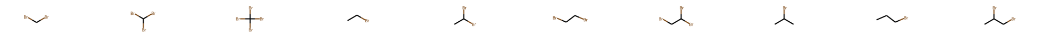

B1201 B1301 B1401 B2101 B2201 B2202 B2301 B3101 B3102 B3201

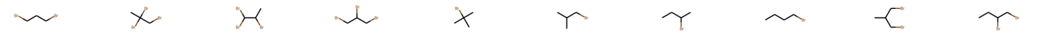

B3202 B3301 B3302 B3303 B4101 B4102 B4103 B4104 B4201 B4202

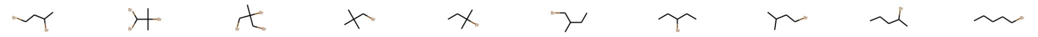

B4203 B4301 B4302 B5101 B5102 B5103 B5104 B5105 B5106 B5107

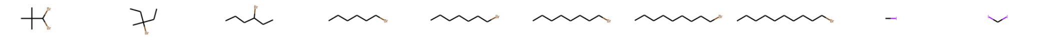

B5201 B6101 B6102 B6103 B7101 B8101 B9101 B0101 I1101 I1201

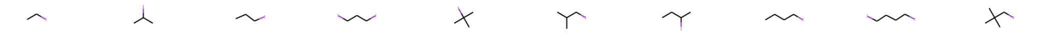

I2101 I3101 I3102 I3201 I4101 I4102 I4103 I4104 I4201 I5101

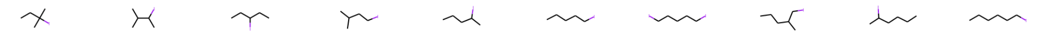

I5102 I5103 I5104 I5105 I5106 I5107 I5201 I6101 I6102 I6103

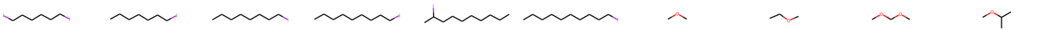

I6201 I7101 I8101 I9101 I0101 I0102 O2101 O3101 O3201 O4101

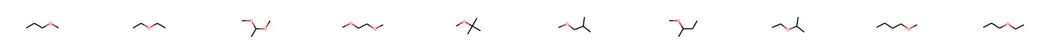

O4102 O4103 O4201 O4202 O5101 O5102 O5103 O5104 O5105 O5106

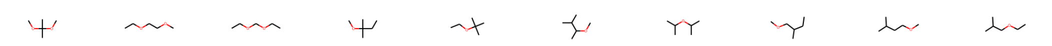

O5201 O5202 O5203 O6101 O6102 O6103 O6104 O6105 O6106 O6107

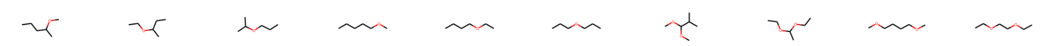

O6108 O6109 O6110 O6111 O6112 O6113 O6201 O6202 O6203 O6204

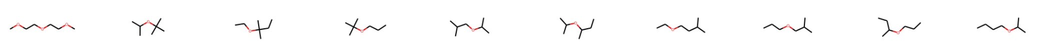

O6301 O7101 O7102 O7103 O7104 O7105 O7106 O7107 O7108 O7109

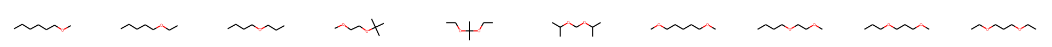

O7110 O7111 O7112 O7201 O7202 O7203 O7204 O7205 O7206 O7207

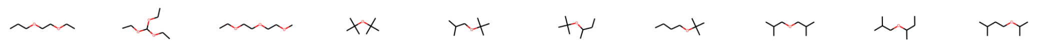

O7208 O7301 O7302 O8101 O8102 O8103 O8104 O8105 O8106 O8107

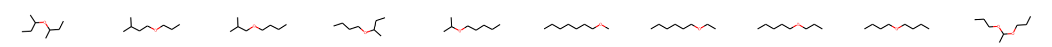

O8108 O8109 O8110 O8111 O8112 O8113 O8114 O8115 O8116 O8201

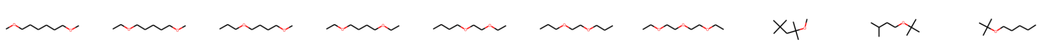

O8202 O8203 O8204 O8205 O8206 O8207 O8301 O9101 O9102 O9103

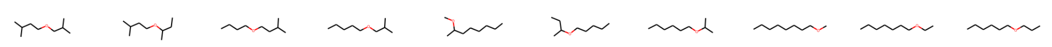

O9104 O9105 O9106 O9107 O9108 O9109 O9110 O9111 O9112 O9113

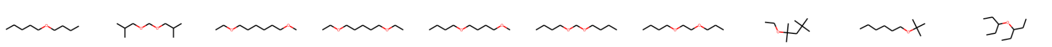

O9114 O9201 O9202 O9203 O9204 O9205 O9206 O0101 O0102 O0103

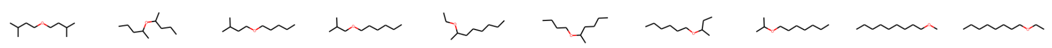

O0104 O0105 O0106 O0107 O0108 O0109 O0110 O0111 O0112 O0113

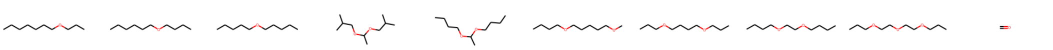

O0114 O0115 O0116 O0201 O0202 O0203 O0204 O0205 O0301 A1101

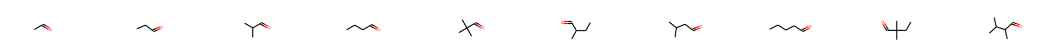

A2101 A3101 A4101 A4102 A5101 A5102 A5103 A5104 A6101 A6102

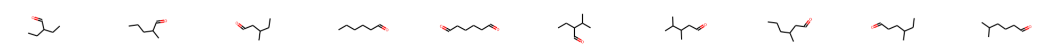

A6103 A6104 A6105 A6106 A6201 A7101 A7102 A7103 A7104 A7105

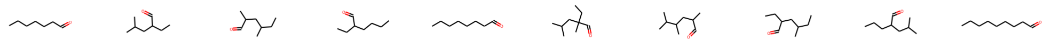
A7106 A8101 A8102 A8103 A8104 A9101 A9102 A9103 A9104 A9105

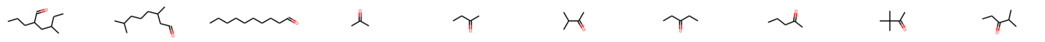
A0101 A0102 A0103 K3101 K4101 K5101 K5102 K5103 K6101 K6102

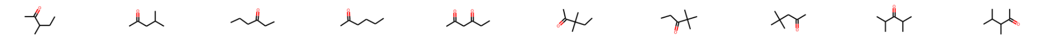
K6103 K6104 K6105 K6106 K6201 K7101 K7102 K7103 K7104 K7105

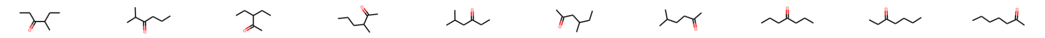
K7106 K7107 K7108 K7109 K7110 K7111 K7112 K7113 K7114 K7115

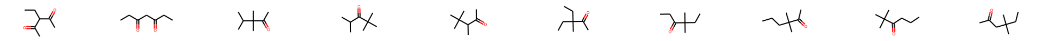
K7201 K7202 K8101 K8102 K8103 K8104 K8105 K8106 K8107 K8108

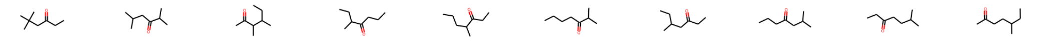
K8109 K8110 K8111 K8112 K8113 K8114 K8115 K8116 K8117 K8118

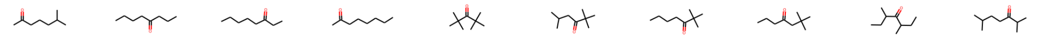
K8119 K8120 K8121 K8122 K9101 K9102 K9103 K9104 K9105 K9106

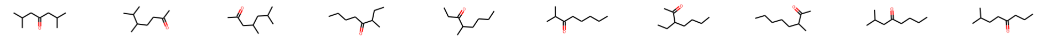
K9107 K9108 K9109 K9110 K9111 K9112 K9113 K9114 K9115 K9116

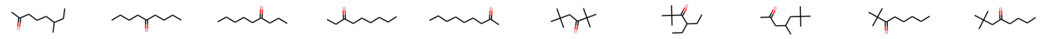
K9117 K9118 K9119 K9120 K9121 K0101 K0102 K0103 K0104 K0105

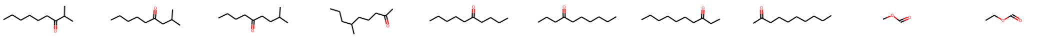
K0106 K0107 K0108 K0109 K0110 K0111 K0112 K0113 E2201 E3201

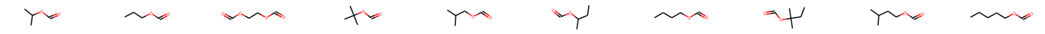
E4201 E4202 E4401 E5201 E5202 E5203 E5204 E6201 E6202 E6203

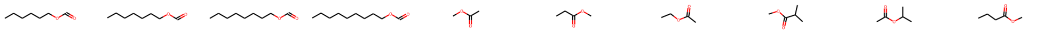
E7201 E8201 E9201 E0201 E3202 E4203 E4204 E5205 E5206 E5207

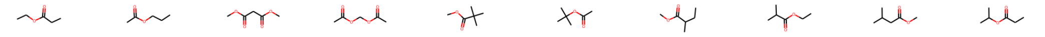
E5208 E5209 E5401 E5402 E6204 E6205 E6206 E6207 E6208 E6209

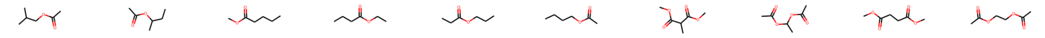
E6210 E6211 E6212 E6213 E6214 E6215 E6401 E6402 E6403 E6404

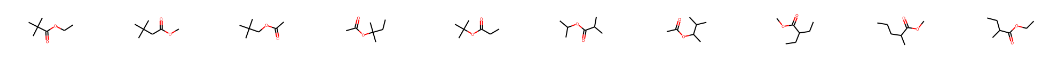

E7202 E7203 E7204 E7205 E7206 E7207 E7208 E7209 E7210 E7211

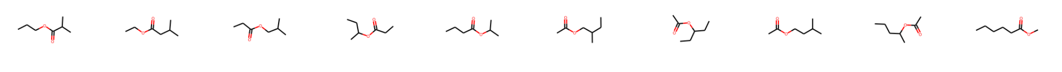

E7212 E7213 E7214 E7215 E7216 E7217 E7218 E7219 E7220 E7221

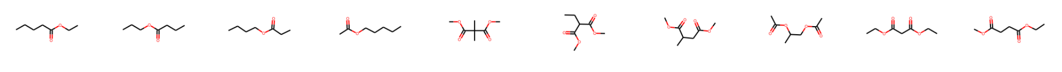

E7222 E7223 E7224 E7225 E7401 E7402 E7403 E7404 E7405 E7406

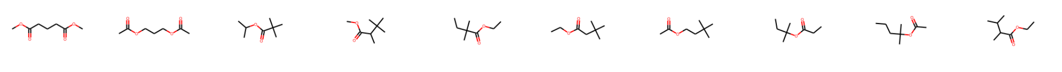

E7407 E7408 E8202 E8203 E8204 E8205 E8206 E8207 E8208 E8209

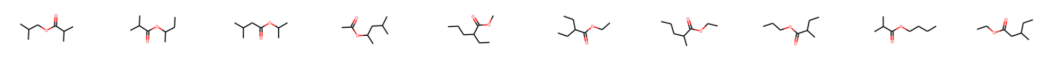

E8210 E8211 E8212 E8213 E8214 E8215 E8216 E8217 E8218 E8219

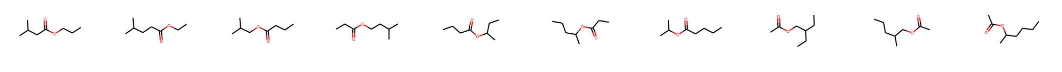

E8220 E8221 E8222 E8223 E8224 E8225 E8226 E8227 E8228 E8229

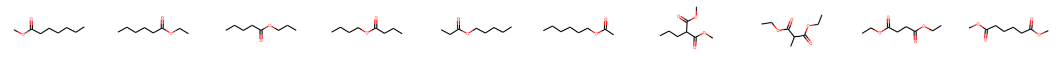

E8230 E8231 E8232 E8233 E8234 E8235 E8401 E8402 E8403 E8404

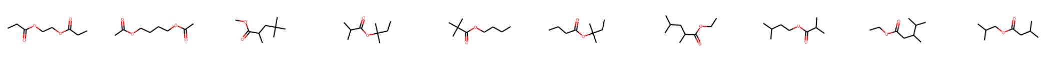

E8405 E8406 E9202 E9203 E9204 E9205 E9206 E9207 E9208 E9209

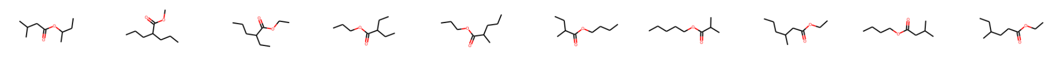

E9210 E9211 E9212 E9213 E9214 E9215 E9216 E9217 E9218 E9219

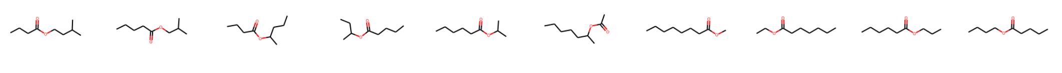

E9220 E9221 E9222 E9223 E9224 E9225 E9226 E9227 E9228 E9229

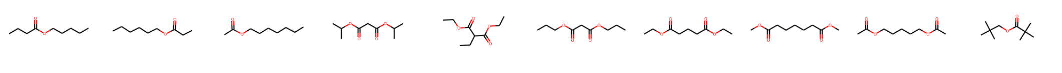

E9230 E9231 E9232 E9401 E9402 E9403 E9404 E9405 E9406 E0202

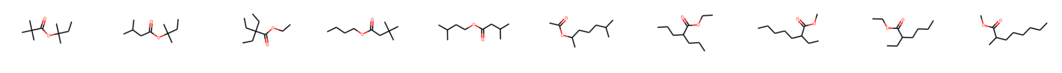

E0203 E0204 E0205 E0206 E0207 E0208 E0209 E0210 E0211 E0212

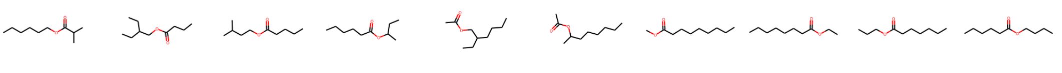

E0213 E0214 E0215 E0216 E0217 E0218 E0219 E0220 E0221 E0222

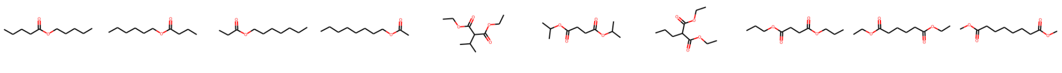

E0223 E0224 E0225 E0226 E0401 E0402 E0403 E0404 E0405 E0406

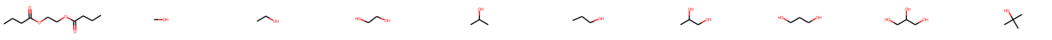

E0407 L1101 L2101 L2201 L3101 L3102 L3201 L3202 L3301 L4101

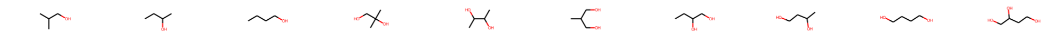

L4102 L4103 L4104 L4201 L4202 L4203 L4204 L4205 L4206 L4301

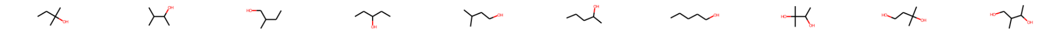

L5101 L5102 L5103 L5104 L5105 L5106 L5107 L5201 L5202 L5203

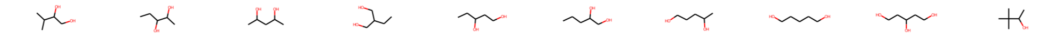

L5204 L5205 L5206 L5207 L5208 L5209 L5210 L5211 L5301 L6101

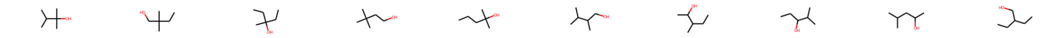

L6102 L6103 L6104 L6105 L6106 L6107 L6108 L6109 L6110 L6111

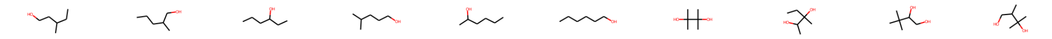

L6112 L6113 L6114 L6115 L6116 L6117 L6201 L6202 L6203 L6204

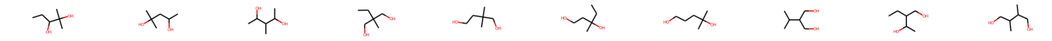

L6205 L6206 L6207 L6208 L6209 L6210 L6211 L6212 L6213 L6214

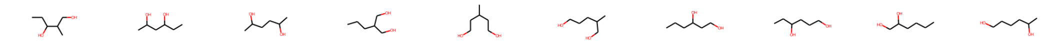

L6215 L6216 L6217 L6218 L6219 L6220 L6221 L6222 L6223 L6224

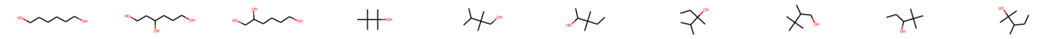

L6225 L6301 L6302 L7101 L7102 L7103 L7104 L7105 L7106 L7107

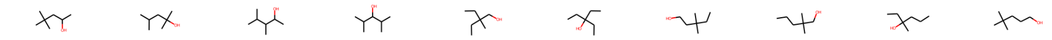

L7108 L7109 L7110 L7111 L7112 L7113 L7114 L7115 L7116 L7117

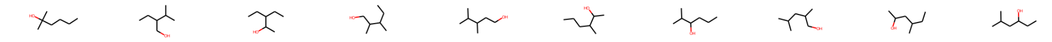

L7118 L7119 L7120 L7121 L7122 L7123 L7124 L7125 L7126 L7127

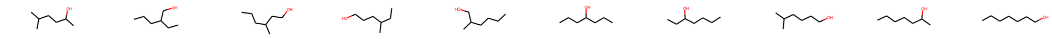

L7128 L7129 L7130 L7131 L7132 L7133 L7134 L7135 L7136 L7137

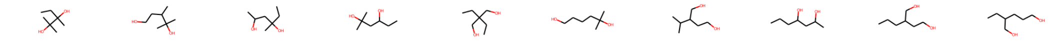

L7201 L7202 L7203 L7204 L7205 L7206 L7207 L7208 L7209 L7210

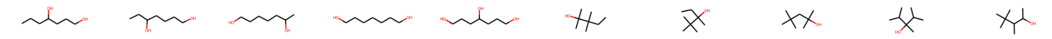

L7211 L7212 L7213 L7214 L7301 L8101 L8102 L8103 L8104 L8105

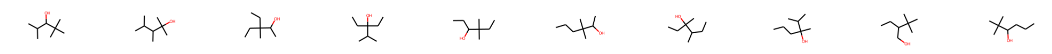

L8106 L8107 L8108 L8109 L8110 L8111 L8112 L8113 L8114 L8115

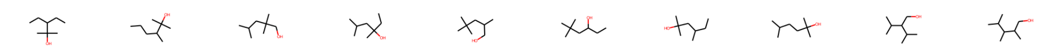

L8116 L8117 L8118 L8119 L8120 L8121 L8122 L8123 L8124 L8125

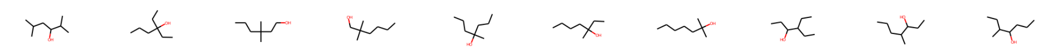

L8126 L8127 L8128 L8129 L8130 L8131 L8132 L8133 L8134 L8135

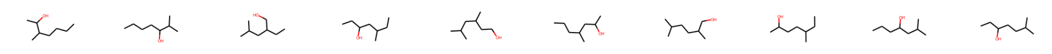

L8136 L8137 L8138 L8139 L8140 L8141 L8142 L8143 L8144 L8145

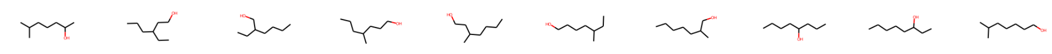

L8146 L8147 L8148 L8149 L8150 L8151 L8152 L8153 L8154 L8155

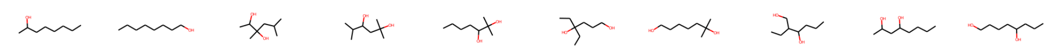

L8156 L8157 L8201 L8202 L8203 L8204 L8205 L8206 L8207 L8208

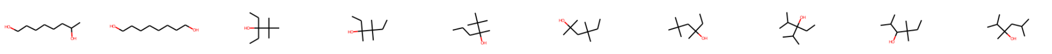

L8209 L8210 L9101 L9102 L9103 L9104 L9105 L9106 L9107 L9108

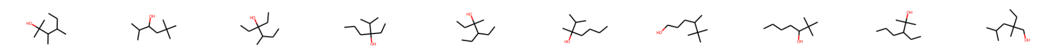

L9109 L9110 L9111 L9112 L9113 L9114 L9115 L9116 L9117 L9118

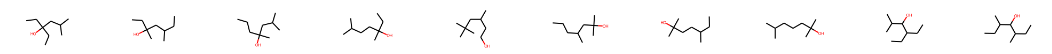

L9119 L9120 L9121 L9122 L9123 L9124 L9125 L9126 L9127 L9128

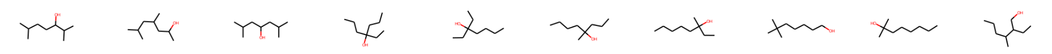

L9129 L9130 L9131 L9132 L9133 L9134 L9135 L9136 L9137 L9138

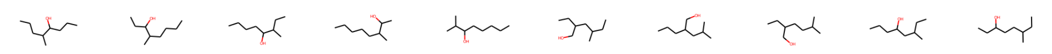

L9139 L9140 L9141 L9142 L9143 L9144 L9145 L9146 L9147 L9148

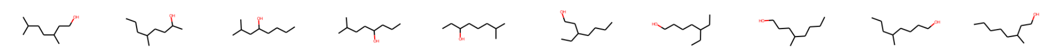

L9149 L9150 L9151 L9152 L9153 L9154 L9155 L9156 L9157 L9158

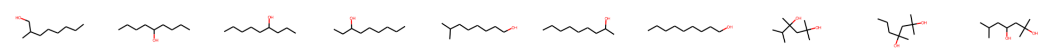

L9159 L9160 L9161 L9162 L9163 L9164 L9165 L9201 L9202 L9203

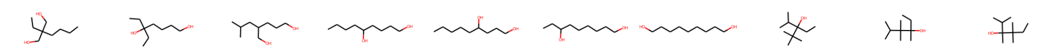

L9204 L9205 L9206 L9207 L9208 L9209 L9210 L0101 L0102 L0103

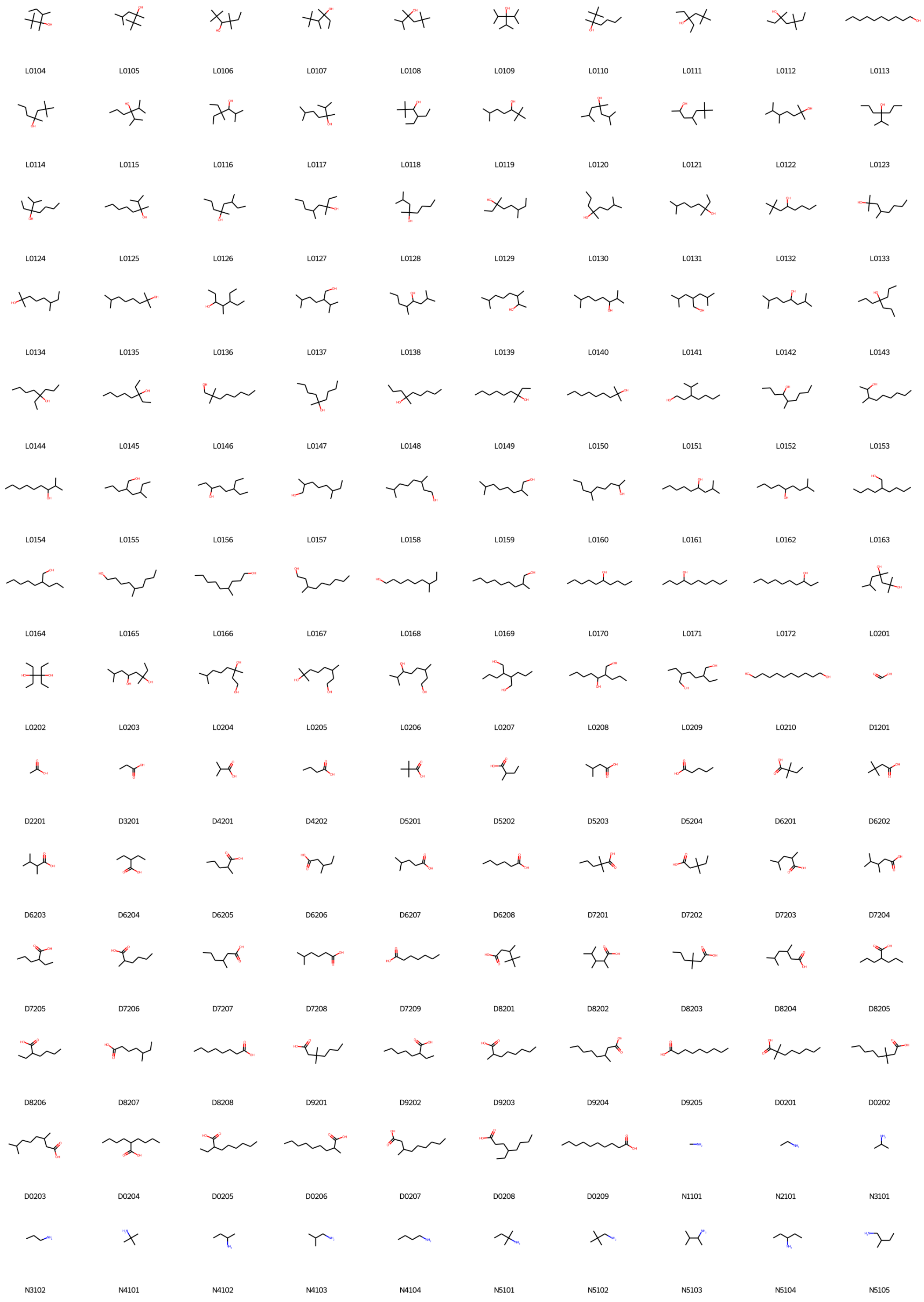
L0104 L0105 L0106 L0107 L0108 L0109 L0110 L0111 L0112 L0113
L0114 L0115 L0116 L0117 L0118 L0119 L0120 L0121 L0122 L0123
L0124 L0125 L0126 L0127 L0128 L0129 L0130 L0131 L0132 L0133
L0134 L0135 L0136 L0137 L0138 L0139 L0140 L0141 L0142 L0143
L0144 L0145 L0146 L0147 L0148 L0149 L0150 L0151 L0152 L0153
L0154 L0155 L0156 L0157 L0158 L0159 L0160 L0161 L0162 L0163
L0164 L0165 L0166 L0167 L0168 L0169 L0170 L0171 L0172 L0201
L0202 L0203 L0204 L0205 L0206 L0207 L0208 L0209 L0210 D1201
D2201 D3201 D4201 D4202 D5201 D5202 D5203 D5204 D6201 D6202
D6203 D6204 D6205 D6206 D6207 D6208 D7201 D7202 D7203 D7204
D7205 D7206 D7207 D7208 D7209 D8201 D8202 D8203 D8204 D8205
D8206 D8207 D8208 D9201 D9202 D9203 D9204 D9205 D0201 D0202
D0203 D0204 D0205 D0206 D0207 D0208 D0209 N1101 N2101 N3101
N3102 N4101 N4102 N4103 N4104 N5101 N5102 N5103 N5104 N5105

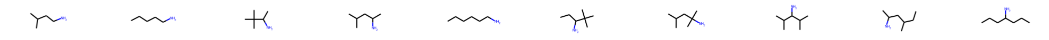

N5106 N5107 N6101 N6102 N6103 N7101 N7102 N7103 N7104 N7105

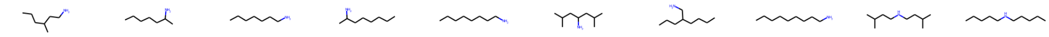

N7106 N7107 N7108 N8101 N8102 N9101 N9102 N9103 N0102 N0103

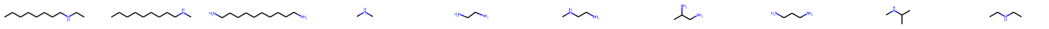

N0104 N0105 N0201 N2102 N2201 N3201 N3202 N3203 N4105 N4106

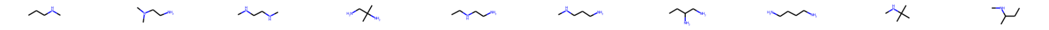

N4107 N4201 N4202 N4203 N4204 N4205 N4206 N4207 N5108 N5109

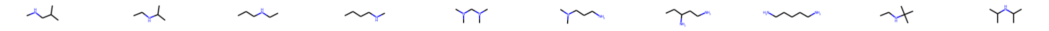

N5110 N5111 N5112 N5113 N5201 N5202 N5203 N5204 N6104 N6105

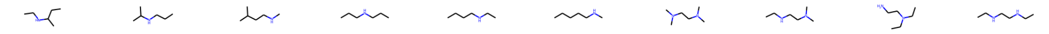

N6106 N6107 N6108 N6109 N6110 N6111 N6201 N6202 N6203 N6204

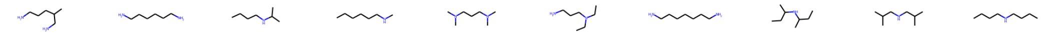

N6205 N6206 N7109 N7110 N7201 N7202 N7203 N8103 N8104 N8105

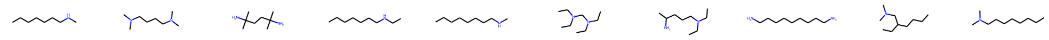

N8106 N8201 N8202 N9104 N9105 N9201 N9202 N9203 N0106 N0107

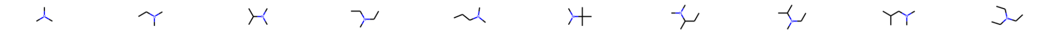

N3103 N4108 N5114 N5115 N5116 N6112 N6113 N6114 N6115 N6116

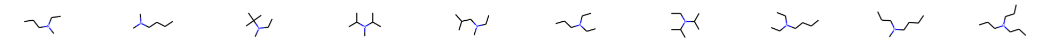

N6117 N6118 N7111 N7112 N7113 N7114 N8107 N8108 N8109 N9106

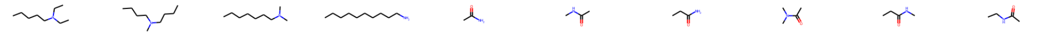

N9107 N9108 N9109 N0101 M2201 M3201 M3202 M4201 M4202 M4203

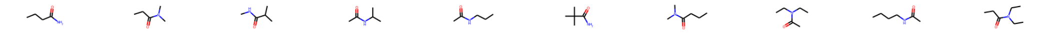

M4204 M5201 M5202 M5203 M5204 M5205 M6201 M6202 M6203 M7201

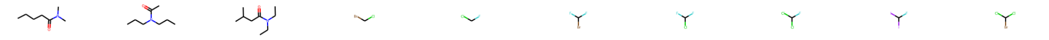

M7202 M8201 M9201 S1201 S1202 S1301 S1302 S1303 S1304 S1305

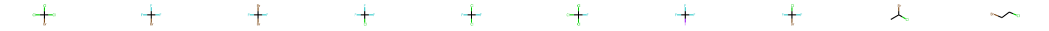

S1401 S1402 S1403 S1404 S1405 S1406 S1407 S1408 S2201 S2202

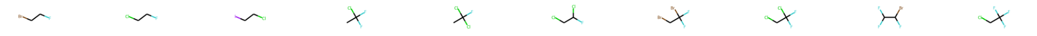

S2203 S2204 S2205 S2301 S2302 S2303 S2401 S2402 S2403 S2404

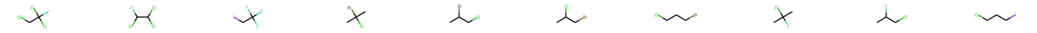

S2405 S2406 S2407 S3201 S3202 S3203 S3204 S3205 S3206 S3207

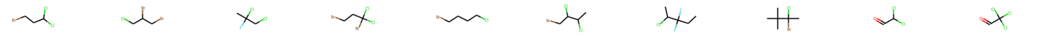

S3301 S3302 S3303 S3401 S4201 S4301 S5301 S6201 S2304 S2408

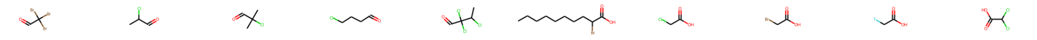

S2409 S3208 S4202 S4203 S4401 S0301 S2305 S2306 S2307 S2410

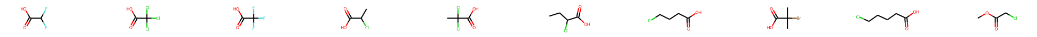

S2411 S2501 S2502 S3304 S3402 S4302 S4303 S4304 S5302 S3305

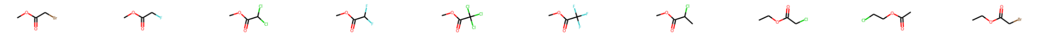

S3306 S3307 S3403 S3404 S3501 S3502 S4305 S4306 S4307 S4308

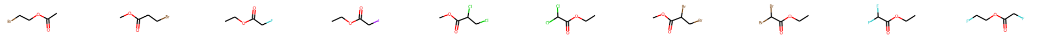

S4309 S4310 S4311 S4312 S4402 S4403 S4404 S4405 S4406 S4407

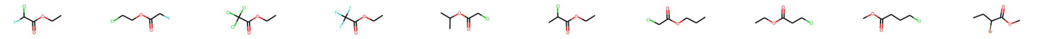

S4408 S4409 S4501 S4502 S5303 S5304 S5305 S5306 S5307 S5308

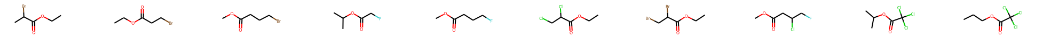

S5309 S5310 S5311 S5312 S5313 S5401 S5402 S5403 S5501 S5502

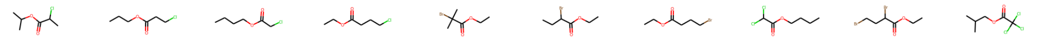

S6301 S6302 S6303 S6304 S6305 S6306 S6307 S6401 S6402 S6501

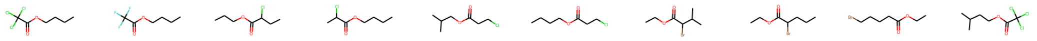

S6502 S6503 S7301 S7302 S7303 S7304 S7305 S7306 S7307 S7501

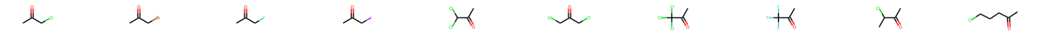

S3209 S3210 S3211 S3212 S3308 S3309 S3405 S3406 S4204 S5201

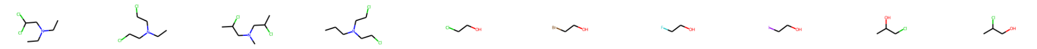

S6308 S6309 S7308 S7309 S2206 S2207 S2208 S2209 S3213 S3214

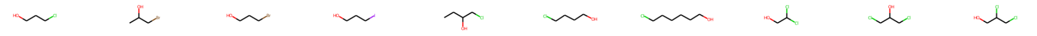

S3215 S3216 S3217 S3218 S4205 S4206 S6202 S2308 S3310 S3311

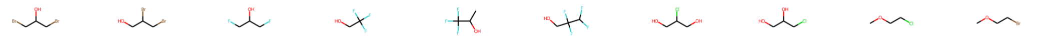

S3312 S3313 S3314 S2412 S3407 S3503 S3315 S3316 S3219 S3220

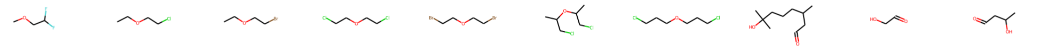

S3317 S4207 S4208 S4313 S4314 S6310 S6311 S0201 S2210 S4209

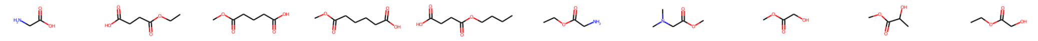

S2309 S6403 S6404 S7401 S8401 S4315 S5314 S3318 S4316 S4317

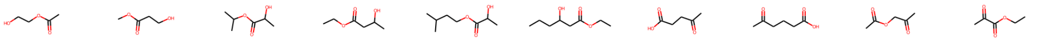

S4318 S4319 S6312 S6313 S8301 S8302 S5315 S6314 S5316 S5317

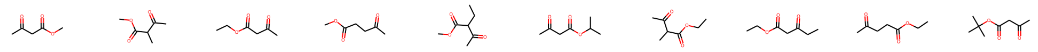

S5318 S6315 S6316 S6317 S7310 S7311 S7312 S7313 S7314 S8303

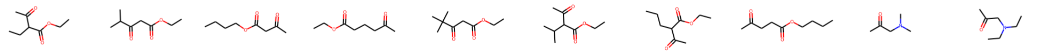

S8304 S8305 S8306 S8307 S9301 S9302 S9303 S9304 S5202 S7201

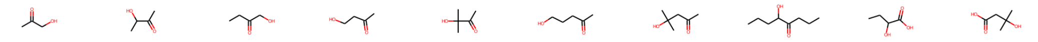

S3221 S4210 S4211 S4212 S5203 S5204 S6203 S8201 S4320 S5319

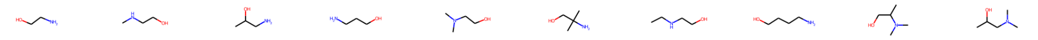

S2211 S3222 S3223 S3224 S4213 S4214 S4215 S4216 S5205 S5206

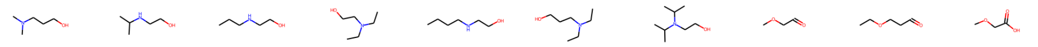

S5207 S5208 S5209 S6204 S6205 S7202 S8202 S3225 S5210 S3319

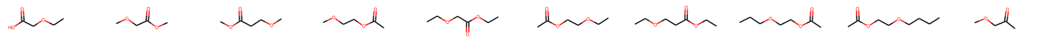

S4321 S4322 S5320 S5321 S6318 S6319 S7315 S7316 S8308 S4217

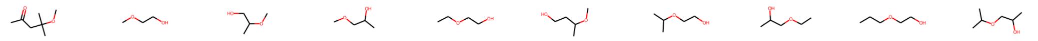

S7203 S3226 S4218 S4219 S4220 S5211 S5212 S5213 S5214 S6206

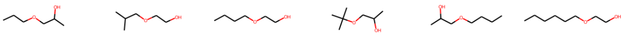

S6207 S6208 S6209 S7204 S7205 S8203

S.3 Reference Experimental Data

The reference experimental data used in this work is reported in Tabs. S.2 and S.3. The $N_{\mathrm{iso}}^{\mathrm{cal}} = 1516$ molecules considered are referred to by their code and SMILES string. The structures of these compounds are displayed in Fig. S.2. For these molecules, simulations are performed at $N_{\mathrm{sim}}^{\mathrm{cal}} = 1607$ thermodynamic state points, with indicated pressure P and temperature T (Tab. S.2). Simulations of the same compound performed at different P, T-points are distinguished by an extra letter (a, b or c) appended to the molecule code. The quantities reported along with a literature source (Src) are the pure-liquid density ρ_{liq}, the vaporization enthalpy ΔH_{vap}, the melting point T_m and the boiling point T_b at $P^{\circ} = 1$ bar, the critical point T_c (pressure P_c unspecified), and the static relative dielectric permittivity ϵ. For ϵ, the column with the source contains either the experimental source or a dash to indicate an educated guess. In the latter case, the ϵ value is reported between parentheses. The experimental values for the surface-tension coefficient γ, the static relative dielectric permittivity ϵ, and the self-diffusion coefficient D of the 66 compounds used in the validation step are reported in Tab. S.3.

Table S.2: Reference experimental data pertaining to the $N_{\text{iso}}^{\text{cal}} = 1516$ molecules (running index n_{iso}) and $N_{\text{sim}}^{\text{cal}} = 1607$ P, T-points (running index n_{sim}) considered in the simulations.

n_{sim}	n_{iso}	Code	Smiles	P [bar]	T [K]	ρ_{liq} [kg·m^{-3}]	Src	ΔH_{vap} [kJ·mol^{-1}]	Src	T_m [K]	Src	T_b [K]	Src	T$_c$ [K]	Src	ϵ	Src
1	1	A1001a	C	0.12	90.69	451.02	81	9.22	81	90.7	87	111.7	81	190.6	81	1.3	86
2	1	A1001b	C	0.41	101.78	436.07	81	8.84	81	90.7	87	111.7	81	190.6	81	1.3	86
3	1	A1001c	C	1.01	111.67	422.0	81	-	-	90.7	87	111.7	81	190.6	81	1.3	86
4	1	A1001d	C	1.01	111.67	-	-	8.46	81	90.7	87	111.7	81	190.6	81	1.3	86
5	2	A2001a	CC	1.26	188.67	526.59	81	-	-	90.4	87	184.6	81	305.3	81	1.4	86
6	2	A2001b	CC	20.88	266.4	409.39	81	9.74	81	90.4	87	184.6	81	305.3	81	1.4	86
7	2	A2001c	CC	1.01	184.57	-	-	14.76	81	90.4	87	184.6	81	305.3	81	1.4	86
8	3	A3001a	CCC	1.0	230.0	582.13	78	18.8	84	85.5	87	231.1	81	369.8	81	1.7	86
9	3	A3001b	CCC	9.78	298.15	493.05	81	-	-	85.5	87	231.1	81	369.8	81	1.7	86
10	4	A4001a	CC(C)C	19.85	260.0	597.94	78	-	-	113.8	87	261.4	81	407.8	81	1.7	86
11	4	A4001b	CC(C)C	25.33	300.0	552.99	78	-	-	113.8	87	261.4	81	407.8	81	1.7	86
12	4	A4001c	CC(C)C	1.0	265.0	-	-	22.4	84	113.8	87	261.4	81	407.8	81	1.7	86
13	5	A4002a	CCCC	1.0	273.15	601.0	78	-	-	134.9	87	272.6	81	425.1	81	1.8	87
14	5	A4002b	CCCC	9.93	300.0	572.45	78	-	-	134.9	87	272.6	81	425.1	81	1.8	87
15	5	A4002c	CCCC	1.0	264.0	-	-	23.1	84	134.9	87	272.6	81	425.1	81	1.8	87
16	6	A5001a	CC(C)(C)C	0.99	282.01	603.71	81	23.65	81	256.8	87	282.6	81	433.8	81	1.8	87
17	6	A5001b	CC(C)(C)C	1.76	298.15	585.96	81	-	-	256.8	87	282.6	81	433.8	81	1.8	87
18	7	A5002a	CCC(C)C	0.92	298.15	616.05	81	25.46	81	113.3	87	301.0	81	460.4	81	1.8	86
19	8	A5003a	CCCCC	1.01	298.15	621.14	78	26.61	86	143.5	87	309.2	81	469.7	81	1.8	86
20	9	A6001a	CCC(C)(C)C	1.01	298.15	644.43	78	28.78	81	174.2	87	322.9	81	489.0	81	1.9	86
21	10	A6002a	CC(C)C(C)C	1.01	298.15	658.56	78	30.03	81	145.1	87	331.1	81	500.0	81	1.9	86
22	11	A6003a	CCC(C)CC	1.01	298.15	659.94	78	30.87	81	110.3	87	336.4	81	504.4	81	1.9	86
23	12	A6004a	CCCC(C)C	1.01	298.15	648.45	78	30.43	81	119.5	87	333.4	81	497.7	81	1.9	86
24	13	A6005a	CCCCCC	1.0	298.15	656.1	78	31.76	81	177.9	87	341.9	81	507.6	81	1.9	86
25	14	A7001a	CC(C)C(C)(C)C	0.13	298.15	687.1	81	32.4	84	248.6	87	354.0	81	531.2	81	1.9	86
26	15	A7002a	CCC(C)(C)CC	0.11	298.15	686.88	81	34.33	81	138.8	87	359.2	81	536.4	81	1.9	86
27	16	A7003a	CCCC(C)(C)C	0.14	298.15	673.08	81	33.39	81	149.4	87	352.3	81	520.5	81	1.9	86
28	17	A7004a	CCC(C)C(C)C	0.09	298.15	691.09	81	34.98	81	-	-	362.9	81	537.3	81	6.7	86
29	18	A7005a	CC(C)CC(C)C	0.13	298.15	667.93	81	33.64	81	154.0	87	353.6	81	519.8	81	1.9	86
30	19	A7006a	CCC(CC)CC	0.08	298.15	695.11	81	35.2	84	154.6	87	366.6	81	527.0	81	1.9	87
31	20	A7007a	CCCC(C)CC	0.08	298.15	683.93	81	35.4	81	153.8	87	365.0	81	535.2	81	1.9	86
32	21	A7008a	CCCCC(C)C	0.09	298.15	673.96	81	35.17	81	154.9	87	363.2	81	530.4	81	1.9	86
33	22	A7009a	CCCCCCC	0.06	298.15	681.95	81	36.5	81	182.6	87	371.6	81	540.2	81	1.9	78
34	23	A8001a	CCC(C)(C)C(C)C	0.04	298.15	722.13	81	37.6	84	171.9	87	387.9	81	573.5	81	2.0	87
35	24	A8002a	CCC(C)C(C)(C)C	0.04	298.15	712.02	81	-	-	160.8	87	383.0	81	563.5	81	2.0	87
36	25	A8003a	CC(C)CC(C)(C)C	1.01	298.15	688.03	78	35.2	84	165.8	87	372.4	81	543.8	81	1.9	86
37	26	A8004a	CC(C)C(C)C(C)C	0.04	298.15	715.92	81	37.7	84	163.8	87	386.6	81	566.4	81	2.0	86
38	27	A8005a	CCC(C)(CC)CC	0.03	298.15	724.0	81	38.0	84	182.3	87	391.4	81	576.5	81	2.0	87
39	28	A8006a	CCCC(C)(C)CC	0.04	298.15	707.11	81	37.5	84	146.9	87	385.1	81	562.0	81	2.0	87
40	29	A8007a	CCCCC(C)(C)C	0.05	298.15	692.08	81	37.3	84	152.0	87	380.0	81	549.8	81	1.9	87
41	30	A8008a	CCC(CC)C(C)C	0.03	298.15	711.08	81	38.5	84	158.2	87	388.8	81	567.0	81	(2.0)	-
42	31	A8009a	CCC(C)C(C)CC	0.03	298.15	715.91	81	39.0	84	-	-	390.9	81	568.8	81	2.0	86
43	32	A8010a	CCCC(C)C(C)C	0.03	298.15	708.06	81	38.8	84	-	-	388.8	81	563.5	81	(2.0)	-
44	33	A8011a	CCC(C)CC(C)C	1.0	298.15	696.43	78	37.8	84	-	-	382.6	81	553.5	81	2.0	78
45	34	A8012a	CC(C)CCC(C)C	0.04	298.15	690.03	81	37.9	84	182.0	87	382.3	81	550.0	81	2.0	87

Table S.2 – Reference experimental data (continued)

n_{sim}	n_{iso}	Code	Smiles	P [bar]	T [K]	ρ_{liq} [kg·m^{-3}]	Src	ΔH_{vap} [kJ·mol^{-1}]	Src	T_m [K]	Src	T_b [K]	Src	T$_c$ [K]	Src	ϵ	Src
46	35	A8013a	CCCC(CC)CC	0.03	298.15	710.13	81	39.71	81	-	-	391.7	81	565.5	81	2.0	87
47	36	A8014a	CCCC(C)CCC	0.03	298.15	713.05	81	39.66	81	152.2	87	390.9	81	561.7	81	(2.0)	-
48	37	A8015a	CCCCC(C)CC	0.03	298.15	702.03	81	39.85	81	152.7	87	392.1	81	563.7	81	1.9	78
49	38	A8016a	CCCCCC(C)C	0.03	298.15	696.05	81	39.72	81	164.2	87	390.8	81	559.6	81	2.0	86
50	39	A8017a	CCCCCCCC	1.01	298.15	698.86	78	41.03	81	216.4	87	398.8	81	568.7	81	1.9	86
51	40	A9001a	CCC(C)(C)C(C)(C)C	1.0	298.15	753.0	87	41.2	84	263.4	87	413.4	81	607.5	81	(1.9)	-
52	41	A9002a	CC(C)(C)CC(C)(C)C	0.03	298.15	716.06	81	38.5	84	206.6	87	395.4	81	574.6	81	(1.9)	-
53	42	A9003a	CC(C)C(C)(C)C(C)C	0.01	301.22	732.95	81	-	-	171.1	87	414.7	81	607.5	81	(1.9)	-
54	42	A9003b	CC(C)C(C)(C)C(C)C	1.0	298.0	-	-	41.8	84	171.1	87	414.7	81	607.5	81	(1.9)	-
55	43	A9004a	CC(C)C(C)C(C)(C)C	0.02	298.15	735.14	81	40.8	84	151.8	87	406.2	81	592.6	81	(1.9)	-
56	44	A9005a	CCC(C)(CC)C(C)C	1.0	298.15	750.8	87	42.7	84	-	-	417.9	81	582.1	81	(1.9)	-
57	45	A9006a	CCC(C)C(C)(C)CC	1.0	298.15	741.4	87	42.2	84	171.9	87	413.6	81	582.1	81	(1.9)	-
58	46	A9007a	CCCC(C)(C)C(C)C	1.0	298.15	734.5	87	42.1	84	156.3	87	410.8	81	582.1	81	(1.9)	-
59	47	A9008a	CCC(CC)C(C)(C)C	0.02	298.15	731.1	81	41.7	84	173.8	87	407.0	81	582.1	81	(1.9)	-
60	48	A9009a	CCCC(C)C(C)(C)C	0.02	298.15	725.06	81	41.7	84	-	-	406.8	81	582.1	81	(1.9)	-
61	49	A9010a	CCC(C)(C)CC(C)C	0.02	298.15	720.06	81	41.1	84	159.8	87	403.8	81	582.1	81	(1.9)	-
62	50	A9011a	CCC(C)CC(C)(C)C	0.02	298.15	713.07	81	40.7	84	151.2	87	399.7	81	582.1	81	(1.9)	-
63	51	A9012a	CC(C)CCC(C)(C)C	0.02	298.15	707.13	81	40.2	84	167.2	87	397.2	81	569.8	81	(1.9)	-
64	52	A9013a	CCC(C(C)C)C(C)C	0.01	298.15	733.86	81	42.3	84	150.8	87	409.9	81	582.1	81	(1.9)	-
65	53	A9014a	CCC(C)C(C)C(C)C	1.0	298.15	735.4	87	42.7	84	-	-	412.2	81	582.1	81	(1.9)	-
66	54	A9015a	CC(C)CC(C)C(C)C	0.02	298.15	718.06	81	41.4	84	145.2	87	404.5	81	582.1	81	(1.9)	-
67	55	A9016a	CCC(CC)(CC)CC	0.03	308.36	727.87	81	-	-	240.1	87	419.3	81	610.0	81	(1.9)	-
68	55	A9016b	CCC(CC)(CC)CC	1.0	298.0	-	-	42.6	84	240.1	87	419.3	81	610.0	81	(1.9)	-
69	56	A9017a	CCCC(C)(CC)CC	1.0	298.15	737.1	87	42.9	84	-	-	413.8	81	582.1	81	(1.9)	-
70	57	A9018a	CCCC(C)(C)CCC	0.01	298.15	721.0	81	42.2	84	-	-	408.4	81	582.1	81	(1.9)	-
71	58	A9019a	CCCCC(C)(C)CC	0.01	305.91	715.91	81	-	-	-	-	410.4	81	582.1	81	(1.9)	-
72	58	A9019b	CCCCC(C)(C)CC	1.0	298.0	-	-	42.6	84	-	-	410.4	81	582.1	81	(1.9)	-
73	59	A9020a	CCCCCC(C)(C)C	0.02	298.15	707.14	81	42.3	84	160.1	87	405.8	81	576.7	81	(1.9)	-
74	60	A9021a	CCC(C)C(CC)CC	0.01	302.75	732.6	81	-	-	-	-	413.6	81	582.1	81	(1.9)	-
75	60	A9021b	CCC(C)C(CC)CC	1.0	298.0	-	-	43.6	84	-	-	413.6	81	582.1	81	(1.9)	-
76	61	A9022a	CCCC(CC)C(C)C	0.01	300.36	727.41	81	-	-	-	-	411.2	81	582.1	81	(1.9)	-
77	61	A9022b	CCCC(CC)C(C)C	1.0	298.0	-	-	43.2	84	-	-	411.2	81	582.1	81	(1.9)	-
78	62	A9023a	CCCC(C)C(C)CC	0.01	302.23	723.9	81	-	-	-	-	413.8	81	582.1	81	(1.9)	-
79	62	A9023b	CCCC(C)C(C)CC	1.0	298.0	-	-	43.6	84	-	-	413.8	81	582.1	81	(1.9)	-
80	63	A9024a	CCCCC(C)C(C)C	0.01	303.24	717.92	81	-	-	157.2	87	413.7	81	582.1	81	(1.9)	-
81	63	A9024b	CCCCC(C)C(C)C	1.0	298.0	-	-	43.6	84	157.2	87	413.7	81	582.1	81	(1.9)	-
82	64	A9025a	CCC(CC)CC(C)C	0.01	298.15	719.05	81	42.9	84	-	-	407.0	81	582.1	81	(1.9)	-
83	65	A9026a	CCC(C)CC(C)CC	0.01	299.6	717.93	81	43.3	84	-	-	409.2	81	582.1	81	(1.9)	-
84	66	A9027a	CCCC(C)CC(C)C	0.01	298.15	711.06	81	42.9	84	-	-	406.1	81	582.1	81	1.9	87
85	67	A9028a	CCC(C)CCC(C)C	0.01	299.78	711.75	81	43.3	84	-	-	409.2	81	582.1	81	1.9	87
86	68	A9029a	CC(C)CCCC(C)C	0.01	298.15	705.92	81	43.3	84	170.1	87	408.4	81	582.1	81	2.0	87
87	69	A9030a	CCCC(CC)CCC	1.0	298.15	724.1	87	44.1	84	-	-	414.4	81	582.1	81	(1.9)	-
88	70	A9031a	CCCCC(CC)CC	1.0	298.15	722.5	87	44.5	84	158.2	87	416.4	81	582.1	81	(1.9)	-
89	71	A9032a	CCCCC(C)CCC	1.0	298.15	716.0	87	44.5	84	157.2	87	415.6	81	582.1	81	2.0	87
90	72	A9033a	CCCCCC(C)CC	1.0	298.15	717.0	87	44.9	84	165.2	87	417.4	81	582.1	81	(1.9)	-
91	73	A9034a	CCCCCCC(C)C	1.0	298.15	709.5	87	-	-	192.8	87	416.4	81	582.8	81	2.0	87
92	73	A9034b	CCCCCCC(C)C	0.01	304.61	-	-	43.84	81	192.8	87	416.4	81	582.8	81	2.0	87
93	74	A9035a	CCCCCCCCC	1.01	298.15	713.71	78	46.4	84	219.7	87	424.0	81	594.6	81	2.0	78

Table S.2 – Reference experimental data (continued)

n_{sim}	n_{iso}	Code	Smiles	P [bar]	T [K]	ρ_{liq} [kg·m^{-3}]	Src	ΔH_{vap} [kJ·mol^{-1}]	Src	T_m [K]	Src	T_b [K]	Src	T$_c$ [K]	Src	ϵ	Src
94	75	A0001a	CC(C)C(C)(C)C(C)(C)C	1.0	298.15	776.7	87	45.2	84	236.7	87	439.2	81	606.6	81	(2.0)	-
95	76	A0002a	CC(C(C)(C)C)C(C)(C)C	1.0	298.15	763.6	87	43.5	84	234.3	87	432.4	81	606.6	81	(2.0)	-
96	77	A0003a	CCC(C)(CC)C(C)(C)C	0.01	319.8	761.5	81	-	-	-	-	442.7	81	606.6	81	(2.0)	-
97	77	A0003b	CCC(C)(CC)C(C)(C)C	1.0	298.0	-	-	46.0	84	-	-	442.7	81	606.6	81	(2.0)	-
98	78	A0004a	CCC(C)(C)C(C)(C)CC	1.0	298.15	778.9	87	42.3	84	-	-	443.2	81	606.6	81	(2.0)	-
99	79	A0005a	CCCC(C)(C)C(C)(C)C	1.0	298.15	760.9	87	45.2	84	219.2	87	433.5	81	623.0	81	(2.0)	-
100	80	A0006a	CCC(C)(C)CC(C)(C)C	1.0	298.0	-	-	43.5	84	-	-	427.0	81	606.6	81	(2.0)	-
101	81	A0007a	CC(C)(C)CCC(C)(C)C	1.0	298.15	714.8	87	43.5	84	260.6	87	410.6	81	581.4	81	(2.0)	-
102	82	A0008a	CCC(C)(C(C)C)C(C)C	1.0	298.0	-	-	46.4	84	-	-	442.6	81	606.6	81	(2.0)	-
103	83	A0009a	CCC(C)C(C)(C)C(C)C	1.0	298.0	-	-	46.4	84	-	-	437.8	81	606.6	81	(2.0)	-
104	84	A0010a	CCC(C)(C)C(C)C(C)C	1.0	298.0	-	-	46.0	84	-	-	434.8	81	606.6	81	(2.0)	-
105	85	A0011a	CC(C)CC(C)(C)C(C)C	1.0	298.0	-	-	45.2	84	-	-	426.3	81	606.6	81	(2.0)	-
106	86	A0012a	CCC(C(C)C)C(C)(C)C	1.0	298.0	-	-	44.8	84	-	-	428.5	81	606.6	81	(2.0)	-
107	87	A0013a	CCC(C)C(C)C(C)(C)C	1.0	298.0	-	-	45.6	84	-	-	432.0	81	606.6	81	(2.0)	-
108	88	A0014a	CC(C)CC(C)C(C)(C)C	1.0	298.0	-	-	45.2	84	-	-	421.6	81	606.6	81	(2.0)	-
109	89	A0015a	CC(C)C(C)CC(C)(C)C	1.0	298.0	-	-	44.4	84	-	-	421.0	81	606.6	81	(2.0)	-
110	90	A0016a	CC(C)C(C(C)C)C(C)C	1.0	298.15	754.5	87	45.6	84	191.4	87	430.2	81	606.6	81	(2.0)	-
111	91	A0017a	CC(C)C(C)C(C)C(C)C	1.0	298.0	-	-	46.0	84	-	-	429.4	81	606.6	81	(2.0)	-
112	92	A0018a	CCC(CC)(CC)C(C)C	1.0	298.0	-	-	47.3	84	-	-	442.9	81	606.6	81	(2.0)	-
113	93	A0019a	CCC(C)C(C)(CC)CC	1.0	298.0	-	-	46.4	84	-	-	435.3	81	606.6	81	(2.0)	-
114	94	A0020a	CCCC(C)(CC)C(C)C	1.0	298.0	-	-	46.9	84	-	-	436.9	81	606.6	81	(2.0)	-
115	95	A0021a	CCC(CC)C(C)(C)CC	1.0	298.0	-	-	46.4	84	-	-	436.1	81	606.6	81	(2.0)	-
116	96	A0022a	CCCC(C)(C)C(C)CC	1.0	298.0	-	-	46.4	84	-	-	434.3	81	606.6	81	(2.0)	-
117	97	A0023a	CCCC(C)C(C)(C)CC	1.0	298.0	-	-	46.9	84	-	-	435.1	81	606.6	81	(2.0)	-
118	98	A0024a	CCCCC(C)(C)C(C)C	1.0	298.0	-	-	46.9	84	-	-	433.4	81	606.6	81	(2.0)	-
119	99	A0025a	CCCC(CC)C(C)(C)C	1.0	298.0	-	-	46.0	84	-	-	429.3	81	606.6	81	(2.0)	-
120	100	A0026a	CCCCC(C)C(C)(C)C	1.0	298.0	-	-	46.9	84	-	-	430.8	81	606.6	81	(2.0)	-
121	101	A0027a	CCC(C)(CC)CC(C)C	1.0	298.0	-	-	46.4	84	-	-	434.3	81	606.6	81	(2.0)	-
122	102	A0028a	CCC(C)CC(C)(C)CC	0.01	313.38	728.1	81	-	-	-	-	428.9	81	609.5	81	(2.0)	-
123	102	A0028b	CCC(C)CC(C)(C)CC	1.0	298.0	-	-	46.0	84	-	-	428.9	81	609.5	81	(2.0)	-
124	103	A0029a	CCCC(C)(C)CC(C)C	1.0	298.0	-	-	45.2	84	-	-	424.2	81	606.6	81	(2.0)	-
125	104	A0030a	CCC(C)(C)CCC(C)C	1.0	298.15	736.2	87	46.0	84	-	-	426.0	81	606.6	81	(2.0)	-
126	105	A0031a	CCC(CC)CC(C)(C)C	1.0	298.0	-	-	45.2	84	-	-	420.2	81	606.6	81	(2.0)	-
127	106	A0032a	CCCC(C)CC(C)(C)C	1.0	298.0	-	-	45.6	84	-	-	421.5	81	606.6	81	(2.0)	-
128	107	A0033a	CCC(C)CCC(C)(C)C	1.0	298.0	-	-	46.0	84	-	-	424.0	81	606.6	81	(2.0)	-
129	108	A0034a	CC(C)CCCC(C)(C)C	1.0	298.15	720.0	87	46.4	84	168.2	87	422.1	81	606.6	81	(2.0)	-
130	109	A0035a	CCC(C)C(CC)C(C)C	1.0	298.0	-	-	46.9	84	-	-	433.3	81	606.6	81	(2.0)	-
131	110	A0036a	CCCC(C(C)C)C(C)C	1.0	298.0	-	-	46.4	84	-	-	439.9	81	606.6	81	(2.0)	-
132	111	A0037a	CCC(CC)C(C)C(C)C	1.0	298.0	-	-	46.9	84	-	-	434.1	81	606.6	81	(2.0)	-
133	112	A0038a	CCC(C)C(C)C(C)CC	1.0	298.15	751.9	87	47.3	84	-	-	435.7	81	606.6	81	(2.0)	-
134	113	A0039a	CCCC(C)C(C)C(C)C	1.0	298.0	-	-	47.3	84	-	-	433.1	81	606.6	81	(2.0)	-
135	114	A0040a	CCC(CC(C)C)C(C)C	1.0	298.0	-	-	46.4	84	-	-	427.3	81	606.6	81	(2.0)	-
136	115	A0041a	CCC(C)C(C)CC(C)C	1.0	298.0	-	-	46.9	84	-	-	429.7	81	606.6	81	(2.0)	-
137	116	A0042a	CCC(C)CC(C)C(C)C	1.0	298.0	-	-	47.3	84	-	-	433.9	81	606.6	81	(2.0)	-
138	117	A0043a	CC(C)CCC(C)C(C)C	1.0	298.0	-	-	47.3	84	-	-	429.2	81	606.6	81	(2.0)	-
139	118	A0044a	CC(C)CC(C)CC(C)C	1.0	298.0	-	-	46.4	84	-	-	420.8	81	606.6	81	(2.0)	-
140	119	A0045a	CCCC(CC)(CC)CC	1.0	298.0	-	-	47.3	84	-	-	439.5	81	606.6	81	(2.0)	-
141	120	A0046a	CCCC(C)(CC)CCC	1.0	298.0	-	-	47.2	84	-	-	434.0	81	606.6	81	(2.0)	-

Table S.2 – Reference experimental data (continued)

n_{sim}	n_{iso}	Code	Smiles	P [bar]	T [K]	ρ_{liq} [kg·m^{-3}]	Src	ΔH_{vap} [kJ·mol^{-1}]	Src	T_m [K]	Src	T_b [K]	Src	T_c [K]	Src	ϵ	Src
142	121	A0047a	CCCCC(C)(CC)CC	1.0	298.0	-	-	47.7	84	-	-	437.0	81	606.6	81	(2.0)	-
143	122	A0048a	CCCCC(C)(C)CCC	1.0	298.0	-	-	48.1	84	-	-	430.7	81	606.6	81	(2.0)	-
144	123	A0049a	CCCCCC(C)(C)CC	1.0	298.0	-	-	48.5	84	-	-	434.4	81	606.6	81	(2.0)	-
145	124	A0050a	CCCCCCC(C)(C)C	1.0	298.15	720.8	87	49.0	84	-	-	430.1	81	606.6	81	(2.0)	-
146	125	A0051a	CCC(CC)C(CC)CC	1.0	298.15	747.2	87	47.7	84	-	-	437.1	81	606.6	81	(2.0)	-
147	126	A0052a	CCCC(CC)C(C)CC	1.0	298.0	-	-	47.7	84	-	-	435.4	81	606.6	81	(2.0)	-
148	127	A0053a	CCCC(C)C(CC)CC	1.0	298.0	-	-	48.1	84	-	-	436.2	81	606.6	81	(2.0)	-
149	128	A0054a	CCCC(CCC)C(C)C	1.0	298.15	735.4	87	47.3	84	-	-	432.1	81	606.6	81	(2.0)	-
150	129	A0055a	CCCCC(CC)C(C)C	1.0	298.0	-	-	48.1	84	-	-	434.4	81	606.6	81	(2.0)	-
151	130	A0056a	CCCC(C)C(C)CCC	1.0	298.0	-	-	48.5	84	-	-	435.3	81	606.6	81	(2.0)	-
152	131	A0057a	CCCCC(C)C(C)CC	1.0	298.15	741.0	87	48.1	84	-	-	436.6	81	606.6	81	(2.0)	-
153	132	A0058a	CCCCCC(C)C(C)C	1.0	293.15	737.7	87	48.1	84	-	-	437.5	81	606.6	81	(2.0)	-
154	133	A0059a	CCC(C)CC(CC)CC	1.0	298.0	-	-	47.7	84	-	-	431.4	81	606.6	81	(2.0)	-
155	134	A0060a	CCCC(CC)CC(C)C	1.0	298.0	-	-	47.3	84	-	-	429.4	81	606.6	81	(2.0)	-
156	135	A0061a	CCC(CC)CCC(C)C	1.0	298.0	-	-	48.1	84	-	-	432.9	81	606.6	81	(2.0)	-
157	136	A0062a	CCCC(C)CC(C)CC	1.0	298.0	-	-	48.5	84	-	-	432.6	81	606.6	81	(2.0)	-
158	137	A0063a	CCCCC(C)CC(C)C	1.0	298.15	722.6	87	48.5	84	-	-	429.1	81	606.6	81	(2.0)	-
159	138	A0064a	CCC(C)CCC(C)CC	1.0	298.15	732.4	87	47.3	84	-	-	434.0	81	606.6	81	(2.0)	-
160	139	A0065a	CCCC(C)CCC(C)C	1.0	298.15	726.4	87	49.0	84	-	-	431.7	81	606.6	81	(2.0)	-
161	140	A0066a	CCC(C)CCCC(C)C	1.0	293.15	731.3	87	49.3	84	-	-	433.5	81	606.6	81	(2.0)	-
162	141	A0067a	CC(C)CCCCC(C)C	1.0	298.15	720.2	87	47.7	84	219.2	87	433.0	81	606.6	81	2.0	87
163	142	A0068a	CCCC(CCC)CCC	1.0	298.15	732.1	87	48.5	84	-	-	430.7	81	606.6	81	2.0	87
164	143	A0069a	CCCCC(CC)CCC	1.0	298.15	734.3	87	48.1	84	-	-	436.8	81	606.6	81	(2.0)	-
165	144	A0070a	CCCCCC(CC)CC	1.0	298.15	735.9	87	49.0	84	-	-	439.7	81	606.6	81	(2.0)	-
166	145	A0071a	CCCCC(C)CCCC	1.0	293.15	732.6	87	49.8	84	186.7	87	438.3	81	606.6	81	(2.0)	-
167	146	A0072a	CCCCCC(C)CCC	1.0	293.15	732.3	87	49.5	84	174.8	87	438.9	81	606.6	81	(2.0)	-
168	147	A0073a	CCCCCCC(C)CC	1.0	293.15	735.4	87	50.2	84	188.4	87	440.9	81	606.6	81	(2.0)	-
169	148	A0074a	CCCCCCCC(C)C	1.0	293.15	728.1	87	51.0	84	198.8	87	440.1	81	606.6	81	(2.0)	-
170	149	A0075a	CCCCCCCCCC	1.01	298.15	725.9	78	50.2	84	243.5	87	447.3	81	617.7	81	2.0	86
171	150	F1101a	CF	1.0	196.93	876.59	77	17.1	84	129.8	87	194.8	81	317.4	81	51.0	87
172	150	F1101b	CF	38.39	298.15	528.33	81	-	-	129.8	87	194.8	81	317.4	81	51.0	87
173	150	F1101c	CF	1.0	298.15	574.41	77	-	-	129.8	87	194.8	81	317.4	81	51.0	87
174	151	F1201a	FCF	1.0	221.17	1213.8	77	20.88	84	136.3	87	221.5	81	351.3	81	53.7	87
175	151	F1201b	FCF	17.62	298.15	891.63	81	-	-	136.3	87	221.5	81	351.3	81	53.7	87
176	151	F1201c	FCF	1.0	298.15	961.0	77	-	-	136.3	87	221.5	81	351.3	81	53.7	87
177	152	F1301a	FC(F)F	1.0	191.15	1442.9	77	16.7	84	118.0	87	191.0	81	299.0	81	5.2	87
178	152	F1301b	FC(F)F	47.1	298.15	636.67	81	-	-	118.0	87	191.0	81	299.0	81	5.2	87
179	152	F1301c	FC(F)F	1.0	298.15	666.7	77	-	-	118.0	87	191.0	81	299.0	81	5.2	87
180	153	F1401a	FC(F)(F)F	1.0	145.0	1605.17	77	12.3	84	89.6	87	145.1	81	227.5	81	1.7	87
181	154	F2101a	CCF	1.0	236.05	817.6	77	20.7	84	129.9	87	235.4	81	375.3	81	(6.1)	-
182	154	F2101b	CCF	9.09	298.15	707.47	81	-	-	129.9	87	235.4	81	375.3	81	(6.1)	-
183	155	F2201a	CC(F)F	1.0	249.66	1009.0	77	22.7	84	154.6	87	247.3	81	386.4	81	(6.1)	-
184	155	F2201b	CC(F)F	6.25	298.15	907.0	81	-	-	154.6	87	247.3	81	386.4	81	(6.1)	-
185	156	F2301a	CC(F)(F)F	1.0	220.16	1182.86	77	19.2	84	161.6	87	225.8	81	345.9	81	10.6	78
186	156	F2301b	CC(F)(F)F	12.92	298.15	-	-	13.12	81	161.6	87	225.8	81	345.9	81	10.6	78
187	157	F3101a	CC(C)F	1.0	263.75	769.2	77	-	-	-	-	263.8	81	421.1	81	(6.1)	-
188	158	F3102a	CCCF	1.0	270.65	781.8	77	-	-	114.2	87	269.9	81	421.1	81	(6.1)	-
189	159	F3201a	CCC(F)F	0.62	276.3	-	-	25.12	81	-	-	281.1	81	430.4	81	(6.1)	-

Table S.2 – Reference experimental data (continued)

n_{sim}	n_{iso}	Code	Smiles	P [bar]	T [K]	ρ_{liq} [kg·m^{-3}]	Src	ΔH_{vap} [kJ·mol^{-1}]	Src	T_m [K]	Src	T_b [K]	Src	T$_c$ [K]	Src	ϵ	Src
190	160	F3202a	FCCCF	1.0	298.15	1005.7	77	-	-	-	-	314.4	81	430.4	81	(6.1)	-
191	161	F4101a	CC(C)(C)F	1.0	285.25	752.7	77	-	-	-	-	285.2	81	460.3	81	(6.1)	-
192	161	F4101b	CC(C)(C)F	1.39	298.15	735.3	81	-	-	-	-	285.2	81	460.3	81	(6.1)	-
193	162	F4102a	CCC(C)F	1.0	298.15	756.56	77	-	-	151.8	87	298.2	81	460.3	81	(6.1)	-
194	163	F4103a	CCCCF	1.0	298.15	770.82	77	-	-	139.2	87	305.6	81	460.3	81	(6.1)	-
195	164	F4201a	FCCCCF	1.0	298.15	976.7	77	-	-	-	-	350.9	81	463.6	81	(6.1)	-
196	165	F4301a	CCCC(F)(F)F	1.38	298.15	1010.0	81	-	-	-	-	289.9	81	406.6	81	(6.1)	-
197	166	F5101a	CCC(C)(C)F	1.0	298.15	773.73	77	-	-	-	-	317.9	81	494.6	81	(3.9)	-
198	167	F5102a	CCC(C)CF	0.34	298.15	791.47	81	-	-	-	-	329.1	81	494.6	81	(3.9)	-
199	168	F5103a	CCCCCF	1.0	298.15	784.9	77	30.93	81	153.2	87	335.9	81	494.6	81	3.9	87
200	169	F6101a	CCCCC(C)F	1.0	293.15	791.4	77	-	-	-	-	359.4	81	525.4	81	(6.1)	-
201	170	F6102a	CCCCCCF	1.0	298.15	795.79	77	35.57	81	170.2	87	364.6	81	525.4	81	(6.1)	-
202	171	F6201a	CC(C)CC(C)(F)F	1.0	293.15	888.2	77	-	-	-	-	-	-	-	-	(6.1)	-
203	172	F6202a	FCCCCCCF	1.0	298.15	940.7	77	-	-	-	-	-	-	-	-	(6.1)	-
204	173	F7101a	CCCCCCCF	1.0	298.15	800.88	77	40.8	84	200.2	87	391.1	81	553.5	81	(6.1)	-
205	174	F8101a	CCCCCCCCF	1.0	298.15	806.69	77	-	-	209.2	87	415.4	81	579.2	81	3.9	87
206	175	F9101a	CCCCCCCCCF	1.0	348.0	-	-	46.8	84	-	-	438.1	81	603.2	81	(6.1)	-
207	176	F0101a	CCCCCCCCCCF	1.0	293.15	819.4	87	-	-	238.2	87	459.4	81	625.5	81	(6.1)	-
208	177	C1101a	CCl	1.0	248.77	1008.26	77	-	-	175.6	87	248.9	81	416.2	81	10.0	87
209	178	C1201a	ClCCl	1.0	298.15	1316.4	77	28.8	84	178.2	87	312.9	81	510.0	81	8.9	87
210	179	C1301a	ClC(Cl)Cl	1.0	298.15	1479.5	77	31.1	84	209.8	87	334.3	81	536.4	81	4.8	87
211	180	C1401a	ClC(Cl)(Cl)Cl	1.0	298.15	1584.31	77	32.44	81	250.3	87	349.8	81	556.4	81	2.2	87
212	181	C2101a	CCCl	1.0	285.45	906.2	77	24.9	84	134.8	87	285.4	81	460.4	81	9.4	87
213	181	C2101b	CCCl	1.39	298.15	889.97	81	-	-	134.8	87	285.4	81	460.4	81	9.4	87
214	182	C2201a	CC(Cl)Cl	1.0	298.15	1168.07	77	30.6	84	176.2	87	330.4	81	523.0	81	10.1	87
215	183	C2202a	ClCCCl	1.0	298.15	1245.58	77	34.4	84	237.6	87	356.6	81	561.6	81	10.4	87
216	184	C2301a	CC(Cl)(Cl)Cl	1.0	298.15	1329.32	77	32.4	84	243.2	87	347.2	81	545.0	81	7.2	87
217	185	C2302a	ClCC(Cl)Cl	1.0	298.15	1432.75	77	40.1	84	236.8	87	387.0	81	551.3	81	7.2	87
218	186	C3101a	CC(C)Cl	1.0	298.15	855.63	77	-	-	156.1	87	308.9	81	496.5	81	(8.6)	-
219	187	C3201a	CC(Cl)CCl	1.0	298.15	1153.0	77	36.2	84	172.6	87	369.5	81	573.3	81	8.4	87
220	188	C3202a	ClCCCCl	1.0	298.15	1180.0	77	40.6	84	173.7	87	393.6	81	573.3	81	10.3	87
221	189	C4101a	CC(C)(C)Cl	1.0	298.15	836.3	77	28.6	84	247.6	87	323.8	81	530.2	81	9.7	87
222	190	C4102a	CC(C)CCl	1.0	298.15	871.35	77	31.7	84	142.8	87	342.0	81	530.2	81	7.0	87
223	191	C4103a	CCC(C)Cl	1.01	298.15	867.47	78	31.5	84	141.8	87	341.2	81	520.6	81	8.6	87
224	192	C4104a	CCCCCl	1.01	298.15	880.4	78	33.5	84	150.1	87	351.6	81	530.2	81	7.3	87
225	193	C4201a	CC(Cl)C(C)Cl	1.0	298.15	1106.3	77	-	-	-	-	391.1	81	599.9	81	(8.1)	-
226	194	C4202a	CCC(Cl)CCl	1.0	298.15	1111.8	77	40.1	84	-	-	397.1	81	599.9	81	7.7	87
227	195	C4203a	ClCCCCCl	1.0	298.15	-	-	46.4	84	234.4	87	427.1	81	599.9	81	9.3	87
228	196	C5101a	CCC(C)(C)Cl	1.0	298.15	859.65	77	-	-	200.6	87	358.8	81	560.5	81	12.3	87
229	197	C5102a	CCC(C)CCl	1.0	298.15	875.0	77	-	-	-	-	373.7	81	560.5	81	(8.3)	-
230	198	C5103a	CC(C)CCCl	1.0	298.15	870.0	77	36.2	84	168.8	87	371.7	81	560.5	81	6.1	87
231	199	C5104a	CCCC(C)Cl	1.0	298.15	866.0	77	36.0	84	-	-	369.7	81	560.5	81	(8.3)	-
232	200	C5105a	CCCCCCl	1.0	298.0	877.8	77	38.2	84	174.2	87	381.5	81	560.5	81	6.7	87
233	201	C5201a	ClCCCCCCl	1.0	298.15	1095.6	87	51.3	84	200.3	87	453.1	81	624.5	81	9.9	87
234	202	C6101a	CCCCCCCl	1.0	298.15	873.54	77	42.0	84	179.2	87	408.2	81	588.0	81	6.1	87
235	203	C6201a	CC(CCl)C(C)(C)Cl	1.0	298.15	1064.0	77	-	-	-	-	-	-	-	-	(8.6)	-
236	204	C6202a	CC(Cl)CCC(C)Cl	1.0	298.15	1044.1	77	-	-	-	-	-	-	-	-	(8.6)	-
237	205	C6203a	ClCCCCCCCl	1.01	298.15	1063.7	78	-	-	-	-	477.1	81	647.4	81	8.6	87

Table S.2 – Reference experimental data (continued)

$n_{\rm sim}$	$n_{\rm iso}$	Code	Smiles	P [bar]	T [K]	$\rho_{\rm liq}$ [kg·m^{-3}]	Src	$\Delta H_{\rm vap}$ [kJ·mol^{-1}]	Src	T_m [K]	Src	T_b [K]	Src	T$_c$ [K]	Src	ϵ	Src
238	206	C7101a	CCCCCCCCl	1.0	298.15	871.5	77	47.0	84	203.8	87	433.6	81	613.5	81	5.5	87
239	207	C8101a	CCCCCCCCCl	1.0	298.15	869.35	77	51.4	84	215.3	87	456.6	81	637.1	81	5.0	87
240	208	C9101a	CCCCCCCCCCl	1.0	298.15	867.4	87	-	-	233.8	87	478.4	81	659.3	81	4.8	87
241	209	C0101a	CCCCCCCCCCCl	1.0	298.15	865.84	77	64.0	84	241.8	87	499.0	81	680.1	81	4.6	87
242	210	B1101a	CBr	1.0	273.23	1729.8	77	-	-	179.4	87	276.7	81	467.0	81	9.7	87
243	210	B1101b	CBr	2.25	298.15	1662.08	81	-	-	179.4	87	276.7	81	467.0	81	9.7	87
244	210	B1101c	CBr	1.0	281.0	-	-	24.6	84	179.4	87	276.7	81	467.0	81	9.7	87
245	211	B1201a	BrCBr	0.06	298.15	2482.01	81	37.0	84	221.1	87	370.1	81	615.9	81	7.8	87
246	212	B1301a	BrC(Br)Br	1.0	298.15	2877.15	77	46.1	84	281.8	87	422.4	81	656.4	81	4.4	87
247	213	B1401a	BrC(Br)(Br)Br	1.0	373.85	2953.3	77	-	-	363.1	87	462.6	81	706.8	81	(2.2)	-
248	213	B1401b	BrC(Br)(Br)Br	1.0	384.0	-	-	48.2	84	363.1	87	462.6	81	706.8	81	(2.2)	-
249	214	B2101a	CCBr	1.0	298.15	1451.2	77	-	-	154.8	87	311.5	81	503.8	81	9.0	87
250	214	B2101b	CCBr	1.0	305.0	-	-	27.6	84	154.8	87	311.5	81	503.8	81	9.0	87
251	215	B2201a	CC(Br)Br	1.0	298.15	2091.75	77	-	-	210.2	87	381.1	81	635.8	81	(5.0)	-
252	216	B2202a	BrCCBr	1.0	298.15	2169.52	77	41.7	84	282.9	87	404.5	81	650.1	81	5.0	87
253	217	B2301a	BrCC(Br)Br	1.0	298.15	2610.1	77	-	-	243.9	87	462.1	81	671.9	81	(6.8)	-
254	218	B3101a	CC(C)Br	1.0	298.15	1301.37	77	30.2	84	184.2	87	332.6	81	532.5	81	9.5	87
255	219	B3102a	CCCBr	1.0	298.15	1345.5	77	31.9	84	163.1	87	344.1	81	536.9	81	8.1	87
256	220	B3201a	CC(Br)CBr	1.0	298.15	1924.95	77	41.7	84	217.8	87	413.2	81	654.7	81	4.6	87
257	221	B3202a	BrCCCBr	1.0	298.15	1971.19	77	-	-	238.2	87	440.4	81	654.7	81	9.5	87
258	222	B3301a	CC(Br)(Br)CBr	1.0	293.15	2298.54	77	-	-	-	-	464.1	81	686.8	81	(6.0)	-
259	223	B3302a	CC(Br)C(Br)Br	1.0	293.15	2354.8	77	-	-	-	-	474.1	81	686.8	81	(6.0)	-
260	224	B3303a	BrCC(Br)CBr	1.0	298.15	2411.04	77	-	-	287.1	87	495.3	81	686.8	81	6.0	87
261	225	B4101a	CC(C)(C)Br	1.0	298.15	1212.5	77	31.8	84	256.4	87	346.4	81	557.6	81	11.0	87
262	226	B4102a	CC(C)CBr	1.0	298.15	1257.09	77	34.9	84	155.3	87	364.7	81	557.6	81	7.7	87
263	227	B4103a	CCC(C)Br	1.0	298.15	1253.6	77	34.8	84	-	-	364.4	81	557.6	81	(8.7)	-
264	228	B4104a	CCCCBr	1.0	298.15	1268.6	77	36.6	84	160.7	87	374.8	81	557.6	81	7.3	87
265	229	B4201a	CC(CBr)CBr	1.0	298.15	1799.5	77	-	-	-	-	448.1	81	672.7	81	(6.6)	-
266	230	B4202a	CCC(Br)CBr	1.0	298.15	1787.0	77	45.6	84	207.8	87	439.5	81	672.7	81	4.7	87
267	231	B4203a	CC(Br)CCBr	1.0	293.15	1796.0	77	-	-	-	-	448.1	81	672.7	81	9.1	87
268	232	B4301a	CC(C)(Br)C(Br)Br	1.0	294.15	2175.3	77	-	-	-	-	503.1	81	701.1	81	(6.8)	-
269	233	B4302a	CC(Br)(CBr)CBr	1.0	298.15	2180.3	77	-	-	-	-	493.1	81	701.1	81	(6.8)	-
270	234	B5101a	CC(C)(C)CBr	0.03	298.15	1193.46	81	-	-	-	-	379.1	81	581.0	81	(7.5)	-
271	235	B5102a	CCC(C)(C)Br	0.03	298.15	1209.52	81	-	-	-	-	381.1	81	581.0	81	9.2	87
272	236	B5103a	CCC(C)CBr	1.0	298.15	1214.4	77	-	-	-	-	393.6	81	581.0	81	(7.5)	-
273	237	B5104a	CCC(Br)CC	1.0	298.15	1205.1	77	-	-	146.9	87	391.8	81	581.0	81	8.4	87
274	238	B5105a	CC(C)CCBr	1.0	298.15	1200.7	77	-	-	161.2	87	393.6	81	581.0	81	6.3	87
275	239	B5106a	CCCC(C)Br	1.0	298.15	1200.5	77	38.5	84	177.7	87	390.6	81	581.0	81	(7.5)	-
276	240	B5107a	CCCCCBr	1.0	298.15	1211.4	77	40.9	84	185.2	87	402.7	81	581.0	81	6.3	87
277	241	B5201a	CC(C)(C)C(Br)Br	1.0	293.15	1669.5	77	-	-	-	-	453.1	81	689.9	81	(7.5)	-
278	242	B6101a	CCC(C)(Br)CC	1.0	293.15	1179.2	77	-	-	-	-	403.1	81	602.9	81	(5.8)	-
279	243	B6102a	CCCC(Br)CC	1.0	298.15	1157.16	77	-	-	-	-	414.4	81	602.9	81	(5.8)	-
280	244	B6103a	CCCCCCBr	1.0	298.15	1168.8	77	45.6	84	188.2	87	428.4	81	602.9	81	5.8	87
281	245	B7101a	CCCCCCCBr	1.0	298.15	1134.8	77	50.4	84	217.1	87	452.1	81	623.5	81	5.3	87
282	246	B8101a	CCCCCCCCBr	1.0	298.15	1107.7	77	55.1	84	218.2	87	473.9	81	643.0	81	5.1	87
283	247	B9101a	CCCCCCCCCBr	1.0	298.15	1084.86	77	-	-	244.2	87	494.6	81	661.6	81	4.7	87
284	248	B0101a	CCCCCCCCCCBr	1.0	298.15	1062.48	77	-	-	243.8	87	513.8	81	679.3	81	4.4	87
285	248	B0101b	CCCCCCCCCCBr	1.0	398.0	-	-	56.6	84	243.8	87	513.8	81	679.3	81	4.4	87

Table S.2 – Reference experimental data (continued)

n_{sim}	n_{iso}	Code	Smiles	P [bar]	T [K]	ρ_{liq} [kg·m^{-3}]	Src	ΔH_{vap} [kJ·mol^{-1}]	Src	T_m [K]	Src	T_b [K]	Src	T$_c$ [K]	Src	ϵ	Src
286	249	I1101a	CI	1.0	298.15	2264.5	77	27.48	81	207.2	87	315.6	81	522.4	81	7.0	87
287	250	I1201a	ICI	1.0	298.15	3307.85	77	49.0	84	279.1	87	455.1	81	697.5	81	5.3	87
288	251	I2101a	CCI	1.0	298.15	1924.04	77	31.7	81	162.2	87	345.4	81	562.2	81	7.8	87
289	252	I3101a	CC(C)I	1.0	298.15	1694.54	77	34.1	84	182.8	87	362.6	81	583.8	81	8.2	87
290	253	I3102a	CCCI	1.0	298.15	1737.25	77	36.01	81	171.8	87	375.6	81	602.3	81	7.1	87
291	254	I3201a	ICCCI	1.0	298.15	2565.1	77	-	-	253.2	87	496.1	81	775.2	81	(6.5)	-
292	254	I3201b	ICCCI	0.01	368.52	-	-	53.5	81	253.2	87	496.1	81	775.2	81	(6.5)	-
293	255	I4101a	CC(C)(C)I	0.06	298.15	1536.03	81	35.71	81	239.6	87	373.2	81	596.8	81	6.7	87
294	256	I4102a	CC(C)CI	0.02	298.15	1595.11	81	38.8	84	-	-	393.6	81	625.0	81	(6.5)	-
295	257	I4103a	CCC(C)I	1.0	298.15	1589.0	77	38.5	84	-	-	393.1	81	623.3	81	(6.5)	-
296	258	I4104a	CCCCI	1.0	298.15	1606.74	77	40.3	84	169.7	87	403.7	81	638.8	81	6.3	87
297	259	I4201a	ICCCCI	1.0	298.15	2349.57	77	59.0	84	279.1	87	477.2	81	806.4	81	(6.5)	-
298	260	I5101a	CC(C)(C)CI	1.0	293.15	1494.0	87	-	-	-	-	407.1	81	670.9	81	(6.8)	-
299	261	I5102a	CCC(C)(C)I	0.01	298.15	1486.59	81	-	-	-	-	402.1	81	670.9	81	8.2	87
300	262	I5103a	CC(C)C(C)I	1.0	293.15	1524.0	77	-	-	-	-	413.1	81	670.9	81	(6.8)	-
301	263	I5104a	CCC(I)CC	1.0	298.15	1505.48	77	-	-	-	-	418.1	81	670.9	81	7.4	87
302	264	I5105a	CC(C)CCI	1.0	298.15	1495.15	77	42.2	84	-	-	421.4	81	659.1	81	5.6	87
303	265	I5106a	CCCC(C)I	1.0	293.15	1500.9	77	-	-	-	-	416.1	81	670.9	81	(6.8)	-
304	266	I5107a	CCCCCI	1.0	298.15	1507.35	77	44.4	84	187.6	87	430.1	81	671.4	81	5.8	87
305	267	I5201a	ICCCCCI	1.0	298.15	2173.44	77	-	-	282.1	87	500.1	81	843.6	81	(6.5)	-
306	268	I6101a	CCCC(C)CI	1.0	293.15	1443.0	77	-	-	-	-	441.1	81	697.2	81	(5.3)	-
307	269	I6102a	CCCCC(C)I	1.0	293.15	1419.3	77	-	-	-	-	442.1	81	697.2	81	(5.3)	-
308	270	I6103a	CCCCCCI	1.0	298.15	1431.81	77	-	-	199.1	87	454.5	81	704.4	81	5.3	87
309	270	I6103b	CCCCCCI	1.0	346.0	-	-	46.2	84	199.1	87	454.5	81	704.4	81	5.3	87
310	271	I6201a	ICCCCCCI	1.0	298.15	2034.2	87	-	-	282.6	87	522.5	81	831.8	81	(6.5)	-
311	272	I7101a	CCCCCCCI	1.0	298.15	1371.9	87	-	-	224.9	87	477.1	81	736.7	81	4.9	87
312	272	I7101b	CCCCCCCI	0.01	356.73	-	-	48.44	81	224.9	87	477.1	81	736.7	81	4.9	87
313	273	I8101a	CCCCCCCCI	1.0	298.15	1326.65	77	-	-	227.4	87	498.3	81	764.8	81	4.7	87
314	273	I8101b	CCCCCCCCI	0.01	373.66	-	-	50.92	81	227.4	87	498.3	81	764.8	81	4.7	87
315	274	I9101a	CCCCCCCCCI	1.0	298.15	1283.6	87	-	-	253.2	87	518.1	81	-	-	(6.5)	-
316	275	I0101a	CCCCCCCCC(C)I	1.0	298.15	1241.2	77	-	-	-	-	-	-	-	-	(6.5)	-
317	276	I0102a	CCCCCCCCCCI	1.0	293.15	1256.7	77	69.8	84	256.9	87	536.9	81	-	-	(6.5)	-
318	277	O2101a	COC	1.0	248.12	735.04	78	21.56	81	131.7	87	248.3	81	400.1	81	6.2	87
319	277	O2101b	COC	9.06	298.28	661.92	78	17.75	81	131.7	87	248.3	81	400.1	81	6.2	87
320	278	O3101a	CCOC	1.0	273.15	726.0	75	-	-	160.2	87	280.5	81	437.8	81	(3.8)	-
321	278	O3101b	CCOC	1.82	298.15	691.94	81	-	-	160.2	87	280.5	81	437.8	81	(3.8)	-
322	278	O3101c	CCOC	1.0	280.5	-	-	31.22	81	160.2	87	280.5	81	437.8	81	(3.8)	-
323	279	O3201a	COCOC	0.51	298.15	854.12	81	28.9	84	168.0	87	315.0	81	480.6	81	2.6	87
324	280	O4101a	COC(C)C	1.0	298.15	708.95	75	26.4	84	-	-	303.9	81	464.5	81	(4.3)	-
325	281	O4102a	CCCOC	1.0	298.15	719.22	75	27.9	84	-	-	311.7	81	476.2	81	(4.3)	-
326	282	O4103a	CCOCC	1.0	298.15	707.82	75	27.2	84	156.9	87	307.6	81	466.7	81	4.3	87
327	283	O4201a	COC(C)OC	1.0	293.15	851.6	75	36.4	84	159.9	87	337.6	81	-	-	(7.3)	-
328	284	O4202a	COCCOC	1.01	298.15	861.36	78	36.8	84	204.2	87	357.2	81	536.1	81	7.3	87
329	285	O5101a	COC(C)(C)C	1.0	298.15	735.22	75	30.4	84	164.6	87	328.4	81	497.1	81	(3.8)	-
330	286	O5102a	COCC(C)C	1.0	298.15	727.2	75	-	-	-	-	331.7	81	500.0	81	(3.8)	-
331	287	O5103a	CCC(C)OC	1.0	298.15	736.7	75	-	-	-	-	332.1	81	500.0	81	(3.8)	-
332	288	O5104a	CCOC(C)C	1.0	298.15	717.3	75	30.0	84	-	-	326.1	81	500.0	81	(3.8)	-
333	289	O5105a	CCCCOC	1.0	298.15	739.4	75	32.5	84	157.4	87	343.4	81	512.7	81	(3.8)	-

Table S.2 – Reference experimental data (continued)

n_{sim}	n_{iso}	Code	Smiles	P [bar]	T [K]	ρ_{liq} [kg·m^{-3}]	Src	ΔH_{vap} [kJ·mol^{-1}]	Src	T_m [K]	Src	T_b [K]	Src	T$_c$ [K]	Src	ϵ	Src
334	290	O5106a	CCCOCC	1.0	298.15	727.0	75	31.4	84	145.7	87	337.0	81	500.2	81	(3.8)	-
335	291	O5201a	COC(C)(C)OC	1.01	298.15	845.09	78	37.6	84	226.2	87	356.1	81	-	-	(2.5)	-
336	292	O5202a	CCOCCOC	1.0	298.15	846.0	87	39.8	84	-	-	375.2	81	-	-	(2.5)	-
337	293	O5203a	CCOCOCC	1.0	298.15	825.17	75	35.7	84	207.2	87	361.1	81	524.0	81	2.5	87
338	294	O6101a	CCC(C)(C)OC	1.0	298.15	765.9	75	35.0	84	-	-	359.4	81	526.0	81	(3.6)	-
339	295	O6102a	CCOC(C)(C)C	1.0	298.15	735.16	75	33.1	84	179.2	87	345.9	81	526.0	81	(3.6)	-
340	296	O6103a	COC(C)C(C)C	1.0	298.15	754.2	75	-	-	-	-	356.1	81	526.0	81	(3.6)	-
341	297	O6104a	CC(C)OC(C)C	1.0	298.15	718.7	75	32.7	84	187.8	87	341.4	81	500.1	81	3.8	87
342	298	O6105a	CCC(C)COC	0.07	298.15	746.09	81	-	-	-	-	363.1	81	526.0	81	(3.6)	-
343	299	O6106a	COCCC(C)C	1.0	298.15	749.05	75	-	-	-	-	363.1	81	526.0	81	(3.6)	-
344	300	O6107a	CCOCC(C)C	1.0	298.15	734.03	75	-	-	-	-	354.2	81	526.0	81	(3.6)	-
345	301	O6108a	CCCC(C)OC	0.06	298.15	749.89	81	-	-	-	-	364.1	81	526.0	81	(3.6)	-
346	302	O6109a	CCOC(C)CC	1.0	298.15	738.3	75	-	-	-	-	354.4	81	526.0	81	(3.6)	-
347	303	O6110a	CCCOC(C)C	1.0	298.15	732.4	75	-	-	-	-	353.1	81	526.0	81	(3.6)	-
348	304	O6111a	CCCCCOC	1.0	298.15	755.2	75	-	-	-	-	372.1	81	546.5	81	(3.6)	-
349	305	O6112a	CCCCOCC	1.0	298.15	744.7	75	36.3	84	149.2	87	365.4	81	526.0	81	(3.6)	-
350	306	O6113a	CCCOCCC	1.0	298.15	741.94	75	35.7	84	158.3	87	362.8	81	530.6	81	3.4	87
351	307	O6201a	COC(OC)C(C)C	1.0	293.15	844.6	75	-	-	-	-	-	-	-	-	(3.9)	-
352	308	O6202a	CCOC(C)OCC	1.01	298.15	821.96	78	39.6	84	167.1	87	376.8	81	539.7	81	(3.9)	-
353	309	O6203a	COCCCCOC	1.0	298.15	852.9	75	-	-	-	-	-	-	-	-	(3.9)	-
354	310	O6204a	CCOCCOCC	1.0	298.15	836.2	75	43.2	84	199.2	87	392.6	81	637.8	81	3.9	87
355	311	O6301a	COCCOCCOC	1.0	298.15	939.24	75	48.0	84	209.2	87	432.9	81	608.0	81	7.2	87
356	312	O7101a	CC(C)OC(C)(C)C	1.0	298.15	736.39	75	34.5	84	184.8	87	378.7	81	549.7	81	(3.8)	-
357	313	O7102a	CCOC(C)(C)CC	1.0	298.15	761.8	75	38.2	84	-	-	375.1	81	546.0	81	(3.8)	-
358	314	O7103a	CCCOC(C)(C)C	1.0	298.15	746.68	75	37.2	84	-	-	373.1	81	549.7	81	(3.8)	-
359	315	O7104a	CC(C)COC(C)C	1.0	298.15	734.9	75	-	-	-	-	371.2	81	549.7	81	(3.8)	-
360	316	O7105a	CCC(C)OC(C)C	1.0	298.15	739.6	75	-	-	-	-	-	-	549.7	81	(3.8)	-
361	317	O7106a	CCOCCC(C)C	1.0	298.15	752.1	75	-	-	-	-	385.6	81	549.7	81	4.0	87
362	318	O7107a	CCCOCC(C)C	1.0	298.15	744.0	75	-	-	-	-	378.1	81	549.7	81	(3.8)	-
363	319	O7108a	CCCOC(C)CC	1.0	298.15	750.1	75	-	-	-	-	-	-	-	-	(3.8)	-
364	320	O7109a	CCCCOC(C)C	1.0	298.15	746.0	75	-	-	-	-	380.1	87	-	-	(3.8)	-
365	321	O7110a	CCCCCCOC	1.0	298.15	766.3	75	-	-	-	-	398.1	87	-	-	(3.8)	-
366	322	O7111a	CCCCCOCC	1.0	298.15	757.2	75	-	-	-	-	391.1	87	-	-	3.6	87
367	323	O7112a	CCCCOCCC	1.0	298.15	754.2	75	-	-	-	-	390.1	87	-	-	(3.8)	-
368	324	O7201a	COCCOC(C)(C)C	1.0	404.65	-	-	34.8	81	-	-	404.6	81	-	-	(4.1)	-
369	325	O7202a	CCOC(C)(C)OCC	1.0	298.15	868.8	75	43.9	84	-	-	387.1	81	-	-	(4.1)	-
370	326	O7203a	CC(C)OCOC(C)C	1.0	298.15	813.72	75	-	-	-	-	-	-	-	-	(4.1)	-
371	327	O7204a	COCCCCCOC	1.0	298.15	851.6	75	-	-	-	-	-	-	-	-	(4.1)	-
372	328	O7205a	CCCCOCCOC	1.0	298.15	-	-	47.8	84	-	-	420.1	81	659.3	81	(4.1)	-
373	329	O7206a	CCCOCCCOC	1.0	298.15	840.3	75	-	-	-	-	-	-	-	-	(4.1)	-
374	330	O7207a	CCOCCCOCC	1.0	298.15	831.2	75	45.9	84	-	-	-	-	-	-	(4.1)	-
375	331	O7208a	CCCOCCOCC	1.0	298.15	832.7	75	46.8	84	-	-	-	-	-	-	(4.1)	-
376	332	O7301a	CCOC(OCC)OCC	1.0	298.15	893.8	75	47.8	84	-	-	416.1	81	-	-	4.8	87
377	333	O7302a	CCOCCOCCOC	1.0	293.15	922.9	75	-	-	-	-	416.9	81	-	-	(4.8)	-
378	334	O8101a	CC(C)(C)OC(C)(C)C	1.0	298.15	757.8	75	37.6	84	-	-	380.4	81	550.0	81	(3.1)	-
379	335	O8102a	CC(C)COC(C)(C)C	1.0	298.15	748.0	75	40.1	84	-	-	439.9	81	571.4	81	(3.1)	-
380	336	O8103a	CCC(C)OC(C)(C)C	1.0	298.15	757.1	75	-	-	-	-	-	-	-	-	(3.1)	-
381	337	O8104a	CCCCOC(C)(C)C	1.0	298.15	758.1	75	42.3	84	-	-	-	-	571.4	81	(3.1)	-

Table S.2 – Reference experimental data (continued)

n_{sim}	n_{iso}	Code	Smiles	P [bar]	T [K]	ρ_{liq} [kg·m^{-3}]	Src	ΔH_{vap} [kJ·mol^{-1}]	Src	T_m [K]	Src	T_b [K]	Src	T$_c$ [K]	Src	ϵ	Src
382	338	O8105a	CC(C)COCC(C)C	1.0	298.15	745.2	75	41.2	84	-	-	395.9	81	571.4	81	(3.1)	-
383	339	O8106a	CCC(C)OCC(C)C	1.0	298.15	750.84	75	-	-	-	-	-	-	-	-	(3.1)	-
384	340	O8107a	CC(C)CCOC(C)C	1.0	298.15	754.5	75	-	-	-	-	-	-	-	-	(3.1)	-
385	341	O8108a	CCC(C)OC(C)CC	1.0	298.15	758.8	75	-	-	-	-	394.2	81	571.4	81	(3.1)	-
386	342	O8109a	CCCOCCC(C)C	1.0	298.15	760.1	75	-	-	-	-	-	-	-	-	(3.1)	-
387	343	O8110a	CCCCOCC(C)C	1.0	298.15	755.2	75	-	-	-	-	424.1	81	571.4	81	(3.1)	-
388	344	O8111a	CCCCOC(C)CC	1.0	298.15	761.1	75	-	-	-	-	-	-	-	-	(3.1)	-
389	345	O8112a	CCCCCOC(C)C	1.0	298.15	759.8	75	-	-	-	-	-	-	571.4	81	(3.1)	-
390	346	O8113a	CCCCCCCOC	1.0	298.15	775.6	75	-	-	-	-	424.1	81	-	-	(3.1)	-
391	347	O8114a	CCCCCCOCC	1.0	298.15	768.2	75	-	-	-	-	415.1	87	-	-	(3.1)	-
392	348	O8115a	CCCCCOCCC	1.0	298.15	765.3	75	-	-	-	-	-	-	571.4	81	(3.1)	-
393	349	O8116a	CCCCOCCCC	1.0	298.15	764.06	75	44.7	84	177.2	87	413.4	81	584.1	81	3.1	87
394	350	O8201a	CCCOC(C)OCCC	1.0	298.15	825.58	75	-	-	-	-	478.1	81	-	-	(4.1)	-
395	351	O8202a	COCCCCCCOC	1.0	298.15	851.8	75	-	-	-	-	-	-	-	-	(4.1)	-
396	352	O8203a	CCOCCCCCOC	1.0	298.15	843.3	75	-	-	-	-	-	-	-	-	(4.1)	-
397	353	O8204a	CCCOCCCCOC	1.0	298.15	840.7	75	-	-	-	-	-	-	-	-	(4.1)	-
398	354	O8205a	CCOCCCCOCC	1.0	298.15	833.5	75	-	-	-	-	-	-	-	-	(4.1)	-
399	355	O8206a	CCCCOCCOCC	1.0	298.15	833.1	75	50.9	84	-	-	434.0	81	-	-	(4.1)	-
400	356	O8207a	CCCOCCOCCC	1.0	298.15	831.2	87	50.6	84	-	-	478.1	81	-	-	(4.1)	-
401	357	O8301a	CCOCCOCCOCC	1.0	298.15	903.3	75	56.4	84	228.8	87	462.1	81	624.0	81	(6.0)	-
402	358	O9101a	COC(C)(C)CC(C)(C)C	1.01	298.15	792.4	78	-	-	-	-	-	-	-	-	(3.8)	-
403	359	O9102a	CC(C)CCOC(C)(C)C	1.0	298.15	761.6	75	-	-	-	-	-	-	-	-	(3.8)	-
404	360	O9103a	CCCCCOC(C)(C)C	1.0	298.15	766.4	75	-	-	-	-	-	-	-	-	(3.8)	-
405	361	O9104a	CC(C)CCOCC(C)C	1.0	298.15	759.4	75	-	-	-	-	-	-	-	-	(3.8)	-
406	362	O9105a	CCC(C)OCCC(C)C	1.0	298.15	764.3	75	-	-	-	-	-	-	-	-	(3.8)	-
407	363	O9106a	CCCCOCCC(C)C	1.0	298.15	767.6	75	-	-	-	-	-	-	-	-	(3.8)	-
408	364	O9107a	CCCCCOCC(C)C	1.0	298.15	765.6	75	-	-	-	-	-	-	-	-	(3.8)	-
409	365	O9108a	CCCCCCC(C)OC	1.0	298.15	802.09	75	-	-	-	-	-	-	-	-	(3.8)	-
410	366	O9109a	CCCCCOC(C)CC	1.0	298.15	768.7	75	-	-	-	-	-	-	-	-	(3.8)	-
411	367	O9110a	CCCCCCOC(C)C	1.0	298.15	767.8	75	-	-	-	-	-	-	-	-	(3.8)	-
412	368	O9111a	CCCCCCCCOC	1.0	298.15	783.0	75	-	-	-	-	-	-	591.5	81	(3.8)	-
413	369	O9112a	CCCCCCCOCC	1.0	298.15	775.3	75	-	-	-	-	-	-	-	-	(3.8)	-
414	370	O9113a	CCCCCCOCCC	1.0	298.15	773.0	75	-	-	-	-	-	-	-	-	(3.8)	-
415	371	O9114a	CCCCCOCCCC	1.0	298.15	772.7	75	-	-	-	-	-	-	-	-	(3.8)	-
416	372	O9201a	CC(C)COCOCC(C)C	1.0	298.15	820.27	75	-	-	-	-	438.6	81	-	-	(4.1)	-
417	373	O9202a	CCOCCCCCCOC	1.0	298.15	844.0	75	-	-	-	-	-	-	-	-	(4.1)	-
418	374	O9203a	CCOCCCCCOCC	1.0	298.15	838.6	75	-	-	-	-	-	-	-	-	(4.1)	-
419	375	O9204a	CCCCOCCCCOC	1.0	298.15	840.6	75	-	-	-	-	-	-	-	-	(4.1)	-
420	376	O9205a	CCCCOCOCCCC	1.0	298.15	831.53	75	-	-	214.2	87	452.4	81	-	-	(4.1)	-
421	377	O9206a	CCCCOCCOCCC	1.0	298.0	-	-	54.7	84	-	-	-	-	-	-	(4.1)	-
422	378	O0101a	CCOC(C)(C)CC(C)(C)C	1.0	298.0	-	-	45.3	84	-	-	-	-	-	-	(2.8)	-
423	379	O0102a	CCCCCCOC(C)(C)C	1.0	298.0	-	-	53.2	84	-	-	-	-	-	-	(2.8)	-
424	380	O0103a	CCC(CC)OC(CC)CC	1.0	293.15	784.8	75	-	-	-	-	-	-	-	-	(2.8)	-
425	381	O0104a	CC(C)CCOCCC(C)C	1.0	298.15	771.5	75	51.4	84	-	-	445.6	81	610.4	81	2.8	87
426	382	O0105a	CCCC(C)OC(C)CCC	1.0	293.15	777.8	75	-	-	-	-	-	-	-	-	(2.8)	-
427	383	O0106a	CCCCCOCCC(C)C	1.0	298.15	775.1	75	-	-	-	-	-	-	-	-	(2.8)	-
428	384	O0107a	CCCCCCOCC(C)C	1.0	298.15	771.2	75	-	-	-	-	-	-	-	-	(2.8)	-
429	385	O0108a	CCCCCCC(C)OCC	1.0	298.15	782.14	75	-	-	-	-	-	-	-	-	(2.8)	-

Table S.2 – Reference experimental data (continued)

n_{sim}	n_{iso}	Code	Smiles	P [bar]	T [K]	ρ_{liq} [kg·m^{-3}]	Src	ΔH_{vap} [kJ·mol^{-1}]	Src	T_m [K]	Src	T_b [K]	Src	T_c [K]	Src	ϵ	Src
430	386	O0109a	CCCCOC(C)CCCC	1.0	293.15	787.4	75	-	-	-	-	-	-	-	-	(2.8)	-
431	387	O0110a	CCCCCCCOC(C)CC	1.0	298.15	775.8	75	-	-	-	-	-	-	-	-	(2.8)	-
432	388	O0111a	CCCCCCCCOC(C)C	1.0	298.15	773.6	75	-	-	-	-	-	-	-	-	(2.8)	-
433	389	O0112a	CCCCCCCCCCOC	1.0	298.15	788.6	75	-	-	-	-	-	-	-	-	(2.8)	-
434	390	O0113a	CCCCCCCCCOCC	1.0	298.15	782.3	75	-	-	-	-	-	-	-	-	(2.8)	-
435	391	O0114a	CCCCCCCCOCCC	1.0	298.15	779.8	75	-	-	-	-	-	-	-	-	(2.8)	-
436	392	O0115a	CCCCCCCOCCCC	1.0	298.15	778.8	75	53.2	84	-	-	-	-	-	-	(2.8)	-
437	393	O0116a	CCCCCOCCCCC	1.0	298.15	779.2	75	-	-	203.9	87	460.1	87	-	-	2.8	87
438	394	O0201a	CC(C)COC(C)OCC(C)C	1.0	298.15	816.8	75	-	-	-	-	444.4	81	-	-	(4.1)	-
439	395	O0202a	CCCCOC(C)OCCCC	1.0	298.15	829.03	75	57.8	84	-	-	415.4	81	-	-	(4.1)	-
440	396	O0203a	CCCCOCCCCCOC	1.0	298.15	840.9	75	-	-	-	-	-	-	-	-	(4.1)	-
441	397	O0204a	CCCOCCCCOCCC	1.0	293.15	840.9	75	-	-	-	-	-	-	-	-	(4.1)	-
442	398	O0205a	CCCCOCCOCCCC	1.0	298.15	833.7	75	58.8	84	204.1	87	476.4	81	715.5	81	(4.1)	-
443	399	O0301a	CCCOCCOCCOCCC	1.0	288.15	886.9	75	-	-	-	-	-	-	-	-	(6.0)	-
444	400	A1101a	C=O	1.36	250.68	805.17	81	-	-	181.2	87	254.1	81	415.2	81	(2.0)	-
445	400	A1101b	C=O	1.0	293.15	814.0	76	-	-	181.2	87	254.1	81	415.2	81	(2.0)	-
446	401	A2101a	CC=O	1.0	298.15	772.0	76	26.9	84	149.8	87	293.6	81	466.0	81	21.0	87
447	402	A3101a	CCC=O	1.0	298.15	791.22	76	29.6	84	193.2	87	321.1	81	504.4	81	18.5	87
448	403	A4101a	CC(C)C=O	1.0	298.15	796.62	76	32.3	84	201.1	87	337.2	81	540.4	81	(13.4)	-
449	404	A4102a	CCCC=O	1.0	298.15	796.6	76	33.7	84	176.3	87	347.9	81	537.2	81	13.4	87
450	405	A5101a	CC(C)(C)C=O	0.14	298.15	783.1	81	-	-	274.1	87	347.1	81	570.0	81	9.1	87
451	406	A5102a	CCC(C)C=O	0.06	298.15	804.11	81	-	-	-	-	365.1	81	570.0	81	(9.5)	-
452	407	A5103a	CC(C)CC=O	1.0	298.15	794.21	76	-	-	222.2	87	365.8	81	570.0	81	(9.5)	-
453	408	A5104a	CCCCC=O	1.0	298.15	806.23	76	38.1	84	191.7	87	376.1	81	566.1	81	10.0	87
454	409	A6101a	CCC(C)(C)C=O	0.03	298.15	801.03	81	-	-	-	-	377.1	81	596.5	81	(13.5)	-
455	410	A6102a	CC(C)C(C)C=O	1.0	298.15	809.7	76	-	-	-	-	386.1	81	596.5	81	(13.5)	-
456	411	A6103a	CCC(C=O)CC	0.02	298.15	814.07	81	-	-	-	-	389.9	81	596.5	81	(13.5)	-
457	412	A6104a	CCCC(C)C=O	0.02	298.15	808.05	81	-	-	-	-	390.1	81	596.5	81	(13.5)	-
458	413	A6105a	CCC(C)CC=O	0.01	299.47	806.65	81	-	-	-	-	395.1	81	596.5	81	(13.5)	-
459	414	A6106a	CCCCCC=O	1.01	298.15	833.12	78	42.3	84	214.9	87	401.4	81	591.0	81	(13.5)	-
460	415	A6201a	O=CCCCCC=O	1.0	292.15	1003.0	87	-	-	265.1	87	435.2	81	-	-	(12.0)	-
461	416	A7101a	CCC(C=O)C(C)C	1.0	298.15	829.5	76	-	-	-	-	-	-	-	-	(9.1)	-
462	417	A7102a	CC(C)C(C)CC=O	1.0	298.15	823.1	76	-	-	-	-	-	-	-	-	(9.1)	-
463	417	A7102b	CC(C)C(C)CC=O	1.0	334.0	-	-	42.4	84	-	-	-	-	-	-	(9.1)	-
464	418	A7103a	CCCC(C)CC=O	1.0	298.15	814.33	76	-	-	-	-	416.1	81	620.4	81	(9.1)	-
465	418	A7103b	CCCC(C)CC=O	1.0	329.0	-	-	42.8	84	-	-	416.1	81	620.4	81	(9.1)	-
466	419	A7104a	CCC(C)CCC=O	1.0	298.15	824.0	76	-	-	-	-	-	-	620.4	81	(9.1)	-
467	420	A7105a	CC(C)CCCC=O	1.0	293.15	820.6	76	-	-	-	-	-	-	620.4	81	(9.1)	-
468	421	A7106a	CCCCCCC=O	1.0	298.15	813.29	76	48.0	84	229.2	87	425.9	81	616.8	81	9.1	87
469	422	A8101a	CCC(C=O)CC(C)C	1.0	293.15	847.6	76	-	-	-	-	-	-	-	-	(13.5)	-
470	423	A8102a	CCC(C)CC(C)C=O	1.0	293.15	847.6	76	-	-	-	-	-	-	-	-	(13.5)	-
471	424	A8103a	CCCCC(C=O)CC	1.0	298.15	815.16	76	-	-	-	-	433.8	81	642.4	81	(13.5)	-
472	425	A8104a	CCCCCCCC=O	1.01	298.15	821.07	78	51.0	84	-	-	447.1	81	638.9	81	(13.5)	-
473	426	A9101a	CCC(C)(C=O)CC(C)C	1.0	298.15	827.0	76	-	-	-	-	-	-	-	-	(13.5)	-
474	427	A9102a	CC(C=O)CC(C)C(C)C	1.0	291.15	884.3	76	-	-	-	-	-	-	-	-	(13.5)	-
475	428	A9103a	CCC(C)CC(C=O)CC	1.0	293.15	848.3	76	-	-	-	-	-	-	-	-	(13.5)	-
476	429	A9104a	CCCC(C=O)CC(C)C	1.0	293.15	842.3	76	-	-	-	-	-	-	-	-	(13.5)	-
477	430	A9105a	CCCCCCCCC=O	1.01	298.15	831.04	78	55.3	84	-	-	468.1	81	662.7	81	(13.5)	-

Table S.2 – Reference experimental data (continued)

n_{sim}	n_{iso}	Code	Smiles	P [bar]	T [K]	ρ_{liq} [kg·m^{-3}]	Src	ΔH_{vap} [kJ·mol^{-1}]	Src	T_m [K]	Src	T_b [K]	Src	T$_c$ [K]	Src	ϵ	Src
478	431	A0101a	CCCC(C=O)CC(C)CC	1.0	293.15	843.0	76	-	-	-	-	-	-	-	-	(13.5)	-
479	432	A0102a	CC(C)CCCC(C)CC=O	1.0	298.15	813.4	76	-	-	-	-	403.1	81	656.6	81	(13.5)	-
480	433	A0103a	CCCCCCCCCC=O	1.0	298.15	824.9	76	59.5	84	-	-	488.1	81	674.2	81	(13.5)	-
481	434	K3101a	CC(C)=O	1.0	298.15	784.37	76	31.3	84	178.2	87	329.4	81	508.2	81	21.0	87
482	435	K4101a	CCC(C)=O	1.0	298.15	799.91	76	34.5	84	186.5	87	352.8	81	535.5	81	18.6	87
483	436	K5101a	CC(=O)C(C)C	1.0	298.15	809.36	76	36.8	84	180.0	87	367.6	81	553.4	81	10.4	87
484	437	K5102a	CCC(=O)CC	1.0	298.15	809.6	76	38.5	84	234.2	87	375.1	81	560.9	81	17.0	87
485	438	K5103a	CCCC(C)=O	1.0	298.15	801.76	76	38.3	84	196.3	87	375.5	81	561.1	81	15.4	87
486	439	K6101a	CC(=O)C(C)(C)C	1.0	298.15	804.3	76	38.3	84	221.8	87	379.2	87	-	-	12.7	87
487	440	K6102a	CCC(=O)C(C)C	1.0	298.15	806.6	76	39.8	84	-	-	386.6	81	587.5	81	(13.5)	-
488	441	K6103a	CCC(C)C(C)=O	1.0	298.15	808.3	76	39.8	84	-	-	390.6	81	587.5	81	(13.5)	-
489	442	K6104a	CC(=O)CC(C)C	1.0	298.15	796.3	76	41.0	84	188.2	87	389.6	81	574.6	81	13.1	87
490	443	K6105a	CCCC(=O)CC	1.0	298.15	811.1	76	40.6	84	217.8	87	396.6	81	582.8	81	(13.5)	-
491	444	K6106a	CCCCC(C)=O	1.0	298.15	807.14	76	42.2	84	217.7	87	400.9	81	587.6	81	14.6	87
492	445	K6201a	CCC(=O)CC(C)=O	1.0	293.15	959.0	87	-	-	-	-	433.1	81	629.0	81	(15.3)	-
493	446	K7101a	CCC(C)(C)C(C)=O	1.0	294.15	823.0	76	-	-	-	-	403.8	81	611.4	81	(12.7)	-
494	447	K7102a	CCC(=O)C(C)(C)C	1.0	298.15	808.4	76	42.3	84	228.2	87	398.1	81	611.4	81	(12.7)	-
495	448	K7103a	CC(=O)CC(C)(C)C	1.0	298.15	801.2	76	-	-	209.2	87	398.1	81	611.4	81	(12.7)	-
496	449	K7104a	CC(C)C(=O)C(C)C	1.0	298.15	799.73	76	41.5	84	204.8	87	397.6	81	611.4	81	(12.7)	-
497	450	K7105a	CC(=O)C(C)C(C)C	1.0	293.15	827.3	76	-	-	-	-	405.1	81	611.4	81	(12.7)	-
498	451	K7106a	CCC(=O)C(C)CC	1.0	298.15	824.0	76	-	-	-	-	409.1	81	611.4	81	(12.7)	-
499	452	K7107a	CCCC(=O)C(C)C	0.01	302.47	809.45	81	-	-	-	-	406.1	81	611.4	81	(12.7)	-
500	453	K7108a	CCC(CC)C(C)=O	1.0	295.15	815.3	76	-	-	-	-	411.1	81	611.4	81	(12.7)	-
501	454	K7109a	CCCC(C)C(C)=O	1.0	298.15	828.0	76	-	-	-	-	413.1	81	611.4	81	(12.7)	-
502	455	K7110a	CCC(=O)CC(C)C	1.0	293.15	812.0	76	-	-	-	-	409.1	81	611.4	81	(12.7)	-
503	456	K7111a	CCC(C)CC(C)=O	1.0	298.15	808.5	76	-	-	-	-	412.1	81	611.4	81	(12.7)	-
504	457	K7112a	CC(=O)CCC(C)C	1.0	293.15	811.6	76	-	-	-	-	417.9	81	611.4	81	13.5	87
505	458	K7113a	CCCC(=O)CCC	1.0	298.15	811.6	76	-	-	241.1	87	417.1	81	602.0	81	12.6	87
506	459	K7114a	CCCCC(=O)CC	1.0	298.15	814.64	76	-	-	235.9	87	420.6	81	606.6	81	12.7	87
507	460	K7115a	CCCCCC(C)=O	1.0	298.15	811.64	76	46.1	84	238.4	87	424.1	81	611.4	81	11.9	87
508	461	K7201a	CCC(C(C)=O)C(C)=O	1.0	292.15	953.1	87	-	-	-	-	451.6	81	645.0	81	(15.3)	-
509	462	K7202a	CCC(=O)CC(=O)CC	1.0	293.15	945.0	87	-	-	-	-	449.1	81	645.0	81	(15.3)	-
510	463	K8101a	CC(=O)C(C)(C)C(C)C	1.0	293.15	839.5	76	-	-	-	-	-	-	-	-	(10.0)	-
511	464	K8102a	CC(C)C(=O)C(C)(C)C	1.0	298.15	802.3	76	43.3	84	-	-	408.2	81	633.4	81	(10.0)	-
512	465	K8103a	CC(=O)C(C)C(C)(C)C	1.0	293.15	826.0	76	-	-	-	-	-	-	-	-	(10.0)	-
513	466	K8104a	CCC(C)(CC)C(C)=O	1.0	293.15	838.9	76	-	-	-	-	426.6	81	633.4	81	(10.0)	-
514	467	K8105a	CCC(=O)C(C)(C)CC	1.0	293.15	829.8	76	-	-	-	-	421.1	81	633.4	81	(10.0)	-
515	468	K8106a	CCCC(C)(C)C(C)=O	1.0	293.15	825.7	76	-	-	-	-	420.1	81	633.4	81	(10.0)	-
516	469	K8107a	CCCC(=O)C(C)(C)C	1.0	298.15	810.5	76	-	-	-	-	419.1	81	633.4	81	(10.0)	-
517	470	K8108a	CCC(C)(C)CC(C)=O	1.0	293.15	829.0	76	-	-	-	-	-	-	-	-	(10.0)	-
518	471	K8109a	CCC(=O)CC(C)(C)C	1.0	293.15	812.0	76	-	-	-	-	-	-	-	-	(10.0)	-
519	472	K8110a	CC(C)CC(=O)C(C)C	1.0	293.15	812.1	76	-	-	-	-	420.6	81	633.4	81	(10.0)	-
520	473	K8111a	CCC(C)C(C)C(C)=O	1.0	295.15	829.5	76	-	-	-	-	431.1	81	633.4	81	(10.0)	-
521	474	K8112a	CCCC(=O)C(C)CC	1.0	298.15	817.0	76	-	-	-	-	426.1	81	633.4	81	(10.0)	-
522	475	K8113a	CCCC(C)C(=O)CC	1.0	298.15	820.0	76	-	-	-	-	-	-	633.4	81	(10.0)	-
523	476	K8114a	CCCCC(=O)C(C)C	1.0	293.15	817.5	76	-	-	-	-	431.1	81	633.4	81	(10.0)	-
524	477	K8115a	CCC(=O)CC(C)CC	1.0	297.15	829.0	76	-	-	-	-	435.1	81	633.4	81	(10.0)	-
525	478	K8116a	CCCC(=O)CC(C)C	1.0	295.15	813.0	87	-	-	-	-	427.1	81	633.4	81	(10.0)	-

Table S.2 – Reference experimental data (continued)

n_{sim}	n_{iso}	Code	Smiles	P [bar]	T [K]	ρ_{liq} [kg·m^{-3}]	Src	ΔH_{vap} [kJ·mol^{-1}]	Src	T_m [K]	Src	T_b [K]	Src	T_c [K]	Src	ϵ	Src
526	479	K8117a	CCC(=O)CCC(C)C	1.0	293.15	830.4	87	-	-	-	-	437.1	81	633.4	81	(10.0)	-
527	480	K8118a	CCC(C)CCC(C)=O	1.0	298.15	811.0	76	-	-	-	-	437.0	81	633.4	81	(10.0)	-
528	481	K8119a	CC(=O)CCCC(C)C	1.0	298.15	810.0	76	-	-	-	-	440.1	81	633.4	81	(10.0)	-
529	482	K8120a	CCCCC(=O)CCC	1.0	298.15	814.72	76	-	-	-	-	436.1	81	633.4	81	(10.0)	-
530	483	K8121a	CCCCCC(=O)CC	1.0	298.15	822.0	87	-	-	-	-	440.6	81	633.4	81	10.5	87
531	484	K8122a	CCCCCCC(C)=O	1.0	298.15	815.25	76	51.8	84	252.8	87	445.8	81	632.7	81	9.5	87
532	485	K9101a	CC(C)(C)C(=O)C(C)(C)C	1.0	298.15	820.2	76	45.4	84	247.9	87	425.1	81	653.7	81	10.0	87
533	486	K9102a	CC(C)CC(=O)C(C)(C)C	1.0	296.15	812.0	76	-	-	-	-	-	-	-	-	(9.9)	-
534	487	K9103a	CCCCC(=O)C(C)(C)C	1.0	293.15	816.8	76	-	-	-	-	-	-	-	-	(9.9)	-
535	488	K9104a	CCCC(=O)CC(C)(C)C	1.0	298.15	809.0	76	-	-	-	-	-	-	-	-	(9.9)	-
536	489	K9105a	CCC(C)C(=O)C(C)CC	1.0	287.15	826.0	76	-	-	-	-	435.1	81	653.7	81	(9.9)	-
537	490	K9106a	CC(C)CCC(=O)C(C)C	1.0	298.15	813.5	76	-	-	-	-	445.0	81	653.7	81	(9.9)	-
538	491	K9107a	CC(C)CC(=O)CC(C)C	1.0	298.15	802.43	76	50.9	84	227.2	87	441.4	81	653.7	81	9.9	87
539	492	K9108a	CC(=O)CCC(C)C(C)C	1.0	293.15	833.5	76	-	-	-	-	-	-	-	-	(9.9)	-
540	493	K9109a	CC(=O)CC(C)CC(C)C	1.0	298.15	817.0	76	-	-	-	-	-	-	-	-	(9.9)	-
541	494	K9110a	CCCCC(=O)C(C)CC	1.0	287.15	829.0	76	-	-	-	-	447.1	81	653.7	81	(9.9)	-
542	495	K9111a	CCCCC(C)C(=O)CC	1.0	298.15	820.0	76	-	-	-	-	456.6	81	653.7	81	(9.9)	-
543	496	K9112a	CCCCCC(=O)C(C)C	1.0	293.15	821.2	76	-	-	-	-	456.0	81	653.7	81	(9.9)	-
544	497	K9113a	CCCCC(CC)C(C)=O	1.0	293.15	824.6	76	-	-	-	-	-	-	-	-	(9.9)	-
545	498	K9114a	CCCCCC(C)C(C)=O	1.0	300.15	832.0	76	-	-	-	-	456.6	81	653.7	81	(9.9)	-
546	499	K9115a	CCCCC(=O)CC(C)C	1.0	298.15	815.0	76	-	-	-	-	-	-	653.7	81	(9.9)	-
547	500	K9116a	CCCC(=O)CCC(C)C	1.0	293.15	823.9	76	-	-	-	-	451.1	81	653.7	81	(9.9)	-
548	501	K9117a	CCC(C)CCCC(C)=O	1.0	298.15	822.0	76	-	-	-	-	-	-	653.7	81	(9.9)	-
549	502	K9118a	CCCCC(=O)CCCC	1.0	298.15	817.77	76	53.3	84	269.3	87	461.6	81	640.0	81	10.6	87
550	503	K9119a	CCCCCC(=O)CCC	1.0	298.15	819.68	76	-	-	-	-	460.6	81	653.7	81	(9.9)	-
551	504	K9120a	CCCCCCC(=O)CC	1.0	298.15	820.44	76	55.6	84	265.1	87	463.1	81	653.7	81	(9.9)	-
552	505	K9121a	CCCCCCCC(C)=O	1.0	298.15	817.78	76	-	-	265.8	87	467.1	81	652.5	81	9.1	87
553	506	K0101a	CC(C)(C)CC(=O)C(C)(C)C	1.0	298.15	816.9	76	48.8	84	-	-	-	-	-	-	(8.3)	-
554	507	K0102a	CCC(CC)C(=O)C(C)(C)C	1.0	298.15	825.2	76	-	-	-	-	-	-	-	-	(8.3)	-
555	508	K0103a	CC(=O)CC(C)CC(C)(C)C	1.0	288.65	825.2	76	-	-	-	-	-	-	-	-	(8.3)	-
556	509	K0104a	CCCCCC(=O)C(C)(C)C	1.0	293.15	832.0	76	-	-	-	-	-	-	-	-	(8.3)	-
557	510	K0105a	CCCCC(=O)CC(C)(C)C	1.0	293.15	814.3	76	-	-	-	-	-	-	-	-	(8.3)	-
558	511	K0106a	CCCCCCC(=O)C(C)C	1.0	293.15	822.6	76	-	-	-	-	473.0	81	672.7	81	(8.3)	-
559	512	K0107a	CCCCCC(=O)CC(C)C	1.0	298.15	818.0	76	-	-	-	-	-	-	-	-	(8.3)	-
560	513	K0108a	CCCCC(=O)CCC(C)C	1.0	293.15	821.3	76	-	-	-	-	476.6	81	672.7	81	(8.3)	-
561	514	K0109a	CCCC(C)CCCC(C)=O	1.0	293.15	838.4	76	-	-	-	-	-	-	-	-	(8.3)	-
562	515	K0110a	CCCCCC(=O)CCCC	1.0	298.15	820.55	76	-	-	-	-	477.1	81	672.7	81	(8.3)	-
563	516	K0111a	CCCCCCC(=O)CCC	1.0	293.65	822.0	76	-	-	264.1	87	479.6	81	672.7	81	(8.3)	-
564	517	K0112a	CCCCCCCC(=O)CC	1.0	298.15	821.92	76	-	-	275.1	87	476.1	81	672.7	81	(8.3)	-
565	518	K0113a	CCCCCCCCC(C)=O	1.0	298.15	820.1	76	60.9	84	287.1	87	483.4	81	672.7	81	8.3	87
566	519	E2201a	COC=O	1.0	298.15	966.82	75	28.4	84	173.4	87	304.9	81	487.2	81	9.2	87
567	520	E3201a	CCOC=O	1.0	298.15	915.87	75	31.5	84	193.6	87	327.5	81	508.4	81	8.6	87
568	521	E3202a	COC(C)=O	1.01	298.15	927.5	78	32.3	84	174.9	87	330.1	81	506.6	81	7.1	87
569	522	E4201a	CC(C)OC=O	0.17	298.15	870.23	81	-	-	-	-	341.2	81	514.9	81	(6.9)	-
570	523	E4202a	CCCOC=O	1.0	298.15	899.89	75	36.6	84	180.2	87	354.0	81	538.0	81	6.9	87
571	524	E4203a	CCC(=O)OC	1.0	298.15	909.1	78	35.7	84	185.7	87	352.6	81	530.6	81	6.2	87
572	525	E4204a	CCOC(C)=O	1.0	298.15	894.27	75	35.1	84	189.3	87	350.2	81	523.3	81	6.1	87
573	526	E4401a	O=COCCOC=O	1.0	273.15	1193.0	87	-	-	-	-	447.1	81	-	-	(7.0)	-

Table S.2 – Reference experimental data (continued)

n_{sim}	n_{iso}	Code	Smiles	P [bar]	T [K]	ρ_{liq} [kg·m^{-3}]	Src	ΔH_{vap} [kJ·mol^{-1}]	Src	T_m [K]	Src	T_b [K]	Src	T$_c$ [K]	Src	ϵ	Src
574	527	E5201a	CC(C)(C)OC=O	0.09	298.15	871.91	81	-	-	-	-	355.9	81	541.0	81	(6.2)	-
575	528	E5202a	CC(C)COC=O	1.0	298.15	875.7	75	-	-	177.7	87	371.2	81	551.4	81	6.4	87
576	529	E5203a	CCC(C)OC=O	0.06	298.15	878.57	81	-	-	-	-	363.6	81	541.0	81	(6.2)	-
577	530	E5204a	CCCCOC=O	1.0	298.15	887.64	75	40.5	84	183.2	87	379.2	81	541.0	81	6.1	87
578	531	E5205a	COC(=O)C(C)C	1.0	298.15	883.3	75	37.3	84	188.6	87	365.6	81	540.7	81	(5.6)	-
579	532	E5206a	CC(=O)OC(C)C	1.0	298.15	869.0	75	37.0	84	199.8	87	361.6	81	532.0	81	(5.6)	-
580	533	E5207a	CCCC(=O)OC	1.01	298.15	892.52	78	39.8	84	187.3	87	375.9	81	554.5	81	5.5	87
581	534	E5208a	CCOC(=O)CC	1.0	298.15	884.04	75	39.3	84	199.6	87	372.2	81	546.0	81	5.8	87
582	535	E5209a	CCCOC(C)=O	1.01	298.15	882.8	78	39.1	84	180.2	87	374.6	81	549.7	81	5.6	87
583	536	E5401a	COC(=O)CC(=O)OC	1.0	298.15	1146.71	75	57.5	84	211.2	87	454.6	81	-	-	9.8	87
584	537	E5402a	CC(=O)OCOC(C)=O	1.0	298.15	1135.5	75	56.4	84	-	-	437.6	81	-	-	(9.8)	-
585	538	E6201a	CCC(C)(C)OC=O	0.02	298.15	883.69	81	-	-	-	-	385.6	81	564.6	81	(5.6)	-
586	539	E6202a	CC(C)CCOC=O	1.0	298.15	877.0	75	-	-	179.7	87	397.1	87	564.6	81	5.4	87
587	540	E6203a	CCCCCOC=O	1.0	298.15	880.37	75	45.2	84	200.2	87	406.6	81	576.0	81	5.7	87
588	541	E6204a	COC(=O)C(C)(C)C	1.0	293.15	850.0	75	38.8	84	-	-	374.2	81	-	-	(5.2)	-
589	542	E6205a	CC(=O)OC(C)(C)C	1.0	298.15	861.59	75	38.0	84	-	-	369.1	81	564.6	81	5.7	87
590	543	E6206a	CCC(C)C(=O)OC	1.0	293.15	884.7	75	-	-	-	-	-	-	-	-	(5.2)	-
591	544	E6207a	CCOC(=O)C(C)C	1.0	298.15	864.04	75	39.8	84	175.3	87	383.0	81	553.1	81	(5.2)	-
592	545	E6208a	COC(=O)CC(C)C	1.0	298.15	875.87	75	-	-	-	-	389.6	81	564.6	81	(5.2)	-
593	546	E6209a	CCC(=O)OC(C)C	1.0	298.15	860.1	75	-	-	-	-	383.1	81	553.0	81	(5.2)	-
594	547	E6210a	CC(=O)OCC(C)C	1.0	298.15	866.29	75	39.52	81	176.1	87	389.8	81	560.8	81	5.1	87
595	548	E6211a	CCC(C)OC(C)=O	1.0	298.15	866.05	75	-	-	174.2	87	385.1	81	564.6	81	5.1	87
596	549	E6212a	CCCCC(=O)OC	1.0	298.15	885.17	75	43.7	84	-	-	400.6	81	564.6	81	5.0	87
597	550	E6213a	CCCC(=O)OCC	1.0	298.15	873.78	75	42.0	84	176.2	87	394.6	81	571.0	81	5.2	87
598	551	E6214a	CCCOC(=O)CC	1.0	298.15	876.35	75	43.2	84	197.2	87	395.6	81	568.6	81	5.2	87
599	552	E6215a	CCCCOC(C)=O	1.0	298.15	876.36	75	42.7	84	196.2	87	399.1	81	575.4	81	5.1	87
600	553	E6401a	COC(=O)C(C)C(=O)OC	1.0	298.15	1093.63	75	-	-	-	-	447.1	81	-	-	(7.7)	-
601	553	E6401b	COC(=O)C(C)C(=O)OC	1.0	293.0	-	-	57.8	84	-	-	447.1	81	-	-	(7.7)	-
602	554	E6402a	CC(=O)OC(C)OC(C)=O	1.0	298.15	1070.0	87	59.0	84	292.1	87	442.1	81	635.0	81	(7.7)	-
603	555	E6403a	COC(=O)CCC(=O)OC	1.0	298.15	1114.0	75	60.9	84	291.8	87	469.6	81	657.0	81	7.2	87
604	556	E6404a	CC(=O)OCCOC(C)=O	1.0	298.15	1098.7	75	61.0	84	242.2	87	463.6	81	653.0	81	7.7	87
605	557	E7201a	CCCCCCOC=O	1.0	298.15	874.61	75	50.0	84	210.6	87	428.6	81	586.3	81	(6.9)	-
606	558	E7202a	CCOC(=O)C(C)(C)C	1.0	298.15	849.5	75	41.3	84	183.7	87	391.6	81	-	-	(4.7)	-
607	559	E7203a	COC(=O)CC(C)(C)C	1.0	293.15	870.0	75	43.9	84	-	-	-	-	-	-	(4.7)	-
608	560	E7204a	CC(=O)OCC(C)(C)C	1.0	298.15	853.9	75	-	-	-	-	-	-	586.3	81	(4.7)	-
609	561	E7205a	CCC(C)(C)OC(C)=O	1.0	297.85	872.5	75	40.3	84	-	-	-	-	-	-	(4.7)	-
610	562	E7206a	CCC(=O)OC(C)(C)C	1.0	293.15	864.7	75	-	-	-	-	414.6	81	-	-	(4.7)	-
611	563	E7207a	CC(C)OC(=O)C(C)C	1.0	294.5	846.74	75	-	-	-	-	396.1	87	-	-	(4.7)	-
612	564	E7208a	CC(=O)OC(C)C(C)C	1.0	298.15	866.0	75	-	-	-	-	401.6	81	586.3	81	(4.7)	-
613	565	E7209a	CCC(CC)C(=O)OC	1.0	293.15	879.7	75	-	-	-	-	409.1	81	586.3	81	(4.7)	-
614	566	E7210a	CCCC(C)C(=O)OC	1.0	293.15	876.5	75	-	-	-	-	-	-	-	-	(4.7)	-
615	567	E7211a	CCOC(=O)C(C)CC	1.0	293.15	867.8	75	44.7	84	-	-	-	-	-	-	(4.7)	-
616	568	E7212a	CCCOC(=O)C(C)C	1.0	298.15	859.46	75	-	-	-	-	407.1	87	-	-	(4.7)	-
617	569	E7213a	CCOC(=O)CC(C)C	1.0	298.15	861.23	75	-	-	173.8	87	408.1	87	-	-	4.7	87
618	570	E7214a	CCC(=O)OCC(C)C	1.0	298.15	867.52	75	-	-	201.8	87	409.1	87	592.0	81	(4.7)	-
619	571	E7215a	CCC(=O)OC(C)CC	1.0	298.15	861.25	75	-	-	-	-	406.1	87	-	-	(4.7)	-
620	572	E7216a	CCCC(=O)OC(C)C	1.0	298.15	853.92	75	-	-	-	-	402.1	87	-	-	(4.7)	-
621	573	E7217a	CCC(C)COC(C)=O	1.0	293.15	867.8	75	-	-	-	-	413.1	81	586.3	81	(4.7)	-

Table S.2 – Reference experimental data (continued)

n_{sim}	n_{iso}	Code	Smiles	P [bar]	T [K]	ρ_{liq} [kg·m^{-3}]	Src	ΔH_{vap} [kJ·mol^{-1}]	Src	T_m [K]	Src	T_b [K]	Src	T_c [K]	Src	ϵ	Src
622	574	E7218a	CCC(CC)OC(C)=O	0.01	303.27	861.47	81	-	-	-	-	405.1	81	586.3	81	(4.7)	-
623	575	E7219a	CC(=O)OCCC(C)C	1.0	298.75	864.8	75	46.4	84	194.7	87	414.8	87	586.1	81	4.7	87
624	576	E7220a	CCCC(C)OC(C)=O	1.0	298.15	863.07	75	-	-	-	-	406.1	81	586.3	81	(4.7)	-
625	577	E7221a	CCCCCC(=O)OC	1.0	298.15	880.4	75	47.7	84	203.2	87	422.6	81	586.3	81	4.6	87
626	578	E7222a	CCCCC(=O)OCC	1.01	298.15	869.42	78	47.0	84	181.9	87	419.2	81	586.3	81	4.7	87
627	579	E7223a	CCCOC(=O)CCC	1.0	298.15	868.23	75	-	-	177.9	87	416.4	81	593.7	81	4.3	87
628	580	E7224a	CCCCOC(=O)CC	1.0	298.15	871.54	75	48.5	84	183.7	87	419.8	81	594.6	81	4.8	87
629	581	E7225a	CCCCCOC(C)=O	1.0	298.15	872.23	75	48.6	84	202.2	87	422.1	81	599.9	81	4.8	87
630	582	E7401a	COC(=O)C(C)(C)C(=O)OC	1.0	293.0	-	-	55.6	84	-	-	-	-	-	-	(7.7)	-
631	583	E7402a	CCC(C(=O)OC)C(=O)OC	1.0	298.15	1061.39	75	-	-	-	-	-	-	-	-	(7.7)	-
632	584	E7403a	COC(=O)CC(C)C(=O)OC	1.0	298.15	1076.0	87	-	-	-	-	469.1	81	-	-	(7.7)	-
633	585	E7404a	CC(=O)OCC(C)OC(C)=O	1.0	293.15	1059.0	87	-	-	-	-	463.6	81	-	-	(7.7)	-
634	586	E7405a	CCOC(=O)CC(=O)OCC	1.0	298.15	1049.8	75	58.7	84	223.2	87	472.1	81	653.0	81	7.5	87
635	587	E7406a	CCOC(=O)CCC(=O)OC	1.0	293.15	1076.0	75	-	-	-	-	481.4	81	-	-	(7.7)	-
636	588	E7407a	COC(=O)CCCC(=O)OC	1.01	293.15	1087.67	78	65.7	84	230.7	87	487.1	81	-	-	7.9	87
637	589	E7408a	CC(=O)OCCCOC(C)=O	1.0	293.15	1054.7	75	-	-	-	-	482.6	81	-	-	(7.7)	-
638	590	E8201a	CCCCCCCCOC=O	1.0	302.25	869.0	75	53.8	84	-	-	451.2	81	606.5	81	(6.9)	-
639	591	E8202a	CC(C)OC(=O)C(C)(C)C	1.0	293.15	830.0	75	-	-	-	-	-	-	-	-	(4.5)	-
640	592	E8203a	COC(=O)C(C)C(C)(C)C	1.0	298.15	874.9	75	-	-	-	-	-	-	-	-	(4.5)	-
641	593	E8204a	CCOC(=O)C(C)(C)CC	1.0	277.15	883.0	75	-	-	-	-	-	-	-	-	(4.5)	-
642	594	E8205a	CCOC(=O)CC(C)(C)C	1.0	293.15	860.4	75	-	-	-	-	-	-	-	-	(4.5)	-
643	595	E8206a	CC(=O)OCCC(C)(C)C	1.0	293.15	867.9	75	-	-	-	-	-	-	-	-	(4.5)	-
644	596	E8207a	CCC(=O)OC(C)(C)CC	0.01	307.57	874.67	81	-	-	-	-	437.4	81	606.5	81	(4.5)	-
645	597	E8208a	CCCC(C)(C)OC(C)=O	0.01	320.07	862.59	81	-	-	-	-	426.1	81	606.5	81	(4.5)	-
646	598	E8209a	CCOC(=O)C(C)C(C)C	1.0	298.15	866.0	75	-	-	-	-	-	-	-	-	(4.5)	-
647	599	E8210a	CC(C)COC(=O)C(C)C	1.0	293.15	847.0	75	48.5	84	192.6	87	421.1	87	-	-	(4.5)	-
648	600	E8211a	CCC(C)OC(=O)C(C)C	1.0	289.15	870.0	75	-	-	-	-	-	-	606.5	81	(4.5)	-
649	601	E8212a	CC(C)CC(=O)OC(C)C	1.0	298.15	846.1	75	-	-	-	-	420.1	87	-	-	(4.5)	-
650	602	E8213a	CC(=O)OC(C)CC(C)C	1.0	298.15	880.5	87	-	-	-	-	420.6	87	-	-	(4.5)	-
651	603	E8214a	CCCC(CC)C(=O)OC	1.0	293.15	875.0	75	-	-	-	-	-	-	-	-	(4.5)	-
652	604	E8215a	CCOC(=O)C(CC)CC	1.0	293.15	863.3	75	-	-	-	-	-	-	-	-	(4.5)	-
653	605	E8216a	CCCC(C)C(=O)OCC	1.0	293.15	876.5	75	48.4	84	-	-	428.3	78	-	-	(4.5)	-
654	605	E8216b	CCCC(C)C(=O)OCC	1.0	298.15	-	-	48.4	78	-	-	428.3	78	-	-	(4.5)	-
655	606	E8217a	CCCOC(=O)C(C)CC	1.0	293.15	869.7	75	-	-	-	-	-	-	-	-	(4.5)	-
656	607	E8218a	CCCCOC(=O)C(C)C	1.0	298.15	857.37	75	-	-	-	-	-	-	-	-	(4.5)	-
657	608	E8219a	CCOC(=O)CC(C)CC	1.0	293.15	878.0	75	-	-	-	-	-	-	-	-	(4.5)	-
658	609	E8220a	CCCOC(=O)CC(C)C	1.0	298.15	857.48	75	-	-	-	-	429.1	81	-	-	(4.5)	-
659	610	E8221a	CCOC(=O)CCC(C)C	1.0	293.15	870.5	87	-	-	-	-	436.1	81	606.5	81	(4.5)	-
660	611	E8222a	CCCC(=O)OCC(C)C	1.0	298.15	860.6	75	-	-	-	-	430.1	87	611.0	81	(4.5)	-
661	612	E8223a	CCC(=O)OCCC(C)C	1.0	298.15	865.0	75	-	-	-	-	446.1	87	606.5	81	5.2	87
662	613	E8224a	CCCC(=O)OC(C)CC	1.0	298.15	863.24	75	-	-	-	-	-	-	-	-	(4.5)	-
663	614	E8225a	CCCC(C)OC(=O)CC	1.0	298.15	861.28	75	-	-	-	-	440.2	81	606.5	81	(4.5)	-
664	615	E8226a	CCCCC(=O)OC(C)C	1.0	293.15	857.9	87	-	-	-	-	436.4	81	606.5	81	(4.5)	-
665	616	E8227a	CCC(CC)COC(C)=O	1.0	293.15	879.0	87	-	-	-	-	435.6	81	606.5	81	(4.5)	-
666	617	E8228a	CCCC(C)COC(C)=O	1.0	298.15	869.1	75	-	-	-	-	436.1	87	606.5	81	(4.5)	-
667	618	E8229a	CCCCC(C)OC(C)=O	1.0	298.15	859.91	75	-	-	-	-	-	-	606.5	81	(4.5)	-
668	619	E8230a	CCCCCCC(=O)OC	1.01	298.15	875.88	78	51.6	84	217.4	87	447.1	81	606.5	81	4.4	87
669	620	E8231a	CCCCCC(=O)OCC	1.0	298.15	866.69	75	50.6	84	205.6	87	440.1	81	606.5	81	4.5	87

Table S.2 – Reference experimental data (continued)

n_{sim}	n_{iso}	Code	Smiles	P [bar]	T [K]	ρ_{liq} [kg·m^{-3}]	Src	ΔH_{vap} [kJ·mol^{-1}]	Src	T_m [K]	Src	T_b [K]	Src	T_c [K]	Src	ϵ	Src
670	621	E8232a	CCCCC(=O)OCCC	1.0	298.15	865.78	75	-	-	202.4	87	440.6	81	606.5	81	4.0	87
671	622	E8233a	CCCCOC(=O)CCC	1.01	298.15	869.14	78	-	-	181.7	87	438.1	81	606.5	81	4.4	87
672	623	E8234a	CCCCCOC(=O)CC	1.0	298.15	868.1	75	52.2	84	200.1	87	441.9	81	606.5	81	4.6	87
673	624	E8235a	CCCCCCOC(C)=O	1.0	298.15	868.6	75	51.9	84	212.2	87	444.6	81	606.5	81	4.4	87
674	625	E8401a	CCCC(C(=O)OC)C(=O)OC	1.0	298.15	1036.93	75	-	-	-	-	-	-	-	-	(6.5)	-
675	626	E8402a	CCOC(=O)C(C)C(=O)OCC	1.0	298.15	1017.38	75	-	-	-	-	474.1	81	-	-	(6.5)	-
676	627	E8403a	CCOC(=O)CCC(=O)OCC	1.0	298.15	1035.3	75	64.5	84	251.6	87	489.6	81	663.0	81	6.1	87
677	628	E8404a	COC(=O)CCCCC(=O)OC	1.0	298.15	1057.57	75	69.0	84	283.4	87	495.1	81	-	-	6.8	87
678	629	E8405a	CCC(=O)OCCOC(=O)CC	1.0	293.15	1042.0	75	67.6	84	-	-	484.1	81	-	-	(6.5)	-
679	630	E8406a	CC(=O)OCCCCOC(C)=O	1.0	293.15	1046.0	75	-	-	285.1	87	502.1	81	-	-	(6.5)	-
680	631	E9201a	CCCCCCCCOC=O	1.0	298.15	871.0	75	58.2	84	234.1	87	471.9	81	625.3	81	(6.9)	-
681	632	E9202a	COC(=O)C(C)CC(C)(C)C	1.0	298.0	-	-	48.4	84	-	-	-	-	-	-	(4.0)	-
682	633	E9203a	CCC(C)(C)OC(=O)C(C)C	1.0	298.0	-	-	47.8	84	-	-	-	-	-	-	(4.0)	-
683	634	E9204a	CCCCOC(=O)C(C)(C)C	1.0	298.0	-	-	50.4	84	-	-	-	-	-	-	(4.0)	-
684	635	E9205a	CCCC(=O)OC(C)(C)CC	1.0	298.0	-	-	50.3	84	-	-	460.3	81	625.3	81	(4.0)	-
685	636	E9206a	CCOC(=O)C(C)CC(C)C	1.0	301.15	855.0	75	-	-	-	-	-	-	-	-	(4.0)	-
686	637	E9207a	CC(C)CCOC(=O)C(C)C	1.0	293.15	862.7	87	51.7	84	-	-	442.1	87	-	-	(4.0)	-
687	638	E9208a	CCOC(=O)CC(C)C(C)C	1.0	298.15	869.0	75	-	-	-	-	-	-	-	-	(4.0)	-
688	639	E9209a	CC(C)COC(=O)CC(C)C	1.0	298.15	861.7	75	-	-	-	-	442.1	87	-	-	(4.0)	-
689	640	E9210a	CCC(C)OC(=O)CC(C)C	1.0	293.15	848.2	75	-	-	-	-	-	-	-	-	(4.0)	-
690	641	E9211a	CCCC(CCC)C(=O)OC	1.0	293.15	871.0	75	-	-	-	-	-	-	-	-	(4.0)	-
691	642	E9212a	CCCC(CC)C(=O)OCC	1.0	293.15	857.2	75	-	-	-	-	-	-	-	-	(4.0)	-
692	643	E9213a	CCCOC(=O)C(CC)CC	1.0	293.15	868.8	75	-	-	-	-	-	-	-	-	(4.0)	-
693	644	E9214a	CCCOC(=O)C(C)CCC	1.0	293.15	866.9	75	-	-	-	-	-	-	-	-	(4.0)	-
694	645	E9215a	CCCCOC(=O)C(C)CC	1.0	293.15	862.0	87	50.6	84	-	-	452.1	81	-	-	(4.0)	-
695	646	E9216a	CCCCCOC(=O)C(C)C	1.0	293.15	872.1	75	-	-	-	-	-	-	625.3	81	(4.0)	-
696	647	E9217a	CCCC(C)CC(=O)OCC	1.0	293.15	867.9	75	-	-	-	-	450.1	81	625.3	81	(4.0)	-
697	648	E9218a	CCCCOC(=O)CC(C)C	1.0	298.15	856.69	75	-	-	-	-	456.9	81	-	-	(4.0)	-
698	649	E9219a	CCOC(=O)CCC(C)CC	1.0	293.15	870.8	87	-	-	-	-	453.1	81	625.3	81	(4.0)	-
699	650	E9220a	CCCC(=O)OCCC(C)C	1.0	298.15	860.3	75	-	-	-	-	452.1	81	625.3	81	4.0	87
700	651	E9221a	CCCCC(=O)OCC(C)C	1.0	298.15	853.57	75	-	-	-	-	456.1	87	625.3	81	3.8	87
701	652	E9222a	CCCC(=O)OC(C)CCC	1.0	298.15	864.72	75	-	-	-	-	455.1	81	625.3	81	(4.0)	-
702	653	E9223a	CCCCC(=O)OC(C)CC	1.0	298.15	854.87	75	-	-	-	-	-	-	-	-	(4.0)	-
703	654	E9224a	CCCCCC(=O)OC(C)C	1.0	298.15	852.5	75	-	-	-	-	-	-	-	-	(4.0)	-
704	655	E9225a	CCCCCC(C)OC(C)=O	1.0	298.15	857.03	75	-	-	-	-	-	-	625.3	81	(4.0)	-
705	656	E9226a	CCCCCCCC(=O)OC	1.0	298.15	873.12	75	56.4	84	236.2	87	466.1	81	625.3	81	4.1	87
706	657	E9227a	CCCCCCC(=O)OCC	1.0	298.15	864.71	75	-	-	206.9	87	460.1	81	625.3	81	(4.0)	-
707	658	E9228a	CCCCCC(=O)OCCC	1.0	298.15	863.02	75	-	-	199.2	87	460.1	81	625.3	81	(4.0)	-
708	659	E9229a	CCCCOC(=O)CCCC	1.0	298.15	863.37	75	-	-	189.4	87	459.1	87	625.3	81	(4.0)	-
709	660	E9230a	CCCCCOC(=O)CCC	1.0	298.15	861.89	75	53.6	84	200.5	87	458.1	81	625.3	81	4.1	87
710	661	E9231a	CCCCCCOC(=O)CC	1.0	298.15	865.42	75	57.1	84	215.7	87	463.1	81	625.3	81	(4.0)	-
711	662	E9232a	CCCCCCCOC(C)=O	1.0	298.15	866.43	75	56.9	84	222.8	87	465.6	81	625.3	81	4.2	87
712	663	E9401a	CC(C)OC(=O)CC(=O)OC(C)C	1.0	298.0	-	-	63.9	84	-	-	509.2	81	-	-	(6.7)	-
713	664	E9402a	CCOC(=O)C(CC)C(=O)OCC	1.0	298.15	1000.71	75	-	-	-	-	481.1	81	-	-	(6.7)	-
714	665	E9403a	CCCOC(=O)CC(=O)OCCC	1.0	298.0	-	-	66.2	84	-	-	502.1	81	-	-	(6.7)	-
715	666	E9404a	CCOC(=O)CCCC(=O)OCC	1.0	293.15	1022.0	87	67.0	84	249.1	87	509.6	81	-	-	6.7	87
716	667	E9405a	COC(=O)CCCCCC(=O)OC	1.0	293.15	1039.1	75	73.5	84	252.2	87	509.2	81	-	-	(6.7)	-
717	668	E9406a	CC(=O)OCCCCCCOC(C)=O	1.0	293.15	1029.6	87	-	-	275.1	87	514.1	81	-	-	(6.7)	-

Table S.2 – Reference experimental data (continued)

n_{sim}	n_{iso}	Code	Smiles	P [bar]	T [K]	ρ_{liq} [kg·m^{-3}]	Src	ΔH_{vap} [kJ·mol^{-1}]	Src	T_m [K]	Src	T_b [K]	Src	T_c [K]	Src	ϵ	Src
718	669	E0201a	CCCCCCCCCOC=O	1.0	293.15	867.0	75	-	-	240.2	87	485.7	81	643.0	81	(6.9)	-
719	670	E0202a	CC(C)(C)COC(=O)C(C)(C)C	1.0	293.15	843.1	75	48.9	84	-	-	-	-	-	-	(4.0)	-
720	671	E0203a	CCC(C)(C)OC(=O)C(C)(C)C	1.0	298.0	-	-	48.0	84	-	-	-	-	-	-	(4.0)	-
721	672	E0204a	CCC(C)(C)OC(=O)CC(C)C	1.0	273.15	872.9	87	-	-	-	-	461.1	87	-	-	(4.0)	-
722	673	E0205a	CCOC(=O)C(CC)(CC)CC	1.0	289.15	883.7	75	-	-	-	-	-	-	-	-	(4.0)	-
723	674	E0206a	CCCCOC(=O)CC(C)(C)C	1.0	293.15	856.8	75	-	-	-	-	-	-	-	-	(4.0)	-
724	675	E0207a	CC(C)CCOC(=O)CC(C)C	1.0	298.15	854.1	75	-	-	-	-	463.1	87	-	-	4.4	87
725	676	E0208a	CC(=O)OC(C)CCCC(C)C	1.0	293.15	847.4	87	-	-	-	-	460.1	87	-	-	(4.0)	-
726	677	E0209a	CCCC(CCC)C(=O)OCC	1.0	293.15	865.9	75	-	-	-	-	-	-	-	-	(4.0)	-
727	678	E0210a	CCCCCC(CC)C(=O)OC	1.0	298.15	864.4	75	-	-	-	-	-	-	-	-	(4.0)	-
728	679	E0211a	CCCCC(CC)C(=O)OCC	1.0	298.15	858.6	87	-	-	-	-	473.7	81	643.0	81	(4.0)	-
729	680	E0212a	CCCCCCC(C)C(=O)OC	1.0	277.15	875.9	75	-	-	-	-	-	-	-	-	(4.0)	-
730	681	E0213a	CCCCCCOC(=O)C(C)C	1.0	293.15	870.0	75	-	-	-	-	-	-	-	-	(4.0)	-
731	682	E0214a	CCCC(=O)OCC(CC)CC	1.0	292.95	872.6	75	-	-	-	-	-	-	643.0	81	(4.0)	-
732	683	E0215a	CCCCC(=O)OCCC(C)C	1.0	293.15	858.0	75	-	-	-	-	466.1	87	643.0	81	3.6	87
733	684	E0216a	CCCCCC(=O)OC(C)CC	1.0	298.15	857.49	75	-	-	-	-	-	-	-	-	(4.0)	-
734	685	E0217a	CCCCC(CC)COC(C)=O	1.0	298.15	868.8	75	-	-	193.2	87	471.8	81	642.4	81	(4.0)	-
735	686	E0218a	CCCCCCC(C)OC(C)=O	1.0	298.15	858.09	75	-	-	-	-	-	-	643.0	81	(4.0)	-
736	687	E0219a	CCCCCCCCC(=O)OC	1.0	298.15	870.87	75	61.6	84	-	-	486.6	81	643.0	81	3.9	87
737	688	E0220a	CCCCCCCC(=O)OCC	1.0	298.15	862.92	75	59.5	84	228.4	87	481.6	81	643.0	81	(4.0)	-
738	689	E0221a	CCCCCCC(=O)OCCC	1.0	298.15	861.59	75	-	-	-	-	481.1	81	643.0	81	(4.0)	-
739	690	E0222a	CCCCCC(=O)OCCCC	1.0	298.15	862.3	75	-	-	223.2	87	481.1	81	643.0	81	(4.0)	-
740	691	E0223a	CCCCCOC(=O)CCCC	1.0	298.15	860.2	75	-	-	194.3	87	476.9	81	643.0	81	4.1	87
741	692	E0224a	CCCCCCOC(=O)CCC	1.01	298.15	851.0	78	-	-	195.2	87	479.1	81	643.0	81	(4.0)	-
742	693	E0225a	CCCCCCCOC(=O)CC	1.0	298.15	863.97	75	-	-	-	-	483.1	81	643.0	81	(4.0)	-
743	694	E0226a	CCCCCCCCOC(C)=O	1.0	298.15	864.3	75	-	-	235.2	87	484.4	81	643.0	81	4.2	87
744	695	E0401a	CCOC(=O)C(C(=O)OCC)C(C)C	1.0	293.15	996.1	87	-	-	-	-	488.1	81	-	-	(6.1)	-
745	696	E0402a	CC(C)OC(=O)CCC(=O)OC(C)C	1.0	298.15	980.26	75	70.8	84	-	-	-	-	-	-	(6.1)	-
746	697	E0403a	CCCC(C(=O)OCC)C(=O)OCC	1.0	298.15	982.75	75	-	-	-	-	494.1	81	-	-	(6.1)	-
747	698	E0404a	CCCOC(=O)CCC(=O)OCCC	1.0	298.15	997.36	75	-	-	267.2	87	524.0	81	-	-	(6.1)	-
748	699	E0405a	CCOC(=O)CCCCC(=O)OCC	1.0	298.15	1003.7	75	73.0	84	253.2	87	518.1	81	-	-	6.1	87
749	700	E0406a	COC(=O)CCCCCCC(=O)OC	1.0	298.15	1019.17	75	78.1	84	279.1	87	541.1	81	-	-	(6.1)	-
750	701	E0407a	CCCC(=O)OCCOC(=O)CCC	1.0	298.15	995.3	75	73.2	84	-	-	513.1	81	-	-	(6.1)	-
751	702	L1101a	CO	1.01	298.15	786.74	78	37.7	84	175.7	87	337.9	81	512.6	81	33.0	87
752	703	L2101a	CCO	1.01	298.15	786.6	78	42.26	84	159.0	87	351.4	81	513.9	81	25.3	87
753	704	L2201a	OCCO	1.0	298.15	1109.94	74	64.8	84	260.1	87	470.4	81	720.0	81	41.4	87
754	705	L3101a	CC(C)O	1.0	298.15	781.23	74	44.4	84	185.2	87	355.4	81	508.3	81	20.2	87
755	706	L3102a	CCCO	1.0	298.15	799.81	74	46.6	84	148.8	87	370.4	81	536.8	81	20.8	87
756	707	L3201a	CC(O)CO	1.0	298.15	1032.52	74	62.2	84	213.2	87	460.8	81	700.2	81	27.5	87
757	708	L3202a	OCCCO	1.0	298.15	1050.3	74	69.8	84	245.6	87	487.6	81	724.0	81	35.1	87
758	709	L3301a	OCC(O)CO	1.0	298.15	1258.3	74	-	-	291.4	87	563.1	81	850.0	81	46.5	87
759	709	L3301b	OCC(O)CO	1.0	308.0	-	-	85.8	84	291.4	87	563.1	81	850.0	81	46.5	87
760	710	L4101a	CC(C)(C)O	1.0	299.15	779.48	74	46.2	84	299.0	87	355.6	81	506.2	81	12.5	87
761	711	L4102a	CC(C)CO	1.0	298.15	797.81	74	50.79	84	171.2	87	380.8	81	547.8	81	17.9	87
762	712	L4103a	CCC(C)O	1.01	298.15	803.01	78	48.5	84	184.7	87	372.7	81	536.0	81	17.3	87
763	713	L4104a	CCCCO	1.0	298.15	805.8	78	52.1	84	184.6	87	390.8	81	563.0	81	17.8	87
764	714	L4201a	CC(C)(O)CO	1.0	298.15	989.6	74	-	-	-	-	451.1	81	728.8	81	(30.4)	-
765	715	L4202a	CC(O)C(C)O	1.0	298.15	999.8	74	-	-	-	-	455.1	81	728.8	81	(30.4)	-

Table S.2 – Reference experimental data (continued)

n_{sim}	n_{iso}	Code	Smiles	P [bar]	T [K]	ρ_{liq} [kg·m^{-3}]	Src	ΔH_{vap} [kJ·mol^{-1}]	Src	T_m [K]	Src	T_b [K]	Src	T$_c$ [K]	Src	ϵ	Src
766	716	L4203a	CC(CO)CO	1.0	293.15	1009.0	74	71.3	84	182.2	87	487.1	81	728.8	81	(30.4)	-
767	717	L4204a	CCC(O)CO	1.0	298.15	999.22	74	-	-	-	-	464.1	81	680.0	81	(30.4)	-
768	718	L4205a	CC(O)CCO	1.0	298.15	1000.2	74	72.6	84	196.2	87	480.1	81	676.0	81	28.8	87
769	719	L4206a	OCCCCO	1.0	298.15	1015.4	74	76.6	84	293.6	87	501.1	81	728.8	81	31.9	87
770	720	L4301a	OCCC(O)CO	1.0	298.15	1184.0	74	-	-	-	-	-	-	697.0	81	(39.0)	-
771	721	L5101a	CCC(C)(C)O	1.0	298.15	804.73	74	50.1	84	264.4	87	375.1	81	543.7	81	5.8	87
772	722	L5102a	CC(C)C(C)O	1.0	298.15	815.0	74	-	-	-	-	384.6	81	556.1	81	(12.0)	-
773	723	L5103a	CCC(C)CO	1.0	298.15	815.2	74	54.1	84	-	-	401.9	81	575.4	81	(12.0)	-
774	724	L5104a	CCC(O)CC	1.0	298.15	815.4	74	52.9	84	203.2	87	388.4	81	559.6	81	13.3	87
775	725	L5105a	CC(C)CCO	1.0	298.15	806.9	74	55.3	84	155.9	87	404.4	81	577.2	81	15.6	87
776	726	L5106a	CCCC(C)O	1.01	298.15	805.3	78	53.6	84	200.2	87	392.1	81	560.3	81	13.7	87
777	727	L5107a	CCCCCO	1.0	298.15	811.34	74	56.9	84	195.6	87	410.9	81	588.1	81	15.1	87
778	728	L5201a	CC(O)C(C)(C)O	1.0	298.15	968.8	74	-	-	-	-	447.1	81	754.5	81	(22.2)	-
779	729	L5202a	CC(C)(O)CCO	1.0	293.15	964.5	74	-	-	-	-	472.1	81	754.5	81	24.6	86
780	730	L5203a	CC(O)C(C)CO	1.0	293.15	991.7	74	-	-	-	-	473.1	81	754.5	81	(22.2)	-
781	731	L5204a	CC(C)C(O)CO	1.0	294.65	984.2	74	-	-	-	-	473.1	81	754.5	81	(22.2)	-
782	732	L5205a	CCC(O)C(C)O	1.0	292.15	979.8	87	-	-	-	-	460.6	81	754.5	81	17.4	87
783	733	L5206a	CC(O)CC(C)O	1.0	298.15	956.0	74	72.5	84	-	-	472.1	81	754.5	81	24.7	87
784	734	L5207a	CCC(CO)CO	1.0	293.15	997.0	74	-	-	-	-	475.2	81	754.5	81	(22.2)	-
785	735	L5208a	CCC(O)CCO	1.0	293.15	981.0	74	-	-	-	-	494.1	81	754.5	81	(22.2)	-
786	736	L5209a	CCCC(O)CO	1.0	297.15	969.1	74	74.6	84	-	-	482.1	81	754.5	81	(22.2)	-
787	737	L5210a	CC(O)CCCO	1.0	293.15	989.5	74	-	-	-	-	497.0	81	754.5	81	26.7	87
788	738	L5211a	OCCCCCO	1.0	298.15	989.71	74	86.8	84	253.2	87	512.1	81	754.5	81	26.2	87
789	739	L5301a	OCCC(O)CCO	1.0	298.15	1103.6	74	-	-	-	-	460.6	81	-	-	(39.0)	-
790	740	L6101a	CC(O)C(C)(C)C	0.01	298.15	813.91	81	53.8	84	-	-	393.1	81	596.0	81	(8.5)	-
791	741	L6102a	CC(C)C(C)(C)O	1.0	298.15	818.59	74	54.0	84	262.6	87	391.8	81	596.0	81	(8.5)	-
792	742	L6103a	CCC(C)(C)CO	1.0	298.15	824.5	74	-	-	-	-	409.9	81	596.0	81	10.5	87
793	743	L6104a	CCC(C)(O)CC	1.0	298.15	823.8	74	-	-	249.6	87	395.6	81	575.6	81	4.3	87
794	743	L6104b	CCC(C)(O)CC	1.0	337.0	-	-	40.1	84	249.6	87	395.6	81	575.6	81	4.3	87
795	744	L6105a	CC(C)(C)CCO	1.0	298.15	809.7	74	58.0	84	213.2	87	416.1	81	596.0	81	(8.5)	-
796	745	L6106a	CCCC(C)(C)O	1.0	298.15	809.45	74	54.7	84	170.2	87	394.6	81	559.5	81	(8.5)	-
797	746	L6107a	CC(C)C(C)CO	1.0	298.15	823.7	74	-	-	-	-	422.1	81	596.0	81	(8.5)	-
798	747	L6108a	CCC(C)C(C)O	1.0	298.15	824.73	74	58.2	84	-	-	407.4	81	596.0	81	(8.5)	-
799	748	L6109a	CCC(O)C(C)C	1.0	298.15	820.13	74	56.0	84	-	-	399.7	81	596.0	81	(8.5)	-
800	749	L6110a	CC(C)CC(C)O	1.0	298.15	803.04	74	-	-	183.2	87	404.9	81	574.4	81	(8.5)	-
801	749	L6110b	CC(C)CC(C)O	1.0	308.0	-	-	49.6	84	183.2	87	404.9	81	574.4	81	(8.5)	-
802	750	L6111a	CCC(CC)CO	1.0	298.15	829.27	74	60.3	84	-	-	419.6	81	596.0	81	6.2	87
803	751	L6112a	CCC(C)CCO	1.0	298.15	820.5	74	61.7	84	-	-	425.6	81	596.0	81	(8.5)	-
804	752	L6113a	CCCC(C)CO	1.0	298.15	820.63	74	59.4	84	-	-	421.1	81	604.4	81	(8.5)	-
805	753	L6114a	CCCC(O)CC	1.0	298.15	814.49	74	58.6	84	-	-	408.6	81	596.0	81	(8.5)	-
806	754	L6115a	CC(C)CCCO	1.0	298.15	809.72	74	-	-	-	-	424.9	81	603.5	81	(8.5)	-
807	755	L6116a	CCCCC(C)O	1.0	298.15	810.34	74	58.3	84	-	-	413.0	81	585.9	81	(8.5)	-
808	756	L6117a	CCCCCCO	1.0	298.15	815.54	74	59.6	84	226.8	87	430.1	81	610.3	81	13.0	87
809	757	L6201a	CC(C)(O)C(C)(C)O	0.002	316.45	970.4	81	-	-	316.4	87	445.9	81	777.8	81	(25.9)	-
810	758	L6202a	CCC(C)(O)C(C)O	1.0	298.15	963.8	74	-	-	-	-	505.8	81	777.8	81	(25.9)	-
811	759	L6203a	CC(C)(C)C(O)CO	1.0	323.15	940.0	74	-	-	-	-	478.6	81	777.8	81	(25.9)	-
812	760	L6204a	CC(CO)C(C)(C)O	1.0	298.15	964.5	74	-	-	-	-	480.0	81	777.8	81	(25.9)	-
813	761	L6205a	CCC(O)C(C)(C)O	1.0	293.15	962.7	74	-	-	-	-	505.8	81	777.8	81	(25.9)	-

Table S.2 – Reference experimental data (continued)

n_{sim}	n_{iso}	Code	Smiles	P [bar]	T [K]	ρ_{liq} [kg·m^{-3}]	Src	ΔH_{vap} [kJ·mol^{-1}]	Src	T_m [K]	Src	T_b [K]	Src	T$_c$ [K]	Src	ϵ	Src
814	762	L6206a	CC(O)CC(C)(C)O	1.0	298.15	918.53	74	68.6	84	223.2	87	470.6	81	777.8	81	25.9	87
815	763	L6207a	CC(O)C(C)C(C)O	1.0	287.15	990.6	74	-	-	-	-	484.6	81	777.8	81	(25.9)	-
816	764	L6208a	CCC(C)(CO)CO	1.0	323.15	958.2	74	-	-	-	-	498.9	81	777.8	81	(25.9)	-
817	765	L6209a	CC(C)(CO)CCO	1.0	277.15	996.0	74	-	-	-	-	480.0	81	777.8	81	(25.9)	-
818	766	L6210a	CCC(C)(O)CCO	1.0	293.15	969.0	74	-	-	-	-	483.0	81	777.8	81	(25.9)	-
819	767	L6211a	CC(C)(O)CCCO	1.0	293.15	964.5	74	-	-	-	-	494.4	81	777.8	81	(25.9)	-
820	768	L6212a	CC(C)C(CO)CO	1.0	293.15	976.8	74	-	-	-	-	496.9	81	777.8	81	(25.9)	-
821	769	L6213a	CCC(CO)C(C)O	1.0	298.15	967.7	74	-	-	-	-	480.0	81	777.8	81	(25.9)	-
822	770	L6214a	CC(CO)C(C)CO	1.0	293.15	977.1	74	-	-	-	-	-	-	-	-	(25.9)	-
823	771	L6215a	CCC(O)C(C)CO	1.0	295.15	973.7	74	-	-	-	-	493.4	81	777.8	81	(25.9)	-
824	772	L6216a	CCC(O)CC(C)O	1.0	294.15	951.6	74	-	-	-	-	484.1	81	777.8	81	(25.9)	-
825	773	L6217a	CC(O)CCC(C)O	1.0	323.15	939.8	74	-	-	316.1	87	493.9	81	777.8	81	(25.9)	-
826	774	L6218a	CCCC(CO)CO	1.0	298.15	963.6	74	-	-	-	-	474.2	81	777.8	81	(25.9)	-
827	775	L6219a	CC(CCO)CCO	1.0	293.15	972.6	74	-	-	-	-	521.5	81	777.8	81	(25.9)	-
828	776	L6220a	CC(CO)CCCO	1.0	293.15	971.9	74	-	-	-	-	496.9	81	777.8	81	(25.9)	-
829	777	L6221a	CCCC(O)CCO	1.0	295.15	958.0	74	-	-	-	-	508.1	81	777.8	81	(25.9)	-
830	778	L6222a	CCC(O)CCCO	1.0	289.45	982.0	74	-	-	-	-	529.1	81	777.8	81	(25.9)	-
831	779	L6223a	CCCCC(O)CO	0.01	384.77	917.43	81	-	-	318.1	87	497.1	81	777.8	81	(25.9)	-
832	780	L6224a	CC(O)CCCCO	1.0	298.15	964.0	74	-	-	-	-	510.1	81	777.8	81	(25.9)	-
833	781	L6225a	OCCCCCCO	1.0	318.15	968.3	74	-	-	314.6	87	516.1	81	777.8	81	(25.9)	-
834	781	L6225b	OCCCCCCO	1.0	342.0	-	-	87.0	84	314.6	87	516.1	81	777.8	81	(25.9)	-
835	782	L6301a	OCCCC(O)CCO	1.0	293.15	1104.1	74	-	-	-	-	-	-	-	-	(31.5)	-
836	783	L6302a	OCCCCC(O)CO	1.0	298.15	1099.98	74	-	-	-	-	472.5	81	-	-	31.5	87
837	784	L7101a	CC(C)(C)C(C)(C)O	1.0	298.15	833.5	74	-	-	290.1	87	404.1	81	619.7	81	(6.2)	-
838	784	L7101b	CC(C)(C)C(C)(C)O	1.0	313.0	-	-	48.7	84	290.1	87	404.1	81	619.7	81	(6.2)	-
839	785	L7102a	CC(C)C(C)(C)CO	1.0	293.15	846.6	74	-	-	-	-	430.1	81	619.7	81	(6.2)	-
840	786	L7103a	CCC(C)(C)C(C)O	1.0	293.15	827.0	74	-	-	-	-	420.1	81	619.7	81	(6.2)	-
841	787	L7104a	CCC(C)(O)C(C)C	1.0	298.15	837.27	74	-	-	-	-	413.1	81	619.7	81	(6.2)	-
842	788	L7105a	CC(CO)C(C)(C)C	1.0	298.15	823.8	74	-	-	-	-	433.1	81	619.7	81	(6.2)	-
843	789	L7106a	CCC(O)C(C)(C)C	1.0	298.15	822.4	74	-	-	282.4	87	409.1	81	619.7	81	(6.2)	-
844	790	L7107a	CCC(C)C(C)(C)O	1.0	298.15	828.5	74	-	-	-	-	412.1	81	619.7	81	(6.2)	-
845	791	L7108a	CC(O)CC(C)(C)C	1.0	293.15	811.9	74	-	-	-	-	411.1	81	619.7	81	(6.2)	-
846	792	L7109a	CC(C)CC(C)(C)O	1.0	298.15	810.0	74	-	-	-	-	406.1	81	619.7	81	(6.2)	-
847	793	L7110a	CC(C)C(C)C(C)O	1.0	294.15	836.0	74	-	-	-	-	426.1	81	619.7	81	(6.2)	-
848	794	L7111a	CC(C)C(O)C(C)C	1.0	298.15	824.94	74	-	-	-	-	411.9	81	619.7	81	(6.2)	-
849	794	L7111b	CC(C)C(O)C(C)C	1.0	322.0	-	-	53.6	84	-	-	411.9	81	619.7	81	(6.2)	-
850	795	L7112a	CCC(C)(CC)CO	1.0	293.15	828.2	74	-	-	-	-	430.1	81	619.7	81	(6.2)	-
851	796	L7113a	CCC(O)(CC)CC	1.0	298.15	839.56	74	57.3	84	260.1	87	415.6	81	619.7	81	3.2	87
852	797	L7114a	CCC(C)(C)CCO	1.0	293.15	832.0	74	-	-	-	-	438.1	81	619.7	81	(6.2)	-
853	798	L7115a	CCCC(C)(C)CO	1.0	293.15	837.9	74	-	-	-	-	426.1	81	619.7	81	6.0	87
854	799	L7116a	CCCC(C)(O)CC	1.0	298.15	820.18	74	-	-	-	-	415.9	81	619.7	81	3.2	87
855	800	L7117a	CC(C)(C)CCCO	1.0	293.15	815.1	74	-	-	-	-	433.1	81	619.7	81	(6.2)	-
856	801	L7118a	CCCCC(C)(C)O	1.0	298.15	809.8	74	58.6	84	-	-	415.9	81	619.7	81	3.3	87
857	802	L7119a	CCC(CO)C(C)C	1.0	298.15	832.7	74	-	-	-	-	435.1	81	619.7	81	(6.2)	-
858	803	L7120a	CCC(CC)C(C)O	1.0	298.15	833.3	74	-	-	-	-	425.1	81	619.7	81	(6.2)	-
859	804	L7121a	CCC(C)C(C)CO	1.0	296.15	836.0	74	-	-	-	-	437.1	81	619.7	81	(6.2)	-
860	805	L7122a	CC(C)C(C)CCO	1.0	297.15	819.0	74	-	-	-	-	438.1	81	619.7	81	(6.2)	-
861	806	L7123a	CCCC(C)C(C)O	1.0	298.15	822.0	87	-	-	-	-	425.1	81	619.7	81	5.0	87

Table S.2 – Reference experimental data (continued)

n_{sim}	n_{iso}	Code	Smiles	P [bar]	T [K]	ρ_{liq} [kg·m^{-3}]	Src	ΔH_{vap} [kJ·mol^{-1}]	Src	T_m [K]	Src	T_b [K]	Src	T$_c$ [K]	Src	ϵ	Src
862	807	L7124a	CCCC(O)C(C)C	1.0	293.15	823.9	74	-	-	-	-	418.1	81	619.7	81	(6.2)	-
863	808	L7125a	CC(C)CC(C)CO	1.0	298.15	816.0	74	-	-	-	-	432.1	81	619.7	81	(6.2)	-
864	809	L7126a	CCC(C)CC(C)O	1.0	298.15	817.7	74	-	-	-	-	424.1	81	619.7	81	(6.2)	-
865	810	L7127a	CCC(O)CC(C)C	1.0	293.15	833.1	74	59.8	84	-	-	421.1	81	619.7	81	(6.2)	-
866	811	L7128a	CC(C)CCC(C)O	1.0	293.15	813.1	74	-	-	-	-	424.1	81	619.7	81	(6.2)	-
867	812	L7129a	CCCC(CC)CO	1.0	298.15	828.8	74	-	-	-	-	439.1	81	619.7	81	(6.2)	-
868	813	L7130a	CCCC(C)CCO	1.0	298.15	824.5	74	-	-	-	-	445.1	81	619.7	81	(6.2)	-
869	814	L7131a	CCC(C)CCCO	1.0	297.15	821.0	74	-	-	-	-	446.1	81	619.7	81	(6.2)	-
870	815	L7132a	CCCCC(C)CO	1.0	293.15	827.0	74	-	-	-	-	436.1	81	619.7	81	10.7	86
871	816	L7133a	CCCC(O)CCC	1.0	298.15	815.6	74	62.4	84	231.9	87	427.9	81	619.7	81	6.2	87
872	817	L7134a	CCCCC(O)CC	1.0	298.15	816.5	74	-	-	-	-	429.9	81	619.7	81	(6.2)	-
873	818	L7135a	CC(C)CCCCO	1.0	298.15	819.2	74	-	-	-	-	445.1	81	619.7	81	(6.2)	-
874	819	L7136a	CCCCCC(C)O	1.0	298.15	813.38	74	62.1	84	-	-	432.4	81	608.3	81	(6.2)	-
875	820	L7137a	CCCCCCCO	1.0	298.15	819.17	74	66.5	84	239.9	87	449.4	81	632.6	81	11.8	87
876	821	L7201a	CCC(C)(O)C(C)(C)O	1.0	293.15	961.3	74	-	-	-	-	525.9	81	799.3	81	(26.4)	-
877	822	L7202a	CC(CCO)C(C)(C)O	1.0	293.15	952.8	74	-	-	-	-	517.0	81	799.3	81	(26.4)	-
878	823	L7203a	CCC(C)(O)CC(C)O	1.0	293.15	929.6	74	-	-	-	-	517.0	81	799.3	81	(26.4)	-
879	824	L7204a	CCC(O)CC(C)(C)O	1.0	291.15	932.1	74	-	-	-	-	517.0	81	799.3	81	(26.4)	-
880	825	L7205a	CCC(CC)(CO)CO	1.0	334.45	949.0	74	-	-	334.4	87	507.1	81	799.3	81	(26.4)	-
881	826	L7206a	CC(C)(O)CCCCO	1.0	295.15	967.0	74	-	-	-	-	494.2	81	799.3	81	(26.4)	-
882	827	L7207a	CC(C)C(CO)CCO	1.0	293.15	967.2	74	-	-	-	-	508.2	81	799.3	81	(26.4)	-
883	828	L7208a	CCCC(O)CC(C)O	1.0	298.15	926.0	74	-	-	-	-	508.2	81	799.3	81	(26.4)	-
884	829	L7209a	CCCC(CO)CCO	1.0	293.15	962.5	74	-	-	-	-	485.4	81	799.3	81	(26.4)	-
885	830	L7210a	CCC(CO)CCCO	1.0	293.15	967.9	74	-	-	-	-	485.4	81	799.3	81	(26.4)	-
886	831	L7211a	CCCC(O)CCCO	1.0	298.15	950.4	74	-	-	-	-	515.1	81	799.3	81	(26.4)	-
887	832	L7212a	CCC(O)CCCCO	1.0	293.15	970.5	74	-	-	-	-	489.8	81	799.3	81	(26.4)	-
888	833	L7213a	CC(O)CCCCCO	1.0	298.15	962.0	74	-	-	-	-	485.4	81	799.3	81	(26.4)	-
889	834	L7214a	OCCCCCCCO	1.0	298.15	952.25	74	96.5	84	295.1	87	535.1	81	799.3	81	(26.4)	-
890	835	L7301a	OCCCC(O)CCCO	1.0	291.0	1075.0	74	-	-	-	-	416.9	81	-	-	(39.0)	-
891	836	L8101a	CCC(C)(C)C(C)(C)O	1.0	298.15	815.1	74	-	-	-	-	433.1	81	641.4	81	(5.5)	-
892	837	L8102a	CCC(C)(O)C(C)(C)C	1.0	298.15	842.91	74	-	-	-	-	425.1	81	641.4	81	(5.5)	-
893	838	L8103a	CC(C)(C)CC(C)(C)O	1.0	293.15	823.7	74	-	-	253.2	87	419.6	81	641.4	81	(5.5)	-
894	839	L8104a	CC(C)C(C)(O)C(C)C	1.0	293.15	849.2	74	-	-	-	-	430.1	81	641.4	81	(5.5)	-
895	840	L8105a	CC(O)C(C)C(C)(C)C	1.0	293.15	840.8	74	-	-	-	-	431.1	81	641.4	81	(5.5)	-
896	841	L8106a	CC(C)C(O)C(C)(C)C	1.0	293.15	832.4	74	-	-	260.1	87	424.1	81	641.4	81	(5.5)	-
897	842	L8107a	CC(C)C(C)C(C)(C)O	1.0	293.15	808.0	74	-	-	-	-	431.1	81	641.4	81	(5.5)	-
898	843	L8108a	CCC(C)(CC)C(C)O	1.0	293.15	857.6	74	-	-	-	-	433.0	81	641.4	81	(5.5)	-
899	844	L8109a	CCC(O)(CC)C(C)C	1.0	293.15	829.5	74	-	-	-	-	433.1	81	641.4	81	(5.5)	-
900	845	L8110a	CCC(O)C(C)(C)CC	1.0	293.15	834.1	74	-	-	-	-	432.1	81	641.4	81	(5.5)	-
901	846	L8111a	CCCC(C)(C)C(C)O	1.0	293.15	845.7	74	-	-	-	-	433.0	81	641.4	81	(5.5)	-
902	847	L8112a	CCC(C)C(C)(O)CC	1.0	298.15	834.5	74	-	-	-	-	425.1	81	641.4	81	(5.5)	-
903	848	L8113a	CCCC(C)(O)C(C)C	1.0	293.15	837.1	74	-	-	-	-	431.4	81	641.4	81	(5.5)	-
904	849	L8114a	CCC(CO)C(C)(C)C	1.0	298.15	842.5	74	-	-	-	-	446.0	81	641.4	81	(5.5)	-
905	850	L8115a	CCCC(O)C(C)(C)C	1.0	293.15	834.2	74	-	-	-	-	429.2	81	641.4	81	(5.5)	-
906	851	L8116a	CCC(CC)C(C)(C)O	1.0	298.15	834.6	74	-	-	-	-	430.9	81	641.4	81	(5.5)	-
907	852	L8117a	CCCC(C)C(C)(C)O	1.0	298.15	831.0	74	-	-	-	-	433.2	81	641.4	81	(5.5)	-
908	853	L8118a	CC(C)CC(C)(C)CO	1.0	293.15	838.4	74	-	-	-	-	441.4	81	641.4	81	(5.5)	-
909	853	L8118b	CC(C)CC(C)(C)CO	1.0	348.0	-	-	54.7	84	-	-	441.4	81	641.4	81	(5.5)	-

Table S.2 – Reference experimental data (continued)

n_{sim}	n_{iso}	Code	Smiles	P [bar]	T [K]	ρ_{liq} [kg·m^{-3}]	Src	ΔH_{vap} [kJ·mol^{-1}]	Src	T_m [K]	Src	T_b [K]	Src	T$_c$ [K]	Src	ϵ	Src
910	854	L8119a	CCC(C)(O)CC(C)C	1.0	298.15	827.0	74	-	-	-	-	425.1	81	641.4	81	(5.5)	-
911	855	L8120a	CC(CO)CC(C)(C)C	1.0	293.15	833.0	74	-	-	-	-	444.1	81	641.4	81	(5.5)	-
912	856	L8121a	CCC(O)CC(C)(C)C	1.0	293.15	833.9	74	-	-	-	-	426.1	81	641.4	81	(5.5)	-
913	857	L8122a	CCC(C)CC(C)(C)O	1.0	301.15	806.5	74	-	-	-	-	423.9	81	641.4	81	(5.5)	-
914	858	L8123a	CC(C)CCC(C)(C)O	1.0	298.15	811.51	74	-	-	-	-	425.6	81	641.4	81	(5.5)	-
915	859	L8124a	CC(C)C(CO)C(C)C	1.0	298.15	842.5	74	-	-	-	-	446.1	81	641.4	81	(5.5)	-
916	860	L8125a	CC(C)C(C)C(C)CO	1.0	293.15	849.8	74	-	-	-	-	456.1	81	641.4	81	(5.5)	-
917	861	L8126a	CC(C)CC(O)C(C)C	1.0	298.15	813.53	74	-	-	-	-	432.1	81	641.4	81	(5.5)	-
918	862	L8127a	CCCC(O)(CC)CC	1.0	298.15	834.2	74	-	-	-	-	432.1	81	641.4	81	(5.5)	-
919	863	L8128a	CCCC(C)(C)CCO	1.0	293.15	839.0	74	-	-	-	-	454.0	81	641.4	81	(5.5)	-
920	864	L8129a	CCCCC(C)(C)CO	1.0	293.15	826.5	74	-	-	-	-	445.6	81	641.4	81	4.5	87
921	865	L8130a	CCCC(C)(O)CCC	1.0	298.15	820.2	74	-	-	191.2	87	434.2	81	641.4	81	2.9	87
922	865	L8130b	CCCC(C)(O)CCC	1.0	345.0	-	-	54.8	84	191.2	87	434.2	81	641.4	81	2.9	87
923	866	L8131a	CCCCC(C)(O)CC	1.0	298.15	824.9	74	-	-	190.2	87	434.1	81	641.4	81	3.0	87
924	866	L8131b	CCCCC(C)(O)CC	1.0	353.0	-	-	54.7	84	190.2	87	434.1	81	641.4	81	3.0	87
925	867	L8132a	CCCCCC(C)(C)O	1.0	298.15	805.0	74	-	-	222.8	87	429.9	81	641.4	81	3.4	87
926	867	L8132b	CCCCCC(C)(C)O	1.0	358.0	-	-	53.1	84	222.8	87	429.9	81	641.4	81	3.4	87
927	868	L8133a	CCC(O)C(CC)CC	0.01	332.39	812.92	81	-	-	-	-	437.1	81	641.4	81	(5.5)	-
928	869	L8134a	CCCC(C)C(O)CC	1.0	298.15	794.16	74	-	-	150.2	87	428.6	81	641.4	81	3.3	87
929	869	L8134b	CCCC(C)C(O)CC	0.01	318.66	-	-	43.54	81	150.2	87	428.6	81	641.4	81	3.3	87
930	870	L8135a	CCCC(O)C(C)CC	1.0	298.15	833.5	74	-	-	-	-	437.9	81	641.4	81	7.5	87
931	870	L8135b	CCCC(O)C(C)CC	1.0	355.0	-	-	48.0	84	-	-	437.9	81	641.4	81	7.5	87
932	871	L8136a	CCCCC(C)C(C)O	1.0	298.15	817.63	74	-	-	-	-	439.2	81	641.4	81	7.5	87
933	871	L8136b	CCCCC(C)C(C)O	1.0	356.0	-	-	48.0	84	-	-	439.2	81	641.4	81	7.5	87
934	872	L8137a	CCCCC(O)C(C)C	1.0	298.15	821.0	74	-	-	-	-	440.8	81	641.4	81	(5.5)	-
935	872	L8137b	CCCCC(O)C(C)C	1.0	364.0	-	-	54.8	84	-	-	440.8	81	641.4	81	(5.5)	-
936	873	L8138a	CCC(CO)CC(C)C	1.0	293.15	827.3	74	-	-	-	-	449.6	81	641.4	81	(5.5)	-
937	873	L8138b	CCC(CO)CC(C)C	1.0	365.0	-	-	53.3	84	-	-	449.6	81	641.4	81	(5.5)	-
938	874	L8139a	CCC(C)CC(O)CC	1.0	298.15	814.14	74	-	-	181.9	87	426.8	81	641.4	81	3.8	87
939	875	L8140a	CC(C)CC(C)CCO	1.0	293.15	828.2	74	-	-	-	-	456.1	81	641.4	81	(5.5)	-
940	876	L8141a	CCCC(C)CC(C)O	1.0	298.15	799.0	74	-	-	171.2	87	444.8	81	641.4	81	3.6	87
941	877	L8142a	CC(C)CCC(C)CO	1.0	298.15	824.54	74	-	-	-	-	452.1	81	641.4	81	(5.5)	-
942	878	L8143a	CCC(C)CCC(C)O	1.0	298.15	810.0	74	-	-	212.2	87	445.1	81	641.4	81	7.5	87
943	879	L8144a	CCCC(O)CC(C)C	1.0	298.15	809.8	74	-	-	192.2	87	439.2	81	641.4	81	3.3	87
944	880	L8145a	CCC(O)CCC(C)C	1.0	298.15	776.6	74	-	-	-	-	433.0	81	641.4	81	(5.5)	-
945	881	L8146a	CC(C)CCCC(C)O	1.0	298.15	803.4	74	-	-	168.2	87	445.1	81	641.4	81	6.4	87
946	882	L8147a	CCCC(CC)CCO	1.0	301.15	829.0	74	-	-	-	-	453.0	81	641.4	81	(5.5)	-
947	883	L8148a	CCCCC(CC)CO	1.0	298.15	828.7	74	68.5	84	203.2	87	457.8	81	640.6	81	7.6	87
948	884	L8149a	CCCC(C)CCCO	1.0	298.15	806.02	74	-	-	-	-	456.4	81	641.4	81	4.6	87
949	885	L8150a	CCCCC(C)CCO	1.0	298.15	784.5	74	-	-	183.2	87	459.1	81	641.4	81	2.9	87
950	886	L8151a	CCC(C)CCCCO	1.0	298.15	815.2	74	-	-	-	-	459.8	81	641.4	81	(5.5)	-
951	887	L8152a	CCCCCC(C)CO	1.0	298.15	798.7	74	-	-	-	-	448.8	81	641.4	81	(5.5)	-
952	888	L8153a	CCCCC(O)CCC	1.0	298.15	815.9	74	67.2	84	-	-	449.8	81	641.4	81	(5.5)	-
953	889	L8154a	CCCCCC(O)CC	1.0	298.15	817.01	74	-	-	228.2	87	447.9	81	641.4	81	5.5	87
954	890	L8155a	CC(C)CCCCCO	1.0	298.15	817.56	74	-	-	167.2	87	460.9	81	641.4	81	10.5	87
955	891	L8156a	CCCCCCC(C)O	1.0	298.15	817.0	74	67.9	84	-	-	452.9	81	629.6	81	(5.5)	-
956	892	L8157a	CCCCCCCCO	1.0	298.15	821.79	74	70.1	84	258.4	87	468.4	81	652.5	81	10.3	87
957	893	L8201a	CC(C)CC(C)(O)C(C)O	1.0	298.15	928.5	74	-	-	-	-	-	-	-	-	(18.7)	-

Table S.2 – Reference experimental data (continued)

$n_{\rm sim}$	$n_{\rm iso}$	Code	Smiles	P [bar]	T [K]	$\rho_{\rm liq}$ [kg·m^{-3}]	Src	$\Delta H_{\rm vap}$ [kJ·mol^{-1}]	Src	T_m [K]	Src	T_b [K]	Src	T$_c$ [K]	Src	ϵ	Src
958	894	L8202a	CC(C)C(O)CC(C)(C)O	1.0	293.15	917.2	74	-	-	-	-	-	-	-	-	(18.7)	-
959	895	L8203a	CCCCC(O)C(C)(C)O	1.0	298.15	929.0	74	-	-	-	-	-	-	-	-	(18.7)	-
960	896	L8204a	CCC(O)(CC)CCCO	1.0	292.15	970.4	74	-	-	-	-	-	-	-	-	(18.7)	-
961	897	L8205a	CC(C)(O)CCCCCO	1.0	298.15	959.0	74	-	-	-	-	-	-	-	-	(18.7)	-
962	898	L8206a	CCCC(O)C(CC)CO	1.0	295.15	932.5	87	-	-	233.2	87	517.1	81	819.3	81	18.7	87
963	899	L8207a	CCCCC(O)CC(C)O	1.0	298.15	918.0	74	-	-	-	-	478.1	81	819.3	81	(18.7)	-
964	900	L8208a	CCCC(O)CCCCO	1.0	298.15	949.0	74	-	-	-	-	-	-	-	-	(18.7)	-
965	901	L8209a	CC(O)CCCCCCO	1.0	298.15	943.0	74	-	-	-	-	-	-	-	-	(18.7)	-
966	902	L8210a	OCCCCCCCCO	1.0	356.0	-	-	101.0	84	332.8	87	544.1	81	819.3	81	(18.7)	-
967	903	L9101a	CCC(O)(CC)C(C)(C)C	1.0	298.15	852.6	74	-	-	-	-	447.1	81	661.5	81	(6.2)	-
968	904	L9102a	CCC(C)(C)C(C)(O)CC	1.0	294.15	832.3	74	-	-	-	-	439.1	81	661.5	81	(6.2)	-
969	905	L9103a	CCCC(C)(O)C(C)(C)C	1.0	293.15	846.2	74	-	-	-	-	446.2	81	661.5	81	(6.2)	-
970	906	L9104a	CCC(C)(C)CC(C)(C)O	1.0	293.15	847.5	74	-	-	-	-	466.0	81	661.5	81	(6.2)	-
971	907	L9105a	CCC(C)(O)CC(C)(C)C	1.0	293.15	835.0	74	-	-	-	-	439.0	81	661.5	81	(6.2)	-
972	908	L9106a	CCC(O)(C(C)C)C(C)C	1.0	298.15	854.92	74	-	-	-	-	451.1	81	661.5	81	(6.2)	-
973	909	L9107a	CCC(C)(C)C(O)C(C)C	1.0	293.15	848.8	74	-	-	-	-	444.1	81	661.5	81	(6.2)	-
974	910	L9108a	CC(C)CC(C)(O)C(C)C	1.0	293.15	825.6	74	-	-	-	-	439.0	81	661.5	81	(6.2)	-
975	911	L9109a	CCC(C)C(C)C(C)(C)O	1.0	288.15	835.3	74	-	-	-	-	466.0	81	661.5	81	(6.2)	-
976	912	L9110a	CC(C)C(O)CC(C)(C)C	1.0	293.15	825.0	74	-	-	-	-	442.0	81	661.5	81	(6.2)	-
977	913	L9111a	CCC(C)C(O)(CC)CC	1.0	288.15	861.0	74	-	-	-	-	452.0	81	661.5	81	(6.2)	-
978	914	L9112a	CCCC(O)(CC)C(C)C	1.0	298.15	844.5	74	-	-	-	-	457.2	81	661.5	81	(6.2)	-
979	915	L9113a	CCC(CC)C(C)(O)CC	1.0	298.15	899.4	74	-	-	-	-	452.0	81	661.5	81	(6.2)	-
980	916	L9114a	CCCCC(C)(O)C(C)C	1.0	294.15	838.3	74	-	-	-	-	447.1	81	661.5	81	(6.2)	-
981	917	L9115a	CC(CCCO)C(C)(C)C	1.0	293.15	846.0	74	-	-	-	-	475.1	81	661.5	81	(6.2)	-
982	918	L9116a	CCCCC(O)C(C)(C)C	1.0	298.15	823.74	74	-	-	-	-	451.0	81	661.5	81	(6.2)	-
983	919	L9117a	CCCC(CC)C(C)(C)O	1.0	298.15	833.4	74	-	-	-	-	451.1	81	661.5	81	(6.2)	-
984	920	L9118a	CCC(C)(CO)CC(C)C	1.0	298.15	837.0	74	-	-	-	-	461.1	81	661.5	81	(6.2)	-
985	921	L9119a	CCC(O)(CC)CC(C)C	1.0	295.15	839.6	74	-	-	-	-	445.1	81	661.5	81	(6.2)	-
986	922	L9120a	CCC(C)CC(C)(O)CC	1.0	298.15	821.83	74	-	-	-	-	450.0	81	661.5	81	(6.2)	-
987	923	L9121a	CCCC(C)(O)CC(C)C	1.0	293.15	824.2	74	-	-	-	-	444.6	81	661.5	81	(6.2)	-
988	924	L9122a	CCC(C)(O)CCC(C)C	1.0	289.15	828.5	74	-	-	-	-	446.1	81	661.5	81	(6.2)	-
989	925	L9123a	CC(CCO)CC(C)(C)C	1.0	298.15	823.6	87	67.9	84	-	-	466.1	81	661.5	81	(6.2)	-
990	926	L9124a	CCCC(C)CC(C)(C)O	1.0	298.15	828.0	74	-	-	-	-	455.0	81	661.5	81	(6.2)	-
991	927	L9125a	CCC(C)CCC(C)(C)O	1.0	295.15	830.0	74	-	-	-	-	-	-	-	-	(6.2)	-
992	928	L9126a	CC(C)CCCC(C)(C)O	1.0	293.15	818.6	87	-	-	-	-	444.1	81	661.5	81	(6.2)	-
993	929	L9127a	CCC(CC)C(O)C(C)C	1.0	293.15	827.5	74	-	-	-	-	452.0	81	661.5	81	(6.2)	-
994	930	L9128a	CCC(C)C(O)C(C)CC	1.0	293.15	859.2	74	-	-	-	-	460.1	81	661.5	81	(6.2)	-
995	931	L9129a	CC(C)CCC(O)C(C)C	1.0	293.15	814.8	74	-	-	-	-	448.1	81	661.5	81	(6.2)	-
996	932	L9130a	CC(C)CC(C)CC(C)O	1.0	273.15	878.7	74	-	-	-	-	467.1	81	661.5	81	(6.2)	-
997	933	L9131a	CC(C)CC(O)CC(C)C	1.0	298.15	806.43	74	65.2	84	-	-	451.0	81	661.5	81	(6.2)	-
998	934	L9132a	CCCC(O)(CC)CCC	1.0	298.15	829.9	74	-	-	-	-	452.1	81	661.5	81	(6.2)	-
999	935	L9133a	CCCCC(O)(CC)CC	1.0	298.15	829.9	74	-	-	-	-	455.4	81	661.5	81	(6.2)	-
1000	936	L9134a	CCCCC(C)(O)CCC	1.0	298.15	824.56	74	-	-	-	-	454.1	81	661.5	81	(6.2)	-
1001	937	L9135a	CCCCCC(C)(O)CC	1.0	298.15	827.5	74	-	-	-	-	-	-	-	-	(6.2)	-
1002	938	L9136a	CC(C)(C)CCCCCO	1.0	293.15	843.9	74	-	-	-	-	455.0	81	661.5	81	(6.2)	-
1003	939	L9137a	CCCCCCC(C)(C)O	1.0	298.15	813.4	74	-	-	-	-	451.1	81	661.5	81	(6.2)	-
1004	940	L9138a	CCCC(C)C(CC)CO	1.0	298.15	835.8	74	-	-	-	-	466.1	81	661.5	81	(6.2)	-
1005	941	L9139a	CCCC(C)C(O)CCC	1.0	298.15	815.6	74	-	-	-	-	455.0	81	661.5	81	(6.2)	-

Table S.2 – Reference experimental data (continued)

n_{sim}	n_{iso}	Code	Smiles	P [bar]	T [K]	ρ_{liq} [kg·m^{-3}]	Src	ΔH_{vap} [kJ·mol^{-1}]	Src	T_m [K]	Src	T_b [K]	Src	T$_c$ [K]	Src	ϵ	Src
1006	942	L9140a	CCCCC(C)C(O)CC	1.0	298.15	843.7	74	-	-	-	-	459.0	81	661.5	81	(6.2)	-
1007	943	L9141a	CCCCC(O)C(C)CC	1.0	291.15	834.0	74	-	-	-	-	453.1	81	661.5	81	(6.2)	-
1008	944	L9142a	CCCCCC(C)C(C)O	1.0	300.15	831.0	74	-	-	-	-	454.0	81	661.5	81	(6.2)	-
1009	945	L9143a	CCCCCC(O)C(C)C	1.0	293.15	827.0	74	-	-	-	-	457.1	81	661.5	81	(6.2)	-
1010	946	L9144a	CCC(C)CC(CC)CO	1.0	293.15	828.8	74	-	-	-	-	468.1	81	661.5	81	(6.2)	-
1011	947	L9145a	CCCC(CO)CC(C)C	1.0	293.15	825.6	74	-	-	-	-	465.1	81	661.5	81	(6.2)	-
1012	948	L9146a	CCC(CO)CCC(C)C	1.0	298.15	820.8	74	-	-	-	-	466.0	81	661.5	81	(6.2)	-
1013	949	L9147a	CCCC(O)CC(C)CC	1.0	296.15	822.0	74	-	-	-	-	455.0	81	661.5	81	(6.2)	-
1014	950	L9148a	CCC(C)CCC(O)CC	1.0	301.15	832.0	74	-	-	-	-	459.0	81	661.5	81	(6.2)	-
1015	951	L9149a	CC(C)CCC(C)CCO	1.0	300.15	823.0	74	-	-	-	-	-	-	-	-	(6.2)	-
1016	952	L9150a	CCCC(C)CCC(C)O	1.0	298.15	821.0	74	-	-	-	-	454.0	81	661.5	81	(6.2)	-
1017	953	L9151a	CCCCC(O)CC(C)C	1.0	298.15	815.0	74	-	-	-	-	457.1	81	661.5	81	(6.2)	-
1018	954	L9152a	CCCC(O)CCC(C)C	1.0	293.15	813.6	74	-	-	-	-	455.0	81	661.5	81	(6.2)	-
1019	955	L9153a	CCC(O)CCCC(C)C	1.0	285.15	840.2	74	-	-	-	-	459.0	81	661.5	81	(6.2)	-
1020	956	L9154a	CCCCC(CC)CCO	1.0	296.15	834.0	74	-	-	-	-	480.1	81	661.5	81	(6.2)	-
1021	957	L9155a	CCC(CC)CCCCO	1.0	298.15	848.0	74	-	-	-	-	472.0	81	661.5	81	(6.2)	-
1022	958	L9156a	CCCCC(C)CCCO	1.0	300.65	820.0	74	-	-	-	-	473.0	81	661.5	81	(6.2)	-
1023	959	L9157a	CCCC(C)CCCCO	1.0	297.15	828.0	74	-	-	-	-	473.0	81	661.5	81	(6.2)	-
1024	960	L9158a	CCCCCC(C)CCO	1.0	297.15	827.0	74	-	-	-	-	473.0	81	661.5	81	(6.2)	-
1025	961	L9159a	CCCCCCC(C)CO	1.0	277.15	841.8	74	-	-	-	-	473.0	81	661.5	81	(6.2)	-
1026	962	L9160a	CCCCC(O)CCCC	1.0	298.15	818.3	74	71.4	84	278.8	87	468.2	81	661.5	81	3.5	87
1027	963	L9161a	CCCCCC(O)CCC	1.0	293.15	826.3	74	71.5	84	-	-	466.1	81	661.5	81	(6.2)	-
1028	964	L9162a	CCCCCCC(O)CC	1.0	298.15	823.55	74	70.9	84	-	-	467.9	81	661.5	81	(6.2)	-
1029	965	L9163a	CC(C)CCCCCCO	1.0	298.15	826.0	74	-	-	-	-	479.1	81	661.5	81	(6.2)	-
1030	966	L9164a	CCCCCCCC(C)O	1.0	298.15	819.35	74	72.9	84	-	-	471.6	81	649.6	81	(6.2)	-
1031	967	L9165a	CCCCCCCCCO	1.0	298.15	824.6	74	72.2	84	268.1	87	486.2	81	670.7	81	8.8	87
1032	968	L9201a	CC(C)C(C)(O)CC(C)(C)O	1.0	301.35	920.7	74	-	-	-	-	-	-	-	-	(26.4)	-
1033	969	L9202a	CCCC(C)(O)CC(C)(C)O	1.0	290.35	913.8	74	-	-	-	-	-	-	-	-	(26.4)	-
1034	970	L9203a	CC(C)CC(O)CC(C)(C)O	1.0	291.15	902.0	74	-	-	-	-	-	-	-	-	(26.4)	-
1035	971	L9204a	CCCCC(CC)(CO)CO	1.0	323.15	929.0	74	-	-	316.1	87	535.1	81	837.9	81	(26.4)	-
1036	971	L9204b	CCCCC(CC)(CO)CO	1.0	460.0	-	-	67.2	84	316.1	87	535.1	81	837.9	81	(26.4)	-
1037	972	L9205a	CCC(O)(CC)CCCCO	1.0	291.15	958.0	74	-	-	-	-	-	-	-	-	(26.4)	-
1038	973	L9206a	CC(C)CC(CO)CCCO	1.0	293.15	941.6	74	-	-	-	-	-	-	-	-	(26.4)	-
1039	974	L9207a	CCCCC(O)CCCCO	1.0	293.15	937.0	74	-	-	-	-	-	-	-	-	(26.4)	-
1040	975	L9208a	CCCCCC(O)CCCO	1.0	293.15	929.5	74	-	-	-	-	571.1	81	837.9	81	(26.4)	-
1041	976	L9209a	CCC(O)CCCCCCO	1.0	298.15	944.0	74	-	-	-	-	-	-	-	-	(26.4)	-
1042	977	L9210a	OCCCCCCCCCO	1.0	323.0	-	-	110.0	84	319.6	87	558.1	81	837.9	81	(26.4)	-
1043	978	L0101a	CCC(O)(C(C)C)C(C)(C)C	1.0	293.15	862.4	74	-	-	-	-	464.1	81	680.4	81	(5.3)	-
1044	979	L0102a	CCC(C)(O)C(C)(C)C(C)C	1.0	293.15	874.2	74	-	-	-	-	475.1	81	680.4	81	(5.3)	-
1045	980	L0103a	CCC(C)(C)C(C)(O)C(C)C	1.0	293.15	874.5	74	-	-	-	-	474.1	81	680.4	81	(5.3)	-
1046	981	L0104a	CCC(C)C(C)(O)C(C)(C)C	1.0	293.15	858.4	74	-	-	-	-	465.1	81	680.4	81	(5.3)	-
1047	982	L0105a	CC(C)CC(C)(O)C(C)(C)C	1.0	293.15	839.3	74	-	-	-	-	513.5	81	680.4	81	(5.3)	-
1048	983	L0106a	CCC(C)(C)C(O)C(C)(C)C	1.0	293.15	854.9	74	-	-	-	-	463.1	81	680.4	81	(5.3)	-
1049	984	L0107a	CCC(C)(O)C(C)C(C)(C)C	1.0	293.15	862.3	74	-	-	-	-	468.1	81	680.4	81	(5.3)	-
1050	985	L0108a	CC(C)C(C)(O)CC(C)(C)C	1.0	293.15	837.8	74	-	-	-	-	475.0	81	680.4	81	(5.3)	-
1051	986	L0109a	CC(C)C(O)(C(C)C)C(C)C	1.0	298.15	859.1	74	-	-	-	-	467.6	81	680.4	81	(5.3)	-
1052	987	L0110a	CCCCC(C)(O)C(C)(C)C	1.0	293.15	848.7	74	-	-	-	-	459.1	81	680.4	81	(5.3)	-
1053	988	L0111a	CCC(O)(CC)CC(C)(C)C	1.0	293.15	842.8	74	-	-	-	-	460.0	81	680.4	81	(5.3)	-

Table S.2 – Reference experimental data (continued)

n_{sim}	n_{iso}	Code	Smiles	P [bar]	T [K]	ρ_{liq} [kg·m^{-3}]	Src	ΔH_{vap} [kJ·mol^{-1}]	Src	T_m [K]	Src	T_b [K]	Src	T$_c$ [K]	Src	ϵ	Src
1054	989	L0112a	CCC(C)(C)CC(C)(O)CC	1.0	293.15	854.3	74	-	-	-	-	468.8	81	680.4	81	(5.3)	-
1055	990	L0113a	CCCCCCCCCCO	1.0	298.15	-	-	80.9	84	280.1	87	503.4	81	687.3	81	7.9	87
1056	991	L0114a	CCCC(C)(O)CC(C)(C)C	1.0	293.15	833.0	74	-	-	-	-	454.1	81	680.4	81	(5.3)	-
1057	992	L0115a	CCCC(O)(C(C)C)C(C)C	1.0	293.15	853.7	74	-	-	-	-	465.1	81	680.4	81	(5.3)	-
1058	993	L0116a	CCC(C)(CC)C(O)C(C)C	1.0	293.15	860.6	74	-	-	-	-	460.0	81	680.4	81	(5.3)	-
1059	994	L0117a	CC(C)CCC(C)(O)C(C)C	1.0	293.15	839.8	74	-	-	-	-	463.0	81	680.4	81	(5.3)	-
1060	995	L0118a	CCC(CC)C(O)C(C)(C)C	1.0	293.15	833.9	74	-	-	-	-	460.1	81	680.4	81	(5.3)	-
1061	996	L0119a	CC(C)CCC(O)C(C)(C)C	1.0	293.15	823.6	74	-	-	-	-	463.0	81	680.4	81	(5.3)	-
1062	997	L0120a	CC(C)CC(C)(O)CC(C)C	1.0	294.15	823.0	74	-	-	-	-	455.1	81	680.4	81	(5.3)	-
1063	998	L0121a	CC(O)CC(C)CC(C)(C)C	1.0	293.15	826.5	74	-	-	-	-	466.0	81	680.4	81	(5.3)	-
1064	999	L0122a	CC(C)C(C)CCC(C)(C)O	1.0	291.15	833.0	74	-	-	-	-	466.1	81	680.4	81	(5.3)	-
1065	1000	L0123a	CCCC(O)(CCC)C(C)C	1.0	298.15	841.34	74	-	-	-	-	463.1	81	680.4	81	(5.3)	-
1066	1001	L0124a	CCCCC(O)(CC)C(C)C	1.0	293.15	845.5	74	-	-	-	-	466.1	81	680.4	81	(5.3)	-
1067	1002	L0125a	CCCCCC(C)(O)C(C)C	1.0	298.15	824.9	74	-	-	-	-	462.2	81	680.4	81	(5.3)	-
1068	1003	L0126a	CCCC(C)(O)CC(C)CC	1.0	301.15	825.8	74	-	-	-	-	464.0	81	680.4	81	(5.3)	-
1069	1004	L0127a	CCCC(C)CC(C)(O)CC	1.0	298.15	837.0	74	-	-	-	-	466.0	81	680.4	81	(5.3)	-
1070	1005	L0128a	CCCCC(C)(O)CC(C)C	1.0	293.15	823.2	74	-	-	-	-	464.0	81	680.4	81	(5.3)	-
1071	1006	L0129a	CCC(C)CCC(C)(O)CC	1.0	295.15	834.7	87	-	-	205.7	87	465.1	81	680.4	81	(5.3)	-
1072	1007	L0130a	CCCC(C)(O)CCC(C)C	1.0	273.15	842.1	74	-	-	-	-	465.1	81	680.4	81	(5.3)	-
1073	1008	L0131a	CCC(C)(O)CCCC(C)C	1.0	298.15	826.0	87	-	-	-	-	469.1	81	680.4	81	(5.3)	-
1074	1009	L0132a	CCCCC(O)CC(C)(C)C	1.0	293.15	821.2	74	-	-	-	-	463.1	81	680.4	81	(5.3)	-
1075	1010	L0133a	CCCCC(C)CC(C)(C)O	1.0	293.15	825.7	74	-	-	-	-	485.0	81	680.4	81	(5.3)	-
1076	1011	L0134a	CCC(C)CCCC(C)(C)O	1.0	293.15	827.3	74	-	-	-	-	485.0	81	680.4	81	(5.3)	-
1077	1012	L0135a	CC(C)CCCCC(C)(C)O	1.0	298.15	821.1	74	-	-	-	-	485.0	81	680.4	81	(5.3)	-
1078	1013	L0136a	CCC(O)C(C)C(CC)CC	1.0	290.15	865.0	74	-	-	-	-	466.0	81	680.4	81	(5.3)	-
1079	1014	L0137a	CC(C)CCC(CO)C(C)C	1.0	293.15	832.2	74	-	-	-	-	486.1	81	680.4	81	(5.3)	-
1080	1015	L0138a	CCCC(C)C(O)CC(C)C	1.0	298.15	821.5	74	-	-	-	-	464.0	81	680.4	81	(5.3)	-
1081	1016	L0139a	CC(C)CCCC(C)C(C)O	1.0	293.15	829.1	74	-	-	-	-	485.0	81	680.4	81	(5.3)	-
1082	1017	L0140a	CC(C)CCCC(O)C(C)C	1.0	293.15	815.2	74	-	-	-	-	467.1	81	680.4	81	(5.3)	-
1083	1018	L0141a	CC(C)CC(CO)CC(C)C	1.0	277.15	846.0	74	-	-	-	-	477.1	81	680.4	81	(5.3)	-
1084	1019	L0142a	CC(C)CCC(O)CC(C)C	1.0	293.15	814.0	74	-	-	-	-	475.1	81	680.4	81	(5.3)	-
1085	1020	L0143a	CCCC(O)(CCC)CCC	1.0	298.15	828.3	74	-	-	-	-	467.1	81	680.4	81	(5.3)	-
1086	1021	L0144a	CCCCC(O)(CC)CCC	1.0	298.15	826.8	74	-	-	-	-	466.0	81	680.4	81	(5.3)	-
1087	1022	L0145a	CCCCCC(O)(CC)CC	1.0	298.15	836.1	74	-	-	-	-	472.1	81	680.4	81	(5.3)	-
1088	1023	L0146a	CCCCCCC(C)(C)CO	1.0	293.15	830.0	74	-	-	-	-	481.1	81	680.4	81	7.9	87
1089	1024	L0147a	CCCCC(C)(O)CCCC	1.0	298.15	825.38	74	-	-	-	-	475.1	81	680.4	81	(5.3)	-
1090	1025	L0148a	CCCCCC(C)(O)CCC	1.0	298.15	827.1	74	-	-	-	-	482.0	81	680.4	81	(5.3)	-
1091	1026	L0149a	CCCCCCC(C)(O)CC	1.0	298.15	826.45	74	-	-	-	-	482.0	81	680.4	81	(5.3)	-
1092	1027	L0150a	CCCCCCCC(C)(C)O	1.0	298.15	807.8	74	-	-	-	-	487.0	81	680.4	81	(5.3)	-
1093	1028	L0151a	CCCCC(CCO)C(C)C	1.0	303.15	834.1	74	-	-	-	-	488.4	81	680.4	81	(5.3)	-
1094	1029	L0152a	CCCCC(C)C(O)CCC	1.0	300.15	826.0	74	-	-	-	-	482.0	81	680.4	81	(5.3)	-
1095	1030	L0153a	CCCCCCC(C)C(C)O	1.0	295.15	845.0	74	-	-	-	-	487.0	81	680.4	81	(5.3)	-
1096	1031	L0154a	CCCCCCC(O)C(C)C	1.0	298.15	824.5	74	-	-	-	-	481.6	81	680.4	81	(5.3)	-
1097	1032	L0155a	CCCC(CO)CC(C)CC	1.0	293.15	828.6	74	-	-	-	-	481.1	81	680.4	81	(5.3)	-
1098	1033	L0156a	CCC(O)CCC(CC)CC	1.0	297.65	839.6	74	-	-	-	-	472.0	81	680.4	81	(5.3)	-
1099	1034	L0157a	CCC(C)CCCC(C)CO	1.0	291.15	830.0	74	-	-	-	-	483.0	81	680.4	81	(5.3)	-
1100	1035	L0158a	CC(C)CCCC(C)CCO	1.0	298.15	832.0	87	-	-	-	-	485.1	81	680.4	81	(5.3)	-
1101	1036	L0159a	CC(C)CCCCC(C)CO	1.0	288.15	830.3	74	-	-	-	-	-	-	-	-	(5.3)	-

Table S.2 – Reference experimental data (continued)

n_{sim}	n_{iso}	Code	Smiles	P [bar]	T [K]	ρ_{liq} [kg·m^{-3}]	Src	ΔH_{vap} [kJ·mol^{-1}]	Src	T_m [K]	Src	T_b [K]	Src	T$_c$ [K]	Src	ϵ	Src
1102	1037	L0160a	CCCC(C)CCCC(C)O	1.0	293.15	833.2	74	-	-	-	-	487.0	81	680.4	81	(5.3)	-
1103	1038	L0161a	CCCCCC(O)CC(C)C	1.0	298.15	820.0	74	-	-	-	-	482.0	81	680.4	81	(5.3)	-
1104	1039	L0162a	CCCCC(O)CCC(C)C	1.0	293.15	821.5	74	-	-	-	-	482.0	81	680.4	81	(5.3)	-
1105	1040	L0163a	CCCCC(CO)CCCC	1.0	289.15	836.0	74	-	-	-	-	-	-	-	-	(5.3)	-
1106	1041	L0164a	CCCCCC(CO)CCC	1.0	293.15	832.2	74	-	-	-	-	491.1	81	680.4	81	(5.3)	-
1107	1042	L0165a	CCCCC(C)CCCCO	1.0	297.15	831.0	74	-	-	-	-	487.0	81	680.4	81	(5.3)	-
1108	1043	L0166a	CCCCCC(C)CCCO	1.0	300.15	826.0	74	-	-	-	-	489.1	81	680.4	81	(5.3)	-
1109	1044	L0167a	CCCCCCC(C)CCO	1.0	296.15	837.0	74	-	-	-	-	487.0	81	680.4	81	(5.3)	-
1110	1045	L0168a	CCC(C)CCCCCCO	1.0	298.15	828.2	74	-	-	-	-	487.0	81	680.4	81	(5.3)	-
1111	1046	L0169a	CCCCCCCC(C)CO	1.0	288.15	833.65	74	-	-	-	-	495.1	81	680.4	81	(5.3)	-
1112	1047	L0170a	CCCCCC(O)CCCC	1.0	298.15	820.6	74	-	-	281.9	87	474.1	81	680.4	81	3.2	87
1113	1048	L0171a	CCCCCCC(O)CCC	1.0	293.15	825.0	74	-	-	262.1	87	483.1	81	680.4	81	3.4	87
1114	1049	L0172a	CCCCCCCC(O)CC	1.0	298.15	822.9	74	-	-	265.6	87	483.0	81	680.4	81	4.0	87
1115	1050	L0201a	CC(C)CC(C)(O)CC(C)(C)O	1.0	288.15	910.24	74	-	-	-	-	-	-	-	-	(26.4)	-
1116	1051	L0202a	CCC(O)(CC)C(O)(CC)CC	1.0	298.15	943.5	74	-	-	-	-	503.1	81	855.5	81	(26.4)	-
1117	1052	L0203a	CCC(C)(O)CC(O)CC(C)C	1.0	285.15	911.8	74	-	-	-	-	-	-	-	-	(26.4)	-
1118	1053	L0204a	CC(C)CCCC(C)(O)CCO	1.0	293.15	916.5	74	-	-	-	-	-	-	-	-	(26.4)	-
1119	1054	L0205a	CC(CCO)CCCC(C)(C)O	1.0	293.15	926.0	74	-	-	-	-	538.1	81	855.5	81	(26.4)	-
1120	1055	L0206a	CC(CCO)CCC(O)C(C)C	1.0	293.15	948.1	74	-	-	-	-	-	-	-	-	(26.4)	-
1121	1056	L0207a	CCCC(CO)C(CO)CCC	1.0	293.15	936.2	74	-	-	-	-	-	-	-	-	(26.4)	-
1122	1057	L0208a	CCCCC(O)C(CO)CCC	1.0	301.15	915.5	74	-	-	-	-	-	-	-	-	(26.4)	-
1123	1058	L0209a	CCC(CO)CCC(CC)CO	1.0	298.15	930.7	74	-	-	-	-	-	-	-	-	(26.4)	-
1124	1059	L0210a	OCCCCCCCCCCO	1.0	353.15	883.0	74	-	-	345.6	87	572.1	81	855.5	81	(26.4)	-
1125	1060	D1201a	O=CO	1.0	298.15	1213.6	76	46.3	84	281.4	87	373.7	81	588.0	81	51.1	87
1126	1061	D2201a	CC(=O)O	1.01	298.15	1043.5	78	50.3	84	290.1	87	391.1	81	592.0	81	6.2	87
1127	1062	D3201a	CCC(=O)O	1.01	298.15	988.1	78	54.9	84	252.7	87	414.3	81	600.8	81	3.4	87
1128	1063	D4201a	CC(C)C(=O)O	1.0	298.15	943.89	76	56.3	84	227.2	87	427.6	87	-	-	2.6	87
1129	1064	D4202a	CCCC(=O)O	1.0	298.15	952.94	76	58.2	84	268.0	87	436.9	87	615.7	81	3.0	87
1130	1065	D5201a	CC(C)(C)C(=O)O	1.0	309.82	907.45	76	-	-	309.1	87	436.9	81	631.0	81	(2.7)	-
1131	1065	D5201b	CC(C)(C)C(=O)O	1.0	320.0	-	-	57.6	84	309.1	87	436.9	81	631.0	81	(2.7)	-
1132	1066	D5202a	CCC(C)C(=O)O	1.0	298.15	934.7	76	-	-	-	-	450.1	87	631.0	81	(2.7)	-
1133	1067	D5203a	CC(C)CC(=O)O	1.0	298.15	924.06	76	61.2	84	243.6	87	448.2	81	629.1	81	(2.7)	-
1134	1068	D5204a	CCCCC(=O)O	1.0	298.15	934.4	76	63.0	84	239.5	87	458.6	81	639.2	81	2.7	87
1135	1069	D6201a	CCC(C)(C)C(=O)O	1.0	293.15	927.47	76	-	-	258.1	87	459.1	81	654.6	81	(2.7)	-
1136	1069	D6201b	CCC(C)(C)C(=O)O	1.0	370.0	-	-	59.4	84	258.1	87	459.1	81	654.6	81	(2.7)	-
1137	1070	D6202a	CC(C)(C)CC(=O)O	1.0	298.05	908.0	76	64.0	84	279.1	87	457.1	87	654.6	81	2.9	87
1138	1071	D6203a	CC(C)C(C)C(=O)O	1.0	293.15	927.45	76	-	-	-	-	464.9	81	654.6	81	(2.7)	-
1139	1072	D6204a	CCC(CC)C(=O)O	1.0	298.15	923.5	76	-	-	241.3	87	466.1	87	654.6	81	2.7	87
1140	1073	D6205a	CCCC(C)C(=O)O	1.0	298.15	918.22	76	-	-	-	-	-	-	654.6	81	(2.7)	-
1141	1074	D6206a	CCC(C)CC(=O)O	1.0	298.15	923.0	76	-	-	-	-	-	-	654.6	81	(2.7)	-
1142	1075	D6207a	CC(C)CCC(=O)O	1.0	298.15	917.0	76	-	-	240.2	87	473.6	81	654.6	81	(2.7)	-
1143	1076	D6208a	CCCCCC(=O)O	1.0	298.15	922.88	76	69.2	84	269.1	87	478.9	81	660.2	81	2.6	87
1144	1077	D7201a	CCCC(C)(C)C(=O)O	1.0	293.15	885.6	76	-	-	-	-	-	-	676.3	81	(3.0)	-
1145	1078	D7202a	CCC(C)(C)CC(=O)O	1.0	298.15	934.8	76	-	-	-	-	-	-	-	-	(3.0)	-
1146	1079	D7203a	CC(C)CC(C)C(=O)O	1.0	298.15	910.0	76	-	-	-	-	-	-	-	-	(3.0)	-
1147	1080	D7204a	CC(C)C(C)CC(=O)O	1.0	298.15	923.9	76	-	-	-	-	-	-	-	-	(3.0)	-
1148	1081	D7205a	CCCC(CC)C(=O)O	1.0	298.15	909.8	76	-	-	-	-	-	-	676.3	81	(3.0)	-
1149	1082	D7206a	CCCCC(C)C(=O)O	1.0	298.15	909.0	76	-	-	-	-	482.6	81	-	-	(3.0)	-

Table S.2 – Reference experimental data (continued)

n_{sim}	n_{iso}	Code	Smiles	P [bar]	T [K]	ρ_{liq} [kg·m^{-3}]	Src	ΔH_{vap} [kJ·mol^{-1}]	Src	T_m [K]	Src	T_b [K]	Src	T$_c$ [K]	Src	ϵ	Src
1150	1083	D7207a	CCCC(C)CC(=O)O	1.0	293.15	918.7	76	-	-	-	-	486.1	81	676.3	81	(3.0)	-
1151	1084	D7208a	CC(C)CCCC(=O)O	1.0	293.15	916.3	76	-	-	-	-	489.1	81	676.3	81	(3.0)	-
1152	1085	D7209a	CCCCCCC(=O)O	1.0	298.15	913.5	78	72.9	84	266.0	87	496.1	81	677.3	81	3.0	87
1153	1086	D8201a	CC(CC(=O)O)C(C)(C)C	1.0	298.15	919.9	76	-	-	-	-	-	-	-	-	(2.8)	-
1154	1087	D8202a	CC(C)C(C)C(C)C(=O)O	1.0	293.15	929.0	76	-	-	-	-	-	-	-	-	(2.8)	-
1155	1088	D8203a	CCCC(C)(C)CC(=O)O	1.0	298.15	900.9	76	-	-	-	-	-	-	-	-	(2.8)	-
1156	1089	D8204a	CC(C)CC(C)CC(=O)O	1.0	298.15	902.0	76	-	-	-	-	-	-	-	-	(2.8)	-
1157	1090	D8205a	CCCC(CCC)C(=O)O	1.0	298.15	904.0	87	74.8	84	-	-	494.1	81	696.5	81	(2.8)	-
1158	1091	D8206a	CCCCC(CC)C(=O)O	1.0	298.15	903.03	76	75.6	84	-	-	501.1	81	673.2	81	2.6	87
1159	1092	D8207a	CCC(C)CCCC(=O)O	1.0	293.15	910.5	76	-	-	-	-	-	-	-	-	(2.8)	-
1160	1093	D8208a	CCCCCCCC(=O)O	1.01	298.15	906.02	78	81.2	84	289.7	87	513.0	81	694.3	81	2.9	87
1161	1094	D9201a	CCCCC(C)(C)CC(=O)O	1.0	298.15	910.2	76	-	-	-	-	-	-	-	-	(2.3)	-
1162	1095	D9202a	CCCCCC(CC)C(=O)O	1.0	298.15	893.5	76	-	-	-	-	456.9	81	715.3	81	2.0	87
1163	1096	D9203a	CCCCCCC(C)C(=O)O	1.0	277.15	909.7	76	-	-	-	-	456.9	81	715.3	81	2.4	87
1164	1097	D9204a	CCCCCC(C)CC(=O)O	1.0	296.15	899.0	76	-	-	-	-	456.9	81	715.3	81	(2.3)	-
1165	1098	D9205a	CCCCCCCCC(=O)O	1.0	298.15	901.63	76	-	-	285.5	87	528.8	81	710.7	81	2.5	87
1166	1098	D9205b	CCCCCCCCC(=O)O	1.0	304.0	-	-	85.3	84	285.5	87	528.8	81	710.7	81	2.5	87
1167	1099	D0201a	CCCCCCC(C)(C)C(=O)O	1.0	293.15	902.2	76	-	-	-	-	473.7	81	733.0	81	2.8	87
1168	1100	D0202a	CCCCCC(C)(C)CC(=O)O	1.0	298.15	912.9	76	-	-	-	-	-	-	-	-	(2.8)	-
1169	1101	D0203a	CC(C)CCCC(C)CC(=O)O	1.0	291.15	897.0	76	-	-	-	-	-	-	-	-	(2.8)	-
1170	1102	D0204a	CCCCC(CCCC)C(=O)O	1.0	291.55	897.8	76	-	-	-	-	-	-	-	-	(2.8)	-
1171	1103	D0205a	CCCCCCC(CC)C(=O)O	1.0	293.15	848.1	76	-	-	-	-	-	-	733.0	81	(2.8)	-
1172	1104	D0206a	CCCCCCCC(C)C(=O)O	1.0	298.15	893.0	76	-	-	-	-	-	-	733.0	81	(2.8)	-
1173	1105	D0207a	CCCCCCC(C)CC(=O)O	1.0	293.15	898.3	76	-	-	-	-	473.7	81	733.0	81	(2.8)	-
1174	1106	D0208a	CCCCC(CC)CCC(=O)O	1.0	293.15	908.6	76	-	-	-	-	-	-	733.0	81	(2.8)	-
1175	1107	D0209a	CCCCCCCCCC(=O)O	1.0	313.15	881.7	76	88.6	84	304.5	87	543.1	81	722.1	81	(2.8)	-
1176	1108	N1101a	CN	1.0	266.85	694.21	76	26.11	81	179.7	87	266.8	81	430.1	81	16.7	87
1177	1108	N1101b	CN	3.52	298.15	655.0	81	-	-	179.7	87	266.8	81	430.1	81	16.7	87
1178	1109	N2101a	CCN	1.0	288.15	688.56	76	27.35	81	192.2	87	289.7	81	456.1	81	8.7	87
1179	1110	N2102a	CNC	1.0	282.82	667.93	76	27.2	84	180.2	87	280.0	81	437.2	81	(3.1)	-
1180	1110	N2102b	CNC	1.96	298.15	649.96	81	-	-	180.2	87	280.0	81	437.2	81	(3.1)	-
1181	1111	N2201a	NCCN	0.02	298.15	892.82	81	45.0	84	284.3	87	390.4	81	593.0	81	13.8	87
1182	1112	N3101a	CC(C)N	1.01	298.15	683.94	78	28.4	84	178.0	87	305.6	81	471.9	81	5.6	87
1183	1113	N3102a	CCCN	1.01	298.15	712.76	78	31.3	84	188.4	87	321.6	81	496.9	81	5.1	87
1184	1114	N3103a	CN(C)C	1.0	273.15	655.7	76	22.91	81	156.1	87	276.0	81	433.2	81	2.4	87
1185	1114	N3103b	CN(C)C	2.33	298.15	628.94	81	-	-	156.1	87	276.0	81	433.2	81	2.4	87
1186	1115	N3201a	CNCCN	1.0	298.15	841.0	87	-	-	-	-	388.1	81	-	-	(13.8)	-
1187	1116	N3202a	CC(N)CN	1.0	298.0	-	-	44.2	84	-	-	392.4	81	585.0	81	(13.8)	-
1188	1117	N3203a	NCCCN	1.0	298.15	884.0	87	50.2	84	262.2	87	412.9	81	608.0	81	(13.8)	-
1189	1118	N4101a	CC(C)(C)N	1.0	298.15	690.07	76	29.6	84	206.2	87	317.6	81	483.9	81	(4.7)	-
1190	1119	N4102a	CCC(C)N	1.01	298.15	717.81	78	32.6	84	-	-	336.1	81	514.3	81	(4.7)	-
1191	1120	N4103a	CC(C)CN	1.0	298.15	729.14	76	33.8	84	187.2	87	340.9	81	522.4	81	(4.7)	-
1192	1121	N4104a	CCCCN	1.0	298.15	736.83	76	35.6	84	224.2	87	350.6	81	531.9	81	4.7	87
1193	1122	N4105a	CNC(C)C	1.0	288.15	740.0	76	30.7	84	-	-	323.6	81	482.4	81	(3.7)	-
1194	1123	N4106a	CCNCC	1.0	298.15	701.2	76	31.2	84	223.2	87	328.6	81	496.6	81	3.7	87
1195	1124	N4107a	CCCNC	0.2	298.15	711.93	81	-	-	-	-	336.1	81	482.4	81	(3.7)	-
1196	1125	N4108a	CCN(C)C	1.0	298.15	669.4	76	-	-	133.2	87	310.1	81	500.0	81	(2.4)	-
1197	1126	N4201a	CN(C)CCN	1.0	298.15	803.0	87	-	-	-	-	378.6	81	-	-	(13.8)	-

Table S.2 – Reference experimental data (continued)

n_{sim}	n_{iso}	Code	Smiles	P [bar]	T [K]	ρ_{liq} [kg·m^{-3}]	Src	ΔH_{vap} [kJ·mol^{-1}]	Src	T_m [K]	Src	T_b [K]	Src	T$_c$ [K]	Src	ϵ	Src
1198	1127	N4202a	CNCCNC	1.0	288.15	828.0	87	-	-	-	-	393.1	81	-	-	(13.8)	-
1199	1128	N4203a	CC(C)(N)CN	1.0	298.15	841.0	87	45.8	84	-	-	396.1	81	-	-	(13.8)	-
1200	1129	N4204a	CCNCCN	1.0	298.15	837.0	87	-	-	-	-	402.1	81	-	-	(13.8)	-
1201	1130	N4205a	CNCCCN	1.0	298.0	-	-	53.1	84	-	-	413.1	81	-	-	(13.8)	-
1202	1131	N4206a	CCC(N)CN	1.0	298.0	-	-	46.9	84	-	-	-	-	-	-	(13.8)	-
1203	1132	N4207a	NCCCCN	1.0	298.15	877.0	87	55.2	84	295.1	87	431.6	81	-	-	(13.8)	-
1204	1133	N5101a	CCC(C)(C)N	0.11	298.15	727.59	81	-	-	168.2	87	350.1	81	557.7	81	(4.3)	-
1205	1134	N5102a	CC(C)(C)CN	0.1	298.15	728.07	81	-	-	-	-	350.1	81	557.7	81	(4.3)	-
1206	1135	N5103a	CC(C)C(C)N	0.08	298.15	753.0	81	-	-	223.2	87	357.1	81	557.7	81	(4.3)	-
1207	1136	N5104a	CCC(N)CC	1.0	291.35	711.3	76	-	-	-	-	362.1	81	557.7	81	(4.3)	-
1208	1137	N5105a	CCC(C)CN	1.0	298.15	750.5	76	-	-	-	-	368.6	81	557.7	81	(4.3)	-
1209	1138	N5106a	CC(C)CCN	1.0	298.15	744.3	76	-	-	-	-	370.1	81	557.7	81	(4.3)	-
1210	1139	N5107a	CCCCCN	0.04	298.15	751.0	81	40.1	84	222.2	87	377.6	81	557.7	81	4.3	87
1211	1140	N5108a	CNC(C)(C)C	1.0	298.15	727.0	76	32.3	84	-	-	350.1	81	517.7	81	(3.1)	-
1212	1141	N5109a	CCC(C)NC	0.1	298.15	719.96	81	-	-	-	-	351.6	81	517.7	81	(3.1)	-
1213	1142	N5110a	CNCC(C)C	0.12	298.15	729.96	81	-	-	-	-	346.1	81	517.7	81	(3.1)	-
1214	1143	N5111a	CCNC(C)C	1.0	298.0	-	-	33.1	84	-	-	349.1	81	517.7	81	(3.1)	-
1215	1144	N5112a	CCCNCC	0.11	298.15	726.66	81	-	-	-	-	353.4	81	517.7	81	(3.1)	-
1216	1145	N5113a	CCCCNC	0.05	298.15	728.09	81	-	-	-	-	364.2	81	517.7	81	(3.1)	-
1217	1146	N5114a	CC(C)N(C)C	1.0	298.15	710.6	76	31.9	84	-	-	339.2	81	517.7	81	(2.4)	-
1218	1147	N5115a	CCN(C)CC	1.0	298.15	701.55	76	31.8	84	77.2	87	339.1	81	517.7	81	(2.4)	-
1219	1148	N5116a	CCCN(C)C	0.21	298.15	695.57	81	-	-	-	-	339.1	81	517.7	81	(2.4)	-
1220	1149	N5201a	CN(C)CN(C)C	1.0	291.15	749.1	87	-	-	-	-	356.1	81	-	-	(13.8)	-
1221	1149	N5201b	CN(C)CN(C)C	1.0	298.0	-	-	33.1	84	-	-	356.1	81	-	-	(13.8)	-
1222	1150	N5202a	CN(C)CCCN	1.0	293.15	827.2	87	42.2	84	-	-	406.6	81	-	-	(13.8)	-
1223	1151	N5203a	CCC(N)CCN	1.0	298.0	-	-	54.9	84	-	-	437.1	81	-	-	(13.8)	-
1224	1152	N5204a	NCCCCCN	1.0	298.15	873.0	87	58.7	84	284.9	87	452.1	81	-	-	(13.8)	-
1225	1153	N6101a	CC(N)C(C)(C)C	0.04	298.15	760.06	81	-	-	253.2	87	375.1	81	589.1	81	(4.1)	-
1226	1154	N6102a	CC(C)CC(C)N	0.02	298.15	745.7	81	-	-	-	-	381.6	81	589.1	81	(4.1)	-
1227	1155	N6103a	CCCCCCN	1.0	298.15	762.99	76	45.0	84	252.2	87	404.6	81	589.1	81	4.1	87
1228	1156	N6104a	CCNC(C)(C)C	1.0	298.15	716.1	76	-	-	-	-	351.1	81	549.1	81	(2.9)	-
1229	1157	N6105a	CC(C)NC(C)C	1.0	298.15	712.27	76	34.5	84	212.2	87	357.1	81	523.1	81	(2.9)	-
1230	1158	N6106a	CCNC(C)CC	0.04	298.15	729.77	81	-	-	-	-	371.1	81	549.1	81	(2.9)	-
1231	1159	N6107a	CCCNC(C)C	0.04	298.15	723.16	81	37.3	84	-	-	371.4	81	549.1	81	(2.9)	-
1232	1160	N6108a	CNCCC(C)C	1.0	295.15	739.0	76	-	-	-	-	-	-	-	-	(2.9)	-
1233	1161	N6109a	CCCNCCC	1.0	298.15	734.9	76	40.0	84	210.2	87	382.0	81	550.0	81	2.9	87
1234	1162	N6110a	CCCCNCC	0.02	298.15	735.42	81	40.2	84	-	-	381.1	81	549.1	81	(2.9)	-
1235	1163	N6111a	CCCCCNC	0.02	298.15	743.01	81	-	-	-	-	390.6	81	549.1	81	(2.9)	-
1236	1164	N6112a	CN(C)C(C)(C)C	1.0	298.15	737.6	76	34.8	84	-	-	363.1	81	549.1	81	(2.4)	-
1237	1165	N6113a	CCC(C)N(C)C	0.05	298.15	733.93	81	-	-	-	-	367.1	81	549.1	81	(2.4)	-
1238	1166	N6114a	CCN(C)C(C)C	0.06	298.15	721.46	81	-	-	-	-	364.6	81	549.1	81	(2.4)	-
1239	1167	N6115a	CC(C)CN(C)C	0.1	298.15	719.96	81	-	-	-	-	354.1	81	549.1	81	(2.4)	-
1240	1168	N6116a	CCN(CC)CC	1.01	298.15	723.01	78	34.91	81	158.4	87	361.9	81	535.1	81	2.4	87
1241	1169	N6117a	CCCN(C)CC	0.06	298.15	718.0	81	-	-	-	-	364.6	81	549.1	81	(2.4)	-
1242	1170	N6118a	CCCCN(C)C	0.06	298.15	716.01	81	-	-	-	-	367.1	81	549.1	81	(2.4)	-
1243	1171	N6201a	CN(C)CCN(C)C	1.0	298.15	770.0	87	41.4	84	215.2	87	394.1	81	-	-	(13.8)	-
1244	1172	N6202a	CCNCCN(C)C	1.0	298.15	738.0	87	-	-	-	-	407.6	81	-	-	(13.8)	-
1245	1173	N6203a	CCN(CC)CCN	1.0	293.15	828.0	87	-	-	-	-	417.1	81	-	-	(13.8)	-

Table S.2 – Reference experimental data (continued)

n_{sim}	n_{iso}	Code	Smiles	P [bar]	T [K]	ρ_{liq} [kg·m^{-3}]	Src	ΔH_{vap} [kJ·mol^{-1}]	Src	T_m [K]	Src	T_b [K]	Src	T_c [K]	Src	ϵ	Src
1246	1174	N6204a	CCNCCNCC	1.0	293.15	828.0	87	-	-	-	-	419.1	81	-	-	(13.8)	-
1247	1175	N6205a	CC(CN)CCCN	1.0	298.0	-	-	60.9	84	-	-	466.1	81	-	-	(13.8)	-
1248	1176	N6206a	NCCCCCCN	1.0	298.0	-	-	63.1	84	311.9	87	475.0	81	-	-	(13.8)	-
1249	1177	N7101a	CCC(N)C(C)(C)C	1.0	298.15	761.5	76	-	-	-	-	-	-	-	-	(3.8)	-
1250	1178	N7102a	CC(C)CC(C)(C)N	0.01	298.15	768.2	81	-	-	-	-	395.1	81	617.7	81	(3.8)	-
1251	1179	N7103a	CC(C)C(N)C(C)C	1.0	293.15	788.2	76	-	-	-	-	-	-	-	-	(3.8)	-
1252	1180	N7104a	CCC(C)CC(C)N	0.01	306.57	755.49	81	-	-	-	-	405.6	81	617.7	81	(3.8)	-
1253	1181	N7105a	CCCC(N)CCC	0.01	312.17	752.73	81	-	-	-	-	412.6	81	617.7	81	(3.8)	-
1254	1182	N7106a	CCCC(C)CCN	0.01	319.77	756.11	81	-	-	-	-	422.1	81	617.7	81	(3.8)	-
1255	1183	N7107a	CCCCCC(C)N	1.0	293.15	762.9	76	-	-	-	-	415.1	81	617.7	81	(3.8)	-
1256	1184	N7108a	CCCCCCCN	1.0	298.15	771.31	76	49.9	84	250.2	87	430.1	81	617.7	81	3.8	87
1257	1185	N7109a	CCCCNC(C)C	1.0	298.0	-	-	42.1	84	-	-	-	-	-	-	(3.1)	-
1258	1186	N7110a	CCCCCCNC	1.0	293.15	778.7	76	-	-	-	-	415.1	81	577.7	81	(3.1)	-
1259	1187	N7111a	CCN(C)C(C)(C)C	0.02	298.15	744.51	81	-	-	-	-	383.1	81	577.7	81	(2.4)	-
1260	1188	N7112a	CC(C)N(C)C(C)C	0.02	298.15	749.49	81	-	-	-	-	385.1	81	577.7	81	(2.4)	-
1261	1189	N7113a	CCN(C)CC(C)C	0.03	298.15	729.47	81	-	-	-	-	380.1	81	577.7	81	(2.4)	-
1262	1190	N7114a	CCCN(CC)CC	0.02	298.15	737.98	81	-	-	-	-	385.1	81	577.7	81	(2.4)	-
1263	1191	N7201a	CN(C)CCCN(C)C	1.0	298.0	-	-	45.3	84	-	-	417.1	81	-	-	(13.8)	-
1264	1192	N7202a	CCN(CC)CCCN	1.0	293.15	-	-	52.4	84	-	-	441.6	81	-	-	(13.8)	-
1265	1193	N7203a	NCCCCCCCN	1.0	298.0	-	-	67.1	84	298.4	87	497.1	81	-	-	(13.8)	-
1266	1194	N8101a	CCCCCCC(C)N	1.0	293.15	772.0	78	-	-	-	-	438.1	81	643.9	81	(3.6)	-
1267	1195	N8102a	CCCCCCCCN	1.01	298.15	779.95	78	55.1	84	273.1	87	452.8	81	643.9	81	3.6	87
1268	1196	N8103a	CCC(C)NC(C)CC	1.0	298.15	749.02	76	-	-	-	-	407.1	81	603.9	81	(2.8)	-
1269	1196	N8103b	CCC(C)NC(C)CC	0.01	307.77	-	-	46.27	81	-	-	407.1	81	603.9	81	(2.8)	-
1270	1197	N8104a	CC(C)CNCC(C)C	1.0	298.15	740.74	76	-	-	199.7	87	412.2	81	603.9	81	(2.8)	-
1271	1198	N8105a	CCCCNCCCC	1.0	298.15	755.72	76	49.4	84	211.3	87	432.0	81	602.3	81	2.8	87
1272	1199	N8106a	CCCCCCCNC	1.0	273.15	771.2	76	-	-	-	-	438.1	81	603.9	81	(2.8)	-
1273	1200	N8107a	CCN(C(C)C)C(C)C	1.0	313.15	749.0	76	-	-	-	-	399.6	81	603.9	81	(2.4)	-
1274	1201	N8108a	CCCCN(CC)CC	1.0	293.15	742.0	76	-	-	-	-	409.1	81	603.9	81	(2.4)	-
1275	1202	N8109a	CCCCN(C)CCC	1.0	298.15	742.0	76	-	-	-	-	-	-	-	-	(2.4)	-
1276	1203	N8201a	CN(C)CCCCN(C)C	1.0	288.15	794.2	87	-	-	-	-	441.1	81	-	-	(13.8)	-
1277	1204	N8202a	CC(C)(N)CCC(C)(C)N	1.0	288.15	848.5	87	-	-	-	-	457.1	81	-	-	(13.8)	-
1278	1205	N9101a	CC(C)CC(N)CC(C)C	1.0	293.15	771.9	76	-	-	-	-	-	-	-	-	(3.4)	-
1279	1206	N9102a	CCCCC(CN)CCC	1.0	298.15	789.2	76	-	-	-	-	-	-	-	-	(3.4)	-
1280	1207	N9103a	CCCCCCCCCN	1.0	293.15	788.6	87	-	-	272.1	87	475.4	81	668.2	81	3.4	87
1281	1208	N9104a	CCCCCCCNCC	1.0	298.15	767.52	76	-	-	-	-	453.9	81	628.2	81	(3.1)	-
1282	1209	N9105a	CCCCCCCCNC	1.0	293.15	782.4	76	-	-	-	-	459.9	81	628.2	81	(3.1)	-
1283	1210	N9106a	CCCN(CCC)CCC	1.0	298.15	752.88	76	46.2	84	172.7	87	429.6	81	628.2	81	2.4	87
1284	1211	N9107a	CCCCCN(CC)CC	1.0	293.15	766.3	76	-	-	-	-	429.1	81	628.2	81	(2.4)	-
1285	1212	N9108a	CCCCN(C)CCCC	1.0	293.15	759.3	76	-	-	-	-	432.8	81	628.2	81	(2.4)	-
1286	1213	N9109a	CCCCCCCN(C)C	1.0	293.15	758.0	76	-	-	-	-	445.1	81	628.2	81	(2.4)	-
1287	1214	N9201a	CCN(CC)CN(CC)CC	1.0	293.15	800.0	87	-	-	-	-	438.9	81	-	-	(13.8)	-
1288	1215	N9202a	CCN(CC)CCCC(C)N	1.0	293.15	814.0	87	-	-	-	-	474.1	81	-	-	(13.8)	-
1289	1216	N9203a	NCCCCCCCCCN	1.0	298.0	-	-	75.5	84	-	-	531.6	81	-	-	(13.8)	-
1290	1217	N0101a	CCCCCCCCCCN	1.0	298.15	789.0	76	64.9	84	288.1	87	493.6	81	690.9	81	3.3	87
1291	1218	N0102a	CC(C)CCNCCC(C)C	1.0	298.15	766.88	76	-	-	229.2	87	461.1	81	650.9	81	(3.1)	-
1292	1219	N0103a	CCCCCNCCCCC	1.0	298.15	773.25	76	61.2	84	-	-	476.1	81	650.9	81	(3.1)	-
1293	1220	N0104a	CCCCCCCCNCC	1.0	298.15	774.3	76	-	-	-	-	475.1	81	650.9	81	(3.1)	-

Table S.2 – Reference experimental data (continued)

n_{sim}	n_{iso}	Code	Smiles	P [bar]	T [K]	ρ_{liq} [kg·m^{-3}]	Src	ΔH_{vap} [kJ·mol^{-1}]	Src	T_m [K]	Src	T_b [K]	Src	T$_c$ [K]	Src	ϵ	Src
1294	1221	N0105a	CCCCCCCCCCNC	1.0	292.15	784.0	76	-	-	-	-	480.1	81	650.9	81	(3.1)	-
1295	1222	N0106a	CCCCC(CC)CN(C)C	1.0	293.15	768.6	76	-	-	-	-	-	-	-	-	(2.4)	-
1296	1223	N0107a	CCCCCCCCCN(C)C	1.0	293.15	768.7	76	-	-	-	-	464.1	81	650.9	81	(2.4)	-
1297	1224	N0201a	NCCCCCCCCCCCN	1.0	345.0	-	-	73.6	84	332.9	87	485.0	81	-	-	(13.8)	-
1298	1225	M2201a	CC(N)=O	0.004	354.2	1001.97	81	-	-	353.3	87	494.3	81	761.0	81	67.6	87
1299	1226	M3201a	CNC(C)=O	1.01	308.15	949.7	78	-	-	303.8	87	478.1	81	718.0	81	179.0	87
1300	1227	M3202a	CCC(N)=O	1.01	355.65	963.2	78	-	-	353.1	87	486.1	81	-	-	(43.9)	-
1301	1227	M3202b	CCC(N)=O	1.0	390.0	-	-	63.9	84	353.1	87	486.1	81	-	-	(43.9)	-
1302	1228	M4201a	CC(=O)N(C)C	1.01	298.15	936.7	78	50.66	78	254.2	87	439.2	81	658.0	81	40.2	79
1303	1229	M4202a	CCC(=O)NC	1.0	298.15	930.5	87	66.6	84	242.2	87	421.1	81	-	-	177.1	86
1304	1230	M4203a	CCNC(C)=O	1.0	277.15	942.0	87	-	-	-	-	478.1	81	-	-	129.0	86
1305	1230	M4203b	CCNC(C)=O	1.0	298.0	-	-	64.9	84	-	-	478.1	81	-	-	129.0	86
1306	1231	M4204a	CCCC(N)=O	1.0	393.15	885.0	87	-	-	389.1	87	489.1	81	-	-	(43.9)	-
1307	1232	M5201a	CCC(=O)N(C)C	1.01	298.15	920.32	78	-	-	228.2	87	444.6	81	-	-	(35.3)	-
1308	1233	M5202a	CNC(=O)C(C)C	1.0	298.0	-	-	67.1	84	-	-	-	-	-	-	(141.4)	-
1309	1234	M5203a	CC(=O)NC(C)C	1.0	298.0	-	-	66.4	84	-	-	-	-	-	-	(141.4)	-
1310	1235	M5204a	CCCNC(C)=O	1.0	298.0	-	-	69.8	84	-	-	-	-	-	-	(141.4)	-
1311	1236	M5205a	CC(C)(C)C(N)=O	1.0	426.97	-	-	36.91	81	425.4	78	427.0	81	-	-	20.1	87
1312	1237	M6201a	CCCC(=O)N(C)C	1.0	298.15	906.4	87	-	-	233.2	87	459.1	81	-	-	(30.4)	-
1313	1238	M6202a	CCN(CC)C(C)=O	1.0	290.15	913.0	87	53.7	78	-	-	458.6	81	700.0	81	30.4	86
1314	1239	M6203a	CCCCNC(C)=O	1.0	298.15	896.0	87	76.1	84	-	-	502.1	81	700.0	81	104.0	87
1315	1240	M7201a	CCC(=O)N(CC)CC	1.0	293.15	897.2	87	-	-	-	-	464.1	81	-	-	(35.3)	-
1316	1241	M7202a	CCCCC(=O)N(C)C	1.0	298.15	896.2	87	-	-	222.2	87	458.8	81	-	-	(35.3)	-
1317	1242	M8201a	CCCN(CCC)C(C)=O	1.0	290.15	899.2	87	-	-	-	-	482.6	81	700.0	81	(35.3)	-
1318	1243	M9201a	CCN(CC)C(=O)CC(C)C	1.0	293.15	876.4	87	-	-	-	-	484.1	81	-	-	(35.3)	-
1319	1244	S1201a	ClCBr	1.01	298.15	1924.88	78	-	-	185.2	87	341.2	81	557.0	81	(8.9)	-
1320	1245	S1202a	FCCl	1.0	260.17	1282.14	77	21.9	81	138.1	87	264.1	81	411.5	81	(8.9)	-
1321	1246	S1301a	FC(F)Br	1.0	298.15	1775.48	77	-	-	128.2	87	257.6	87	412.0	81	(4.8)	-
1322	1246	S1301b	FC(F)Br	1.0	244.0	-	-	24.0	84	128.2	87	257.6	87	412.0	81	(4.8)	-
1323	1247	S1302a	FC(F)Cl	1.0	233.15	1408.0	77	20.2	84	115.7	87	232.3	81	369.3	81	(4.8)	-
1324	1247	S1302b	FC(F)Cl	10.77	298.15	1193.01	81	-	-	115.7	87	232.3	81	369.3	81	(4.8)	-
1325	1248	S1303a	FC(Cl)Cl	1.83	298.15	1366.98	81	-	-	142.8	87	282.1	81	451.6	81	(4.8)	-
1326	1248	S1303b	FC(Cl)Cl	1.0	267.0	-	-	26.1	84	142.8	87	282.1	81	451.6	81	(4.8)	-
1327	1249	S1304a	FC(I)I	1.0	294.65	3196.9	77	-	-	-	-	373.1	81	-	-	(4.8)	-
1328	1250	S1305a	ClC(Cl)Br	1.0	298.15	1983.47	77	-	-	217.2	87	363.1	81	-	-	(4.8)	-
1329	1251	S1401a	ClC(Cl)(Cl)Br	1.01	298.15	2002.14	78	36.14	81	267.6	87	378.1	81	606.0	81	2.4	87
1330	1252	S1402a	FC(F)(F)Br	1.0	215.15	1989.9	77	17.5	81	98.8	87	215.3	81	340.1	81	3.7	87
1331	1252	S1402b	FC(F)(F)Br	16.64	298.15	1536.09	81	-	-	98.8	87	215.3	81	340.1	81	3.7	87
1332	1253	S1403a	FC(F)(Br)Br	1.0	298.15	2251.23	77	24.96	81	163.1	87	295.9	81	478.0	81	2.9	87
1333	1254	S1404a	FC(F)(F)Cl	1.0	190.0	1527.8	77	15.39	81	92.0	87	191.7	81	301.8	81	3.0	87
1334	1254	S1404b	FC(F)(F)Cl	36.66	298.15	840.94	81	-	-	92.0	87	191.7	81	301.8	81	3.0	87
1335	1255	S1405a	FC(F)(Cl)Cl	1.0	243.14	1488.0	77	20.1	84	116.1	87	243.4	81	384.9	81	3.5	87
1336	1255	S1405b	FC(F)(Cl)Cl	6.74	298.15	1307.01	81	-	-	116.1	87	243.4	81	384.9	81	3.5	87
1337	1256	S1406a	FC(Cl)(Cl)Cl	1.06	298.15	1477.01	81	24.85	81	162.7	87	297.0	81	471.2	81	3.0	87
1338	1257	S1407a	FC(F)(F)I	20.0	298.15	2046.84	78	-	-	-	-	250.7	81	-	-	(2.2)	-
1339	1258	S1408a	FC(F)(Cl)Br	1.0	268.71	1899.64	77	23.1	84	113.7	87	269.1	81	426.1	81	3.9	87
1340	1258	S1408b	FC(F)(Cl)Br	2.6	298.15	1809.99	81	-	-	113.7	87	269.1	81	426.1	81	3.9	87
1341	1259	S2201a	CC(Cl)Br	1.0	283.15	1667.0	87	-	-	-	-	356.1	81	-	-	(7.4)	-

Table S.2 – Reference experimental data (continued)

n_{sim}	n_{iso}	Code	Smiles	P [bar]	T [K]	ρ_{liq} [kg·m^{-3}]	Src	ΔH_{vap} [kJ·mol^{-1}]	Src	T_m [K]	Src	T_b [K]	Src	T$_c$ [K]	Src	ϵ	Src
1342	1260	S2202a	ClCCBr	1.01	298.15	1727.01	78	-	-	256.4	87	380.1	81	-	-	7.4	87
1343	1260	S2202b	ClCCBr	1.0	308.0	-	-	37.6	84	256.4	87	380.1	81	-	-	7.4	87
1344	1261	S2203a	FCCBr	1.0	298.15	1704.4	87	-	-	-	-	344.6	81	-	-	(7.4)	-
1345	1262	S2204a	FCCCl	0.33	298.15	1167.53	81	-	-	-	-	326.1	81	497.1	81	(7.4)	-
1346	1263	S2205a	ClCCI	1.0	288.43	2133.57	77	-	-	-	-	413.1	81	-	-	(7.4)	-
1347	1264	S2206a	OCCCl	1.01	298.15	1200.9	78	48.3	84	205.2	87	401.8	81	585.0	81	25.1	86
1348	1265	S2207a	OCCBr	1.0	293.15	1762.9	87	54.1	84	-	-	423.1	81	-	-	(25.1)	-
1349	1266	S2208a	OCCF	1.0	293.15	1104.0	87	-	-	246.8	87	376.6	81	-	-	(25.1)	-
1350	1266	S2208b	OCCF	1.0	288.0	-	-	44.1	84	246.8	87	376.6	81	-	-	(25.1)	-
1351	1267	S2209a	OCCI	1.0	293.15	2196.7	87	-	-	-	-	449.1	81	-	-	(25.1)	-
1352	1267	S2209b	OCCI	1.0	288.0	-	-	57.0	84	-	-	449.1	81	-	-	(25.1)	-
1353	1268	S2210a	O=CCO	1.0	373.15	1366.0	87	-	-	370.1	87	-	-	-	-	(12.0)	-
1354	1269	S2211a	NCCO	1.01	298.15	1012.12	78	59.6	84	283.6	87	444.1	81	678.2	81	37.7	86
1355	1270	S2301a	CC(F)(F)Cl	1.07	264.56	1188.76	81	22.7	84	142.7	87	263.1	81	410.3	81	(7.4)	-
1356	1270	S2301b	CC(F)(F)Cl	3.61	298.15	1107.72	81	19.74	81	142.7	87	263.1	81	410.3	81	(7.4)	-
1357	1271	S2302a	CC(F)(Cl)Cl	1.0	298.15	1233.66	77	26.04	81	169.7	87	304.9	81	478.9	81	(7.4)	-
1358	1272	S2303a	FC(Cl)CCl	1.0	298.14	1369.2	77	-	-	213.2	87	346.9	81	523.6	81	(7.4)	-
1359	1273	S2304a	O=CC(Cl)Cl	0.07	298.15	1433.0	81	-	-	-	-	362.0	81	555.0	81	(7.0)	-
1360	1274	S2305a	O=C(O)CCl	0.01	351.6	1354.15	81	-	-	335.1	87	462.5	81	686.0	81	12.3	87
1361	1275	S2306a	O=C(O)CBr	1.0	323.15	1933.5	87	-	-	323.1	87	481.1	81	-	-	(12.3)	-
1362	1276	S2307a	O=C(O)CF	1.0	309.15	1369.3	87	-	-	308.4	87	441.1	81	-	-	(12.3)	-
1363	1277	S2308a	OCC(Cl)Cl	1.0	298.15	1404.0	87	-	-	-	-	419.1	81	-	-	(33.0)	-
1364	1278	S2309a	NCC(=O)O	1.0	571.3	1607.0	87	-	-	563.1	87	-	-	-	-	(8.0)	-
1365	1279	S2401a	FC(F)(Br)CBr	1.0	293.15	2223.8	87	-	-	211.8	87	365.6	81	-	-	(7.4)	-
1366	1280	S2402a	FC(F)(Cl)CCl	1.0	298.14	1406.6	77	-	-	171.9	87	319.8	81	479.1	81	(7.4)	-
1367	1281	S2403a	FC(F)C(F)Br	1.0	283.15	1874.0	77	-	-	-	-	-	-	-	-	(7.4)	-
1368	1282	S2404a	FC(F)(F)CCl	1.0	284.6	1353.9	77	-	-	167.7	87	279.2	81	426.2	81	(7.4)	-
1369	1283	S2405a	FC(Cl)(Cl)CCl	1.0	298.14	1482.6	77	-	-	168.4	87	361.1	81	535.0	81	(7.4)	-
1370	1284	S2406a	FC(Cl)C(Cl)Cl	1.0	298.14	1529.8	77	-	-	-	-	375.1	81	555.6	81	(7.4)	-
1371	1285	S2407a	FC(F)(F)CI	1.0	298.15	2130.0	87	-	-	-	-	327.6	81	-	-	(7.4)	-
1372	1286	S2408a	O=CC(Cl)(Cl)Cl	0.06	298.15	1499.12	81	-	-	215.7	87	370.9	81	565.0	81	6.8	87
1373	1287	S2409a	O=CC(Br)(Br)Br	1.0	298.15	2664.9	87	-	-	-	-	447.1	81	-	-	7.6	87
1374	1288	S2410a	O=C(O)C(Cl)Cl	0.01	356.28	1473.52	81	-	-	285.1	87	467.1	81	686.0	81	8.3	87
1375	1289	S2411a	O=C(O)C(F)F	1.0	298.15	1526.0	87	-	-	272.1	87	406.1	81	-	-	(8.3)	-
1376	1290	S2412a	OCC(F)(F)F	1.01	298.15	1382.4	78	-	-	229.2	87	347.1	81	-	-	26.7	86
1377	1290	S2412b	OCC(F)(F)F	1.0	289.0	-	-	45.9	84	229.2	87	347.1	81	-	-	26.7	86
1378	1291	S2501a	O=C(O)C(Cl)(Cl)Cl	1.0	337.15	1612.6	87	-	-	332.2	87	469.6	81	688.0	81	4.3	87
1379	1292	S2502a	O=C(O)C(F)(F)F	1.0	298.15	1535.1	87	35.9	84	257.9	87	344.9	81	491.2	81	8.4	87
1380	1293	S3201a	CC(C)(Cl)Br	1.0	293.15	1495.0	87	-	-	-	-	368.1	81	-	-	(7.4)	-
1381	1294	S3202a	CC(Br)CCl	1.0	293.15	1537.0	87	-	-	-	-	390.1	81	-	-	(7.4)	-
1382	1295	S3203a	CC(Cl)CBr	1.0	293.15	1531.0	87	-	-	-	-	391.1	81	-	-	(7.4)	-
1383	1296	S3204a	ClCCCBr	1.0	293.15	1596.9	87	-	-	214.3	87	416.4	81	-	-	(7.4)	-
1384	1297	S3205a	CC(C)(F)Cl	1.0	293.15	998.2	77	-	-	-	-	-	-	515.4	81	(7.4)	-
1385	1298	S3206a	CC(F)CCl	1.0	293.15	1086.0	77	-	-	-	-	-	-	-	-	(7.4)	-
1386	1299	S3207a	ClCCCI	1.0	293.15	1904.0	87	-	-	-	-	444.1	81	-	-	(7.4)	-
1387	1300	S3208a	CC(Cl)C=O	1.0	288.15	1182.0	87	-	-	-	-	359.1	81	-	-	(7.0)	-
1388	1301	S3209a	CC(=O)CCl	1.0	293.15	1150.0	87	-	-	228.7	87	392.1	81	-	-	(14.6)	-
1389	1302	S3210a	CC(=O)CBr	1.0	296.15	1634.0	87	-	-	236.7	87	411.1	81	-	-	(14.6)	-

Table S.2 – Reference experimental data (continued)

n_{sim}	n_{iso}	Code	Smiles	P [bar]	T [K]	ρ_{liq} [kg·m^{-3}]	Src	ΔH_{vap} [kJ·mol^{-1}]	Src	T_m [K]	Src	T_b [K]	Src	T$_c$ [K]	Src	ϵ	Src
1390	1303	S3211a	CC(=O)CF	1.0	293.15	1028.8	87	-	-	-	-	350.1	81	-	-	(14.6)	-
1391	1304	S3212a	CC(=O)CI	1.0	288.15	2170.0	87	-	-	-	-	-	-	-	-	(14.6)	-
1392	1305	S3213a	CC(O)CCl	1.0	293.15	1113.0	87	-	-	-	-	400.1	81	-	-	59.0	87
1393	1306	S3214a	CC(Cl)CO	1.0	293.15	1103.0	87	-	-	-	-	406.6	81	-	-	(47.5)	-
1394	1307	S3215a	OCCCCl	1.0	293.15	1130.9	87	-	-	-	-	438.1	81	-	-	36.0	87
1395	1308	S3216a	CC(O)CBr	1.0	303.15	1558.5	87	-	-	-	-	419.6	81	-	-	(47.5)	-
1396	1309	S3217a	OCCCBr	1.0	293.15	1537.4	87	-	-	-	-	395.3	81	-	-	(47.5)	-
1397	1310	S3218a	OCCCI	1.0	499.15	-	-	43.8	81	-	-	499.1	81	-	-	(47.5)	-
1398	1311	S3219a	COCCCl	1.0	293.15	1034.5	87	-	-	-	-	365.6	81	-	-	(14.0)	-
1399	1312	S3220a	COCCBr	1.0	293.15	1462.3	87	-	-	-	-	383.1	81	-	-	(14.0)	-
1400	1313	S3221a	CC(=O)CO	1.0	298.15	1074.31	81	44.18	81	256.1	87	418.6	81	596.0	81	(18.2)	-
1401	1314	S3222a	CNCCO	1.0	293.15	937.0	87	57.8	84	-	-	431.1	81	717.6	81	(21.1)	-
1402	1315	S3223a	CC(O)CN	1.01	298.15	956.51	78	-	-	274.9	87	432.6	81	717.6	81	(21.1)	-
1403	1316	S3224a	NCCCO	1.01	298.15	987.42	78	-	-	285.2	87	460.6	81	717.6	81	(21.1)	-
1404	1317	S3225a	COCC=O	1.0	298.15	1005.0	87	-	-	-	-	365.1	81	-	-	(8.0)	-
1405	1318	S3226a	COCCO	1.01	298.15	960.23	78	45.2	84	188.1	87	397.6	81	564.0	81	17.2	87
1406	1319	S3301a	ClC(Cl)CCBr	1.0	293.15	1708.4	77	-	-	-	-	-	-	-	-	(7.4)	-
1407	1320	S3302a	ClCC(Br)CBr	1.0	287.15	2093.0	87	-	-	-	-	469.1	81	-	-	(7.4)	-
1408	1321	S3303a	CC(F)(Cl)CCl	0.07	298.15	1255.25	81	-	-	-	-	361.8	81	536.5	81	(7.4)	-
1409	1322	S3304a	CC(Cl)C(=O)O	1.0	293.15	1258.5	87	-	-	-	-	458.1	81	-	-	(12.3)	-
1410	1323	S3305a	COC(=O)CCl	1.0	293.15	1236.0	87	46.7	84	240.8	87	403.0	81	600.0	81	12.0	87
1411	1324	S3306a	COC(=O)CBr	1.0	293.15	1635.0	87	-	-	-	-	405.1	81	-	-	(12.0)	-
1412	1325	S3307a	COC(=O)CF	1.0	288.0	-	-	42.7	84	-	-	377.6	81	-	-	(12.0)	-
1413	1326	S3308a	CC(=O)C(Cl)Cl	1.0	291.15	1304.0	87	-	-	-	-	393.1	81	-	-	14.6	87
1414	1327	S3309a	O=C(CCl)CCl	1.0	319.15	1382.6	87	-	-	318.1	87	446.6	81	-	-	(14.6)	-
1415	1328	S3310a	OC(CCl)CCl	0.87	298.15	1356.46	78	-	-	-	-	449.1	81	633.0	81	(33.0)	-
1416	1328	S3310b	OC(CCl)CCl	1.0	316.0	-	-	50.4	84	-	-	449.1	81	633.0	81	(33.0)	-
1417	1329	S3311a	OCC(Cl)CCl	1.0	293.15	1360.7	87	-	-	-	-	457.1	81	647.0	81	(33.0)	-
1418	1330	S3312a	OC(CBr)CBr	1.0	293.15	2136.4	87	-	-	-	-	492.1	81	-	-	(33.0)	-
1419	1331	S3313a	OCC(Br)CBr	1.0	293.15	2120.0	87	-	-	-	-	492.1	81	-	-	(33.0)	-
1420	1332	S3314a	OC(CF)CF	1.0	298.15	1240.0	87	-	-	-	-	400.1	81	-	-	(33.0)	-
1421	1333	S3315a	OCC(Cl)CO	1.0	293.15	1321.9	87	-	-	-	-	489.1	81	-	-	(31.0)	-
1422	1334	S3316a	OCC(O)CCl	1.0	298.15	1315.88	81	-	-	-	-	486.1	81	648.0	81	31.0	87
1423	1335	S3317a	COCC(F)F	1.0	303.0	-	-	31.8	84	-	-	-	-	-	-	(12.4)	-
1424	1336	S3318a	COC(=O)CO	1.0	291.15	1167.7	87	-	-	-	-	422.1	81	-	-	(12.1)	-
1425	1337	S3319a	COCC(=O)O	1.0	293.15	1176.8	87	-	-	-	-	478.3	81	691.0	81	(4.0)	-
1426	1338	S3401a	ClC(Cl)(Br)CCBr	1.0	293.15	2077.2	77	-	-	-	-	-	-	-	-	(7.3)	-
1427	1339	S3402a	CC(Cl)(Cl)C(=O)O	1.0	285.15	1389.0	87	-	-	-	-	460.6	81	-	-	(8.3)	-
1428	1340	S3403a	COC(=O)C(Cl)Cl	1.0	293.15	1377.4	87	47.7	84	221.2	87	416.1	81	-	-	(9.0)	-
1429	1341	S3404a	COC(=O)C(F)F	1.0	288.0	-	-	41.9	84	-	-	358.5	81	-	-	(9.0)	-
1430	1342	S3405a	CC(=O)C(Cl)(Cl)Cl	1.0	293.15	1435.0	87	-	-	-	-	422.1	81	-	-	(14.6)	-
1431	1343	S3406a	CC(=O)C(F)(F)F	1.0	298.15	1252.0	87	-	-	-	-	295.1	81	-	-	(14.6)	-
1432	1344	S3407a	CC(O)C(F)(F)F	1.0	298.0	-	-	44.8	84	-	-	348.1	81	-	-	(26.7)	-
1433	1345	S3501a	COC(=O)C(Cl)(Cl)Cl	1.0	293.15	1487.4	87	48.3	84	255.7	87	426.9	81	-	-	(7.8)	-
1434	1346	S3502a	COC(=O)C(F)(F)F	1.0	293.15	1280.0	87	-	-	-	-	316.6	81	-	-	(7.8)	-
1435	1347	S3503a	OCC(F)(F)C(F)F	1.01	301.55	1482.8	78	53.6	84	258.1	87	382.6	81	-	-	21.0	87
1436	1348	S4201a	ClCCCCBr	1.01	298.15	1485.12	78	-	-	-	-	448.1	81	-	-	(7.4)	-
1437	1349	S4202a	CC(C)(Cl)C=O	1.0	288.15	1053.0	87	-	-	-	-	363.1	81	-	-	(7.0)	-

Table S.2 – Reference experimental data (continued)

n_{sim}	n_{iso}	Code	Smiles	P [bar]	T [K]	ρ_{liq} [kg·m^{-3}]	Src	ΔH_{vap} [kJ·mol^{-1}]	Src	T_m [K]	Src	T_b [K]	Src	T$_c$ [K]	Src	ϵ	Src
1438	1350	S4203a	O=CCCCCl	1.0	281.15	1106.0	87	-	-	-	-	394.6	81	-	-	(7.0)	-
1439	1351	S4204a	CC(=O)C(C)Cl	1.0	298.15	1055.4	87	-	-	-	-	388.1	81	-	-	(14.6)	-
1440	1352	S4205a	CCC(O)CCl	1.0	298.15	1068.0	87	-	-	-	-	414.1	81	-	-	(35.4)	-
1441	1353	S4206a	OCCCCCl	1.0	293.15	1088.3	87	-	-	-	-	388.3	81	-	-	(35.4)	-
1442	1354	S4207a	CCOCCCl	1.0	293.15	989.5	87	-	-	-	-	380.6	81	-	-	(14.0)	-
1443	1355	S4208a	CCOCCBr	1.0	273.15	1385.2	87	-	-	-	-	400.6	81	-	-	(14.0)	-
1444	1356	S4209a	CC(O)CC=O	1.0	298.15	1096.89	81	-	-	-	-	408.1	81	620.0	81	(12.0)	-
1445	1357	S4210a	CC(=O)C(C)O	1.0	298.0	-	-	48.7	84	-	-	418.6	81	-	-	(18.2)	-
1446	1358	S4211a	CCC(=O)CO	1.0	293.15	1027.2	87	-	-	-	-	433.1	81	-	-	(18.2)	-
1447	1359	S4212a	CC(=O)CCO	1.0	293.15	1023.3	87	-	-	-	-	455.1	81	-	-	(18.2)	-
1448	1360	S4213a	CN(C)CCO	1.01	298.15	883.83	78	46.7	84	208.2	87	407.1	81	-	-	13.7	83
1449	1361	S4214a	CC(C)(N)CO	1.01	298.15	932.6	78	-	-	298.6	87	438.6	81	-	-	20.6	78
1450	1361	S4214b	CC(C)(N)CO	1.0	308.0	-	-	63.3	84	298.6	87	438.6	81	-	-	20.6	78
1451	1362	S4215a	CCNCCO	1.0	293.15	914.0	87	61.0	84	-	-	442.6	81	-	-	(17.1)	-
1452	1363	S4216a	NCCCCO	1.0	285.15	967.0	87	-	-	-	-	478.1	81	-	-	(17.1)	-
1453	1364	S4217a	COCC(C)=O	1.0	298.15	957.0	87	-	-	-	-	389.1	81	-	-	(7.0)	-
1454	1365	S4218a	COC(C)CO	1.0	293.15	938.0	87	-	-	-	-	403.1	81	588.8	81	(13.4)	-
1455	1366	S4219a	COCC(C)O	1.01	298.15	915.9	78	46.2	84	-	-	392.1	81	588.8	81	(13.4)	-
1456	1367	S4220a	CCOCCO	1.0	298.15	925.36	78	48.2	84	203.2	87	408.1	81	588.8	81	13.4	87
1457	1368	S4301a	CC(Cl)C(Cl)CBr	1.0	293.15	1598.5	77	-	-	-	-	-	-	-	-	(7.4)	-
1458	1369	S4302a	CCC(Cl)C(=O)O	1.0	293.15	1179.6	87	-	-	-	-	406.8	81	-	-	(12.3)	-
1459	1370	S4303a	O=C(O)CCCCl	1.0	293.15	1223.6	87	-	-	289.1	87	406.8	81	-	-	(12.3)	-
1460	1371	S4304a	CC(C)(Br)C(=O)O	1.0	333.15	1496.9	87	-	-	321.6	87	472.1	81	-	-	(12.3)	-
1461	1372	S4305a	COC(=O)C(C)Cl	1.0	298.15	1075.0	87	-	-	-	-	405.6	81	-	-	11.4	87
1462	1373	S4306a	CCOC(=O)CCl	1.0	293.15	1158.5	87	49.5	84	252.2	87	417.4	81	618.0	81	(9.0)	-
1463	1374	S4307a	CC(=O)OCCCl	1.0	293.15	1178.0	87	-	-	-	-	418.1	81	-	-	(9.0)	-
1464	1375	S4308a	CCOC(=O)CBr	1.0	293.15	1503.2	87	-	-	-	-	441.6	81	-	-	9.8	87
1465	1376	S4309a	CC(=O)OCCBr	1.0	293.15	1514.0	87	-	-	259.4	87	435.6	81	-	-	(9.0)	-
1466	1377	S4310a	COC(=O)CCBr	1.0	291.15	1412.3	87	-	-	-	-	-	-	-	-	5.8	87
1467	1378	S4311a	CCOC(=O)CF	1.0	293.15	1091.2	87	-	-	-	-	393.1	81	-	-	(9.0)	-
1468	1378	S4311b	CCOC(=O)CF	1.0	288.0	-	-	41.9	84	-	-	393.1	81	-	-	(9.0)	-
1469	1379	S4312a	CCOC(=O)CI	1.0	286.15	1817.3	87	-	-	-	-	452.1	81	-	-	(9.0)	-
1470	1380	S4313a	ClCCOCCCl	0.01	333.33	1176.53	81	-	-	226.2	87	451.6	81	666.0	81	21.2	87
1471	1381	S4314a	BrCCOCCBr	1.0	293.15	1845.2	87	-	-	-	-	458.1	81	-	-	(21.2)	-
1472	1382	S4315a	CCOC(=O)CN	1.0	283.15	1027.5	87	-	-	-	-	422.1	81	-	-	(6.0)	-
1473	1383	S4316a	COC(=O)C(C)O	1.0	298.15	-	-	44.29	81	-	-	417.6	81	584.0	81	(12.9)	-
1474	1384	S4317a	CCOC(=O)CO	1.0	296.15	1082.6	87	-	-	-	-	433.1	81	-	-	(12.9)	-
1475	1385	S4318a	CC(=O)OCCO	1.0	288.15	1108.0	87	-	-	-	-	461.1	81	-	-	12.9	87
1476	1386	S4319a	COC(=O)CCO	1.0	336.0	-	-	60.0	84	-	-	452.1	81	-	-	(12.9)	-
1477	1387	S4320a	CCC(O)C(=O)O	1.0	355.87	1046.08	81	-	-	317.4	87	485.1	81	664.0	81	37.7	87
1478	1388	S4321a	CCOCC(=O)O	1.0	293.15	1102.1	87	-	-	-	-	479.6	81	-	-	(4.0)	-
1479	1389	S4322a	COCC(=O)OC	1.0	293.15	1051.1	87	-	-	-	-	404.1	81	-	-	(7.6)	-
1480	1390	S4401a	CC(Cl)C(Cl)(Cl)C=O	1.0	293.15	1395.6	87	-	-	-	-	437.1	81	-	-	(7.2)	-
1481	1391	S4402a	COC(=O)C(Cl)CCl	1.0	293.15	1328.2	87	-	-	-	-	-	-	-	-	(9.0)	-
1482	1392	S4403a	CCOC(=O)C(Cl)Cl	1.0	293.15	1282.7	87	50.6	84	-	-	428.1	81	-	-	(9.0)	-
1483	1393	S4404a	COC(=O)C(Br)CBr	1.0	293.15	1933.3	87	-	-	-	-	479.1	81	-	-	(9.0)	-
1484	1394	S4405a	CCOC(=O)C(Br)Br	1.0	293.15	1899.1	87	-	-	-	-	467.1	81	-	-	(9.0)	-
1485	1395	S4406a	CCOC(=O)C(F)F	1.0	293.15	1176.5	87	-	-	-	-	373.1	81	-	-	(9.0)	-

Table S.2 – Reference experimental data (continued)

n_{sim}	n_{iso}	Code	Smiles	P [bar]	T [K]	ρ_{liq} [kg·m^{-3}]	Src	ΔH_{vap} [kJ·mol^{-1}]	Src	T_m [K]	Src	T_b [K]	Src	T$_c$ [K]	Src	ϵ	Src
1486	1396	S4407a	O=C(CF)OCCF	1.0	288.0	-	-	55.1	84	-	-	-	-	-	-	(9.0)	-
1487	1397	S4408a	CCOC(=O)C(F)Cl	1.0	293.15	1225.0	87	-	-	-	-	402.1	81	-	-	(9.0)	-
1488	1398	S4409a	O=C(CF)OCCCl	1.0	288.0	-	-	56.4	84	-	-	393.1	81	-	-	(9.0)	-
1489	1399	S4501a	CCOC(=O)C(Cl)(Cl)Cl	1.0	293.15	1383.6	87	51.0	84	-	-	440.6	81	-	-	8.4	87
1490	1400	S4502a	CCOC(=O)C(F)(F)F	1.0	293.15	1194.0	87	-	-	-	-	334.1	81	-	-	(8.4)	-
1491	1401	S5201a	CC(=O)CCCCl	1.0	293.15	1052.3	87	-	-	-	-	396.5	81	-	-	(14.6)	-
1492	1402	S5202a	CC(=O)CN(C)C	1.0	298.0	-	-	43.6	84	-	-	392.6	81	-	-	(8.0)	-
1493	1403	S5203a	CC(=O)C(C)(C)O	1.0	293.15	952.6	87	-	-	-	-	413.6	81	-	-	(18.2)	-
1494	1404	S5204a	CC(=O)CCCO	1.0	293.15	1007.1	87	-	-	-	-	482.1	81	-	-	(18.2)	-
1495	1405	S5205a	CC(CO)N(C)C	1.0	299.15	882.0	87	-	-	-	-	423.4	81	-	-	(21.1)	-
1496	1406	S5206a	CC(O)CN(C)C	1.0	298.15	837.0	87	-	-	-	-	397.6	81	-	-	(21.1)	-
1497	1407	S5207a	CN(C)CCCO	1.01	298.15	880.82	78	57.0	84	-	-	436.6	81	-	-	(21.1)	-
1498	1408	S5208a	CC(C)NCCO	1.0	298.15	893.23	78	-	-	401.6	87	446.1	81	-	-	(21.1)	-
1499	1409	S5209a	CCCNCCO	1.0	293.15	900.5	87	-	-	-	-	455.1	81	-	-	(21.1)	-
1500	1410	S5210a	CCOCCC=O	1.0	293.15	916.5	87	-	-	-	-	408.4	81	-	-	(8.0)	-
1501	1411	S5211a	COC(C)CCO	1.0	296.15	923.0	87	-	-	-	-	430.1	81	614.5	81	(11.1)	-
1502	1412	S5212a	CC(C)OCCO	1.01	298.15	899.69	78	50.1	84	-	-	418.1	81	614.5	81	10.8	79
1503	1413	S5213a	CCOCC(C)O	1.01	298.15	896.65	78	-	-	-	-	404.1	81	614.5	81	(11.1)	-
1504	1414	S5214a	CCCOCCO	1.01	298.15	907.88	78	52.1	84	-	-	424.5	81	615.2	81	11.5	78
1505	1415	S5301a	CCC(F)(F)C(C)Cl	1.0	293.15	1108.5	77	-	-	-	-	-	-	-	-	(7.4)	-
1506	1416	S5302a	O=C(O)CCCCCl	1.0	298.15	1341.6	87	-	-	291.1	87	503.1	81	-	-	(12.3)	-
1507	1417	S5303a	CC(C)OC(=O)CCl	1.0	293.15	1088.8	87	-	-	-	-	423.6	81	-	-	(10.3)	-
1508	1418	S5304a	CCOC(=O)C(C)Cl	1.0	293.15	1079.3	87	-	-	-	-	420.1	81	-	-	11.9	87
1509	1419	S5305a	CCCOC(=O)CCl	1.0	293.15	1104.0	87	-	-	-	-	434.1	81	-	-	(10.3)	-
1510	1420	S5306a	CCOC(=O)CCCl	1.0	293.15	1108.6	87	-	-	-	-	435.1	81	-	-	10.2	87
1511	1421	S5307a	COC(=O)CCCCl	1.0	293.15	1129.3	87	-	-	-	-	447.1	81	-	-	9.5	87
1512	1422	S5308a	CCC(Br)C(=O)OC	1.0	293.15	1452.8	87	-	-	-	-	441.1	81	-	-	(10.3)	-
1513	1423	S5309a	CCOC(=O)C(C)Br	1.0	293.15	1413.5	87	-	-	-	-	433.1	81	-	-	9.4	87
1514	1424	S5310a	CCOC(=O)CCBr	1.0	291.15	1412.3	87	-	-	-	-	452.1	81	-	-	(10.3)	-
1515	1425	S5311a	COC(=O)CCCBr	1.0	298.15	1400.0	87	-	-	-	-	459.6	81	-	-	(10.3)	-
1516	1426	S5312a	CC(C)OC(=O)CF	1.0	288.0	-	-	44.3	84	-	-	-	-	-	-	(10.3)	-
1517	1427	S5313a	COC(=O)CCCF	1.0	288.0	-	-	47.3	84	-	-	-	-	-	-	(10.3)	-
1518	1428	S5314a	COC(=O)CN(C)C	1.0	293.0	-	-	43.9	84	-	-	-	-	-	-	(6.0)	-
1519	1429	S5315a	CC(=O)CCC(=O)O	1.0	308.15	1120.13	81	-	-	306.1	87	519.0	81	738.0	81	19.1	83
1520	1430	S5316a	CC(=O)COC(C)=O	1.0	293.15	1075.7	87	-	-	-	-	444.1	81	-	-	(13.0)	-
1521	1431	S5317a	CCOC(=O)C(C)=O	1.0	288.15	1059.6	87	-	-	223.2	87	428.1	81	-	-	(13.0)	-
1522	1432	S5318a	COC(=O)CC(C)=O	0.01	332.74	1035.66	81	45.58	81	300.6	87	444.9	81	642.0	81	(13.0)	-
1523	1433	S5319a	CC(C)(O)CC(=O)O	1.0	293.15	938.4	87	-	-	-	-	426.0	81	588.0	81	(33.9)	-
1524	1434	S5320a	COCCC(=O)OC	1.0	288.15	1013.9	87	-	-	-	-	415.6	81	-	-	(7.6)	-
1525	1435	S5321a	COCCOC(C)=O	1.0	292.15	1007.4	87	-	-	203.2	87	416.1	81	-	-	(7.6)	-
1526	1435	S5321b	COCCOC(C)=O	1.0	298.0	-	-	50.3	84	203.2	87	416.1	81	-	-	(7.6)	-
1527	1436	S5401a	CCOC(=O)C(Cl)CCl	1.0	293.15	1240.1	87	-	-	-	-	456.6	81	-	-	(9.0)	-
1528	1437	S5402a	CCOC(=O)C(Br)CBr	1.0	293.15	1796.6	87	-	-	-	-	487.6	81	-	-	(9.0)	-
1529	1438	S5403a	COC(=O)CC(Cl)CF	1.0	288.0	-	-	54.5	84	-	-	-	-	-	-	(9.0)	-
1530	1439	S5501a	CC(C)OC(=O)C(Cl)(Cl)Cl	1.0	298.15	1291.1	87	-	-	-	-	448.1	81	-	-	(8.3)	-
1531	1440	S5502a	CCCOC(=O)C(Cl)(Cl)Cl	1.0	293.15	1322.1	87	-	-	-	-	460.1	81	-	-	8.3	87
1532	1441	S6201a	CC(C)(C)C(C)(Cl)Br	1.0	293.15	1250.0	77	-	-	-	-	-	-	-	-	(7.4)	-
1533	1442	S6202a	OCCCCCCCl	1.0	293.15	1024.1	87	-	-	-	-	431.3	81	-	-	21.6	87

Table S.2 – Reference experimental data (continued)

n_{sim}	n_{iso}	Code	Smiles	P [bar]	T [K]	ρ_{liq} [kg·m^{-3}]	Src	ΔH_{vap} [kJ·mol^{-1}]	Src	T_m [K]	Src	T_b [K]	Src	T_c [K]	Src	ϵ	Src
1534	1443	S6203a	CC(=O)CC(C)(C)O	1.0	293.15	938.7	87	-	-	226.2	87	441.0	81	606.0	81	18.2	87
1535	1444	S6204a	CCN(CC)CCO	1.01	298.15	880.37	78	52.5	84	-	-	436.1	81	-	-	(12.4)	-
1536	1445	S6205a	CCCCNCCO	1.0	293.15	890.7	87	-	-	-	-	472.1	81	-	-	(12.4)	-
1537	1446	S6206a	CC(O)COC(C)C	1.0	293.15	879.0	87	-	-	-	-	410.6	81	637.8	81	(9.2)	-
1538	1447	S6207a	CCCOCC(C)O	1.01	298.15	880.84	78	-	-	-	-	423.1	81	637.8	81	8.7	83
1539	1448	S6208a	CC(C)COCCO	1.0	293.15	890.0	87	-	-	-	-	433.1	81	637.8	81	9.1	79
1540	1449	S6209a	CCCCOCCO	1.01	298.15	896.16	78	56.6	84	198.3	87	444.5	81	633.9	81	9.9	86
1541	1450	S6301a	CC(C)OC(=O)C(C)Cl	1.0	293.15	1031.5	87	-	-	-	-	424.6	81	-	-	(8.6)	-
1542	1451	S6302a	CCCOC(=O)CCCl	1.0	293.15	1065.6	87	-	-	-	-	453.1	81	-	-	(8.6)	-
1543	1452	S6303a	CCCCOC(=O)CCl	1.0	293.15	1070.4	87	-	-	-	-	456.1	81	-	-	(8.6)	-
1544	1453	S6304a	CCOC(=O)CCCCl	1.0	293.15	1075.6	87	-	-	-	-	457.1	81	-	-	(8.6)	-
1545	1454	S6305a	CCOC(=O)C(C)(C)Br	1.0	293.15	1326.3	87	-	-	-	-	436.1	81	-	-	8.6	87
1546	1455	S6306a	CCOC(=O)C(Br)CC	1.0	293.15	1327.3	87	-	-	-	-	450.1	81	-	-	8.6	87
1547	1456	S6307a	CCOC(=O)CCCBr	1.0	293.15	1354.0	87	-	-	-	-	465.1	81	-	-	(8.6)	-
1548	1457	S6308a	CCN(CC)CC(Cl)Cl	1.0	288.0	-	-	54.9	84	-	-	-	-	-	-	(7.0)	-
1549	1458	S6309a	CCN(CCCl)CCCl	1.0	296.15	1086.1	87	-	-	239.2	87	-	-	-	-	(7.0)	-
1550	1459	S6310a	CC(CCl)OC(C)CCl	1.0	293.15	1103.0	87	-	-	-	-	460.1	81	-	-	(12.4)	-
1551	1460	S6311a	ClCCCOCCCCl	1.0	293.15	1136.0	87	-	-	-	-	489.1	81	-	-	(12.4)	-
1552	1461	S6312a	CC(C)OC(=O)C(C)O	1.0	293.15	998.0	87	-	-	-	-	440.1	81	-	-	(12.1)	-
1553	1462	S6313a	CCOC(=O)CC(C)O	1.0	298.0	-	-	55.9	84	-	-	450.6	81	-	-	(12.1)	-
1554	1463	S6314a	CC(=O)CCCC(=O)O	1.0	298.15	1090.0	87	-	-	286.6	87	547.6	81	-	-	(19.1)	-
1555	1464	S6315a	COC(=O)C(C)C(C)=O	1.0	298.15	1021.7	87	-	-	-	-	450.6	81	-	-	(14.4)	-
1556	1465	S6316a	CCOC(=O)CC(C)=O	1.0	283.15	1036.8	87	-	-	228.2	87	453.9	81	643.0	81	14.0	87
1557	1466	S6317a	COC(=O)CCC(C)=O	1.0	293.15	1051.1	87	-	-	-	-	469.1	81	-	-	14.8	83
1558	1467	S6318a	CCOCC(=O)OCC	1.0	293.15	970.2	87	-	-	-	-	431.1	81	-	-	(7.6)	-
1559	1468	S6319a	CCOCCOC(C)=O	1.0	293.15	974.0	87	52.7	84	211.4	87	429.4	81	607.3	81	7.6	87
1560	1469	S6401a	CCCCOC(=O)C(Cl)Cl	1.0	293.15	1182.0	87	-	-	-	-	466.6	81	-	-	(9.0)	-
1561	1470	S6402a	CCOC(=O)C(Br)CCBr	1.0	293.15	1698.7	87	-	-	-	-	495.6	81	-	-	(9.0)	-
1562	1471	S6403a	CCOC(=O)CCC(=O)O	1.0	293.15	1146.6	87	-	-	281.1	87	482.9	81	-	-	(8.4)	-
1563	1472	S6404a	COC(=O)CCCC(=O)O	1.0	298.15	1169.0	87	-	-	-	-	482.9	81	-	-	8.4	87
1564	1473	S6501a	CC(C)COC(=O)C(Cl)(Cl)Cl	1.0	293.15	1263.6	87	-	-	-	-	461.1	81	-	-	7.7	87
1565	1474	S6502a	CCCCOC(=O)C(Cl)(Cl)Cl	1.0	293.15	1277.8	87	-	-	-	-	477.1	81	-	-	7.5	87
1566	1475	S6503a	CCCCOC(=O)C(F)(F)F	1.0	295.15	1026.8	87	-	-	-	-	375.1	81	-	-	(7.6)	-
1567	1476	S7201a	CCN(CC)CC(C)=O	1.0	298.0	-	-	47.7	84	-	-	430.1	81	-	-	(8.0)	-
1568	1477	S7202a	CCN(CC)CCCO	1.0	293.15	860.0	87	-	-	-	-	462.6	81	-	-	(21.1)	-
1569	1478	S7203a	COC(C)(C)CC(C)=O	1.0	298.15	898.0	87	-	-	-	-	433.1	81	-	-	(7.0)	-
1570	1479	S7204a	CC(O)COC(C)(C)C	0.01	319.87	-	-	47.66	81	-	-	420.2	81	659.3	81	(11.5)	-
1571	1480	S7205a	CCCCOCC(C)O	1.0	298.15	874.63	78	-	-	-	-	444.6	81	659.3	81	(11.5)	-
1572	1481	S7301a	CCCOC(=O)C(Cl)CC	1.0	293.15	1025.2	87	-	-	-	-	456.1	81	-	-	(9.7)	-
1573	1482	S7302a	CCCCOC(=O)C(C)Cl	1.0	293.15	1025.3	87	-	-	-	-	457.1	81	-	-	(9.7)	-
1574	1483	S7303a	CC(C)COC(=O)CCCl	1.0	293.15	1032.3	87	-	-	-	-	464.4	81	-	-	(9.7)	-
1575	1484	S7304a	CCCCOC(=O)CCCl	1.0	293.15	1037.0	87	-	-	-	-	449.4	81	-	-	(9.7)	-
1576	1485	S7305a	CCOC(=O)C(Br)C(C)C	1.0	293.15	1276.0	87	-	-	-	-	459.1	81	-	-	(9.7)	-
1577	1486	S7306a	CCCC(Br)C(=O)OCC	1.0	291.15	1226.0	87	-	-	-	-	464.1	81	-	-	(9.7)	-
1578	1487	S7307a	CCOC(=O)CCCCBr	1.0	293.15	1308.5	87	-	-	-	-	499.9	81	-	-	(9.7)	-
1579	1488	S7308a	CC(Cl)CN(C)CC(C)Cl	1.0	288.0	-	-	54.6	84	-	-	-	-	-	-	(7.0)	-
1580	1489	S7309a	CCCN(CCCl)CCCl	1.0	288.0	-	-	56.8	84	-	-	-	-	-	-	(7.0)	-
1581	1490	S7310a	CCC(C(C)=O)C(=O)OC	1.0	287.15	995.0	87	-	-	-	-	455.1	81	-	-	(12.8)	-

Table S.2 – Reference experimental data (continued)

n_{sim}	n_{iso}	Code	Smiles	P [bar]	T [K]	ρ_{liq} [kg·m^{-3}]	Src	ΔH_{vap} [kJ·mol^{-1}]	Src	T_m [K]	Src	T_b [K]	Src	T$_c$ [K]	Src	ϵ	Src
1582	1491	S7311a	CC(=O)CC(=O)OC(C)C	1.0	293.15	983.5	87	-	-	245.8	87	459.1	81	-	-	(12.8)	-
1583	1492	S7312a	CCOC(=O)C(C)C(C)=O	1.0	293.15	994.1	87	-	-	-	-	460.1	81	-	-	(12.8)	-
1584	1493	S7313a	CCOC(=O)CC(=O)CC	1.0	293.15	1012.0	87	-	-	-	-	464.1	81	-	-	(12.8)	-
1585	1494	S7314a	CCOC(=O)CCC(C)=O	1.0	293.15	1011.1	87	-	-	-	-	478.9	81	-	-	12.9	83
1586	1495	S7315a	CCOCCC(=O)OCC	1.0	293.15	949.0	87	-	-	-	-	438.1	81	621.0	81	(7.6)	-
1587	1496	S7316a	CCCOCCOC(C)=O	1.0	298.0	-	-	55.6	84	-	-	442.2	81	-	-	(7.6)	-
1588	1497	S7401a	COC(=O)CCCCC(=O)O	1.0	293.15	1062.3	87	-	-	282.1	87	464.2	81	-	-	6.7	87
1589	1498	S7501a	CC(C)CCOC(=O)C(Cl)(Cl)Cl	1.0	293.15	1231.4	87	-	-	-	-	490.1	81	-	-	7.3	87
1590	1499	S8201a	CCCC(=O)C(O)CCC	1.0	289.15	910.7	87	-	-	263.1	87	458.1	81	-	-	(18.2)	-
1591	1500	S8202a	CC(C)N(CCO)C(C)C	1.0	298.15	826.0	87	-	-	-	-	463.1	81	-	-	(21.1)	-
1592	1501	S8203a	CCCCCCOCCO	1.0	293.15	887.8	87	-	-	228.1	87	481.1	81	679.3	81	(11.5)	-
1593	1502	S8301a	CC(C)CCOC(=O)C(C)O	1.0	298.15	958.9	87	-	-	-	-	475.6	81	-	-	11.2	87
1594	1503	S8302a	CCCC(O)CC(=O)OCC	1.0	298.0	-	-	61.9	84	-	-	-	-	-	-	(11.2)	-
1595	1504	S8303a	CC(=O)CC(=O)OC(C)(C)C	1.0	293.15	975.6	87	-	-	-	-	460.1	81	-	-	(13.0)	-
1596	1505	S8304a	CCOC(=O)C(CC)C(C)=O	1.0	289.15	984.7	87	-	-	-	-	471.1	81	-	-	(13.0)	-
1597	1506	S8305a	CCOC(=O)CC(=O)C(C)C	1.0	298.15	980.0	87	-	-	264.1	87	446.1	81	-	-	(13.0)	-
1598	1507	S8306a	CCCCOC(=O)CC(C)=O	1.0	298.15	967.1	87	-	-	237.6	87	469.0	81	-	-	(13.0)	-
1599	1508	S8307a	CCOC(=O)CCCC(C)=O	1.0	298.15	989.0	87	-	-	-	-	494.6	81	-	-	(13.0)	-
1600	1509	S8308a	CCCCOCCOC(C)=O	1.0	298.15	-	-	59.5	84	-	-	465.7	81	641.2	81	(7.6)	-
1601	1510	S8401a	CCCCOC(=O)CCC(=O)O	1.0	293.15	1073.2	87	-	-	281.8	87	495.1	81	-	-	(7.5)	-
1602	1511	S9301a	CCOC(=O)CC(=O)C(C)(C)C	1.0	291.15	970.0	87	-	-	-	-	-	-	-	-	(10.3)	-
1603	1512	S9302a	CCOC(=O)C(C(C)=O)C(C)C	1.0	291.15	964.8	87	-	-	-	-	474.1	81	-	-	(10.3)	-
1604	1513	S9303a	CCCC(C(C)=O)C(=O)OCC	1.0	293.15	966.1	87	-	-	-	-	497.1	81	-	-	(10.3)	-
1605	1514	S9304a	CCCCOC(=O)CCC(C)=O	1.0	293.15	973.5	87	-	-	-	-	510.6	81	-	-	10.3	83
1606	1515	S0201a	CC(CC=O)CCCC(C)(C)O	1.0	293.15	922.0	87	75.3	84	-	-	473.7	81	-	-	(12.0)	-
1607	1516	S0301a	CCCCCCCCC(Br)C(=O)O	1.0	297.15	1191.2	87	-	-	275.1	87	-	-	-	-	(12.3)	-

Code	P [bar]	T [K]	γ [mN·m^{-1}]	Src	P [bar]	T [K]	ϵ	Src	P [bar]	T [K]	D [10^{-9} m^2·s^{-1}]	Src
F1101	38.4	298.1	2.1	81	1.0	193.7	25.1	86	2.0	178.0	3.200	82
C1401	1.0	298.1	26.2	86	1.0	298.1	2.2	87	1.0	298.2	1.340	82
B1201	1.0	298.1	39.0	80	1.0	298.1	7.2	87	1.0	297.8	1.526	82
I1101	1.0	298.1	29.8	81	1.0	298.2	6.9	86	1.0	298.2	3.500	82
O2101	1.0	248.2	18.7	86	1.0	246.6	6.6	87	19.7	235.5	2.160	82
K3101	1.0	298.1	23.1	86	1.0	298.2	20.6	86	1.0	298.2	4.530	82
E3202	1.0	298.1	24.7	85	1.0	298.1	6.7	83	1.0	298.2	3.280	82
L1101	1.0	298.1	22.1	85	1.0	298.1	32.6	78	1.0	298.2	2.950	82
D2201	1.0	298.1	27.0	85	1.0	303.1	6.1	83	1.0	298.2	1.190	82
M3201	1.0	320.0	31.3	80	1.0	313.1	163.8	78	1.0	305.5	0.370	82
C1101	1.0	229.0	26.4	81	1.0	247.2	13.0	86	1.0	233.0	3.400	82
C1201	1.0	298.1	27.2	80	1.0	298.1	8.8	87	1.0	298.2	3.800	82
C1301	1.0	298.1	26.6	86	1.0	298.1	4.7	87	1.0	298.2	3.040	82
I1201	1.0	293.1	67.0	85	1.0	298.1	5.3	87	1.0	297.1	0.532	82
S1405	1.0	243.2	15.9	86	1.0	223.2	2.5	86	1.0	293.2	4.100	82
S1406	1.0	297.0	18.1	86	1.0	293.2	3.0	86	1.1	379.0	6.020	82
S1302	1.0	232.3	18.7	85	1.0	130.0	24.9	83	1.0	232.0	3.310	82
C2202	1.0	298.1	31.8	86	1.0	298.2	10.2	86	1.0	298.2	0.890	82
C2301	1.0	301.4	24.8	85	1.0	298.1	7.1	87	1.0	293.2	1.390	82
C4101	1.0	298.1	18.9	86	1.0	298.2	9.6	86	1.0	298.2	1.600	82
B2101	1.0	298.1	23.6	80	1.0	298.1	9.0	87	1.0	295.7	3.800	82
B2202	1.0	298.2	38.2	86	1.0	298.2	4.8	86	1.0	294.7	0.780	82
B4101	1.0	298.1	21.1	81	1.0	293.1	11.0	87	1.0	300.1	1.510	82
I2101	1.0	293.5	28.1	85	1.0	298.2	7.6	86	1.0	292.5	2.212	82
I4101	1.0	298.1	20.6	81	1.0	298.1	6.4	87	1.0	296.1	0.940	82
I4104	1.0	298.1	28.8	81	1.0	298.1	6.2	87	1.0	292.5	1.347	82
D4201	1.0	296.6	24.8	85	1.0	283.2	2.7	86	1.0	299.5	0.830	82
D8208	1.0	293.1	29.2	85	1.0	300.3	2.5	86	1.0	323.2	0.390	82
D9205	1.0	306.6	36.2	81	1.0	298.1	2.5	87	1.0	298.2	0.160	82
E4204	1.0	298.1	23.4	80	1.0	298.1	6.0	83	1.0	298.2	2.770	82
E5208	1.0	298.1	23.8	80	1.0	298.1	5.7	83	1.0	303.2	2.500	82
E6215	1.0	298.1	24.3	85	1.0	298.1	5.0	83	1.0	303.2	1.710	82
E7225	1.0	298.1	25.2	85	1.0	298.1	4.7	87	1.0	303.2	1.360	82
E8235	1.0	298.1	26.4	85	1.0	303.1	4.4	83	1.0	303.2	1.090	82
E0226	1.0	298.1	25.5	81	1.0	298.1	4.1	87	1.0	303.2	0.720	82
K5103	1.0	293.1	24.8	85	1.0	298.1	15.2	87	1.0	298.2	2.970	82
K6106	1.0	298.1	25.4	80	1.0	303.1	14.0	83	1.0	298.2	2.060	82
K7115	1.0	298.1	26.1	80	1.0	298.2	11.7	86	1.0	298.2	1.610	82
K8122	1.0	295.2	25.6	85	1.0	298.2	9.5	86	1.0	298.2	1.180	82
K9121	1.0	293.4	26.3	85	1.0	295.2	9.1	86	1.0	298.2	0.940	82
K0113	1.0	298.1	25.6	81	1.0	287.1	8.3	87	1.0	298.2	0.750	82
L2101	1.0	298.1	22.0	86	1.0	298.1	24.9	87	1.0	298.2	1.160	82
L3101	1.0	298.1	20.9	85	1.0	298.1	19.3	87	1.0	298.2	0.580	82
L3102	1.0	298.1	23.4	86	1.0	298.1	20.5	86	1.0	298.2	0.590	82
L4101	1.0	308.1	19.3	85	1.0	299.2	12.3	86	1.0	299.4	0.310	82
L4104	1.0	298.1	23.9	78	1.0	298.1	17.4	86	1.0	298.2	0.460	82
L5104	1.0	298.1	23.6	80	1.0	298.1	13.2	78	4.9	297.2	0.140	82
L5105	1.0	298.1	23.8	86	1.0	298.1	14.9	87	1.0	296.2	0.270	82
L5106	1.0	298.1	23.3	86	1.0	298.2	13.2	86	9.9	296.9	0.120	82
L5107	1.0	298.1	25.1	86	1.0	298.1	15.0	86	1.0	298.2	0.300	82
L6117	1.0	298.1	25.4	85	1.0	298.2	12.9	86	1.0	298.2	0.220	82
L7137	1.0	298.2	26.8	86	1.0	298.1	11.5	78	1.0	298.2	0.170	82
L8157	1.0	298.1	27.1	80	1.0	298.1	10.0	78	1.0	298.2	0.140	82
L2201	1.0	298.1	48.2	86	1.0	298.1	40.6	86	1.0	298.2	0.100	82
L3201	1.0	298.1	35.7	85	1.0	298.1	28.7	86	1.0	304.0	0.060	82
L3202	1.0	298.1	53.1	86	1.0	298.1	34.3	86	1.0	304.0	0.060	82
L4205	1.0	298.1	37.8	85	1.0	298.1	28.7	86	1.0	297.4	0.110	82
L4206	1.0	298.1	45.5	86	1.0	298.1	31.6	78	1.0	304.0	0.030	82
L6206	1.0	293.1	33.1	85	1.0	298.1	25.1	83	1.0	297.4	0.230	82
L3301	1.0	298.1	62.5	85	1.0	298.1	44.1	86	1.0	298.1	0.002	82
O4103	1.0	296.7	16.8	85	1.0	298.1	4.2	87	1.0	287.4	6.300	82
O4202	1.0	298.1	23.9	78	1.0	298.1	7.2	83	1.0	292.9	3.000	82
O5203	1.0	294.2	21.1	85	1.0	293.1	2.5	87	1.0	294.7	3.200	82
O6204	1.0	298.1	18.6	81	1.0	298.1	3.9	87	1.0	303.2	2.200	82
O6301	1.0	298.1	29.3	80	1.0	298.2	7.3	86	1.0	303.2	1.080	82
M4201	1.0	298.1	33.1	80	1.0	298.1	39.4	83	9.9	298.0	0.790	82

Table S.3: Reference experimental data for the surface-tension coefficient γ, the static relative dielectric permittivity ϵ, and the self-diffusion coefficient D.

S.4 Covalent Interaction Parameters

The covalent interaction parameters were not subject to optimization, and ported directly from the 2016H66 parameter set.[94] The corresponding values are provided in Tab. S.4. For the torsional-dihedral potentials, the 2016H66 parameter set includes 12 types (labeled A-L) relevant for the molecules considered here. These are shown in Fig. S.3a, and the corresponding parameters are listed in Tab. S.4. Due to the inclusion of polyfunctional compounds in the present calculations (multiple occurrences of the same functional group), the assignment of these torsional-dihedral types has been generalized as shown in Fig. S.3b. The van der Waals radii assigned to the atoms when calculating the charges *via* the EE scheme are listed in Tab. S.5.

Bond stretching		
Quartic force constant [10^6 kJ·mol^{-1}·nm^{-4}]	Reference bond length [nm]	Usage
15.70	0.1000	H - OA
18.70	0.1000	H - N
12.30	0.1090	HC - C
16.60	0.1230	C = O
11.80	0.1330	C, CR1 - N, NR
10.20	0.1360	C - OA,OE
8.18	0.1430	CHn - OA,OE
8.71	0.1470	CHn - N, NR
7.15	0.1530	C,CHn - C,CHn
15.09	0.13307	C - F
8.12	0.17580	C - Cl
6.95	0.19212	C - Br
5.67	0.21610	C - I

Bond-angle bending		
Cosine-harmonic force constant [kJ·mol^{-1}]	Reference bond angle [deg]	Usage
380	109.50	H - NL, NT - H, CHn - OA - CHn
425	109.50	H - NL - C, CHn H - NT - CHn
450	109.50	X - OA, SI - X
520	109.50	CHn,C - CHn - C, CHn, OA, OM, N, NE
530	111.00	CHn - CHn - C,CHn,OA,OE,NR,NT,NL
545	113.00	CHn - CH2 - S or CHn - C - OA,OE
460	115.00	H - N - CHn
610	115.00	CHn, C - C - OA, N, NT, NL
620	116.00	CH2 - N - CH1 or CHn - C - CHn,HC
635	117.00	CH3 - N - C or CHn - C - OM or C - OE - CHn
390	120.00	H - NT, NZ, NE - C
445	120.00	H - NT, NZ - H
505	120.00	H - N - CH3, H / H - NT - CHn / HC - C - O
685	121.00	O - C - CHn, C CH3 - N - CHn
700	122.00	CH1, CH2 - N - C / O - C - OA, OE, CHn
415	123.00	H - N - C
730	124.00	O - C - OA, N, NT, NL C - NE - CH2
750	125.00	CHn - C - O (carb. acids and esters)
618	109.5	X,C - C - X

Improper-dihedral distortion		
Harmonic force constant [kJ·mol^{-1} deg^{-2}]	Reference improper dihedral angle [deg]	Usage
0.051	0.0	planar groups
0.102	35.26439	tetrahedral centers

Torsional-dihedral rotation				
Type	Force constant [kJ·mol^{-1}]	Phase shift	Multiplicity	Usage
A	5.920	0.0	3	X,C - C - C - C,X
B	0.931	180.0	1	CHn-CHn-OE-CHn
C	0.569	0.0	2	CHn-CHn-OE-CHn
D	4.682	0.0	3	CHn-CHn-OE-CHn
E	6.942	180.0	1	OE-CHn-CHn-OE
F	3.312	0.0	2	OE-CHn-CHn-OE
G	6.787	0.0	3	OE-CHn-CHn-OE
H	3.770	0.0	3	-C,CHn,SI-
I	1.000	0.0	6	-CHn-C,NR(ring), CR1-
J	1.260	0.0	3	-CHn-OA(no sugar)-
K	3.500	180.0	2	-C-N,NT,NE,NZ,NR-
L	16.700	180.0	2	-C-OA,OE-

Table S.4: Covalent types of the GROMOS-compatible force field along with the values of the associated parameters. The covalent types are listed along with their usage and the values of the interaction parameters. These are force constants, along with reference bond lengths, bond angles, improper dihedral angles, and, for the torsions, multiplicities and phase-shift angles. These $N_{\mathrm{prm}}^{\mathrm{cov}} = 104$ covalent interaction parameters are ported from the 2016H66 parameter set[94] or, whenever unavailable, inferred from QM calculation results. They are not subject to optimization. The torsional types are illustrated in Fig. S.3.

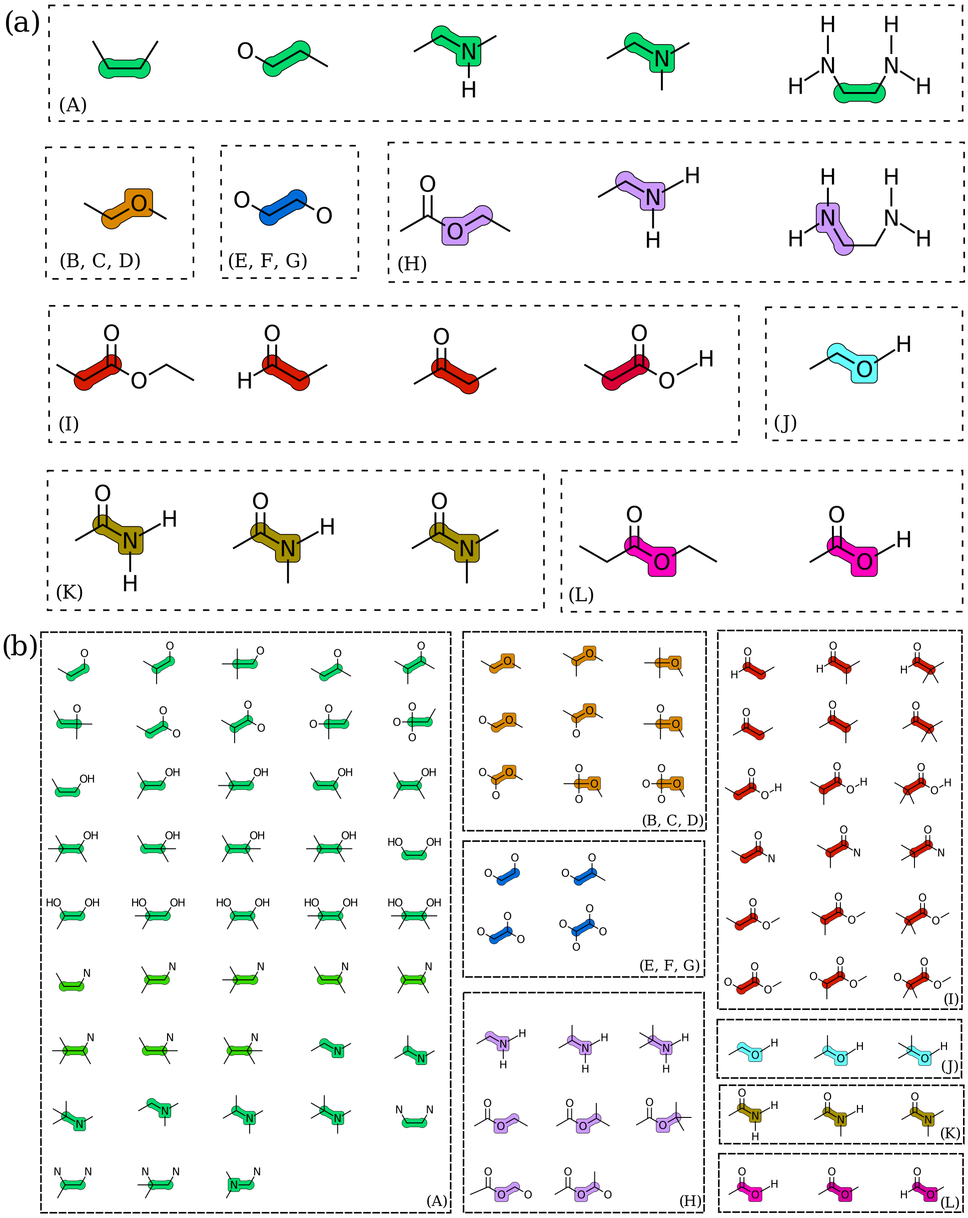

Figure S.3: Application of the different torsional-dihedral types considering (a) the 2016H66 parameter set[94] and (b) the GROMOS-compatible force field for the molecules considered here. The parameters associated with the 12 types A-L are provided in Tab. S.4.

Atom	radius [nm]
H	0.120
C	0.170
CH0	0.170
CH1	0.220
CH2	0.220
CH3	0.220
CH4	0.220
O	0.152
N	0.155
F	0.147
Cl	0.175
Br	0.185
I	0.198

Table S.5: van der Waals radii of the atoms used in the calculation of the charges *via* the EE scheme.

S.5 Final Values of the Non-Bonded Parameters

The final values of the non-bonded interaction parameters, obtained after calibration, are reported in Tab. S.6 for the LJ-types and in Tab. S.7 for the EE-types.

	GM					LB					WH				
LJ type	σ	$\tilde{\sigma}$	σ^*	ϵ	ϵ^*	σ	$\tilde{\sigma}$	σ^*	ϵ	ϵ^*	σ	$\tilde{\sigma}$	σ^*	ϵ	ϵ^*
		[nm]		[kJ·mol^{-1}]			[nm]		[kJ·mol^{-1}]			[nm]		[kJ·mol^{-1}]	
Carbon															
CH0	0.635	-	0.336	0.006	0.406	0.598	-	0.478	0.006	0.005	0.555	-	0.444	0.007	0.005
CH1	0.465	-	0.330	0.102	0.567	0.462	-	0.369	0.103	0.082	0.440	-	0.352	0.105	0.084
CH2	0.400	-	0.316	0.414	1.176	0.400	-	0.320	0.415	0.332	0.391	-	0.313	0.423	0.339
CH3	0.381	-	0.310	0.850	1.947	0.382	-	0.305	0.837	0.670	0.381	-	0.304	0.891	0.713
CH4	0.372	-	0.304	1.302	0.713	0.382	-	0.306	1.199	0.959	0.367	-	0.294	1.203	0.962
C=O	0.383	-	0.336	0.390	0.406	0.383	-	0.306	0.431	0.344	0.355	-	0.284	0.451	0.361
Halogen															
F	0.265	-	0.284	0.705	0.361	0.251	-	0.201	0.799	0.639	0.301	-	0.241	0.843	0.674
Cl	0.334	-	0.241	1.610	0.674	0.333	-	0.266	1.695	1.356	0.332	-	0.265	1.923	1.538
Br	0.354	-	0.265	2.393	1.538	0.355	-	0.284	2.402	1.922	0.356	-	0.285	2.449	1.959
I	0.386	-	0.285	3.134	1.959	0.386	-	0.309	3.187	2.550	0.388	-	0.311	3.106	2.484
Oxygen															
OC	0.301	0.329	0.287	0.523	1.011	0.295	0.317	0.236	0.479	0.383	0.335	0.361	0.268	0.488	0.390
O=C	0.256	0.278	0.263	1.017	1.725	0.258	0.288	0.206	1.140	0.912	0.283	0.310	0.227	1.167	0.934
OH	0.282	0.299	0.287	0.871	1.011	0.275	0.299	0.220	0.923	0.738	0.308	0.320	0.247	0.843	0.674
Nitrogen															
N_amn	0.301	0.290	0.298	0.481	0.877	0.309	0.301	0.247	0.484	0.387	0.294	0.291	0.235	0.526	0.421
N_amd	0.341	0.339	0.298	0.482	0.877	0.326	0.319	0.261	0.403	0.322	0.337	0.326	0.269	0.437	0.350
Hydrogen															
HC	0.193	-	0.269	0.127	0.350	0.193	-	0.155	0.127	0.101	0.213	-	0.170	0.112	0.090
HB	0.000	-	0.170	0.000	0.090	0.000	-	0.000	0.000	0.000	0.000	-	0.000	0.000	0.000

Table S.6: Final values of the LJ parameters for the force fields calibrated using GM, LB, and WH combination rules (considering the main calibration replica). This table is similar to Main Article Tab. 4 (see the corresponding caption for details).

Atom type (EE-type)	LJ-type	GM		LB		WH	
		η [e^{-1}·V]	χ [V]	η [e^{-1}·V]	χ [V]	η [e^{-1}·V]	χ [V]
Aliphatic carbon (united-)atoms							
CH0	CH0	-	-	-	-	-	-
CH1	CH1	-	-	-	-	-	-
CH2	CH2	-	-	-	-	-	-
CH3	CH3	-	-	-	-	-	-
CH4	CH4	-	-	-	-	-	-
Halogen							
F_hal	F	35.785	17.444	36.715	17.865	33.002	18.356
Cl_hal	Cl	24.045	13.035	27.483	12.490	25.194	12.495
Br_hal	Br	28.100	11.153	23.974	10.024	25.080	8.291
I_hal	I	24.876	6.476	28.390	7.833	27.515	8.532
CH0_hal	CH0	10.432	6.770	10.710	7.720	10.765	7.904
CH1_hal	CH1	10.565	6.520	10.484	6.235	10.168	6.421
CH2_hal	CH2	11.370	6.258	11.156	6.308	8.487	9.108
CH3_hal	CH3	10.267	7.323	9.578	7.564	9.213	8.786
Ether							
O_eth	OC	12.660	12.543	12.291	11.923	14.064	11.485
CH0_O_eth	CH0	9.072	7.580	8.600	7.404	10.058	7.085
CH1_O_eth	CH1	10.535	6.823	11.017	6.663	10.156	6.542
CH2_O_eth	CH2	10.532	8.226	8.700	7.893	11.105	8.000
CH3_O_eth	CH3	10.482	7.122	10.620	6.587	10.000	7.051
Aldehyde							
H_CO_ald	HC	16.097	5.826	16.202	6.023	16.681	5.866
C_ald	C=O	9.628	7.299	9.974	8.680	10.227	7.952
O_ald	O=C	12.401	9.150	11.905	9.101	13.169	10.219
Ketone							
C_ket	C=O	8.781	7.532	10.541	7.485	8.030	6.340
O_ket	O=C	12.610	10.629	14.373	8.258	13.154	10.378
Ester							
H_CO_est	HC	18.603	7.060	16.884	6.848	17.414	6.287
C_est	C=O	12.944	7.030	11.785	6.333	11.173	6.600
O_est	O=C	14.556	9.501	15.135	8.797	14.116	9.712
O_C_est	OC	10.645	7.618	12.796	9.493	11.929	9.608
CH0_O_est	CH0	9.607	6.425	9.630	7.674	8.963	6.983
CH1_O_est	CH1	9.944	7.966	10.683	8.662	10.754	8.678
CH2_O_est	CH2	11.405	7.776	11.523	7.960	10.513	8.461
CH3_O_est	CH3	10.006	8.126	10.334	8.436	10.065	8.797
Alcohol							
H_ol	HB	14.241	5.537	13.905	6.127	14.137	5.317
O_ol	OH	12.027	10.482	12.056	10.996	12.476	11.223
CH0_O_ol	CH0	10.990	8.001	10.990	8.095	9.531	8.094
CH1_O_ol	CH1	10.107	7.601	12.282	7.518	11.963	7.456
CH2_O_ol	CH2	10.476	7.388	10.948	7.375	10.641	7.232
CH3_O_ol	CH3	11.938	7.179	8.201	7.873	11.505	6.934
Carboxylic acid							
H_CO_acd	HC	14.230	5.790	16.609	6.932	17.608	6.598
C_acd	C=O	9.805	7.819	10.078	6.475	10.470	6.862
O_acd	O=C	14.953	8.928	13.833	10.200	12.360	10.551
H_O_acd	HB	15.772	5.316	14.980	5.562	14.036	5.355
O_H_acd	OH	11.169	10.631	13.607	11.331	11.250	11.127
H_N_amn	HB	14.113	5.333	14.585	5.664	13.793	5.259
Amine							
N_amn	N_amn	10.816	8.808	9.747	9.272	10.204	8.806
CH0_N_amn	CH0	10.703	8.922	9.140	8.333	11.818	9.001
CH1_N_amn	CH1	10.075	8.424	11.675	8.551	9.656	7.725
CH2_N_amn	CH2	9.875	8.342	13.052	7.673	11.463	7.121
CH3_N_amn	CH3	14.058	8.111	10.727	7.365	13.117	6.448
Amide							
H_N_amd	HC	16.401	5.916	14.597	6.018	17.320	5.437
C_amd	C=O	10.268	5.964	9.667	6.996	10.356	5.688
O_amd	O=C	13.463	10.989	12.619	11.314	11.846	10.279
N_amd	N_amd	9.521	10.121	10.106	9.943	9.282	10.482
CH0_N_amd	CH0	(9.740)	(6.872)	(9.984)	(7.278)	(10.118)	(7.011)
CH1_N_amd	CH1	8.560	5.397	10.395	7.937	8.958	6.574
CH2_N_amd	CH2	10.298	7.927	8.725	7.179	11.451	7.056
CH3_N_amd	CH3	10.362	7.291	10.833	6.717	9.945	7.402

Table S.7: Final values of the EE parameters for the force fields calibrated using GM, LB, and WH combination rules (considering the main calibration replica). For the EE-type CH0_N_amd, no representative molecule is available at all, and the corresponding EE parameters η and χ are estimated using the average values over the three other CHn_N_amd types. This table is similar to Main Article Tab. 3 (see the corresponding caption for details).

S.6 Initial Values of the Non-Bonded Parameters

The initial values of the non-bonded interaction parameters, used to start the optimization, are reported in Tab. S.8 for the 17 LJ-types and in Tab. S.9 for the 56 EE-types.

LJ type	σ	$\tilde{\sigma}$ [nm]	σ^*	ϵ	ϵ^* [kJ·mol^{-1}]	Usage
Carbon						
CH0	0.664	-	0.336	0.007	0.406	CH_0 carbon atom (methanetetryl group)
CH1	0.502	-	0.330	0.095	0.567	CH_1 carbon united-atom (methanetriyl group)
CH2	0.407	-	0.316	0.411	1.176	CH_2 carbon united-atom (methylene group)
CH3	0.375	-	0.309	0.867	1.946	CH_3 carbon united-atom (methyl group)
CH4	0.371	-	-	1.264	-	CH_4 carbon united-atom (methane group)
C=O	0.345	-	0.336	0.326	0.406	carbonyl carbon atom
Halogen						
F	0.274	-	0.274	0.697	0.697	fluorine atom
Cl	0.339	-	0.339	1.437	1.437	chlorine atom
Br	0.358	-	0.358	2.026	2.026	bromine atom
I	0.382	-	0.382	2.743	2.743	iodine atom
Oxygen						
OC	0.301	0.309	0.287	0.359	1.011	ether oxygen atom
O=C	0.296	0.313	0.263	0.857	1.725	carbonyl oxygen atom
OH	0.287	0.298	0.287	0.776	1.011	hydoxyl oxygen atom
Nitrogen						
N_amn	0.312	0.308	0.298	0.572	0.877	amine nitrogen atom
N_amd	0.320	0.312	0.298	0.429	0.877	amide nitrogen atom
Hydrogen						
HC	0.223	-	0.223	0.119	0.119	carbonyl-linked hydrogen atom
HB	0.000	-	0.000	0.000	0.000	oxygen- or nitrogen-linked hydrogen atom

Table S.8: Initial values of the LJ parameters used to start the optimization. This table is similar to Main Article Tab. 4 (see the corresponding caption for details).

Atom type (EE-type)	LJ-type	η [$e^{-1}\cdot V$]	χ [V]	Usage
Aliphatic carbon (united-)atoms				
CH0	CH0	-	-	CH_0 carbon atom (methanetetryl group)
CH1	CH1	-	-	CH_1 carbon united-atom (methanetriyl group)
CH2	CH2	-	-	CH_2 carbon united-atom (methylene group)
CH3	CH3	-	-	CH_3 carbon united-atom (methyl group)
CH4	CH4	-	-	CH_4 carbon united-atom (methane group)
Halogen				
F_hal	F	34.970	17.800	fluorine atom
Cl_hal	Cl	27.500	9.810	chlorine atom
Br_hal	Br	26.000	7.360	bromine atom
I_hal	I	25.000	7.000	iodine atom
CH0_hal	CH0	10.490	7.660	halogenated CH_0 atom
CH1_hal	CH1	10.490	7.660	halogenated CH_1 united-atom
CH2_hal	CH2	10.490	7.660	halogenated CH_2 united-atom
CH3_hal	CH3	10.490	7.660	halogenated CH_3 united-atom
Ether				
O_eth	OC	13.280	9.530	ether oxygen atom
CH0_O_eth	CH0	10.490	7.660	alkoxylated CH_0 atom
CH1_O_eth	CH1	10.490	7.660	alkoxylated CH_1 united-atom
CH2_O_eth	CH2	10.490	7.660	alkoxylated CH_2 united-atom
CH3_O_eth	CH3	10.490	7.660	alkoxylated CH_3 united-atom
Aldehyde				
H_CO_ald	HC	15.710	6.180	aldehyde hydrogen atom
C_ald	C=O	10.490	7.660	aldehyde carbonyl carbon atom
O_ald	O=C	13.280	9.530	aldehyde carbonyl oxygen atom
Ketone				
C_ket	C=O	10.490	7.660	ketone carbonyl carbon atom
O_ket	O=C	13.280	9.530	ketone carbonyl oxygen atom
Ester				
H_CO_est	HC	15.710	6.180	formate ester hydrogen atom
C_est	C=O	10.490	7.660	ester carbonyl carbon atom
O_est	O=C	13.280	9.530	ester carbonyl oxygen atom
O_C_est	OC	13.280	9.530	ester acylated oxygen atom
CH0_O_est	CH0	10.490	7.660	ester oxygen-linked CH_0 atom
CH1_O_est	CH1	10.490	7.660	ester oxygen-linked CH_1 united-atom
CH2_O_est	CH2	10.490	7.660	ester oxygen-linked CH_2 united-atom
CH3_O_est	CH3	10.490	7.660	ester oxygen-linked CH_3 united-atom
Alcohol				
H_ol	HB	15.710	6.180	hydroxyl hydrogen atom
O_ol	OH	13.280	9.530	hydoxyl oxygen atom
CH0_O_ol	CH0	10.490	7.660	hydroxylated CH_0 atom
CH1_O_ol	CH1	10.490	7.660	hydroxylated CH_1 united-atom
CH2_O_ol	CH2	10.490	7.660	hydroxylated CH_2 united-atom
CH3_O_ol	CH3	10.490	7.660	hydroxylated CH_3 united-atom
Carboxylic acid				
H_CO_acd	HC	15.710	6.180	formic acid hydrogen atom
C_acd	C=O	10.490	7.660	carboxylic acid carbonyl carbon atom
O_acd	O=C	13.280	9.530	carboxylic acid carbonyl oxygen atom
H_O_acd	HB	15.710	6.180	carboxylic acid hydroxyl hydrogen atom
O_H_acd	OH	13.280	9.530	carboxylic acid hydroxyl oxygen atom
Amine				
H_N_amn	HB	15.710	6.180	amine hydrogen atom
N_amn	N_amn	10.810	8.650	amine nitrogen atom
CH0_N_amn	CH0	10.490	7.660	aminated CH_0 atom
CH1_N_amn	CH1	10.490	7.660	aminated CH_1 united-atom
CH2_N_amn	CH2	10.490	7.660	aminated CH_2 united-atom
CH3_N_amn	CH3	10.490	7.660	aminated CH_3 united-atom
Amide				
H_N_amd	HB	15.710	6.180	amide nitrogen-linked hydrogen atom
C_amd	C=O	10.490	7.660	amide carbonyl carbon atom
O_amd	O=C	13.280	9.530	amide carbonyl oxygen atom
N_amd	N_amd	10.810	8.650	amide acylated nitrogen atom
CH0_N_amd	CH0	10.490	7.660	amide nitrogen-linked CH_0 atom (estimated)
CH1_N_amd	CH1	10.490	7.660	amide nitrogen-linked CH_1 united-atom
CH2_N_amd	CH2	10.490	7.660	amide nitrogen-linked CH_2 united-atom
CH3_N_amd	CH3	10.490	7.660	amide nitrogen-linked CH_3 united-atom

Table S.9: Initial values of the EE parameters used to start the optimization. This table is similar to Main Article Tab. 3 (see the corresponding caption for details).

S.7 Observable-to-parameter Ratio

In this section, the observable-to-parameter ratio is analyzed separately for the different LJ- and EE-types of the force field, see Tabs. 4 and 3 in the Main Article. A favorable observable-to-parameter ratio is observed in most cases, except for EE-types occurring in a single molecule. These are the CH3 united-atom of EE-type CH3_O_ol (only found in methanol), the carbonyl-bound hydrogen atom of EE-type H_CO_acd (only found in formic acid) and the CH1 united-atom of EE-type CH1_N_amd (only found in N-isopropyl acetamide). In one case, for the EE-type CH0_N_amd, no representative molecule is available at all, and the corresponding EE parameters η and χ are estimated using the average values over the three other CHn_N_amd types.

LJ-type	N^{cal}	N_{ρ}^{cal}	$N_{\Delta H}^{\text{cal}}$
Carbon			
CH0	396	363	149
CH1	843	784	259
CH2	1341	1251	498
CH3	1385	1307	541
CH4	1	3	3
C=O	528	500	165
Halogen			
F	79	83	40
Cl	142	142	49
Br	92	95	27
I	37	36	19
Oxygen			
OC	440	413	154
O=C	528	500	165
OH	507	496	121
Nitrogen			
N_amn	143	127	64
N_amd	19	15	10
Hydrogen			
HC	63	64	22

Table S.10: Representation of the different LJ-types in the molecules of the calibration set, including availability of experimental ρ_{liq} and ΔH_{vap} values. The entries N^{cal}, N_{ρ}^{cal} and $N_{\Delta H}^{\text{cal}}$ provide numbers of molecules, of ρ_{liq} values and of ΔH_{vap} values, respectively, in the calibration set ($N_{\text{iso}}^{\text{cal}}$ = 1516 compounds depicted in Fig. S.2). The values reported correspond to the number of molecules including at least one occurrence of the specific LJ-type. The LJ-types are those listed in Tab. 4 in the Main Article, except for the LJ-type HB (not subject to optimization).

Atom type (EE-type)	N^{cal}	N_{ρ}^{cal}	$N_{\Delta H}^{\mathrm{cal}}$
Haloakane			
F_hal	79	83	40
Cl_hal	142	142	49
Br_hal	92	95	27
I_hal	37	36	19
CH0_hal	65	70	25
CH1_hal	96	94	29
CH2_hal	173	167	67
CH3_hal	4	7	3
Ether			
O_eth	161	154	60
CH0_eth	20	16	15
CH1_eth	42	42	9
CH2_eth	137	129	50
CH3_eth	53	52	20
Aldehyde			
H_ald	46	47	13
C_ald	46	47	13
O_ald	46	47	13
Ketone			
C_ket	129	126	27
O_ket	129	126	27
Ester			
H_est	16	16	8
C_est	287	265	98
O_est	287	265	98
O_C_est	287	265	98
CH0_O_est	12	9	5
CH1_O_est	37	35	5
CH2_O_est	178	170	56
CH3_O_est	62	53	32
Alcohol			
H_ol	436	425	103
O_ol	436	425	103
CH0_O_ol	122	122	13
CH1_O_ol	167	161	39
CH2_O_ol	202	197	55
CH3_O_ol	1	1	1
Carboxylic acid			
H_CO_acd	1	1	1
C_acd	73	73	18
O_acd	73	73	18
H_O_acd	73	73	18
O_H_acd	73	73	18
Amine			
H_N_amn	99	89	46
N_amd	143	127	64
CH0_N_amn	10	10	5
CH1_N_amn	33	28	12
CH2_N_amn	112	93	51
CH3_N_amn	51	49	19
Amide			
H_N_amd	11	7	8
C_amd	19	15	10
O_amd	19	15	10
N_amd	19	15	10
CH0_N_amd	0	0	0
CH1_N_amd	1	0	1
CH2_N_amd	7	6	4
CH3_N_amd	7	6	3

Table S.11: Representation of the different EE-types (or, equivalently, atom types) in the molecules of the calibration set, including availability of experimental ρ_{liq} and ΔH_{vap} values. The entries N^{cal}, N_{ρ}^{cal} and $N_{\Delta H}^{\mathrm{cal}}$ provide numbers of molecules, of ρ_{liq} values and of ΔH_{vap} values, respectively, in the calibration set ($N_{\mathrm{iso}}^{\mathrm{cal}} = 1516$ compounds depicted in Fig. S.2). The values reported correspond to the number of molecules including at least one occurrence of the specific EE-type.

S.8 Compounds that Vaporized

Sets of compounds that vaporized during the main optimization runs using the GM (Fig. S.4), LB (Fig. S.5), or WH (Fig. S.6) combination rules.

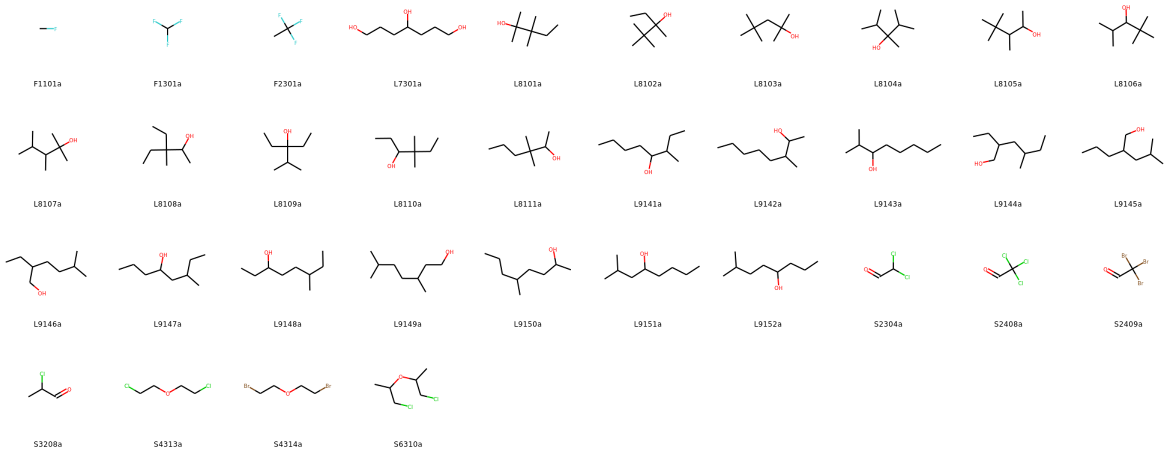

Figure S.4: Chemical structures of the 34 molecules that vaporized when using GM.

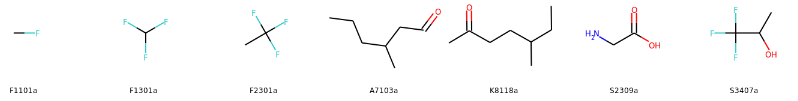

Figure S.5: Chemical structures of the 7 molecules that vaporized when using LB.

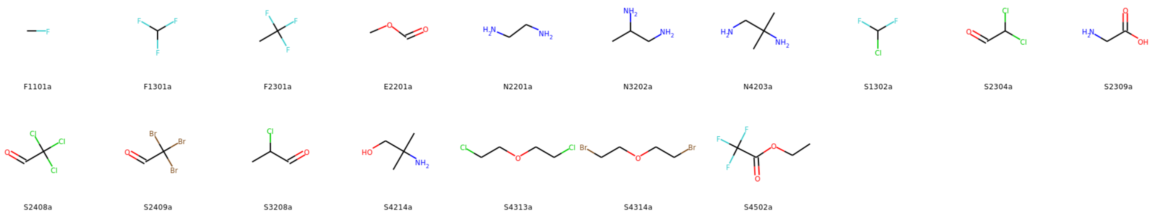

Figure S.6: Chemical structures of the 17 molecules that vaporized when using WH.

S.9 Partial Charges on Esters with the GM rule

The atomic partial charges of specific ester atoms with the GM rule are shown in Fig. S.7.

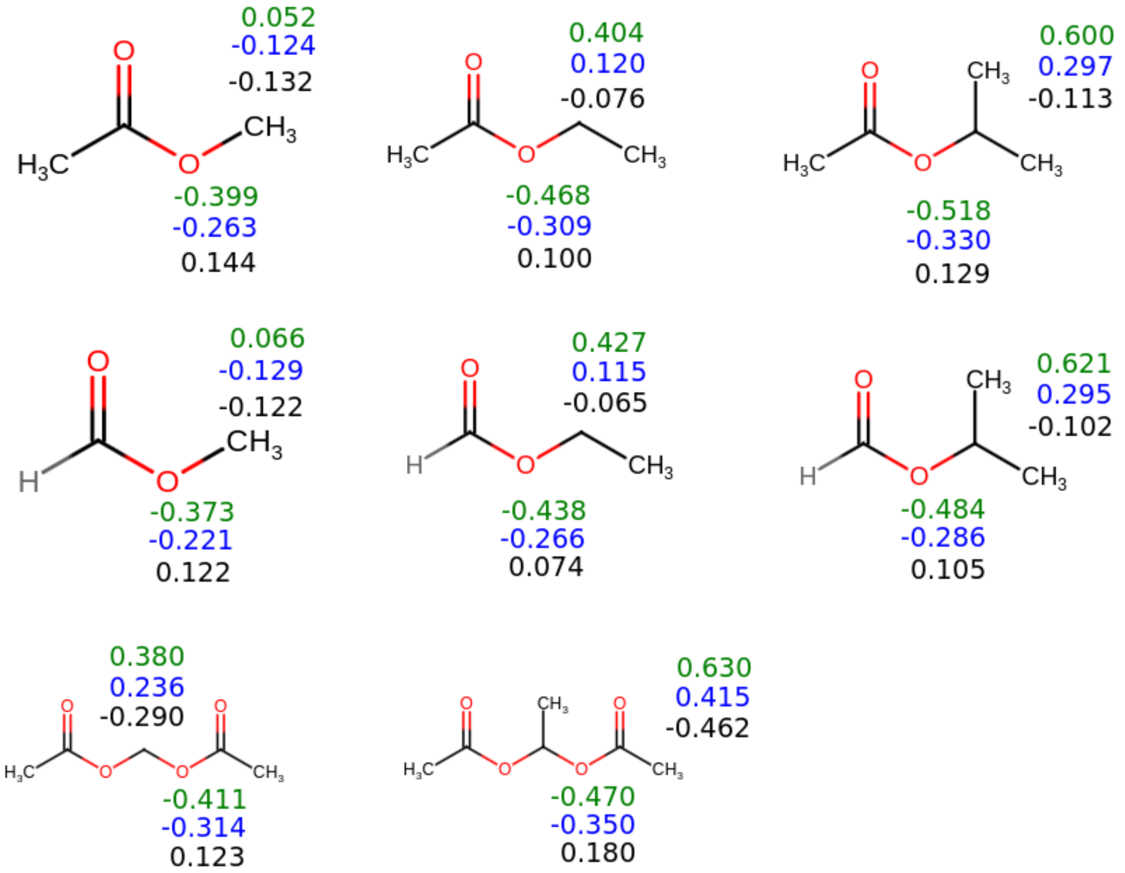

Figure S.7: Atomic partial charges on the oxygen and carbon atoms of esters obtained from the optimized force field with the GM combination rule (black), from DDEC charge fitting[114] (blue) and from RESP charge fitting[115] (green). The charges are reported in units of elementary charge e. For DDEC and RESP, the fitting is based on QM calculations at the TPSSh/def2-TZVP level[116–118] in vacuum.

S.10 Comparison of Experimental and Simulated Properties

In this section, we provide a detailed comparison between the experimental properties and their values calculated using the final force-field parameters for each of the three combination rules.

The first two properties are the liquid density ρ_{liq} and the vaporization enthalpy ΔH_{vap}. The experimental (exp) and simulated (sim) values, deviation (dev) and percentage error (err) are reported in Tabs. S.12, S.13, and S.14, for the GM, LB, and WH combination rules, respectively, considering the $N_{\text{sim}}^{\text{cal}} = 1607$ compounds/P,T-points (for $N_{\text{iso}}^{\text{cal}} = 1516$ compounds) considered in the simulations (Tab. S.2). Simulation results with errors larger than 80.0 kg·m^{-3} for ρ_{liq} and/or larger than 8.0 kJ·mol^{-1} for ΔH_{vap} (114, 68, and 101 simulations concerning 105, 61, and 91 molecules for GM, LB, and WH, respectively) are marked as outliers, and are shown in Sec. S.11.

For further comparisons in terms of the surface-tension coefficient γ, the static relative dielectric permittivity ϵ, and the self-diffusion coefficient D, considering the 66 compounds, the results are reported in Tabs. S.15-S.17.

Table S.12: Experimental and simulated properties of the 1607 compounds/P, T-points considered in the simulations using GM combination rules.

n_{sim}	n_{iso}	Code	Outlier	T_m [K]	T_b [K]	T [K]	P [bar]	$\rho_{\text{liq}}[\text{kg}\cdot\text{m}^{-3}]$ exp	sim	dev	err [%]	$\Delta H_{\text{vap}}[\text{kJ}\cdot\text{mol}^{-1}]$ exp	sim	dev	err [%]
1	1	A1001a		111.7	190.6	91	0.12	451.0	456.9	5.9	1.3	9.2	9.0	-0.2	-2.3
2	1	A1001b		111.7	190.6	102	0.41	436.1	441.5	5.5	1.3	8.8	8.8	-0.1	-1.0
3	1	A1001c		111.7	190.6	112	1.01	422.0	427.1	5.1	1.2	-	-	-	-
4	1	A1001d		111.7	190.6	112	1.01	-	-	-	-	8.5	8.5	0.0	0.6
5	2	A2001a		184.6	305.3	189	1.26	526.6	518.1	-8.5	-1.6	-	-	-	-
6	2	A2001b		184.6	305.3	266	20.88	409.4	394.8	-14.6	-3.6	9.7	11.2	1.5	14.9
7	2	A2001c		184.6	305.3	185	1.01	-	-	-	-	14.8	14.0	-0.8	-5.2
8	3	A3001a		231.1	369.8	230	1.0	582.1	572.5	-9.6	-1.6	18.8	18.2	-0.6	-3.4
9	3	A3001b		231.1	369.8	298	9.78	493.1	485.4	-7.7	-1.6	-	-	-	-
10	4	A4001a		261.4	407.8	260	19.85	597.9	597.9	-0.0	-0.0	-	-	-	-
11	4	A4001b		261.4	407.8	300	25.33	553.0	556.2	3.2	0.6	-	-	-	-
12	4	A4001c		261.4	407.8	265	1.0	-	-	-	-	22.4	21.0	-1.4	-6.3
13	5	A4002a		272.6	425.1	273	1.0	601.0	596.0	-5.0	-0.8	-	-	-	-
14	5	A4002b		272.6	425.1	300	9.93	572.5	568.4	-4.0	-0.7	-	-	-	-
15	5	A4002c		272.6	425.1	264	1.0	-	-	-	-	23.1	22.4	-0.7	-3.2
16	6	A5001a		282.6	433.8	282	0.99	603.7	614.0	10.3	1.7	23.6	23.1	-0.5	-2.3
17	6	A5001b		282.6	433.8	298	1.76	586.0	599.1	13.2	2.2	-	-	-	-
18	7	A5002a		301.0	460.4	298	0.92	616.0	615.4	-0.7	-0.1	25.5	25.0	-0.5	-1.9
19	8	A5003a		309.2	469.7	298	1.01	621.1	621.2	0.1	0.0	26.6	26.2	-0.4	-1.6
20	9	A6001a		322.9	489.0	298	1.01	644.4	653.2	8.8	1.4	28.8	27.7	-1.1	-3.8
21	10	A6002a		331.1	500.0	298	1.01	658.6	658.8	0.2	0.0	30.0	29.1	-0.9	-3.1
22	11	A6003a		336.4	504.4	298	1.01	659.9	659.1	-0.9	-0.1	30.9	29.9	-1.0	-3.2
23	12	A6004a		333.4	497.7	298	1.01	648.5	654.1	5.6	0.9	30.4	30.0	-0.4	-1.4
24	13	A6005a		341.9	507.6	298	1.0	656.1	657.5	1.4	0.2	31.8	31.2	-0.6	-1.8
25	14	A7001a		354.0	531.2	298	0.13	687.1	698.7	11.6	1.7	32.4	32.3	-0.1	-0.2
26	15	A7002a		359.2	536.4	298	0.11	686.9	696.0	9.2	1.3	34.3	32.8	-1.5	-4.4
27	16	A7003a		352.3	520.5	298	0.14	673.1	684.1	11.0	1.6	33.4	32.7	-0.7	-2.0
28	17	A7004a		362.9	537.3	298	0.09	691.1	692.1	1.0	0.2	35.0	34.2	-0.8	-2.2
29	18	A7005a		353.6	519.8	298	0.13	667.9	680.5	12.6	1.9	33.6	33.7	0.0	0.1
30	19	A7006a		366.6	527.0	298	0.08	695.1	691.0	-4.1	-0.6	35.2	34.7	-0.5	-1.5
31	20	A7007a		365.0	535.2	298	0.08	683.9	687.1	3.1	0.5	35.4	34.8	-0.6	-1.6
32	21	A7008a		363.2	530.4	298	0.09	674.0	681.8	7.8	1.2	35.2	34.8	-0.4	-1.0
33	22	A7009a		371.6	540.2	298	0.06	682.0	684.6	2.6	0.4	36.5	36.1	-0.4	-1.0
34	23	A8001a		387.9	573.5	298	0.04	722.1	731.7	9.6	1.3	37.6	37.5	-0.1	-0.3
35	24	A8002a		383.0	563.5	298	0.04	712.0	725.0	13.0	1.8	-	-	-	-
36	25	A8003a		372.4	543.8	298	1.01	688.0	705.6	17.6	2.6	35.2	36.2	1.0	2.7
37	26	A8004a		386.6	566.4	298	0.04	715.9	721.6	5.7	0.8	37.7	38.1	0.4	1.2
38	27	A8005a		391.4	576.5	298	0.03	724.0	728.7	4.7	0.7	38.0	37.9	-0.1	-0.4
39	28	A8006a		385.1	562.0	298	0.04	707.1	717.2	10.1	1.4	37.5	37.7	0.2	0.6
40	29	A8007a		380.0	549.8	298	0.05	692.1	705.6	13.5	2.0	37.3	37.5	0.2	0.6
41	30	A8008a		388.8	567.0	298	0.03	711.1	716.6	5.6	0.8	38.5	38.7	0.2	0.5
42	31	A8009a		390.9	568.8	298	0.03	715.9	717.8	1.9	0.3	39.0	38.8	-0.2	-0.6
43	32	A8010a		388.8	563.5	298	0.03	708.1	713.3	5.3	0.7	38.8	38.8	0.0	0.1
44	33	A8011a		382.6	553.5	298	1.0	696.4	706.7	10.3	1.5	37.8	38.3	0.5	1.3
45	34	A8012a		382.3	550.0	298	0.04	690.0	699.7	9.6	1.4	37.9	38.3	0.4	1.1
46	35	A8013a		391.7	565.5	298	0.03	710.1	711.6	1.4	0.2	39.7	39.3	-0.4	-1.0

Table S.12 – Comparison of experimental and simulated properties using GM combination rules (continued).

n_{sim}	n_{iso}	Code	Outlier	T_m [K]	T_b [K]	T [K]	P [bar]	ρ_{liq}[kg·m^{-3}] exp	sim	dev	err [%]	ΔH_{vap}[kJ·mol^{-1}] exp	sim	dev	err [%]
47	36	A8014a		390.9	561.7	298	0.03	713.0	707.8	-5.2	-0.7	39.7	39.7	0.0	0.0
48	37	A8015a		392.1	563.7	298	0.03	702.0	707.7	5.7	0.8	39.9	39.7	-0.1	-0.3
49	38	A8016a		390.8	559.6	298	0.03	696.0	702.9	6.8	1.0	39.7	39.8	0.1	0.3
50	39	A8017a		398.8	568.7	298	1.01	698.9	705.7	6.8	1.0	41.0	41.1	0.1	0.3
51	40	A9001a		413.4	607.5	298	1.0	753.0	768.7	15.7	2.1	41.2	41.4	0.2	0.5
52	41	A9002a		395.4	574.6	298	0.03	716.1	728.9	12.9	1.8	38.5	38.9	0.4	1.1
53	42	A9003a		414.7	607.5	301	0.01	733.0	759.5	26.6	3.6	-	-	-	-
54	42	A9003b		414.7	607.5	298	1.0	-	-	-	-	41.8	47.3	5.5	13.0
55	43	A9004a		406.2	592.6	298	0.02	735.1	747.3	12.1	1.6	40.8	40.8	0.0	0.1
56	44	A9005a		417.9	582.1	298	1.0	750.8	759.4	8.6	1.2	42.7	42.3	-0.4	-0.8
57	45	A9006a		413.6	582.1	298	1.0	741.4	752.7	11.3	1.5	42.2	42.3	0.1	0.2
58	46	A9007a		410.8	582.1	298	1.0	734.5	747.3	12.8	1.7	42.1	42.3	0.2	0.5
59	47	A9008a		407.0	582.1	298	0.02	731.1	743.0	11.9	1.6	41.7	41.3	-0.4	-1.1
60	48	A9009a		406.8	582.1	298	0.02	725.1	741.5	16.4	2.3	41.7	42.0	0.3	0.8
61	49	A9010a		403.8	582.1	298	0.02	720.1	733.8	13.7	1.9	41.1	41.0	-0.1	-0.3
62	50	A9011a		399.7	582.1	298	0.02	713.1	726.7	13.7	1.9	40.7	40.7	-0.0	-0.1
63	51	A9012a		397.2	569.8	298	0.02	707.1	719.6	12.4	1.8	40.2	40.9	0.7	1.8
64	52	A9013a		409.9	582.1	298	0.01	733.9	739.2	5.3	0.7	42.3	42.2	-0.1	-0.2
65	53	A9014a		412.2	582.1	298	1.0	735.4	741.6	6.2	0.8	42.7	42.9	0.2	0.4
66	54	A9015a		404.5	582.1	298	0.02	718.1	727.4	9.3	1.3	41.4	41.8	0.4	0.9
67	55	A9016a		419.3	610.0	308	0.03	727.9	747.4	19.5	2.7	-	-	-	-
68	55	A9016b		419.3	610.0	298	1.0	-	-	-	-	42.6	42.6	-0.0	-0.0
69	56	A9017a		413.8	582.1	298	1.0	737.1	744.6	7.5	1.0	42.9	42.7	-0.2	-0.4
70	57	A9018a		408.4	582.1	298	0.01	721.0	734.6	13.6	1.9	42.2	42.6	0.4	0.9
71	58	A9019a		410.4	582.1	306	0.01	715.9	727.7	11.8	1.6	-	-	-	-
72	58	A9019b		410.4	582.1	298	1.0	-	-	-	-	42.6	42.5	-0.1	-0.2
73	59	A9020a		405.8	576.7	298	0.02	707.1	722.4	15.2	2.2	42.3	42.4	0.1	0.1
74	60	A9021a		413.6	582.1	303	0.01	732.6	733.3	0.7	0.1	-	-	-	-
75	60	A9021b		413.6	582.1	298	1.0	-	-	-	-	43.6	43.1	-0.5	-1.1
76	61	A9022a		411.2	582.1	300	0.01	727.4	731.3	3.9	0.5	-	-	-	-
77	61	A9022b		411.2	582.1	298	1.0	-	-	-	-	43.2	43.1	-0.1	-0.2
78	62	A9023a		413.8	582.1	302	0.01	723.9	731.0	7.1	1.0	-	-	-	-
79	62	A9023b		413.8	582.1	298	1.0	-	-	-	-	43.6	43.4	-0.2	-0.4
80	63	A9024a		413.7	582.1	303	0.01	717.9	725.3	7.4	1.0	-	-	-	-
81	63	A9024b		413.7	582.1	298	1.0	-	-	-	-	43.6	43.5	-0.1	-0.1
82	64	A9025a		407.0	582.1	298	0.01	719.0	726.1	7.1	1.0	42.9	42.5	-0.4	-1.0
83	65	A9026a		409.2	582.1	300	0.01	717.9	725.8	7.8	1.1	43.3	42.8	-0.5	-1.1
84	66	A9027a		406.1	582.1	298	0.01	711.1	723.7	12.6	1.8	42.9	43.2	0.3	0.6
85	67	A9028a		409.2	582.1	300	0.01	711.8	719.5	7.8	1.1	43.3	42.9	-0.4	-0.8
86	68	A9029a		408.4	582.1	298	0.01	705.9	716.8	10.9	1.5	43.3	43.2	-0.1	-0.1
87	69	A9030a		414.4	582.1	298	1.0	724.1	728.2	4.1	0.6	44.1	44.1	-0.0	-0.0
88	70	A9031a		416.4	582.1	298	1.0	722.5	728.0	5.5	0.8	44.5	44.2	-0.3	-0.8
89	71	A9032a		415.6	582.1	298	1.0	716.0	724.8	8.8	1.2	44.5	44.6	0.1	0.2
90	72	A9033a		417.4	582.1	298	1.0	717.0	723.9	6.9	1.0	44.9	44.6	-0.3	-0.7
91	73	A9034a		416.4	582.8	298	1.0	709.5	719.9	10.4	1.5	-	-	-	-
92	73	A9034b		416.4	582.8	305	0.01	-	-	-	-	43.8	44.4	0.5	1.2
93	74	A9035a		424.0	594.6	298	1.01	713.7	722.5	8.8	1.2	46.4	46.2	-0.2	-0.4
94	75	A0001a		439.2	606.6	298	1.0	776.7	797.3	20.6	2.7	45.2	45.0	-0.2	-0.4

Table S.12 – Comparison of experimental and simulated properties using GM combination rules (continued).

n_{sim}	n_{iso}	Code	Outlier	T_m [K]	T_b [K]	T [K]	P [bar]	ρ_{liq}[kg·m^{-3}] exp	sim	dev	err [%]	ΔH_{vap}[kJ·mol^{-1}] exp	sim	dev	err [%]
95	76	A0002a		432.4	606.6	298	1.0	763.6	780.7	17.1	2.2	43.5	44.9	1.4	3.2
96	77	A0003a		442.7	606.6	320	0.01	761.5	773.9	12.4	1.6	-	-	-	-
97	77	A0003b		442.7	606.6	298	1.0	-	-	-	-	46.0	46.0	0.0	0.0
98	78	A0004a		443.2	606.6	298	1.0	778.9	791.7	12.8	1.6	42.3	46.6	4.3	10.1
99	79	A0005a		433.5	623.0	298	1.0	760.9	780.1	19.2	2.5	45.2	46.2	1.0	2.2
100	80	A0006a		427.0	606.6	298	1.0	-	-	-	-	43.5	43.6	0.1	0.2
101	81	A0007a		410.6	581.4	298	1.0	714.8	736.5	21.7	3.0	43.5	43.1	-0.4	-0.9
102	82	A0008a		442.6	606.6	298	1.0	-	-	-	-	46.4	47.6	1.2	2.7
103	83	A0009a		437.8	606.6	298	1.0	-	-	-	-	46.4	47.7	1.3	2.8
104	84	A0010a		434.8	606.6	298	1.0	-	-	-	-	46.0	46.1	0.1	0.2
105	85	A0011a		426.3	606.6	298	1.0	-	-	-	-	45.2	44.8	-0.4	-0.8
106	86	A0012a		428.5	606.6	298	1.0	-	-	-	-	44.8	45.1	0.3	0.6
107	87	A0013a		432.0	606.6	298	1.0	-	-	-	-	45.6	45.4	-0.2	-0.5
108	88	A0014a		421.6	606.6	298	1.0	-	-	-	-	45.2	45.2	-0.0	-0.1
109	89	A0015a		421.0	606.6	298	1.0	-	-	-	-	44.4	44.4	-0.0	-0.0
110	90	A0016a		430.2	606.6	298	1.0	754.5	759.9	5.4	0.7	45.6	46.1	0.5	1.1
111	91	A0017a		429.4	606.6	298	1.0	-	-	-	-	46.0	46.7	0.7	1.5
112	92	A0018a		442.9	606.6	298	1.0	-	-	-	-	47.3	46.6	-0.7	-1.5
113	93	A0019a		435.3	606.6	298	1.0	-	-	-	-	46.4	46.9	0.5	1.0
114	94	A0020a		436.9	606.6	298	1.0	-	-	-	-	46.9	47.0	0.1	0.1
115	95	A0021a		436.1	606.6	298	1.0	-	-	-	-	46.4	46.2	-0.2	-0.4
116	96	A0022a		434.3	606.6	298	1.0	-	-	-	-	46.4	47.1	0.7	1.5
117	97	A0023a		435.1	606.6	298	1.0	-	-	-	-	46.9	47.0	0.1	0.3
118	98	A0024a		433.4	606.6	298	1.0	-	-	-	-	46.9	47.0	0.1	0.2
119	99	A0025a		429.3	606.6	298	1.0	-	-	-	-	46.0	45.8	-0.2	-0.4
120	100	A0026a		430.8	606.6	298	1.0	-	-	-	-	46.9	46.8	-0.1	-0.3
121	101	A0027a		434.3	606.6	298	1.0	-	-	-	-	46.4	45.8	-0.6	-1.4
122	102	A0028a		428.9	609.5	313	0.01	728.1	739.3	11.2	1.5	-	-	-	-
123	102	A0028b		428.9	609.5	298	1.0	-	-	-	-	46.0	45.5	-0.5	-1.1
124	103	A0029a		424.2	606.6	298	1.0	-	-	-	-	45.2	45.8	0.6	1.3
125	104	A0030a		426.0	606.6	298	1.0	736.2	743.3	7.1	1.0	46.0	45.7	-0.3	-0.7
126	105	A0031a		420.2	606.6	298	1.0	-	-	-	-	45.2	44.7	-0.5	-1.1
127	106	A0032a		421.5	606.6	298	1.0	-	-	-	-	45.6	45.4	-0.2	-0.5
128	107	A0033a		424.0	606.6	298	1.0	-	-	-	-	46.0	45.5	-0.5	-1.2
129	108	A0034a		422.1	606.6	298	1.0	720.0	732.7	12.7	1.8	46.4	45.6	-0.8	-1.8
130	109	A0035a		433.3	606.6	298	1.0	-	-	-	-	46.9	46.6	-0.3	-0.6
131	110	A0036a		439.9	606.6	298	1.0	-	-	-	-	46.4	46.6	0.2	0.5
132	111	A0037a		434.1	606.6	298	1.0	-	-	-	-	46.9	47.1	0.2	0.4
133	112	A0038a		435.7	606.6	298	1.0	751.9	758.7	6.8	0.9	47.3	47.5	0.2	0.4
134	113	A0039a		433.1	606.6	298	1.0	-	-	-	-	47.3	47.6	0.3	0.6
135	114	A0040a		427.3	606.6	298	1.0	-	-	-	-	46.4	46.1	-0.3	-0.7
136	115	A0041a		429.7	606.6	298	1.0	-	-	-	-	46.9	46.9	0.0	0.0
137	116	A0042a		433.9	606.6	298	1.0	-	-	-	-	47.3	46.8	-0.5	-1.0
138	117	A0043a		429.2	606.6	298	1.0	-	-	-	-	47.3	46.6	-0.7	-1.4
139	118	A0044a		420.8	606.6	298	1.0	-	-	-	-	46.4	46.3	-0.1	-0.3
140	119	A0045a		439.5	606.6	298	1.0	-	-	-	-	47.3	47.0	-0.3	-0.5
141	120	A0046a		434.0	606.6	298	1.0	-	-	-	-	47.2	47.4	0.2	0.4
142	121	A0047a		437.0	606.6	298	1.0	-	-	-	-	47.7	47.3	-0.4	-0.9

Table S.12 – Comparison of experimental and simulated properties using GM combination rules (continued).

n_{sim}	n_{iso}	Code	Outlier	T_m [K]	T_b [K]	T [K]	P [bar]	ρ_{liq}[kg·m^{-3}] exp	sim	dev	err [%]	ΔH_{vap}[kJ·mol^{-1}] exp	sim	dev	err [%]
143	122	A0048a		430.7	606.6	298	1.0	-	-	-	-	48.1	47.4	-0.7	-1.5
144	123	A0049a		434.4	606.6	298	1.0	-	-	-	-	48.5	47.3	-1.2	-2.4
145	124	A0050a		430.1	606.6	298	1.0	720.8	736.7	15.9	2.2	49.0	47.3	-1.7	-3.4
146	125	A0051a		437.1	606.6	298	1.0	747.2	751.9	4.7	0.6	47.7	46.6	-1.1	-2.2
147	126	A0052a		435.4	606.6	298	1.0	-	-	-	-	47.7	47.5	-0.2	-0.3
148	127	A0053a		436.2	606.6	298	1.0	-	-	-	-	48.1	47.5	-0.6	-1.2
149	128	A0054a		432.1	606.6	298	1.0	735.4	746.0	10.6	1.4	47.3	47.9	0.6	1.2
150	129	A0055a		434.4	606.6	298	1.0	-	-	-	-	48.1	47.8	-0.3	-0.5
151	130	A0056a		435.3	606.6	298	1.0	-	-	-	-	48.5	48.1	-0.4	-0.8
152	131	A0057a		436.6	606.6	298	1.0	741.0	746.5	5.5	0.7	48.1	48.2	0.1	0.2
153	132	A0058a		437.5	606.6	293	1.0	737.7	745.7	8.0	1.1	48.1	48.7	0.6	1.3
154	133	A0059a		431.4	606.6	298	1.0	-	-	-	-	47.7	47.0	-0.7	-1.4
155	134	A0060a		429.4	606.6	298	1.0	-	-	-	-	47.3	47.2	-0.1	-0.3
156	135	A0061a		432.9	606.6	298	1.0	-	-	-	-	48.1	47.3	-0.8	-1.7
157	136	A0062a		432.6	606.6	298	1.0	-	-	-	-	48.5	47.8	-0.8	-1.5
158	137	A0063a		429.1	606.6	298	1.0	722.6	737.3	14.7	2.0	48.5	48.0	-0.5	-1.1
159	138	A0064a		434.0	606.6	298	1.0	732.4	738.4	6.0	0.8	47.3	47.7	0.4	0.9
160	139	A0065a		431.7	606.6	298	1.0	726.4	735.3	8.9	1.2	49.0	47.9	-1.1	-2.3
161	140	A0066a		433.5	606.6	293	1.0	731.3	738.6	7.3	1.0	49.3	48.3	-1.0	-2.0
162	141	A0067a		433.0	606.6	298	1.0	720.2	731.6	11.4	1.6	47.7	48.3	0.6	1.3
163	142	A0068a		430.7	606.6	298	1.0	732.1	741.1	9.0	1.2	48.5	48.9	0.4	0.8
164	143	A0069a		436.8	606.6	298	1.0	734.3	741.0	6.7	0.9	48.1	49.0	0.9	1.9
165	144	A0070a		439.7	606.6	298	1.0	735.9	740.8	4.9	0.7	49.0	49.0	0.0	0.0
166	145	A0071a		438.3	606.6	293	1.0	732.6	741.8	9.2	1.3	49.8	49.7	-0.1	-0.2
167	146	A0072a		438.9	606.6	293	1.0	732.3	741.6	9.3	1.3	49.5	49.7	0.2	0.5
168	147	A0073a		440.9	606.6	293	1.0	735.4	741.4	6.0	0.8	50.2	49.9	-0.3	-0.6
169	148	A0074a		440.1	606.6	293	1.0	728.1	737.5	9.4	1.3	51.0	50.0	-1.0	-1.9
170	149	A0075a		447.3	617.7	298	1.01	725.9	736.4	10.5	1.4	50.2	51.2	1.0	2.0
171	150	F1101a		194.8	317.4	197	1.0	876.6	845.9	-30.7	-3.5	17.1	16.0	-1.1	-6.4
172	150	F1101b	vap	194.8	317.4	298	38.39	528.3	-	-	-	0.0	-	-	-
173	150	F1101c	vap	194.8	317.4	298	1.0	574.4	-	-	-	0.0	-	-	-
174	151	F1201a		221.5	351.3	221	1.0	1213.8	1204.5	-9.3	-0.8	20.9	19.7	-1.1	-5.5
175	151	F1201b	×	221.5	351.3	298	17.62	891.6	974.7	83.1	9.3	-	-	-	-
176	151	F1201c		221.5	351.3	298	1.0	961.0	965.3	4.3	0.4	-	-	-	-
177	152	F1301a	×	191.0	299.0	191	1.0	1442.9	1787.5	344.6	23.9	16.7	19.2	2.5	15.2
178	152	F1301b	vap	191.0	299.0	298	47.1	636.7	-	-	-	0.0	-	-	-
179	152	F1301c	vap	191.0	299.0	298	1.0	666.7	-	-	-	0.0	-	-	-
180	153	F1401a	×	145.1	227.5	145	1.0	1605.2	1734.5	129.4	8.1	12.3	10.1	-2.2	-17.8
181	154	F2101a		235.4	375.3	236	1.0	817.6	791.5	-26.1	-3.2	20.7	19.8	-0.9	-4.6
182	154	F2101b		235.4	375.3	298	9.09	707.5	678.9	-28.6	-4.0	-	-	-	-
183	155	F2201a	×	247.3	386.4	250	1.0	1009.0	896.4	-112.6	-11.2	22.7	18.2	-4.5	-20.0
184	155	F2201b	×	247.3	386.4	298	6.25	907.0	759.1	-147.9	-16.3	-	-	-	-
185	156	F2301a	×	225.8	345.9	220	1.0	1182.9	871.1	-311.7	-26.4	19.2	12.2	-7.0	-36.4
186	156	F2301b	vap	225.8	345.9	298	12.92	-	-	-	-	13.1	-	-	-
187	157	F3101a		263.8	421.1	264	1.0	769.2	717.6	-51.6	-6.7	-	-	-	-
188	158	F3102a		269.9	421.1	271	1.0	781.8	773.2	-8.6	-1.1	-	-	-	-
189	159	F3201a		281.1	430.4	276	0.62	-	-	-	-	25.1	22.2	-3.0	-11.8
190	160	F3202a		314.4	430.4	298	1.0	1005.7	990.4	-15.3	-1.5	-	-	-	-

Table S.12 – Comparison of experimental and simulated properties using GM combination rules (continued).

n_{sim}	n_{iso}	Code	Outlier	T_m	T_b	T	P	ρ_{liq}[kg·m^{-3}]				ΔH_{vap}[kJ·mol^{-1}]			
				[K]	[K]	[K]	[bar]	exp	sim	dev	err [%]	exp	sim	dev	err [%]
191	161	F4101a		285.2	460.3	285	1.0	752.7	673.9	-78.8	-10.5	-	-	-	-
192	161	F4101b	×	285.2	460.3	298	1.39	735.3	654.4	-80.9	-11.0	-	-	-	-
193	162	F4102a		298.2	460.3	298	1.0	756.6	722.8	-33.7	-4.5	-	-	-	-
194	163	F4103a		305.6	460.3	298	1.0	770.8	764.1	-6.7	-0.9	-	-	-	-
195	164	F4201a		350.9	463.6	298	1.0	976.7	974.9	-1.8	-0.2	-	-	-	-
196	165	F4301a	×	289.9	406.6	298	1.38	1010.0	851.1	-158.9	-15.7	-	-	-	-
197	166	F5101a		317.9	494.6	298	1.0	773.7	722.7	-51.1	-6.6	-	-	-	-
198	167	F5102a		329.1	494.6	298	0.34	791.5	778.7	-12.7	-1.6	-	-	-	-
199	168	F5103a		335.9	494.6	298	1.0	784.9	782.6	-2.3	-0.3	30.9	31.4	0.4	1.4
200	169	F6101a		359.4	525.4	293	1.0	791.4	773.9	-17.5	-2.2	-	-	-	-
201	170	F6102a		364.6	525.4	298	1.0	795.8	795.9	0.1	0.0	35.6	36.1	0.5	1.5
202	171	F6201a		-	-	293	1.0	888.2	836.0	-52.2	-5.9	-	-	-	-
203	172	F6202a		-	-	298	1.0	940.7	945.0	4.3	0.5	-	-	-	-
204	173	F7101a		391.1	553.5	298	1.0	800.9	804.9	4.0	0.5	40.8	40.8	-0.0	-0.0
205	174	F8101a		415.4	579.2	298	1.0	806.7	812.0	5.3	0.7	-	-	-	-
206	175	F9101a		438.1	603.2	348	1.0	-	-	-	-	46.8	46.9	0.1	0.1
207	176	F0101a		459.4	625.5	293	1.0	819.4	826.2	6.8	0.8	-	-	-	-
208	177	C1101a		248.9	416.2	249	1.0	1008.3	973.3	-34.9	-3.5	-	-	-	-
209	178	C1201a		312.9	510.0	298	1.0	1316.4	1324.0	7.6	0.6	28.8	29.5	0.7	2.5
210	179	C1301a		334.3	536.4	298	1.0	1479.5	1498.7	19.2	1.3	31.1	33.1	2.0	6.3
211	180	C1401a		349.8	556.4	298	1.0	1584.3	1608.2	23.9	1.5	32.4	34.9	2.5	7.7
212	181	C2101a		285.4	460.4	285	1.0	906.2	894.7	-11.5	-1.3	24.9	24.0	-0.9	-3.7
213	181	C2101b		285.4	460.4	298	1.39	890.0	875.2	-14.7	-1.7	-	-	-	-
214	182	C2201a		330.4	523.0	298	1.0	1168.1	1151.1	-17.0	-1.5	30.6	29.8	-0.8	-2.7
215	183	C2202a		356.6	561.6	298	1.0	1245.6	1241.6	-3.9	-0.3	34.4	34.9	0.5	1.3
216	184	C2301a		347.2	545.0	298	1.0	1329.3	1322.5	-6.8	-0.5	32.4	32.4	0.0	0.0
217	185	C2302a		387.0	551.3	298	1.0	1432.8	1436.1	3.3	0.2	40.1	42.1	2.0	4.9
218	186	C3101a		308.9	496.5	298	1.0	855.6	835.9	-19.7	-2.3	-	-	-	-
219	187	C3201a		369.5	573.3	298	1.0	1153.0	1142.3	-10.7	-0.9	36.2	36.1	-0.1	-0.3
220	188	C3202a		393.6	573.3	298	1.0	1180.0	1182.6	2.6	0.2	40.6	41.0	0.4	0.9
221	189	C4101a		323.8	530.2	298	1.0	836.3	821.2	-15.1	-1.8	28.6	27.2	-1.4	-5.1
222	190	C4102a		342.0	530.2	298	1.0	871.4	867.7	-3.7	-0.4	31.7	31.4	-0.3	-1.1
223	191	C4103a		341.2	520.6	298	1.01	867.5	854.5	-13.0	-1.5	31.5	30.3	-1.2	-3.7
224	192	C4104a		351.6	530.2	298	1.01	880.4	876.7	-3.7	-0.4	33.5	32.8	-0.7	-2.1
225	193	C4201a		391.1	599.9	298	1.0	1106.3	1088.1	-18.2	-1.6	-	-	-	-
226	194	C4202a		397.1	599.9	298	1.0	1111.8	1107.9	-3.9	-0.3	40.1	40.5	0.4	1.0
227	195	C4203a		427.1	599.9	298	1.0	-	-	-	-	46.4	47.4	1.0	2.1
228	196	C5101a		358.8	560.5	298	1.0	859.6	853.7	-6.0	-0.7	-	-	-	-
229	197	C5102a		373.7	560.5	298	1.0	875.0	876.7	1.7	0.2	-	-	-	-
230	198	C5103a		371.7	560.5	298	1.0	870.0	872.2	2.2	0.3	36.2	36.2	0.0	0.1
231	199	C5104a		369.7	560.5	298	1.0	866.0	859.0	-7.0	-0.8	36.0	35.1	-0.9	-2.4
232	200	C5105a		381.5	560.5	298	1.0	877.8	876.6	-1.2	-0.1	38.2	37.5	-0.7	-1.8
233	201	C5201a		453.1	624.5	298	1.0	1095.6	1098.3	2.7	0.2	51.3	51.7	0.4	0.8
234	202	C6101a		408.2	588.0	298	1.0	873.5	875.8	2.3	0.3	42.0	42.2	0.2	0.5
235	203	C6201a		-	-	298	1.0	1064.0	1076.7	12.7	1.2	-	-	-	-
236	204	C6202a		-	-	298	1.0	1044.1	1041.2	-2.9	-0.3	-	-	-	-
237	205	C6203a		477.1	647.4	298	1.01	1063.7	1069.4	5.7	0.5	-	-	-	-
238	206	C7101a		433.6	613.5	298	1.0	871.5	875.1	3.6	0.4	47.0	47.0	-0.0	-0.0

Table S.12 – Comparison of experimental and simulated properties using GM combination rules (continued).

n_{sim}	n_{iso}	Code	Outlier	T_m [K]	T_b [K]	T [K]	P [bar]	$\rho_{\mathrm{liq}}[\mathrm{kg{\cdot}m^{-3}}]$ exp	sim	dev	err [%]	$\Delta H_{\mathrm{vap}}[\mathrm{kJ{\cdot}mol^{-1}}]$ exp	sim	dev	err [%]
239	207	C8101a		456.6	637.1	298	1.0	869.4	874.4	5.0	0.6	51.4	51.7	0.3	0.6
240	208	C9101a		478.4	659.3	298	1.0	867.4	874.3	6.9	0.8	-	-	-	-
241	209	C0101a		499.0	680.1	298	1.0	865.8	873.7	7.8	0.9	64.0	61.2	-2.8	-4.3
242	210	B1101a		276.7	467.0	273	1.0	1729.8	1684.4	-45.4	-2.6	-	-	-	-
243	210	B1101b		276.7	467.0	298	2.25	1662.1	1617.9	-44.2	-2.7	-	-	-	-
244	210	B1101c		276.7	467.0	281	1.0	-	-	-	-	24.6	22.0	-2.6	-10.6
245	211	B1201a		370.1	615.9	298	0.06	2482.0	2480.1	-1.9	-0.1	37.0	37.4	0.4	1.1
246	212	B1301a		422.4	656.4	298	1.0	2877.2	2893.5	16.3	0.6	46.1	48.4	2.3	5.0
247	213	B1401a		462.6	706.8	374	1.0	2953.3	3026.5	73.2	2.5	-	-	-	-
248	213	B1401b		462.6	706.8	384	1.0	-	-	-	-	48.2	53.5	5.3	11.0
249	214	B2101a		311.5	503.8	298	1.0	1451.2	1439.8	-11.4	-0.8	-	-	-	-
250	214	B2101b		311.5	503.8	305	1.0	-	-	-	-	27.6	25.9	-1.7	-6.2
251	215	B2201a		381.1	635.8	298	1.0	2091.8	2082.4	-9.4	-0.4	-	-	-	-
252	216	B2202a		404.5	650.1	298	1.0	2169.5	2171.8	2.3	0.1	41.7	42.2	0.5	1.2
253	217	B2301a		462.1	671.9	298	1.0	2610.1	2602.8	-7.3	-0.3	-	-	-	-
254	218	B3101a		332.6	532.5	298	1.0	1301.4	1290.8	-10.6	-0.8	30.2	28.6	-1.6	-5.2
255	219	B3102a		344.1	536.9	298	1.0	1345.5	1337.1	-8.4	-0.6	31.9	30.9	-1.0	-3.1
256	220	B3201a		413.2	654.7	298	1.0	1925.0	1925.7	0.7	0.0	41.7	44.4	2.7	6.5
257	221	B3202a		440.4	654.7	298	1.0	1971.2	1964.4	-6.8	-0.3	-	-	-	-
258	222	B3301a		464.1	686.8	293	1.0	2298.5	2364.2	65.7	2.9	-	-	-	-
259	223	B3302a		474.1	686.8	293	1.0	2354.8	2353.8	-1.0	-0.0	-	-	-	-
260	224	B3303a		495.3	686.8	298	1.0	2411.0	2395.1	-16.0	-0.7	-	-	-	-
261	225	B4101a		346.4	557.6	298	1.0	1212.5	1204.9	-7.6	-0.6	31.8	30.3	-1.5	-4.6
262	226	B4102a		364.7	557.6	298	1.0	1257.1	1255.7	-1.4	-0.1	34.9	34.4	-0.5	-1.3
263	227	B4103a		364.4	557.6	298	1.0	1253.6	1244.9	-8.7	-0.7	34.8	33.6	-1.2	-3.5
264	228	B4104a		374.8	557.6	298	1.0	1268.6	1267.3	-1.3	-0.1	36.6	35.8	-0.8	-2.3
265	229	B4201a		448.1	672.7	298	1.0	1799.5	1807.9	8.4	0.5	-	-	-	-
266	230	B4202a		439.5	672.7	298	1.0	1787.0	1790.1	3.1	0.2	45.6	48.3	2.7	6.0
267	231	B4203a		448.1	672.7	293	1.0	1796.0	1794.0	-2.0	-0.1	-	-	-	-
268	232	B4301a		503.1	701.1	294	1.0	2175.3	2203.1	27.8	1.3	-	-	-	-
269	233	B4302a		493.1	701.1	298	1.0	2180.3	2225.4	45.1	2.1	-	-	-	-
270	234	B5101a		379.1	581.0	298	0.03	1193.5	1210.4	16.9	1.4	-	-	-	-
271	235	B5102a		381.1	581.0	298	0.03	1209.5	1198.6	-10.9	-0.9	-	-	-	-
272	236	B5103a		393.6	581.0	298	1.0	1214.4	1216.3	1.9	0.2	-	-	-	-
273	237	B5104a		391.8	581.0	298	1.0	1205.1	1203.3	-1.8	-0.1	-	-	-	-
274	238	B5105a		393.6	581.0	298	1.0	1200.7	1210.1	9.4	0.8	-	-	-	-
275	239	B5106a		390.6	581.0	298	1.0	1200.5	1197.8	-2.7	-0.2	38.5	38.3	-0.2	-0.5
276	240	B5107a		402.7	581.0	298	1.0	1211.4	1215.1	3.7	0.3	40.9	40.6	-0.3	-0.7
277	241	B5201a		453.1	689.9	293	1.0	1669.5	1696.5	27.0	1.6	-	-	-	-
278	242	B6101a		403.1	602.9	293	1.0	1179.2	1187.1	7.9	0.7	-	-	-	-
279	243	B6102a		414.4	602.9	298	1.0	1157.2	1166.2	9.0	0.8	-	-	-	-
280	244	B6103a		428.4	602.9	298	1.0	1168.8	1175.0	6.2	0.5	45.6	45.4	-0.2	-0.5
281	245	B7101a		452.1	623.5	298	1.0	1134.8	1143.0	8.2	0.7	50.4	50.2	-0.2	-0.3
282	246	B8101a		473.9	643.0	298	1.0	1107.7	1117.3	9.6	0.9	55.1	55.1	-0.0	-0.1
283	247	B9101a		494.6	661.6	298	1.0	1084.9	1095.8	11.0	1.0	-	-	-	-
284	248	B0101a		513.8	679.3	298	1.0	1062.5	1077.8	15.3	1.4	-	-	-	-
285	248	B0101b		513.8	679.3	398	1.0	-	-	-	-	56.6	57.4	0.8	1.4
286	249	I1101a		315.6	522.4	298	1.0	2264.5	2201.3	-63.2	-2.8	27.5	26.5	-1.0	-3.5

Table S.12 – Comparison of experimental and simulated properties using GM combination rules (continued).

n_{sim}	n_{iso}	Code	Outlier	T_m [K]	T_b [K]	T [K]	P [bar]	ρ_{liq}[kg·m^{-3}] exp	sim	dev	err [%]	ΔH_{vap}[kJ·mol^{-1}] exp	sim	dev	err [%]
287	250	I1201a	×	455.1	697.5	298	1.0	3307.8	3174.8	-133.1	-4.0	49.0	46.6	-2.4	-4.8
288	251	I2101a		345.4	562.2	298	1.0	1924.0	1910.4	-13.7	-0.7	31.7	30.7	-1.0	-3.1
289	252	I3101a		362.6	583.8	298	1.0	1694.5	1688.7	-5.8	-0.3	34.1	33.4	-0.7	-2.1
290	253	I3102a		375.6	602.3	298	1.0	1737.2	1733.5	-3.7	-0.2	36.0	35.5	-0.5	-1.5
291	254	I3201a		496.1	775.2	298	1.0	2565.1	2526.4	-38.7	-1.5	-	-	-	-
292	254	I3201b		496.1	775.2	369	0.01	-	-	-	-	53.5	52.3	-1.2	-2.3
293	255	I4101a		373.2	596.8	298	0.06	1536.0	1557.0	21.0	1.4	35.7	35.2	-0.5	-1.4
294	256	I4102a		393.6	625.0	298	0.02	1595.1	1598.8	3.7	0.2	38.8	39.0	0.2	0.6
295	257	I4103a		393.1	623.3	298	1.0	1589.0	1589.8	0.8	0.0	38.5	38.3	-0.2	-0.5
296	258	I4104a		403.7	638.8	298	1.0	1606.7	1609.4	2.7	0.2	40.3	40.3	-0.0	-0.0
297	259	I4201a		477.2	806.4	298	1.0	2349.6	2321.2	-28.3	-1.2	59.0	60.3	1.3	2.1
298	260	I5101a		407.1	670.9	293	1.0	1494.0	1524.7	30.7	2.1	-	-	-	-
299	261	I5102a		402.1	670.9	298	0.01	1486.6	1513.1	26.5	1.8	-	-	-	-
300	262	I5103a		413.1	670.9	293	1.0	1524.0	1516.4	-7.6	-0.5	-	-	-	-
301	263	I5104a		418.1	670.9	298	1.0	1505.5	1511.0	5.5	0.4	-	-	-	-
302	264	I5105a		421.4	659.1	298	1.0	1495.2	1511.1	16.0	1.1	42.2	43.8	1.6	3.9
303	265	I5106a		416.1	670.9	293	1.0	1500.9	1509.2	8.3	0.6	-	-	-	-
304	266	I5107a		430.1	671.4	298	1.0	1507.3	1516.3	9.0	0.6	44.4	45.1	0.7	1.6
305	267	I5201a		500.1	843.6	298	1.0	2173.4	2161.5	-11.9	-0.5	-	-	-	-
306	268	I6101a		441.1	697.2	293	1.0	1443.0	1453.5	10.5	0.7	-	-	-	-
307	269	I6102a		442.1	697.2	293	1.0	1419.3	1438.8	19.5	1.4	-	-	-	-
308	270	I6103a		454.5	704.4	298	1.0	1431.8	1443.4	11.6	0.8	-	-	-	-
309	270	I6103b		454.5	704.4	346	1.0	-	-	-	-	46.2	47.3	1.1	2.3
310	271	I6201a		522.5	831.8	298	1.0	2034.2	2033.7	-0.5	-0.0	-	-	-	-
311	272	I7101a		477.1	736.7	298	1.0	1371.9	1385.9	14.0	1.0	-	-	-	-
312	272	I7101b		477.1	736.7	357	0.01	-	-	-	-	48.4	51.2	2.8	5.7
313	273	I8101a		498.3	764.8	298	1.0	1326.7	1338.7	12.0	0.9	-	-	-	-
314	273	I8101b		498.3	764.8	374	0.01	-	-	-	-	50.9	54.7	3.8	7.4
315	274	I9101a		518.1	-	298	1.0	1283.6	1299.6	16.0	1.2	-	-	-	-
316	275	I0101a		-	-	298	1.0	1241.2	1259.6	18.4	1.5	-	-	-	-
317	276	I0102a		536.9	-	293	1.0	1256.7	1271.4	14.7	1.2	69.8	69.6	-0.2	-0.3
318	277	O2101a		248.3	400.1	248	1.0	735.0	736.1	1.1	0.1	21.6	25.7	4.2	19.3
319	277	O2101b		248.3	400.1	298	9.06	661.9	675.8	13.9	2.1	17.8	23.7	5.9	33.2
320	278	O3101a		280.5	437.8	273	1.0	726.0	717.6	-8.4	-1.2	-	-	-	-
321	278	O3101b		280.5	437.8	298	1.82	691.9	689.4	-2.6	-0.4	-	-	-	-
322	278	O3101c		280.5	437.8	280	1.0	-	-	-	-	31.2	27.3	-3.9	-12.6
323	279	O3201a		315.0	480.6	298	0.51	854.1	794.7	-59.4	-7.0	28.9	26.9	-2.0	-6.8
324	280	O4101a		303.9	464.5	298	1.0	709.0	701.1	-7.9	-1.1	26.4	29.0	2.6	9.7
325	281	O4102a		311.7	476.2	298	1.0	719.2	717.6	-1.6	-0.2	27.9	30.8	2.9	10.2
326	282	O4103a		307.6	466.7	298	1.0	707.8	689.7	-18.2	-2.6	27.2	27.2	0.1	0.2
327	283	O4201a		337.6	-	293	1.0	851.6	782.6	-69.0	-8.1	36.4	29.7	-6.7	-18.5
328	284	O4202a		357.2	536.1	298	1.01	861.4	859.2	-2.1	-0.2	36.8	39.9	3.1	8.3
329	285	O5101a		328.4	497.1	298	1.0	735.2	716.7	-18.6	-2.5	30.4	30.1	-0.3	-1.0
330	286	O5102a		331.7	500.0	298	1.0	727.2	730.0	2.8	0.4	-	-	-	-
331	287	O5103a		332.1	500.0	298	1.0	736.7	729.0	-7.7	-1.0	-	-	-	-
332	288	O5104a		326.1	500.0	298	1.0	717.3	711.9	-5.4	-0.8	30.0	32.2	2.2	7.3
333	289	O5105a		343.4	512.7	298	1.0	739.4	739.2	-0.2	-0.0	32.5	35.4	2.9	8.9
334	290	O5106a		337.0	500.2	298	1.0	727.0	714.1	-12.9	-1.8	31.4	31.6	0.2	0.7

Table S.12 – Comparison of experimental and simulated properties using GM combination rules (continued).

n_{sim}	n_{iso}	Code	Outlier	T_m [K]	T_b [K]	T [K]	P [bar]	ρ_{liq}[kg·m^{-3}] exp	sim	dev	err [%]	ΔH_{vap}[kJ·mol^{-1}] exp	sim	dev	err [%]
335	291	O5201a		356.1	-	298	1.01	845.1	778.3	-66.8	-7.9	37.6	30.4	-7.2	-19.3
336	292	O5202a		375.2	-	298	1.0	846.0	833.4	-12.6	-1.5	39.8	40.8	1.0	2.5
337	293	O5203a		361.1	524.0	298	1.0	825.2	784.2	-40.9	-5.0	35.7	33.9	-1.8	-5.1
338	294	O6101a		359.4	526.0	298	1.0	765.9	748.5	-17.4	-2.3	35.0	34.6	-0.4	-1.3
339	295	O6102a		345.9	526.0	298	1.0	735.2	726.8	-8.3	-1.1	33.1	34.1	1.0	3.0
340	296	O6103a		356.1	526.0	298	1.0	754.2	747.6	-6.6	-0.9	-	-	-	-
341	297	O6104a		341.4	500.1	298	1.0	718.7	722.6	3.9	0.5	32.7	34.9	2.2	6.7
342	298	O6105a		363.1	526.0	298	0.07	746.1	753.2	7.1	0.9	-	-	-	-
343	299	O6106a		363.1	526.0	298	1.0	749.0	752.1	3.1	0.4	-	-	-	-
344	300	O6107a		354.2	526.0	298	1.0	734.0	724.9	-9.2	-1.3	-	-	-	-
345	301	O6108a		364.1	526.0	298	0.06	749.9	745.6	-4.3	-0.6	-	-	-	-
346	302	O6109a		354.4	526.0	298	1.0	738.3	733.6	-4.7	-0.6	-	-	-	-
347	303	O6110a		353.1	526.0	298	1.0	732.4	728.5	-3.9	-0.5	-	-	-	-
348	304	O6111a		372.1	546.5	298	1.0	755.2	754.8	-0.4	-0.1	-	-	-	-
349	305	O6112a		365.4	526.0	298	1.0	744.7	734.1	-10.6	-1.4	36.3	36.3	-0.0	-0.0
350	306	O6113a		362.8	530.6	298	1.0	741.9	731.6	-10.4	-1.4	35.7	35.9	0.2	0.6
351	307	O6201a		-	-	293	1.0	844.6	805.7	-38.9	-4.6	-	-	-	-
352	308	O6202a		376.8	539.7	298	1.01	822.0	783.0	-39.0	-4.7	39.6	38.1	-1.5	-3.8
353	309	O6203a		-	-	298	1.0	852.9	856.7	3.8	0.4	-	-	-	-
354	310	O6204a		392.6	637.8	298	1.0	836.2	817.7	-18.5	-2.2	43.2	43.7	0.5	1.3
355	311	O6301a	×	432.9	608.0	298	1.0	939.2	930.6	-8.6	-0.9	48.0	56.5	8.5	17.6
356	312	O7101a		378.7	549.7	298	1.0	736.4	735.6	-0.8	-0.1	34.5	36.3	1.8	5.2
357	313	O7102a		375.1	546.0	298	1.0	761.8	752.7	-9.1	-1.2	38.2	38.3	0.1	0.2
358	314	O7103a		373.1	549.7	298	1.0	746.7	741.2	-5.5	-0.7	37.2	38.2	1.0	2.7
359	315	O7104a		371.2	549.7	298	1.0	734.9	735.0	0.1	0.0	-	-	-	-
360	316	O7105a		-	549.7	298	1.0	739.6	741.3	1.7	0.2	-	-	-	-
361	317	O7106a		385.6	549.7	298	1.0	752.1	746.1	-6.0	-0.8	-	-	-	-
362	318	O7107a		378.1	549.7	298	1.0	744.0	738.6	-5.4	-0.7	-	-	-	-
363	319	O7108a		-	-	298	1.0	750.1	746.1	-4.0	-0.5	-	-	-	-
364	320	O7109a		380.1	-	298	1.0	746.0	744.2	-1.8	-0.2	-	-	-	-
365	321	O7110a		398.1	-	298	1.0	766.3	767.7	1.4	0.2	-	-	-	-
366	322	O7111a		391.1	-	298	1.0	757.2	749.9	-7.3	-1.0	-	-	-	-
367	323	O7112a		390.1	-	298	1.0	754.2	747.0	-7.2	-1.0	-	-	-	-
368	324	O7201a		404.6	-	405	1.0	-	-	-	-	34.8	38.7	3.9	11.3
369	325	O7202a		387.1	-	298	1.0	868.8	794.3	-74.5	-8.6	43.9	41.9	-2.0	-4.6
370	326	O7203a		-	-	298	1.0	813.7	786.4	-27.4	-3.4	-	-	-	-
371	327	O7204a		-	-	298	1.0	851.6	855.4	3.8	0.4	-	-	-	-
372	328	O7205a		420.1	659.3	298	1.0	-	-	-	-	47.8	48.6	0.8	1.7
373	329	O7206a		-	-	298	1.0	840.3	832.0	-8.3	-1.0	-	-	-	-
374	330	O7207a		-	-	298	1.0	831.2	815.3	-15.9	-1.9	45.9	45.9	-0.0	-0.1
375	331	O7208a		-	-	298	1.0	832.7	818.0	-14.7	-1.8	46.8	47.2	0.5	1.0
376	332	O7301a		416.1	-	298	1.0	893.8	825.7	-68.1	-7.6	47.8	42.9	-4.9	-10.3
377	333	O7302a		416.9	-	293	1.0	922.9	908.5	-14.4	-1.6	-	-	-	-
378	334	O8101a		380.4	550.0	298	1.0	757.8	743.3	-14.5	-1.9	37.6	36.8	-0.8	-2.1
379	335	O8102a		439.9	571.4	298	1.0	748.0	745.2	-2.8	-0.4	40.1	40.5	0.4	0.9
380	336	O8103a		-	-	298	1.0	757.1	752.0	-5.1	-0.7	-	-	-	-
381	337	O8104a		-	571.4	298	1.0	758.1	753.3	-4.8	-0.6	42.3	42.7	0.4	0.9
382	338	O8105a		395.9	571.4	298	1.0	745.2	742.9	-2.3	-0.3	41.2	41.3	0.1	0.3

Table S.12 – Comparison of experimental and simulated properties using GM combination rules (continued).

n_{sim}	n_{iso}	Code	Outlier	T_m	T_b	T	P	$\rho_{\mathrm{liq}}[\mathrm{kg{\cdot}m^{-3}}]$				$\Delta H_{\mathrm{vap}}[\mathrm{kJ{\cdot}mol^{-1}}]$			
				[K]	[K]	[K]	[bar]	exp	sim	dev	err [%]	exp	sim	dev	err [%]
383	339	O8106a		-	-	298	1.0	750.8	749.5	-1.3	-0.2	-	-	-	-
384	340	O8107a		-	-	298	1.0	754.5	753.9	-0.6	-0.1	-	-	-	-
385	341	O8108a		394.2	571.4	298	1.0	758.8	755.9	-2.9	-0.4	-	-	-	-
386	342	O8109a		-	-	298	1.0	760.1	756.8	-3.3	-0.4	-	-	-	-
387	343	O8110a		424.1	571.4	298	1.0	755.2	752.0	-3.2	-0.4	-	-	-	-
388	344	O8111a		-	-	298	1.0	761.1	758.5	-2.6	-0.3	-	-	-	-
389	345	O8112a		-	571.4	298	1.0	759.8	756.9	-2.9	-0.4	-	-	-	-
390	346	O8113a		424.1	-	298	1.0	775.6	777.5	1.9	0.2	-	-	-	-
391	347	O8114a		415.1	-	298	1.0	768.2	762.0	-6.2	-0.8	-	-	-	-
392	348	O8115a		-	571.4	298	1.0	765.3	759.8	-5.5	-0.7	-	-	-	-
393	349	O8116a		413.4	584.1	298	1.0	764.1	759.4	-4.6	-0.6	44.7	45.4	0.7	1.5
394	350	O8201a		478.1	-	298	1.0	825.6	796.4	-29.2	-3.5	-	-	-	-
395	351	O8202a		-	-	298	1.0	851.8	855.3	3.5	0.4	-	-	-	-
396	352	O8203a		-	-	298	1.0	843.3	838.5	-4.8	-0.6	-	-	-	-
397	353	O8204a		-	-	298	1.0	840.7	836.1	-4.6	-0.6	-	-	-	-
398	354	O8205a		-	-	298	1.0	833.5	821.8	-11.7	-1.4	-	-	-	-
399	355	O8206a		434.0	-	298	1.0	833.1	820.9	-12.2	-1.5	50.9	51.5	0.6	1.2
400	356	O8207a		478.1	-	298	1.0	831.2	818.5	-12.7	-1.5	50.6	50.8	0.2	0.4
401	357	O8301a		462.1	624.0	298	1.0	903.3	883.6	-19.7	-2.2	56.4	59.7	3.3	5.8
402	358	O9101a		-	-	298	1.01	792.4	784.3	-8.1	-1.0	-	-	-	-
403	359	O9102a		-	-	298	1.0	761.6	761.1	-0.5	-0.1	-	-	-	-
404	360	O9103a		-	-	298	1.0	766.4	765.1	-1.3	-0.2	-	-	-	-
405	361	O9104a		-	-	298	1.0	759.4	760.4	1.0	0.1	-	-	-	-
406	362	O9105a		-	-	298	1.0	764.3	766.1	1.8	0.2	-	-	-	-
407	363	O9106a		-	-	298	1.0	767.6	767.2	-0.4	-0.1	-	-	-	-
408	364	O9107a		-	-	298	1.0	765.6	762.6	-3.0	-0.4	-	-	-	-
409	365	O9108a		-	-	298	1.0	802.1	778.3	-23.8	-3.0	-	-	-	-
410	366	O9109a		-	-	298	1.0	768.7	768.5	-0.2	-0.0	-	-	-	-
411	367	O9110a		-	-	298	1.0	767.8	767.1	-0.7	-0.1	-	-	-	-
412	368	O9111a		-	591.5	298	1.0	783.0	785.7	2.7	0.3	-	-	-	-
413	369	O9112a		-	-	298	1.0	775.3	771.7	-3.6	-0.5	-	-	-	-
414	370	O9113a		-	-	298	1.0	773.0	769.9	-3.1	-0.4	-	-	-	-
415	371	O9114a		-	-	298	1.0	772.7	769.3	-3.4	-0.4	-	-	-	-
416	372	O9201a		438.6	-	298	1.0	820.3	800.1	-20.1	-2.5	-	-	-	-
417	373	O9202a		-	-	298	1.0	844.0	840.6	-3.4	-0.4	-	-	-	-
418	374	O9203a		-	-	298	1.0	838.6	825.9	-12.7	-1.5	-	-	-	-
419	375	O9204a		-	-	298	1.0	840.6	837.2	-3.4	-0.4	-	-	-	-
420	376	O9205a		452.4	-	298	1.0	831.5	812.1	-19.4	-2.3	-	-	-	-
421	377	O9206a		-	-	298	1.0	-	-	-	-	54.7	55.0	0.3	0.6
422	378	O0101a		-	-	298	1.0	-	-	-	-	45.3	46.3	1.0	2.2
423	379	O0102a		-	-	298	1.0	-	-	-	-	53.2	52.0	-1.2	-2.2
424	380	O0103a		-	-	293	1.0	784.8	780.2	-4.6	-0.6	-	-	-	-
425	381	O0104a		445.6	610.4	298	1.0	771.5	773.6	2.1	0.3	51.4	52.3	0.9	1.7
426	382	O0105a		-	-	293	1.0	777.8	778.2	0.4	0.1	-	-	-	-
427	383	O0106a		-	-	298	1.0	775.1	775.2	0.1	0.0	-	-	-	-
428	384	O0107a		-	-	298	1.0	771.2	771.7	0.5	0.1	-	-	-	-
429	385	O0108a		-	-	298	1.0	782.1	778.4	-3.7	-0.5	-	-	-	-
430	386	O0109a		-	-	293	1.0	787.4	780.0	-7.4	-0.9	-	-	-	-

Table S.12 – Comparison of experimental and simulated properties using GM combination rules (continued).

n_{sim}	n_{iso}	Code	Outlier	T_m [K]	T_b [K]	T [K]	P [bar]	ρ_{liq}[kg·m^{-3}] exp	sim	dev	err [%]	ΔH_{vap}[kJ·mol^{-1}] exp	sim	dev	err [%]
431	387	O0110a		-	-	298	1.0	775.8	776.8	1.0	0.1	-	-	-	-
432	388	O0111a		-	-	298	1.0	773.6	775.0	1.4	0.2	-	-	-	-
433	389	O0112a		-	-	298	1.0	788.6	792.1	3.5	0.4	-	-	-	-
434	390	O0113a		-	-	298	1.0	782.3	779.9	-2.4	-0.3	-	-	-	-
435	391	O0114a		-	-	298	1.0	779.8	777.8	-2.0	-0.3	-	-	-	-
436	392	O0115a		-	-	298	1.0	778.8	778.0	-0.8	-0.1	53.2	54.9	1.7	3.1
437	393	O0116a		460.1	-	298	1.0	779.2	778.0	-1.2	-0.2	-	-	-	-
438	394	O0201a		444.4	-	298	1.0	816.8	794.0	-22.8	-2.8	-	-	-	-
439	395	O0202a		415.4	-	298	1.0	829.0	807.3	-21.7	-2.6	57.8	55.7	-2.1	-3.7
440	396	O0203a		-	-	298	1.0	840.9	839.4	-1.5	-0.2	-	-	-	-
441	397	O0204a		-	-	293	1.0	840.9	827.9	-13.0	-1.5	-	-	-	-
442	398	O0205a		476.4	715.5	298	1.0	833.7	824.2	-9.5	-1.1	58.8	59.4	0.6	1.0
443	399	O0301a		-	-	288	1.0	886.9	881.4	-5.5	-0.6	-	-	-	-
444	400	A1101a		254.1	415.2	251	1.36	805.2	816.2	11.0	1.4	-	-	-	-
445	400	A1101b	×	254.1	415.2	293	1.0	814.0	732.8	-81.2	-10.0	-	-	-	-
446	401	A2101a		293.6	466.0	298	1.0	772.0	777.7	5.7	0.7	26.9	24.5	-2.4	-9.0
447	402	A3101a		321.1	504.4	298	1.0	791.2	782.5	-8.7	-1.1	29.6	28.5	-1.1	-3.6
448	403	A4101a		337.2	540.4	298	1.0	796.6	781.4	-15.2	-1.9	32.3	31.4	-0.9	-2.7
449	404	A4102a		347.9	537.2	298	1.0	796.6	799.6	3.0	0.4	33.7	33.2	-0.5	-1.6
450	405	A5101a		347.1	570.0	298	0.14	783.1	787.6	4.5	0.6	-	-	-	-
451	406	A5102a		365.1	570.0	298	0.06	804.1	800.5	-3.6	-0.5	-	-	-	-
452	407	A5103a		365.8	570.0	298	1.0	794.2	803.7	9.5	1.2	-	-	-	-
453	408	A5104a		376.1	566.1	298	1.0	806.2	810.2	4.0	0.5	38.1	37.8	-0.3	-0.8
454	409	A6101a		377.1	596.5	298	0.03	801.0	813.1	12.1	1.5	-	-	-	-
455	410	A6102a		386.1	596.5	298	1.0	809.7	813.9	4.2	0.5	-	-	-	-
456	411	A6103a		389.9	596.5	298	0.02	814.1	814.1	-0.0	-0.0	-	-	-	-
457	412	A6104a		390.1	596.5	298	0.02	808.0	809.4	1.3	0.2	-	-	-	-
458	413	A6105a		395.1	596.5	299	0.01	806.6	817.3	10.6	1.3	-	-	-	-
459	414	A6106a		401.4	591.0	298	1.01	833.1	816.6	-16.5	-2.0	42.3	42.4	0.1	0.3
460	415	A6201a		435.2	-	292	1.0	1003.0	991.9	-11.1	-1.1	-	-	-	-
461	416	A7101a		-	-	298	1.0	829.5	823.7	-5.8	-0.7	-	-	-	-
462	417	A7102a		-	-	298	1.0	823.1	830.7	7.6	0.9	-	-	-	-
463	417	A7102b		-	-	334	1.0	-	-	-	-	42.4	42.4	0.0	0.0
464	418	A7103a		416.1	620.4	298	1.0	814.3	823.9	9.6	1.2	-	-	-	-
465	418	A7103b		416.1	620.4	329	1.0	-	-	-	-	42.8	43.5	0.7	1.6
466	419	A7104a		-	620.4	298	1.0	824.0	826.4	2.4	0.3	-	-	-	-
467	420	A7105a		-	620.4	293	1.0	820.6	823.5	2.9	0.4	-	-	-	-
468	421	A7106a		425.9	616.8	298	1.0	813.3	822.1	8.8	1.1	48.0	47.1	-0.9	-2.0
469	422	A8101a		-	-	293	1.0	847.6	828.3	-19.3	-2.3	-	-	-	-
470	423	A8102a		-	-	293	1.0	847.6	829.4	-18.2	-2.1	-	-	-	-
471	424	A8103a		433.8	642.4	298	1.0	815.2	824.9	9.7	1.2	-	-	-	-
472	425	A8104a		447.1	638.9	298	1.01	821.1	827.0	5.9	0.7	51.0	51.8	0.8	1.5
473	426	A9101a		-	-	298	1.0	827.0	840.4	13.4	1.6	-	-	-	-
474	427	A9102a		-	-	291	1.0	884.3	841.2	-43.1	-4.9	-	-	-	-
475	428	A9103a		-	-	293	1.0	848.3	835.6	-12.7	-1.5	-	-	-	-
476	429	A9104a		-	-	293	1.0	842.3	832.0	-10.3	-1.2	-	-	-	-
477	430	A9105a		468.1	662.7	298	1.01	831.0	830.4	-0.7	-0.1	55.3	56.5	1.2	2.2
478	431	A0101a		-	-	293	1.0	843.0	838.8	-4.2	-0.5	-	-	-	-

Table S.12 – Comparison of experimental and simulated properties using GM combination rules (continued).

n_{sim}	n_{iso}	Code	Outlier	T_m	T_b	T	P	ρ_{liq}[kg·m^{-3}]				ΔH_{vap}[kJ·mol^{-1}]			
				[K]	[K]	[K]	[bar]	exp	sim	dev	err [%]	exp	sim	dev	err [%]
479	432	A0102a		403.1	656.6	298	1.0	813.4	831.9	18.5	2.3	-	-	-	-
480	433	A0103a		488.1	674.2	298	1.0	824.9	833.5	8.6	1.0	59.5	61.2	1.7	2.9
481	434	K3101a		329.4	508.2	298	1.0	784.4	798.0	13.7	1.7	31.3	29.4	-1.9	-6.0
482	435	K4101a		352.8	535.5	298	1.0	799.9	802.1	2.2	0.3	34.5	32.9	-1.6	-4.6
483	436	K5101a		367.6	553.4	298	1.0	809.4	799.5	-9.8	-1.2	36.8	35.5	-1.3	-3.6
484	437	K5102a		375.1	560.9	298	1.0	809.6	804.6	-5.0	-0.6	38.5	36.5	-2.0	-5.2
485	438	K5103a		375.5	561.1	298	1.0	801.8	809.7	7.9	1.0	38.3	37.5	-0.8	-2.1
486	439	K6101a		379.2	-	298	1.0	804.3	817.2	12.9	1.6	38.3	37.7	-0.6	-1.6
487	440	K6102a		386.6	587.5	298	1.0	806.6	802.0	-4.6	-0.6	39.8	39.0	-0.8	-2.1
488	441	K6103a		390.6	587.5	298	1.0	808.3	813.1	4.8	0.6	39.8	39.8	0.0	0.1
489	442	K6104a		389.6	574.6	298	1.0	796.3	810.5	14.2	1.8	41.0	40.2	-0.8	-1.9
490	443	K6105a		396.6	582.8	298	1.0	811.1	810.3	-0.8	-0.1	40.6	41.0	0.4	1.0
491	444	K6106a		400.9	587.6	298	1.0	807.1	816.4	9.3	1.1	42.2	42.1	-0.1	-0.2
492	445	K6201a		433.1	629.0	293	1.0	959.0	980.4	21.4	2.2	-	-	-	-
493	446	K7101a		403.8	611.4	294	1.0	823.0	839.1	16.1	2.0	-	-	-	-
494	447	K7102a		398.1	611.4	298	1.0	808.4	817.5	9.1	1.1	42.3	41.3	-1.0	-2.5
495	448	K7103a		398.1	611.4	298	1.0	801.2	820.0	18.8	2.3	-	-	-	-
496	449	K7104a		397.6	611.4	298	1.0	799.7	801.5	1.8	0.2	41.5	41.4	-0.1	-0.1
497	450	K7105a		405.1	611.4	293	1.0	827.3	827.5	0.2	0.0	-	-	-	-
498	451	K7106a		409.1	611.4	298	1.0	824.0	813.6	-10.4	-1.3	-	-	-	-
499	452	K7107a		406.1	611.4	302	0.01	809.5	804.5	-4.9	-0.6	-	-	-	-
500	453	K7108a		411.1	611.4	295	1.0	815.3	824.7	9.4	1.2	-	-	-	-
501	454	K7109a		413.1	611.4	298	1.0	828.0	819.1	-8.9	-1.1	-	-	-	-
502	455	K7110a		409.1	611.4	293	1.0	812.0	815.3	3.3	0.4	-	-	-	-
503	456	K7111a		412.1	611.4	298	1.0	808.5	822.0	13.5	1.7	-	-	-	-
504	457	K7112a		417.9	611.4	293	1.0	811.6	823.0	11.4	1.4	-	-	-	-
505	458	K7113a		417.1	602.0	298	1.0	811.6	815.0	3.4	0.4	-	-	-	-
506	459	K7114a		420.6	606.6	298	1.0	814.6	816.5	1.8	0.2	-	-	-	-
507	460	K7115a		424.1	611.4	298	1.0	811.6	821.6	9.9	1.2	46.1	46.8	0.7	1.4
508	461	K7201a		451.6	645.0	292	1.0	953.1	971.1	18.0	1.9	-	-	-	-
509	462	K7202a		449.1	645.0	293	1.0	945.0	959.3	14.3	1.5	-	-	-	-
510	463	K8101a		-	-	293	1.0	839.5	858.9	19.4	2.3	-	-	-	-
511	464	K8102a		408.2	633.4	298	1.0	802.3	820.0	17.7	2.2	43.3	43.9	0.6	1.5
512	465	K8103a		-	-	293	1.0	826.0	845.8	19.8	2.4	-	-	-	-
513	466	K8104a		426.6	633.4	293	1.0	838.9	854.5	15.6	1.9	-	-	-	-
514	467	K8105a		421.1	633.4	293	1.0	829.8	838.7	8.9	1.1	-	-	-	-
515	468	K8106a		420.1	633.4	293	1.0	825.7	843.5	17.8	2.2	-	-	-	-
516	469	K8107a		419.1	633.4	298	1.0	810.5	822.2	11.7	1.4	-	-	-	-
517	470	K8108a		-	-	293	1.0	829.0	842.2	13.2	1.6	-	-	-	-
518	471	K8109a		-	-	293	1.0	812.0	824.5	12.5	1.5	-	-	-	-
519	472	K8110a		420.6	633.4	293	1.0	812.1	813.1	1.0	0.1	-	-	-	-
520	473	K8111a		431.1	633.4	295	1.0	829.5	835.1	5.6	0.7	-	-	-	-
521	474	K8112a		426.1	633.4	298	1.0	817.0	817.4	0.4	0.1	-	-	-	-
522	475	K8113a		-	633.4	298	1.0	820.0	818.9	-1.1	-0.1	-	-	-	-
523	476	K8114a		431.1	633.4	293	1.0	817.5	818.0	0.5	0.1	-	-	-	-
524	477	K8115a		435.1	633.4	297	1.0	829.0	822.4	-6.6	-0.8	-	-	-	-
525	478	K8116a		427.1	633.4	295	1.0	813.0	817.9	4.9	0.6	-	-	-	-
526	479	K8117a		437.1	633.4	293	1.0	830.4	822.6	-7.8	-0.9	-	-	-	-

Table S.12 – Comparison of experimental and simulated properties using GM combination rules (continued).

n_{sim}	n_{iso}	Code	Outlier	T_m [K]	T_b [K]	T [K]	P [bar]	ρ_{liq}[kg·m^{-3}] exp	sim	dev	err [%]	ΔH_{vap}[kJ·mol^{-1}] exp	sim	dev	err [%]
527	480	K8118a		437.0	633.4	298	1.0	811.0	828.8	17.8	2.2	-	-	-	-
528	481	K8119a		440.1	633.4	298	1.0	810.0	823.6	13.6	1.7	-	-	-	-
529	482	K8120a		436.1	633.4	298	1.0	814.7	819.8	5.1	0.6	-	-	-	-
530	483	K8121a		440.6	633.4	298	1.0	822.0	821.1	-0.9	-0.1	-	-	-	-
531	484	K8122a		445.8	632.7	298	1.0	815.2	825.9	10.6	1.3	51.8	51.5	-0.3	-0.7
532	485	K9101a		425.1	653.7	298	1.0	820.2	842.5	22.3	2.7	45.4	46.7	1.3	2.9
533	486	K9102a		-	-	296	1.0	812.0	823.8	11.8	1.5	-	-	-	-
534	487	K9103a		-	-	293	1.0	816.8	829.3	12.5	1.5	-	-	-	-
535	488	K9104a		-	-	298	1.0	809.0	823.9	14.9	1.8	-	-	-	-
536	489	K9105a		435.1	653.7	287	1.0	826.0	828.9	2.9	0.3	-	-	-	-
537	490	K9106a		445.0	653.7	298	1.0	813.5	816.3	2.8	0.3	-	-	-	-
538	491	K9107a		441.4	653.7	298	1.0	802.4	816.0	13.5	1.7	50.9	51.0	0.1	0.1
539	492	K9108a		-	-	293	1.0	833.5	841.2	7.7	0.9	-	-	-	-
540	493	K9109a		-	-	298	1.0	817.0	828.8	11.8	1.5	-	-	-	-
541	494	K9110a		447.1	653.7	287	1.0	829.0	830.2	1.2	0.1	-	-	-	-
542	495	K9111a		456.6	653.7	298	1.0	820.0	823.0	3.0	0.4	-	-	-	-
543	496	K9112a		456.0	653.7	293	1.0	821.2	822.3	1.1	0.1	-	-	-	-
544	497	K9113a		-	-	293	1.0	824.6	833.5	8.9	1.1	-	-	-	-
545	498	K9114a		456.6	653.7	300	1.0	832.0	825.7	-6.3	-0.8	-	-	-	-
546	499	K9115a		-	653.7	298	1.0	815.0	820.2	5.2	0.6	-	-	-	-
547	500	K9116a		451.1	653.7	293	1.0	823.9	825.8	1.9	0.2	-	-	-	-
548	501	K9117a		-	653.7	298	1.0	822.0	832.3	10.3	1.3	-	-	-	-
549	502	K9118a		461.6	640.0	298	1.0	817.8	824.1	6.3	0.8	53.3	54.9	1.6	3.0
550	503	K9119a		460.6	653.7	298	1.0	819.7	824.0	4.3	0.5	-	-	-	-
551	504	K9120a		463.1	653.7	298	1.0	820.4	824.6	4.2	0.5	55.6	55.0	-0.6	-1.1
552	505	K9121a		467.1	652.5	298	1.0	817.8	829.6	11.8	1.4	-	-	-	-
553	506	K0101a		-	-	298	1.0	816.9	829.5	12.6	1.5	48.8	50.1	1.3	2.7
554	507	K0102a		-	-	298	1.0	825.2	834.0	8.8	1.1	-	-	-	-
555	508	K0103a		-	-	289	1.0	825.2	844.2	19.0	2.3	-	-	-	-
556	509	K0104a		-	-	293	1.0	832.0	833.0	1.0	0.1	-	-	-	-
557	510	K0105a		-	-	293	1.0	814.3	831.3	17.0	2.1	-	-	-	-
558	511	K0106a		473.0	672.7	293	1.0	822.6	826.6	4.0	0.5	-	-	-	-
559	512	K0107a		-	-	298	1.0	818.0	823.4	5.4	0.7	-	-	-	-
560	513	K0108a		476.6	672.7	293	1.0	821.3	829.1	7.8	0.9	-	-	-	-
561	514	K0109a		-	-	293	1.0	838.4	838.6	0.2	0.0	-	-	-	-
562	515	K0110a		477.1	672.7	298	1.0	820.5	827.1	6.6	0.8	-	-	-	-
563	516	K0111a		479.6	672.7	294	1.0	822.0	830.7	8.7	1.1	-	-	-	-
564	517	K0112a		476.1	672.7	298	1.0	821.9	828.5	6.5	0.8	-	-	-	-
565	518	K0113a		483.4	672.7	298	1.0	820.1	832.5	12.4	1.5	60.9	60.9	-0.0	-0.0
566	519	E2201a	×	304.9	487.2	298	1.0	966.8	1159.9	193.1	20.0	28.4	28.3	-0.1	-0.2
567	520	E3201a	×	327.5	508.4	298	1.0	915.9	1091.4	175.5	19.2	31.5	33.6	2.1	6.7
568	521	E3202a		330.1	506.6	298	1.01	927.5	897.1	-30.4	-3.3	32.3	31.0	-1.3	-3.9
569	522	E4201a		341.2	514.9	298	0.17	870.2	865.3	-5.0	-0.6	-	-	-	-
570	523	E4202a	×	354.0	538.0	298	1.0	899.9	1051.5	151.6	16.9	36.6	39.1	2.5	6.8
571	524	E4203a		352.6	530.6	298	1.0	909.1	882.7	-26.4	-2.9	35.7	34.8	-0.9	-2.4
572	525	E4204a		350.2	523.3	298	1.0	894.3	874.4	-19.9	-2.2	35.1	34.3	-0.8	-2.4
573	526	E4401a	×	447.1	-	273	1.0	1193.0	1297.7	104.7	8.8	-	-	-	-
574	527	E5201a		355.9	541.0	298	0.09	871.9	864.2	-7.7	-0.9	-	-	-	-

Table S.12 – Comparison of experimental and simulated properties using GM combination rules (continued).

n_{sim}	n_{iso}	Code	Outlier	T_m [K]	T_b [K]	T [K]	P [bar]	ρ_{liq}[kg·m^{-3}] exp	sim	dev	err [%]	ΔH_{vap}[kJ·mol^{-1}] exp	sim	dev	err [%]
575	528	E5202a	×	371.2	551.4	298	1.0	875.7	1022.7	147.0	16.8	-	-	-	-
576	529	E5203a		363.6	541.0	298	0.06	878.6	873.7	-4.9	-0.6	-	-	-	-
577	530	E5204a	×	379.2	541.0	298	1.0	887.6	1023.8	136.2	15.3	40.5	44.6	4.1	10.0
578	531	E5205a		365.6	540.7	298	1.0	883.3	866.0	-17.3	-2.0	37.3	37.4	0.1	0.3
579	532	E5206a		361.6	532.0	298	1.0	869.0	855.8	-13.2	-1.5	37.0	37.1	0.1	0.3
580	533	E5207a		375.9	554.5	298	1.01	892.5	878.2	-14.3	-1.6	39.8	39.3	-0.5	-1.3
581	534	E5208a		372.2	546.0	298	1.0	884.0	865.0	-19.0	-2.1	39.3	38.1	-1.2	-3.2
582	535	E5209a		374.6	549.7	298	1.01	882.8	870.3	-12.5	-1.4	39.1	38.6	-0.5	-1.3
583	536	E5401a		454.6	-	298	1.0	1146.7	1109.3	-37.4	-3.3	57.5	55.6	-1.9	-3.3
584	537	E5402a		437.6	-	298	1.0	1135.5	1108.6	-26.9	-2.4	56.4	56.4	0.0	0.1
585	538	E6201a		385.6	564.6	298	0.02	883.7	882.9	-0.8	-0.1	-	-	-	-
586	539	E6202a	×	397.1	564.6	298	1.0	877.0	1009.1	132.1	15.1	-	-	-	-
587	540	E6203a	×	406.6	576.0	298	1.0	880.4	1004.0	123.7	14.0	45.2	50.0	4.8	10.5
588	541	E6204a		374.2	-	293	1.0	850.0	870.4	20.4	2.4	38.8	39.2	0.4	1.1
589	542	E6205a		369.1	564.6	298	1.0	861.6	847.4	-14.2	-1.6	38.0	37.2	-0.8	-2.1
590	543	E6206a		-	-	293	1.0	884.7	874.5	-10.2	-1.2	-	-	-	-
591	544	E6207a		383.0	553.1	298	1.0	864.0	852.3	-11.8	-1.4	39.8	40.7	0.9	2.3
592	545	E6208a		389.6	564.6	298	1.0	875.9	870.5	-5.4	-0.6	-	-	-	-
593	546	E6209a		383.1	553.0	298	1.0	860.1	849.0	-11.1	-1.3	-	-	-	-
594	547	E6210a		389.8	560.8	298	1.0	866.3	860.9	-5.4	-0.6	39.5	41.7	2.2	5.6
595	548	E6211a		385.1	564.6	298	1.0	866.0	859.8	-6.3	-0.7	-	-	-	-
596	549	E6212a		400.6	564.6	298	1.0	885.2	876.7	-8.5	-1.0	43.7	44.2	0.5	1.1
597	550	E6213a		394.6	571.0	298	1.0	873.8	863.8	-10.0	-1.1	42.0	42.7	0.7	1.6
598	551	E6214a		395.6	568.6	298	1.0	876.4	862.6	-13.7	-1.6	43.2	42.5	-0.7	-1.6
599	552	E6215a		399.1	575.4	298	1.0	876.4	870.3	-6.0	-0.7	42.7	43.6	0.9	2.2
600	553	E6401a		447.1	-	298	1.0	1093.6	1067.5	-26.1	-2.4	-	-	-	-
601	553	E6401b		447.1	-	293	1.0	-	-	-	-	57.8	58.2	0.4	0.7
602	554	E6402a		442.1	635.0	298	1.0	1070.0	1063.8	-6.2	-0.6	59.0	59.4	0.4	0.6
603	555	E6403a		469.6	657.0	298	1.0	1114.0	1077.9	-36.1	-3.2	60.9	59.6	-1.3	-2.2
604	556	E6404a		463.6	653.0	298	1.0	1098.7	1065.0	-33.7	-3.1	61.0	58.2	-2.8	-4.6
605	557	E7201a	×	428.6	586.3	298	1.0	874.6	990.0	115.3	13.2	50.0	55.7	5.7	11.4
606	558	E7202a		391.6	-	298	1.0	849.5	851.4	1.9	0.2	41.3	41.8	0.5	1.2
607	559	E7203a		-	-	293	1.0	870.0	878.2	8.2	0.9	43.9	44.3	0.4	0.9
608	560	E7204a		-	586.3	298	1.0	853.9	861.0	7.1	0.8	-	-	-	-
609	561	E7205a		-	-	298	1.0	872.5	861.3	-11.2	-1.3	40.3	41.6	1.3	3.1
610	562	E7206a		414.6	-	293	1.0	864.7	847.8	-16.9	-2.0	-	-	-	-
611	563	E7207a		396.1	-	294	1.0	846.7	842.1	-4.6	-0.5	-	-	-	-
612	564	E7208a		401.6	586.3	298	1.0	866.0	861.4	-4.6	-0.5	-	-	-	-
613	565	E7209a		409.1	586.3	293	1.0	879.7	876.8	-2.9	-0.3	-	-	-	-
614	566	E7210a		-	-	293	1.0	876.5	873.9	-2.6	-0.3	-	-	-	-
615	567	E7211a		-	-	293	1.0	867.8	861.7	-6.1	-0.7	44.7	45.2	0.5	1.2
616	568	E7212a		407.1	-	298	1.0	859.5	851.2	-8.3	-1.0	-	-	-	-
617	569	E7213a		408.1	-	298	1.0	861.2	858.2	-3.1	-0.4	-	-	-	-
618	570	E7214a		409.1	592.0	298	1.0	867.5	854.0	-13.5	-1.6	-	-	-	-
619	571	E7215a		406.1	-	298	1.0	861.2	853.3	-7.9	-0.9	-	-	-	-
620	572	E7216a		402.1	-	298	1.0	853.9	849.5	-4.5	-0.5	-	-	-	-
621	573	E7217a		413.1	586.3	293	1.0	867.8	871.7	3.9	0.4	-	-	-	-
622	574	E7218a		405.1	586.3	303	0.01	861.5	857.1	-4.4	-0.5	-	-	-	-

Table S.12 – Comparison of experimental and simulated properties using GM combination rules (continued).

n_{sim}	n_{iso}	Code	Outlier	T_m [K]	T_b [K]	T [K]	P [bar]	ρ_{liq}[kg·m^{-3}] exp	sim	dev	err [%]	ΔH_{vap}[kJ·mol^{-1}] exp	sim	dev	err [%]
623	575	E7219a		414.8	586.1	299	1.0	864.8	866.9	2.1	0.2	46.4	47.0	0.6	1.4
624	576	E7220a		406.1	586.3	298	1.0	863.1	861.3	-1.8	-0.2	-	-	-	-
625	577	E7221a		422.6	586.3	298	1.0	880.4	875.4	-5.0	-0.6	47.7	48.9	1.2	2.5
626	578	E7222a		419.2	586.3	298	1.01	869.4	864.1	-5.3	-0.6	47.0	47.4	0.4	0.8
627	579	E7223a		416.4	593.7	298	1.0	868.2	861.2	-7.0	-0.8	-	-	-	-
628	580	E7224a		419.8	594.6	298	1.0	871.5	863.2	-8.3	-1.0	48.5	47.2	-1.3	-2.6
629	581	E7225a		422.1	599.9	298	1.0	872.2	870.2	-2.0	-0.2	48.6	48.4	-0.2	-0.4
630	582	E7401a		-	-	293	1.0	-	-	-	-	55.6	56.6	1.0	1.9
631	583	E7402a		-	-	298	1.0	1061.4	1046.3	-15.1	-1.4	-	-	-	-
632	584	E7403a		469.1	-	298	1.0	1076.0	1044.5	-31.5	-2.9	-	-	-	-
633	585	E7404a		463.6	-	293	1.0	1059.0	1032.5	-26.5	-2.5	-	-	-	-
634	586	E7405a		472.1	653.0	298	1.0	1049.8	1029.5	-20.3	-1.9	58.7	60.7	2.0	3.5
635	587	E7406a		481.4	-	293	1.0	1076.0	1045.6	-30.4	-2.8	-	-	-	-
636	588	E7407a		487.1	-	293	1.01	1087.7	1058.0	-29.6	-2.7	65.7	65.0	-0.7	-1.1
637	589	E7408a		482.6	-	293	1.0	1054.7	1044.8	-9.9	-0.9	-	-	-	-
638	590	E8201a	×	451.2	606.5	302	1.0	869.0	949.8	80.8	9.3	53.8	58.6	4.8	8.8
639	591	E8202a		-	-	293	1.0	830.0	843.4	13.4	1.6	-	-	-	-
640	592	E8203a		-	-	298	1.0	874.9	886.8	11.9	1.4	-	-	-	-
641	593	E8204a		-	-	277	1.0	883.0	881.8	-1.2	-0.1	-	-	-	-
642	594	E8205a		-	-	293	1.0	860.4	865.4	5.0	0.6	-	-	-	-
643	595	E8206a		-	-	293	1.0	867.9	878.7	10.8	1.2	-	-	-	-
644	596	E8207a		437.4	606.5	308	0.01	874.7	845.5	-29.2	-3.3	-	-	-	-
645	597	E8208a		426.1	606.5	320	0.01	862.6	842.8	-19.8	-2.3	-	-	-	-
646	598	E8209a		-	-	298	1.0	866.0	861.5	-4.5	-0.5	-	-	-	-
647	599	E8210a		421.1	-	293	1.0	847.0	848.4	1.4	0.2	48.5	48.0	-0.5	-1.0
648	600	E8211a		-	606.5	289	1.0	870.0	851.0	-19.0	-2.2	-	-	-	-
649	601	E8212a		420.1	-	298	1.0	846.1	845.3	-0.8	-0.1	-	-	-	-
650	602	E8213a		420.6	-	298	1.0	880.5	860.3	-20.2	-2.3	-	-	-	-
651	603	E8214a		-	-	293	1.0	875.0	875.8	0.8	0.1	-	-	-	-
652	604	E8215a		-	-	293	1.0	863.3	864.6	1.3	0.1	-	-	-	-
653	605	E8216a		428.3	-	293	1.0	876.5	863.0	-13.5	-1.5	48.4	49.8	1.4	2.9
654	605	E8216b		428.3	-	298	1.0	-	-	-	-	48.4	49.6	1.2	2.4
655	606	E8217a		-	-	293	1.0	869.7	859.7	-10.0	-1.2	-	-	-	-
656	607	E8218a		-	-	298	1.0	857.4	853.2	-4.2	-0.5	-	-	-	-
657	608	E8219a		-	-	293	1.0	878.0	868.1	-9.9	-1.1	-	-	-	-
658	609	E8220a		429.1	-	298	1.0	857.5	856.1	-1.4	-0.2	-	-	-	-
659	610	E8221a		436.1	606.5	293	1.0	870.5	866.6	-3.9	-0.4	-	-	-	-
660	611	E8222a		430.1	611.0	298	1.0	860.6	853.7	-6.9	-0.8	-	-	-	-
661	612	E8223a		446.1	606.5	298	1.0	865.0	861.4	-3.6	-0.4	-	-	-	-
662	613	E8224a		-	-	298	1.0	863.2	852.8	-10.5	-1.2	-	-	-	-
663	614	E8225a		440.2	606.5	298	1.0	861.3	854.5	-6.8	-0.8	-	-	-	-
664	615	E8226a		436.4	606.5	293	1.0	857.9	855.2	-2.7	-0.3	-	-	-	-
665	616	E8227a		435.6	606.5	293	1.0	879.0	876.3	-2.7	-0.3	-	-	-	-
666	617	E8228a		436.1	606.5	298	1.0	869.1	867.6	-1.5	-0.2	-	-	-	-
667	618	E8229a		-	606.5	298	1.0	859.9	862.1	2.2	0.3	-	-	-	-
668	619	E8230a		447.1	606.5	298	1.01	875.9	874.6	-1.3	-0.1	51.6	53.6	2.0	3.9
669	620	E8231a		440.1	606.5	298	1.0	866.7	864.3	-2.4	-0.3	50.6	52.2	1.6	3.2
670	621	E8232a		440.6	606.5	298	1.0	865.8	861.9	-3.9	-0.4	-	-	-	-

Table S.12 – Comparison of experimental and simulated properties using GM combination rules (continued).

n_{sim}	n_{iso}	Code	Outlier	T_m	T_b	T	P	ρ_{liq}[kg·m^{-3}]				ΔH_{vap}[kJ·mol^{-1}]			
				[K]	[K]	[K]	[bar]	exp	sim	dev	err [%]	exp	sim	dev	err [%]
671	622	E8233a		438.1	606.5	298	1.01	869.1	862.2	-6.9	-0.8	-	-	-	-
672	623	E8234a		441.9	606.5	298	1.0	868.1	863.1	-5.0	-0.6	52.2	52.0	-0.2	-0.4
673	624	E8235a		444.6	606.5	298	1.0	868.6	870.1	1.5	0.2	51.9	53.0	1.1	2.1
674	625	E8401a		-	-	298	1.0	1036.9	1027.4	-9.5	-0.9	-	-	-	-
675	626	E8402a		474.1	-	298	1.0	1017.4	1002.8	-14.6	-1.4	-	-	-	-
676	627	E8403a		489.6	663.0	298	1.0	1035.3	1011.9	-23.4	-2.3	64.5	64.4	-0.1	-0.1
677	628	E8404a		495.1	-	298	1.0	1057.6	1034.9	-22.7	-2.1	69.0	69.3	0.3	0.5
678	629	E8405a		484.1	-	293	1.0	1042.0	1014.9	-27.1	-2.6	67.6	64.2	-3.3	-5.0
679	630	E8406a		502.1	-	293	1.0	1046.0	1027.8	-18.2	-1.7	-	-	-	-
680	631	E9201a	×	471.9	625.3	298	1.0	871.0	952.7	81.7	9.4	58.2	64.8	6.6	11.3
681	632	E9202a		-	-	298	1.0	-	-	-	-	48.4	51.2	2.8	5.8
682	633	E9203a		-	-	298	1.0	-	-	-	-	47.8	47.5	-0.3	-0.7
683	634	E9204a		-	-	298	1.0	-	-	-	-	50.4	50.8	0.4	0.7
684	635	E9205a		460.3	625.3	298	1.0	-	-	-	-	50.3	49.6	-0.7	-1.5
685	636	E9206a		-	-	301	1.0	855.0	856.1	1.1	0.1	-	-	-	-
686	637	E9207a		442.1	-	293	1.0	862.7	856.9	-5.8	-0.7	51.7	53.8	2.1	4.0
687	638	E9208a		-	-	298	1.0	869.0	869.1	0.1	0.0	-	-	-	-
688	639	E9209a		442.1	-	298	1.0	861.7	849.8	-11.9	-1.4	-	-	-	-
689	640	E9210a		-	-	293	1.0	848.2	853.0	4.8	0.6	-	-	-	-
690	641	E9211a		-	-	293	1.0	871.0	874.8	3.8	0.4	-	-	-	-
691	642	E9212a		-	-	293	1.0	857.2	864.8	7.6	0.9	-	-	-	-
692	643	E9213a		-	-	293	1.0	868.8	861.8	-7.0	-0.8	-	-	-	-
693	644	E9214a		-	-	293	1.0	866.9	860.9	-6.0	-0.7	-	-	-	-
694	645	E9215a		452.1	-	293	1.0	862.0	860.6	-1.4	-0.2	50.6	54.2	3.6	7.0
695	646	E9216a		-	625.3	293	1.0	872.1	858.4	-13.7	-1.6	-	-	-	-
696	647	E9217a		450.1	625.3	293	1.0	867.9	867.8	-0.1	-0.0	-	-	-	-
697	648	E9218a		456.9	-	298	1.0	856.7	857.5	0.8	0.1	-	-	-	-
698	649	E9219a		453.1	625.3	293	1.0	870.8	871.6	0.8	0.1	-	-	-	-
699	650	E9220a		452.1	625.3	298	1.0	860.3	860.7	0.4	0.0	-	-	-	-
700	651	E9221a		456.1	625.3	298	1.0	853.6	855.0	1.4	0.2	-	-	-	-
701	652	E9222a		455.1	625.3	298	1.0	864.7	854.0	-10.8	-1.2	-	-	-	-
702	653	E9223a		-	-	298	1.0	854.9	854.1	-0.7	-0.1	-	-	-	-
703	654	E9224a		-	-	298	1.0	852.5	852.6	0.1	0.0	-	-	-	-
704	655	E9225a		-	625.3	298	1.0	857.0	862.2	5.1	0.6	-	-	-	-
705	656	E9226a		466.1	625.3	298	1.0	873.1	873.8	0.7	0.1	56.4	58.5	2.1	3.7
706	657	E9227a		460.1	625.3	298	1.0	864.7	864.4	-0.3	-0.0	-	-	-	-
707	658	E9228a		460.1	625.3	298	1.0	863.0	862.2	-0.8	-0.1	-	-	-	-
708	659	E9229a		459.1	625.3	298	1.0	863.4	862.3	-1.0	-0.1	-	-	-	-
709	660	E9230a		458.1	625.3	298	1.0	861.9	862.5	0.6	0.1	53.6	56.5	2.9	5.5
710	661	E9231a		463.1	625.3	298	1.0	865.4	863.8	-1.7	-0.2	57.1	56.8	-0.3	-0.6
711	662	E9232a		465.6	625.3	298	1.0	866.4	869.4	2.9	0.3	56.9	57.9	1.0	1.7
712	663	E9401a		509.2	-	298	1.0	-	-	-	-	63.9	64.5	0.6	0.9
713	664	E9402a		481.1	-	298	1.0	1000.7	989.9	-10.8	-1.1	-	-	-	-
714	665	E9403a		502.1	-	298	1.0	-	-	-	-	66.2	68.7	2.5	3.8
715	666	E9404a		509.6	-	293	1.0	1022.0	1004.3	-17.7	-1.7	67.0	70.4	3.4	5.1
716	667	E9405a		509.2	-	293	1.0	1039.1	1023.2	-15.9	-1.5	73.5	73.9	0.4	0.5
717	668	E9406a		514.1	-	293	1.0	1029.6	1013.2	-16.4	-1.6	-	-	-	-
718	669	E0201a		485.7	643.0	293	1.0	867.0	893.2	26.2	3.0	-	-	-	-

Table S.12 – Comparison of experimental and simulated properties using GM combination rules (continued).

n_{sim}	n_{iso}	Code	Outlier	T_m [K]	T_b [K]	T [K]	P [bar]	ρ_{liq}[kg·m^{-3}] exp	sim	dev	err [%]	ΔH_{vap}[kJ·mol^{-1}] exp	sim	dev	err [%]
719	670	E0202a		-	-	293	1.0	843.1	845.8	2.7	0.3	48.9	50.5	1.6	3.3
720	671	E0203a		-	-	298	1.0	-	-	-	-	48.0	48.9	0.9	1.8
721	672	E0204a		461.1	-	273	1.0	872.9	869.9	-3.0	-0.3	-	-	-	-
722	673	E0205a		-	-	289	1.0	883.7	888.0	4.3	0.5	-	-	-	-
723	674	E0206a		-	-	293	1.0	856.8	864.1	7.3	0.9	-	-	-	-
724	675	E0207a		463.1	-	298	1.0	854.1	856.8	2.7	0.3	-	-	-	-
725	676	E0208a		460.1	-	293	1.0	847.4	863.7	16.3	1.9	-	-	-	-
726	677	E0209a		-	-	293	1.0	865.9	864.7	-1.2	-0.1	-	-	-	-
727	678	E0210a		-	-	298	1.0	864.4	869.9	5.5	0.6	-	-	-	-
728	679	E0211a		473.7	643.0	298	1.0	858.6	860.9	2.3	0.3	-	-	-	-
729	680	E0212a		-	-	277	1.0	875.9	885.1	9.2	1.1	-	-	-	-
730	681	E0213a		-	-	293	1.0	870.0	859.3	-10.7	-1.2	-	-	-	-
731	682	E0214a		-	643.0	293	1.0	872.6	868.0	-4.6	-0.5	-	-	-	-
732	683	E0215a		466.1	643.0	293	1.0	858.0	865.0	7.0	0.8	-	-	-	-
733	684	E0216a		-	-	298	1.0	857.5	855.0	-2.4	-0.3	-	-	-	-
734	685	E0217a		471.8	642.4	298	1.0	868.8	870.5	1.7	0.2	-	-	-	-
735	686	E0218a		-	643.0	298	1.0	858.1	862.5	4.4	0.5	-	-	-	-
736	687	E0219a		486.6	643.0	298	1.0	870.9	873.0	2.1	0.2	61.6	63.2	1.6	2.6
737	688	E0220a		481.6	643.0	298	1.0	862.9	864.7	1.8	0.2	59.5	61.8	2.3	3.9
738	689	E0221a		481.1	643.0	298	1.0	861.6	862.6	1.0	0.1	-	-	-	-
739	690	E0222a		481.1	643.0	298	1.0	862.3	862.5	0.2	0.0	-	-	-	-
740	691	E0223a		476.9	643.0	298	1.0	860.2	862.5	2.3	0.3	-	-	-	-
741	692	E0224a		479.1	643.0	298	1.01	851.0	862.3	11.3	1.3	-	-	-	-
742	693	E0225a		483.1	643.0	298	1.0	864.0	863.9	-0.1	-0.0	-	-	-	-
743	694	E0226a		484.4	643.0	298	1.0	864.3	869.3	5.0	0.6	-	-	-	-
744	695	E0401a		488.1	-	293	1.0	996.1	984.8	-11.3	-1.1	-	-	-	-
745	696	E0402a		-	-	298	1.0	980.3	967.7	-12.5	-1.3	70.8	68.5	-2.3	-3.3
746	697	E0403a		494.1	-	298	1.0	982.8	979.1	-3.6	-0.4	-	-	-	-
747	698	E0404a		524.0	-	298	1.0	997.4	983.8	-13.6	-1.4	-	-	-	-
748	699	E0405a		518.1	-	298	1.0	1003.7	988.6	-15.1	-1.5	73.0	74.5	1.5	2.0
749	700	E0406a		541.1	-	298	1.0	1019.2	1006.1	-13.1	-1.3	78.1	78.0	-0.1	-0.1
750	701	E0407a		513.1	-	298	1.0	995.3	982.9	-12.4	-1.2	73.2	71.9	-1.3	-1.8
751	702	L1101a		337.9	512.6	298	1.01	786.7	733.7	-53.1	-6.7	37.7	37.1	-0.6	-1.5
752	703	L2101a		351.4	513.9	298	1.01	786.6	754.6	-32.0	-4.1	42.3	42.2	-0.1	-0.3
753	704	L2201a		470.4	720.0	298	1.0	1109.9	1070.9	-39.0	-3.5	64.8	64.4	-0.4	-0.6
754	705	L3101a		355.4	508.3	298	1.0	781.2	755.7	-25.5	-3.3	44.4	44.8	0.4	1.0
755	706	L3102a		370.4	536.8	298	1.0	799.8	782.4	-17.4	-2.2	46.6	47.1	0.5	1.2
756	707	L3201a		460.8	700.2	298	1.0	1032.5	1001.5	-31.0	-3.0	62.2	65.2	3.0	4.9
757	708	L3202a	×	487.6	724.0	298	1.0	1050.3	1022.1	-28.2	-2.7	69.8	60.3	-9.5	-13.6
758	709	L3301a		563.1	850.0	298	1.0	1258.3	1210.8	-47.5	-3.8	-	-	-	-
759	709	L3301b		563.1	850.0	308	1.0	-	-	-	-	85.8	79.0	-6.8	-8.0
760	710	L4101a		355.6	506.2	299	1.0	779.5	762.2	-17.3	-2.2	46.2	42.3	-3.9	-8.4
761	711	L4102a		380.8	547.8	298	1.0	797.8	792.0	-5.8	-0.7	50.8	50.4	-0.4	-0.8
762	712	L4103a		372.7	536.0	298	1.01	803.0	782.9	-20.1	-2.5	48.5	49.4	0.9	1.8
763	713	L4104a		390.8	563.0	298	1.0	805.8	796.9	-8.9	-1.1	52.1	52.0	-0.1	-0.2
764	714	L4201a		451.1	728.8	298	1.0	989.6	957.2	-32.4	-3.3	-	-	-	-
765	715	L4202a		455.1	728.8	298	1.0	999.8	950.0	-49.8	-5.0	-	-	-	-
766	716	L4203a	×	487.1	728.8	293	1.0	1009.0	976.3	-32.7	-3.2	71.3	61.0	-10.3	-14.4

Table S.12 – Comparison of experimental and simulated properties using GM combination rules (continued).

n_{sim}	n_{iso}	Code	Outlier	T_m [K]	T_b [K]	T [K]	P [bar]	ρ_{liq}[kg·m^{-3}] exp	sim	dev	err [%]	ΔH_{vap}[kJ·mol^{-1}] exp	sim	dev	err [%]
767	717	L4204a		464.1	680.0	298	1.0	999.2	983.2	-16.1	-1.6	-	-	-	-
768	718	L4205a	×	480.1	676.0	298	1.0	1000.2	964.2	-36.0	-3.6	72.6	60.6	-12.0	-16.6
769	719	L4206a		501.1	728.8	298	1.0	1015.4	998.2	-17.2	-1.7	76.6	70.7	-5.9	-7.7
770	720	L4301a		-	697.0	298	1.0	1184.0	1153.0	-31.0	-2.6	-	-	-	-
771	721	L5101a		375.1	543.7	298	1.0	804.7	782.1	-22.6	-2.8	50.1	45.5	-4.6	-9.1
772	722	L5102a		384.6	556.1	298	1.0	815.0	800.9	-14.1	-1.7	-	-	-	-
773	723	L5103a		401.9	575.4	298	1.0	815.2	810.9	-4.4	-0.5	54.1	55.1	1.0	1.9
774	724	L5104a		388.4	559.6	298	1.0	815.4	803.3	-12.1	-1.5	52.9	54.0	1.1	2.1
775	725	L5105a		404.4	577.2	298	1.0	806.9	803.8	-3.1	-0.4	55.3	55.8	0.5	0.9
776	726	L5106a		392.1	560.3	298	1.01	805.3	796.7	-8.6	-1.1	53.6	54.2	0.6	1.1
777	727	L5107a		410.9	588.1	298	1.0	811.3	806.2	-5.2	-0.6	56.9	56.6	-0.3	-0.5
778	728	L5201a		447.1	754.5	298	1.0	968.8	949.5	-19.3	-2.0	-	-	-	-
779	729	L5202a		472.1	754.5	293	1.0	964.5	920.3	-44.2	-4.6	-	-	-	-
780	730	L5203a		473.1	754.5	293	1.0	991.7	930.3	-61.4	-6.2	-	-	-	-
781	731	L5204a		473.1	754.5	295	1.0	984.2	964.6	-19.6	-2.0	-	-	-	-
782	732	L5205a		460.6	754.5	292	1.0	979.8	943.1	-36.7	-3.7	-	-	-	-
783	733	L5206a	×	472.1	754.5	298	1.0	956.0	910.6	-45.4	-4.8	72.5	61.2	-11.3	-15.6
784	734	L5207a		475.2	754.5	293	1.0	997.0	960.1	-36.9	-3.7	-	-	-	-
785	735	L5208a		494.1	754.5	293	1.0	981.0	954.8	-26.2	-2.7	-	-	-	-
786	736	L5209a		482.1	754.5	297	1.0	969.1	957.6	-11.5	-1.2	74.6	73.6	-1.0	-1.3
787	737	L5210a		497.0	754.5	293	1.0	989.5	967.6	-21.9	-2.2	-	-	-	-
788	738	L5211a		512.1	754.5	298	1.0	989.7	979.6	-10.1	-1.0	86.8	86.5	-0.3	-0.4
789	739	L5301a		460.6	-	298	1.0	1103.6	1102.1	-1.5	-0.1	-	-	-	-
790	740	L6101a		393.1	596.0	298	0.01	813.9	817.0	3.1	0.4	53.8	52.4	-1.4	-2.6
791	741	L6102a		391.8	596.0	298	1.0	818.6	807.5	-11.1	-1.4	54.0	49.1	-4.9	-9.1
792	742	L6103a		409.9	596.0	298	1.0	824.5	833.0	8.5	1.0	-	-	-	-
793	743	L6104a		395.6	575.6	298	1.0	823.8	804.0	-19.8	-2.4	-	-	-	-
794	743	L6104b		395.6	575.6	337	1.0	-	-	-	-	40.1	43.4	3.3	8.3
795	744	L6105a		416.1	596.0	298	1.0	809.7	818.5	8.8	1.1	58.0	58.8	0.8	1.4
796	745	L6106a		394.6	559.5	298	1.0	809.5	791.7	-17.7	-2.2	54.7	50.3	-4.4	-8.0
797	746	L6107a		422.1	596.0	298	1.0	823.7	825.5	1.8	0.2	-	-	-	-
798	747	L6108a		407.4	596.0	298	1.0	824.7	813.3	-11.4	-1.4	58.2	55.8	-2.4	-4.1
799	748	L6109a		399.7	596.0	298	1.0	820.1	803.9	-16.2	-2.0	56.0	54.9	-1.1	-1.9
800	749	L6110a		404.9	574.4	298	1.0	803.0	805.6	2.6	0.3	-	-	-	-
801	749	L6110b		404.9	574.4	308	1.0	-	-	-	-	49.6	56.4	6.8	13.7
802	750	L6111a		419.6	596.0	298	1.0	829.3	826.2	-3.0	-0.4	60.3	60.1	-0.2	-0.4
803	751	L6112a		425.6	596.0	298	1.0	820.5	818.9	-1.6	-0.2	61.7	60.6	-1.1	-1.8
804	752	L6113a		421.1	604.4	298	1.0	820.6	818.6	-2.1	-0.3	59.4	60.0	0.6	1.0
805	753	L6114a		408.6	596.0	298	1.0	814.5	807.7	-6.8	-0.8	58.6	58.0	-0.6	-0.9
806	754	L6115a		424.9	603.5	298	1.0	809.7	811.3	1.5	0.2	-	-	-	-
807	755	L6116a		413.0	585.9	298	1.0	810.3	803.5	-6.8	-0.8	58.3	58.6	0.4	0.6
808	756	L6117a		430.1	610.3	298	1.0	815.5	814.5	-1.0	-0.1	59.6	61.5	1.9	3.2
809	757	L6201a		445.9	777.8	316	0.002	970.4	957.3	-13.1	-1.3	-	-	-	-
810	758	L6202a		505.8	777.8	298	1.0	963.8	949.6	-14.2	-1.5	-	-	-	-
811	759	L6203a		478.6	777.8	323	1.0	940.0	941.6	1.6	0.2	-	-	-	-
812	760	L6204a		480.0	777.8	298	1.0	964.5	921.7	-42.8	-4.4	-	-	-	-
813	761	L6205a		505.8	777.8	293	1.0	962.7	943.0	-19.7	-2.0	-	-	-	-
814	762	L6206a	×	470.6	777.8	298	1.0	918.5	882.3	-36.2	-3.9	68.6	59.7	-8.9	-13.0

Table S.12 – Comparison of experimental and simulated properties using GM combination rules (continued).

n_{sim}	n_{iso}	Code	Outlier	T_m [K]	T_b [K]	T [K]	P [bar]	ρ_{liq}[kg·m^{-3}] exp	sim	dev	err [%]	ΔH_{vap}[kJ·mol^{-1}] exp	sim	dev	err [%]
815	763	L6207a		484.6	777.8	287	1.0	990.6	917.3	-73.3	-7.4	-	-	-	-
816	764	L6208a		498.9	777.8	323	1.0	958.2	929.6	-28.6	-3.0	-	-	-	-
817	765	L6209a		480.0	777.8	277	1.0	996.0	988.0	-8.0	-0.8	-	-	-	-
818	766	L6210a		483.0	777.8	293	1.0	969.0	920.6	-48.4	-5.0	-	-	-	-
819	767	L6211a		494.4	777.8	293	1.0	964.5	955.3	-9.2	-0.9	-	-	-	-
820	768	L6212a		496.9	777.8	293	1.0	976.8	950.7	-26.1	-2.7	-	-	-	-
821	769	L6213a		480.0	777.8	298	1.0	967.7	932.1	-35.6	-3.7	-	-	-	-
822	770	L6214a		-	-	293	1.0	977.1	975.7	-1.4	-0.1	-	-	-	-
823	771	L6215a		493.4	777.8	295	1.0	973.7	924.2	-49.5	-5.1	-	-	-	-
824	772	L6216a		484.1	777.8	294	1.0	951.6	909.5	-42.1	-4.4	-	-	-	-
825	773	L6217a		493.9	777.8	323	1.0	939.8	921.9	-17.9	-1.9	-	-	-	-
826	774	L6218a		474.2	777.8	298	1.0	963.6	940.7	-22.9	-2.4	-	-	-	-
827	775	L6219a		521.5	777.8	293	1.0	972.6	973.8	1.2	0.1	-	-	-	-
828	776	L6220a		496.9	777.8	293	1.0	971.9	971.1	-0.8	-0.1	-	-	-	-
829	777	L6221a		508.1	777.8	295	1.0	958.0	937.4	-20.6	-2.1	-	-	-	-
830	778	L6222a		529.1	777.8	289	1.0	982.0	959.1	-22.9	-2.3	-	-	-	-
831	779	L6223a		497.1	777.8	385	0.01	917.4	867.4	-50.0	-5.4	-	-	-	-
832	780	L6224a		510.1	777.8	298	1.0	964.0	949.7	-14.3	-1.5	-	-	-	-
833	781	L6225a		516.1	777.8	318	1.0	968.3	951.8	-16.5	-1.7	-	-	-	-
834	781	L6225b		516.1	777.8	342	1.0	-	-	-	-	87.0	86.1	-0.9	-1.1
835	782	L6301a		-	-	293	1.0	1104.1	1087.0	-17.1	-1.5	-	-	-	-
836	783	L6302a		472.5	-	298	1.0	1100.0	1091.8	-8.1	-0.7	-	-	-	-
837	784	L7101a		404.1	619.7	298	1.0	833.5	841.2	7.7	0.9	-	-	-	-
838	784	L7101b		404.1	619.7	313	1.0	-	-	-	-	48.7	49.2	0.5	1.0
839	785	L7102a		430.1	619.7	293	1.0	846.6	855.7	9.1	1.1	-	-	-	-
840	786	L7103a		420.1	619.7	293	1.0	827.0	840.4	13.4	1.6	-	-	-	-
841	787	L7104a		413.1	619.7	298	1.0	837.3	825.3	-12.0	-1.4	-	-	-	-
842	788	L7105a		433.1	619.7	298	1.0	823.8	847.0	23.2	2.8	-	-	-	-
843	789	L7106a		409.1	619.7	298	1.0	822.4	825.6	3.2	0.4	-	-	-	-
844	790	L7107a		412.1	619.7	298	1.0	828.5	820.7	-7.8	-0.9	-	-	-	-
845	791	L7108a		411.1	619.7	293	1.0	811.9	820.9	9.0	1.1	-	-	-	-
846	792	L7109a		406.1	619.7	298	1.0	810.0	802.2	-7.8	-1.0	-	-	-	-
847	793	L7110a		426.1	619.7	294	1.0	836.0	827.8	-8.2	-1.0	-	-	-	-
848	794	L7111a		411.9	619.7	298	1.0	824.9	819.4	-5.5	-0.7	-	-	-	-
849	794	L7111b		411.9	619.7	322	1.0	-	-	-	-	53.6	53.7	0.1	0.1
850	795	L7112a		430.1	619.7	293	1.0	828.2	850.8	22.6	2.7	-	-	-	-
851	796	L7113a		415.6	619.7	298	1.0	839.6	823.0	-16.6	-2.0	57.3	52.2	-5.1	-9.0
852	797	L7114a		438.1	619.7	293	1.0	832.0	841.7	9.7	1.2	-	-	-	-
853	798	L7115a		426.1	619.7	293	1.0	837.9	836.9	-1.0	-0.1	-	-	-	-
854	799	L7116a		415.9	619.7	298	1.0	820.2	810.9	-9.2	-1.1	-	-	-	-
855	800	L7117a		433.1	619.7	293	1.0	815.1	827.7	12.6	1.5	-	-	-	-
856	801	L7118a		415.9	619.7	298	1.0	809.8	799.8	-10.0	-1.2	58.6	55.3	-3.3	-5.6
857	802	L7119a		435.1	619.7	298	1.0	832.7	836.3	3.6	0.4	-	-	-	-
858	803	L7120a		425.1	619.7	298	1.0	833.3	820.8	-12.5	-1.5	-	-	-	-
859	804	L7121a		437.1	619.7	296	1.0	836.0	838.9	2.9	0.3	-	-	-	-
860	805	L7122a		438.1	619.7	297	1.0	819.0	831.4	12.4	1.5	-	-	-	-
861	806	L7123a		425.1	619.7	298	1.0	822.0	817.1	-4.9	-0.6	-	-	-	-
862	807	L7124a		418.1	619.7	293	1.0	823.9	815.2	-8.7	-1.1	-	-	-	-

Table S.12 – Comparison of experimental and simulated properties using GM combination rules (continued).

n_{sim}	n_{iso}	Code	Outlier	T_m [K]	T_b [K]	T [K]	P [bar]	ρ_{liq}[kg·m^{-3}] exp	sim	dev	err [%]	ΔH_{vap}[kJ·mol^{-1}] exp	sim	dev	err [%]
863	808	L7125a		432.1	619.7	298	1.0	816.0	823.3	7.3	0.9	-	-	-	-
864	809	L7126a		424.1	619.7	298	1.0	817.7	816.4	-1.3	-0.2	-	-	-	-
865	810	L7127a		421.1	619.7	293	1.0	833.1	813.3	-19.8	-2.4	59.8	61.6	1.8	3.0
866	811	L7128a		424.1	619.7	293	1.0	813.1	811.3	-1.8	-0.2	-	-	-	-
867	812	L7129a		439.1	619.7	298	1.0	828.8	828.4	-0.4	-0.0	-	-	-	-
868	813	L7130a		445.1	619.7	298	1.0	824.5	824.7	0.2	0.0	-	-	-	-
869	814	L7131a		446.1	619.7	297	1.0	821.0	823.7	2.7	0.3	-	-	-	-
870	815	L7132a		436.1	619.7	293	1.0	827.0	827.3	0.3	0.0	-	-	-	-
871	816	L7133a		427.9	619.7	298	1.0	815.6	810.2	-5.4	-0.7	62.4	62.4	-0.0	-0.0
872	817	L7134a		429.9	619.7	298	1.0	816.5	810.3	-6.2	-0.8	-	-	-	-
873	818	L7135a		445.1	619.7	298	1.0	819.2	818.7	-0.5	-0.1	-	-	-	-
874	819	L7136a		432.4	608.3	298	1.0	813.4	810.5	-2.9	-0.4	62.1	63.2	1.1	1.8
875	820	L7137a		449.4	632.6	298	1.0	819.2	821.2	2.1	0.3	66.5	66.5	0.0	0.0
876	821	L7201a		525.9	799.3	293	1.0	961.3	975.8	14.5	1.5	-	-	-	-
877	822	L7202a		517.0	799.3	293	1.0	952.8	963.6	10.8	1.1	-	-	-	-
878	823	L7203a		517.0	799.3	293	1.0	929.6	894.8	-34.8	-3.7	-	-	-	-
879	824	L7204a		517.0	799.3	291	1.0	932.1	890.3	-41.8	-4.5	-	-	-	-
880	825	L7205a		507.1	799.3	334	1.0	949.0	922.9	-26.1	-2.7	-	-	-	-
881	826	L7206a		494.2	799.3	295	1.0	967.0	948.8	-18.2	-1.9	-	-	-	-
882	827	L7207a		508.2	799.3	293	1.0	967.2	959.2	-8.0	-0.8	-	-	-	-
883	828	L7208a		508.2	799.3	298	1.0	926.0	898.9	-27.1	-2.9	-	-	-	-
884	829	L7209a		485.4	799.3	293	1.0	962.5	955.2	-7.3	-0.8	-	-	-	-
885	830	L7210a		485.4	799.3	293	1.0	967.9	963.9	-4.0	-0.4	-	-	-	-
886	831	L7211a		515.1	799.3	298	1.0	950.4	944.2	-6.2	-0.7	-	-	-	-
887	832	L7212a		489.8	799.3	293	1.0	970.5	947.1	-23.4	-2.4	-	-	-	-
888	833	L7213a		485.4	799.3	298	1.0	962.0	943.4	-18.6	-1.9	-	-	-	-
889	834	L7214a		535.1	799.3	298	1.0	952.2	951.3	-0.9	-0.1	96.5	96.9	0.4	0.4
890	835	L7301a	vap	416.9	-	291	1.0	1075.0	-	-	-	0.0	-	-	-
891	836	L8101a	vap	433.1	641.4	298	1.0	815.1	-	-	-	0.0	-	-	-
892	837	L8102a	vap	425.1	641.4	298	1.0	842.9	-	-	-	0.0	-	-	-
893	838	L8103a	vap	419.6	641.4	293	1.0	823.7	-	-	-	0.0	-	-	-
894	839	L8104a	vap	430.1	641.4	293	1.0	849.2	-	-	-	0.0	-	-	-
895	840	L8105a	vap	431.1	641.4	293	1.0	840.8	-	-	-	0.0	-	-	-
896	841	L8106a	vap	424.1	641.4	293	1.0	832.4	-	-	-	0.0	-	-	-
897	842	L8107a	vap	431.1	641.4	293	1.0	808.0	-	-	-	0.0	-	-	-
898	843	L8108a	vap	433.0	641.4	293	1.0	857.6	-	-	-	0.0	-	-	-
899	844	L8109a	vap	433.1	641.4	293	1.0	829.5	-	-	-	0.0	-	-	-
900	845	L8110a	vap	432.1	641.4	293	1.0	834.1	-	-	-	0.0	-	-	-
901	846	L8111a	vap	433.0	641.4	293	1.0	845.7	-	-	-	0.0	-	-	-
902	847	L8112a		425.1	641.4	298	1.0	834.5	832.9	-1.6	-0.2	-	-	-	-
903	848	L8113a		431.4	641.4	293	1.0	837.1	834.5	-2.6	-0.3	-	-	-	-
904	849	L8114a		446.0	641.4	298	1.0	842.5	856.2	13.7	1.6	-	-	-	-
905	850	L8115a		429.2	641.4	293	1.0	834.2	834.3	0.1	0.0	-	-	-	-
906	851	L8116a		430.9	641.4	298	1.0	834.6	827.1	-7.5	-0.9	-	-	-	-
907	852	L8117a		433.2	641.4	298	1.0	831.0	826.3	-4.7	-0.6	-	-	-	-
908	853	L8118a		441.4	641.4	293	1.0	838.4	842.9	4.5	0.5	-	-	-	-
909	853	L8118b		441.4	641.4	348	1.0	-	-	-	-	54.7	56.3	1.6	2.9
910	854	L8119a		425.1	641.4	298	1.0	827.0	812.6	-14.4	-1.7	-	-	-	-

Table S.12 – Comparison of experimental and simulated properties using GM combination rules (continued).

n_{sim}	n_{iso}	Code	Outlier	T_m [K]	T_b [K]	T [K]	P [bar]	ρ_{liq}[kg·m^{-3}] exp	sim	dev	err [%]	ΔH_{vap}[kJ·mol^{-1}] exp	sim	dev	err [%]
911	855	L8120a		444.1	641.4	293	1.0	833.0	838.5	5.5	0.7	-	-	-	-
912	856	L8121a		426.1	641.4	293	1.0	833.9	828.2	-5.7	-0.7	-	-	-	-
913	857	L8122a		423.9	641.4	301	1.0	806.5	810.7	4.2	0.5	-	-	-	-
914	858	L8123a		425.6	641.4	298	1.0	811.5	804.7	-6.8	-0.8	-	-	-	-
915	859	L8124a		446.1	641.4	298	1.0	842.5	849.1	6.6	0.8	-	-	-	-
916	860	L8125a		456.1	641.4	293	1.0	849.8	854.2	4.4	0.5	-	-	-	-
917	861	L8126a		432.1	641.4	298	1.0	813.5	816.4	2.9	0.4	-	-	-	-
918	862	L8127a		432.1	641.4	298	1.0	834.2	826.6	-7.6	-0.9	-	-	-	-
919	863	L8128a		454.0	641.4	293	1.0	839.0	846.3	7.3	0.9	-	-	-	-
920	864	L8129a		445.6	641.4	293	1.0	826.5	840.1	13.6	1.6	-	-	-	-
921	865	L8130a		434.2	641.4	298	1.0	820.2	817.3	-2.9	-0.4	-	-	-	-
922	865	L8130b		434.2	641.4	345	1.0	-	-	-	-	54.8	50.9	-3.9	-7.0
923	866	L8131a		434.1	641.4	298	1.0	824.9	816.2	-8.7	-1.1	-	-	-	-
924	866	L8131b		434.1	641.4	353	1.0	-	-	-	-	54.7	49.7	-5.0	-9.2
925	867	L8132a		429.9	641.4	298	1.0	805.0	806.8	1.8	0.2	-	-	-	-
926	867	L8132b		429.9	641.4	358	1.0	-	-	-	-	53.1	50.2	-2.9	-5.5
927	868	L8133a		437.1	641.4	332	0.01	812.9	798.2	-14.7	-1.8	-	-	-	-
928	869	L8134a		428.6	641.4	298	1.0	794.2	822.0	27.8	3.5	-	-	-	-
929	869	L8134b	×	428.6	641.4	319	0.01	-	-	-	-	43.5	61.9	18.4	42.1
930	870	L8135a		437.9	641.4	298	1.0	833.5	821.3	-12.2	-1.5	-	-	-	-
931	870	L8135b		437.9	641.4	355	1.0	-	-	-	-	48.0	55.3	7.3	15.1
932	871	L8136a		439.2	641.4	298	1.0	817.6	820.8	3.2	0.4	-	-	-	-
933	871	L8136b		439.2	641.4	356	1.0	-	-	-	-	48.0	56.0	8.0	16.6
934	872	L8137a		440.8	641.4	298	1.0	821.0	815.4	-5.6	-0.7	-	-	-	-
935	872	L8137b		440.8	641.4	364	1.0	-	-	-	-	54.8	53.7	-1.1	-2.0
936	873	L8138a		449.6	641.4	293	1.0	827.3	835.7	8.4	1.0	-	-	-	-
937	873	L8138b		449.6	641.4	365	1.0	-	-	-	-	53.3	57.7	4.4	8.3
938	874	L8139a		426.8	641.4	298	1.0	814.1	818.0	3.9	0.5	-	-	-	-
939	875	L8140a		456.1	641.4	293	1.0	828.2	832.2	4.0	0.5	-	-	-	-
940	876	L8141a		444.8	641.4	298	1.0	799.0	819.9	20.9	2.6	-	-	-	-
941	877	L8142a		452.1	641.4	298	1.0	824.5	824.7	0.2	0.0	-	-	-	-
942	878	L8143a		445.1	641.4	298	1.0	810.0	818.4	8.4	1.0	-	-	-	-
943	879	L8144a		439.2	641.4	298	1.0	809.8	812.9	3.1	0.4	-	-	-	-
944	880	L8145a		433.0	641.4	298	1.0	776.6	814.9	38.3	4.9	-	-	-	-
945	881	L8146a		445.1	641.4	298	1.0	803.4	812.4	9.0	1.1	-	-	-	-
946	882	L8147a		453.0	641.4	301	1.0	829.0	832.1	3.1	0.4	-	-	-	-
947	883	L8148a		457.8	640.6	298	1.0	828.7	831.1	2.4	0.3	68.5	68.8	0.3	0.4
948	884	L8149a		456.4	641.4	298	1.0	806.0	828.7	22.7	2.8	-	-	-	-
949	885	L8150a		459.1	641.4	298	1.0	784.5	828.8	44.3	5.6	-	-	-	-
950	886	L8151a		459.8	641.4	298	1.0	815.2	829.1	13.9	1.7	-	-	-	-
951	887	L8152a		448.8	641.4	298	1.0	798.7	825.9	27.2	3.4	-	-	-	-
952	888	L8153a		449.8	641.4	298	1.0	815.9	812.9	-3.0	-0.4	67.2	66.9	-0.3	-0.4
953	889	L8154a		447.9	641.4	298	1.0	817.0	815.0	-2.0	-0.2	-	-	-	-
954	890	L8155a		460.9	641.4	298	1.0	817.6	822.9	5.4	0.7	-	-	-	-
955	891	L8156a		452.9	629.6	298	1.0	817.0	815.1	-1.9	-0.2	67.9	67.7	-0.2	-0.3
956	892	L8157a		468.4	652.5	298	1.0	821.8	825.9	4.1	0.5	70.1	71.1	1.0	1.4
957	893	L8201a		-	-	298	1.0	928.5	927.5	-1.0	-0.1	-	-	-	-
958	894	L8202a		-	-	293	1.0	917.2	888.8	-28.4	-3.1	-	-	-	-

Table S.12 – Comparison of experimental and simulated properties using GM combination rules (continued).

n_{sim}	n_{iso}	Code	Outlier	T_m [K]	T_b [K]	T [K]	P [bar]	ρ_{liq}[kg·m^{-3}] exp	sim	dev	err [%]	ΔH_{vap}[kJ·mol^{-1}] exp	sim	dev	err [%]
959	895	L8203a		-	-	298	1.0	929.0	917.7	-11.3	-1.2	-	-	-	-
960	896	L8204a		-	-	292	1.0	970.4	955.4	-15.0	-1.5	-	-	-	-
961	897	L8205a		-	-	298	1.0	959.0	937.5	-21.5	-2.2	-	-	-	-
962	898	L8206a		517.1	819.3	295	1.0	932.5	920.5	-12.0	-1.3	-	-	-	-
963	899	L8207a		478.1	819.3	298	1.0	918.0	895.6	-22.4	-2.4	-	-	-	-
964	900	L8208a		-	-	298	1.0	949.0	939.7	-9.3	-1.0	-	-	-	-
965	901	L8209a		-	-	298	1.0	943.0	935.0	-8.0	-0.9	-	-	-	-
966	902	L8210a	×	544.1	819.3	356	1.0	-	-	-	-	101.0	92.7	-8.3	-8.2
967	903	L9101a		447.1	661.5	298	1.0	852.6	845.4	-7.2	-0.8	-	-	-	-
968	904	L9102a		439.1	661.5	294	1.0	832.3	862.3	30.0	3.6	-	-	-	-
969	905	L9103a		446.2	661.5	293	1.0	846.2	853.4	7.2	0.9	-	-	-	-
970	906	L9104a		466.0	661.5	293	1.0	847.5	832.9	-14.6	-1.7	-	-	-	-
971	907	L9105a		439.0	661.5	293	1.0	835.0	825.2	-9.8	-1.2	-	-	-	-
972	908	L9106a		451.1	661.5	298	1.0	854.9	844.8	-10.1	-1.2	-	-	-	-
973	909	L9107a		444.1	661.5	293	1.0	848.8	855.7	6.9	0.8	-	-	-	-
974	910	L9108a		439.0	661.5	293	1.0	825.6	830.9	5.3	0.6	-	-	-	-
975	911	L9109a		466.0	661.5	288	1.0	835.3	846.9	11.6	1.4	-	-	-	-
976	912	L9110a		442.0	661.5	293	1.0	825.0	838.6	13.6	1.6	-	-	-	-
977	913	L9111a		452.0	661.5	288	1.0	861.0	850.8	-10.2	-1.2	-	-	-	-
978	914	L9112a		457.2	661.5	298	1.0	844.5	836.8	-7.7	-0.9	-	-	-	-
979	915	L9113a		452.0	661.5	298	1.0	899.4	834.6	-64.8	-7.2	-	-	-	-
980	916	L9114a		447.1	661.5	294	1.0	838.3	836.2	-2.1	-0.3	-	-	-	-
981	917	L9115a		475.1	661.5	293	1.0	846.0	857.0	11.0	1.3	-	-	-	-
982	918	L9116a		451.0	661.5	298	1.0	823.7	833.4	9.6	1.2	-	-	-	-
983	919	L9117a		451.1	661.5	298	1.0	833.4	831.4	-2.0	-0.2	-	-	-	-
984	920	L9118a		461.1	661.5	298	1.0	837.0	849.7	12.7	1.5	-	-	-	-
985	921	L9119a		445.1	661.5	295	1.0	839.6	825.4	-14.2	-1.7	-	-	-	-
986	922	L9120a		450.0	661.5	298	1.0	821.8	822.2	0.3	0.0	-	-	-	-
987	923	L9121a		444.6	661.5	293	1.0	824.2	821.9	-2.3	-0.3	-	-	-	-
988	924	L9122a		446.1	661.5	289	1.0	828.5	825.9	-2.6	-0.3	-	-	-	-
989	925	L9123a		466.1	661.5	298	1.0	823.6	839.0	15.4	1.9	67.9	70.7	2.8	4.1
990	926	L9124a		455.0	661.5	298	1.0	828.0	817.2	-10.8	-1.3	-	-	-	-
991	927	L9125a		-	-	295	1.0	830.0	817.3	-12.7	-1.5	-	-	-	-
992	928	L9126a		444.1	661.5	293	1.0	818.6	813.7	-4.9	-0.6	-	-	-	-
993	929	L9127a		452.0	661.5	293	1.0	827.5	841.7	14.2	1.7	-	-	-	-
994	930	L9128a		460.1	661.5	293	1.0	859.2	842.3	-16.9	-2.0	-	-	-	-
995	931	L9129a		448.1	661.5	293	1.0	814.8	821.8	7.0	0.9	-	-	-	-
996	932	L9130a		467.1	661.5	273	1.0	878.7	842.6	-36.1	-4.1	-	-	-	-
997	933	L9131a		451.0	661.5	298	1.0	806.4	817.5	11.1	1.4	65.2	70.2	5.0	7.7
998	934	L9132a		452.1	661.5	298	1.0	829.9	829.5	-0.4	-0.0	-	-	-	-
999	935	L9133a		455.4	661.5	298	1.0	829.9	830.2	0.3	0.0	-	-	-	-
1000	936	L9134a		454.1	661.5	298	1.0	824.6	821.3	-3.2	-0.4	-	-	-	-
1001	937	L9135a		-	-	298	1.0	827.5	821.0	-6.5	-0.8	-	-	-	-
1002	938	L9136a		455.0	661.5	293	1.0	843.9	836.1	-7.8	-0.9	-	-	-	-
1003	939	L9137a		451.1	661.5	298	1.0	813.4	812.1	-1.3	-0.2	-	-	-	-
1004	940	L9138a		466.1	661.5	298	1.0	835.8	845.5	9.7	1.2	-	-	-	-
1005	941	L9139a		455.0	661.5	298	1.0	815.6	825.5	9.9	1.2	-	-	-	-
1006	942	L9140a		459.0	661.5	298	1.0	843.7	825.8	-17.9	-2.1	-	-	-	-

Table S.12 – Comparison of experimental and simulated properties using GM combination rules (continued).

n_{sim}	n_{iso}	Code	Outlier	T_m [K]	T_b [K]	T [K]	P [bar]	ρ_{liq}[kg·m^{-3}] exp	sim	dev	err [%]	ΔH_{vap}[kJ·mol^{-1}] exp	sim	dev	err [%]
1007	943	L9141a	vap	453.1	661.5	291	1.0	834.0	-	-	-	0.0	-	-	-
1008	944	L9142a	vap	454.0	661.5	300	1.0	831.0	-	-	-	0.0	-	-	-
1009	945	L9143a	vap	457.1	661.5	293	1.0	827.0	-	-	-	0.0	-	-	-
1010	946	L9144a	vap	468.1	661.5	293	1.0	828.8	-	-	-	0.0	-	-	-
1011	947	L9145a	vap	465.1	661.5	293	1.0	825.6	-	-	-	0.0	-	-	-
1012	948	L9146a	vap	466.0	661.5	298	1.0	820.8	-	-	-	0.0	-	-	-
1013	949	L9147a	vap	455.0	661.5	296	1.0	822.0	-	-	-	0.0	-	-	-
1014	950	L9148a	vap	459.0	661.5	301	1.0	832.0	-	-	-	0.0	-	-	-
1015	951	L9149a	vap	-	-	300	1.0	823.0	-	-	-	0.0	-	-	-
1016	952	L9150a	vap	454.0	661.5	298	1.0	821.0	-	-	-	0.0	-	-	-
1017	953	L9151a	vap	457.1	661.5	298	1.0	815.0	-	-	-	0.0	-	-	-
1018	954	L9152a	vap	455.0	661.5	293	1.0	813.6	-	-	-	0.0	-	-	-
1019	955	L9153a		459.0	661.5	285	1.0	840.2	826.8	-13.4	-1.6	-	-	-	-
1020	956	L9154a		480.1	661.5	296	1.0	834.0	837.3	3.3	0.4	-	-	-	-
1021	957	L9155a		472.0	661.5	298	1.0	848.0	835.9	-12.1	-1.4	-	-	-	-
1022	958	L9156a		473.0	661.5	301	1.0	820.0	830.0	10.0	1.2	-	-	-	-
1023	959	L9157a		473.0	661.5	297	1.0	828.0	832.5	4.5	0.5	-	-	-	-
1024	960	L9158a		473.0	661.5	297	1.0	827.0	831.6	4.6	0.6	-	-	-	-
1025	961	L9159a		473.0	661.5	277	1.0	841.8	844.7	2.9	0.3	-	-	-	-
1026	962	L9160a		468.2	661.5	298	1.0	818.3	818.0	-0.3	-0.0	71.4	71.6	0.2	0.3
1027	963	L9161a		466.1	661.5	293	1.0	826.3	821.7	-4.6	-0.6	71.5	72.3	0.8	1.1
1028	964	L9162a		467.9	661.5	298	1.0	823.5	819.6	-4.0	-0.5	70.9	71.6	0.7	1.0
1029	965	L9163a		479.1	661.5	298	1.0	826.0	827.7	1.7	0.2	-	-	-	-
1030	966	L9164a		471.6	649.6	298	1.0	819.4	819.4	0.0	0.0	72.9	72.4	-0.5	-0.6
1031	967	L9165a		486.2	670.7	298	1.0	824.6	829.1	4.5	0.5	72.2	75.8	3.6	5.0
1032	968	L9201a		-	-	301	1.0	920.7	890.4	-30.3	-3.3	-	-	-	-
1033	969	L9202a		-	-	290	1.0	913.8	888.2	-25.6	-2.8	-	-	-	-
1034	970	L9203a		-	-	291	1.0	902.0	884.8	-17.2	-1.9	-	-	-	-
1035	971	L9204a		535.1	837.9	323	1.0	929.0	916.2	-12.8	-1.4	-	-	-	-
1036	971	L9204b		535.1	837.9	460	1.0	-	-	-	-	67.2	62.2	-5.0	-7.4
1037	972	L9205a		-	-	291	1.0	958.0	949.7	-8.3	-0.9	-	-	-	-
1038	973	L9206a		-	-	293	1.0	941.6	946.7	5.1	0.5	-	-	-	-
1039	974	L9207a		-	-	293	1.0	937.0	933.5	-3.5	-0.4	-	-	-	-
1040	975	L9208a		571.1	837.9	293	1.0	929.5	927.5	-2.0	-0.2	-	-	-	-
1041	976	L9209a		-	-	298	1.0	944.0	930.1	-13.9	-1.5	-	-	-	-
1042	977	L9210a		558.1	837.9	323	1.0	-	-	-	-	110.0	102.3	-7.7	-7.0
1043	978	L0101a		464.1	680.4	293	1.0	862.4	859.5	-2.9	-0.3	-	-	-	-
1044	979	L0102a		475.1	680.4	293	1.0	874.2	877.2	3.0	0.3	-	-	-	-
1045	980	L0103a		474.1	680.4	293	1.0	874.5	874.3	-0.2	-0.0	-	-	-	-
1046	981	L0104a		465.1	680.4	293	1.0	858.4	867.7	9.3	1.1	-	-	-	-
1047	982	L0105a		513.5	680.4	293	1.0	839.3	851.2	11.9	1.4	-	-	-	-
1048	983	L0106a		463.1	680.4	293	1.0	854.9	870.0	15.1	1.8	-	-	-	-
1049	984	L0107a		468.1	680.4	293	1.0	862.3	864.6	2.3	0.3	-	-	-	-
1050	985	L0108a		475.0	680.4	293	1.0	837.8	836.9	-0.9	-0.1	-	-	-	-
1051	986	L0109a		467.6	680.4	298	1.0	859.1	852.0	-7.1	-0.8	-	-	-	-
1052	987	L0110a		459.1	680.4	293	1.0	848.7	852.6	3.9	0.5	-	-	-	-
1053	988	L0111a		460.0	680.4	293	1.0	842.8	833.7	-9.1	-1.1	-	-	-	-
1054	989	L0112a		468.8	680.4	293	1.0	854.3	839.1	-15.2	-1.8	-	-	-	-

Table S.12 – Comparison of experimental and simulated properties using GM combination rules (continued).

n_{sim}	n_{iso}	Code	Outlier	T_m [K]	T_b [K]	T [K]	P [bar]	ρ_{liq}[kg·m^{-3}] exp	sim	dev	err [%]	ΔH_{vap}[kJ·mol^{-1}] exp	sim	dev	err [%]
1055	990	L0113a		503.4	687.3	298	1.0	-	-	-	-	80.9	80.7	-0.2	-0.3
1056	991	L0114a		454.1	680.4	293	1.0	833.0	829.6	-3.4	-0.4	-	-	-	-
1057	992	L0115a		465.1	680.4	293	1.0	853.7	844.5	-9.2	-1.1	-	-	-	-
1058	993	L0116a		460.0	680.4	293	1.0	860.6	866.9	6.3	0.7	-	-	-	-
1059	994	L0117a		463.0	680.4	293	1.0	839.8	837.3	-2.5	-0.3	-	-	-	-
1060	995	L0118a		460.1	680.4	293	1.0	833.9	852.9	19.0	2.3	-	-	-	-
1061	996	L0119a		463.0	680.4	293	1.0	823.6	836.6	13.0	1.6	-	-	-	-
1062	997	L0120a		455.1	680.4	294	1.0	823.0	821.7	-1.3	-0.2	-	-	-	-
1063	998	L0121a		466.0	680.4	293	1.0	826.5	836.1	9.6	1.2	-	-	-	-
1064	999	L0122a		466.1	680.4	291	1.0	833.0	829.6	-3.4	-0.4	-	-	-	-
1065	1000	L0123a		463.1	680.4	298	1.0	841.3	838.5	-2.8	-0.3	-	-	-	-
1066	1001	L0124a		466.1	680.4	293	1.0	845.5	843.3	-2.2	-0.3	-	-	-	-
1067	1002	L0125a		462.2	680.4	298	1.0	824.9	834.1	9.2	1.1	-	-	-	-
1068	1003	L0126a		464.0	680.4	301	1.0	825.8	823.0	-2.8	-0.3	-	-	-	-
1069	1004	L0127a		466.0	680.4	298	1.0	837.0	825.3	-11.7	-1.4	-	-	-	-
1070	1005	L0128a		464.0	680.4	293	1.0	823.2	827.4	4.2	0.5	-	-	-	-
1071	1006	L0129a		465.1	680.4	295	1.0	834.7	829.0	-5.7	-0.7	-	-	-	-
1072	1007	L0130a		465.1	680.4	273	1.0	842.1	842.2	0.1	0.0	-	-	-	-
1073	1008	L0131a		469.1	680.4	298	1.0	826.0	822.7	-3.3	-0.4	-	-	-	-
1074	1009	L0132a		463.1	680.4	293	1.0	821.2	834.9	13.7	1.7	-	-	-	-
1075	1010	L0133a		485.0	680.4	293	1.0	825.7	826.3	0.6	0.1	-	-	-	-
1076	1011	L0134a		485.0	680.4	293	1.0	827.3	822.7	-4.6	-0.6	-	-	-	-
1077	1012	L0135a		485.0	680.4	298	1.0	821.1	815.6	-5.5	-0.7	-	-	-	-
1078	1013	L0136a		466.0	680.4	290	1.0	865.0	849.2	-15.8	-1.8	-	-	-	-
1079	1014	L0137a		486.1	680.4	293	1.0	832.2	841.3	9.1	1.1	-	-	-	-
1080	1015	L0138a		464.0	680.4	298	1.0	821.5	829.1	7.6	0.9	-	-	-	-
1081	1016	L0139a		485.0	680.4	293	1.0	829.1	829.4	0.3	0.0	-	-	-	-
1082	1017	L0140a		467.1	680.4	293	1.0	815.2	826.9	11.7	1.4	-	-	-	-
1083	1018	L0141a		477.1	680.4	277	1.0	846.0	846.7	0.7	0.1	-	-	-	-
1084	1019	L0142a		475.1	680.4	293	1.0	814.0	822.4	8.4	1.0	-	-	-	-
1085	1020	L0143a		467.1	680.4	298	1.0	828.3	833.3	5.0	0.6	-	-	-	-
1086	1021	L0144a		466.0	680.4	298	1.0	826.8	833.3	6.5	0.8	-	-	-	-
1087	1022	L0145a		472.1	680.4	298	1.0	836.1	832.6	-3.5	-0.4	-	-	-	-
1088	1023	L0146a		481.1	680.4	293	1.0	830.0	843.9	13.9	1.7	-	-	-	-
1089	1024	L0147a		475.1	680.4	298	1.0	825.4	825.6	0.3	0.0	-	-	-	-
1090	1025	L0148a		482.0	680.4	298	1.0	827.1	824.7	-2.4	-0.3	-	-	-	-
1091	1026	L0149a		482.0	680.4	298	1.0	826.5	824.4	-2.0	-0.2	-	-	-	-
1092	1027	L0150a		487.0	680.4	298	1.0	807.8	818.1	10.3	1.3	-	-	-	-
1093	1028	L0151a		488.4	680.4	303	1.0	834.1	839.6	5.5	0.7	-	-	-	-
1094	1029	L0152a		482.0	680.4	300	1.0	826.0	827.3	1.3	0.2	-	-	-	-
1095	1030	L0153a		487.0	680.4	295	1.0	845.0	829.7	-15.3	-1.8	-	-	-	-
1096	1031	L0154a		481.6	680.4	298	1.0	824.5	824.2	-0.3	-0.0	-	-	-	-
1097	1032	L0155a		481.1	680.4	293	1.0	828.6	841.0	12.4	1.5	-	-	-	-
1098	1033	L0156a		472.0	680.4	298	1.0	839.6	828.2	-11.4	-1.4	-	-	-	-
1099	1034	L0157a		483.0	680.4	291	1.0	830.0	839.5	9.5	1.1	-	-	-	-
1100	1035	L0158a		485.1	680.4	298	1.0	832.0	831.8	-0.2	-0.0	-	-	-	-
1101	1036	L0159a		-	-	288	1.0	830.3	837.3	7.0	0.8	-	-	-	-
1102	1037	L0160a		487.0	680.4	293	1.0	833.2	829.2	-4.0	-0.5	-	-	-	-

Table S.12 – Comparison of experimental and simulated properties using GM combination rules (continued).

n_{sim}	n_{iso}	Code	Outlier	T_m [K]	T_b [K]	T [K]	P [bar]	ρ_{liq}[kg·m^{-3}] exp	sim	dev	err [%]	ΔH_{vap}[kJ·mol^{-1}] exp	sim	dev	err [%]
1103	1038	L0161a		482.0	680.4	298	1.0	820.0	820.8	0.8	0.1	-	-	-	-
1104	1039	L0162a		482.0	680.4	293	1.0	821.5	823.1	1.6	0.2	-	-	-	-
1105	1040	L0163a		-	-	289	1.0	836.0	841.7	5.7	0.7	-	-	-	-
1106	1041	L0164a		491.1	680.4	293	1.0	832.2	837.5	5.3	0.6	-	-	-	-
1107	1042	L0165a		487.0	680.4	297	1.0	831.0	835.8	4.8	0.6	-	-	-	-
1108	1043	L0166a		489.1	680.4	300	1.0	826.0	833.3	7.3	0.9	-	-	-	-
1109	1044	L0167a		487.0	680.4	296	1.0	837.0	835.9	-1.1	-0.1	-	-	-	-
1110	1045	L0168a		487.0	680.4	298	1.0	828.2	834.5	6.3	0.8	-	-	-	-
1111	1046	L0169a		495.1	680.4	288	1.0	833.6	840.7	7.0	0.8	-	-	-	-
1112	1047	L0170a		474.1	680.4	298	1.0	820.6	821.8	1.2	0.1	-	-	-	-
1113	1048	L0171a		483.1	680.4	293	1.0	825.0	825.3	0.3	0.0	-	-	-	-
1114	1049	L0172a		483.0	680.4	298	1.0	822.9	822.8	-0.1	-0.0	-	-	-	-
1115	1050	L0201a		-	-	288	1.0	910.2	891.8	-18.5	-2.0	-	-	-	-
1116	1051	L0202a		503.1	855.5	298	1.0	943.5	951.2	7.7	0.8	-	-	-	-
1117	1052	L0203a		-	-	285	1.0	911.8	896.9	-14.9	-1.6	-	-	-	-
1118	1053	L0204a		-	-	293	1.0	916.5	899.4	-17.1	-1.9	-	-	-	-
1119	1054	L0205a		538.1	855.5	293	1.0	926.0	929.5	3.5	0.4	-	-	-	-
1120	1055	L0206a		-	-	293	1.0	948.1	935.3	-12.8	-1.4	-	-	-	-
1121	1056	L0207a		-	-	293	1.0	936.2	944.2	8.0	0.9	-	-	-	-
1122	1057	L0208a		-	-	301	1.0	915.5	906.8	-8.7	-1.0	-	-	-	-
1123	1058	L0209a		-	-	298	1.0	930.7	939.4	8.7	0.9	-	-	-	-
1124	1059	L0210a		572.1	855.5	353	1.0	883.0	895.7	12.7	1.4	-	-	-	-
1125	1060	D1201a	×	373.7	588.0	298	1.0	1213.6	1332.7	119.1	9.8	46.3	43.3	-3.0	-6.5
1126	1061	D2201a	×	391.1	592.0	298	1.01	1043.5	1135.8	92.3	8.8	50.3	50.4	0.1	0.2
1127	1062	D3201a		414.3	600.8	298	1.01	988.1	1047.3	59.2	6.0	54.9	53.9	-1.0	-1.7
1128	1063	D4201a		427.6	-	298	1.0	943.9	997.6	53.7	5.7	56.3	56.5	0.2	0.3
1129	1064	D4202a		436.9	615.7	298	1.0	952.9	1008.7	55.8	5.9	58.2	58.7	0.5	0.9
1130	1065	D5201a		436.9	631.0	310	1.0	907.5	961.9	54.5	6.0	-	-	-	-
1131	1065	D5201b		436.9	631.0	320	1.0	-	-	-	-	57.6	50.8	-6.8	-11.8
1132	1066	D5202a		450.1	631.0	298	1.0	934.7	979.5	44.8	4.8	-	-	-	-
1133	1067	D5203a		448.2	629.1	298	1.0	924.1	979.1	55.1	6.0	61.2	61.7	0.5	0.8
1134	1068	D5204a		458.6	639.2	298	1.0	934.4	983.5	49.1	5.3	63.0	63.3	0.3	0.5
1135	1069	D6201a		459.1	654.6	293	1.0	927.5	977.0	49.5	5.3	-	-	-	-
1136	1069	D6201b	×	459.1	654.6	370	1.0	-	-	-	-	59.4	49.2	-10.2	-17.2
1137	1070	D6202a		457.1	654.6	298	1.0	908.0	971.4	63.4	7.0	64.0	63.8	-0.2	-0.2
1138	1071	D6203a		464.9	654.6	293	1.0	927.5	974.3	46.9	5.1	-	-	-	-
1139	1072	D6204a		466.1	654.6	298	1.0	923.5	968.5	45.0	4.9	-	-	-	-
1140	1073	D6205a		-	654.6	298	1.0	918.2	963.6	45.4	4.9	-	-	-	-
1141	1074	D6206a		-	654.6	298	1.0	923.0	968.9	45.9	5.0	-	-	-	-
1142	1075	D6207a		473.6	654.6	298	1.0	917.0	964.2	47.2	5.1	-	-	-	-
1143	1076	D6208a		478.9	660.2	298	1.0	922.9	966.7	43.8	4.7	69.2	68.2	-1.0	-1.4
1144	1077	D7201a		-	676.3	293	1.0	885.6	962.9	77.3	8.7	-	-	-	-
1145	1078	D7202a		-	-	298	1.0	934.8	973.1	38.3	4.1	-	-	-	-
1146	1079	D7203a		-	-	298	1.0	910.0	951.6	41.6	4.6	-	-	-	-
1147	1080	D7204a		-	-	298	1.0	923.9	962.4	38.5	4.2	-	-	-	-
1148	1081	D7205a		-	676.3	298	1.0	909.8	954.3	44.5	4.9	-	-	-	-
1149	1082	D7206a		482.6	-	298	1.0	909.0	950.4	41.4	4.6	-	-	-	-
1150	1083	D7207a		486.1	676.3	293	1.0	918.7	960.2	41.5	4.5	-	-	-	-

Table S.12 – Comparison of experimental and simulated properties using GM combination rules (continued).

n_{sim}	n_{iso}	Code	Outlier	T_m [K]	T_b [K]	T [K]	P [bar]	$\rho_{\text{liq}}[\text{kg}\cdot\text{m}^{-3}]$ exp	sim	dev	err [%]	$\Delta H_{\text{vap}}[\text{kJ}\cdot\text{mol}^{-1}]$ exp	sim	dev	err [%]
1151	1084	D7208a		489.1	676.3	293	1.0	916.3	956.0	39.7	4.3	-	-	-	-
1152	1085	D7209a		496.1	677.3	298	1.0	913.5	953.2	39.7	4.3	72.9	73.1	0.2	0.3
1153	1086	D8201a		-	-	298	1.0	919.9	971.8	51.9	5.6	-	-	-	-
1154	1087	D8202a		-	-	293	1.0	929.0	965.9	36.9	4.0	-	-	-	-
1155	1088	D8203a		-	-	298	1.0	900.9	960.9	60.0	6.7	-	-	-	-
1156	1089	D8204a		-	-	298	1.0	902.0	945.2	43.2	4.8	-	-	-	-
1157	1090	D8205a		494.1	696.5	298	1.0	904.0	945.1	41.1	4.6	74.8	75.5	0.7	0.9
1158	1091	D8206a		501.1	673.2	298	1.0	903.0	943.8	40.8	4.5	75.6	75.7	0.1	0.1
1159	1092	D8207a		-	-	293	1.0	910.5	949.2	38.7	4.2	-	-	-	-
1160	1093	D8208a		513.0	694.3	298	1.01	906.0	943.1	37.1	4.1	81.2	78.1	-3.1	-3.9
1161	1094	D9201a		-	-	298	1.0	910.2	953.8	43.6	4.8	-	-	-	-
1162	1095	D9202a		456.9	715.3	298	1.0	893.5	935.1	41.6	4.7	-	-	-	-
1163	1096	D9203a		456.9	715.3	277	1.0	909.7	948.4	38.7	4.3	-	-	-	-
1164	1097	D9204a		456.9	715.3	296	1.0	899.0	938.2	39.2	4.4	-	-	-	-
1165	1098	D9205a		528.8	710.7	298	1.0	901.6	935.4	33.7	3.7	-	-	-	-
1166	1098	D9205b		528.8	710.7	304	1.0	-	-	-	-	85.3	81.6	-3.7	-4.3
1167	1099	D0201a		473.7	733.0	293	1.0	902.2	934.9	32.7	3.6	-	-	-	-
1168	1100	D0202a		-	-	298	1.0	912.9	941.9	29.0	3.2	-	-	-	-
1169	1101	D0203a		-	-	291	1.0	897.0	932.8	35.8	4.0	-	-	-	-
1170	1102	D0204a		-	-	292	1.0	897.8	932.6	34.8	3.9	-	-	-	-
1171	1103	D0205a	×	-	733.0	293	1.0	848.1	931.1	83.0	9.8	-	-	-	-
1172	1104	D0206a		-	733.0	298	1.0	893.0	924.8	31.8	3.6	-	-	-	-
1173	1105	D0207a		473.7	733.0	293	1.0	898.3	933.7	35.4	3.9	-	-	-	-
1174	1106	D0208a		-	733.0	293	1.0	908.6	937.8	29.2	3.2	-	-	-	-
1175	1107	D0209a		543.1	722.1	313	1.0	881.7	918.1	36.4	4.1	88.6	84.9	-3.7	-4.2
1176	1108	N1101a		266.8	430.1	267	1.0	694.2	742.2	48.0	6.9	26.1	23.5	-2.7	-10.2
1177	1108	N1101b		266.8	430.1	298	3.52	655.0	671.9	16.9	2.6	-	-	-	-
1178	1109	N2101a		289.7	456.1	288	1.0	688.6	732.8	44.3	6.4	27.4	27.9	0.5	1.9
1179	1110	N2102a	×	280.0	437.2	283	1.0	667.9	840.8	172.9	25.9	27.2	30.8	3.6	13.2
1180	1110	N2102b	×	280.0	437.2	298	1.96	650.0	833.2	183.3	28.2	-	-	-	-
1181	1111	N2201a	×	390.4	593.0	298	0.02	892.8	1069.7	176.9	19.8	45.0	49.2	4.2	9.3
1182	1112	N3101a		305.6	471.9	298	1.01	683.9	687.8	3.8	0.6	28.4	26.3	-2.1	-7.4
1183	1113	N3102a		321.6	496.9	298	1.01	712.8	735.4	22.7	3.2	31.3	30.4	-0.9	-2.8
1184	1114	N3103a		276.0	433.2	273	1.0	655.7	673.2	17.5	2.7	22.9	23.1	0.2	0.7
1185	1114	N3103b		276.0	433.2	298	2.33	628.9	645.0	16.0	2.6	-	-	-	-
1186	1115	N3201a	×	388.1	-	298	1.0	841.0	927.4	86.4	10.3	-	-	-	-
1187	1116	N3202a		392.4	585.0	298	1.0	-	-	-	-	44.2	44.6	0.4	1.0
1188	1117	N3203a	×	412.9	608.0	298	1.0	884.0	1003.9	119.9	13.6	50.2	52.2	2.0	4.1
1189	1118	N4101a		317.6	483.9	298	1.0	690.1	690.4	0.4	0.1	29.6	27.0	-2.6	-8.7
1190	1119	N4102a		336.1	514.3	298	1.01	717.8	718.5	0.7	0.1	32.6	29.9	-2.7	-8.2
1191	1120	N4103a		340.9	522.4	298	1.0	729.1	740.8	11.6	1.6	33.8	32.3	-1.5	-4.4
1192	1121	N4104a		350.6	531.9	298	1.0	736.8	755.1	18.3	2.5	35.6	34.7	-0.9	-2.4
1193	1122	N4105a		323.6	482.4	288	1.0	740.0	727.9	-12.1	-1.6	30.7	29.7	-1.0	-3.4
1194	1123	N4106a		328.6	496.6	298	1.0	701.2	769.1	67.9	9.7	31.2	35.0	3.8	12.1
1195	1124	N4107a		336.1	482.4	298	0.2	711.9	748.8	36.9	5.2	-	-	-	-
1196	1125	N4108a		310.1	500.0	298	1.0	669.4	678.6	9.2	1.4	-	-	-	-
1197	1126	N4201a		378.6	-	298	1.0	803.0	839.9	36.9	4.6	-	-	-	-
1198	1127	N4202a	×	393.1	-	288	1.0	828.0	932.4	104.4	12.6	-	-	-	-

Table S.12 – Comparison of experimental and simulated properties using GM combination rules (continued).

n_{sim}	n_{iso}	Code	Outlier	T_m [K]	T_b [K]	T [K]	P [bar]	$\rho_{\mathrm{liq}}[\mathrm{kg{\cdot}m^{-3}}]$ exp	sim	dev	err [%]	$\Delta H_{\mathrm{vap}}[\mathrm{kJ{\cdot}mol^{-1}}]$ exp	sim	dev	err [%]
1199	1128	N4203a		396.1	-	298	1.0	841.0	902.0	61.0	7.2	45.8	41.9	-3.9	-8.5
1200	1129	N4204a		402.1	-	298	1.0	837.0	892.1	55.1	6.6	-	-	-	-
1201	1130	N4205a		413.1	-	298	1.0	-	-	-	-	53.1	47.6	-5.5	-10.4
1202	1131	N4206a		-	-	298	1.0	-	-	-	-	46.9	47.0	0.1	0.2
1203	1132	N4207a	×	431.6	-	298	1.0	877.0	963.6	86.6	9.9	55.2	56.5	1.3	2.3
1204	1133	N5101a		350.1	557.7	298	0.11	727.6	727.4	-0.2	-0.0	-	-	-	-
1205	1134	N5102a		350.1	557.7	298	0.1	728.1	753.2	25.1	3.5	-	-	-	-
1206	1135	N5103a		357.1	557.7	298	0.08	753.0	740.3	-12.7	-1.7	-	-	-	-
1207	1136	N5104a		362.1	557.7	291	1.0	711.3	746.9	35.6	5.0	-	-	-	-
1208	1137	N5105a		368.6	557.7	298	1.0	750.5	764.9	14.4	1.9	-	-	-	-
1209	1138	N5106a		370.1	557.7	298	1.0	744.3	766.1	21.8	2.9	-	-	-	-
1210	1139	N5107a		377.6	557.7	298	0.04	751.0	769.9	18.9	2.5	40.1	39.2	-0.9	-2.1
1211	1140	N5108a	×	350.1	517.7	298	1.0	727.0	837.6	110.6	15.2	32.3	34.7	2.4	7.4
1212	1141	N5109a		351.6	517.7	298	0.1	720.0	724.9	4.9	0.7	-	-	-	-
1213	1142	N5110a		346.1	517.7	298	0.12	730.0	731.3	1.3	0.2	-	-	-	-
1214	1143	N5111a		349.1	517.7	298	1.0	-	-	-	-	33.1	33.1	-0.0	-0.1
1215	1144	N5112a		353.4	517.7	298	0.11	726.7	737.5	10.8	1.5	-	-	-	-
1216	1145	N5113a		364.2	517.7	298	0.05	728.1	748.4	20.3	2.8	-	-	-	-
1217	1146	N5114a		339.2	517.7	298	1.0	710.6	700.7	-9.9	-1.4	31.9	29.7	-2.2	-7.0
1218	1147	N5115a		339.1	517.7	298	1.0	701.5	702.4	0.9	0.1	31.8	30.7	-1.1	-3.3
1219	1148	N5116a		339.1	517.7	298	0.21	695.6	706.7	11.2	1.6	-	-	-	-
1220	1149	N5201a		356.1	-	291	1.0	749.1	779.2	30.1	4.0	-	-	-	-
1221	1149	N5201b		356.1	-	298	1.0	-	-	-	-	33.1	35.6	2.5	7.5
1222	1150	N5202a		406.6	-	293	1.0	827.2	846.1	18.9	2.3	42.2	44.9	2.7	6.5
1223	1151	N5203a		437.1	-	298	1.0	-	-	-	-	54.9	50.9	-4.0	-7.2
1224	1152	N5204a		452.1	-	298	1.0	873.0	940.6	67.6	7.7	58.7	60.0	1.3	2.1
1225	1153	N6101a		375.1	589.1	298	0.04	760.1	766.7	6.6	0.9	-	-	-	-
1226	1154	N6102a		381.6	589.1	298	0.02	745.7	754.9	9.2	1.2	-	-	-	-
1227	1155	N6103a		404.6	589.1	298	1.0	763.0	782.2	19.3	2.5	45.0	43.9	-1.1	-2.6
1228	1156	N6104a	×	351.1	549.1	298	1.0	716.1	812.5	96.4	13.5	-	-	-	-
1229	1157	N6105a		357.1	523.1	298	1.0	712.3	729.4	17.1	2.4	34.5	34.9	0.4	1.0
1230	1158	N6106a		371.1	549.1	298	0.04	729.8	735.1	5.4	0.7	-	-	-	-
1231	1159	N6107a		371.4	549.1	298	0.04	723.2	730.6	7.4	1.0	37.3	36.5	-0.8	-2.2
1232	1160	N6108a		-	-	295	1.0	739.0	760.1	21.1	2.8	-	-	-	-
1233	1161	N6109a		382.0	550.0	298	1.0	734.9	740.9	6.0	0.8	40.0	39.0	-1.0	-2.4
1234	1162	N6110a		381.1	549.1	298	0.02	735.4	745.5	10.1	1.4	40.2	39.8	-0.4	-1.0
1235	1163	N6111a		390.6	549.1	298	0.02	743.0	758.2	15.2	2.0	-	-	-	-
1236	1164	N6112a		363.1	549.1	298	1.0	737.6	733.1	-4.5	-0.6	34.8	32.8	-2.0	-5.8
1237	1165	N6113a		367.1	549.1	298	0.05	733.9	726.5	-7.4	-1.0	-	-	-	-
1238	1166	N6114a		364.6	549.1	298	0.06	721.5	717.7	-3.7	-0.5	-	-	-	-
1239	1167	N6115a		354.1	549.1	298	0.1	720.0	721.3	1.4	0.2	-	-	-	-
1240	1168	N6116a		361.9	535.1	298	1.01	723.0	719.9	-3.1	-0.4	34.9	34.5	-0.4	-1.1
1241	1169	N6117a		364.6	549.1	298	0.06	718.0	722.7	4.7	0.7	-	-	-	-
1242	1170	N6118a		367.1	549.1	298	0.06	716.0	728.6	12.6	1.8	-	-	-	-
1243	1171	N6201a		394.1	-	298	1.0	770.0	791.3	21.3	2.8	41.4	41.3	-0.1	-0.4
1244	1172	N6202a		407.6	-	298	1.0	738.0	805.7	67.7	9.2	-	-	-	-
1245	1173	N6203a		417.1	-	293	1.0	828.0	838.3	10.3	1.2	-	-	-	-
1246	1174	N6204a		419.1	-	293	1.0	828.0	857.1	29.1	3.5	-	-	-	-

Table S.12 – Comparison of experimental and simulated properties using GM combination rules (continued).

n_{sim}	n_{iso}	Code	Outlier	T_m [K]	T_b [K]	T [K]	P [bar]	$\rho_{\text{liq}}[\text{kg}\cdot\text{m}^{-3}]$ exp	sim	dev	err [%]	$\Delta H_{\text{vap}}[\text{kJ}\cdot\text{mol}^{-1}]$ exp	sim	dev	err [%]
1247	1175	N6205a		466.1	-	298	1.0	-	-	-	-	60.9	61.1	0.2	0.3
1248	1176	N6206a		475.0	-	298	1.0	-	-	-	-	63.1	63.4	0.3	0.5
1249	1177	N7101a		-	-	298	1.0	761.5	777.3	15.8	2.1	-	-	-	-
1250	1178	N7102a		395.1	617.7	298	0.01	768.2	760.4	-7.8	-1.0	-	-	-	-
1251	1179	N7103a		-	-	293	1.0	788.2	776.4	-11.8	-1.5	-	-	-	-
1252	1180	N7104a		405.6	617.7	307	0.01	755.5	764.4	8.9	1.2	-	-	-	-
1253	1181	N7105a		412.6	617.7	312	0.01	752.7	754.8	2.1	0.3	-	-	-	-
1254	1182	N7106a		422.1	617.7	320	0.01	756.1	773.2	17.1	2.3	-	-	-	-
1255	1183	N7107a		415.1	617.7	293	1.0	762.9	772.4	9.5	1.2	-	-	-	-
1256	1184	N7108a		430.1	617.7	298	1.0	771.3	791.0	19.7	2.6	49.9	48.4	-1.5	-2.9
1257	1185	N7109a		-	-	298	1.0	-	-	-	-	42.1	40.9	-1.2	-2.8
1258	1186	N7110a		415.1	577.7	293	1.0	778.7	772.2	-6.5	-0.8	-	-	-	-
1259	1187	N7111a		383.1	577.7	298	0.02	744.5	745.7	1.2	0.2	-	-	-	-
1260	1188	N7112a		385.1	577.7	298	0.02	749.5	727.0	-22.5	-3.0	-	-	-	-
1261	1189	N7113a		380.1	577.7	298	0.03	729.5	732.8	3.3	0.5	-	-	-	-
1262	1190	N7114a		385.1	577.7	298	0.02	738.0	735.8	-2.1	-0.3	-	-	-	-
1263	1191	N7201a		417.1	-	298	1.0	-	-	-	-	45.3	46.1	0.9	1.9
1264	1192	N7202a		441.6	-	293	1.0	-	-	-	-	52.4	51.6	-0.8	-1.5
1265	1193	N7203a		497.1	-	298	1.0	-	-	-	-	67.1	66.9	-0.2	-0.3
1266	1194	N8101a		438.1	643.9	293	1.0	772.0	782.2	10.2	1.3	-	-	-	-
1267	1195	N8102a		452.8	643.9	298	1.01	780.0	798.4	18.5	2.4	55.1	53.1	-2.0	-3.6
1268	1196	N8103a		407.1	603.9	298	1.0	749.0	748.9	-0.1	-0.0	-	-	-	-
1269	1196	N8103b		407.1	603.9	308	0.01	-	-	-	-	46.3	41.5	-4.8	-10.3
1270	1197	N8104a		412.2	603.9	298	1.0	740.7	743.8	3.0	0.4	-	-	-	-
1271	1198	N8105a		432.0	602.3	298	1.0	755.7	761.5	5.8	0.8	49.4	48.1	-1.3	-2.6
1272	1199	N8106a		438.1	603.9	273	1.0	771.2	798.1	26.9	3.5	-	-	-	-
1273	1200	N8107a		399.6	603.9	313	1.0	749.0	731.7	-17.3	-2.3	-	-	-	-
1274	1201	N8108a		409.1	603.9	293	1.0	742.0	753.5	11.5	1.6	-	-	-	-
1275	1202	N8109a		-	-	298	1.0	742.0	752.3	10.3	1.4	-	-	-	-
1276	1203	N8201a		441.1	-	288	1.0	794.2	814.6	20.4	2.6	-	-	-	-
1277	1204	N8202a		457.1	-	288	1.0	848.5	867.6	19.1	2.3	-	-	-	-
1278	1205	N9101a		-	-	293	1.0	771.9	787.0	15.1	2.0	-	-	-	-
1279	1206	N9102a		-	-	298	1.0	789.2	804.3	15.1	1.9	-	-	-	-
1280	1207	N9103a		475.4	668.2	293	1.0	788.6	809.2	20.6	2.6	-	-	-	-
1281	1208	N9104a		453.9	628.2	298	1.0	767.5	774.7	7.1	0.9	-	-	-	-
1282	1209	N9105a		459.9	628.2	293	1.0	782.4	786.7	4.3	0.5	-	-	-	-
1283	1210	N9106a		429.6	628.2	298	1.0	752.9	758.9	6.1	0.8	46.2	48.0	1.8	3.8
1284	1211	N9107a		429.1	628.2	293	1.0	766.3	764.6	-1.7	-0.2	-	-	-	-
1285	1212	N9108a		432.8	628.2	293	1.0	759.3	766.8	7.5	1.0	-	-	-	-
1286	1213	N9109a		445.1	628.2	293	1.0	758.0	772.1	14.1	1.9	-	-	-	-
1287	1214	N9201a		438.9	-	293	1.0	800.0	794.4	-5.6	-0.7	-	-	-	-
1288	1215	N9202a		474.1	-	293	1.0	814.0	826.2	12.2	1.5	-	-	-	-
1289	1216	N9203a		531.6	-	298	1.0	-	-	-	-	75.5	75.1	-0.4	-0.6
1290	1217	N0101a		493.6	690.9	298	1.0	789.0	809.4	20.4	2.6	64.9	62.4	-2.5	-3.8
1291	1218	N0102a		461.1	650.9	298	1.0	766.9	774.3	7.4	1.0	-	-	-	-
1292	1219	N0103a		476.1	650.9	298	1.0	773.2	777.3	4.0	0.5	61.2	57.3	-3.9	-6.3
1293	1220	N0104a		475.1	650.9	298	1.0	774.3	781.2	7.0	0.9	-	-	-	-
1294	1221	N0105a		480.1	650.9	292	1.0	784.0	794.8	10.8	1.4	-	-	-	-

Table S.12 – Comparison of experimental and simulated properties using GM combination rules (continued).

n_{sim}	n_{iso}	Code	Outlier	T_m [K]	T_b [K]	T [K]	P [bar]	ρ_{liq}[kg·m^{-3}] exp	sim	dev	err [%]	ΔH_{vap}[kJ·mol^{-1}] exp	sim	dev	err [%]
1295	1222	N0106a		-	-	293	1.0	768.6	781.6	13.0	1.7	-	-	-	-
1296	1223	N0107a		464.1	650.9	293	1.0	768.7	780.2	11.5	1.5	-	-	-	-
1297	1224	N0201a		485.0	-	345	1.0	-	-	-	-	73.6	72.0	-1.6	-2.2
1298	1225	M2201a		494.3	761.0	354	0.004	1002.0	984.5	-17.4	-1.7	-	-	-	-
1299	1226	M3201a		478.1	718.0	308	1.01	949.7	944.4	-5.3	-0.6	-	-	-	-
1300	1227	M3202a		486.1	-	356	1.01	963.2	928.2	-35.0	-3.6	-	-	-	-
1301	1227	M3202b	×	486.1	-	390	1.0	-	-	-	-	63.9	52.3	-11.6	-18.2
1302	1228	M4201a		439.2	658.0	298	1.01	936.7	942.1	5.4	0.6	50.7	50.7	0.1	0.1
1303	1229	M4202a		421.1	-	298	1.0	930.5	926.5	-4.0	-0.4	66.6	65.0	-1.6	-2.4
1304	1230	M4203a		478.1	-	277	1.0	942.0	959.4	17.4	1.8	-	-	-	-
1305	1230	M4203b		478.1	-	298	1.0	-	-	-	-	64.9	71.1	6.2	9.5
1306	1231	M4204a		489.1	-	393	1.0	885.0	870.7	-14.3	-1.6	-	-	-	-
1307	1232	M5201a		444.6	-	298	1.01	920.3	920.4	0.1	0.0	-	-	-	-
1308	1233	M5202a		-	-	298	1.0	-	-	-	-	67.1	68.3	1.2	1.8
1309	1234	M5203a		-	-	298	1.0	-	-	-	-	66.4	66.4	-0.0	-0.0
1310	1235	M5204a		-	-	298	1.0	-	-	-	-	69.8	75.2	5.4	7.8
1311	1236	M5205a	×	427.0	-	427	1.0	-	-	-	-	36.9	50.2	13.3	36.1
1312	1237	M6201a		459.1	-	298	1.0	906.4	909.8	3.4	0.4	-	-	-	-
1313	1238	M6202a		458.6	700.0	290	1.0	913.0	908.5	-4.5	-0.5	53.7	53.9	0.2	0.3
1314	1239	M6203a		502.1	700.0	298	1.0	896.0	908.0	12.0	1.3	76.1	70.3	-5.8	-7.7
1315	1240	M7201a		464.1	-	293	1.0	897.2	893.4	-3.8	-0.4	-	-	-	-
1316	1241	M7202a		458.8	-	298	1.0	896.2	902.1	5.9	0.7	-	-	-	-
1317	1242	M8201a		482.6	700.0	290	1.0	899.2	895.8	-3.4	-0.4	-	-	-	-
1318	1243	M9201a		484.1	-	293	1.0	876.4	882.6	6.2	0.7	-	-	-	-
1319	1244	S1201a		341.2	557.0	298	1.01	1924.9	1937.2	12.3	0.6	-	-	-	-
1320	1245	S1202a		264.1	411.5	260	1.0	1282.1	1280.5	-1.6	-0.1	21.9	24.7	2.8	12.7
1321	1246	S1301a	×	257.6	412.0	298	1.0	1775.5	1894.7	119.2	6.7	-	-	-	-
1322	1246	S1301b		257.6	412.0	244	1.0	-	-	-	-	24.0	27.0	3.0	12.5
1323	1247	S1302a		232.3	369.3	233	1.0	1408.0	1410.2	2.2	0.2	20.2	21.3	1.1	5.5
1324	1247	S1302b		232.3	369.3	298	10.77	1193.0	1213.4	20.3	1.7	-	-	-	-
1325	1248	S1303a		282.1	451.6	298	1.83	1367.0	1396.0	29.0	2.1	-	-	-	-
1326	1248	S1303b		282.1	451.6	267	1.0	-	-	-	-	26.1	27.5	1.4	5.3
1327	1249	S1304a		373.1	-	295	1.0	3196.9	3204.2	7.3	0.2	-	-	-	-
1328	1250	S1305a		363.1	-	298	1.0	1983.5	2005.4	21.9	1.1	-	-	-	-
1329	1251	S1401a		378.1	606.0	298	1.01	2002.1	2041.9	39.8	2.0	36.1	40.7	4.6	12.7
1330	1252	S1402a	×	215.3	340.1	215	1.0	1989.9	2531.5	541.6	27.2	17.5	23.6	6.1	34.8
1331	1252	S1402b		215.3	340.1	298	16.64	1536.1	1597.8	61.8	4.0	-	-	-	-
1332	1253	S1403a	×	295.9	478.0	298	1.0	2251.2	2361.9	110.6	4.9	25.0	30.4	5.4	21.6
1333	1254	S1404a	×	191.7	301.8	190	1.0	1527.8	1869.1	341.3	22.3	15.4	17.7	2.3	14.9
1334	1254	S1404b	×	191.7	301.8	298	36.66	840.9	1113.2	272.2	32.4	-	-	-	-
1335	1255	S1405a		243.4	384.9	243	1.0	1488.0	1476.0	-12.0	-0.8	20.1	21.0	0.9	4.4
1336	1255	S1405b		243.4	384.9	298	6.74	1307.0	1315.3	8.3	0.6	-	-	-	-
1337	1256	S1406a		297.0	471.2	298	1.06	1477.0	1492.8	15.8	1.1	24.9	26.9	2.0	8.2
1338	1257	S1407a		250.7	-	298	20.0	2046.8	2110.7	63.9	3.1	-	-	-	-
1339	1258	S1408a		269.1	426.1	269	1.0	1899.6	1941.9	42.3	2.2	23.1	25.8	2.7	11.5
1340	1258	S1408b		269.1	426.1	298	2.6	1810.0	1851.8	41.8	2.3	-	-	-	-
1341	1259	S2201a		356.1	-	283	1.0	1667.0	1664.8	-2.2	-0.1	-	-	-	-
1342	1260	S2202a		380.1	-	298	1.01	1727.0	1727.8	0.8	0.0	-	-	-	-

Table S.12 – Comparison of experimental and simulated properties using GM combination rules (continued).

n_{sim}	n_{iso}	Code	Outlier	T_m [K]	T_b [K]	T [K]	P [bar]	ρ_{liq}[kg·m^{-3}] exp	sim	dev	err [%]	ΔH_{vap}[kJ·mol^{-1}] exp	sim	dev	err [%]
1343	1260	S2202b		380.1	-	308	1.0	-	-	-	-	37.6	38.1	0.5	1.3
1344	1261	S2203a		344.6	-	298	1.0	1704.4	1691.5	-12.9	-0.8	-	-	-	-
1345	1262	S2204a		326.1	497.1	298	0.33	1167.5	1144.8	-22.8	-2.0	-	-	-	-
1346	1263	S2205a		413.1	-	288	1.0	2133.6	2132.8	-0.7	-0.0	-	-	-	-
1347	1264	S2206a		401.8	585.0	298	1.01	1200.9	1173.2	-27.7	-2.3	48.3	46.0	-2.3	-4.7
1348	1265	S2207a		423.1	-	293	1.0	1762.9	1743.8	-19.1	-1.1	54.1	53.2	-0.9	-1.7
1349	1266	S2208a		376.6	-	293	1.0	1104.0	1051.9	-52.1	-4.7	-	-	-	-
1350	1266	S2208b		376.6	-	288	1.0	-	-	-	-	44.1	41.3	-2.8	-6.4
1351	1267	S2209a		449.1	-	293	1.0	2196.7	2193.5	-3.2	-0.1	-	-	-	-
1352	1267	S2209b		449.1	-	288	1.0	-	-	-	-	57.0	62.7	5.7	9.9
1353	1268	S2210a	×	-	-	373	1.0	1366.0	1011.9	-354.1	-25.9	-	-	-	-
1354	1269	S2211a		444.1	678.2	298	1.01	1012.1	1056.5	44.4	4.4	59.6	55.7	-3.9	-6.5
1355	1270	S2301a	×	263.1	410.3	265	1.07	1188.8	1064.5	-124.2	-10.5	22.7	18.4	-4.3	-18.9
1356	1270	S2301b	×	263.1	410.3	298	3.61	1107.7	969.4	-138.4	-12.5	19.7	16.9	-2.9	-14.6
1357	1271	S2302a		304.9	478.9	298	1.0	1233.7	1183.6	-50.1	-4.1	26.0	24.7	-1.3	-5.0
1358	1272	S2303a		346.9	523.6	298	1.0	1369.2	1357.3	-11.9	-0.9	-	-	-	-
1359	1273	S2304a	vap	362.0	555.0	298	0.07	1433.0	-	-	-	0.0	-	-	-
1360	1274	S2305a		462.5	686.0	352	0.01	1354.2	1397.5	43.3	3.2	-	-	-	-
1361	1275	S2306a		481.1	-	323	1.0	1933.5	2001.3	67.8	3.5	-	-	-	-
1362	1276	S2307a		441.1	-	309	1.0	1369.3	1357.9	-11.4	-0.8	-	-	-	-
1363	1277	S2308a		419.1	-	298	1.0	1404.0	1404.3	0.3	0.0	-	-	-	-
1364	1278	S2309a	×	-	-	571	1.0	1607.0	1014.6	-592.4	-36.9	-	-	-	-
1365	1279	S2401a		365.6	-	293	1.0	2223.8	2208.9	-14.9	-0.7	-	-	-	-
1366	1280	S2402a		319.8	479.1	298	1.0	1406.6	1366.4	-40.2	-2.9	-	-	-	-
1367	1281	S2403a		-	-	283	1.0	1874.0	1864.1	-9.9	-0.5	-	-	-	-
1368	1282	S2404a	×	279.2	426.2	285	1.0	1353.9	1215.2	-138.7	-10.2	-	-	-	-
1369	1283	S2405a		361.1	535.0	298	1.0	1482.6	1482.3	-0.3	-0.0	-	-	-	-
1370	1284	S2406a		375.1	555.6	298	1.0	1529.8	1529.5	-0.3	-0.0	-	-	-	-
1371	1285	S2407a	×	327.6	-	298	1.0	2130.0	1993.3	-136.7	-6.4	-	-	-	-
1372	1286	S2408a	vap	370.9	565.0	298	0.06	1499.1	-	-	-	0.0	-	-	-
1373	1287	S2409a	vap	447.1	-	298	1.0	2664.9	-	-	-	0.0	-	-	-
1374	1288	S2410a	×	467.1	686.0	356	0.01	1473.5	1559.5	86.0	5.8	-	-	-	-
1375	1289	S2411a		406.1	-	298	1.0	1526.0	1496.4	-29.6	-1.9	-	-	-	-
1376	1290	S2412a	×	347.1	-	298	1.01	1382.4	1164.2	-218.2	-15.8	-	-	-	-
1377	1290	S2412b	×	347.1	-	289	1.0	-	-	-	-	45.9	34.7	-11.2	-24.5
1378	1291	S2501a	×	469.6	688.0	337	1.0	1612.6	1693.8	81.2	5.0	-	-	-	-
1379	1292	S2502a		344.9	491.2	298	1.0	1535.1	1475.9	-59.2	-3.9	35.9	37.8	1.9	5.3
1380	1293	S3201a		368.1	-	293	1.0	1495.0	1478.6	-16.4	-1.1	-	-	-	-
1381	1294	S3202a		390.1	-	293	1.0	1537.0	1557.2	20.2	1.3	-	-	-	-
1382	1295	S3203a		391.1	-	293	1.0	1531.0	1552.3	21.3	1.4	-	-	-	-
1383	1296	S3204a		416.4	-	293	1.0	1596.9	1595.6	-1.3	-0.1	-	-	-	-
1384	1297	S3205a	×	-	515.4	293	1.0	998.2	913.7	-84.5	-8.5	-	-	-	-
1385	1298	S3206a		-	-	293	1.0	1086.0	1041.1	-44.9	-4.1	-	-	-	-
1386	1299	S3207a		444.1	-	293	1.0	1904.0	1926.0	22.0	1.2	-	-	-	-
1387	1300	S3208a	vap	359.1	-	288	1.0	1182.0	-	-	-	0.0	-	-	-
1388	1301	S3209a		392.1	-	293	1.0	1150.0	1151.8	1.8	0.2	-	-	-	-
1389	1302	S3210a		411.1	-	296	1.0	1634.0	1622.5	-11.5	-0.7	-	-	-	-
1390	1303	S3211a		350.1	-	293	1.0	1028.8	1057.0	28.2	2.7	-	-	-	-

Table S.12 – Comparison of experimental and simulated properties using GM combination rules (continued).

n_{sim}	n_{iso}	Code	Outlier	T_m [K]	T_b [K]	T [K]	P [bar]	ρ_{liq}[kg·m^{-3}] exp	sim	dev	err [%]	ΔH_{vap}[kJ·mol^{-1}] exp	sim	dev	err [%]
1391	1304	S3212a	×	-	-	288	1.0	2170.0	2011.3	-158.7	-7.3	-	-	-	-
1392	1305	S3213a		400.1	-	293	1.0	1113.0	1087.8	-25.2	-2.3	-	-	-	-
1393	1306	S3214a		406.6	-	293	1.0	1103.0	1087.0	-16.0	-1.4	-	-	-	-
1394	1307	S3215a		438.1	-	293	1.0	1130.9	1121.6	-9.3	-0.8	-	-	-	-
1395	1308	S3216a		419.6	-	303	1.0	1558.5	1534.3	-24.2	-1.5	-	-	-	-
1396	1309	S3217a		395.3	-	293	1.0	1537.4	1583.9	46.5	3.0	-	-	-	-
1397	1310	S3218a		499.1	-	499	1.0	-	-	-	-	43.8	44.0	0.2	0.5
1398	1311	S3219a		365.6	-	293	1.0	1034.5	1010.8	-23.7	-2.3	-	-	-	-
1399	1312	S3220a		383.1	-	293	1.0	1462.3	1441.6	-20.7	-1.4	-	-	-	-
1400	1313	S3221a	×	418.6	596.0	298	1.0	1074.3	1039.4	-34.9	-3.2	44.2	54.3	10.1	22.9
1401	1314	S3222a		431.1	717.6	293	1.0	937.0	944.4	7.4	0.8	57.8	55.4	-2.4	-4.1
1402	1315	S3223a		432.6	717.6	298	1.01	956.5	962.1	5.5	0.6	-	-	-	-
1403	1316	S3224a		460.6	717.6	298	1.01	987.4	1003.4	15.9	1.6	-	-	-	-
1404	1317	S3225a		365.1	-	298	1.0	1005.0	941.4	-63.6	-6.3	-	-	-	-
1405	1318	S3226a		397.6	564.0	298	1.01	960.2	940.0	-20.3	-2.1	45.2	49.7	4.5	10.0
1406	1319	S3301a		-	-	293	1.0	1708.4	1716.4	8.0	0.5	-	-	-	-
1407	1320	S3302a		469.1	-	287	1.0	2093.0	2102.4	9.4	0.4	-	-	-	-
1408	1321	S3303a		361.8	536.5	298	0.07	1255.2	1224.9	-30.4	-2.4	-	-	-	-
1409	1322	S3304a		458.1	-	293	1.0	1258.5	1308.0	49.5	3.9	-	-	-	-
1410	1323	S3305a		403.0	600.0	293	1.0	1236.0	1206.0	-30.0	-2.4	46.7	44.8	-1.9	-4.0
1411	1324	S3306a		405.1	-	293	1.0	1635.0	1629.8	-5.2	-0.3	-	-	-	-
1412	1325	S3307a		377.6	-	288	1.0	-	-	-	-	42.7	37.8	-4.9	-11.5
1413	1326	S3308a		393.1	-	291	1.0	1304.0	1349.9	45.9	3.5	-	-	-	-
1414	1327	S3309a		446.6	-	319	1.0	1382.6	1385.1	2.5	0.2	-	-	-	-
1415	1328	S3310a		449.1	633.0	298	0.87	1356.5	1343.9	-12.6	-0.9	-	-	-	-
1416	1328	S3310b		449.1	633.0	316	1.0	-	-	-	-	50.4	51.3	0.9	1.7
1417	1329	S3311a		457.1	647.0	293	1.0	1360.7	1347.8	-12.9	-0.9	-	-	-	-
1418	1330	S3312a		492.1	-	293	1.0	2136.4	2115.4	-21.0	-1.0	-	-	-	-
1419	1331	S3313a		492.1	-	293	1.0	2120.0	2124.9	4.9	0.2	-	-	-	-
1420	1332	S3314a		400.1	-	298	1.0	1240.0	1186.3	-53.7	-4.3	-	-	-	-
1421	1333	S3315a		489.1	-	293	1.0	1321.9	1287.4	-34.5	-2.6	-	-	-	-
1422	1334	S3316a		486.1	648.0	298	1.0	1315.9	1287.5	-28.4	-2.2	-	-	-	-
1423	1335	S3317a		-	-	303	1.0	-	-	-	-	31.8	31.1	-0.7	-2.3
1424	1336	S3318a		422.1	-	291	1.0	1167.7	1120.6	-47.1	-4.0	-	-	-	-
1425	1337	S3319a		478.3	691.0	293	1.0	1176.8	1183.7	6.9	0.6	-	-	-	-
1426	1338	S3401a		-	-	293	1.0	2077.2	2116.2	39.0	1.9	-	-	-	-
1427	1339	S3402a	×	460.6	-	285	1.0	1389.0	1497.4	108.4	7.8	-	-	-	-
1428	1340	S3403a		416.1	-	293	1.0	1377.4	1373.7	-3.7	-0.3	47.7	48.6	0.9	1.8
1429	1341	S3404a		358.5	-	288	1.0	-	-	-	-	41.9	33.9	-8.0	-19.1
1430	1342	S3405a		422.1	-	293	1.0	1435.0	1484.7	49.7	3.5	-	-	-	-
1431	1343	S3406a	×	295.1	-	298	1.0	1252.0	1138.5	-113.5	-9.1	-	-	-	-
1432	1344	S3407a	×	348.1	-	298	1.0	-	-	-	-	44.8	34.8	-10.0	-22.3
1433	1345	S3501a		426.9	-	293	1.0	1487.4	1486.0	-1.4	-0.1	48.3	50.0	1.7	3.5
1434	1346	S3502a	×	316.6	-	293	1.0	1280.0	1181.6	-98.4	-7.7	-	-	-	-
1435	1347	S3503a	×	382.6	-	302	1.01	1482.8	1319.4	-163.4	-11.0	53.6	38.4	-15.2	-28.3
1436	1348	S4201a		448.1	-	298	1.01	1485.1	1482.6	-2.5	-0.2	-	-	-	-
1437	1349	S4202a		363.1	-	288	1.0	1053.0	1048.7	-4.3	-0.4	-	-	-	-
1438	1350	S4203a		394.6	-	281	1.0	1106.0	1119.3	13.3	1.2	-	-	-	-

Table S.12 – Comparison of experimental and simulated properties using GM combination rules (continued).

n_{sim}	n_{iso}	Code	Outlier	T_m [K]	T_b [K]	T [K]	P [bar]	ρ_{liq}[kg·m^{-3}] exp	sim	dev	err [%]	ΔH_{vap}[kJ·mol^{-1}] exp	sim	dev	err [%]
1439	1351	S4204a		388.1	-	298	1.0	1055.4	1064.7	9.3	0.9	-	-	-	-
1440	1352	S4205a		414.1	-	298	1.0	1068.0	1051.5	-16.5	-1.5	-	-	-	-
1441	1353	S4206a		388.3	-	293	1.0	1088.3	1081.8	-6.5	-0.6	-	-	-	-
1442	1354	S4207a		380.6	-	293	1.0	989.5	961.1	-28.4	-2.9	-	-	-	-
1443	1355	S4208a		400.6	-	273	1.0	1385.2	1350.7	-34.5	-2.5	-	-	-	-
1444	1356	S4209a	×	408.1	620.0	298	1.0	1096.9	982.4	-114.5	-10.4	-	-	-	-
1445	1357	S4210a		418.6	-	298	1.0	-	-	-	-	48.7	55.2	6.5	13.4
1446	1358	S4211a		433.1	-	293	1.0	1027.2	1002.6	-24.6	-2.4	-	-	-	-
1447	1359	S4212a		455.1	-	293	1.0	1023.3	1013.2	-10.1	-1.0	-	-	-	-
1448	1360	S4213a	×	407.1	-	298	1.01	883.8	878.8	-5.1	-0.6	46.7	57.5	10.8	23.1
1449	1361	S4214a		438.6	-	298	1.01	932.6	913.6	-19.0	-2.0	-	-	-	-
1450	1361	S4214b	×	438.6	-	308	1.0	-	-	-	-	63.3	52.2	-11.1	-17.5
1451	1362	S4215a		442.6	-	293	1.0	914.0	918.4	4.4	0.5	61.0	58.9	-2.1	-3.5
1452	1363	S4216a		478.1	-	285	1.0	967.0	992.9	25.9	2.7	-	-	-	-
1453	1364	S4217a		389.1	-	298	1.0	957.0	932.0	-25.0	-2.6	-	-	-	-
1454	1365	S4218a		403.1	588.8	293	1.0	938.0	912.9	-25.1	-2.7	-	-	-	-
1455	1366	S4219a		392.1	588.8	298	1.01	915.9	900.1	-15.8	-1.7	46.2	48.3	2.1	4.6
1456	1367	S4220a		408.1	588.8	298	1.0	925.4	892.6	-32.8	-3.5	48.2	50.2	2.0	4.1
1457	1368	S4301a		-	-	293	1.0	1598.5	1620.9	22.4	1.4	-	-	-	-
1458	1369	S4302a		406.8	-	293	1.0	1179.6	1236.7	57.1	4.8	-	-	-	-
1459	1370	S4303a		406.8	-	293	1.0	1223.6	1271.5	47.9	3.9	-	-	-	-
1460	1371	S4304a		472.1	-	333	1.0	1496.9	1569.9	73.0	4.9	-	-	-	-
1461	1372	S4305a		405.6	-	298	1.0	1075.0	1113.2	38.2	3.6	-	-	-	-
1462	1373	S4306a		417.4	618.0	293	1.0	1158.5	1131.3	-27.2	-2.3	49.5	47.4	-2.1	-4.3
1463	1374	S4307a		418.1	-	293	1.0	1178.0	1134.7	-43.3	-3.7	-	-	-	-
1464	1375	S4308a		441.6	-	293	1.0	1503.2	1491.1	-12.1	-0.8	-	-	-	-
1465	1376	S4309a		435.6	-	293	1.0	1514.0	1497.2	-16.8	-1.1	-	-	-	-
1466	1377	S4310a	×	-	-	291	1.0	1412.3	1515.2	102.9	7.3	-	-	-	-
1467	1378	S4311a		393.1	-	293	1.0	1091.2	1057.0	-34.2	-3.1	-	-	-	-
1468	1378	S4311b		393.1	-	288	1.0	-	-	-	-	41.9	40.7	-1.2	-2.9
1469	1379	S4312a		452.1	-	286	1.0	1817.3	1804.7	-12.6	-0.7	-	-	-	-
1470	1380	S4313a	vap	451.6	666.0	333	0.01	1176.5	-	-	-	0.0	-	-	-
1471	1381	S4314a	vap	458.1	-	293	1.0	1845.2	-	-	-	0.0	-	-	-
1472	1382	S4315a		422.1	-	283	1.0	1027.5	1028.3	0.8	0.1	-	-	-	-
1473	1383	S4316a	×	417.6	584.0	298	1.0	-	-	-	-	44.3	55.1	10.9	24.5
1474	1384	S4317a		433.1	-	296	1.0	1082.6	1053.5	-29.1	-2.7	-	-	-	-
1475	1385	S4318a		461.1	-	288	1.0	1108.0	1073.6	-34.4	-3.1	-	-	-	-
1476	1386	S4319a		452.1	-	336	1.0	-	-	-	-	60.0	57.0	-3.0	-5.1
1477	1387	S4320a		485.1	664.0	356	1.0	1046.1	1109.8	63.7	6.1	-	-	-	-
1478	1388	S4321a		479.6	-	293	1.0	1102.1	1096.6	-5.5	-0.5	-	-	-	-
1479	1389	S4322a		404.1	-	293	1.0	1051.1	998.8	-52.3	-5.0	-	-	-	-
1480	1390	S4401a		437.1	-	293	1.0	1395.6	1445.2	49.6	3.6	-	-	-	-
1481	1391	S4402a		-	-	293	1.0	1328.2	1325.9	-2.3	-0.2	-	-	-	-
1482	1392	S4403a		428.1	-	293	1.0	1282.7	1283.4	0.7	0.1	50.6	51.5	0.9	1.8
1483	1393	S4404a		479.1	-	293	1.0	1933.3	1959.3	26.0	1.3	-	-	-	-
1484	1394	S4405a		467.1	-	293	1.0	1899.1	1915.6	16.5	0.9	-	-	-	-
1485	1395	S4406a		373.1	-	293	1.0	1176.5	1136.0	-40.5	-3.4	-	-	-	-
1486	1396	S4407a		-	-	288	1.0	-	-	-	-	55.1	48.8	-6.3	-11.4

Table S.12 – Comparison of experimental and simulated properties using GM combination rules (continued).

n_{sim}	n_{iso}	Code	Outlier	T_m [K]	T_b [K]	T [K]	P [bar]	ρ_{liq}[kg·m^{-3}] exp	sim	dev	err [%]	ΔH_{vap}[kJ·mol^{-1}] exp	sim	dev	err [%]
1487	1397	S4408a		402.1	-	293	1.0	1225.0	1214.3	-10.7	-0.9	-	-	-	-
1488	1398	S4409a		393.1	-	288	1.0	-	-	-	-	56.4	54.5	-1.9	-3.4
1489	1399	S4501a		440.6	-	293	1.0	1383.6	1389.7	6.1	0.4	51.0	52.9	1.9	3.7
1490	1400	S4502a		334.1	-	293	1.0	1194.0	1126.1	-67.9	-5.7	-	-	-	-
1491	1401	S5201a		396.5	-	293	1.0	1052.3	1065.9	13.6	1.3	-	-	-	-
1492	1402	S5202a		392.6	-	298	1.0	-	-	-	-	43.6	42.3	-1.3	-3.1
1493	1403	S5203a		413.6	-	293	1.0	952.6	948.1	-4.5	-0.5	-	-	-	-
1494	1404	S5204a		482.1	-	293	1.0	1007.1	992.4	-14.7	-1.5	-	-	-	-
1495	1405	S5205a		423.4	-	299	1.0	882.0	876.5	-5.5	-0.6	-	-	-	-
1496	1406	S5206a		397.6	-	298	1.0	837.0	864.2	27.2	3.2	-	-	-	-
1497	1407	S5207a		436.6	-	298	1.01	880.8	876.8	-4.1	-0.5	57.0	62.0	5.0	8.8
1498	1408	S5208a		446.1	-	298	1.0	893.2	886.5	-6.8	-0.8	-	-	-	-
1499	1409	S5209a		455.1	-	293	1.0	900.5	903.2	2.7	0.3	-	-	-	-
1500	1410	S5210a		408.4	-	293	1.0	916.5	901.8	-14.7	-1.6	-	-	-	-
1501	1411	S5211a		430.1	614.5	296	1.0	923.0	908.5	-14.5	-1.6	-	-	-	-
1502	1412	S5212a		418.1	614.5	298	1.01	899.7	892.4	-7.3	-0.8	50.1	54.8	4.7	9.4
1503	1413	S5213a		404.1	614.5	298	1.01	896.6	865.4	-31.3	-3.5	-	-	-	-
1504	1414	S5214a		424.5	615.2	298	1.01	907.9	886.1	-21.8	-2.4	52.1	54.4	2.3	4.5
1505	1415	S5301a		-	-	293	1.0	1108.5	1057.8	-50.7	-4.6	-	-	-	-
1506	1416	S5302a	×	503.1	-	298	1.0	1341.6	1210.8	-130.8	-9.7	-	-	-	-
1507	1417	S5303a		423.6	-	293	1.0	1088.8	1078.4	-10.4	-1.0	-	-	-	-
1508	1418	S5304a		420.1	-	293	1.0	1079.3	1068.0	-11.3	-1.0	-	-	-	-
1509	1419	S5305a		434.1	-	293	1.0	1104.0	1092.5	-11.5	-1.0	-	-	-	-
1510	1420	S5306a		435.1	-	293	1.0	1108.6	1092.7	-15.9	-1.4	-	-	-	-
1511	1421	S5307a		447.1	-	293	1.0	1129.3	1111.0	-18.3	-1.6	-	-	-	-
1512	1422	S5308a		441.1	-	293	1.0	1452.8	1407.3	-45.5	-3.1	-	-	-	-
1513	1423	S5309a		433.1	-	293	1.0	1413.5	1381.8	-31.7	-2.2	-	-	-	-
1514	1424	S5310a		452.1	-	291	1.0	1412.3	1410.8	-1.5	-0.1	-	-	-	-
1515	1425	S5311a		459.6	-	298	1.0	1400.0	1423.8	23.8	1.7	-	-	-	-
1516	1426	S5312a		-	-	288	1.0	-	-	-	-	44.3	42.9	-1.4	-3.1
1517	1427	S5313a		-	-	288	1.0	-	-	-	-	47.3	47.9	0.6	1.3
1518	1428	S5314a		-	-	293	1.0	-	-	-	-	43.9	44.9	1.0	2.3
1519	1429	S5315a		519.0	738.0	308	1.0	1120.1	1152.1	31.9	2.9	-	-	-	-
1520	1430	S5316a		444.1	-	293	1.0	1075.7	1057.8	-17.9	-1.7	-	-	-	-
1521	1431	S5317a		428.1	-	288	1.0	1059.6	1054.7	-4.9	-0.5	-	-	-	-
1522	1432	S5318a		444.9	642.0	333	0.01	1035.7	1029.4	-6.2	-0.6	45.6	51.1	5.5	12.1
1523	1433	S5319a	×	426.0	588.0	293	1.0	938.4	1089.9	151.5	16.1	-	-	-	-
1524	1434	S5320a		415.6	-	288	1.0	1013.9	982.1	-31.8	-3.1	-	-	-	-
1525	1435	S5321a		416.1	-	292	1.0	1007.4	970.1	-37.3	-3.7	-	-	-	-
1526	1435	S5321b		416.1	-	298	1.0	-	-	-	-	50.3	47.4	-2.9	-5.8
1527	1436	S5401a		456.6	-	293	1.0	1240.1	1250.4	10.3	0.8	-	-	-	-
1528	1437	S5402a		487.6	-	293	1.0	1796.6	1810.2	13.6	0.8	-	-	-	-
1529	1438	S5403a		-	-	288	1.0	-	-	-	-	54.5	54.4	-0.1	-0.2
1530	1439	S5501a		448.1	-	298	1.0	1291.1	1307.5	16.4	1.3	-	-	-	-
1531	1440	S5502a		460.1	-	293	1.0	1322.1	1328.4	6.3	0.5	-	-	-	-
1532	1441	S6201a	×	-	-	293	1.0	1250.0	1454.7	204.7	16.4	-	-	-	-
1533	1442	S6202a		431.3	-	293	1.0	1024.1	1028.2	4.1	0.4	-	-	-	-
1534	1443	S6203a		441.0	606.0	293	1.0	938.7	939.8	1.1	0.1	-	-	-	-

Table S.12 – Comparison of experimental and simulated properties using GM combination rules (continued).

n_{sim}	n_{iso}	Code	Outlier	T_m [K]	T_b [K]	T [K]	P [bar]	ρ_{liq}[kg·m^{-3}] exp	sim	dev	err [%]	ΔH_{vap}[kJ·mol^{-1}] exp	sim	dev	err [%]
1535	1444	S6204a	×	436.1	-	298	1.01	880.4	865.9	-14.4	-1.6	52.5	64.9	12.4	23.6
1536	1445	S6205a		472.1	-	293	1.0	890.7	896.4	5.7	0.6	-	-	-	-
1537	1446	S6206a		410.6	637.8	293	1.0	879.0	868.9	-10.1	-1.1	-	-	-	-
1538	1447	S6207a		423.1	637.8	298	1.01	880.8	862.8	-18.1	-2.0	-	-	-	-
1539	1448	S6208a		433.1	637.8	293	1.0	890.0	879.8	-10.2	-1.1	-	-	-	-
1540	1449	S6209a		444.5	633.9	298	1.01	896.2	882.7	-13.4	-1.5	56.6	59.1	2.5	4.4
1541	1450	S6301a		424.6	-	293	1.0	1031.5	1027.6	-3.9	-0.4	-	-	-	-
1542	1451	S6302a		453.1	-	293	1.0	1065.6	1063.0	-2.6	-0.2	-	-	-	-
1543	1452	S6303a		456.1	-	293	1.0	1070.4	1066.0	-4.4	-0.4	-	-	-	-
1544	1453	S6304a		457.1	-	293	1.0	1075.6	1066.3	-9.3	-0.9	-	-	-	-
1545	1454	S6305a		436.1	-	293	1.0	1326.3	1312.9	-13.4	-1.0	-	-	-	-
1546	1455	S6306a		450.1	-	293	1.0	1327.3	1328.5	1.2	0.1	-	-	-	-
1547	1456	S6307a		465.1	-	293	1.0	1354.0	1347.7	-6.3	-0.5	-	-	-	-
1548	1457	S6308a		-	-	288	1.0	-	-	-	-	54.9	51.5	-3.4	-6.3
1549	1458	S6309a		-	-	296	1.0	1086.1	1095.4	9.3	0.9	-	-	-	-
1550	1459	S6310a	vap	460.1	-	293	1.0	1103.0	-	-	-	0.0	-	-	-
1551	1460	S6311a		489.1	-	293	1.0	1136.0	1115.7	-20.3	-1.8	-	-	-	-
1552	1461	S6312a		440.1	-	293	1.0	998.0	970.7	-27.3	-2.7	-	-	-	-
1553	1462	S6313a	×	450.6	-	298	1.0	-	-	-	-	55.9	64.9	9.0	16.1
1554	1463	S6314a		547.6	-	298	1.0	1090.0	1122.7	32.7	3.0	-	-	-	-
1555	1464	S6315a		450.6	-	298	1.0	1021.7	1027.8	6.1	0.6	-	-	-	-
1556	1465	S6316a		453.9	643.0	283	1.0	1036.8	1032.8	-4.0	-0.4	-	-	-	-
1557	1466	S6317a		469.1	-	293	1.0	1051.1	1040.9	-10.2	-1.0	-	-	-	-
1558	1467	S6318a		431.1	-	293	1.0	970.2	935.9	-34.3	-3.5	-	-	-	-
1559	1468	S6319a		429.4	607.3	293	1.0	974.0	936.7	-37.3	-3.8	52.7	48.8	-3.9	-7.4
1560	1469	S6401a		466.6	-	293	1.0	1182.0	1189.3	7.3	0.6	-	-	-	-
1561	1470	S6402a		495.6	-	293	1.0	1698.7	1721.2	22.5	1.3	-	-	-	-
1562	1471	S6403a		482.9	-	293	1.0	1146.6	1153.1	6.5	0.6	-	-	-	-
1563	1472	S6404a		482.9	-	298	1.0	1169.0	1165.7	-3.3	-0.3	-	-	-	-
1564	1473	S6501a		461.1	-	293	1.0	1263.6	1271.6	8.0	0.6	-	-	-	-
1565	1474	S6502a		477.1	-	293	1.0	1277.8	1283.9	6.1	0.5	-	-	-	-
1566	1475	S6503a		375.1	-	295	1.0	1026.8	1075.6	48.8	4.7	-	-	-	-
1567	1476	S7201a		430.1	-	298	1.0	-	-	-	-	47.7	49.0	1.3	2.7
1568	1477	S7202a		462.6	-	293	1.0	860.0	868.9	8.9	1.0	-	-	-	-
1569	1478	S7203a		433.1	-	298	1.0	898.0	898.7	0.7	0.1	-	-	-	-
1570	1479	S7204a		420.2	659.3	320	0.01	-	-	-	-	47.7	48.9	1.3	2.6
1571	1480	S7205a		444.6	659.3	298	1.0	874.6	861.7	-12.9	-1.5	-	-	-	-
1572	1481	S7301a		456.1	-	293	1.0	1025.2	1024.9	-0.3	-0.0	-	-	-	-
1573	1482	S7302a		457.1	-	293	1.0	1025.3	1023.0	-2.3	-0.2	-	-	-	-
1574	1483	S7303a		464.4	-	293	1.0	1032.3	1033.8	1.5	0.1	-	-	-	-
1575	1484	S7304a		449.4	-	293	1.0	1037.0	1042.0	5.0	0.5	-	-	-	-
1576	1485	S7305a		459.1	-	293	1.0	1276.0	1284.0	8.0	0.6	-	-	-	-
1577	1486	S7306a		464.1	-	291	1.0	1226.0	1284.3	58.3	4.8	-	-	-	-
1578	1487	S7307a		499.9	-	293	1.0	1308.5	1299.8	-8.7	-0.7	-	-	-	-
1579	1488	S7308a		-	-	288	1.0	-	-	-	-	54.6	58.8	4.2	7.8
1580	1489	S7309a		-	-	288	1.0	-	-	-	-	56.8	63.1	6.3	11.0
1581	1490	S7310a		455.1	-	287	1.0	995.0	1019.8	24.8	2.5	-	-	-	-
1582	1491	S7311a		459.1	-	293	1.0	983.5	991.8	8.3	0.8	-	-	-	-

Table S.12 – Comparison of experimental and simulated properties using GM combination rules (continued).

n_{sim}	n_{iso}	Code	Outlier	T_m [K]	T_b [K]	T [K]	P [bar]	ρ_{liq}[kg·m^{-3}] exp	sim	dev	err [%]	ΔH_{vap}[kJ·mol^{-1}] exp	sim	dev	err [%]
1583	1492	S7312a		460.1	-	293	1.0	994.1	998.8	4.7	0.5	-	-	-	-
1584	1493	S7313a		464.1	-	293	1.0	1012.0	999.9	-12.1	-1.2	-	-	-	-
1585	1494	S7314a		478.9	-	293	1.0	1011.1	1007.2	-3.9	-0.4	-	-	-	-
1586	1495	S7315a		438.1	621.0	293	1.0	949.0	922.3	-26.7	-2.8	-	-	-	-
1587	1496	S7316a		442.2	-	298	1.0	-	-	-	-	55.6	52.4	-3.2	-5.8
1588	1497	S7401a		464.2	-	293	1.0	1062.3	1136.6	74.3	7.0	-	-	-	-
1589	1498	S7501a		490.1	-	293	1.0	1231.4	1245.9	14.5	1.2	-	-	-	-
1590	1499	S8201a		458.1	-	289	1.0	910.7	928.5	17.8	2.0	-	-	-	-
1591	1500	S8202a		463.1	-	298	1.0	826.0	858.8	32.8	4.0	-	-	-	-
1592	1501	S8203a		481.1	679.3	293	1.0	887.8	883.1	-4.7	-0.5	-	-	-	-
1593	1502	S8301a		475.6	-	298	1.0	958.9	953.9	-5.0	-0.5	-	-	-	-
1594	1503	S8302a	×	-	-	298	1.0	-	-	-	-	61.9	73.8	11.9	19.2
1595	1504	S8303a		460.1	-	293	1.0	975.6	969.9	-5.7	-0.6	-	-	-	-
1596	1505	S8304a		471.1	-	289	1.0	984.7	988.5	3.8	0.4	-	-	-	-
1597	1506	S8305a		446.1	-	298	1.0	980.0	973.3	-6.7	-0.7	-	-	-	-
1598	1507	S8306a		469.0	-	298	1.0	967.1	984.2	17.1	1.8	-	-	-	-
1599	1508	S8307a		494.6	-	298	1.0	989.0	988.4	-0.6	-0.1	-	-	-	-
1600	1509	S8308a		465.7	641.2	298	1.0	-	-	-	-	59.5	57.0	-2.5	-4.2
1601	1510	S8401a		495.1	-	293	1.0	1073.2	1090.8	17.6	1.6	-	-	-	-
1602	1511	S9301a		-	-	291	1.0	970.0	976.1	6.1	0.6	-	-	-	-
1603	1512	S9302a		474.1	-	291	1.0	964.8	978.1	13.3	1.4	-	-	-	-
1604	1513	S9303a		497.1	-	293	1.0	966.1	973.9	7.8	0.8	-	-	-	-
1605	1514	S9304a		510.6	-	293	1.0	973.5	978.1	4.6	0.5	-	-	-	-
1606	1515	S0201a		473.7	-	293	1.0	922.0	929.1	7.1	0.8	75.3	78.8	3.5	4.6
1607	1516	S0301a		-	-	297	1.0	1191.2	1254.5	63.3	5.3	-	-	-	-

Table S.13: Experimental and simulated properties of the 1607 compounds/P, T-points considered in the simulations using LB combination rules.

n_{sim}	n_{iso}	Code	Outlier	T_m [K]	T_b [K]	T [K]	P [bar]	$\rho_{\text{liq}}[\text{kg}\cdot\text{m}^{-3}]$ exp	sim	dev	err [%]	$\Delta H_{\text{vap}}[\text{kJ}\cdot\text{mol}^{-1}]$ exp	sim	dev	err [%]
1	1	A1001a		111.7	190.6	91	0.12	451.0	409.4	-41.6	-9.2	9.2	8.1	-1.1	-12.1
2	1	A1001b		111.7	190.6	102	0.41	436.1	393.3	-42.8	-9.8	8.8	7.8	-1.0	-11.4
3	1	A1001c		111.7	190.6	112	1.01	422.0	378.2	-43.8	-10.4	-	-	-	-
4	1	A1001d		111.7	190.6	112	1.01	-	-	-	-	8.5	7.6	-0.9	-10.3
5	2	A2001a		184.6	305.3	189	1.26	526.6	510.8	-15.8	-3.0	-	-	-	-
6	2	A2001b		184.6	305.3	266	20.88	409.4	382.1	-27.3	-6.7	9.7	10.8	1.1	11.3
7	2	A2001c		184.6	305.3	185	1.01	-	-	-	-	14.8	13.7	-1.0	-7.1
8	3	A3001a		231.1	369.8	230	1.0	582.1	566.5	-15.6	-2.7	18.8	17.9	-0.9	-4.5
9	3	A3001b		231.1	369.8	298	9.78	493.1	478.2	-14.9	-3.0	-	-	-	-
10	4	A4001a		261.4	407.8	260	19.85	597.9	592.5	-5.4	-0.9	-	-	-	-
11	4	A4001b		261.4	407.8	300	25.33	553.0	549.4	-3.6	-0.6	-	-	-	-
12	4	A4001c		261.4	407.8	265	1.0	-	-	-	-	22.4	20.7	-1.7	-7.6
13	5	A4002a		272.6	425.1	273	1.0	601.0	593.2	-7.8	-1.3	-	-	-	-
14	5	A4002b		272.6	425.1	300	9.93	572.5	565.3	-7.2	-1.3	-	-	-	-
15	5	A4002c		272.6	425.1	264	1.0	-	-	-	-	23.1	22.1	-1.0	-4.1
16	6	A5001a		282.6	433.8	282	0.99	603.7	612.4	8.7	1.4	23.6	23.0	-0.7	-2.8
17	6	A5001b		282.6	433.8	298	1.76	586.0	597.1	11.2	1.9	-	-	-	-
18	7	A5002a		301.0	460.4	298	0.92	616.0	613.3	-2.7	-0.4	25.5	24.7	-0.7	-2.9
19	8	A5003a		309.2	469.7	298	1.01	621.1	620.1	-1.0	-0.2	26.6	26.0	-0.6	-2.4
20	9	A6001a		322.9	489.0	298	1.01	644.4	655.2	10.8	1.7	28.8	27.7	-1.1	-3.8
21	10	A6002a		331.1	500.0	298	1.01	658.6	658.1	-0.4	-0.1	30.0	28.9	-1.2	-3.8
22	11	A6003a		336.4	504.4	298	1.01	659.9	661.0	1.1	0.2	30.9	29.9	-1.0	-3.1
23	12	A6004a		333.4	497.7	298	1.01	648.5	652.0	3.6	0.6	30.4	29.8	-0.7	-2.2
24	13	A6005a		341.9	507.6	298	1.0	656.1	657.5	1.4	0.2	31.8	31.0	-0.8	-2.5
25	14	A7001a		354.0	531.2	298	0.13	687.1	699.5	12.4	1.8	32.4	32.2	-0.2	-0.6
26	15	A7002a		359.2	536.4	298	0.11	686.9	700.7	13.8	2.0	34.3	33.0	-1.4	-4.0
27	16	A7003a		352.3	520.5	298	0.14	673.1	684.0	10.9	1.6	33.4	32.7	-0.7	-2.2
28	17	A7004a		362.9	537.3	298	0.09	691.1	694.6	3.5	0.5	35.0	33.9	-1.0	-2.9
29	18	A7005a		353.6	519.8	298	0.13	667.9	677.8	9.9	1.5	33.6	33.4	-0.2	-0.7
30	19	A7006a		366.6	527.0	298	0.08	695.1	694.6	-0.5	-0.1	35.2	34.7	-0.5	-1.3
31	20	A7007a		365.0	535.2	298	0.08	683.9	687.5	3.6	0.5	35.4	34.8	-0.6	-1.8
32	21	A7008a		363.2	530.4	298	0.09	674.0	681.4	7.5	1.1	35.2	34.6	-0.6	-1.7
33	22	A7009a		371.6	540.2	298	0.06	682.0	684.7	2.7	0.4	36.5	35.8	-0.7	-1.9
34	23	A8001a		387.9	573.5	298	0.04	722.1	735.9	13.8	1.9	37.6	37.4	-0.2	-0.5
35	24	A8002a		383.0	563.5	298	0.04	712.0	728.1	16.1	2.3	-	-	-	-
36	25	A8003a		372.4	543.8	298	1.01	688.0	703.8	15.7	2.3	35.2	36.0	0.8	2.2
37	26	A8004a		386.6	566.4	298	0.04	715.9	723.7	7.7	1.1	37.7	38.0	0.3	0.8
38	27	A8005a		391.4	576.5	298	0.03	724.0	735.9	11.9	1.7	38.0	38.2	0.2	0.5
39	28	A8006a		385.1	562.0	298	0.04	707.1	721.5	14.4	2.0	37.5	38.0	0.5	1.2
40	29	A8007a		380.0	549.8	298	0.05	692.1	707.4	15.3	2.2	37.3	37.4	0.1	0.3
41	30	A8008a		388.8	567.0	298	0.03	711.1	720.3	9.3	1.3	38.5	39.0	0.5	1.3
42	31	A8009a		390.9	568.8	298	0.03	715.9	721.8	5.9	0.8	39.0	39.0	-0.0	-0.1
43	32	A8010a		388.8	563.5	298	0.03	708.1	714.7	6.6	0.9	38.8	38.8	-0.0	-0.1
44	33	A8011a		382.6	553.5	298	1.0	696.4	706.7	10.2	1.5	37.8	38.2	0.4	1.1
45	34	A8012a		382.3	550.0	298	0.04	690.0	700.3	10.3	1.5	37.9	38.1	0.2	0.4
46	35	A8013a		391.7	565.5	298	0.03	710.1	714.4	4.2	0.6	39.7	39.6	-0.2	-0.4

Table S.13 – Comparison of experimental and simulated properties using LB combination rules (continued).

n_{sim}	n_{iso}	Code	Outlier	T_m	T_b	T	P	ρ_{liq}[kg·m^{-3}]				ΔH_{vap}[kJ·mol^{-1}]			
				[K]	[K]	[K]	[bar]	exp	sim	dev	err [%]	exp	sim	dev	err [%]
47	36	A8014a		390.9	561.7	298	0.03	713.0	708.4	-4.7	-0.7	39.7	39.7	0.0	0.1
48	37	A8015a		392.1	563.7	298	0.03	702.0	709.0	7.0	1.0	39.9	39.5	-0.3	-0.8
49	38	A8016a		390.8	559.6	298	0.03	696.0	702.5	6.4	0.9	39.7	39.4	-0.3	-0.8
50	39	A8017a		398.8	568.7	298	1.01	698.9	706.3	7.5	1.1	41.0	40.8	-0.3	-0.7
51	40	A9001a		413.4	607.5	298	1.0	753.0	773.1	20.1	2.7	41.2	41.4	0.2	0.5
52	41	A9002a		395.4	574.6	298	0.03	716.1	727.3	11.2	1.6	38.5	38.7	0.2	0.6
53	42	A9003a		414.7	607.5	301	0.01	733.0	764.2	31.2	4.3	-	-	-	-
54	42	A9003b		414.7	607.5	298	1.0	-	-	-	-	41.8	42.0	0.2	0.5
55	43	A9004a		406.2	592.6	298	0.02	735.1	748.2	13.0	1.8	40.8	40.7	-0.1	-0.1
56	44	A9005a		417.9	582.1	298	1.0	750.8	765.1	14.3	1.9	42.7	42.4	-0.4	-0.8
57	45	A9006a		413.6	582.1	298	1.0	741.4	758.2	16.8	2.3	42.2	42.4	0.2	0.6
58	46	A9007a		410.8	582.1	298	1.0	734.5	751.0	16.5	2.3	42.1	42.3	0.2	0.4
59	47	A9008a		407.0	582.1	298	0.02	731.1	744.9	13.8	1.9	41.7	41.3	-0.4	-0.8
60	48	A9009a		406.8	582.1	298	0.02	725.1	743.7	18.6	2.6	41.7	42.0	0.3	0.8
61	49	A9010a		403.8	582.1	298	0.02	720.1	734.8	14.8	2.0	41.1	41.0	-0.1	-0.3
62	50	A9011a		399.7	582.1	298	0.02	713.1	728.2	15.2	2.1	40.7	40.6	-0.1	-0.2
63	51	A9012a		397.2	569.8	298	0.02	707.1	721.5	14.4	2.0	40.2	40.6	0.4	1.0
64	52	A9013a		409.9	582.1	298	0.01	733.9	739.6	5.8	0.8	42.3	41.9	-0.4	-0.8
65	53	A9014a		412.2	582.1	298	1.0	735.4	744.9	9.5	1.3	42.7	42.9	0.2	0.6
66	54	A9015a		404.5	582.1	298	0.02	718.1	728.7	10.6	1.5	41.4	41.8	0.4	1.0
67	55	A9016a		419.3	610.0	308	0.03	727.9	755.3	27.5	3.8	-	-	-	-
68	55	A9016b		419.3	610.0	298	1.0	-	-	-	-	42.6	43.0	0.4	0.9
69	56	A9017a		413.8	582.1	298	1.0	737.1	750.5	13.4	1.8	42.9	43.0	0.1	0.2
70	57	A9018a		408.4	582.1	298	0.01	721.0	737.3	16.3	2.3	42.2	42.8	0.6	1.5
71	58	A9019a		410.4	582.1	306	0.01	715.9	731.7	15.8	2.2	-	-	-	-
72	58	A9019b		410.4	582.1	298	1.0	-	-	-	-	42.6	42.5	-0.1	-0.1
73	59	A9020a		405.8	576.7	298	0.02	707.1	723.7	16.5	2.3	42.3	42.1	-0.2	-0.5
74	60	A9021a		413.6	582.1	303	0.01	732.6	738.3	5.7	0.8	-	-	-	-
75	60	A9021b		413.6	582.1	298	1.0	-	-	-	-	43.6	43.9	0.3	0.6
76	61	A9022a		411.2	582.1	300	0.01	727.4	733.9	6.5	0.9	-	-	-	-
77	61	A9022b		411.2	582.1	298	1.0	-	-	-	-	43.2	43.4	0.2	0.6
78	62	A9023a		413.8	582.1	302	0.01	723.9	734.9	11.0	1.5	-	-	-	-
79	62	A9023b		413.8	582.1	298	1.0	-	-	-	-	43.6	43.8	0.2	0.4
80	63	A9024a		413.7	582.1	303	0.01	717.9	727.2	9.3	1.3	-	-	-	-
81	63	A9024b		413.7	582.1	298	1.0	-	-	-	-	43.6	43.3	-0.3	-0.6
82	64	A9025a		407.0	582.1	298	0.01	719.0	727.7	8.6	1.2	42.9	42.5	-0.4	-1.0
83	65	A9026a		409.2	582.1	300	0.01	717.9	728.3	10.4	1.5	43.3	42.8	-0.5	-1.2
84	66	A9027a		406.1	582.1	298	0.01	711.1	723.3	12.3	1.7	42.9	43.1	0.2	0.4
85	67	A9028a		409.2	582.1	300	0.01	711.8	721.3	9.6	1.3	43.3	42.7	-0.6	-1.4
86	68	A9029a		408.4	582.1	298	0.01	705.9	716.2	10.3	1.5	43.3	42.8	-0.5	-1.2
87	69	A9030a		414.4	582.1	298	1.0	724.1	730.1	6.0	0.8	44.1	44.3	0.2	0.4
88	70	A9031a		416.4	582.1	298	1.0	722.5	730.7	8.2	1.1	44.5	44.2	-0.3	-0.8
89	71	A9032a		415.6	582.1	298	1.0	716.0	725.6	9.6	1.3	44.5	44.4	-0.1	-0.2
90	72	A9033a		417.4	582.1	298	1.0	717.0	725.7	8.7	1.2	44.9	44.3	-0.6	-1.3
91	73	A9034a		416.4	582.8	298	1.0	709.5	720.4	10.9	1.5	-	-	-	-
92	73	A9034b		416.4	582.8	305	0.01	-	-	-	-	43.8	43.9	0.1	0.2
93	74	A9035a		424.0	594.6	298	1.01	713.7	723.4	9.7	1.4	46.4	45.7	-0.7	-1.6
94	75	A0001a		439.2	606.6	298	1.0	776.7	799.6	22.9	2.9	45.2	45.8	0.6	1.4

Table S.13 – Comparison of experimental and simulated properties using LB combination rules (continued).

n_{sim}	n_{iso}	Code	Outlier	T_m [K]	T_b [K]	T [K]	P [bar]	ρ_{liq}[kg·m^{-3}] exp	sim	dev	err [%]	ΔH_{vap}[kJ·mol^{-1}] exp	sim	dev	err [%]
95	76	A0002a		432.4	606.6	298	1.0	763.6	780.3	16.7	2.2	43.5	44.6	1.1	2.5
96	77	A0003a		442.7	606.6	320	0.01	761.5	778.1	16.6	2.2	-	-	-	-
97	77	A0003b		442.7	606.6	298	1.0	-	-	-	-	46.0	45.9	-0.1	-0.1
98	78	A0004a		443.2	606.6	298	1.0	778.9	797.8	18.9	2.4	42.3	46.7	4.4	10.4
99	79	A0005a		433.5	623.0	298	1.0	760.9	782.2	21.3	2.8	45.2	46.1	0.9	1.9
100	80	A0006a		427.0	606.6	298	1.0	-	-	-	-	43.5	43.6	0.1	0.3
101	81	A0007a		410.6	581.4	298	1.0	714.8	740.2	25.4	3.5	43.5	43.2	-0.3	-0.8
102	82	A0008a		442.6	606.6	298	1.0	-	-	-	-	46.4	46.6	0.2	0.4
103	83	A0009a		437.8	606.6	298	1.0	-	-	-	-	46.4	47.0	0.6	1.3
104	84	A0010a		434.8	606.6	298	1.0	-	-	-	-	46.0	46.0	-0.0	-0.0
105	85	A0011a		426.3	606.6	298	1.0	-	-	-	-	45.2	44.8	-0.4	-0.9
106	86	A0012a		428.5	606.6	298	1.0	-	-	-	-	44.8	44.6	-0.2	-0.5
107	87	A0013a		432.0	606.6	298	1.0	-	-	-	-	45.6	45.4	-0.2	-0.4
108	88	A0014a		421.6	606.6	298	1.0	-	-	-	-	45.2	45.1	-0.1	-0.3
109	89	A0015a		421.0	606.6	298	1.0	-	-	-	-	44.4	44.5	0.1	0.3
110	90	A0016a		430.2	606.6	298	1.0	754.5	758.7	4.2	0.6	45.6	45.6	-0.0	-0.0
111	91	A0017a		429.4	606.6	298	1.0	-	-	-	-	46.0	46.5	0.5	1.1
112	92	A0018a		442.9	606.6	298	1.0	-	-	-	-	47.3	47.3	0.0	0.1
113	93	A0019a		435.3	606.6	298	1.0	-	-	-	-	46.4	47.3	0.9	1.9
114	94	A0020a		436.9	606.6	298	1.0	-	-	-	-	46.9	48.0	1.1	2.3
115	95	A0021a		436.1	606.6	298	1.0	-	-	-	-	46.4	46.6	0.2	0.4
116	96	A0022a		434.3	606.6	298	1.0	-	-	-	-	46.4	47.2	0.8	1.8
117	97	A0023a		435.1	606.6	298	1.0	-	-	-	-	46.9	47.3	0.4	0.8
118	98	A0024a		433.4	606.6	298	1.0	-	-	-	-	46.9	46.7	-0.2	-0.4
119	99	A0025a		429.3	606.6	298	1.0	-	-	-	-	46.0	45.8	-0.2	-0.3
120	100	A0026a		430.8	606.6	298	1.0	-	-	-	-	46.9	46.5	-0.4	-0.9
121	101	A0027a		434.3	606.6	298	1.0	-	-	-	-	46.4	45.8	-0.6	-1.2
122	102	A0028a		428.9	609.5	313	0.01	728.1	742.5	14.4	2.0	-	-	-	-
123	102	A0028b		428.9	609.5	298	1.0	-	-	-	-	46.0	45.7	-0.3	-0.7
124	103	A0029a		424.2	606.6	298	1.0	-	-	-	-	45.2	45.8	0.6	1.4
125	104	A0030a		426.0	606.6	298	1.0	736.2	747.4	11.2	1.5	46.0	45.6	-0.4	-0.9
126	105	A0031a		420.2	606.6	298	1.0	-	-	-	-	45.2	44.7	-0.5	-1.0
127	106	A0032a		421.5	606.6	298	1.0	-	-	-	-	45.6	45.3	-0.3	-0.6
128	107	A0033a		424.0	606.6	298	1.0	-	-	-	-	46.0	45.3	-0.7	-1.5
129	108	A0034a		422.1	606.6	298	1.0	720.0	734.4	14.4	2.0	46.4	45.3	-1.1	-2.3
130	109	A0035a		433.3	606.6	298	1.0	-	-	-	-	46.9	46.6	-0.3	-0.6
131	110	A0036a		439.9	606.6	298	1.0	-	-	-	-	46.4	46.3	-0.1	-0.2
132	111	A0037a		434.1	606.6	298	1.0	-	-	-	-	46.9	47.5	0.6	1.3
133	112	A0038a		435.7	606.6	298	1.0	751.9	763.0	11.1	1.5	47.3	47.6	0.3	0.7
134	113	A0039a		433.1	606.6	298	1.0	-	-	-	-	47.3	47.6	0.3	0.6
135	114	A0040a		427.3	606.6	298	1.0	-	-	-	-	46.4	46.0	-0.4	-0.9
136	115	A0041a		429.7	606.6	298	1.0	-	-	-	-	46.9	46.8	-0.1	-0.2
137	116	A0042a		433.9	606.6	298	1.0	-	-	-	-	47.3	46.6	-0.7	-1.5
138	117	A0043a		429.2	606.6	298	1.0	-	-	-	-	47.3	46.4	-0.9	-1.9
139	118	A0044a		420.8	606.6	298	1.0	-	-	-	-	46.4	46.0	-0.4	-1.0
140	119	A0045a		439.5	606.6	298	1.0	-	-	-	-	47.3	47.5	0.2	0.4
141	120	A0046a		434.0	606.6	298	1.0	-	-	-	-	47.2	47.8	0.5	1.2
142	121	A0047a		437.0	606.6	298	1.0	-	-	-	-	47.7	47.5	-0.2	-0.5

Table S.13 – Comparison of experimental and simulated properties using LB combination rules (continued).

n_{sim}	n_{iso}	Code	Outlier	T_m [K]	T_b [K]	T [K]	P [bar]	ρ_{liq}[kg·m^{-3}] exp	sim	dev	err [%]	ΔH_{vap}[kJ·mol^{-1}] exp	sim	dev	err [%]
143	122	A0048a		430.7	606.6	298	1.0	-	-	-	-	48.1	47.4	-0.7	-1.5
144	123	A0049a		434.4	606.6	298	1.0	-	-	-	-	48.5	47.2	-1.3	-2.6
145	124	A0050a		430.1	606.6	298	1.0	720.8	738.0	17.2	2.4	49.0	47.0	-2.0	-4.2
146	125	A0051a		437.1	606.6	298	1.0	747.2	756.8	9.6	1.3	47.7	47.9	0.2	0.4
147	126	A0052a		435.4	606.6	298	1.0	-	-	-	-	47.7	48.0	0.3	0.6
148	127	A0053a		436.2	606.6	298	1.0	-	-	-	-	48.1	48.0	-0.1	-0.1
149	128	A0054a		432.1	606.6	298	1.0	735.4	748.0	12.6	1.7	47.3	47.9	0.6	1.2
150	129	A0055a		434.4	606.6	298	1.0	-	-	-	-	48.1	47.6	-0.5	-1.0
151	130	A0056a		435.3	606.6	298	1.0	-	-	-	-	48.5	48.6	0.1	0.2
152	131	A0057a		436.6	606.6	298	1.0	741.0	750.1	9.1	1.2	48.1	48.3	0.2	0.5
153	132	A0058a		437.5	606.6	293	1.0	737.7	747.7	10.0	1.4	48.1	48.5	0.4	0.8
154	133	A0059a		431.4	606.6	298	1.0	-	-	-	-	47.7	47.2	-0.5	-1.0
155	134	A0060a		429.4	606.6	298	1.0	-	-	-	-	47.3	47.2	-0.1	-0.2
156	135	A0061a		432.9	606.6	298	1.0	-	-	-	-	48.1	47.2	-0.9	-1.8
157	136	A0062a		432.6	606.6	298	1.0	-	-	-	-	48.5	47.7	-0.8	-1.7
158	137	A0063a		429.1	606.6	298	1.0	722.6	738.2	15.6	2.2	48.5	47.8	-0.7	-1.5
159	138	A0064a		434.0	606.6	298	1.0	732.4	742.1	9.7	1.3	47.3	47.7	0.4	0.8
160	139	A0065a		431.7	606.6	298	1.0	726.4	737.4	11.0	1.5	49.0	47.7	-1.3	-2.6
161	140	A0066a		433.5	606.6	293	1.0	731.3	740.1	8.8	1.2	49.3	48.0	-1.3	-2.6
162	141	A0067a		433.0	606.6	298	1.0	720.2	732.5	12.3	1.7	47.7	47.9	0.2	0.5
163	142	A0068a		430.7	606.6	298	1.0	732.1	742.7	10.6	1.4	48.5	49.0	0.5	1.0
164	143	A0069a		436.8	606.6	298	1.0	734.3	743.5	9.2	1.2	48.1	48.9	0.8	1.6
165	144	A0070a		439.7	606.6	298	1.0	735.9	743.7	7.8	1.1	49.0	49.0	0.0	0.0
166	145	A0071a		438.3	606.6	293	1.0	732.6	743.4	10.8	1.5	49.8	49.4	-0.4	-0.8
167	146	A0072a		438.9	606.6	293	1.0	732.3	742.7	10.4	1.4	49.5	49.5	0.0	0.1
168	147	A0073a		440.9	606.6	293	1.0	735.4	743.5	8.1	1.1	50.2	49.6	-0.6	-1.3
169	148	A0074a		440.1	606.6	293	1.0	728.1	738.1	10.0	1.4	51.0	49.4	-1.6	-3.1
170	149	A0075a		447.3	617.7	298	1.01	725.9	736.9	11.0	1.5	50.2	50.5	0.3	0.5
171	150	F1101a		194.8	317.4	197	1.0	876.6	847.0	-29.6	-3.4	17.1	16.2	-0.9	-5.3
172	150	F1101b		194.8	317.4	298	38.39	528.3	532.2	3.9	0.7	-	-	-	-
173	150	F1101c	vap	194.8	317.4	298	1.0	574.4	-	-	-	0.0	-	-	-
174	151	F1201a		221.5	351.3	221	1.0	1213.8	1212.1	-1.7	-0.1	20.9	20.1	-0.8	-3.7
175	151	F1201b	×	221.5	351.3	298	17.62	891.6	997.5	105.9	11.9	-	-	-	-
176	151	F1201c		221.5	351.3	298	1.0	961.0	984.9	23.9	2.5	-	-	-	-
177	152	F1301a	×	191.0	299.0	191	1.0	1442.9	1801.0	358.1	24.8	16.7	19.5	2.8	16.6
178	152	F1301b	vap	191.0	299.0	298	47.1	636.7	-	-	-	0.0	-	-	-
179	152	F1301c	vap	191.0	299.0	298	1.0	666.7	-	-	-	0.0	-	-	-
180	153	F1401a		145.1	227.5	145	1.0	1605.2	1677.0	71.8	4.5	12.3	8.4	-3.9	-31.5
181	154	F2101a		235.4	375.3	236	1.0	817.6	793.8	-23.8	-2.9	20.7	20.1	-0.6	-2.8
182	154	F2101b		235.4	375.3	298	9.09	707.5	682.3	-25.2	-3.6	-	-	-	-
183	155	F2201a	×	247.3	386.4	250	1.0	1009.0	904.2	-104.8	-10.4	22.7	18.9	-3.8	-16.7
184	155	F2201b	×	247.3	386.4	298	6.25	907.0	782.8	-124.2	-13.7	-	-	-	-
185	156	F2301a	×	225.8	345.9	220	1.0	1182.9	848.2	-334.7	-28.3	19.2	11.8	-7.4	-38.7
186	156	F2301b	vap	225.8	345.9	298	12.92	-	-	-	-	13.1	-	-	-
187	157	F3101a		263.8	421.1	264	1.0	769.2	721.9	-47.3	-6.1	-	-	-	-
188	158	F3102a		269.9	421.1	271	1.0	781.8	775.7	-6.1	-0.8	-	-	-	-
189	159	F3201a		281.1	430.4	276	0.62	-	-	-	-	25.1	23.1	-2.0	-8.1
190	160	F3202a		314.4	430.4	298	1.0	1005.7	995.7	-10.0	-1.0	-	-	-	-

Table S.13 – Comparison of experimental and simulated properties using LB combination rules (continued).

n_{sim}	n_{iso}	Code	Outlier	T_m [K]	T_b [K]	T [K]	P [bar]	ρ_{liq}[kg·m^{-3}] exp	sim	dev	err [%]	ΔH_{vap}[kJ·mol^{-1}] exp	sim	dev	err [%]
191	161	F4101a		285.2	460.3	285	1.0	752.7	683.8	-68.9	-9.1	-	-	-	-
192	161	F4101b		285.2	460.3	298	1.39	735.3	661.0	-74.3	-10.1	-	-	-	-
193	162	F4102a		298.2	460.3	298	1.0	756.6	728.4	-28.2	-3.7	-	-	-	-
194	163	F4103a		305.6	460.3	298	1.0	770.8	768.1	-2.7	-0.4	-	-	-	-
195	164	F4201a		350.9	463.6	298	1.0	976.7	978.9	2.2	0.2	-	-	-	-
196	165	F4301a	×	289.9	406.6	298	1.38	1010.0	872.4	-137.6	-13.6	-	-	-	-
197	166	F5101a		317.9	494.6	298	1.0	773.7	729.0	-44.7	-5.8	-	-	-	-
198	167	F5102a		329.1	494.6	298	0.34	791.5	783.3	-8.1	-1.0	-	-	-	-
199	168	F5103a		335.9	494.6	298	1.0	784.9	787.0	2.1	0.3	30.9	31.9	1.0	3.1
200	169	F6101a		359.4	525.4	293	1.0	791.4	778.7	-12.7	-1.6	-	-	-	-
201	170	F6102a		364.6	525.4	298	1.0	795.8	799.5	3.7	0.5	35.6	36.5	1.0	2.7
202	171	F6201a		-	-	293	1.0	888.2	842.6	-45.6	-5.1	-	-	-	-
203	172	F6202a		-	-	298	1.0	940.7	951.7	11.0	1.2	-	-	-	-
204	173	F7101a		391.1	553.5	298	1.0	800.9	808.3	7.5	0.9	40.8	41.2	0.4	1.1
205	174	F8101a		415.4	579.2	298	1.0	806.7	815.6	8.9	1.1	-	-	-	-
206	175	F9101a		438.1	603.2	348	1.0	-	-	-	-	46.8	47.3	0.5	1.0
207	176	F0101a		459.4	625.5	293	1.0	819.4	829.6	10.2	1.2	-	-	-	-
208	177	C1101a		248.9	416.2	249	1.0	1008.3	952.6	-55.7	-5.5	-	-	-	-
209	178	C1201a		312.9	510.0	298	1.0	1316.4	1313.5	-2.9	-0.2	28.8	28.5	-0.3	-1.2
210	179	C1301a		334.3	536.4	298	1.0	1479.5	1505.7	26.2	1.8	31.1	33.8	2.7	8.8
211	180	C1401a		349.8	556.4	298	1.0	1584.3	1625.5	41.2	2.6	32.4	36.6	4.2	12.9
212	181	C2101a		285.4	460.4	285	1.0	906.2	879.5	-26.7	-2.9	24.9	22.6	-2.3	-9.2
213	181	C2101b		285.4	460.4	298	1.39	890.0	860.1	-29.9	-3.4	-	-	-	-
214	182	C2201a		330.4	523.0	298	1.0	1168.1	1146.8	-21.3	-1.8	30.6	29.5	-1.1	-3.8
215	183	C2202a		356.6	561.6	298	1.0	1245.6	1230.3	-15.3	-1.2	34.4	34.0	-0.4	-1.2
216	184	C2301a		347.2	545.0	298	1.0	1329.3	1329.2	-0.1	-0.0	32.4	32.8	0.4	1.2
217	185	C2302a		387.0	551.3	298	1.0	1432.8	1434.6	1.9	0.1	40.1	41.5	1.4	3.5
218	186	C3101a		308.9	496.5	298	1.0	855.6	826.9	-28.8	-3.4	-	-	-	-
219	187	C3201a		369.5	573.3	298	1.0	1153.0	1137.5	-15.5	-1.3	36.2	35.6	-0.6	-1.7
220	188	C3202a		393.6	573.3	298	1.0	1180.0	1171.5	-8.5	-0.7	40.6	38.2	-2.4	-6.0
221	189	C4101a		323.8	530.2	298	1.0	836.3	815.8	-20.5	-2.5	28.6	25.9	-2.7	-9.3
222	190	C4102a		342.0	530.2	298	1.0	871.4	863.3	-8.0	-0.9	31.7	30.2	-1.5	-4.8
223	191	C4103a		341.2	520.6	298	1.01	867.5	853.1	-14.3	-1.7	31.5	29.7	-1.8	-5.7
224	192	C4104a		351.6	530.2	298	1.01	880.4	873.2	-7.2	-0.8	33.5	31.7	-1.8	-5.4
225	193	C4201a		391.1	599.9	298	1.0	1106.3	1090.3	-16.0	-1.4	-	-	-	-
226	194	C4202a		397.1	599.9	298	1.0	1111.8	1109.1	-2.7	-0.2	40.1	40.1	-0.0	-0.0
227	195	C4203a		427.1	599.9	298	1.0	-	-	-	-	46.4	43.8	-2.6	-5.6
228	196	C5101a		358.8	560.5	298	1.0	859.6	852.4	-7.2	-0.8	-	-	-	-
229	197	C5102a		373.7	560.5	298	1.0	875.0	877.4	2.4	0.3	-	-	-	-
230	198	C5103a		371.7	560.5	298	1.0	870.0	869.0	-1.0	-0.1	36.2	35.3	-0.9	-2.4
231	199	C5104a		369.7	560.5	298	1.0	866.0	857.0	-9.0	-1.0	36.0	34.6	-1.4	-4.0
232	200	C5105a		381.5	560.5	298	1.0	877.8	874.9	-2.9	-0.3	38.2	36.5	-1.7	-4.4
233	201	C5201a		453.1	624.5	298	1.0	1095.6	1092.0	-3.6	-0.3	51.3	48.7	-2.6	-5.1
234	202	C6101a		408.2	588.0	298	1.0	873.5	874.8	1.3	0.1	42.0	41.2	-0.8	-1.9
235	203	C6201a		-	-	298	1.0	1064.0	1074.9	10.9	1.0	-	-	-	-
236	204	C6202a		-	-	298	1.0	1044.1	1038.0	-6.1	-0.6	-	-	-	-
237	205	C6203a		477.1	647.4	298	1.01	1063.7	1065.7	2.0	0.2	-	-	-	-
238	206	C7101a		433.6	613.5	298	1.0	871.5	874.7	3.2	0.4	47.0	46.0	-1.0	-2.2

Table S.13 – Comparison of experimental and simulated properties using LB combination rules (continued).

n_{sim}	n_{iso}	Code	Outlier	T_m [K]	T_b [K]	T [K]	P [bar]	$\rho_{\text{liq}}[\text{kg}\cdot\text{m}^{-3}]$ exp	sim	dev	err [%]	$\Delta H_{\text{vap}}[\text{kJ}\cdot\text{mol}^{-1}]$ exp	sim	dev	err [%]
239	207	C8101a		456.6	637.1	298	1.0	869.4	875.0	5.6	0.6	51.4	50.7	-0.7	-1.4
240	208	C9101a		478.4	659.3	298	1.0	867.4	874.8	7.4	0.9	-	-	-	-
241	209	C0101a		499.0	680.1	298	1.0	865.8	874.5	8.6	1.0	64.0	60.1	-3.9	-6.1
242	210	B1101a		276.7	467.0	273	1.0	1729.8	1661.7	-68.1	-3.9	-	-	-	-
243	210	B1101b		276.7	467.0	298	2.25	1662.1	1593.9	-68.2	-4.1	-	-	-	-
244	210	B1101c		276.7	467.0	281	1.0	-	-	-	-	24.6	21.6	-3.0	-12.4
245	211	B1201a		370.1	615.9	298	0.06	2482.0	2455.7	-26.3	-1.1	37.0	37.1	0.1	0.3
246	212	B1301a		422.4	656.4	298	1.0	2877.2	2872.5	-4.7	-0.2	46.1	48.6	2.5	5.4
247	213	B1401a		462.6	706.8	374	1.0	2953.3	3011.2	57.9	2.0	-	-	-	-
248	213	B1401b		462.6	706.8	384	1.0	-	-	-	-	48.2	54.0	5.8	12.0
249	214	B2101a		311.5	503.8	298	1.0	1451.2	1424.8	-26.5	-1.8	-	-	-	-
250	214	B2101b		311.5	503.8	305	1.0	-	-	-	-	27.6	25.6	-2.0	-7.4
251	215	B2201a		381.1	635.8	298	1.0	2091.8	2065.0	-26.7	-1.3	-	-	-	-
252	216	B2202a		404.5	650.1	298	1.0	2169.5	2162.7	-6.8	-0.3	41.7	42.0	0.3	0.8
253	217	B2301a		462.1	671.9	298	1.0	2610.1	2603.7	-6.4	-0.2	-	-	-	-
254	218	B3101a		332.6	532.5	298	1.0	1301.4	1280.2	-21.2	-1.6	30.2	28.4	-1.8	-5.8
255	219	B3102a		344.1	536.9	298	1.0	1345.5	1334.3	-11.2	-0.8	31.9	30.5	-1.4	-4.4
256	220	B3201a		413.2	654.7	298	1.0	1925.0	1924.8	-0.2	-0.0	41.7	43.9	2.2	5.2
257	221	B3202a		440.4	654.7	298	1.0	1971.2	1962.0	-9.2	-0.5	-	-	-	-
258	222	B3301a		464.1	686.8	293	1.0	2298.5	2367.4	68.9	3.0	-	-	-	-
259	223	B3302a		474.1	686.8	293	1.0	2354.8	2363.8	9.0	0.4	-	-	-	-
260	224	B3303a		495.3	686.8	298	1.0	2411.0	2404.8	-6.3	-0.3	-	-	-	-
261	225	B4101a		346.4	557.6	298	1.0	1212.5	1202.2	-10.3	-0.9	31.8	30.1	-1.7	-5.4
262	226	B4102a		364.7	557.6	298	1.0	1257.1	1255.5	-1.6	-0.1	34.9	33.8	-1.1	-3.2
263	227	B4103a		364.4	557.6	298	1.0	1253.6	1243.5	-10.1	-0.8	34.8	33.3	-1.5	-4.3
264	228	B4104a		374.8	557.6	298	1.0	1268.6	1264.8	-3.8	-0.3	36.6	35.3	-1.3	-3.4
265	229	B4201a		448.1	672.7	298	1.0	1799.5	1814.3	14.8	0.8	-	-	-	-
266	230	B4202a		439.5	672.7	298	1.0	1787.0	1798.0	11.0	0.6	45.6	48.3	2.7	6.0
267	231	B4203a		448.1	672.7	293	1.0	1796.0	1792.0	-4.0	-0.2	-	-	-	-
268	232	B4301a		503.1	701.1	294	1.0	2175.3	2213.0	37.7	1.7	-	-	-	-
269	233	B4302a		493.1	701.1	298	1.0	2180.3	2242.7	62.4	2.9	-	-	-	-
270	234	B5101a		379.1	581.0	298	0.03	1193.5	1215.4	22.0	1.8	-	-	-	-
271	235	B5102a		381.1	581.0	298	0.03	1209.5	1200.4	-9.1	-0.8	-	-	-	-
272	236	B5103a		393.6	581.0	298	1.0	1214.4	1220.4	6.0	0.5	-	-	-	-
273	237	B5104a		391.8	581.0	298	1.0	1205.1	1208.9	3.8	0.3	-	-	-	-
274	238	B5105a		393.6	581.0	298	1.0	1200.7	1207.5	6.8	0.6	-	-	-	-
275	239	B5106a		390.6	581.0	298	1.0	1200.5	1195.9	-4.6	-0.4	38.5	38.2	-0.3	-0.8
276	240	B5107a		402.7	581.0	298	1.0	1211.4	1214.6	3.2	0.3	40.9	40.1	-0.8	-1.9
277	241	B5201a		453.1	689.9	293	1.0	1669.5	1706.8	37.3	2.2	-	-	-	-
278	242	B6101a		403.1	602.9	293	1.0	1179.2	1195.0	15.8	1.3	-	-	-	-
279	243	B6102a		414.4	602.9	298	1.0	1157.2	1170.0	12.9	1.1	-	-	-	-
280	244	B6103a		428.4	602.9	298	1.0	1168.8	1174.8	6.0	0.5	45.6	44.8	-0.8	-1.8
281	245	B7101a		452.1	623.5	298	1.0	1134.8	1143.7	8.9	0.8	50.4	49.6	-0.8	-1.6
282	246	B8101a		473.9	643.0	298	1.0	1107.7	1118.2	10.5	1.0	55.1	54.3	-0.8	-1.4
283	247	B9101a		494.6	661.6	298	1.0	1084.9	1097.3	12.5	1.1	-	-	-	-
284	248	B0101a		513.8	679.3	298	1.0	1062.5	1079.1	16.6	1.6	-	-	-	-
285	248	B0101b		513.8	679.3	398	1.0	-	-	-	-	56.6	56.8	0.2	0.4
286	249	I1101a		315.6	522.4	298	1.0	2264.5	2196.2	-68.3	-3.0	27.5	26.8	-0.7	-2.6

Table S.13 – Comparison of experimental and simulated properties using LB combination rules (continued).

n_{sim}	n_{iso}	Code	Outlier	T_m [K]	T_b [K]	T [K]	P [bar]	ρ_{liq}[kg·m^{-3}] exp	sim	dev	err [%]	ΔH_{vap}[kJ·mol^{-1}] exp	sim	dev	err [%]
287	250	I1201a	×	455.1	697.5	298	1.0	3307.8	3173.9	-133.9	-4.0	49.0	47.6	-1.4	-2.9
288	251	I2101a		345.4	562.2	298	1.0	1924.0	1904.9	-19.1	-1.0	31.7	31.0	-0.7	-2.3
289	252	I3101a		362.6	583.8	298	1.0	1694.5	1684.0	-10.5	-0.6	34.1	33.5	-0.6	-1.7
290	253	I3102a		375.6	602.3	298	1.0	1737.2	1742.2	5.0	0.3	36.0	35.6	-0.4	-1.2
291	254	I3201a		496.1	775.2	298	1.0	2565.1	2546.7	-18.4	-0.7	-	-	-	-
292	254	I3201b		496.1	775.2	369	0.01	-	-	-	-	53.5	52.7	-0.8	-1.5
293	255	I4101a		373.2	596.8	298	0.06	1536.0	1559.4	23.4	1.5	35.7	35.5	-0.2	-0.5
294	256	I4102a		393.6	625.0	298	0.02	1595.1	1608.4	13.3	0.8	38.8	38.8	-0.0	-0.1
295	257	I4103a		393.1	623.3	298	1.0	1589.0	1597.0	8.0	0.5	38.5	38.3	-0.2	-0.5
296	258	I4104a		403.7	638.8	298	1.0	1606.7	1615.1	8.4	0.5	40.3	40.3	0.0	0.1
297	259	I4201a		477.2	806.4	298	1.0	2349.6	2338.1	-11.5	-0.5	59.0	60.3	1.3	2.1
298	260	I5101a		407.1	670.9	293	1.0	1494.0	1537.6	43.6	2.9	-	-	-	-
299	261	I5102a		402.1	670.9	298	0.01	1486.6	1522.4	35.8	2.4	-	-	-	-
300	262	I5103a		413.1	670.9	293	1.0	1524.0	1526.5	2.5	0.2	-	-	-	-
301	263	I5104a		418.1	670.9	298	1.0	1505.5	1524.2	18.7	1.2	-	-	-	-
302	264	I5105a		421.4	659.1	298	1.0	1495.2	1514.8	19.6	1.3	42.2	43.9	1.7	4.1
303	265	I5106a		416.1	670.9	293	1.0	1500.9	1513.5	12.6	0.8	-	-	-	-
304	266	I5107a		430.1	671.4	298	1.0	1507.3	1522.1	14.7	1.0	44.4	45.0	0.6	1.3
305	267	I5201a		500.1	843.6	298	1.0	2173.4	2175.3	1.9	0.1	-	-	-	-
306	268	I6101a		441.1	697.2	293	1.0	1443.0	1464.1	21.1	1.5	-	-	-	-
307	269	I6102a		442.1	697.2	293	1.0	1419.3	1443.1	23.8	1.7	-	-	-	-
308	270	I6103a		454.5	704.4	298	1.0	1431.8	1448.8	17.0	1.2	-	-	-	-
309	270	I6103b		454.5	704.4	346	1.0	-	-	-	-	46.2	47.1	0.9	2.0
310	271	I6201a		522.5	831.8	298	1.0	2034.2	2046.9	12.7	0.6	-	-	-	-
311	272	I7101a		477.1	736.7	298	1.0	1371.9	1391.0	19.1	1.4	-	-	-	-
312	272	I7101b		477.1	736.7	357	0.01	-	-	-	-	48.4	51.1	2.6	5.4
313	273	I8101a		498.3	764.8	298	1.0	1326.7	1343.3	16.7	1.3	-	-	-	-
314	273	I8101b		498.3	764.8	374	0.01	-	-	-	-	50.9	54.4	3.5	6.9
315	274	I9101a		518.1	-	298	1.0	1283.6	1303.8	20.2	1.6	-	-	-	-
316	275	I0101a		-	-	298	1.0	1241.2	1263.5	22.3	1.8	-	-	-	-
317	276	I0102a		536.9	-	293	1.0	1256.7	1275.8	19.1	1.5	69.8	68.7	-1.1	-1.5
318	277	O2101a		248.3	400.1	248	1.0	735.0	725.4	-9.7	-1.3	21.6	25.3	3.7	17.1
319	277	O2101b		248.3	400.1	298	9.06	661.9	664.1	2.1	0.3	17.8	23.2	5.4	30.5
320	278	O3101a		280.5	437.8	273	1.0	726.0	714.8	-11.2	-1.5	-	-	-	-
321	278	O3101b		280.5	437.8	298	1.82	691.9	685.9	-6.0	-0.9	-	-	-	-
322	278	O3101c		280.5	437.8	280	1.0	-	-	-	-	31.2	26.8	-4.4	-14.0
323	279	O3201a	×	315.0	480.6	298	0.51	854.1	773.9	-80.3	-9.4	28.9	25.0	-3.9	-13.6
324	280	O4101a		303.9	464.5	298	1.0	709.0	697.7	-11.3	-1.6	26.4	28.3	1.9	7.3
325	281	O4102a		311.7	476.2	298	1.0	719.2	713.3	-5.9	-0.8	27.9	30.2	2.3	8.2
326	282	O4103a		307.6	466.7	298	1.0	707.8	691.5	-16.3	-2.3	27.2	27.5	0.3	1.2
327	283	O4201a	×	337.6	-	293	1.0	851.6	772.8	-78.8	-9.3	36.4	27.6	-8.8	-24.1
328	284	O4202a		357.2	536.1	298	1.01	861.4	856.0	-5.4	-0.6	36.8	39.4	2.6	7.0
329	285	O5101a		328.4	497.1	298	1.0	735.2	722.9	-12.4	-1.7	30.4	30.6	0.2	0.6
330	286	O5102a		331.7	500.0	298	1.0	727.2	723.1	-4.1	-0.6	-	-	-	-
331	287	O5103a		332.1	500.0	298	1.0	736.7	728.7	-8.0	-1.1	-	-	-	-
332	288	O5104a		326.1	500.0	298	1.0	717.3	704.4	-12.9	-1.8	30.0	30.8	0.8	2.5
333	289	O5105a		343.4	512.7	298	1.0	739.4	737.4	-2.0	-0.3	32.5	34.8	2.3	7.2
334	290	O5106a		337.0	500.2	298	1.0	727.0	715.2	-11.8	-1.6	31.4	31.7	0.3	0.9

Table S.13 – Comparison of experimental and simulated properties using LB combination rules (continued).

n_{sim}	n_{iso}	Code	Outlier	T_m [K]	T_b [K]	T [K]	P [bar]	ρ_{liq}[kg·m^{-3}] exp	sim	dev	err [%]	ΔH_{vap}[kJ·mol^{-1}] exp	sim	dev	err [%]
335	291	O5201a		356.1	-	298	1.01	845.1	793.5	-51.6	-6.1	37.6	32.0	-5.6	-14.9
336	292	O5202a		375.2	-	298	1.0	846.0	835.0	-11.0	-1.3	39.8	40.5	0.7	1.8
337	293	O5203a		361.1	524.0	298	1.0	825.2	784.1	-41.1	-5.0	35.7	33.5	-2.2	-6.3
338	294	O6101a		359.4	526.0	298	1.0	765.9	756.6	-9.3	-1.2	35.0	35.1	0.1	0.2
339	295	O6102a		345.9	526.0	298	1.0	735.2	729.3	-5.9	-0.8	33.1	34.0	0.9	2.7
340	296	O6103a		356.1	526.0	298	1.0	754.2	746.9	-7.3	-1.0	-	-	-	-
341	297	O6104a		341.4	500.1	298	1.0	718.7	716.1	-2.6	-0.4	32.7	33.7	1.0	3.2
342	298	O6105a		363.1	526.0	298	0.07	746.1	749.9	3.8	0.5	-	-	-	-
343	299	O6106a		363.1	526.0	298	1.0	749.0	751.5	2.5	0.3	-	-	-	-
344	300	O6107a		354.2	526.0	298	1.0	734.0	724.2	-9.8	-1.3	-	-	-	-
345	301	O6108a		364.1	526.0	298	0.06	749.9	745.9	-4.0	-0.5	-	-	-	-
346	302	O6109a		354.4	526.0	298	1.0	738.3	730.3	-8.0	-1.1	-	-	-	-
347	303	O6110a		353.1	526.0	298	1.0	732.4	722.0	-10.4	-1.4	-	-	-	-
348	304	O6111a		372.1	546.5	298	1.0	755.2	753.6	-1.6	-0.2	-	-	-	-
349	305	O6112a		365.4	526.0	298	1.0	744.7	735.6	-9.1	-1.2	36.3	36.3	-0.0	-0.1
350	306	O6113a		362.8	530.6	298	1.0	741.9	731.0	-10.9	-1.5	35.7	35.8	0.0	0.1
351	307	O6201a		-	-	293	1.0	844.6	798.6	-46.0	-5.5	-	-	-	-
352	308	O6202a		376.8	539.7	298	1.01	822.0	774.9	-47.1	-5.7	39.6	35.7	-3.9	-9.8
353	309	O6203a		-	-	298	1.0	852.9	855.2	2.3	0.3	-	-	-	-
354	310	O6204a		392.6	637.8	298	1.0	836.2	822.8	-13.4	-1.6	43.2	44.2	1.0	2.4
355	311	O6301a	×	432.9	608.0	298	1.0	939.2	931.0	-8.2	-0.9	48.0	56.2	8.2	17.0
356	312	O7101a		378.7	549.7	298	1.0	736.4	733.1	-3.3	-0.5	34.5	35.8	1.3	3.8
357	313	O7102a		375.1	546.0	298	1.0	761.8	757.3	-4.5	-0.6	38.2	38.4	0.1	0.4
358	314	O7103a		373.1	549.7	298	1.0	746.7	742.5	-4.1	-0.6	37.2	37.8	0.6	1.6
359	315	O7104a		371.2	549.7	298	1.0	734.9	728.9	-6.0	-0.8	-	-	-	-
360	316	O7105a		-	549.7	298	1.0	739.6	737.3	-2.3	-0.3	-	-	-	-
361	317	O7106a		385.6	549.7	298	1.0	752.1	748.1	-4.0	-0.5	-	-	-	-
362	318	O7107a		378.1	549.7	298	1.0	744.0	735.6	-8.4	-1.1	-	-	-	-
363	319	O7108a		-	-	298	1.0	750.1	742.2	-7.9	-1.0	-	-	-	-
364	320	O7109a		380.1	-	298	1.0	746.0	739.7	-6.3	-0.8	-	-	-	-
365	321	O7110a		398.1	-	298	1.0	766.3	767.1	0.8	0.1	-	-	-	-
366	322	O7111a		391.1	-	298	1.0	757.2	751.1	-6.1	-0.8	-	-	-	-
367	323	O7112a		390.1	-	298	1.0	754.2	747.1	-7.1	-0.9	-	-	-	-
368	324	O7201a		404.6	-	405	1.0	-	-	-	-	34.8	37.9	3.1	8.8
369	325	O7202a		387.1	-	298	1.0	868.8	806.0	-62.8	-7.2	43.9	44.4	0.5	1.1
370	326	O7203a		-	-	298	1.0	813.7	781.3	-32.4	-4.0	-	-	-	-
371	327	O7204a		-	-	298	1.0	851.6	855.3	3.7	0.4	-	-	-	-
372	328	O7205a		420.1	659.3	298	1.0	-	-	-	-	47.8	48.1	0.3	0.6
373	329	O7206a		-	-	298	1.0	840.3	831.2	-9.1	-1.1	-	-	-	-
374	330	O7207a		-	-	298	1.0	831.2	818.2	-13.0	-1.6	45.9	45.3	-0.6	-1.4
375	331	O7208a		-	-	298	1.0	832.7	821.1	-11.6	-1.4	46.8	47.5	0.7	1.5
376	332	O7301a		416.1	-	298	1.0	893.8	822.7	-71.1	-8.0	47.8	41.4	-6.4	-13.3
377	333	O7302a		416.9	-	293	1.0	922.9	912.2	-10.7	-1.2	-	-	-	-
378	334	O8101a		380.4	550.0	298	1.0	757.8	738.9	-18.9	-2.5	37.6	36.3	-1.3	-3.6
379	335	O8102a		439.9	571.4	298	1.0	748.0	746.7	-1.3	-0.2	40.1	40.0	-0.1	-0.1
380	336	O8103a		-	-	298	1.0	757.1	751.9	-5.2	-0.7	-	-	-	-
381	337	O8104a		-	571.4	298	1.0	758.1	755.7	-2.4	-0.3	42.3	42.2	-0.1	-0.1
382	338	O8105a		395.9	571.4	298	1.0	745.2	738.1	-7.1	-1.0	41.2	40.4	-0.8	-1.9

Table S.13 – Comparison of experimental and simulated properties using LB combination rules (continued).

n_{sim}	n_{iso}	Code	Outlier	T_m [K]	T_b [K]	T [K]	P [bar]	$\rho_{\mathrm{liq}}[\mathrm{kg \cdot m^{-3}}]$ exp	sim	dev	err [%]	$\Delta H_{\mathrm{vap}}[\mathrm{kJ \cdot mol^{-1}}]$ exp	sim	dev	err [%]
383	339	O8106a		-	-	298	1.0	750.8	745.5	-5.4	-0.7	-	-	-	-
384	340	O8107a		-	-	298	1.0	754.5	750.2	-4.3	-0.6	-	-	-	-
385	341	O8108a		394.2	571.4	298	1.0	758.8	753.8	-5.0	-0.7	-	-	-	-
386	342	O8109a		-	-	298	1.0	760.1	757.9	-2.2	-0.3	-	-	-	-
387	343	O8110a		424.1	571.4	298	1.0	755.2	751.0	-4.2	-0.6	-	-	-	-
388	344	O8111a		-	-	298	1.0	761.1	756.1	-5.0	-0.7	-	-	-	-
389	345	O8112a		-	571.4	298	1.0	759.8	753.6	-6.2	-0.8	-	-	-	-
390	346	O8113a		424.1	-	298	1.0	775.6	777.0	1.4	0.2	-	-	-	-
391	347	O8114a		415.1	-	298	1.0	768.2	763.6	-4.6	-0.6	-	-	-	-
392	348	O8115a		-	571.4	298	1.0	765.3	760.4	-4.9	-0.6	-	-	-	-
393	349	O8116a		413.4	584.1	298	1.0	764.1	760.7	-3.4	-0.4	44.7	45.0	0.3	0.7
394	350	O8201a		478.1	-	298	1.0	825.6	789.2	-36.4	-4.4	-	-	-	-
395	351	O8202a		-	-	298	1.0	851.8	855.8	4.0	0.5	-	-	-	-
396	352	O8203a		-	-	298	1.0	843.3	841.2	-2.1	-0.2	-	-	-	-
397	353	O8204a		-	-	298	1.0	840.7	836.5	-4.2	-0.5	-	-	-	-
398	354	O8205a		-	-	298	1.0	833.5	826.0	-7.5	-0.9	-	-	-	-
399	355	O8206a		434.0	-	298	1.0	833.1	824.8	-8.3	-1.0	50.9	51.6	0.8	1.5
400	356	O8207a		478.1	-	298	1.0	831.2	820.1	-11.1	-1.3	50.6	50.8	0.2	0.3
401	357	O8301a		462.1	624.0	298	1.0	903.3	889.9	-13.4	-1.5	56.4	60.7	4.3	7.5
402	358	O9101a		-	-	298	1.01	792.4	788.4	-4.0	-0.5	-	-	-	-
403	359	O9102a		-	-	298	1.0	761.6	762.7	1.1	0.1	-	-	-	-
404	360	O9103a		-	-	298	1.0	766.4	766.9	0.5	0.1	-	-	-	-
405	361	O9104a		-	-	298	1.0	759.4	760.3	0.9	0.1	-	-	-	-
406	362	O9105a		-	-	298	1.0	764.3	763.9	-0.4	-0.1	-	-	-	-
407	363	O9106a		-	-	298	1.0	767.6	768.3	0.7	0.1	-	-	-	-
408	364	O9107a		-	-	298	1.0	765.6	762.2	-3.4	-0.4	-	-	-	-
409	365	O9108a		-	-	298	1.0	802.1	780.2	-21.9	-2.7	-	-	-	-
410	366	O9109a		-	-	298	1.0	768.7	766.7	-2.0	-0.3	-	-	-	-
411	367	O9110a		-	-	298	1.0	767.8	763.9	-3.9	-0.5	-	-	-	-
412	368	O9111a		-	591.5	298	1.0	783.0	785.6	2.6	0.3	-	-	-	-
413	369	O9112a		-	-	298	1.0	775.3	773.6	-1.6	-0.2	-	-	-	-
414	370	O9113a		-	-	298	1.0	773.0	770.5	-2.5	-0.3	-	-	-	-
415	371	O9114a		-	-	298	1.0	772.7	770.5	-2.2	-0.3	-	-	-	-
416	372	O9201a		438.6	-	298	1.0	820.3	795.2	-25.1	-3.1	-	-	-	-
417	373	O9202a		-	-	298	1.0	844.0	843.1	-0.9	-0.1	-	-	-	-
418	374	O9203a		-	-	298	1.0	838.6	830.0	-8.6	-1.0	-	-	-	-
419	375	O9204a		-	-	298	1.0	840.6	839.0	-1.6	-0.2	-	-	-	-
420	376	O9205a		452.4	-	298	1.0	831.5	812.6	-18.9	-2.3	-	-	-	-
421	377	O9206a		-	-	298	1.0	-	-	-	-	54.7	54.9	0.2	0.4
422	378	O0101a		-	-	298	1.0	-	-	-	-	45.3	46.1	0.8	1.9
423	379	O0102a		-	-	298	1.0	-	-	-	-	53.2	51.4	-1.8	-3.4
424	380	O0103a		-	-	293	1.0	784.8	779.8	-5.0	-0.6	-	-	-	-
425	381	O0104a		445.6	610.4	298	1.0	771.5	775.0	3.5	0.5	51.4	51.8	0.4	0.8
426	382	O0105a		-	-	293	1.0	777.8	776.6	-1.2	-0.2	-	-	-	-
427	383	O0106a		-	-	298	1.0	775.1	777.2	2.1	0.3	-	-	-	-
428	384	O0107a		-	-	298	1.0	771.2	771.2	-0.0	-0.0	-	-	-	-
429	385	O0108a		-	-	298	1.0	782.1	777.5	-4.6	-0.6	-	-	-	-
430	386	O0109a		-	-	293	1.0	787.4	779.2	-8.2	-1.0	-	-	-	-

Table S.13 – Comparison of experimental and simulated properties using LB combination rules (continued).

n_{sim}	n_{iso}	Code	Outlier	T_m [K]	T_b [K]	T [K]	P [bar]	ρ_{liq}[kg·m^{-3}] exp	sim	dev	err [%]	ΔH_{vap}[kJ·mol^{-1}] exp	sim	dev	err [%]
431	387	O0110a		-	-	298	1.0	775.8	775.9	0.1	0.0	-	-	-	-
432	388	O0111a		-	-	298	1.0	773.6	773.3	-0.3	-0.0	-	-	-	-
433	389	O0112a		-	-	298	1.0	788.6	792.2	3.6	0.5	-	-	-	-
434	390	O0113a		-	-	298	1.0	782.3	781.6	-0.7	-0.1	-	-	-	-
435	391	O0114a		-	-	298	1.0	779.8	778.9	-0.9	-0.1	-	-	-	-
436	392	O0115a		-	-	298	1.0	778.8	779.0	0.2	0.0	53.2	54.2	1.0	1.9
437	393	O0116a		460.1	-	298	1.0	779.2	779.4	0.2	0.0	-	-	-	-
438	394	O0201a		444.4	-	298	1.0	816.8	788.6	-28.2	-3.5	-	-	-	-
439	395	O0202a		415.4	-	298	1.0	829.0	804.0	-25.1	-3.0	57.8	52.8	-5.0	-8.6
440	396	O0203a		-	-	298	1.0	840.9	841.7	0.8	0.1	-	-	-	-
441	397	O0204a		-	-	293	1.0	840.9	829.6	-11.3	-1.3	-	-	-	-
442	398	O0205a		476.4	715.5	298	1.0	833.7	826.9	-6.8	-0.8	58.8	59.1	0.3	0.5
443	399	O0301a		-	-	288	1.0	886.9	884.3	-2.6	-0.3	-	-	-	-
444	400	A1101a		254.1	415.2	251	1.36	805.2	811.6	6.5	0.8	-	-	-	-
445	400	A1101b		254.1	415.2	293	1.0	814.0	735.0	-79.0	-9.7	-	-	-	-
446	401	A2101a	×	293.6	466.0	298	1.0	772.0	923.8	151.8	19.7	26.9	27.2	0.3	1.1
447	402	A3101a		321.1	504.4	298	1.0	791.2	780.0	-11.2	-1.4	29.6	28.2	-1.4	-4.9
448	403	A4101a		337.2	540.4	298	1.0	796.6	776.6	-20.0	-2.5	32.3	30.9	-1.4	-4.2
449	404	A4102a		347.9	537.2	298	1.0	796.6	796.9	0.3	0.0	33.7	32.9	-0.8	-2.5
450	405	A5101a		347.1	570.0	298	0.14	783.1	784.6	1.5	0.2	-	-	-	-
451	406	A5102a		365.1	570.0	298	0.06	804.1	798.4	-5.7	-0.7	-	-	-	-
452	407	A5103a		365.8	570.0	298	1.0	794.2	801.1	6.9	0.9	-	-	-	-
453	408	A5104a		376.1	566.1	298	1.0	806.2	808.5	2.2	0.3	38.1	37.7	-0.4	-1.1
454	409	A6101a		377.1	596.5	298	0.03	801.0	813.4	12.4	1.6	-	-	-	-
455	410	A6102a		386.1	596.5	298	1.0	809.7	812.5	2.8	0.3	-	-	-	-
456	411	A6103a		389.9	596.5	298	0.02	814.1	813.5	-0.6	-0.1	-	-	-	-
457	412	A6104a		390.1	596.5	298	0.02	808.0	807.2	-0.8	-0.1	-	-	-	-
458	413	A6105a		395.1	596.5	299	0.01	806.6	817.7	11.0	1.4	-	-	-	-
459	414	A6106a		401.4	591.0	298	1.01	833.1	816.2	-16.9	-2.0	42.3	42.4	0.1	0.2
460	415	A6201a		435.2	-	292	1.0	1003.0	991.1	-11.9	-1.2	-	-	-	-
461	416	A7101a		-	-	298	1.0	829.5	824.0	-5.5	-0.7	-	-	-	-
462	417	A7102a		-	-	298	1.0	823.1	831.5	8.4	1.0	-	-	-	-
463	417	A7102b		-	-	334	1.0	-	-	-	-	42.4	42.9	0.5	1.2
464	418	A7103a	vap	416.1	620.4	298	1.0	814.3	-	-	-	0.0	-	-	-
465	418	A7103b		416.1	620.4	329	1.0	-	-	-	-	42.8	43.8	1.0	2.4
466	419	A7104a		-	620.4	298	1.0	824.0	828.1	4.1	0.5	-	-	-	-
467	420	A7105a		-	620.4	293	1.0	820.6	823.0	2.4	0.3	-	-	-	-
468	421	A7106a		425.9	616.8	298	1.0	813.3	822.2	8.9	1.1	48.0	47.1	-0.9	-1.9
469	422	A8101a		-	-	293	1.0	847.6	827.2	-20.4	-2.4	-	-	-	-
470	423	A8102a		-	-	293	1.0	847.6	829.2	-18.4	-2.2	-	-	-	-
471	424	A8103a		433.8	642.4	298	1.0	815.2	824.2	9.1	1.1	-	-	-	-
472	425	A8104a		447.1	638.9	298	1.01	821.1	826.5	5.4	0.7	51.0	51.8	0.8	1.5
473	426	A9101a		-	-	298	1.0	827.0	840.0	13.0	1.6	-	-	-	-
474	427	A9102a		-	-	291	1.0	884.3	842.6	-41.7	-4.7	-	-	-	-
475	428	A9103a		-	-	293	1.0	848.3	836.4	-11.9	-1.4	-	-	-	-
476	429	A9104a		-	-	293	1.0	842.3	830.1	-12.2	-1.5	-	-	-	-
477	430	A9105a		468.1	662.7	298	1.01	831.0	830.3	-0.7	-0.1	55.3	56.4	1.1	2.0
478	431	A0101a		-	-	293	1.0	843.0	838.5	-4.5	-0.5	-	-	-	-

Table S.13 – Comparison of experimental and simulated properties using LB combination rules (continued).

n_{sim}	n_{iso}	Code	Outlier	T_m [K]	T_b [K]	T [K]	P [bar]	ρ_{liq}[kg·m^{-3}] exp	sim	dev	err [%]	ΔH_{vap}[kJ·mol^{-1}] exp	sim	dev	err [%]
479	432	A0102a		403.1	656.6	298	1.0	813.4	831.6	18.2	2.2	-	-	-	-
480	433	A0103a		488.1	674.2	298	1.0	824.9	833.4	8.5	1.0	59.5	61.1	1.6	2.7
481	434	K3101a		329.4	508.2	298	1.0	784.4	773.4	-10.9	-1.4	31.3	26.5	-4.8	-15.2
482	435	K4101a		352.8	535.5	298	1.0	799.9	787.7	-12.2	-1.5	34.5	30.9	-3.6	-10.5
483	436	K5101a		367.6	553.4	298	1.0	809.4	791.9	-17.5	-2.2	36.8	33.9	-2.9	-7.8
484	437	K5102a		375.1	560.9	298	1.0	809.6	795.3	-14.3	-1.8	38.5	35.0	-3.5	-9.1
485	438	K5103a		375.5	561.1	298	1.0	801.8	798.5	-3.2	-0.4	38.3	35.6	-2.7	-7.0
486	439	K6101a		379.2	-	298	1.0	804.3	810.9	6.6	0.8	38.3	36.5	-1.8	-4.7
487	440	K6102a		386.6	587.5	298	1.0	806.6	796.9	-9.7	-1.2	39.8	37.9	-1.9	-4.8
488	441	K6103a		390.6	587.5	298	1.0	808.3	809.3	1.0	0.1	39.8	38.7	-1.1	-2.7
489	442	K6104a		389.6	574.6	298	1.0	796.3	801.2	4.9	0.6	41.0	38.6	-2.4	-5.9
490	443	K6105a		396.6	582.8	298	1.0	811.1	802.8	-8.3	-1.0	40.6	39.6	-1.0	-2.4
491	444	K6106a		400.9	587.6	298	1.0	807.1	809.2	2.1	0.3	42.2	40.6	-1.6	-3.9
492	445	K6201a		433.1	629.0	293	1.0	959.0	956.6	-2.4	-0.2	-	-	-	-
493	446	K7101a		403.8	611.4	294	1.0	823.0	838.3	15.3	1.9	-	-	-	-
494	447	K7102a		398.1	611.4	298	1.0	808.4	812.8	4.4	0.5	42.3	40.5	-1.8	-4.4
495	448	K7103a		398.1	611.4	298	1.0	801.2	813.8	12.6	1.6	-	-	-	-
496	449	K7104a		397.6	611.4	298	1.0	799.7	796.8	-2.9	-0.4	41.5	40.6	-0.9	-2.2
497	450	K7105a		405.1	611.4	293	1.0	827.3	825.0	-2.3	-0.3	-	-	-	-
498	451	K7106a		409.1	611.4	298	1.0	824.0	811.2	-12.8	-1.5	-	-	-	-
499	452	K7107a		406.1	611.4	302	0.01	809.5	799.9	-9.5	-1.2	-	-	-	-
500	453	K7108a		411.1	611.4	295	1.0	815.3	821.8	6.5	0.8	-	-	-	-
501	454	K7109a		413.1	611.4	298	1.0	828.0	816.6	-11.4	-1.4	-	-	-	-
502	455	K7110a		409.1	611.4	293	1.0	812.0	808.9	-3.1	-0.4	-	-	-	-
503	456	K7111a		412.1	611.4	298	1.0	808.5	817.2	8.7	1.1	-	-	-	-
504	457	K7112a		417.9	611.4	293	1.0	811.6	817.9	6.3	0.8	-	-	-	-
505	458	K7113a		417.1	602.0	298	1.0	811.6	808.9	-2.7	-0.3	-	-	-	-
506	459	K7114a		420.6	606.6	298	1.0	814.6	812.1	-2.6	-0.3	-	-	-	-
507	460	K7115a		424.1	611.4	298	1.0	811.6	816.3	4.7	0.6	46.1	45.4	-0.7	-1.5
508	461	K7201a		451.6	645.0	292	1.0	953.1	950.2	-2.9	-0.3	-	-	-	-
509	462	K7202a		449.1	645.0	293	1.0	945.0	941.3	-3.7	-0.4	-	-	-	-
510	463	K8101a		-	-	293	1.0	839.5	857.5	18.0	2.1	-	-	-	-
511	464	K8102a		408.2	633.4	298	1.0	802.3	814.7	12.4	1.5	43.3	43.1	-0.2	-0.4
512	465	K8103a		-	-	293	1.0	826.0	843.4	17.4	2.1	-	-	-	-
513	466	K8104a		426.6	633.4	293	1.0	838.9	856.1	17.2	2.0	-	-	-	-
514	467	K8105a		421.1	633.4	293	1.0	829.8	837.9	8.1	1.0	-	-	-	-
515	468	K8106a		420.1	633.4	293	1.0	825.7	842.9	17.2	2.1	-	-	-	-
516	469	K8107a		419.1	633.4	298	1.0	810.5	818.4	7.9	1.0	-	-	-	-
517	470	K8108a		-	-	293	1.0	829.0	839.5	10.5	1.3	-	-	-	-
518	471	K8109a		-	-	293	1.0	812.0	820.2	8.2	1.0	-	-	-	-
519	472	K8110a		420.6	633.4	293	1.0	812.1	809.6	-2.5	-0.3	-	-	-	-
520	473	K8111a		431.1	633.4	295	1.0	829.5	835.8	6.3	0.8	-	-	-	-
521	474	K8112a		426.1	633.4	298	1.0	817.0	816.0	-1.0	-0.1	-	-	-	-
522	475	K8113a		-	633.4	298	1.0	820.0	817.1	-2.9	-0.4	-	-	-	-
523	476	K8114a		431.1	633.4	293	1.0	817.5	815.3	-2.2	-0.3	-	-	-	-
524	477	K8115a		435.1	633.4	297	1.0	829.0	819.6	-9.4	-1.1	-	-	-	-
525	478	K8116a		427.1	633.4	295	1.0	813.0	812.2	-0.8	-0.1	-	-	-	-
526	479	K8117a		437.1	633.4	293	1.0	830.4	819.5	-10.9	-1.3	-	-	-	-

Table S.13 – Comparison of experimental and simulated properties using LB combination rules (continued).

n_{sim}	n_{iso}	Code	Outlier	T_m [K]	T_b [K]	T [K]	P [bar]	ρ_{liq}[kg·m^{-3}] exp	sim	dev	err [%]	ΔH_{vap}[kJ·mol^{-1}] exp	sim	dev	err [%]
527	480	K8118a	vap	437.0	633.4	298	1.0	811.0	-	-	-	0.0	-	-	-
528	481	K8119a		440.1	633.4	298	1.0	810.0	819.0	9.0	1.1	-	-	-	-
529	482	K8120a		436.1	633.4	298	1.0	814.7	816.2	1.5	0.2	-	-	-	-
530	483	K8121a		440.6	633.4	298	1.0	822.0	817.8	-4.2	-0.5	-	-	-	-
531	484	K8122a		445.8	632.7	298	1.0	815.2	821.8	6.5	0.8	51.8	50.2	-1.6	-3.1
532	485	K9101a		425.1	653.7	298	1.0	820.2	837.0	16.8	2.0	45.4	46.1	0.7	1.4
533	486	K9102a		-	-	296	1.0	812.0	820.9	8.9	1.1	-	-	-	-
534	487	K9103a		-	-	293	1.0	816.8	827.8	11.0	1.4	-	-	-	-
535	488	K9104a		-	-	298	1.0	809.0	820.6	11.6	1.4	-	-	-	-
536	489	K9105a		435.1	653.7	287	1.0	826.0	829.0	3.0	0.4	-	-	-	-
537	490	K9106a		445.0	653.7	298	1.0	813.5	814.8	1.3	0.2	-	-	-	-
538	491	K9107a		441.4	653.7	298	1.0	802.4	811.0	8.6	1.1	50.9	49.8	-1.1	-2.1
539	492	K9108a		-	-	293	1.0	833.5	840.4	6.9	0.8	-	-	-	-
540	493	K9109a		-	-	298	1.0	817.0	823.8	6.8	0.8	-	-	-	-
541	494	K9110a		447.1	653.7	287	1.0	829.0	829.6	0.6	0.1	-	-	-	-
542	495	K9111a		456.6	653.7	298	1.0	820.0	822.6	2.6	0.3	-	-	-	-
543	496	K9112a		456.0	653.7	293	1.0	821.2	820.4	-0.8	-0.1	-	-	-	-
544	497	K9113a		-	-	293	1.0	824.6	832.0	7.4	0.9	-	-	-	-
545	498	K9114a		456.6	653.7	300	1.0	832.0	825.2	-6.8	-0.8	-	-	-	-
546	499	K9115a		-	653.7	298	1.0	815.0	816.6	1.6	0.2	-	-	-	-
547	500	K9116a		451.1	653.7	293	1.0	823.9	823.2	-0.7	-0.1	-	-	-	-
548	501	K9117a		-	653.7	298	1.0	822.0	829.9	7.9	1.0	-	-	-	-
549	502	K9118a		461.6	640.0	298	1.0	817.8	821.7	3.9	0.5	53.3	54.0	0.7	1.3
550	503	K9119a		460.6	653.7	298	1.0	819.7	820.8	1.1	0.1	-	-	-	-
551	504	K9120a		463.1	653.7	298	1.0	820.4	822.7	2.2	0.3	55.6	54.1	-1.5	-2.7
552	505	K9121a		467.1	652.5	298	1.0	817.8	826.4	8.7	1.1	-	-	-	-
553	506	K0101a		-	-	298	1.0	816.9	828.8	11.9	1.5	48.8	49.7	0.9	1.8
554	507	K0102a		-	-	298	1.0	825.2	833.1	7.9	1.0	-	-	-	-
555	508	K0103a		-	-	289	1.0	825.2	840.2	15.0	1.8	-	-	-	-
556	509	K0104a		-	-	293	1.0	832.0	831.7	-0.3	-0.0	-	-	-	-
557	510	K0105a		-	-	293	1.0	814.3	828.9	14.6	1.8	-	-	-	-
558	511	K0106a		473.0	672.7	293	1.0	822.6	825.3	2.7	0.3	-	-	-	-
559	512	K0107a		-	-	298	1.0	818.0	820.6	2.6	0.3	-	-	-	-
560	513	K0108a		476.6	672.7	293	1.0	821.3	827.8	6.5	0.8	-	-	-	-
561	514	K0109a		-	-	293	1.0	838.4	836.7	-1.7	-0.2	-	-	-	-
562	515	K0110a		477.1	672.7	298	1.0	820.5	825.5	5.0	0.6	-	-	-	-
563	516	K0111a		479.6	672.7	294	1.0	822.0	828.2	6.2	0.8	-	-	-	-
564	517	K0112a		476.1	672.7	298	1.0	821.9	826.5	4.6	0.6	-	-	-	-
565	518	K0113a		483.4	672.7	298	1.0	820.1	830.1	10.0	1.2	60.9	59.7	-1.2	-1.9
566	519	E2201a	×	304.9	487.2	298	1.0	966.8	1108.9	142.1	14.7	28.4	30.2	1.8	6.4
567	520	E3201a		327.5	508.4	298	1.0	915.9	886.9	-28.9	-3.2	31.5	30.5	-1.0	-3.1
568	521	E3202a		330.1	506.6	298	1.01	927.5	898.1	-29.4	-3.2	32.3	31.2	-1.1	-3.3
569	522	E4201a		341.2	514.9	298	0.17	870.2	859.3	-10.9	-1.3	-	-	-	-
570	523	E4202a		354.0	538.0	298	1.0	899.9	881.7	-18.2	-2.0	36.6	35.1	-1.5	-4.2
571	524	E4203a		352.6	530.6	298	1.0	909.1	884.8	-24.3	-2.7	35.7	35.2	-0.5	-1.4
572	525	E4204a		350.2	523.3	298	1.0	894.3	874.5	-19.7	-2.2	35.1	34.7	-0.4	-1.2
573	526	E4401a		447.1	-	273	1.0	1193.0	1168.4	-24.6	-2.1	-	-	-	-
574	527	E5201a		355.9	541.0	298	0.09	871.9	858.1	-13.8	-1.6	-	-	-	-

Table S.13 – Comparison of experimental and simulated properties using LB combination rules (continued).

n_{sim}	n_{iso}	Code	Outlier	T_m [K]	T_b [K]	T [K]	P [bar]	ρ_{liq}[kg·m^{-3}] exp	sim	dev	err [%]	ΔH_{vap}[kJ·mol^{-1}] exp	sim	dev	err [%]
575	528	E5202a		371.2	551.4	298	1.0	875.7	867.7	-8.0	-0.9	-	-	-	-
576	529	E5203a		363.6	541.0	298	0.06	878.6	870.6	-7.9	-0.9	-	-	-	-
577	530	E5204a		379.2	541.0	298	1.0	887.6	885.6	-2.1	-0.2	40.5	40.2	-0.3	-0.8
578	531	E5205a		365.6	540.7	298	1.0	883.3	866.2	-17.1	-1.9	37.3	37.4	0.1	0.3
579	532	E5206a		361.6	532.0	298	1.0	869.0	854.3	-14.7	-1.7	37.0	37.6	0.6	1.7
580	533	E5207a		375.9	554.5	298	1.01	892.5	879.6	-12.9	-1.4	39.8	39.3	-0.5	-1.2
581	534	E5208a		372.2	546.0	298	1.0	884.0	866.0	-18.1	-2.0	39.3	38.5	-0.8	-2.0
582	535	E5209a		374.6	549.7	298	1.01	882.8	871.1	-11.7	-1.3	39.1	39.1	0.0	0.0
583	536	E5401a		454.6	-	298	1.0	1146.7	1112.0	-34.7	-3.0	57.5	54.7	-2.8	-4.9
584	537	E5402a		437.6	-	298	1.0	1135.5	1107.4	-28.1	-2.5	56.4	55.4	-1.0	-1.8
585	538	E6201a		385.6	564.6	298	0.02	883.7	877.7	-6.0	-0.7	-	-	-	-
586	539	E6202a		397.1	564.6	298	1.0	877.0	883.1	6.1	0.7	-	-	-	-
587	540	E6203a		406.6	576.0	298	1.0	880.4	884.7	4.3	0.5	45.2	45.1	-0.1	-0.3
588	541	E6204a		374.2	-	293	1.0	850.0	871.0	21.0	2.5	38.8	39.5	0.7	1.8
589	542	E6205a		369.1	564.6	298	1.0	861.6	848.8	-12.8	-1.5	38.0	38.0	0.0	0.0
590	543	E6206a		-	-	293	1.0	884.7	877.8	-6.9	-0.8	-	-	-	-
591	544	E6207a		383.0	553.1	298	1.0	864.0	852.2	-11.8	-1.4	39.8	40.8	1.0	2.6
592	545	E6208a		389.6	564.6	298	1.0	875.9	871.3	-4.6	-0.5	-	-	-	-
593	546	E6209a		383.1	553.0	298	1.0	860.1	848.8	-11.3	-1.3	-	-	-	-
594	547	E6210a		389.8	560.8	298	1.0	866.3	860.6	-5.7	-0.7	39.5	41.9	2.3	5.9
595	548	E6211a		385.1	564.6	298	1.0	866.0	861.3	-4.7	-0.5	-	-	-	-
596	549	E6212a		400.6	564.6	298	1.0	885.2	879.0	-6.2	-0.7	43.7	44.4	0.7	1.7
597	550	E6213a		394.6	571.0	298	1.0	873.8	863.8	-10.0	-1.1	42.0	43.0	1.0	2.4
598	551	E6214a		395.6	568.6	298	1.0	876.4	862.7	-13.7	-1.6	43.2	42.7	-0.5	-1.2
599	552	E6215a		399.1	575.4	298	1.0	876.4	871.9	-4.5	-0.5	42.7	43.9	1.2	2.7
600	553	E6401a		447.1	-	298	1.0	1093.6	1064.8	-28.9	-2.6	-	-	-	-
601	553	E6401b		447.1	-	293	1.0	-	-	-	-	57.8	56.0	-1.8	-3.2
602	554	E6402a		442.1	635.0	298	1.0	1070.0	1057.2	-12.8	-1.2	59.0	59.4	0.4	0.7
603	555	E6403a		469.6	657.0	298	1.0	1114.0	1081.5	-32.5	-2.9	60.9	59.7	-1.2	-2.0
604	556	E6404a		463.6	653.0	298	1.0	1098.7	1066.5	-32.2	-2.9	61.0	58.6	-2.4	-4.0
605	557	E7201a		428.6	586.3	298	1.0	874.6	883.5	8.9	1.0	50.0	49.8	-0.2	-0.3
606	558	E7202a		391.6	-	298	1.0	849.5	852.6	3.1	0.4	41.3	42.4	1.1	2.7
607	559	E7203a		-	-	293	1.0	870.0	878.8	8.8	1.0	43.9	44.5	0.6	1.3
608	560	E7204a		-	586.3	298	1.0	853.9	862.5	8.6	1.0	-	-	-	-
609	561	E7205a		-	-	298	1.0	872.5	864.0	-8.5	-1.0	40.3	42.7	2.4	5.9
610	562	E7206a		414.6	-	293	1.0	864.7	849.0	-15.7	-1.8	-	-	-	-
611	563	E7207a		396.1	-	294	1.0	846.7	841.1	-5.7	-0.7	-	-	-	-
612	564	E7208a		401.6	586.3	298	1.0	866.0	862.8	-3.2	-0.4	-	-	-	-
613	565	E7209a		409.1	586.3	293	1.0	879.7	879.3	-0.4	-0.0	-	-	-	-
614	566	E7210a		-	-	293	1.0	876.5	876.9	0.4	0.0	-	-	-	-
615	567	E7211a		-	-	293	1.0	867.8	863.9	-3.9	-0.5	44.7	45.5	0.8	1.9
616	568	E7212a		407.1	-	298	1.0	859.5	849.9	-9.5	-1.1	-	-	-	-
617	569	E7213a		408.1	-	298	1.0	861.2	858.3	-2.9	-0.3	-	-	-	-
618	570	E7214a		409.1	592.0	298	1.0	867.5	853.4	-14.1	-1.6	-	-	-	-
619	571	E7215a		406.1	-	298	1.0	861.2	854.6	-6.6	-0.8	-	-	-	-
620	572	E7216a		402.1	-	298	1.0	853.9	848.9	-5.0	-0.6	-	-	-	-
621	573	E7217a		413.1	586.3	293	1.0	867.8	874.2	6.4	0.7	-	-	-	-
622	574	E7218a		405.1	586.3	303	0.01	861.5	859.2	-2.3	-0.3	-	-	-	-

Table S.13 – Comparison of experimental and simulated properties using LB combination rules (continued).

n_{sim}	n_{iso}	Code	Outlier	T_m [K]	T_b [K]	T [K]	P [bar]	ρ_{liq}[kg·m^{-3}] exp	sim	dev	err [%]	ΔH_{vap}[kJ·mol^{-1}] exp	sim	dev	err [%]
623	575	E7219a		414.8	586.1	299	1.0	864.8	868.5	3.7	0.4	46.4	47.6	1.2	2.7
624	576	E7220a		406.1	586.3	298	1.0	863.1	861.4	-1.7	-0.2	-	-	-	-
625	577	E7221a		422.6	586.3	298	1.0	880.4	878.0	-2.4	-0.3	47.7	49.0	1.3	2.8
626	578	E7222a		419.2	586.3	298	1.01	869.4	866.1	-3.4	-0.4	47.0	47.8	0.8	1.7
627	579	E7223a		416.4	593.7	298	1.0	868.2	861.2	-7.0	-0.8	-	-	-	-
628	580	E7224a		419.8	594.6	298	1.0	871.5	863.9	-7.6	-0.9	48.5	47.5	-1.0	-2.0
629	581	E7225a		422.1	599.9	298	1.0	872.2	871.8	-0.5	-0.1	48.6	48.9	0.3	0.6
630	582	E7401a		-	-	293	1.0	-	-	-	-	55.6	56.3	0.7	1.3
631	583	E7402a		-	-	298	1.0	1061.4	1044.3	-17.1	-1.6	-	-	-	-
632	584	E7403a		469.1	-	298	1.0	1076.0	1048.6	-27.4	-2.5	-	-	-	-
633	585	E7404a		463.6	-	293	1.0	1059.0	1033.4	-25.6	-2.4	-	-	-	-
634	586	E7405a		472.1	653.0	298	1.0	1049.8	1027.6	-22.2	-2.1	58.7	59.8	1.1	1.9
635	587	E7406a		481.4	-	293	1.0	1076.0	1046.6	-29.4	-2.7	-	-	-	-
636	588	E7407a		487.1	-	293	1.01	1087.7	1061.7	-26.0	-2.4	65.7	65.2	-0.5	-0.7
637	589	E7408a		482.6	-	293	1.0	1054.7	1047.2	-7.5	-0.7	-	-	-	-
638	590	E8201a		451.2	606.5	302	1.0	869.0	878.4	9.4	1.1	53.8	54.2	0.5	0.8
639	591	E8202a		-	-	293	1.0	830.0	843.9	13.9	1.7	-	-	-	-
640	592	E8203a		-	-	298	1.0	874.9	890.4	15.5	1.8	-	-	-	-
641	593	E8204a		-	-	277	1.0	883.0	885.9	2.9	0.3	-	-	-	-
642	594	E8205a		-	-	293	1.0	860.4	867.0	6.6	0.8	-	-	-	-
643	595	E8206a		-	-	293	1.0	867.9	881.5	13.6	1.6	-	-	-	-
644	596	E8207a		437.4	606.5	308	0.01	874.7	849.9	-24.8	-2.8	-	-	-	-
645	597	E8208a		426.1	606.5	320	0.01	862.6	846.3	-16.3	-1.9	-	-	-	-
646	598	E8209a		-	-	298	1.0	866.0	863.7	-2.3	-0.3	-	-	-	-
647	599	E8210a		421.1	-	293	1.0	847.0	846.8	-0.2	-0.0	48.5	47.7	-0.8	-1.7
648	600	E8211a		-	606.5	289	1.0	870.0	852.0	-18.0	-2.1	-	-	-	-
649	601	E8212a		420.1	-	298	1.0	846.1	845.0	-1.1	-0.1	-	-	-	-
650	602	E8213a		420.6	-	298	1.0	880.5	859.8	-20.7	-2.4	-	-	-	-
651	603	E8214a		-	-	293	1.0	875.0	878.1	3.1	0.4	-	-	-	-
652	604	E8215a		-	-	293	1.0	863.3	866.2	2.9	0.3	-	-	-	-
653	605	E8216a		428.3	-	293	1.0	876.5	864.5	-12.0	-1.4	48.4	50.4	2.0	4.2
654	605	E8216b		428.3	-	298	1.0	-	-	-	-	48.4	50.0	1.6	3.4
655	606	E8217a		-	-	293	1.0	869.7	860.7	-9.0	-1.0	-	-	-	-
656	607	E8218a		-	-	298	1.0	857.4	853.1	-4.3	-0.5	-	-	-	-
657	608	E8219a		-	-	293	1.0	878.0	870.7	-7.3	-0.8	-	-	-	-
658	609	E8220a		429.1	-	298	1.0	857.5	856.3	-1.2	-0.1	-	-	-	-
659	610	E8221a		436.1	606.5	293	1.0	870.5	868.7	-1.8	-0.2	-	-	-	-
660	611	E8222a		430.1	611.0	298	1.0	860.6	853.5	-7.1	-0.8	-	-	-	-
661	612	E8223a		446.1	606.5	298	1.0	865.0	863.1	-1.9	-0.2	-	-	-	-
662	613	E8224a		-	-	298	1.0	863.2	853.7	-9.6	-1.1	-	-	-	-
663	614	E8225a		440.2	606.5	298	1.0	861.3	855.6	-5.7	-0.7	-	-	-	-
664	615	E8226a		436.4	606.5	293	1.0	857.9	856.3	-1.6	-0.2	-	-	-	-
665	616	E8227a		435.6	606.5	293	1.0	879.0	879.9	0.9	0.1	-	-	-	-
666	617	E8228a		436.1	606.5	298	1.0	869.1	870.1	1.0	0.1	-	-	-	-
667	618	E8229a		-	606.5	298	1.0	859.9	863.5	3.6	0.4	-	-	-	-
668	619	E8230a		447.1	606.5	298	1.01	875.9	877.0	1.1	0.1	51.6	53.9	2.3	4.5
669	620	E8231a		440.1	606.5	298	1.0	866.7	865.9	-0.8	-0.1	50.6	52.6	2.0	3.9
670	621	E8232a		440.6	606.5	298	1.0	865.8	862.5	-3.3	-0.4	-	-	-	-

Table S.13 – Comparison of experimental and simulated properties using LB combination rules (continued).

n_{sim}	n_{iso}	Code	Outlier	T_m [K]	T_b [K]	T [K]	P [bar]	ρ_{liq}[kg·m^{-3}] exp	sim	dev	err [%]	ΔH_{vap}[kJ·mol^{-1}] exp	sim	dev	err [%]
671	622	E8233a		438.1	606.5	298	1.01	869.1	862.8	-6.3	-0.7	-	-	-	-
672	623	E8234a		441.9	606.5	298	1.0	868.1	865.3	-2.8	-0.3	52.2	52.4	0.2	0.3
673	624	E8235a		444.6	606.5	298	1.0	868.6	871.6	3.0	0.3	51.9	53.5	1.6	3.1
674	625	E8401a		-	-	298	1.0	1036.9	1026.2	-10.7	-1.0	-	-	-	-
675	626	E8402a		474.1	-	298	1.0	1017.4	998.7	-18.7	-1.8	-	-	-	-
676	627	E8403a		489.6	663.0	298	1.0	1035.3	1012.3	-23.0	-2.2	64.5	65.2	0.7	1.0
677	628	E8404a		495.1	-	298	1.0	1057.6	1038.7	-18.8	-1.8	69.0	69.2	0.2	0.3
678	629	E8405a		484.1	-	293	1.0	1042.0	1014.6	-27.4	-2.6	67.6	64.8	-2.8	-4.2
679	630	E8406a		502.1	-	293	1.0	1046.0	1030.1	-15.9	-1.5	-	-	-	-
680	631	E9201a		471.9	625.3	298	1.0	871.0	880.2	9.2	1.1	58.2	59.3	1.1	1.9
681	632	E9202a		-	-	298	1.0	-	-	-	-	48.4	51.8	3.4	6.9
682	633	E9203a		-	-	298	1.0	-	-	-	-	47.8	48.2	0.4	0.9
683	634	E9204a		-	-	298	1.0	-	-	-	-	50.4	51.1	0.7	1.4
684	635	E9205a		460.3	625.3	298	1.0	-	-	-	-	50.3	50.3	0.0	0.0
685	636	E9206a		-	-	301	1.0	855.0	858.4	3.4	0.4	-	-	-	-
686	637	E9207a		442.1	-	293	1.0	862.7	857.6	-5.1	-0.6	51.7	53.8	2.1	4.1
687	638	E9208a		-	-	298	1.0	869.0	873.3	4.3	0.5	-	-	-	-
688	639	E9209a		442.1	-	298	1.0	861.7	849.8	-11.9	-1.4	-	-	-	-
689	640	E9210a		-	-	293	1.0	848.2	853.6	5.4	0.6	-	-	-	-
690	641	E9211a		-	-	293	1.0	871.0	876.8	5.8	0.7	-	-	-	-
691	642	E9212a		-	-	293	1.0	857.2	866.5	9.3	1.1	-	-	-	-
692	643	E9213a		-	-	293	1.0	868.8	863.4	-5.4	-0.6	-	-	-	-
693	644	E9214a		-	-	293	1.0	866.9	861.4	-5.5	-0.6	-	-	-	-
694	645	E9215a		452.1	-	293	1.0	862.0	862.7	0.7	0.1	50.6	54.3	3.7	7.3
695	646	E9216a		-	625.3	293	1.0	872.1	858.9	-13.2	-1.5	-	-	-	-
696	647	E9217a		450.1	625.3	293	1.0	867.9	869.6	1.7	0.2	-	-	-	-
697	648	E9218a		456.9	-	298	1.0	856.7	858.0	1.3	0.2	-	-	-	-
698	649	E9219a		453.1	625.3	293	1.0	870.8	875.2	4.4	0.5	-	-	-	-
699	650	E9220a		452.1	625.3	298	1.0	860.3	861.5	1.2	0.1	-	-	-	-
700	651	E9221a		456.1	625.3	298	1.0	853.6	855.5	2.0	0.2	-	-	-	-
701	652	E9222a		455.1	625.3	298	1.0	864.7	855.2	-9.5	-1.1	-	-	-	-
702	653	E9223a		-	-	298	1.0	854.9	856.1	1.3	0.1	-	-	-	-
703	654	E9224a		-	-	298	1.0	852.5	853.5	1.0	0.1	-	-	-	-
704	655	E9225a		-	625.3	298	1.0	857.0	863.4	6.4	0.7	-	-	-	-
705	656	E9226a		466.1	625.3	298	1.0	873.1	876.4	3.3	0.4	56.4	58.5	2.1	3.8
706	657	E9227a		460.1	625.3	298	1.0	864.7	866.2	1.5	0.2	-	-	-	-
707	658	E9228a		460.1	625.3	298	1.0	863.0	862.6	-0.4	-0.0	-	-	-	-
708	659	E9229a		459.1	625.3	298	1.0	863.4	863.8	0.4	0.0	-	-	-	-
709	660	E9230a		458.1	625.3	298	1.0	861.9	863.4	1.5	0.2	53.6	56.7	3.1	5.7
710	661	E9231a		463.1	625.3	298	1.0	865.4	864.9	-0.5	-0.1	57.1	56.9	-0.2	-0.3
711	662	E9232a		465.6	625.3	298	1.0	866.4	871.4	5.0	0.6	56.9	58.3	1.4	2.5
712	663	E9401a		509.2	-	298	1.0	-	-	-	-	63.9	63.8	-0.1	-0.1
713	664	E9402a		481.1	-	298	1.0	1000.7	987.6	-13.2	-1.3	-	-	-	-
714	665	E9403a		502.1	-	298	1.0	-	-	-	-	66.2	66.8	0.6	0.9
715	666	E9404a		509.6	-	293	1.0	1022.0	1004.6	-17.4	-1.7	67.0	70.9	3.9	5.9
716	667	E9405a		509.2	-	293	1.0	1039.1	1026.3	-12.8	-1.2	73.5	73.8	0.3	0.4
717	668	E9406a		514.1	-	293	1.0	1029.6	1015.4	-14.2	-1.4	-	-	-	-
718	669	E0201a		485.7	643.0	293	1.0	867.0	881.0	14.0	1.6	-	-	-	-

Table S.13 – Comparison of experimental and simulated properties using LB combination rules (continued).

n_{sim}	n_{iso}	Code	Outlier	T_m [K]	T_b [K]	T [K]	P [bar]	ρ_{liq}[kg·m^{-3}] exp	sim	dev	err [%]	ΔH_{vap}[kJ·mol^{-1}] exp	sim	dev	err [%]
719	670	E0202a		-	-	293	1.0	843.1	850.6	7.5	0.9	48.9	51.3	2.4	5.0
720	671	E0203a		-	-	298	1.0	-	-	-	-	48.0	49.8	1.8	3.8
721	672	E0204a		461.1	-	273	1.0	872.9	873.0	0.1	0.0	-	-	-	-
722	673	E0205a		-	-	289	1.0	883.7	895.3	11.6	1.3	-	-	-	-
723	674	E0206a		-	-	293	1.0	856.8	866.2	9.4	1.1	-	-	-	-
724	675	E0207a		463.1	-	298	1.0	854.1	858.1	4.0	0.5	-	-	-	-
725	676	E0208a		460.1	-	293	1.0	847.4	864.4	17.0	2.0	-	-	-	-
726	677	E0209a		-	-	293	1.0	865.9	866.7	0.8	0.1	-	-	-	-
727	678	E0210a		-	-	298	1.0	864.4	873.4	9.0	1.0	-	-	-	-
728	679	E0211a		473.7	643.0	298	1.0	858.6	862.9	4.3	0.5	-	-	-	-
729	680	E0212a		-	-	277	1.0	875.9	888.2	12.3	1.4	-	-	-	-
730	681	E0213a		-	-	293	1.0	870.0	859.9	-10.1	-1.2	-	-	-	-
731	682	E0214a		-	643.0	293	1.0	872.6	870.7	-1.9	-0.2	-	-	-	-
732	683	E0215a		466.1	643.0	293	1.0	858.0	867.0	9.0	1.0	-	-	-	-
733	684	E0216a		-	-	298	1.0	857.5	857.0	-0.5	-0.1	-	-	-	-
734	685	E0217a		471.8	642.4	298	1.0	868.8	874.0	5.2	0.6	-	-	-	-
735	686	E0218a		-	643.0	298	1.0	858.1	864.3	6.2	0.7	-	-	-	-
736	687	E0219a		486.6	643.0	298	1.0	870.9	875.6	4.7	0.5	61.6	63.4	1.8	2.9
737	688	E0220a		481.6	643.0	298	1.0	862.9	866.4	3.5	0.4	59.5	62.1	2.6	4.3
738	689	E0221a		481.1	643.0	298	1.0	861.6	863.4	1.8	0.2	-	-	-	-
739	690	E0222a		481.1	643.0	298	1.0	862.3	863.8	1.5	0.2	-	-	-	-
740	691	E0223a		476.9	643.0	298	1.0	860.2	864.3	4.1	0.5	-	-	-	-
741	692	E0224a		479.1	643.0	298	1.01	851.0	863.7	12.7	1.5	-	-	-	-
742	693	E0225a		483.1	643.0	298	1.0	864.0	865.4	1.5	0.2	-	-	-	-
743	694	E0226a		484.4	643.0	298	1.0	864.3	871.4	7.1	0.8	-	-	-	-
744	695	E0401a		488.1	-	293	1.0	996.1	983.1	-13.0	-1.3	-	-	-	-
745	696	E0402a		-	-	298	1.0	980.3	968.1	-12.2	-1.2	70.8	69.9	-0.9	-1.3
746	697	E0403a		494.1	-	298	1.0	982.8	977.0	-5.7	-0.6	-	-	-	-
747	698	E0404a		524.0	-	298	1.0	997.4	983.8	-13.6	-1.4	-	-	-	-
748	699	E0405a		518.1	-	298	1.0	1003.7	990.1	-13.6	-1.4	73.0	74.6	1.6	2.2
749	700	E0406a		541.1	-	298	1.0	1019.2	1009.8	-9.4	-0.9	78.1	77.9	-0.2	-0.3
750	701	E0407a		513.1	-	298	1.0	995.3	983.0	-12.3	-1.2	73.2	72.1	-1.1	-1.5
751	702	L1101a		337.9	512.6	298	1.01	786.7	737.7	-49.0	-6.2	37.7	37.8	0.1	0.2
752	703	L2101a		351.4	513.9	298	1.01	786.6	756.7	-29.9	-3.8	42.3	42.4	0.2	0.4
753	704	L2201a		470.4	720.0	298	1.0	1109.9	1112.6	2.6	0.2	64.8	67.5	2.7	4.1
754	705	L3101a		355.4	508.3	298	1.0	781.2	752.0	-29.2	-3.7	44.4	44.8	0.4	0.8
755	706	L3102a		370.4	536.8	298	1.0	799.8	782.9	-16.9	-2.1	46.6	47.2	0.6	1.2
756	707	L3201a		460.8	700.2	298	1.0	1032.5	1010.5	-22.0	-2.1	62.2	63.1	0.9	1.4
757	708	L3202a	×	487.6	724.0	298	1.0	1050.3	1018.0	-32.3	-3.1	69.8	59.1	-10.7	-15.3
758	709	L3301a	×	563.1	850.0	298	1.0	1258.3	1164.6	-93.7	-7.4	-	-	-	-
759	709	L3301b		563.1	850.0	308	1.0	-	-	-	-	85.8	78.5	-7.3	-8.6
760	710	L4101a		355.6	506.2	299	1.0	779.5	748.8	-30.7	-3.9	46.2	41.8	-4.4	-9.6
761	711	L4102a		380.8	547.8	298	1.0	797.8	791.5	-6.3	-0.8	50.8	50.0	-0.8	-1.6
762	712	L4103a		372.7	536.0	298	1.01	803.0	783.2	-19.8	-2.5	48.5	49.1	0.6	1.3
763	713	L4104a		390.8	563.0	298	1.0	805.8	799.4	-6.4	-0.8	52.1	52.1	-0.0	-0.0
764	714	L4201a		451.1	728.8	298	1.0	989.6	938.8	-50.8	-5.1	-	-	-	-
765	715	L4202a		455.1	728.8	298	1.0	999.8	949.9	-49.9	-5.0	-	-	-	-
766	716	L4203a	×	487.1	728.8	293	1.0	1009.0	978.5	-30.5	-3.0	71.3	62.1	-9.2	-12.9

Table S.13 – Comparison of experimental and simulated properties using LB combination rules (continued).

n_{sim}	n_{iso}	Code	Outlier	T_m [K]	T_b [K]	T [K]	P [bar]	ρ_{liq}[kg·m^{-3}] exp	sim	dev	err [%]	ΔH_{vap}[kJ·mol^{-1}] exp	sim	dev	err [%]
767	717	L4204a		464.1	680.0	298	1.0	999.2	979.6	-19.6	-2.0	-	-	-	-
768	718	L4205a	×	480.1	676.0	298	1.0	1000.2	950.8	-49.4	-4.9	72.6	58.8	-13.8	-18.9
769	719	L4206a		501.1	728.8	298	1.0	1015.4	1005.4	-10.0	-1.0	76.6	74.6	-2.0	-2.7
770	720	L4301a		-	697.0	298	1.0	1184.0	1125.6	-58.4	-4.9	-	-	-	-
771	721	L5101a		375.1	543.7	298	1.0	804.7	782.7	-22.0	-2.7	50.1	46.2	-3.9	-7.8
772	722	L5102a		384.6	556.1	298	1.0	815.0	794.0	-21.0	-2.6	-	-	-	-
773	723	L5103a		401.9	575.4	298	1.0	815.2	812.1	-3.1	-0.4	54.1	54.9	0.8	1.4
774	724	L5104a		388.4	559.6	298	1.0	815.4	795.6	-19.8	-2.4	52.9	52.9	0.0	0.0
775	725	L5105a		404.4	577.2	298	1.0	806.9	805.6	-1.3	-0.2	55.3	55.9	0.6	1.1
776	726	L5106a		392.1	560.3	298	1.01	805.3	793.3	-12.0	-1.5	53.6	53.8	0.1	0.3
777	727	L5107a		410.9	588.1	298	1.0	811.3	808.5	-2.8	-0.3	56.9	56.7	-0.2	-0.3
778	728	L5201a		447.1	754.5	298	1.0	968.8	937.1	-31.7	-3.3	-	-	-	-
779	729	L5202a		472.1	754.5	293	1.0	964.5	921.3	-43.2	-4.5	-	-	-	-
780	730	L5203a		473.1	754.5	293	1.0	991.7	936.9	-54.8	-5.5	-	-	-	-
781	731	L5204a		473.1	754.5	295	1.0	984.2	953.5	-30.7	-3.1	-	-	-	-
782	732	L5205a		460.6	754.5	292	1.0	979.8	945.4	-34.4	-3.5	-	-	-	-
783	733	L5206a	×	472.1	754.5	298	1.0	956.0	903.2	-52.8	-5.5	72.5	61.6	-10.9	-15.0
784	734	L5207a		475.2	754.5	293	1.0	997.0	963.2	-33.8	-3.4	-	-	-	-
785	735	L5208a		494.1	754.5	293	1.0	981.0	945.4	-35.6	-3.6	-	-	-	-
786	736	L5209a		482.1	754.5	297	1.0	969.1	958.0	-11.1	-1.1	74.6	74.8	0.2	0.2
787	737	L5210a		497.0	754.5	293	1.0	989.5	973.2	-16.3	-1.6	-	-	-	-
788	738	L5211a		512.1	754.5	298	1.0	989.7	984.9	-4.8	-0.5	86.8	86.4	-0.4	-0.5
789	739	L5301a		460.6	-	298	1.0	1103.6	1083.2	-20.4	-1.8	-	-	-	-
790	740	L6101a		393.1	596.0	298	0.01	813.9	816.4	2.5	0.3	53.8	52.6	-1.2	-2.3
791	741	L6102a		391.8	596.0	298	1.0	818.6	809.0	-9.6	-1.2	54.0	49.7	-4.3	-8.0
792	742	L6103a		409.9	596.0	298	1.0	824.5	830.4	5.9	0.7	-	-	-	-
793	743	L6104a		395.6	575.6	298	1.0	823.8	808.9	-14.9	-1.8	-	-	-	-
794	743	L6104b		395.6	575.6	337	1.0	-	-	-	-	40.1	43.9	3.8	9.6
795	744	L6105a		416.1	596.0	298	1.0	809.7	822.5	12.8	1.6	58.0	59.2	1.2	2.1
796	745	L6106a		394.6	559.5	298	1.0	809.5	792.3	-17.1	-2.1	54.7	51.2	-3.5	-6.4
797	746	L6107a		422.1	596.0	298	1.0	823.7	826.6	2.9	0.3	-	-	-	-
798	747	L6108a		407.4	596.0	298	1.0	824.7	811.6	-13.1	-1.6	58.2	56.0	-2.2	-3.7
799	748	L6109a		399.7	596.0	298	1.0	820.1	805.8	-14.3	-1.7	56.0	55.7	-0.3	-0.6
800	749	L6110a		404.9	574.4	298	1.0	803.0	797.8	-5.2	-0.7	-	-	-	-
801	749	L6110b		404.9	574.4	308	1.0	-	-	-	-	49.6	56.0	6.4	13.0
802	750	L6111a		419.6	596.0	298	1.0	829.3	824.8	-4.5	-0.5	60.3	59.3	-1.0	-1.7
803	751	L6112a		425.6	596.0	298	1.0	820.5	822.0	1.5	0.2	61.7	60.6	-1.1	-1.8
804	752	L6113a		421.1	604.4	298	1.0	820.6	817.7	-2.9	-0.4	59.4	59.4	0.0	0.1
805	753	L6114a		408.6	596.0	298	1.0	814.5	801.4	-13.1	-1.6	58.6	57.7	-0.9	-1.6
806	754	L6115a		424.9	603.5	298	1.0	809.7	813.4	3.7	0.5	-	-	-	-
807	755	L6116a		413.0	585.9	298	1.0	810.3	800.6	-9.7	-1.2	58.3	58.2	-0.1	-0.1
808	756	L6117a		430.1	610.3	298	1.0	815.5	816.5	1.0	0.1	59.6	61.5	1.9	3.2
809	757	L6201a		445.9	777.8	316	0.002	970.4	919.4	-51.0	-5.3	-	-	-	-
810	758	L6202a		505.8	777.8	298	1.0	963.8	941.1	-22.7	-2.4	-	-	-	-
811	759	L6203a		478.6	777.8	323	1.0	940.0	932.4	-7.6	-0.8	-	-	-	-
812	760	L6204a		480.0	777.8	298	1.0	964.5	928.1	-36.4	-3.8	-	-	-	-
813	761	L6205a		505.8	777.8	293	1.0	962.7	931.4	-31.3	-3.3	-	-	-	-
814	762	L6206a		470.6	777.8	298	1.0	918.5	884.8	-33.8	-3.7	68.6	61.1	-7.5	-10.9

Table S.13 – Comparison of experimental and simulated properties using LB combination rules (continued).

n_{sim}	n_{iso}	Code	Outlier	T_m [K]	T_b [K]	T [K]	P [bar]	ρ_{liq}[kg·m^{-3}] exp	sim	dev	err [%]	ΔH_{vap}[kJ·mol^{-1}] exp	sim	dev	err [%]
815	763	L6207a		484.6	777.8	287	1.0	990.6	921.5	-69.1	-7.0	-	-	-	-
816	764	L6208a		498.9	777.8	323	1.0	958.2	938.6	-19.6	-2.0	-	-	-	-
817	765	L6209a		480.0	777.8	277	1.0	996.0	1002.5	6.5	0.7	-	-	-	-
818	766	L6210a		483.0	777.8	293	1.0	969.0	927.2	-41.8	-4.3	-	-	-	-
819	767	L6211a		494.4	777.8	293	1.0	964.5	959.2	-5.3	-0.5	-	-	-	-
820	768	L6212a		496.9	777.8	293	1.0	976.8	952.5	-24.3	-2.5	-	-	-	-
821	769	L6213a		480.0	777.8	298	1.0	967.7	931.5	-36.2	-3.7	-	-	-	-
822	770	L6214a		-	-	293	1.0	977.1	990.1	13.0	1.3	-	-	-	-
823	771	L6215a		493.4	777.8	295	1.0	973.7	927.9	-45.8	-4.7	-	-	-	-
824	772	L6216a		484.1	777.8	294	1.0	951.6	906.2	-45.4	-4.8	-	-	-	-
825	773	L6217a		493.9	777.8	323	1.0	939.8	926.5	-13.3	-1.4	-	-	-	-
826	774	L6218a		474.2	777.8	298	1.0	963.6	942.9	-20.7	-2.1	-	-	-	-
827	775	L6219a		521.5	777.8	293	1.0	972.6	979.4	6.8	0.7	-	-	-	-
828	776	L6220a		496.9	777.8	293	1.0	971.9	975.5	3.6	0.4	-	-	-	-
829	777	L6221a		508.1	777.8	295	1.0	958.0	932.7	-25.3	-2.6	-	-	-	-
830	778	L6222a		529.1	777.8	289	1.0	982.0	969.6	-12.4	-1.3	-	-	-	-
831	779	L6223a		497.1	777.8	385	0.01	917.4	864.2	-53.2	-5.8	-	-	-	-
832	780	L6224a		510.1	777.8	298	1.0	964.0	956.0	-8.0	-0.8	-	-	-	-
833	781	L6225a		516.1	777.8	318	1.0	968.3	957.4	-10.9	-1.1	-	-	-	-
834	781	L6225b		516.1	777.8	342	1.0	-	-	-	-	87.0	86.0	-1.0	-1.1
835	782	L6301a		-	-	293	1.0	1104.1	1086.3	-17.8	-1.6	-	-	-	-
836	783	L6302a		472.5	-	298	1.0	1100.0	1080.7	-19.2	-1.7	-	-	-	-
837	784	L7101a		404.1	619.7	298	1.0	833.5	843.1	9.6	1.2	-	-	-	-
838	784	L7101b		404.1	619.7	313	1.0	-	-	-	-	48.7	49.6	0.9	1.8
839	785	L7102a		430.1	619.7	293	1.0	846.6	854.9	8.3	1.0	-	-	-	-
840	786	L7103a		420.1	619.7	293	1.0	827.0	844.5	17.5	2.1	-	-	-	-
841	787	L7104a		413.1	619.7	298	1.0	837.3	828.3	-9.0	-1.1	-	-	-	-
842	788	L7105a		433.1	619.7	298	1.0	823.8	850.9	27.1	3.3	-	-	-	-
843	789	L7106a		409.1	619.7	298	1.0	822.4	827.9	5.5	0.7	-	-	-	-
844	790	L7107a		412.1	619.7	298	1.0	828.5	823.7	-4.8	-0.6	-	-	-	-
845	791	L7108a		411.1	619.7	293	1.0	811.9	817.1	5.2	0.6	-	-	-	-
846	792	L7109a		406.1	619.7	298	1.0	810.0	802.2	-7.8	-1.0	-	-	-	-
847	793	L7110a		426.1	619.7	294	1.0	836.0	829.2	-6.8	-0.8	-	-	-	-
848	794	L7111a		411.9	619.7	298	1.0	824.9	820.3	-4.6	-0.6	-	-	-	-
849	794	L7111b		411.9	619.7	322	1.0	-	-	-	-	53.6	53.2	-0.4	-0.8
850	795	L7112a		430.1	619.7	293	1.0	828.2	854.2	26.0	3.1	-	-	-	-
851	796	L7113a		415.6	619.7	298	1.0	839.6	826.7	-12.8	-1.5	57.3	52.0	-5.3	-9.3
852	797	L7114a		438.1	619.7	293	1.0	832.0	848.2	16.2	2.0	-	-	-	-
853	798	L7115a		426.1	619.7	293	1.0	837.9	837.0	-0.9	-0.1	-	-	-	-
854	799	L7116a		415.9	619.7	298	1.0	820.2	814.4	-5.7	-0.7	-	-	-	-
855	800	L7117a		433.1	619.7	293	1.0	815.1	831.3	16.2	2.0	-	-	-	-
856	801	L7118a		415.9	619.7	298	1.0	809.8	802.0	-7.8	-1.0	58.6	55.8	-2.8	-4.7
857	802	L7119a		435.1	619.7	298	1.0	832.7	836.8	4.1	0.5	-	-	-	-
858	803	L7120a		425.1	619.7	298	1.0	833.3	822.9	-10.4	-1.2	-	-	-	-
859	804	L7121a		437.1	619.7	296	1.0	836.0	842.6	6.6	0.8	-	-	-	-
860	805	L7122a		438.1	619.7	297	1.0	819.0	835.4	16.4	2.0	-	-	-	-
861	806	L7123a		425.1	619.7	298	1.0	822.0	816.9	-5.1	-0.6	-	-	-	-
862	807	L7124a		418.1	619.7	293	1.0	823.9	816.0	-7.9	-1.0	-	-	-	-

Table S.13 – Comparison of experimental and simulated properties using LB combination rules (continued).

n_{sim}	n_{iso}	Code	Outlier	T_m [K]	T_b [K]	T [K]	P [bar]	ρ_{liq}[kg·m^{-3}] exp	sim	dev	err [%]	ΔH_{vap}[kJ·mol^{-1}] exp	sim	dev	err [%]
863	808	L7125a		432.1	619.7	298	1.0	816.0	820.5	4.5	0.5	-	-	-	-
864	809	L7126a		424.1	619.7	298	1.0	817.7	811.8	-5.9	-0.7	-	-	-	-
865	810	L7127a		421.1	619.7	293	1.0	833.1	811.7	-21.4	-2.6	59.8	62.5	2.7	4.4
866	811	L7128a		424.1	619.7	293	1.0	813.1	807.8	-5.3	-0.7	-	-	-	-
867	812	L7129a		439.1	619.7	298	1.0	828.8	827.1	-1.7	-0.2	-	-	-	-
868	813	L7130a		445.1	619.7	298	1.0	824.5	827.3	2.8	0.3	-	-	-	-
869	814	L7131a		446.1	619.7	297	1.0	821.0	827.5	6.5	0.8	-	-	-	-
870	815	L7132a		436.1	619.7	293	1.0	827.0	827.8	0.8	0.1	-	-	-	-
871	816	L7133a		427.9	619.7	298	1.0	815.6	807.7	-7.9	-1.0	62.4	62.7	0.3	0.5
872	817	L7134a		429.9	619.7	298	1.0	816.5	809.2	-7.3	-0.9	-	-	-	-
873	818	L7135a		445.1	619.7	298	1.0	819.2	820.4	1.2	0.1	-	-	-	-
874	819	L7136a		432.4	608.3	298	1.0	813.4	807.6	-5.7	-0.7	62.1	62.8	0.7	1.1
875	820	L7137a		449.4	632.6	298	1.0	819.2	822.7	3.6	0.4	66.5	66.2	-0.3	-0.4
876	821	L7201a		525.9	799.3	293	1.0	961.3	943.9	-17.4	-1.8	-	-	-	-
877	822	L7202a		517.0	799.3	293	1.0	952.8	974.1	21.3	2.2	-	-	-	-
878	823	L7203a		517.0	799.3	293	1.0	929.6	901.4	-28.2	-3.0	-	-	-	-
879	824	L7204a		517.0	799.3	291	1.0	932.1	892.5	-39.6	-4.2	-	-	-	-
880	825	L7205a		507.1	799.3	334	1.0	949.0	932.9	-16.1	-1.7	-	-	-	-
881	826	L7206a		494.2	799.3	295	1.0	967.0	953.6	-13.4	-1.4	-	-	-	-
882	827	L7207a		508.2	799.3	293	1.0	967.2	969.4	2.2	0.2	-	-	-	-
883	828	L7208a		508.2	799.3	298	1.0	926.0	898.6	-27.4	-3.0	-	-	-	-
884	829	L7209a		485.4	799.3	293	1.0	962.5	967.6	5.1	0.5	-	-	-	-
885	830	L7210a		485.4	799.3	293	1.0	967.9	969.6	1.7	0.2	-	-	-	-
886	831	L7211a		515.1	799.3	298	1.0	950.4	949.0	-1.4	-0.1	-	-	-	-
887	832	L7212a		489.8	799.3	293	1.0	970.5	955.8	-14.7	-1.5	-	-	-	-
888	833	L7213a		485.4	799.3	298	1.0	962.0	947.1	-14.9	-1.6	-	-	-	-
889	834	L7214a		535.1	799.3	298	1.0	952.2	959.0	6.7	0.7	96.5	97.1	0.6	0.6
890	835	L7301a		416.9	-	291	1.0	1075.0	1078.6	3.6	0.3	-	-	-	-
891	836	L8101a		433.1	641.4	298	1.0	815.1	862.6	47.5	5.8	-	-	-	-
892	837	L8102a		425.1	641.4	298	1.0	842.9	848.0	5.1	0.6	-	-	-	-
893	838	L8103a		419.6	641.4	293	1.0	823.7	817.9	-5.8	-0.7	-	-	-	-
894	839	L8104a		430.1	641.4	293	1.0	849.2	845.2	-4.0	-0.5	-	-	-	-
895	840	L8105a		431.1	641.4	293	1.0	840.8	850.4	9.6	1.1	-	-	-	-
896	841	L8106a		424.1	641.4	293	1.0	832.4	837.3	4.9	0.6	-	-	-	-
897	842	L8107a		431.1	641.4	293	1.0	808.0	837.8	29.8	3.7	-	-	-	-
898	843	L8108a		433.0	641.4	293	1.0	857.6	863.9	6.3	0.7	-	-	-	-
899	844	L8109a		433.1	641.4	293	1.0	829.5	844.1	14.6	1.8	-	-	-	-
900	845	L8110a		432.1	641.4	293	1.0	834.1	852.7	18.6	2.2	-	-	-	-
901	846	L8111a		433.0	641.4	293	1.0	845.7	846.3	0.6	0.1	-	-	-	-
902	847	L8112a		425.1	641.4	298	1.0	834.5	839.4	4.9	0.6	-	-	-	-
903	848	L8113a		431.4	641.4	293	1.0	837.1	837.3	0.2	0.0	-	-	-	-
904	849	L8114a		446.0	641.4	298	1.0	842.5	856.3	13.8	1.6	-	-	-	-
905	850	L8115a		429.2	641.4	293	1.0	834.2	837.4	3.2	0.4	-	-	-	-
906	851	L8116a		430.9	641.4	298	1.0	834.6	830.9	-3.7	-0.4	-	-	-	-
907	852	L8117a		433.2	641.4	298	1.0	831.0	829.2	-1.8	-0.2	-	-	-	-
908	853	L8118a		441.4	641.4	293	1.0	838.4	839.4	1.0	0.1	-	-	-	-
909	853	L8118b		441.4	641.4	348	1.0	-	-	-	-	54.7	56.3	1.6	2.9
910	854	L8119a		425.1	641.4	298	1.0	827.0	815.3	-11.7	-1.4	-	-	-	-

Table S.13 – Comparison of experimental and simulated properties using LB combination rules (continued).

n_{sim}	n_{iso}	Code	Outlier	T_m [K]	T_b [K]	T [K]	P [bar]	ρ_{liq}[kg·m^{-3}] exp	sim	dev	err [%]	ΔH_{vap}[kJ·mol^{-1}] exp	sim	dev	err [%]
911	855	L8120a		444.1	641.4	293	1.0	833.0	836.0	3.0	0.4	-	-	-	-
912	856	L8121a		426.1	641.4	293	1.0	833.9	830.1	-3.8	-0.5	-	-	-	-
913	857	L8122a		423.9	641.4	301	1.0	806.5	813.0	6.5	0.8	-	-	-	-
914	858	L8123a		425.6	641.4	298	1.0	811.5	806.7	-4.8	-0.6	-	-	-	-
915	859	L8124a		446.1	641.4	298	1.0	842.5	847.1	4.6	0.5	-	-	-	-
916	860	L8125a		456.1	641.4	293	1.0	849.8	854.1	4.3	0.5	-	-	-	-
917	861	L8126a		432.1	641.4	298	1.0	813.5	819.4	5.9	0.7	-	-	-	-
918	862	L8127a		432.1	641.4	298	1.0	834.2	829.4	-4.8	-0.6	-	-	-	-
919	863	L8128a		454.0	641.4	293	1.0	839.0	850.5	11.5	1.4	-	-	-	-
920	864	L8129a		445.6	641.4	293	1.0	826.5	841.0	14.5	1.8	-	-	-	-
921	865	L8130a		434.2	641.4	298	1.0	820.2	818.6	-1.6	-0.2	-	-	-	-
922	865	L8130b		434.2	641.4	345	1.0	-	-	-	-	54.8	51.8	-3.0	-5.6
923	866	L8131a		434.1	641.4	298	1.0	824.9	820.8	-4.1	-0.5	-	-	-	-
924	866	L8131b		434.1	641.4	353	1.0	-	-	-	-	54.7	50.3	-4.4	-8.0
925	867	L8132a		429.9	641.4	298	1.0	805.0	808.8	3.8	0.5	-	-	-	-
926	867	L8132b		429.9	641.4	358	1.0	-	-	-	-	53.1	50.5	-2.6	-4.9
927	868	L8133a		437.1	641.4	332	0.01	812.9	803.0	-9.9	-1.2	-	-	-	-
928	869	L8134a		428.6	641.4	298	1.0	794.2	825.2	31.0	3.9	-	-	-	-
929	869	L8134b	×	428.6	641.4	319	0.01	-	-	-	-	43.5	62.5	19.0	43.6
930	870	L8135a		437.9	641.4	298	1.0	833.5	824.5	-9.0	-1.1	-	-	-	-
931	870	L8135b		437.9	641.4	355	1.0	-	-	-	-	48.0	55.9	7.9	16.4
932	871	L8136a		439.2	641.4	298	1.0	817.6	822.8	5.2	0.6	-	-	-	-
933	871	L8136b	×	439.2	641.4	356	1.0	-	-	-	-	48.0	56.6	8.6	17.9
934	872	L8137a		440.8	641.4	298	1.0	821.0	817.8	-3.2	-0.4	-	-	-	-
935	872	L8137b		440.8	641.4	364	1.0	-	-	-	-	54.8	53.7	-1.1	-2.0
936	873	L8138a		449.6	641.4	293	1.0	827.3	833.5	6.2	0.7	-	-	-	-
937	873	L8138b		449.6	641.4	365	1.0	-	-	-	-	53.3	57.8	4.5	8.4
938	874	L8139a		426.8	641.4	298	1.0	814.1	820.6	6.4	0.8	-	-	-	-
939	875	L8140a		456.1	641.4	293	1.0	828.2	834.8	6.5	0.8	-	-	-	-
940	876	L8141a		444.8	641.4	298	1.0	799.0	818.1	19.1	2.4	-	-	-	-
941	877	L8142a		452.1	641.4	298	1.0	824.5	826.4	1.9	0.2	-	-	-	-
942	878	L8143a		445.1	641.4	298	1.0	810.0	818.5	8.5	1.0	-	-	-	-
943	879	L8144a		439.2	641.4	298	1.0	809.8	812.4	2.6	0.3	-	-	-	-
944	880	L8145a		433.0	641.4	298	1.0	776.6	813.0	36.4	4.7	-	-	-	-
945	881	L8146a		445.1	641.4	298	1.0	803.4	810.0	6.6	0.8	-	-	-	-
946	882	L8147a		453.0	641.4	301	1.0	829.0	834.7	5.7	0.7	-	-	-	-
947	883	L8148a		457.8	640.6	298	1.0	828.7	831.9	3.2	0.4	68.5	68.3	-0.2	-0.2
948	884	L8149a		456.4	641.4	298	1.0	806.0	830.6	24.6	3.0	-	-	-	-
949	885	L8150a		459.1	641.4	298	1.0	784.5	831.6	47.1	6.0	-	-	-	-
950	886	L8151a		459.8	641.4	298	1.0	815.2	831.7	16.5	2.0	-	-	-	-
951	887	L8152a		448.8	641.4	298	1.0	798.7	827.3	28.6	3.6	-	-	-	-
952	888	L8153a		449.8	641.4	298	1.0	815.9	813.9	-2.0	-0.2	67.2	67.6	0.4	0.6
953	889	L8154a		447.9	641.4	298	1.0	817.0	815.0	-2.0	-0.2	-	-	-	-
954	890	L8155a		460.9	641.4	298	1.0	817.6	824.3	6.7	0.8	-	-	-	-
955	891	L8156a		452.9	629.6	298	1.0	817.0	814.1	-2.9	-0.4	67.9	67.6	-0.3	-0.5
956	892	L8157a		468.4	652.5	298	1.0	821.8	826.4	4.7	0.6	70.1	70.7	0.6	0.8
957	893	L8201a		-	-	298	1.0	928.5	922.4	-6.1	-0.7	-	-	-	-
958	894	L8202a		-	-	293	1.0	917.2	893.7	-23.5	-2.6	-	-	-	-

Table S.13 – Comparison of experimental and simulated properties using LB combination rules (continued).

n_{sim}	n_{iso}	Code	Outlier	T_m [K]	T_b [K]	T [K]	P [bar]	ρ_{liq}[kg·m^{-3}] exp	sim	dev	err [%]	ΔH_{vap}[kJ·mol^{-1}] exp	sim	dev	err [%]
959	895	L8203a		-	-	298	1.0	929.0	916.6	-12.4	-1.3	-	-	-	-
960	896	L8204a		-	-	292	1.0	970.4	963.7	-6.7	-0.7	-	-	-	-
961	897	L8205a		-	-	298	1.0	959.0	939.6	-19.4	-2.0	-	-	-	-
962	898	L8206a		517.1	819.3	295	1.0	932.5	921.6	-10.9	-1.2	-	-	-	-
963	899	L8207a		478.1	819.3	298	1.0	918.0	895.8	-22.2	-2.4	-	-	-	-
964	900	L8208a		-	-	298	1.0	949.0	940.3	-8.7	-0.9	-	-	-	-
965	901	L8209a		-	-	298	1.0	943.0	938.6	-4.4	-0.5	-	-	-	-
966	902	L8210a	×	544.1	819.3	356	1.0	-	-	-	-	101.0	92.6	-8.4	-8.3
967	903	L9101a		447.1	661.5	298	1.0	852.6	844.8	-7.8	-0.9	-	-	-	-
968	904	L9102a		439.1	661.5	294	1.0	832.3	868.5	36.2	4.4	-	-	-	-
969	905	L9103a		446.2	661.5	293	1.0	846.2	855.5	9.3	1.1	-	-	-	-
970	906	L9104a		466.0	661.5	293	1.0	847.5	835.6	-11.9	-1.4	-	-	-	-
971	907	L9105a		439.0	661.5	293	1.0	835.0	828.7	-6.3	-0.8	-	-	-	-
972	908	L9106a		451.1	661.5	298	1.0	854.9	839.8	-15.1	-1.8	-	-	-	-
973	909	L9107a		444.1	661.5	293	1.0	848.8	854.7	5.9	0.7	-	-	-	-
974	910	L9108a		439.0	661.5	293	1.0	825.6	833.7	8.1	1.0	-	-	-	-
975	911	L9109a		466.0	661.5	288	1.0	835.3	850.8	15.5	1.9	-	-	-	-
976	912	L9110a		442.0	661.5	293	1.0	825.0	840.1	15.1	1.8	-	-	-	-
977	913	L9111a		452.0	661.5	288	1.0	861.0	857.9	-3.1	-0.4	-	-	-	-
978	914	L9112a		457.2	661.5	298	1.0	844.5	841.3	-3.2	-0.4	-	-	-	-
979	915	L9113a		452.0	661.5	298	1.0	899.4	840.9	-58.5	-6.5	-	-	-	-
980	916	L9114a		447.1	661.5	294	1.0	838.3	839.4	1.1	0.1	-	-	-	-
981	917	L9115a		475.1	661.5	293	1.0	846.0	859.6	13.6	1.6	-	-	-	-
982	918	L9116a		451.0	661.5	298	1.0	823.7	835.8	12.1	1.5	-	-	-	-
983	919	L9117a		451.1	661.5	298	1.0	833.4	833.8	0.4	0.0	-	-	-	-
984	920	L9118a		461.1	661.5	298	1.0	837.0	851.3	14.3	1.7	-	-	-	-
985	921	L9119a		445.1	661.5	295	1.0	839.6	830.1	-9.5	-1.1	-	-	-	-
986	922	L9120a		450.0	661.5	298	1.0	821.8	826.8	5.0	0.6	-	-	-	-
987	923	L9121a		444.6	661.5	293	1.0	824.2	823.6	-0.6	-0.1	-	-	-	-
988	924	L9122a		446.1	661.5	289	1.0	828.5	831.3	2.8	0.3	-	-	-	-
989	925	L9123a		466.1	661.5	298	1.0	823.6	840.5	16.9	2.0	67.9	70.6	2.7	4.0
990	926	L9124a		455.0	661.5	298	1.0	828.0	819.9	-8.1	-1.0	-	-	-	-
991	927	L9125a		-	-	295	1.0	830.0	821.5	-8.5	-1.0	-	-	-	-
992	928	L9126a		444.1	661.5	293	1.0	818.6	815.8	-2.8	-0.3	-	-	-	-
993	929	L9127a		452.0	661.5	293	1.0	827.5	843.1	15.6	1.9	-	-	-	-
994	930	L9128a		460.1	661.5	293	1.0	859.2	845.2	-14.0	-1.6	-	-	-	-
995	931	L9129a		448.1	661.5	293	1.0	814.8	823.3	8.5	1.0	-	-	-	-
996	932	L9130a		467.1	661.5	273	1.0	878.7	839.8	-38.9	-4.4	-	-	-	-
997	933	L9131a		451.0	661.5	298	1.0	806.4	817.4	10.9	1.4	65.2	70.4	5.2	8.0
998	934	L9132a		452.1	661.5	298	1.0	829.9	832.3	2.4	0.3	-	-	-	-
999	935	L9133a		455.4	661.5	298	1.0	829.9	833.1	3.2	0.4	-	-	-	-
1000	936	L9134a		454.1	661.5	298	1.0	824.6	824.4	-0.1	-0.0	-	-	-	-
1001	937	L9135a		-	-	298	1.0	827.5	824.5	-3.0	-0.4	-	-	-	-
1002	938	L9136a		455.0	661.5	293	1.0	843.9	839.7	-4.2	-0.5	-	-	-	-
1003	939	L9137a		451.1	661.5	298	1.0	813.4	816.0	2.6	0.3	-	-	-	-
1004	940	L9138a		466.1	661.5	298	1.0	835.8	847.3	11.5	1.4	-	-	-	-
1005	941	L9139a		455.0	661.5	298	1.0	815.6	828.9	13.3	1.6	-	-	-	-
1006	942	L9140a		459.0	661.5	298	1.0	843.7	829.6	-14.1	-1.7	-	-	-	-

Table S.13 – Comparison of experimental and simulated properties using LB combination rules (continued).

n_{sim}	n_{iso}	Code	Outlier	T_m [K]	T_b [K]	T [K]	P [bar]	ρ_{liq}[kg·m^{-3}] exp	sim	dev	err [%]	ΔH_{vap}[kJ·mol^{-1}] exp	sim	dev	err [%]
1007	943	L9141a		453.1	661.5	291	1.0	834.0	834.0	-0.0	-0.0	-	-	-	-
1008	944	L9142a		454.0	661.5	300	1.0	831.0	824.6	-6.4	-0.8	-	-	-	-
1009	945	L9143a		457.1	661.5	293	1.0	827.0	826.4	-0.6	-0.1	-	-	-	-
1010	946	L9144a		468.1	661.5	293	1.0	828.8	842.3	13.5	1.6	-	-	-	-
1011	947	L9145a		465.1	661.5	293	1.0	825.6	835.2	9.6	1.2	-	-	-	-
1012	948	L9146a		466.0	661.5	298	1.0	820.8	832.6	11.8	1.4	-	-	-	-
1013	949	L9147a		455.0	661.5	296	1.0	822.0	825.4	3.4	0.4	-	-	-	-
1014	950	L9148a		459.0	661.5	301	1.0	832.0	821.1	-10.9	-1.3	-	-	-	-
1015	951	L9149a		-	-	300	1.0	823.0	831.3	8.3	1.0	-	-	-	-
1016	952	L9150a		454.0	661.5	298	1.0	821.0	821.5	0.5	0.1	-	-	-	-
1017	953	L9151a		457.1	661.5	298	1.0	815.0	818.5	3.5	0.4	-	-	-	-
1018	954	L9152a		455.0	661.5	293	1.0	813.6	820.3	6.7	0.8	-	-	-	-
1019	955	L9153a		459.0	661.5	285	1.0	840.2	827.4	-12.8	-1.5	-	-	-	-
1020	956	L9154a		480.1	661.5	296	1.0	834.0	840.3	6.3	0.8	-	-	-	-
1021	957	L9155a		472.0	661.5	298	1.0	848.0	839.4	-8.6	-1.0	-	-	-	-
1022	958	L9156a		473.0	661.5	301	1.0	820.0	832.8	12.8	1.6	-	-	-	-
1023	959	L9157a		473.0	661.5	297	1.0	828.0	835.2	7.2	0.9	-	-	-	-
1024	960	L9158a		473.0	661.5	297	1.0	827.0	835.2	8.2	1.0	-	-	-	-
1025	961	L9159a		473.0	661.5	277	1.0	841.8	846.0	4.2	0.5	-	-	-	-
1026	962	L9160a		468.2	661.5	298	1.0	818.3	819.3	1.0	0.1	71.4	72.2	0.8	1.1
1027	963	L9161a		466.1	661.5	293	1.0	826.3	822.5	-3.8	-0.5	71.5	72.8	1.3	1.8
1028	964	L9162a		467.9	661.5	298	1.0	823.5	820.2	-3.4	-0.4	70.9	71.7	0.8	1.1
1029	965	L9163a		479.1	661.5	298	1.0	826.0	829.1	3.1	0.4	-	-	-	-
1030	966	L9164a		471.6	649.6	298	1.0	819.4	818.8	-0.6	-0.1	72.9	72.1	-0.8	-1.1
1031	967	L9165a		486.2	670.7	298	1.0	824.6	830.9	6.3	0.8	72.2	75.5	3.3	4.5
1032	968	L9201a		-	-	301	1.0	920.7	896.0	-24.7	-2.7	-	-	-	-
1033	969	L9202a		-	-	290	1.0	913.8	891.6	-22.2	-2.4	-	-	-	-
1034	970	L9203a		-	-	291	1.0	902.0	887.7	-14.3	-1.6	-	-	-	-
1035	971	L9204a		535.1	837.9	323	1.0	929.0	927.6	-1.4	-0.2	-	-	-	-
1036	971	L9204b		535.1	837.9	460	1.0	-	-	-	-	67.2	63.7	-3.5	-5.2
1037	972	L9205a		-	-	291	1.0	958.0	958.0	0.0	0.0	-	-	-	-
1038	973	L9206a		-	-	293	1.0	941.6	949.1	7.5	0.8	-	-	-	-
1039	974	L9207a		-	-	293	1.0	937.0	935.4	-1.6	-0.2	-	-	-	-
1040	975	L9208a		571.1	837.9	293	1.0	929.5	933.7	4.2	0.4	-	-	-	-
1041	976	L9209a		-	-	298	1.0	944.0	938.2	-5.8	-0.6	-	-	-	-
1042	977	L9210a	×	558.1	837.9	323	1.0	-	-	-	-	110.0	101.6	-8.4	-7.7
1043	978	L0101a		464.1	680.4	293	1.0	862.4	859.3	-3.1	-0.4	-	-	-	-
1044	979	L0102a		475.1	680.4	293	1.0	874.2	886.7	12.5	1.4	-	-	-	-
1045	980	L0103a		474.1	680.4	293	1.0	874.5	883.1	8.6	1.0	-	-	-	-
1046	981	L0104a		465.1	680.4	293	1.0	858.4	875.1	16.7	1.9	-	-	-	-
1047	982	L0105a		513.5	680.4	293	1.0	839.3	855.3	16.0	1.9	-	-	-	-
1048	983	L0106a		463.1	680.4	293	1.0	854.9	869.8	14.9	1.7	-	-	-	-
1049	984	L0107a		468.1	680.4	293	1.0	862.3	870.5	8.2	0.9	-	-	-	-
1050	985	L0108a		475.0	680.4	293	1.0	837.8	842.3	4.5	0.5	-	-	-	-
1051	986	L0109a		467.6	680.4	298	1.0	859.1	850.1	-9.0	-1.0	-	-	-	-
1052	987	L0110a		459.1	680.4	293	1.0	848.7	855.7	7.0	0.8	-	-	-	-
1053	988	L0111a		460.0	680.4	293	1.0	842.8	837.0	-5.8	-0.7	-	-	-	-
1054	989	L0112a		468.8	680.4	293	1.0	854.3	844.5	-9.8	-1.1	-	-	-	-

Table S.13 – Comparison of experimental and simulated properties using LB combination rules (continued).

n_{sim}	n_{iso}	Code	Outlier	T_m	T_b	T	P	ρ_{liq}[kg·m^{-3}]				ΔH_{vap}[kJ·mol^{-1}]			
				[K]	[K]	[K]	[bar]	exp	sim	dev	err [%]	exp	sim	dev	err [%]
1055	990	L0113a		503.4	687.3	298	1.0	-	-	-	-	80.9	80.1	-0.8	-0.9
1056	991	L0114a		454.1	680.4	293	1.0	833.0	831.4	-1.6	-0.2	-	-	-	-
1057	992	L0115a		465.1	680.4	293	1.0	853.7	844.8	-8.9	-1.0	-	-	-	-
1058	993	L0116a		460.0	680.4	293	1.0	860.6	866.7	6.1	0.7	-	-	-	-
1059	994	L0117a		463.0	680.4	293	1.0	839.8	838.2	-1.6	-0.2	-	-	-	-
1060	995	L0118a		460.1	680.4	293	1.0	833.9	853.0	19.1	2.3	-	-	-	-
1061	996	L0119a		463.0	680.4	293	1.0	823.6	839.3	15.7	1.9	-	-	-	-
1062	997	L0120a		455.1	680.4	294	1.0	823.0	821.9	-1.1	-0.1	-	-	-	-
1063	998	L0121a		466.0	680.4	293	1.0	826.5	836.7	10.2	1.2	-	-	-	-
1064	999	L0122a		466.1	680.4	291	1.0	833.0	833.6	0.6	0.1	-	-	-	-
1065	1000	L0123a		463.1	680.4	298	1.0	841.3	842.4	1.0	0.1	-	-	-	-
1066	1001	L0124a		466.1	680.4	293	1.0	845.5	846.4	0.9	0.1	-	-	-	-
1067	1002	L0125a		462.2	680.4	298	1.0	824.9	836.7	11.8	1.4	-	-	-	-
1068	1003	L0126a		464.0	680.4	301	1.0	825.8	826.3	0.5	0.1	-	-	-	-
1069	1004	L0127a		466.0	680.4	298	1.0	837.0	828.0	-9.0	-1.1	-	-	-	-
1070	1005	L0128a		464.0	680.4	293	1.0	823.2	828.7	5.5	0.7	-	-	-	-
1071	1006	L0129a		465.1	680.4	295	1.0	834.7	835.5	0.8	0.1	-	-	-	-
1072	1007	L0130a		465.1	680.4	273	1.0	842.1	846.2	4.1	0.5	-	-	-	-
1073	1008	L0131a		469.1	680.4	298	1.0	826.0	826.6	0.6	0.1	-	-	-	-
1074	1009	L0132a		463.1	680.4	293	1.0	821.2	837.1	15.9	1.9	-	-	-	-
1075	1010	L0133a		485.0	680.4	293	1.0	825.7	829.0	3.3	0.4	-	-	-	-
1076	1011	L0134a		485.0	680.4	293	1.0	827.3	826.6	-0.7	-0.1	-	-	-	-
1077	1012	L0135a		485.0	680.4	298	1.0	821.1	818.3	-2.8	-0.3	-	-	-	-
1078	1013	L0136a		466.0	680.4	290	1.0	865.0	852.3	-12.7	-1.5	-	-	-	-
1079	1014	L0137a		486.1	680.4	293	1.0	832.2	842.5	10.3	1.2	-	-	-	-
1080	1015	L0138a		464.0	680.4	298	1.0	821.5	834.5	13.0	1.6	-	-	-	-
1081	1016	L0139a		485.0	680.4	293	1.0	829.1	831.4	2.3	0.3	-	-	-	-
1082	1017	L0140a		467.1	680.4	293	1.0	815.2	829.1	13.9	1.7	-	-	-	-
1083	1018	L0141a		477.1	680.4	277	1.0	846.0	846.4	0.4	0.0	-	-	-	-
1084	1019	L0142a		475.1	680.4	293	1.0	814.0	825.5	11.5	1.4	-	-	-	-
1085	1020	L0143a		467.1	680.4	298	1.0	828.3	834.2	5.9	0.7	-	-	-	-
1086	1021	L0144a		466.0	680.4	298	1.0	826.8	835.8	9.0	1.1	-	-	-	-
1087	1022	L0145a		472.1	680.4	298	1.0	836.1	835.3	-0.8	-0.1	-	-	-	-
1088	1023	L0146a		481.1	680.4	293	1.0	830.0	847.0	17.0	2.0	-	-	-	-
1089	1024	L0147a		475.1	680.4	298	1.0	825.4	828.8	3.5	0.4	-	-	-	-
1090	1025	L0148a		482.0	680.4	298	1.0	827.1	828.4	1.3	0.2	-	-	-	-
1091	1026	L0149a		482.0	680.4	298	1.0	826.5	829.5	3.1	0.4	-	-	-	-
1092	1027	L0150a		487.0	680.4	298	1.0	807.8	820.2	12.4	1.5	-	-	-	-
1093	1028	L0151a		488.4	680.4	303	1.0	834.1	841.8	7.7	0.9	-	-	-	-
1094	1029	L0152a		482.0	680.4	300	1.0	826.0	831.7	5.7	0.7	-	-	-	-
1095	1030	L0153a		487.0	680.4	295	1.0	845.0	832.6	-12.4	-1.5	-	-	-	-
1096	1031	L0154a		481.6	680.4	298	1.0	824.5	827.7	3.2	0.4	-	-	-	-
1097	1032	L0155a		481.1	680.4	293	1.0	828.6	843.0	14.4	1.7	-	-	-	-
1098	1033	L0156a		472.0	680.4	298	1.0	839.6	832.7	-6.9	-0.8	-	-	-	-
1099	1034	L0157a		483.0	680.4	291	1.0	830.0	841.7	11.7	1.4	-	-	-	-
1100	1035	L0158a		485.1	680.4	298	1.0	832.0	835.1	3.1	0.4	-	-	-	-
1101	1036	L0159a		-	-	288	1.0	830.3	839.0	8.7	1.0	-	-	-	-
1102	1037	L0160a		487.0	680.4	293	1.0	833.2	829.3	-3.9	-0.5	-	-	-	-

Table S.13 – Comparison of experimental and simulated properties using LB combination rules (continued).

n_{sim}	n_{iso}	Code	Outlier	T_m [K]	T_b [K]	T [K]	P [bar]	ρ_{liq}[kg·m^{-3}] exp	sim	dev	err [%]	ΔH_{vap}[kJ·mol^{-1}] exp	sim	dev	err [%]
1103	1038	L0161a		482.0	680.4	298	1.0	820.0	822.6	2.6	0.3	-	-	-	-
1104	1039	L0162a		482.0	680.4	293	1.0	821.5	825.3	3.8	0.5	-	-	-	-
1105	1040	L0163a		-	-	289	1.0	836.0	842.9	6.9	0.8	-	-	-	-
1106	1041	L0164a		491.1	680.4	293	1.0	832.2	839.1	6.9	0.8	-	-	-	-
1107	1042	L0165a		487.0	680.4	297	1.0	831.0	837.9	6.9	0.8	-	-	-	-
1108	1043	L0166a		489.1	680.4	300	1.0	826.0	835.2	9.2	1.1	-	-	-	-
1109	1044	L0167a		487.0	680.4	296	1.0	837.0	838.5	1.5	0.2	-	-	-	-
1110	1045	L0168a		487.0	680.4	298	1.0	828.2	837.2	9.0	1.1	-	-	-	-
1111	1046	L0169a		495.1	680.4	288	1.0	833.6	841.0	7.3	0.9	-	-	-	-
1112	1047	L0170a		474.1	680.4	298	1.0	820.6	823.6	3.0	0.4	-	-	-	-
1113	1048	L0171a		483.1	680.4	293	1.0	825.0	826.5	1.5	0.2	-	-	-	-
1114	1049	L0172a		483.0	680.4	298	1.0	822.9	824.5	1.6	0.2	-	-	-	-
1115	1050	L0201a		-	-	288	1.0	910.2	891.0	-19.3	-2.1	-	-	-	-
1116	1051	L0202a		503.1	855.5	298	1.0	943.5	958.9	15.4	1.6	-	-	-	-
1117	1052	L0203a		-	-	285	1.0	911.8	901.7	-10.1	-1.1	-	-	-	-
1118	1053	L0204a		-	-	293	1.0	916.5	903.4	-13.1	-1.4	-	-	-	-
1119	1054	L0205a		538.1	855.5	293	1.0	926.0	932.7	6.7	0.7	-	-	-	-
1120	1055	L0206a		-	-	293	1.0	948.1	941.3	-6.8	-0.7	-	-	-	-
1121	1056	L0207a		-	-	293	1.0	936.2	947.7	11.5	1.2	-	-	-	-
1122	1057	L0208a		-	-	301	1.0	915.5	908.0	-7.5	-0.8	-	-	-	-
1123	1058	L0209a		-	-	298	1.0	930.7	947.9	17.2	1.9	-	-	-	-
1124	1059	L0210a		572.1	855.5	353	1.0	883.0	900.0	17.0	1.9	-	-	-	-
1125	1060	D1201a		373.7	588.0	298	1.0	1213.6	1256.6	43.0	3.5	46.3	39.4	-6.9	-14.8
1126	1061	D2201a		391.1	592.0	298	1.01	1043.5	1114.7	71.2	6.8	50.3	48.8	-1.5	-3.0
1127	1062	D3201a		414.3	600.8	298	1.01	988.1	1028.8	40.7	4.1	54.9	53.8	-1.1	-2.1
1128	1063	D4201a		427.6	-	298	1.0	943.9	980.6	36.7	3.9	56.3	57.0	0.7	1.3
1129	1064	D4202a		436.9	615.7	298	1.0	952.9	994.2	41.2	4.3	58.2	58.5	0.3	0.5
1130	1065	D5201a		436.9	631.0	310	1.0	907.5	954.7	47.3	5.2	-	-	-	-
1131	1065	D5201b		436.9	631.0	320	1.0	-	-	-	-	57.6	57.3	-0.3	-0.5
1132	1066	D5202a		450.1	631.0	298	1.0	934.7	970.2	35.5	3.8	-	-	-	-
1133	1067	D5203a		448.2	629.1	298	1.0	924.1	967.2	43.1	4.7	61.2	61.9	0.7	1.2
1134	1068	D5204a		458.6	639.2	298	1.0	934.4	973.8	39.4	4.2	63.0	63.6	0.6	1.0
1135	1069	D6201a		459.1	654.6	293	1.0	927.5	973.9	46.4	5.0	-	-	-	-
1136	1069	D6201b		459.1	654.6	370	1.0	-	-	-	-	59.4	57.6	-1.8	-3.0
1137	1070	D6202a		457.1	654.6	298	1.0	908.0	961.6	53.6	5.9	64.0	64.4	0.4	0.6
1138	1071	D6203a		464.9	654.6	293	1.0	927.5	966.4	38.9	4.2	-	-	-	-
1139	1072	D6204a		466.1	654.6	298	1.0	923.5	960.2	36.7	4.0	-	-	-	-
1140	1073	D6205a		-	654.6	298	1.0	918.2	954.9	36.7	4.0	-	-	-	-
1141	1074	D6206a		-	654.6	298	1.0	923.0	962.3	39.3	4.3	-	-	-	-
1142	1075	D6207a		473.6	654.6	298	1.0	917.0	956.8	39.8	4.3	-	-	-	-
1143	1076	D6208a		478.9	660.2	298	1.0	922.9	958.7	35.8	3.9	69.2	68.5	-0.7	-1.0
1144	1077	D7201a		-	676.3	293	1.0	885.6	960.5	74.9	8.5	-	-	-	-
1145	1078	D7202a		-	-	298	1.0	934.8	967.5	32.7	3.5	-	-	-	-
1146	1079	D7203a		-	-	298	1.0	910.0	942.9	32.9	3.6	-	-	-	-
1147	1080	D7204a		-	-	298	1.0	923.9	958.6	34.7	3.8	-	-	-	-
1148	1081	D7205a		-	676.3	298	1.0	909.8	947.4	37.6	4.1	-	-	-	-
1149	1082	D7206a		482.6	-	298	1.0	909.0	944.8	35.8	3.9	-	-	-	-
1150	1083	D7207a		486.1	676.3	293	1.0	918.7	954.0	35.3	3.8	-	-	-	-

Table S.13 – Comparison of experimental and simulated properties using LB combination rules (continued).

n_{sim}	n_{iso}	Code	Outlier	T_m [K]	T_b [K]	T [K]	P [bar]	ρ_{liq}[kg·m^{-3}] exp	sim	dev	err [%]	ΔH_{vap}[kJ·mol^{-1}] exp	sim	dev	err [%]
1151	1084	D7208a		489.1	676.3	293	1.0	916.3	948.9	32.6	3.6	-	-	-	-
1152	1085	D7209a		496.1	677.3	298	1.0	913.5	947.5	34.0	3.7	72.9	73.4	0.5	0.6
1153	1086	D8201a		-	-	298	1.0	919.9	968.4	48.5	5.3	-	-	-	-
1154	1087	D8202a		-	-	293	1.0	929.0	961.5	32.5	3.5	-	-	-	-
1155	1088	D8203a		-	-	298	1.0	900.9	956.2	55.3	6.1	-	-	-	-
1156	1089	D8204a		-	-	298	1.0	902.0	938.7	36.7	4.1	-	-	-	-
1157	1090	D8205a		494.1	696.5	298	1.0	904.0	936.9	32.9	3.6	74.8	75.9	1.1	1.5
1158	1091	D8206a		501.1	673.2	298	1.0	903.0	938.4	35.4	3.9	75.6	75.9	0.3	0.4
1159	1092	D8207a		-	-	293	1.0	910.5	946.8	36.3	4.0	-	-	-	-
1160	1093	D8208a		513.0	694.3	298	1.01	906.0	939.0	33.0	3.6	81.2	78.2	-3.0	-3.6
1161	1094	D9201a		-	-	298	1.0	910.2	947.7	37.5	4.1	-	-	-	-
1162	1095	D9202a		456.9	715.3	298	1.0	893.5	930.3	36.8	4.1	-	-	-	-
1163	1096	D9203a		456.9	715.3	277	1.0	909.7	943.9	34.2	3.8	-	-	-	-
1164	1097	D9204a		456.9	715.3	296	1.0	899.0	934.3	35.3	3.9	-	-	-	-
1165	1098	D9205a		528.8	710.7	298	1.0	901.6	932.4	30.8	3.4	-	-	-	-
1166	1098	D9205b		528.8	710.7	304	1.0	-	-	-	-	85.3	82.4	-2.9	-3.4
1167	1099	D0201a		473.7	733.0	293	1.0	902.2	936.5	34.3	3.8	-	-	-	-
1168	1100	D0202a		-	-	298	1.0	912.9	940.3	27.4	3.0	-	-	-	-
1169	1101	D0203a		-	-	291	1.0	897.0	930.9	33.9	3.8	-	-	-	-
1170	1102	D0204a		-	-	292	1.0	897.8	930.0	32.2	3.6	-	-	-	-
1171	1103	D0205a	×	-	733.0	293	1.0	848.1	929.4	81.3	9.6	-	-	-	-
1172	1104	D0206a		-	733.0	298	1.0	893.0	923.4	30.4	3.4	-	-	-	-
1173	1105	D0207a		473.7	733.0	293	1.0	898.3	931.7	33.4	3.7	-	-	-	-
1174	1106	D0208a		-	733.0	293	1.0	908.6	938.7	30.1	3.3	-	-	-	-
1175	1107	D0209a		543.1	722.1	313	1.0	881.7	916.1	34.4	3.9	88.6	86.0	-2.6	-3.0
1176	1108	N1101a		266.8	430.1	267	1.0	694.2	710.9	16.7	2.4	26.1	25.8	-0.3	-1.3
1177	1108	N1101b		266.8	430.1	298	3.52	655.0	642.3	-12.7	-1.9	-	-	-	-
1178	1109	N2101a		289.7	456.1	288	1.0	688.6	699.9	11.3	1.6	27.4	27.9	0.6	2.2
1179	1110	N2102a	×	280.0	437.2	283	1.0	667.9	751.9	83.9	12.6	27.2	32.6	5.4	19.9
1180	1110	N2102b		280.0	437.2	298	1.96	650.0	704.5	54.5	8.4	-	-	-	-
1181	1111	N2201a	×	390.4	593.0	298	0.02	892.8	995.2	102.4	11.5	45.0	47.1	2.1	4.6
1182	1112	N3101a		305.6	471.9	298	1.01	683.9	689.8	5.9	0.9	28.4	28.8	0.4	1.4
1183	1113	N3102a		321.6	496.9	298	1.01	712.8	715.6	2.9	0.4	31.3	30.3	-1.0	-3.3
1184	1114	N3103a		276.0	433.2	273	1.0	655.7	674.8	19.1	2.9	22.9	23.7	0.8	3.3
1185	1114	N3103b		276.0	433.2	298	2.33	628.9	647.4	18.4	2.9	-	-	-	-
1186	1115	N3201a		388.1	-	298	1.0	841.0	890.7	49.7	5.9	-	-	-	-
1187	1116	N3202a		392.4	585.0	298	1.0	-	-	-	-	44.2	44.7	0.5	1.1
1188	1117	N3203a		412.9	608.0	298	1.0	884.0	940.6	56.6	6.4	50.2	47.1	-3.1	-6.3
1189	1118	N4101a		317.6	483.9	298	1.0	690.1	676.8	-13.3	-1.9	29.6	25.9	-3.7	-12.5
1190	1119	N4102a		336.1	514.3	298	1.01	717.8	721.3	3.4	0.5	32.6	32.0	-0.6	-2.0
1191	1120	N4103a		340.9	522.4	298	1.0	729.1	725.1	-4.0	-0.6	33.8	31.9	-1.9	-5.6
1192	1121	N4104a		350.6	531.9	298	1.0	736.8	743.0	6.2	0.8	35.6	35.0	-0.6	-1.6
1193	1122	N4105a		323.6	482.4	288	1.0	740.0	712.4	-27.6	-3.7	30.7	30.7	0.0	0.0
1194	1123	N4106a		328.6	496.6	298	1.0	701.2	712.9	11.7	1.7	31.2	31.6	0.4	1.1
1195	1124	N4107a		336.1	482.4	298	0.2	711.9	711.6	-0.3	-0.0	-	-	-	-
1196	1125	N4108a		310.1	500.0	298	1.0	669.4	686.8	17.4	2.6	-	-	-	-
1197	1126	N4201a		378.6	-	298	1.0	803.0	843.6	40.6	5.1	-	-	-	-
1198	1127	N4202a		393.1	-	288	1.0	828.0	874.4	46.4	5.6	-	-	-	-

Table S.13 – Comparison of experimental and simulated properties using LB combination rules (continued).

n_{sim}	n_{iso}	Code	Outlier	T_m	T_b	T	P	$\rho_{\mathrm{liq}}[\mathrm{kg{\cdot}m^{-3}}]$				$\Delta H_{\mathrm{vap}}[\mathrm{kJ{\cdot}mol^{-1}}]$			
				[K]	[K]	[K]	[bar]	exp	sim	dev	err [%]	exp	sim	dev	err [%]
1199	1128	N4203a		396.1	-	298	1.0	841.0	858.8	17.8	2.1	45.8	39.3	-6.5	-14.2
1200	1129	N4204a		402.1	-	298	1.0	837.0	863.0	26.0	3.1	-	-	-	-
1201	1130	N4205a	×	413.1	-	298	1.0	-	-	-	-	53.1	43.6	-9.5	-17.8
1202	1131	N4206a		-	-	298	1.0	-	-	-	-	46.9	47.0	0.1	0.3
1203	1132	N4207a		431.6	-	298	1.0	877.0	932.1	55.1	6.3	55.2	54.9	-0.3	-0.5
1204	1133	N5101a		350.1	557.7	298	0.11	727.6	719.5	-8.0	-1.1	-	-	-	-
1205	1134	N5102a		350.1	557.7	298	0.1	728.1	743.9	15.8	2.2	-	-	-	-
1206	1135	N5103a		357.1	557.7	298	0.08	753.0	741.3	-11.7	-1.6	-	-	-	-
1207	1136	N5104a		362.1	557.7	291	1.0	711.3	749.7	38.4	5.4	-	-	-	-
1208	1137	N5105a		368.6	557.7	298	1.0	750.5	756.5	6.0	0.8	-	-	-	-
1209	1138	N5106a		370.1	557.7	298	1.0	744.3	759.1	14.8	2.0	-	-	-	-
1210	1139	N5107a		377.6	557.7	298	0.04	751.0	760.9	9.9	1.3	40.1	39.6	-0.5	-1.2
1211	1140	N5108a		350.1	517.7	298	1.0	727.0	739.4	12.4	1.7	32.3	30.9	-1.4	-4.2
1212	1141	N5109a		351.6	517.7	298	0.1	720.0	723.8	3.8	0.5	-	-	-	-
1213	1142	N5110a		346.1	517.7	298	0.12	730.0	715.2	-14.8	-2.0	-	-	-	-
1214	1143	N5111a		349.1	517.7	298	1.0	-	-	-	-	33.1	32.5	-0.6	-1.9
1215	1144	N5112a		353.4	517.7	298	0.11	726.7	721.3	-5.4	-0.7	-	-	-	-
1216	1145	N5113a		364.2	517.7	298	0.05	728.1	734.0	5.9	0.8	-	-	-	-
1217	1146	N5114a		339.2	517.7	298	1.0	710.6	714.3	3.7	0.5	31.9	31.1	-0.8	-2.4
1218	1147	N5115a		339.1	517.7	298	1.0	701.5	713.3	11.7	1.7	31.8	31.8	-0.0	-0.0
1219	1148	N5116a		339.1	517.7	298	0.21	695.6	711.8	16.3	2.3	-	-	-	-
1220	1149	N5201a		356.1	-	291	1.0	749.1	786.3	37.2	5.0	-	-	-	-
1221	1149	N5201b		356.1	-	298	1.0	-	-	-	-	33.1	36.8	3.7	11.2
1222	1150	N5202a		406.6	-	293	1.0	827.2	849.0	21.8	2.6	42.2	46.5	4.3	10.1
1223	1151	N5203a		437.1	-	298	1.0	-	-	-	-	54.9	49.5	-5.4	-9.9
1224	1152	N5204a		452.1	-	298	1.0	873.0	918.4	45.4	5.2	58.7	59.1	0.4	0.7
1225	1153	N6101a		375.1	589.1	298	0.04	760.1	767.2	7.2	0.9	-	-	-	-
1226	1154	N6102a		381.6	589.1	298	0.02	745.7	755.4	9.7	1.3	-	-	-	-
1227	1155	N6103a		404.6	589.1	298	1.0	763.0	775.3	12.3	1.6	45.0	44.3	-0.7	-1.6
1228	1156	N6104a		351.1	549.1	298	1.0	716.1	749.1	33.0	4.6	-	-	-	-
1229	1157	N6105a		357.1	523.1	298	1.0	712.3	724.7	12.4	1.7	34.5	34.5	0.0	0.1
1230	1158	N6106a		371.1	549.1	298	0.04	729.8	731.5	1.7	0.2	-	-	-	-
1231	1159	N6107a		371.4	549.1	298	0.04	723.2	726.7	3.5	0.5	37.3	35.8	-1.5	-4.1
1232	1160	N6108a		-	-	295	1.0	739.0	752.6	13.6	1.8	-	-	-	-
1233	1161	N6109a		382.0	550.0	298	1.0	734.9	730.7	-4.2	-0.6	40.0	37.1	-2.9	-7.2
1234	1162	N6110a		381.1	549.1	298	0.02	735.4	738.1	2.7	0.4	40.2	38.6	-1.6	-4.0
1235	1163	N6111a		390.6	549.1	298	0.02	743.0	749.3	6.3	0.8	-	-	-	-
1236	1164	N6112a		363.1	549.1	298	1.0	737.6	743.7	6.1	0.8	34.8	33.8	-1.0	-3.0
1237	1165	N6113a		367.1	549.1	298	0.05	733.9	741.4	7.4	1.0	-	-	-	-
1238	1166	N6114a		364.6	549.1	298	0.06	721.5	732.4	11.0	1.5	-	-	-	-
1239	1167	N6115a		354.1	549.1	298	0.1	720.0	724.0	4.1	0.6	-	-	-	-
1240	1168	N6116a		361.9	535.1	298	1.01	723.0	732.8	9.7	1.3	34.9	35.9	1.0	2.8
1241	1169	N6117a		364.6	549.1	298	0.06	718.0	731.5	13.5	1.9	-	-	-	-
1242	1170	N6118a		367.1	549.1	298	0.06	716.0	733.9	17.9	2.5	-	-	-	-
1243	1171	N6201a		394.1	-	298	1.0	770.0	805.0	35.0	4.5	41.4	43.8	2.4	5.7
1244	1172	N6202a		407.6	-	298	1.0	738.0	809.5	71.5	9.7	-	-	-	-
1245	1173	N6203a		417.1	-	293	1.0	828.0	847.1	19.1	2.3	-	-	-	-
1246	1174	N6204a		419.1	-	293	1.0	828.0	829.3	1.3	0.2	-	-	-	-

Table S.13 – Comparison of experimental and simulated properties using LB combination rules (continued).

n_{sim}	n_{iso}	Code	Outlier	T_m [K]	T_b [K]	T [K]	P [bar]	ρ_{liq}[kg·m^{-3}] exp	sim	dev	err [%]	ΔH_{vap}[kJ·mol^{-1}] exp	sim	dev	err [%]
1247	1175	N6205a		466.1	-	298	1.0	-	-	-	-	60.9	60.3	-0.6	-0.9
1248	1176	N6206a		475.0	-	298	1.0	-	-	-	-	63.1	62.9	-0.2	-0.4
1249	1177	N7101a		-	-	298	1.0	761.5	776.7	15.2	2.0	-	-	-	-
1250	1178	N7102a		395.1	617.7	298	0.01	768.2	750.1	-18.1	-2.4	-	-	-	-
1251	1179	N7103a		-	-	293	1.0	788.2	773.9	-14.3	-1.8	-	-	-	-
1252	1180	N7104a		405.6	617.7	307	0.01	755.5	767.7	12.2	1.6	-	-	-	-
1253	1181	N7105a		412.6	617.7	312	0.01	752.7	754.5	1.8	0.2	-	-	-	-
1254	1182	N7106a		422.1	617.7	320	0.01	756.1	768.8	12.6	1.7	-	-	-	-
1255	1183	N7107a		415.1	617.7	293	1.0	762.9	775.1	12.2	1.6	-	-	-	-
1256	1184	N7108a		430.1	617.7	298	1.0	771.3	785.4	14.1	1.8	49.9	48.8	-1.1	-2.3
1257	1185	N7109a		-	-	298	1.0	-	-	-	-	42.1	40.3	-1.8	-4.4
1258	1186	N7110a		415.1	577.7	293	1.0	778.7	767.4	-11.3	-1.4	-	-	-	-
1259	1187	N7111a		383.1	577.7	298	0.02	744.5	758.3	13.8	1.8	-	-	-	-
1260	1188	N7112a		385.1	577.7	298	0.02	749.5	747.3	-2.2	-0.3	-	-	-	-
1261	1189	N7113a		380.1	577.7	298	0.03	729.5	739.2	9.7	1.3	-	-	-	-
1262	1190	N7114a		385.1	577.7	298	0.02	738.0	746.0	8.1	1.1	-	-	-	-
1263	1191	N7201a		417.1	-	298	1.0	-	-	-	-	45.3	48.1	2.8	6.2
1264	1192	N7202a		441.6	-	293	1.0	-	-	-	-	52.4	53.3	0.9	1.6
1265	1193	N7203a		497.1	-	298	1.0	-	-	-	-	67.1	67.1	-0.0	-0.1
1266	1194	N8101a		438.1	643.9	293	1.0	772.0	784.6	12.6	1.6	-	-	-	-
1267	1195	N8102a		452.8	643.9	298	1.01	780.0	794.0	14.0	1.8	55.1	53.4	-1.7	-3.1
1268	1196	N8103a		407.1	603.9	298	1.0	749.0	751.7	2.7	0.4	-	-	-	-
1269	1196	N8103b		407.1	603.9	308	0.01	-	-	-	-	46.3	41.6	-4.7	-10.2
1270	1197	N8104a		412.2	603.9	298	1.0	740.7	734.5	-6.2	-0.8	-	-	-	-
1271	1198	N8105a		432.0	602.3	298	1.0	755.7	758.2	2.5	0.3	49.4	46.5	-2.9	-5.8
1272	1199	N8106a		438.1	603.9	273	1.0	771.2	796.2	25.0	3.2	-	-	-	-
1273	1200	N8107a		399.6	603.9	313	1.0	749.0	739.7	-9.3	-1.2	-	-	-	-
1274	1201	N8108a		409.1	603.9	293	1.0	742.0	763.6	21.6	2.9	-	-	-	-
1275	1202	N8109a		-	-	298	1.0	742.0	759.2	17.2	2.3	-	-	-	-
1276	1203	N8201a		441.1	-	288	1.0	794.2	824.1	29.9	3.8	-	-	-	-
1277	1204	N8202a		457.1	-	288	1.0	848.5	862.9	14.4	1.7	-	-	-	-
1278	1205	N9101a		-	-	293	1.0	771.9	786.6	14.7	1.9	-	-	-	-
1279	1206	N9102a		-	-	298	1.0	789.2	802.3	13.1	1.7	-	-	-	-
1280	1207	N9103a		475.4	668.2	293	1.0	788.6	805.5	16.9	2.1	-	-	-	-
1281	1208	N9104a		453.9	628.2	298	1.0	767.5	772.7	5.2	0.7	-	-	-	-
1282	1209	N9105a		459.9	628.2	293	1.0	782.4	785.1	2.7	0.4	-	-	-	-
1283	1210	N9106a		429.6	628.2	298	1.0	752.9	765.4	12.5	1.7	46.2	48.9	2.7	5.9
1284	1211	N9107a		429.1	628.2	293	1.0	766.3	773.5	7.2	0.9	-	-	-	-
1285	1212	N9108a		432.8	628.2	293	1.0	759.3	774.4	15.1	2.0	-	-	-	-
1286	1213	N9109a		445.1	628.2	293	1.0	758.0	776.2	18.2	2.4	-	-	-	-
1287	1214	N9201a		438.9	-	293	1.0	800.0	810.5	10.5	1.3	-	-	-	-
1288	1215	N9202a		474.1	-	293	1.0	814.0	839.2	25.2	3.1	-	-	-	-
1289	1216	N9203a		531.6	-	298	1.0	-	-	-	-	75.5	75.5	-0.0	-0.0
1290	1217	N0101a		493.6	690.9	298	1.0	789.0	806.6	17.6	2.2	64.9	62.6	-2.3	-3.6
1291	1218	N0102a		461.1	650.9	298	1.0	766.9	773.9	7.0	0.9	-	-	-	-
1292	1219	N0103a		476.1	650.9	298	1.0	773.2	775.2	2.0	0.3	61.2	55.7	-5.5	-8.9
1293	1220	N0104a		475.1	650.9	298	1.0	774.3	779.6	5.3	0.7	-	-	-	-
1294	1221	N0105a		480.1	650.9	292	1.0	784.0	793.2	9.2	1.2	-	-	-	-

Table S.13 – Comparison of experimental and simulated properties using LB combination rules (continued).

n_{sim}	n_{iso}	Code	Outlier	T_m [K]	T_b [K]	T [K]	P [bar]	ρ_{liq}[kg·m^{-3}] exp	sim	dev	err [%]	ΔH_{vap}[kJ·mol^{-1}] exp	sim	dev	err [%]
1295	1222	N0106a		-	-	293	1.0	768.6	786.1	17.5	2.3	-	-	-	-
1296	1223	N0107a		464.1	650.9	293	1.0	768.7	784.6	15.9	2.1	-	-	-	-
1297	1224	N0201a		485.0	-	345	1.0	-	-	-	-	73.6	70.4	-3.2	-4.3
1298	1225	M2201a		494.3	761.0	354	0.004	1002.0	1006.1	4.1	0.4	-	-	-	-
1299	1226	M3201a		478.1	718.0	308	1.01	949.7	941.9	-7.8	-0.8	-	-	-	-
1300	1227	M3202a		486.1	-	356	1.01	963.2	949.0	-14.2	-1.5	-	-	-	-
1301	1227	M3202b	×	486.1	-	390	1.0	-	-	-	-	63.9	53.6	-10.3	-16.1
1302	1228	M4201a		439.2	658.0	298	1.01	936.7	926.6	-10.1	-1.1	50.7	49.0	-1.6	-3.2
1303	1229	M4202a		421.1	-	298	1.0	930.5	922.6	-7.9	-0.8	66.6	65.5	-1.1	-1.6
1304	1230	M4203a		478.1	-	277	1.0	942.0	947.2	5.2	0.6	-	-	-	-
1305	1230	M4203b		478.1	-	298	1.0	-	-	-	-	64.9	66.2	1.3	1.9
1306	1231	M4204a		489.1	-	393	1.0	885.0	888.9	3.9	0.4	-	-	-	-
1307	1232	M5201a		444.6	-	298	1.01	920.3	908.3	-12.0	-1.3	-	-	-	-
1308	1233	M5202a		-	-	298	1.0	-	-	-	-	67.1	68.1	1.0	1.5
1309	1234	M5203a		-	-	298	1.0	-	-	-	-	66.4	66.5	0.1	0.1
1310	1235	M5204a		-	-	298	1.0	-	-	-	-	69.8	70.0	0.2	0.3
1311	1236	M5205a	×	427.0	-	427	1.0	-	-	-	-	36.9	51.2	14.3	38.7
1312	1237	M6201a		459.1	-	298	1.0	906.4	899.8	-6.6	-0.7	-	-	-	-
1313	1238	M6202a		458.6	700.0	290	1.0	913.0	909.3	-3.7	-0.4	53.7	54.9	1.2	2.2
1314	1239	M6203a		502.1	700.0	298	1.0	896.0	914.3	18.3	2.0	76.1	69.9	-6.2	-8.1
1315	1240	M7201a		464.1	-	293	1.0	897.2	895.5	-1.7	-0.2	-	-	-	-
1316	1241	M7202a		458.8	-	298	1.0	896.2	895.0	-1.2	-0.1	-	-	-	-
1317	1242	M8201a		482.6	700.0	290	1.0	899.2	895.6	-3.6	-0.4	-	-	-	-
1318	1243	M9201a		484.1	-	293	1.0	876.4	884.8	8.4	1.0	-	-	-	-
1319	1244	S1201a		341.2	557.0	298	1.01	1924.9	1917.0	-7.9	-0.4	-	-	-	-
1320	1245	S1202a		264.1	411.5	260	1.0	1282.1	1284.8	2.6	0.2	21.9	24.7	2.8	12.7
1321	1246	S1301a	×	257.6	412.0	298	1.0	1775.5	1912.2	136.8	7.7	-	-	-	-
1322	1246	S1301b		257.6	412.0	244	1.0	-	-	-	-	24.0	28.2	4.2	17.4
1323	1247	S1302a		232.3	369.3	233	1.0	1408.0	1423.2	15.2	1.1	20.2	22.2	2.0	9.7
1324	1247	S1302b		232.3	369.3	298	10.77	1193.0	1238.0	45.0	3.8	-	-	-	-
1325	1248	S1303a		282.1	451.6	298	1.83	1367.0	1412.6	45.7	3.3	-	-	-	-
1326	1248	S1303b		282.1	451.6	267	1.0	-	-	-	-	26.1	28.4	2.3	8.9
1327	1249	S1304a		373.1	-	295	1.0	3196.9	3201.3	4.4	0.1	-	-	-	-
1328	1250	S1305a		363.1	-	298	1.0	1983.5	2002.4	18.9	1.0	-	-	-	-
1329	1251	S1401a		378.1	606.0	298	1.01	2002.1	2053.5	51.4	2.6	36.1	42.2	6.0	16.6
1330	1252	S1402a	×	215.3	340.1	215	1.0	1989.9	1896.5	-93.4	-4.7	17.5	20.2	2.7	15.7
1331	1252	S1402b		215.3	340.1	298	16.64	1536.1	1523.3	-12.8	-0.8	-	-	-	-
1332	1253	S1403a	×	295.9	478.0	298	1.0	2251.2	2375.0	123.8	5.5	25.0	32.1	7.1	28.5
1333	1254	S1404a	×	191.7	301.8	190	1.0	1527.8	1851.6	323.8	21.2	15.4	17.6	2.2	14.4
1334	1254	S1404b	×	191.7	301.8	298	36.66	840.9	1017.2	176.2	21.0	-	-	-	-
1335	1255	S1405a		243.4	384.9	243	1.0	1488.0	1476.2	-11.8	-0.8	20.1	21.6	1.5	7.4
1336	1255	S1405b		243.4	384.9	298	6.74	1307.0	1324.4	17.3	1.3	-	-	-	-
1337	1256	S1406a		297.0	471.2	298	1.06	1477.0	1510.4	33.4	2.3	24.9	28.1	3.2	13.1
1338	1257	S1407a	×	250.7	-	298	20.0	2046.8	2129.7	82.9	4.0	-	-	-	-
1339	1258	S1408a		269.1	426.1	269	1.0	1899.6	1844.0	-55.7	-2.9	23.1	26.3	3.2	13.9
1340	1258	S1408b		269.1	426.1	298	2.6	1810.0	1732.6	-77.4	-4.3	-	-	-	-
1341	1259	S2201a		356.1	-	283	1.0	1667.0	1652.9	-14.1	-0.8	-	-	-	-
1342	1260	S2202a		380.1	-	298	1.01	1727.0	1716.0	-11.0	-0.6	-	-	-	-

Table S.13 – Comparison of experimental and simulated properties using LB combination rules (continued).

n_{sim}	n_{iso}	Code	Outlier	T_m [K]	T_b [K]	T [K]	P [bar]	ρ_{liq}[kg·m^{-3}] exp	sim	dev	err [%]	ΔH_{vap}[kJ·mol^{-1}] exp	sim	dev	err [%]
1343	1260	S2202b		380.1	-	308	1.0	-	-	-	-	37.6	37.8	0.2	0.4
1344	1261	S2203a		344.6	-	298	1.0	1704.4	1689.1	-15.3	-0.9	-	-	-	-
1345	1262	S2204a		326.1	497.1	298	0.33	1167.5	1139.3	-28.2	-2.4	-	-	-	-
1346	1263	S2205a		413.1	-	288	1.0	2133.6	2133.5	-0.1	-0.0	-	-	-	-
1347	1264	S2206a		401.8	585.0	298	1.01	1200.9	1166.8	-34.1	-2.8	48.3	47.7	-0.6	-1.2
1348	1265	S2207a		423.1	-	293	1.0	1762.9	1737.6	-25.3	-1.4	54.1	54.5	0.4	0.7
1349	1266	S2208a		376.6	-	293	1.0	1104.0	1056.9	-47.1	-4.3	-	-	-	-
1350	1266	S2208b		376.6	-	288	1.0	-	-	-	-	44.1	42.1	-2.0	-4.5
1351	1267	S2209a		449.1	-	293	1.0	2196.7	2195.8	-0.9	-0.0	-	-	-	-
1352	1267	S2209b		449.1	-	288	1.0	-	-	-	-	57.0	62.7	5.7	9.9
1353	1268	S2210a	×	-	-	373	1.0	1366.0	1007.6	-358.4	-26.2	-	-	-	-
1354	1269	S2211a		444.1	678.2	298	1.01	1012.1	1005.1	-7.0	-0.7	59.6	60.1	0.5	0.9
1355	1270	S2301a	×	263.1	410.3	265	1.07	1188.8	1078.9	-109.9	-9.2	22.7	18.8	-3.9	-17.3
1356	1270	S2301b	×	263.1	410.3	298	3.61	1107.7	979.5	-128.3	-11.6	19.7	17.2	-2.6	-13.0
1357	1271	S2302a		304.9	478.9	298	1.0	1233.7	1194.0	-39.7	-3.2	26.0	25.2	-0.8	-3.2
1358	1272	S2303a		346.9	523.6	298	1.0	1369.2	1355.9	-13.3	-1.0	-	-	-	-
1359	1273	S2304a		362.0	555.0	298	0.07	1433.0	1440.1	7.1	0.5	-	-	-	-
1360	1274	S2305a		462.5	686.0	352	0.01	1354.2	1395.2	41.1	3.0	-	-	-	-
1361	1275	S2306a		481.1	-	323	1.0	1933.5	1981.8	48.3	2.5	-	-	-	-
1362	1276	S2307a		441.1	-	309	1.0	1369.3	1382.0	12.7	0.9	-	-	-	-
1363	1277	S2308a		419.1	-	298	1.0	1404.0	1400.8	-3.2	-0.2	-	-	-	-
1364	1278	S2309a	vap	-	-	571	1.0	1607.0	-	-	-	0.0	-	-	-
1365	1279	S2401a		365.6	-	293	1.0	2223.8	2218.8	-5.0	-0.2	-	-	-	-
1366	1280	S2402a		319.8	479.1	298	1.0	1406.6	1354.6	-52.0	-3.7	-	-	-	-
1367	1281	S2403a		-	-	283	1.0	1874.0	1873.6	-0.4	-0.0	-	-	-	-
1368	1282	S2404a	×	279.2	426.2	285	1.0	1353.9	1180.4	-173.5	-12.8	-	-	-	-
1369	1283	S2405a		361.1	535.0	298	1.0	1482.6	1481.4	-1.2	-0.1	-	-	-	-
1370	1284	S2406a		375.1	555.6	298	1.0	1529.8	1540.6	10.8	0.7	-	-	-	-
1371	1285	S2407a	×	327.6	-	298	1.0	2130.0	2024.4	-105.6	-5.0	-	-	-	-
1372	1286	S2408a		370.9	565.0	298	0.06	1499.1	1561.7	62.5	4.2	-	-	-	-
1373	1287	S2409a		447.1	-	298	1.0	2664.9	2702.5	37.6	1.4	-	-	-	-
1374	1288	S2410a	×	467.1	686.0	356	0.01	1473.5	1565.3	91.8	6.2	-	-	-	-
1375	1289	S2411a		406.1	-	298	1.0	1526.0	1510.0	-16.0	-1.0	-	-	-	-
1376	1290	S2412a	×	347.1	-	298	1.01	1382.4	1131.2	-251.2	-18.2	-	-	-	-
1377	1290	S2412b	×	347.1	-	289	1.0	-	-	-	-	45.9	36.3	-9.6	-21.0
1378	1291	S2501a	×	469.6	688.0	337	1.0	1612.6	1698.7	86.1	5.3	-	-	-	-
1379	1292	S2502a	×	344.9	491.2	298	1.0	1535.1	1407.0	-128.1	-8.3	35.9	38.2	2.3	6.5
1380	1293	S3201a		368.1	-	293	1.0	1495.0	1470.2	-24.8	-1.7	-	-	-	-
1381	1294	S3202a		390.1	-	293	1.0	1537.0	1552.1	15.1	1.0	-	-	-	-
1382	1295	S3203a		391.1	-	293	1.0	1531.0	1550.0	19.0	1.2	-	-	-	-
1383	1296	S3204a		416.4	-	293	1.0	1596.9	1587.7	-9.2	-0.6	-	-	-	-
1384	1297	S3205a		-	515.4	293	1.0	998.2	921.8	-76.4	-7.7	-	-	-	-
1385	1298	S3206a		-	-	293	1.0	1086.0	1038.8	-47.2	-4.3	-	-	-	-
1386	1299	S3207a		444.1	-	293	1.0	1904.0	1934.8	30.8	1.6	-	-	-	-
1387	1300	S3208a		359.1	-	288	1.0	1182.0	1108.4	-73.6	-6.2	-	-	-	-
1388	1301	S3209a		392.1	-	293	1.0	1150.0	1121.2	-28.8	-2.5	-	-	-	-
1389	1302	S3210a		411.1	-	296	1.0	1634.0	1593.0	-41.0	-2.5	-	-	-	-
1390	1303	S3211a		350.1	-	293	1.0	1028.8	1026.5	-2.3	-0.2	-	-	-	-

Table S.13 – Comparison of experimental and simulated properties using LB combination rules (continued).

n_{sim}	n_{iso}	Code	Outlier	T_m [K]	T_b [K]	T [K]	P [bar]	ρ_{liq}[kg·m^{-3}] exp	sim	dev	err [%]	ΔH_{vap}[kJ·mol^{-1}] exp	sim	dev	err [%]
1391	1304	S3212a	×	-	-	288	1.0	2170.0	2003.2	-166.8	-7.7	-	-	-	-
1392	1305	S3213a		400.1	-	293	1.0	1113.0	1087.1	-25.9	-2.3	-	-	-	-
1393	1306	S3214a		406.6	-	293	1.0	1103.0	1084.8	-18.2	-1.7	-	-	-	-
1394	1307	S3215a		438.1	-	293	1.0	1130.9	1121.4	-9.5	-0.8	-	-	-	-
1395	1308	S3216a		419.6	-	303	1.0	1558.5	1534.6	-23.9	-1.5	-	-	-	-
1396	1309	S3217a		395.3	-	293	1.0	1537.4	1586.8	49.4	3.2	-	-	-	-
1397	1310	S3218a		499.1	-	499	1.0	-	-	-	-	43.8	44.4	0.6	1.4
1398	1311	S3219a		365.6	-	293	1.0	1034.5	1005.6	-28.9	-2.8	-	-	-	-
1399	1312	S3220a		383.1	-	293	1.0	1462.3	1435.8	-26.5	-1.8	-	-	-	-
1400	1313	S3221a	×	418.6	596.0	298	1.0	1074.3	1028.9	-45.4	-4.2	44.2	57.2	13.0	29.4
1401	1314	S3222a		431.1	717.6	293	1.0	937.0	931.9	-5.1	-0.5	57.8	56.6	-1.2	-2.2
1402	1315	S3223a		432.6	717.6	298	1.01	956.5	916.7	-39.8	-4.2	-	-	-	-
1403	1316	S3224a		460.6	717.6	298	1.01	987.4	954.6	-32.8	-3.3	-	-	-	-
1404	1317	S3225a		365.1	-	298	1.0	1005.0	947.7	-57.3	-5.7	-	-	-	-
1405	1318	S3226a		397.6	564.0	298	1.01	960.2	953.5	-6.8	-0.7	45.2	39.7	-5.5	-12.1
1406	1319	S3301a		-	-	293	1.0	1708.4	1719.1	10.7	0.6	-	-	-	-
1407	1320	S3302a		469.1	-	287	1.0	2093.0	2103.7	10.7	0.5	-	-	-	-
1408	1321	S3303a		361.8	536.5	298	0.07	1255.2	1218.9	-36.4	-2.9	-	-	-	-
1409	1322	S3304a		458.1	-	293	1.0	1258.5	1301.0	42.5	3.4	-	-	-	-
1410	1323	S3305a		403.0	600.0	293	1.0	1236.0	1198.5	-37.5	-3.0	46.7	43.4	-3.3	-7.1
1411	1324	S3306a		405.1	-	293	1.0	1635.0	1625.5	-9.5	-0.6	-	-	-	-
1412	1325	S3307a		377.6	-	288	1.0	-	-	-	-	42.7	38.9	-3.8	-8.9
1413	1326	S3308a		393.1	-	291	1.0	1304.0	1332.8	28.8	2.2	-	-	-	-
1414	1327	S3309a		446.6	-	319	1.0	1382.6	1354.2	-28.4	-2.1	-	-	-	-
1415	1328	S3310a		449.1	633.0	298	0.87	1356.5	1339.3	-17.1	-1.3	-	-	-	-
1416	1328	S3310b		449.1	633.0	316	1.0	-	-	-	-	50.4	51.0	0.6	1.3
1417	1329	S3311a		457.1	647.0	293	1.0	1360.7	1346.7	-14.0	-1.0	-	-	-	-
1418	1330	S3312a		492.1	-	293	1.0	2136.4	2121.2	-15.2	-0.7	-	-	-	-
1419	1331	S3313a		492.1	-	293	1.0	2120.0	2131.2	11.2	0.5	-	-	-	-
1420	1332	S3314a		400.1	-	298	1.0	1240.0	1193.3	-46.7	-3.8	-	-	-	-
1421	1333	S3315a		489.1	-	293	1.0	1321.9	1284.2	-37.7	-2.9	-	-	-	-
1422	1334	S3316a		486.1	648.0	298	1.0	1315.9	1302.0	-13.9	-1.1	-	-	-	-
1423	1335	S3317a		-	-	303	1.0	-	-	-	-	31.8	31.4	-0.4	-1.4
1424	1336	S3318a		422.1	-	291	1.0	1167.7	1121.6	-46.1	-3.9	-	-	-	-
1425	1337	S3319a		478.3	691.0	293	1.0	1176.8	1221.6	44.8	3.8	-	-	-	-
1426	1338	S3401a		-	-	293	1.0	2077.2	2122.2	45.0	2.2	-	-	-	-
1427	1339	S3402a	×	460.6	-	285	1.0	1389.0	1488.2	99.2	7.1	-	-	-	-
1428	1340	S3403a		416.1	-	293	1.0	1377.4	1369.1	-8.3	-0.6	47.7	49.1	1.4	2.9
1429	1341	S3404a		358.5	-	288	1.0	-	-	-	-	41.9	35.9	-6.0	-14.2
1430	1342	S3405a		422.1	-	293	1.0	1435.0	1481.5	46.5	3.2	-	-	-	-
1431	1343	S3406a	×	295.1	-	298	1.0	1252.0	1102.2	-149.8	-12.0	-	-	-	-
1432	1344	S3407a	vap	348.1	-	298	1.0	0.0	-	-	-	44.8	-	-	-
1433	1345	S3501a		426.9	-	293	1.0	1487.4	1491.0	3.6	0.2	48.3	51.2	2.9	5.9
1434	1346	S3502a	×	316.6	-	293	1.0	1280.0	1167.6	-112.4	-8.8	-	-	-	-
1435	1347	S3503a	×	382.6	-	302	1.01	1482.8	1307.9	-174.9	-11.8	53.6	36.5	-17.1	-31.9
1436	1348	S4201a		448.1	-	298	1.01	1485.1	1478.3	-6.9	-0.5	-	-	-	-
1437	1349	S4202a		363.1	-	288	1.0	1053.0	1041.3	-11.7	-1.1	-	-	-	-
1438	1350	S4203a		394.6	-	281	1.0	1106.0	1111.3	5.3	0.5	-	-	-	-

Table S.13 – Comparison of experimental and simulated properties using LB combination rules (continued).

n_{sim}	n_{iso}	Code	Outlier	T_m [K]	T_b [K]	T [K]	P [bar]	ρ_{liq} [kg·m^{-3}] exp	sim	dev	err [%]	ΔH_{vap} [kJ·mol^{-1}] exp	sim	dev	err [%]
1439	1351	S4204a		388.1	-	298	1.0	1055.4	1047.8	-7.6	-0.7	-	-	-	-
1440	1352	S4205a		414.1	-	298	1.0	1068.0	1053.8	-14.2	-1.3	-	-	-	-
1441	1353	S4206a		388.3	-	293	1.0	1088.3	1081.9	-6.4	-0.6	-	-	-	-
1442	1354	S4207a		380.6	-	293	1.0	989.5	961.8	-27.7	-2.8	-	-	-	-
1443	1355	S4208a		400.6	-	273	1.0	1385.2	1351.3	-33.9	-2.4	-	-	-	-
1444	1356	S4209a	×	408.1	620.0	298	1.0	1096.9	978.4	-118.5	-10.8	-	-	-	-
1445	1357	S4210a	×	418.6	-	298	1.0	-	-	-	-	48.7	58.3	9.6	19.7
1446	1358	S4211a		433.1	-	293	1.0	1027.2	998.6	-28.6	-2.8	-	-	-	-
1447	1359	S4212a		455.1	-	293	1.0	1023.3	1003.2	-20.0	-2.0	-	-	-	-
1448	1360	S4213a	×	407.1	-	298	1.01	883.8	888.2	4.4	0.5	46.7	55.6	8.9	19.0
1449	1361	S4214a		438.6	-	298	1.01	932.6	889.1	-43.5	-4.7	-	-	-	-
1450	1361	S4214b		438.6	-	308	1.0	-	-	-	-	63.3	66.3	3.0	4.7
1451	1362	S4215a		442.6	-	293	1.0	914.0	903.4	-10.6	-1.2	61.0	58.7	-2.3	-3.8
1452	1363	S4216a		478.1	-	285	1.0	967.0	985.8	18.8	1.9	-	-	-	-
1453	1364	S4217a		389.1	-	298	1.0	957.0	910.3	-46.7	-4.9	-	-	-	-
1454	1365	S4218a		403.1	588.8	293	1.0	938.0	936.1	-1.9	-0.2	-	-	-	-
1455	1366	S4219a		392.1	588.8	298	1.01	915.9	940.9	25.0	2.7	46.2	41.7	-4.5	-9.8
1456	1367	S4220a		408.1	588.8	298	1.0	925.4	893.4	-31.9	-3.4	48.2	48.4	0.2	0.3
1457	1368	S4301a		-	-	293	1.0	1598.5	1625.7	27.2	1.7	-	-	-	-
1458	1369	S4302a		406.8	-	293	1.0	1179.6	1240.0	60.4	5.1	-	-	-	-
1459	1370	S4303a		406.8	-	293	1.0	1223.6	1266.5	42.9	3.5	-	-	-	-
1460	1371	S4304a		472.1	-	333	1.0	1496.9	1565.0	68.1	4.5	-	-	-	-
1461	1372	S4305a		405.6	-	298	1.0	1075.0	1107.1	32.1	3.0	-	-	-	-
1462	1373	S4306a		417.4	618.0	293	1.0	1158.5	1123.4	-35.1	-3.0	49.5	46.3	-3.2	-6.6
1463	1374	S4307a		418.1	-	293	1.0	1178.0	1126.8	-51.2	-4.3	-	-	-	-
1464	1375	S4308a		441.6	-	293	1.0	1503.2	1484.7	-18.5	-1.2	-	-	-	-
1465	1376	S4309a		435.6	-	293	1.0	1514.0	1491.3	-22.7	-1.5	-	-	-	-
1466	1377	S4310a	×	-	-	291	1.0	1412.3	1514.7	102.4	7.2	-	-	-	-
1467	1378	S4311a		393.1	-	293	1.0	1091.2	1058.8	-32.4	-3.0	-	-	-	-
1468	1378	S4311b		393.1	-	288	1.0	-	-	-	-	41.9	41.8	-0.1	-0.2
1469	1379	S4312a		452.1	-	286	1.0	1817.3	1807.0	-10.3	-0.6	-	-	-	-
1470	1380	S4313a		451.6	666.0	333	0.01	1176.5	1132.7	-43.8	-3.7	-	-	-	-
1471	1381	S4314a		458.1	-	293	1.0	1845.2	1809.6	-35.6	-1.9	-	-	-	-
1472	1382	S4315a		422.1	-	283	1.0	1027.5	1019.4	-8.1	-0.8	-	-	-	-
1473	1383	S4316a	×	417.6	584.0	298	1.0	-	-	-	-	44.3	56.9	12.6	28.5
1474	1384	S4317a		433.1	-	296	1.0	1082.6	1052.8	-29.8	-2.8	-	-	-	-
1475	1385	S4318a		461.1	-	288	1.0	1108.0	1079.7	-28.3	-2.6	-	-	-	-
1476	1386	S4319a		452.1	-	336	1.0	-	-	-	-	60.0	57.6	-2.4	-4.0
1477	1387	S4320a		485.1	664.0	356	1.0	1046.1	1111.3	65.2	6.2	-	-	-	-
1478	1388	S4321a		479.6	-	293	1.0	1102.1	1117.8	15.7	1.4	-	-	-	-
1479	1389	S4322a		404.1	-	293	1.0	1051.1	996.6	-54.5	-5.2	-	-	-	-
1480	1390	S4401a		437.1	-	293	1.0	1395.6	1445.6	50.0	3.6	-	-	-	-
1481	1391	S4402a		-	-	293	1.0	1328.2	1323.9	-4.3	-0.3	-	-	-	-
1482	1392	S4403a		428.1	-	293	1.0	1282.7	1278.1	-4.6	-0.4	50.6	52.0	1.4	2.7
1483	1393	S4404a		479.1	-	293	1.0	1933.3	1962.8	29.5	1.5	-	-	-	-
1484	1394	S4405a		467.1	-	293	1.0	1899.1	1904.1	5.0	0.3	-	-	-	-
1485	1395	S4406a		373.1	-	293	1.0	1176.5	1142.5	-34.0	-2.9	-	-	-	-
1486	1396	S4407a		-	-	288	1.0	-	-	-	-	55.1	51.4	-3.7	-6.8

Table S.13 – Comparison of experimental and simulated properties using LB combination rules (continued).

n_{sim}	n_{iso}	Code	Outlier	T_m [K]	T_b [K]	T [K]	P [bar]	ρ_{liq}[kg·m^{-3}] exp	sim	dev	err [%]	ΔH_{vap}[kJ·mol^{-1}] exp	sim	dev	err [%]
1487	1397	S4408a		402.1	-	293	1.0	1225.0	1217.8	-7.2	-0.6	-	-	-	-
1488	1398	S4409a		393.1	-	288	1.0	-	-	-	-	56.4	54.7	-1.7	-3.1
1489	1399	S4501a		440.6	-	293	1.0	1383.6	1395.0	11.4	0.8	51.0	54.4	3.4	6.7
1490	1400	S4502a		334.1	-	293	1.0	1194.0	1125.6	-68.4	-5.7	-	-	-	-
1491	1401	S5201a		396.5	-	293	1.0	1052.3	1046.4	-5.9	-0.6	-	-	-	-
1492	1402	S5202a		392.6	-	298	1.0	-	-	-	-	43.6	41.4	-2.2	-5.1
1493	1403	S5203a		413.6	-	293	1.0	952.6	956.2	3.6	0.4	-	-	-	-
1494	1404	S5204a		482.1	-	293	1.0	1007.1	983.1	-24.0	-2.4	-	-	-	-
1495	1405	S5205a		423.4	-	299	1.0	882.0	893.8	11.8	1.3	-	-	-	-
1496	1406	S5206a		397.6	-	298	1.0	837.0	861.3	24.3	2.9	-	-	-	-
1497	1407	S5207a		436.6	-	298	1.01	880.8	885.3	4.4	0.5	57.0	57.9	0.9	1.6
1498	1408	S5208a		446.1	-	298	1.0	893.2	875.2	-18.0	-2.0	-	-	-	-
1499	1409	S5209a		455.1	-	293	1.0	900.5	891.8	-8.7	-1.0	-	-	-	-
1500	1410	S5210a		408.4	-	293	1.0	916.5	901.5	-15.0	-1.6	-	-	-	-
1501	1411	S5211a		430.1	614.5	296	1.0	923.0	898.5	-24.5	-2.6	-	-	-	-
1502	1412	S5212a		418.1	614.5	298	1.01	899.7	879.4	-20.3	-2.3	50.1	45.4	-4.7	-9.3
1503	1413	S5213a		404.1	614.5	298	1.01	896.6	856.8	-39.8	-4.4	-	-	-	-
1504	1414	S5214a		424.5	615.2	298	1.01	907.9	885.2	-22.6	-2.5	52.1	52.4	0.3	0.5
1505	1415	S5301a		-	-	293	1.0	1108.5	1067.1	-41.4	-3.7	-	-	-	-
1506	1416	S5302a	×	503.1	-	298	1.0	1341.6	1204.3	-137.3	-10.2	-	-	-	-
1507	1417	S5303a		423.6	-	293	1.0	1088.8	1072.6	-16.2	-1.5	-	-	-	-
1508	1418	S5304a		420.1	-	293	1.0	1079.3	1061.9	-17.4	-1.6	-	-	-	-
1509	1419	S5305a		434.1	-	293	1.0	1104.0	1086.6	-17.4	-1.6	-	-	-	-
1510	1420	S5306a		435.1	-	293	1.0	1108.6	1089.2	-19.4	-1.8	-	-	-	-
1511	1421	S5307a		447.1	-	293	1.0	1129.3	1110.6	-18.7	-1.7	-	-	-	-
1512	1422	S5308a		441.1	-	293	1.0	1452.8	1410.0	-42.8	-2.9	-	-	-	-
1513	1423	S5309a		433.1	-	293	1.0	1413.5	1376.6	-36.9	-2.6	-	-	-	-
1514	1424	S5310a		452.1	-	291	1.0	1412.3	1407.8	-4.5	-0.3	-	-	-	-
1515	1425	S5311a		459.6	-	298	1.0	1400.0	1426.0	26.0	1.9	-	-	-	-
1516	1426	S5312a		-	-	288	1.0	-	-	-	-	44.3	44.9	0.6	1.3
1517	1427	S5313a		-	-	288	1.0	-	-	-	-	47.3	48.2	0.9	2.0
1518	1428	S5314a		-	-	293	1.0	-	-	-	-	43.9	46.2	2.3	5.3
1519	1429	S5315a		519.0	738.0	308	1.0	1120.1	1134.6	14.5	1.3	-	-	-	-
1520	1430	S5316a		444.1	-	293	1.0	1075.7	1044.0	-31.7	-2.9	-	-	-	-
1521	1431	S5317a		428.1	-	288	1.0	1059.6	1041.8	-17.8	-1.7	-	-	-	-
1522	1432	S5318a		444.9	642.0	333	0.01	1035.7	1013.9	-21.8	-2.1	45.6	48.3	2.8	6.0
1523	1433	S5319a	×	426.0	588.0	293	1.0	938.4	1093.5	155.1	16.5	-	-	-	-
1524	1434	S5320a		415.6	-	288	1.0	1013.9	980.6	-33.3	-3.3	-	-	-	-
1525	1435	S5321a		416.1	-	292	1.0	1007.4	976.9	-30.5	-3.0	-	-	-	-
1526	1435	S5321b		416.1	-	298	1.0	-	-	-	-	50.3	48.9	-1.4	-2.9
1527	1436	S5401a		456.6	-	293	1.0	1240.1	1246.9	6.8	0.6	-	-	-	-
1528	1437	S5402a		487.6	-	293	1.0	1796.6	1809.3	12.7	0.7	-	-	-	-
1529	1438	S5403a		-	-	288	1.0	-	-	-	-	54.5	54.4	-0.1	-0.1
1530	1439	S5501a		448.1	-	298	1.0	1291.1	1312.6	21.5	1.7	-	-	-	-
1531	1440	S5502a		460.1	-	293	1.0	1322.1	1333.9	11.8	0.9	-	-	-	-
1532	1441	S6201a	×	-	-	293	1.0	1250.0	1455.5	205.5	16.4	-	-	-	-
1533	1442	S6202a		431.3	-	293	1.0	1024.1	1031.1	7.0	0.7	-	-	-	-
1534	1443	S6203a		441.0	606.0	293	1.0	938.7	939.2	0.5	0.1	-	-	-	-

Table S.13 – Comparison of experimental and simulated properties using LB combination rules (continued).

n_{sim}	n_{iso}	Code	Outlier	T_m [K]	T_b [K]	T [K]	P [bar]	ρ_{liq}[kg·m^{-3}] exp	sim	dev	err [%]	ΔH_{vap}[kJ·mol^{-1}] exp	sim	dev	err [%]
1535	1444	S6204a	×	436.1	-	298	1.01	880.4	879.0	-1.3	-0.2	52.5	64.9	12.4	23.7
1536	1445	S6205a		472.1	-	293	1.0	890.7	888.6	-2.1	-0.2	-	-	-	-
1537	1446	S6206a		410.6	637.8	293	1.0	879.0	854.3	-24.7	-2.8	-	-	-	-
1538	1447	S6207a		423.1	637.8	298	1.01	880.8	853.4	-27.4	-3.1	-	-	-	-
1539	1448	S6208a		433.1	637.8	293	1.0	890.0	876.4	-13.6	-1.5	-	-	-	-
1540	1449	S6209a		444.5	633.9	298	1.01	896.2	883.0	-13.2	-1.5	56.6	56.9	0.3	0.6
1541	1450	S6301a		424.6	-	293	1.0	1031.5	1023.5	-8.0	-0.8	-	-	-	-
1542	1451	S6302a		453.1	-	293	1.0	1065.6	1059.9	-5.7	-0.5	-	-	-	-
1543	1452	S6303a		456.1	-	293	1.0	1070.4	1062.5	-7.9	-0.7	-	-	-	-
1544	1453	S6304a		457.1	-	293	1.0	1075.6	1065.7	-9.9	-0.9	-	-	-	-
1545	1454	S6305a		436.1	-	293	1.0	1326.3	1313.1	-13.2	-1.0	-	-	-	-
1546	1455	S6306a		450.1	-	293	1.0	1327.3	1329.0	1.7	0.1	-	-	-	-
1547	1456	S6307a		465.1	-	293	1.0	1354.0	1349.7	-4.3	-0.3	-	-	-	-
1548	1457	S6308a		-	-	288	1.0	-	-	-	-	54.9	53.1	-1.8	-3.2
1549	1458	S6309a		-	-	296	1.0	1086.1	1099.9	13.8	1.3	-	-	-	-
1550	1459	S6310a		460.1	-	293	1.0	1103.0	1100.2	-2.8	-0.3	-	-	-	-
1551	1460	S6311a		489.1	-	293	1.0	1136.0	1112.6	-23.4	-2.1	-	-	-	-
1552	1461	S6312a		440.1	-	293	1.0	998.0	970.0	-28.0	-2.8	-	-	-	-
1553	1462	S6313a	×	450.6	-	298	1.0	-	-	-	-	55.9	66.2	10.3	18.5
1554	1463	S6314a		547.6	-	298	1.0	1090.0	1105.6	15.6	1.4	-	-	-	-
1555	1464	S6315a		450.6	-	298	1.0	1021.7	1013.4	-8.3	-0.8	-	-	-	-
1556	1465	S6316a		453.9	643.0	283	1.0	1036.8	1019.9	-16.9	-1.6	-	-	-	-
1557	1466	S6317a		469.1	-	293	1.0	1051.1	1028.9	-22.2	-2.1	-	-	-	-
1558	1467	S6318a		431.1	-	293	1.0	970.2	936.7	-33.5	-3.4	-	-	-	-
1559	1468	S6319a		429.4	607.3	293	1.0	974.0	942.8	-31.2	-3.2	52.7	50.3	-2.4	-4.6
1560	1469	S6401a		466.6	-	293	1.0	1182.0	1188.6	6.6	0.6	-	-	-	-
1561	1470	S6402a		495.6	-	293	1.0	1698.7	1722.5	23.8	1.4	-	-	-	-
1562	1471	S6403a		482.9	-	293	1.0	1146.6	1147.3	0.7	0.1	-	-	-	-
1563	1472	S6404a		482.9	-	298	1.0	1169.0	1159.9	-9.1	-0.8	-	-	-	-
1564	1473	S6501a		461.1	-	293	1.0	1263.6	1276.0	12.4	1.0	-	-	-	-
1565	1474	S6502a		477.1	-	293	1.0	1277.8	1290.5	12.7	1.0	-	-	-	-
1566	1475	S6503a		375.1	-	295	1.0	1026.8	1085.5	58.7	5.7	-	-	-	-
1567	1476	S7201a		430.1	-	298	1.0	-	-	-	-	47.7	49.1	1.4	2.9
1568	1477	S7202a		462.6	-	293	1.0	860.0	880.7	20.7	2.4	-	-	-	-
1569	1478	S7203a		433.1	-	298	1.0	898.0	893.3	-4.7	-0.5	-	-	-	-
1570	1479	S7204a		420.2	659.3	320	0.01	-	-	-	-	47.7	47.1	-0.6	-1.2
1571	1480	S7205a		444.6	659.3	298	1.0	874.6	855.8	-18.9	-2.2	-	-	-	-
1572	1481	S7301a		456.1	-	293	1.0	1025.2	1024.2	-1.0	-0.1	-	-	-	-
1573	1482	S7302a		457.1	-	293	1.0	1025.3	1020.5	-4.8	-0.5	-	-	-	-
1574	1483	S7303a		464.4	-	293	1.0	1032.3	1029.7	-2.6	-0.2	-	-	-	-
1575	1484	S7304a		449.4	-	293	1.0	1037.0	1040.8	3.8	0.4	-	-	-	-
1576	1485	S7305a		459.1	-	293	1.0	1276.0	1287.0	11.0	0.9	-	-	-	-
1577	1486	S7306a		464.1	-	291	1.0	1226.0	1285.0	59.0	4.8	-	-	-	-
1578	1487	S7307a		499.9	-	293	1.0	1308.5	1299.8	-8.7	-0.7	-	-	-	-
1579	1488	S7308a		-	-	288	1.0	-	-	-	-	54.6	58.3	3.7	6.7
1580	1489	S7309a		-	-	288	1.0	-	-	-	-	56.8	62.7	5.9	10.4
1581	1490	S7310a		455.1	-	287	1.0	995.0	1007.4	12.4	1.2	-	-	-	-
1582	1491	S7311a		459.1	-	293	1.0	983.5	978.2	-5.3	-0.5	-	-	-	-

Table S.13 – Comparison of experimental and simulated properties using LB combination rules (continued).

n_{sim}	n_{iso}	Code	Outlier	T_m [K]	T_b [K]	T [K]	P [bar]	ρ_{liq}[kg·m^{-3}] exp	sim	dev	err [%]	ΔH_{vap}[kJ·mol^{-1}] exp	sim	dev	err [%]
1583	1492	S7312a		460.1	-	293	1.0	994.1	986.5	-7.6	-0.8	-	-	-	-
1584	1493	S7313a		464.1	-	293	1.0	1012.0	990.8	-21.2	-2.1	-	-	-	-
1585	1494	S7314a		478.9	-	293	1.0	1011.1	997.1	-14.0	-1.4	-	-	-	-
1586	1495	S7315a		438.1	621.0	293	1.0	949.0	925.7	-23.3	-2.5	-	-	-	-
1587	1496	S7316a		442.2	-	298	1.0	-	-	-	-	55.6	53.4	-2.2	-3.9
1588	1497	S7401a		464.2	-	293	1.0	1062.3	1131.3	69.0	6.5	-	-	-	-
1589	1498	S7501a		490.1	-	293	1.0	1231.4	1253.3	21.9	1.8	-	-	-	-
1590	1499	S8201a		458.1	-	289	1.0	910.7	929.8	19.1	2.1	-	-	-	-
1591	1500	S8202a		463.1	-	298	1.0	826.0	865.1	39.1	4.7	-	-	-	-
1592	1501	S8203a		481.1	679.3	293	1.0	887.8	883.6	-4.2	-0.5	-	-	-	-
1593	1502	S8301a		475.6	-	298	1.0	958.9	954.1	-4.8	-0.5	-	-	-	-
1594	1503	S8302a	×	-	-	298	1.0	-	-	-	-	61.9	76.3	14.4	23.3
1595	1504	S8303a		460.1	-	293	1.0	975.6	961.8	-13.8	-1.4	-	-	-	-
1596	1505	S8304a		471.1	-	289	1.0	984.7	978.9	-5.8	-0.6	-	-	-	-
1597	1506	S8305a		446.1	-	298	1.0	980.0	967.4	-12.6	-1.3	-	-	-	-
1598	1507	S8306a		469.0	-	298	1.0	967.1	976.0	8.9	0.9	-	-	-	-
1599	1508	S8307a		494.6	-	298	1.0	989.0	980.5	-8.5	-0.9	-	-	-	-
1600	1509	S8308a		465.7	641.2	298	1.0	-	-	-	-	59.5	57.8	-1.8	-2.9
1601	1510	S8401a		495.1	-	293	1.0	1073.2	1090.2	17.0	1.6	-	-	-	-
1602	1511	S9301a		-	-	291	1.0	970.0	970.4	0.4	0.0	-	-	-	-
1603	1512	S9302a		474.1	-	291	1.0	964.8	969.7	4.9	0.5	-	-	-	-
1604	1513	S9303a		497.1	-	293	1.0	966.1	966.0	-0.1	-0.0	-	-	-	-
1605	1514	S9304a		510.6	-	293	1.0	973.5	971.8	-1.7	-0.2	-	-	-	-
1606	1515	S0201a		473.7	-	293	1.0	922.0	928.4	6.4	0.7	75.3	79.0	3.7	4.9
1607	1516	S0301a		-	-	297	1.0	1191.2	1258.2	67.0	5.6	-	-	-	-

Table S.14: Experimental and simulated properties of the 1607 compounds/P,T-points considered in the simulations using WH combination rules.

n_{sim}	n_{iso}	Code	Outlier	T_m	T_b	T	P	ρ_{liq}[kg·m^{-3}]				ΔH_{vap}[kJ·mol^{-1}]			
				[K]	[K]	[K]	[bar]	exp	sim	dev	err [%]	exp	sim	dev	err [%]
1	1	A1001a		111.7	190.6	91	0.12	451.0	462.1	11.1	2.5	9.2	8.2	-1.1	-11.4
2	1	A1001b		111.7	190.6	102	0.41	436.1	444.2	8.2	1.9	8.8	7.9	-0.9	-10.6
3	1	A1001c		111.7	190.6	112	1.01	422.0	427.4	5.4	1.3	-	-	-	-
4	1	A1001d		111.7	190.6	112	1.01	-	-	-	-	8.5	7.7	-0.8	-9.5
5	2	A2001a		184.6	305.3	189	1.26	526.6	529.2	2.6	0.5	-	-	-	-
6	2	A2001b		184.6	305.3	266	20.88	409.4	422.7	13.3	3.2	9.7	12.3	2.6	26.6
7	2	A2001c		184.6	305.3	185	1.01	-	-	-	-	14.8	14.9	0.2	1.1
8	3	A3001a		231.1	369.8	230	1.0	582.1	588.2	6.1	1.1	18.8	19.0	0.2	1.2
9	3	A3001b		231.1	369.8	298	9.78	493.1	506.3	13.2	2.7	-	-	-	-
10	4	A4001a		261.4	407.8	260	19.85	597.9	609.7	11.8	2.0	-	-	-	-
11	4	A4001b		261.4	407.8	300	25.33	553.0	568.7	15.7	2.8	-	-	-	-
12	4	A4001c		261.4	407.8	265	1.0	-	-	-	-	22.4	21.7	-0.7	-3.2
13	5	A4002a		272.6	425.1	273	1.0	601.0	615.0	14.0	2.3	-	-	-	-
14	5	A4002b		272.6	425.1	300	9.93	572.5	588.6	16.2	2.8	-	-	-	-
15	5	A4002c		272.6	425.1	264	1.0	-	-	-	-	23.1	23.1	-0.1	-0.2
16	6	A5001a		282.6	433.8	282	0.99	603.7	621.9	18.2	3.0	23.6	23.8	0.1	0.5
17	6	A5001b		282.6	433.8	298	1.76	586.0	606.0	20.0	3.4	-	-	-	-
18	7	A5002a		301.0	460.4	298	0.92	616.0	631.2	15.2	2.5	25.5	25.5	0.0	0.1
19	8	A5003a		309.2	469.7	298	1.01	621.1	641.8	20.6	3.3	26.6	26.7	0.1	0.4
20	9	A6001a		322.9	489.0	298	1.01	644.4	665.9	21.5	3.3	28.8	28.3	-0.5	-1.7
21	10	A6002a		331.1	500.0	298	1.01	658.6	672.2	13.7	2.1	30.0	29.5	-0.5	-1.7
22	11	A6003a		336.4	504.4	298	1.01	659.9	677.2	17.3	2.6	30.9	30.4	-0.5	-1.5
23	12	A6004a		333.4	497.7	298	1.01	648.5	669.7	21.2	3.3	30.4	30.4	-0.1	-0.2
24	13	A6005a		341.9	507.6	298	1.0	656.1	679.4	23.3	3.6	31.8	31.5	-0.2	-0.7
25	14	A7001a		354.0	531.2	298	0.13	687.1	706.5	19.4	2.8	32.4	32.7	0.3	0.9
26	15	A7002a		359.2	536.4	298	0.11	686.9	710.9	24.0	3.5	34.3	33.3	-1.0	-2.9
27	16	A7003a		352.3	520.5	298	0.14	673.1	694.3	21.2	3.2	33.4	33.0	-0.3	-1.0
28	17	A7004a		362.9	537.3	298	0.09	691.1	707.4	16.3	2.4	35.0	34.4	-0.6	-1.8
29	18	A7005a		353.6	519.8	298	0.13	667.9	690.9	23.0	3.4	33.6	33.8	0.1	0.4
30	19	A7006a		366.6	527.0	298	0.08	695.1	710.4	15.3	2.2	35.2	35.1	-0.1	-0.4
31	20	A7007a		365.0	535.2	298	0.08	683.9	705.1	21.1	3.1	35.4	35.1	-0.3	-0.7
32	21	A7008a		363.2	530.4	298	0.09	674.0	699.6	25.7	3.8	35.2	35.0	-0.2	-0.4
33	22	A7009a		371.6	540.2	298	0.06	682.0	707.7	25.7	3.8	36.5	36.3	-0.2	-0.7
34	23	A8001a		387.9	573.5	298	0.04	722.1	744.5	22.4	3.1	37.6	37.9	0.2	0.7
35	24	A8002a		383.0	563.5	298	0.04	712.0	736.2	24.2	3.4	-	-	-	-
36	25	A8003a		372.4	543.8	298	1.01	688.0	711.2	23.2	3.4	35.2	36.2	1.0	2.8
37	26	A8004a		386.6	566.4	298	0.04	715.9	732.8	16.9	2.4	37.7	38.2	0.5	1.3
38	27	A8005a		391.4	576.5	298	0.03	724.0	746.4	22.4	3.1	38.0	38.3	0.3	0.8
39	28	A8006a		385.1	562.0	298	0.04	707.1	731.9	24.8	3.5	37.5	38.1	0.6	1.6
40	29	A8007a		380.0	549.8	298	0.05	692.1	719.5	27.4	4.0	37.3	37.6	0.3	0.9
41	30	A8008a		388.8	567.0	298	0.03	711.1	732.4	21.3	3.0	38.5	38.9	0.4	1.0
42	31	A8009a		390.9	568.8	298	0.03	715.9	735.3	19.4	2.7	39.0	39.1	0.1	0.1
43	32	A8010a		388.8	563.5	298	0.03	708.1	728.8	20.7	2.9	38.8	39.0	0.2	0.5
44	33	A8011a		382.6	553.5	298	1.0	696.4	720.2	23.8	3.4	37.8	38.4	0.6	1.6
45	34	A8012a		382.3	550.0	298	0.04	690.0	715.5	25.4	3.7	37.9	38.4	0.5	1.2
46	35	A8013a		391.7	565.5	298	0.03	710.1	731.4	21.3	3.0	39.7	39.7	-0.0	-0.0

Table S.14 – Comparison of experimental and simulated properties using WH combination rules (continued).

n_{sim}	n_{iso}	Code	Outlier	T_m [K]	T_b [K]	T [K]	P [bar]	ρ_{liq}[kg·m^{-3}] exp	sim	dev	err [%]	ΔH_{vap}[kJ·mol^{-1}] exp	sim	dev	err [%]
47	36	A8014a		390.9	561.7	298	0.03	713.0	726.4	13.4	1.9	39.7	39.9	0.2	0.6
48	37	A8015a		392.1	563.7	298	0.03	702.0	728.1	26.1	3.7	39.9	39.8	-0.1	-0.2
49	38	A8016a		390.8	559.6	298	0.03	696.0	721.8	25.8	3.7	39.7	39.7	-0.0	-0.1
50	39	A8017a		398.8	568.7	298	1.01	698.9	730.0	31.1	4.5	41.0	41.0	-0.0	-0.0
51	40	A9001a		413.4	607.5	298	1.0	753.0	777.9	24.9	3.3	41.2	41.6	0.4	1.1
52	41	A9002a		395.4	574.6	298	0.03	716.1	731.0	15.0	2.1	38.5	38.9	0.4	1.1
53	42	A9003a		414.7	607.5	301	0.01	733.0	770.5	37.6	5.1	-	-	-	-
54	42	A9003b		414.7	607.5	298	1.0	-	-	-	-	41.8	42.8	1.0	2.4
55	43	A9004a		406.2	592.6	298	0.02	735.1	753.6	18.5	2.5	40.8	40.8	-0.0	-0.0
56	44	A9005a		417.9	582.1	298	1.0	750.8	773.6	22.8	3.0	42.7	42.5	-0.2	-0.6
57	45	A9006a		413.6	582.1	298	1.0	741.4	766.3	24.9	3.4	42.2	42.5	0.3	0.6
58	46	A9007a		410.8	582.1	298	1.0	734.5	760.0	25.5	3.5	42.1	42.3	0.2	0.5
59	47	A9008a		407.0	582.1	298	0.02	731.1	752.1	21.0	2.9	41.7	41.3	-0.4	-0.9
60	48	A9009a		406.8	582.1	298	0.02	725.1	753.1	28.1	3.9	41.7	42.1	0.4	1.0
61	49	A9010a		403.8	582.1	298	0.02	720.1	743.5	23.5	3.3	41.1	41.1	-0.0	-0.1
62	50	A9011a		399.7	582.1	298	0.02	713.1	736.3	23.3	3.3	40.7	40.7	-0.0	-0.0
63	51	A9012a		397.2	569.8	298	0.02	707.1	731.7	24.5	3.5	40.2	40.8	0.6	1.5
64	52	A9013a		409.9	582.1	298	0.01	733.9	748.9	15.0	2.0	42.3	42.0	-0.3	-0.8
65	53	A9014a		412.2	582.1	298	1.0	735.4	754.8	19.4	2.6	42.7	42.8	0.1	0.3
66	54	A9015a		404.5	582.1	298	0.02	718.1	739.8	21.8	3.0	41.4	41.9	0.5	1.2
67	55	A9016a		419.3	610.0	308	0.03	727.9	765.4	37.5	5.1	-	-	-	-
68	55	A9016b		419.3	610.0	298	1.0	-	-	-	-	42.6	43.0	0.4	1.0
69	56	A9017a		413.8	582.1	298	1.0	737.1	761.9	24.8	3.4	42.9	42.9	0.0	0.1
70	57	A9018a		408.4	582.1	298	0.01	721.0	749.0	28.0	3.9	42.2	42.8	0.6	1.5
71	58	A9019a		410.4	582.1	306	0.01	715.9	744.3	28.4	4.0	-	-	-	-
72	58	A9019b		410.4	582.1	298	1.0	-	-	-	-	42.6	42.6	-0.0	-0.0
73	59	A9020a		405.8	576.7	298	0.02	707.1	737.4	30.2	4.3	42.3	42.2	-0.1	-0.3
74	60	A9021a		413.6	582.1	303	0.01	732.6	750.4	17.8	2.4	-	-	-	-
75	60	A9021b		413.6	582.1	298	1.0	-	-	-	-	43.6	43.4	-0.2	-0.4
76	61	A9022a		411.2	582.1	300	0.01	727.4	747.4	20.0	2.8	-	-	-	-
77	61	A9022b		411.2	582.1	298	1.0	-	-	-	-	43.2	43.3	0.1	0.2
78	62	A9023a		413.8	582.1	302	0.01	723.9	748.7	24.8	3.4	-	-	-	-
79	62	A9023b		413.8	582.1	298	1.0	-	-	-	-	43.6	43.7	0.1	0.3
80	63	A9024a		413.7	582.1	303	0.01	717.9	742.9	25.0	3.5	-	-	-	-
81	63	A9024b		413.7	582.1	298	1.0	-	-	-	-	43.6	43.4	-0.2	-0.4
82	64	A9025a		407.0	582.1	298	0.01	719.0	741.6	22.5	3.1	42.9	42.4	-0.5	-1.1
83	65	A9026a		409.2	582.1	300	0.01	717.9	742.4	24.5	3.4	43.3	42.8	-0.5	-1.2
84	66	A9027a		406.1	582.1	298	0.01	711.1	738.0	27.0	3.8	42.9	43.1	0.2	0.4
85	67	A9028a		409.2	582.1	300	0.01	711.8	737.8	26.0	3.7	43.3	42.9	-0.4	-1.0
86	68	A9029a		408.4	582.1	298	0.01	705.9	733.0	27.1	3.8	43.3	43.0	-0.3	-0.8
87	69	A9030a		414.4	582.1	298	1.0	724.1	748.0	23.9	3.3	44.1	44.2	0.1	0.3
88	70	A9031a		416.4	582.1	298	1.0	722.5	749.1	26.6	3.7	44.5	44.1	-0.4	-0.8
89	71	A9032a		415.6	582.1	298	1.0	716.0	745.0	29.0	4.1	44.5	44.5	-0.0	-0.1
90	72	A9033a		417.4	582.1	298	1.0	717.0	745.7	28.7	4.0	44.9	44.4	-0.5	-1.0
91	73	A9034a		416.4	582.8	298	1.0	709.5	740.9	31.4	4.4	-	-	-	-
92	73	A9034b		416.4	582.8	305	0.01	-	-	-	-	43.8	44.1	0.2	0.5
93	74	A9035a		424.0	594.6	298	1.01	713.7	747.7	34.0	4.8	46.4	45.7	-0.7	-1.4
94	75	A0001a		439.2	606.6	298	1.0	776.7	803.2	26.5	3.4	45.2	46.3	1.1	2.3

Table S.14 – Comparison of experimental and simulated properties using WH combination rules (continued).

n_{sim}	n_{iso}	Code	Outlier	T_m [K]	T_b [K]	T [K]	P [bar]	ρ_{liq}[kg·m^{-3}] exp	sim	dev	err [%]	ΔH_{vap}[kJ·mol^{-1}] exp	sim	dev	err [%]
95	76	A0002a		432.4	606.6	298	1.0	763.6	783.0	19.4	2.5	43.5	44.7	1.2	2.7
96	77	A0003a		442.7	606.6	320	0.01	761.5	783.2	21.7	2.8	-	-	-	-
97	77	A0003b		442.7	606.6	298	1.0	-	-	-	-	46.0	46.0	-0.0	-0.1
98	78	A0004a		443.2	606.6	298	1.0	778.9	803.1	24.2	3.1	42.3	46.7	4.4	10.4
99	79	A0005a		433.5	623.0	298	1.0	760.9	788.5	27.6	3.6	45.2	46.2	1.0	2.2
100	80	A0006a		427.0	606.6	298	1.0	-	-	-	-	43.5	43.7	0.2	0.4
101	81	A0007a		410.6	581.4	298	1.0	714.8	745.0	30.2	4.2	43.5	43.1	-0.4	-0.9
102	82	A0008a		442.6	606.6	298	1.0	-	-	-	-	46.4	46.2	-0.2	-0.5
103	83	A0009a		437.8	606.6	298	1.0	-	-	-	-	46.4	47.4	1.0	2.1
104	84	A0010a		434.8	606.6	298	1.0	-	-	-	-	46.0	45.9	-0.1	-0.3
105	85	A0011a		426.3	606.6	298	1.0	-	-	-	-	45.2	44.6	-0.6	-1.4
106	86	A0012a		428.5	606.6	298	1.0	-	-	-	-	44.8	44.3	-0.5	-1.0
107	87	A0013a		432.0	606.6	298	1.0	-	-	-	-	45.6	45.2	-0.4	-0.8
108	88	A0014a		421.6	606.6	298	1.0	-	-	-	-	45.2	44.9	-0.3	-0.7
109	89	A0015a		421.0	606.6	298	1.0	-	-	-	-	44.4	44.3	-0.1	-0.2
110	90	A0016a		430.2	606.6	298	1.0	754.5	765.0	10.5	1.4	45.6	45.4	-0.2	-0.4
111	91	A0017a		429.4	606.6	298	1.0	-	-	-	-	46.0	46.2	0.2	0.4
112	92	A0018a		442.9	606.6	298	1.0	-	-	-	-	47.3	46.6	-0.7	-1.4
113	93	A0019a		435.3	606.6	298	1.0	-	-	-	-	46.4	47.0	0.6	1.3
114	94	A0020a		436.9	606.6	298	1.0	-	-	-	-	46.9	47.5	0.6	1.4
115	95	A0021a		436.1	606.6	298	1.0	-	-	-	-	46.4	46.3	-0.1	-0.1
116	96	A0022a		434.3	606.6	298	1.0	-	-	-	-	46.4	47.2	0.8	1.7
117	97	A0023a		435.1	606.6	298	1.0	-	-	-	-	46.9	47.2	0.3	0.6
118	98	A0024a		433.4	606.6	298	1.0	-	-	-	-	46.9	46.7	-0.2	-0.4
119	99	A0025a		429.3	606.6	298	1.0	-	-	-	-	46.0	45.7	-0.3	-0.8
120	100	A0026a		430.8	606.6	298	1.0	-	-	-	-	46.9	46.4	-0.5	-1.0
121	101	A0027a		434.3	606.6	298	1.0	-	-	-	-	46.4	45.7	-0.7	-1.4
122	102	A0028a		428.9	609.5	313	0.01	728.1	751.5	23.4	3.2	-	-	-	-
123	102	A0028b		428.9	609.5	298	1.0	-	-	-	-	46.0	45.5	-0.5	-1.1
124	103	A0029a		424.2	606.6	298	1.0	-	-	-	-	45.2	45.7	0.5	1.0
125	104	A0030a		426.0	606.6	298	1.0	736.2	758.3	22.1	3.0	46.0	45.6	-0.4	-1.0
126	105	A0031a		420.2	606.6	298	1.0	-	-	-	-	45.2	44.6	-0.6	-1.4
127	106	A0032a		421.5	606.6	298	1.0	-	-	-	-	45.6	45.2	-0.4	-0.9
128	107	A0033a		424.0	606.6	298	1.0	-	-	-	-	46.0	45.3	-0.7	-1.5
129	108	A0034a		422.1	606.6	298	1.0	720.0	745.0	25.0	3.5	46.4	45.3	-1.1	-2.4
130	109	A0035a		433.3	606.6	298	1.0	-	-	-	-	46.9	46.5	-0.4	-0.8
131	110	A0036a		439.9	606.6	298	1.0	-	-	-	-	46.4	46.0	-0.4	-0.9
132	111	A0037a		434.1	606.6	298	1.0	-	-	-	-	46.9	47.1	0.2	0.3
133	112	A0038a		435.7	606.6	298	1.0	751.9	773.8	21.9	2.9	47.3	47.4	0.1	0.2
134	113	A0039a		433.1	606.6	298	1.0	-	-	-	-	47.3	47.4	0.1	0.2
135	114	A0040a		427.3	606.6	298	1.0	-	-	-	-	46.4	45.6	-0.8	-1.7
136	115	A0041a		429.7	606.6	298	1.0	-	-	-	-	46.9	46.7	-0.2	-0.4
137	116	A0042a		433.9	606.6	298	1.0	-	-	-	-	47.3	46.4	-0.9	-1.8
138	117	A0043a		429.2	606.6	298	1.0	-	-	-	-	47.3	46.4	-0.9	-1.9
139	118	A0044a		420.8	606.6	298	1.0	-	-	-	-	46.4	45.9	-0.5	-1.1
140	119	A0045a		439.5	606.6	298	1.0	-	-	-	-	47.3	47.3	-0.0	-0.0
141	120	A0046a		434.0	606.6	298	1.0	-	-	-	-	47.2	47.5	0.3	0.7
142	121	A0047a		437.0	606.6	298	1.0	-	-	-	-	47.7	47.3	-0.4	-0.8

Table S.14 – Comparison of experimental and simulated properties using WH combination rules (continued).

n_{sim}	n_{iso}	Code	Outlier	T_m [K]	T_b [K]	T [K]	P [bar]	ρ_{liq}[kg·m^{-3}] exp	sim	dev	err [%]	ΔH_{vap}[kJ·mol^{-1}] exp	sim	dev	err [%]
143	122	A0048a		430.7	606.6	298	1.0	-	-	-	-	48.1	47.3	-0.8	-1.7
144	123	A0049a		434.4	606.6	298	1.0	-	-	-	-	48.5	47.1	-1.4	-2.8
145	124	A0050a		430.1	606.6	298	1.0	720.8	753.6	32.8	4.5	49.0	47.0	-2.0	-4.1
146	125	A0051a		437.1	606.6	298	1.0	747.2	768.9	21.7	2.9	47.7	47.1	-0.6	-1.2
147	126	A0052a		435.4	606.6	298	1.0	-	-	-	-	47.7	47.8	0.1	0.1
148	127	A0053a		436.2	606.6	298	1.0	-	-	-	-	48.1	47.7	-0.4	-0.9
149	128	A0054a		432.1	606.6	298	1.0	735.4	762.7	27.3	3.7	47.3	47.6	0.3	0.6
150	129	A0055a		434.4	606.6	298	1.0	-	-	-	-	48.1	47.4	-0.7	-1.5
151	130	A0056a		435.3	606.6	298	1.0	-	-	-	-	48.5	48.5	-0.0	-0.0
152	131	A0057a		436.6	606.6	298	1.0	741.0	766.1	25.1	3.4	48.1	48.2	0.1	0.2
153	132	A0058a		437.5	606.6	293	1.0	737.7	765.1	27.4	3.7	48.1	48.5	0.4	0.8
154	133	A0059a		431.4	606.6	298	1.0	-	-	-	-	47.7	47.0	-0.7	-1.5
155	134	A0060a		429.4	606.6	298	1.0	-	-	-	-	47.3	47.0	-0.3	-0.6
156	135	A0061a		432.9	606.6	298	1.0	-	-	-	-	48.1	47.2	-0.9	-1.8
157	136	A0062a		432.6	606.6	298	1.0	-	-	-	-	48.5	47.5	-1.0	-2.0
158	137	A0063a		429.1	606.6	298	1.0	722.6	753.8	31.2	4.3	48.5	47.6	-0.9	-1.8
159	138	A0064a		434.0	606.6	298	1.0	732.4	758.6	26.2	3.6	47.3	47.6	0.3	0.6
160	139	A0065a		431.7	606.6	298	1.0	726.4	753.9	27.5	3.8	49.0	47.7	-1.3	-2.7
161	140	A0066a		433.5	606.6	293	1.0	731.3	757.3	26.0	3.6	49.3	48.0	-1.3	-2.7
162	141	A0067a		433.0	606.6	298	1.0	720.2	750.3	30.1	4.2	47.7	48.0	0.3	0.5
163	142	A0068a		430.7	606.6	298	1.0	732.1	761.4	29.2	4.0	48.5	48.8	0.3	0.6
164	143	A0069a		436.8	606.6	298	1.0	734.3	762.5	28.2	3.8	48.1	48.7	0.6	1.2
165	144	A0070a		439.7	606.6	298	1.0	735.9	763.4	27.5	3.7	49.0	48.8	-0.2	-0.5
166	145	A0071a		438.3	606.6	293	1.0	732.6	764.2	31.6	4.3	49.8	49.3	-0.5	-0.9
167	146	A0072a		438.9	606.6	293	1.0	732.3	763.0	30.7	4.2	49.5	49.4	-0.1	-0.2
168	147	A0073a		440.9	606.6	293	1.0	735.4	764.4	29.0	3.9	50.2	49.4	-0.8	-1.5
169	148	A0074a		440.1	606.6	293	1.0	728.1	760.1	32.0	4.4	51.0	49.4	-1.6	-3.1
170	149	A0075a		447.3	617.7	298	1.01	725.9	762.3	36.4	5.0	50.2	50.4	0.2	0.4
171	150	F1101a	×	194.8	317.4	197	1.0	876.6	759.8	-116.8	-13.3	17.1	16.6	-0.5	-2.8
172	150	F1101b		194.8	317.4	298	38.39	528.3	526.7	-1.6	-0.3	-	-	-	-
173	150	F1101c	vap	194.8	317.4	298	1.0	574.4	-	-	-	0.0	-	-	-
174	151	F1201a	×	221.5	351.3	221	1.0	1213.8	1046.1	-167.7	-13.8	20.9	21.0	0.1	0.5
175	151	F1201b		221.5	351.3	298	17.62	891.6	878.4	-13.3	-1.5	-	-	-	-
176	151	F1201c	×	221.5	351.3	298	1.0	961.0	872.9	-88.1	-9.2	-	-	-	-
177	152	F1301a		191.0	299.0	191	1.0	1442.9	1507.4	64.5	4.5	16.7	24.1	7.4	44.5
178	152	F1301b	×	191.0	299.0	298	47.1	636.7	966.0	329.3	51.7	-	-	-	-
179	152	F1301c	vap	191.0	299.0	298	1.0	666.7	-	-	-	0.0	-	-	-
180	153	F1401a	×	145.1	227.5	145	1.0	1605.2	1493.9	-111.3	-6.9	12.3	15.4	3.1	25.3
181	154	F2101a		235.4	375.3	236	1.0	817.6	738.7	-78.9	-9.6	20.7	19.3	-1.4	-6.9
182	154	F2101b		235.4	375.3	298	9.09	707.5	632.0	-75.4	-10.7	-	-	-	-
183	155	F2201a	×	247.3	386.4	250	1.0	1009.0	843.9	-165.1	-16.4	22.7	21.1	-1.6	-7.3
184	155	F2201b	×	247.3	386.4	298	6.25	907.0	752.4	-154.6	-17.0	-	-	-	-
185	156	F2301a	×	225.8	345.9	220	1.0	1182.9	911.2	-271.6	-23.0	19.2	16.0	-3.2	-16.5
186	156	F2301b	vap	225.8	345.9	298	12.92	0.0	-	-	-	13.1	-	-	-
187	157	F3101a		263.8	421.1	264	1.0	769.2	705.9	-63.3	-8.2	-	-	-	-
188	158	F3102a		269.9	421.1	271	1.0	781.8	738.9	-42.9	-5.5	-	-	-	-
189	159	F3201a		281.1	430.4	276	0.62	-	-	-	-	25.1	24.5	-0.6	-2.5
190	160	F3202a	×	314.4	430.4	298	1.0	1005.7	891.7	-114.0	-11.3	-	-	-	-

Table S.14 – Comparison of experimental and simulated properties using WH combination rules (continued).

n_{sim}	n_{iso}	Code	Outlier	T_m	T_b	T	P	$\rho_{\text{liq}}[\text{kg}\cdot\text{m}^{-3}]$				$\Delta H_{\text{vap}}[\text{kJ}\cdot\text{mol}^{-1}]$			
				[K]	[K]	[K]	[bar]	exp	sim	dev	err [%]	exp	sim	dev	err [%]
191	161	F4101a	×	285.2	460.3	285	1.0	752.7	667.7	-85.0	-11.3	-	-	-	-
192	161	F4101b	×	285.2	460.3	298	1.39	735.3	649.9	-85.4	-11.6	-	-	-	-
193	162	F4102a		298.2	460.3	298	1.0	756.6	718.9	-37.6	-5.0	-	-	-	-
194	163	F4103a		305.6	460.3	298	1.0	770.8	740.6	-30.2	-3.9	-	-	-	-
195	164	F4201a		350.9	463.6	298	1.0	976.7	897.8	-78.9	-8.1	-	-	-	-
196	165	F4301a	×	289.9	406.6	298	1.38	1010.0	831.3	-178.7	-17.7	-	-	-	-
197	166	F5101a		317.9	494.6	298	1.0	773.7	721.7	-52.0	-6.7	-	-	-	-
198	167	F5102a		329.1	494.6	298	0.34	791.5	758.1	-33.4	-4.2	-	-	-	-
199	168	F5103a		335.9	494.6	298	1.0	784.9	767.1	-17.8	-2.3	30.9	30.6	-0.3	-1.1
200	169	F6101a		359.4	525.4	293	1.0	791.4	775.6	-15.8	-2.0	-	-	-	-
201	170	F6102a		364.6	525.4	298	1.0	795.8	785.8	-10.0	-1.3	35.6	35.1	-0.5	-1.4
202	171	F6201a		-	-	293	1.0	888.2	809.7	-78.5	-8.8	-	-	-	-
203	172	F6202a		-	-	298	1.0	940.7	897.2	-43.5	-4.6	-	-	-	-
204	173	F7101a		391.1	553.5	298	1.0	800.9	799.8	-1.1	-0.1	40.8	39.7	-1.1	-2.8
205	174	F8101a		415.4	579.2	298	1.0	806.7	810.9	4.2	0.5	-	-	-	-
206	175	F9101a		438.1	603.2	348	1.0	-	-	-	-	46.8	45.4	-1.4	-2.9
207	176	F0101a		459.4	625.5	293	1.0	819.4	831.4	12.0	1.5	-	-	-	-
208	177	C1101a		248.9	416.2	249	1.0	1008.3	959.8	-48.5	-4.8	-	-	-	-
209	178	C1201a		312.9	510.0	298	1.0	1316.4	1334.1	17.7	1.3	28.8	28.7	-0.1	-0.4
210	179	C1301a		334.3	536.4	298	1.0	1479.5	1543.7	64.2	4.3	31.1	36.6	5.5	17.6
211	180	C1401a		349.8	556.4	298	1.0	1584.3	1643.4	59.1	3.7	32.4	39.1	6.6	20.5
212	181	C2101a		285.4	460.4	285	1.0	906.2	883.8	-22.4	-2.5	24.9	22.1	-2.8	-11.4
213	181	C2101b		285.4	460.4	298	1.39	890.0	864.8	-25.1	-2.8	-	-	-	-
214	182	C2201a		330.4	523.0	298	1.0	1168.1	1159.5	-8.5	-0.7	30.6	30.6	-0.0	-0.0
215	183	C2202a		356.6	561.6	298	1.0	1245.6	1232.3	-13.3	-1.1	34.4	32.6	-1.8	-5.1
216	184	C2301a		347.2	545.0	298	1.0	1329.3	1327.3	-2.0	-0.2	32.4	33.7	1.3	4.1
217	185	C2302a		387.0	551.3	298	1.0	1432.8	1446.8	14.0	1.0	40.1	41.8	1.7	4.2
218	186	C3101a		308.9	496.5	298	1.0	855.6	837.6	-18.1	-2.1	-	-	-	-
219	187	C3201a		369.5	573.3	298	1.0	1153.0	1138.1	-14.9	-1.3	36.2	35.3	-0.9	-2.4
220	188	C3202a		393.6	573.3	298	1.0	1180.0	1174.1	-5.9	-0.5	40.6	36.3	-4.3	-10.6
221	189	C4101a		323.8	530.2	298	1.0	836.3	815.4	-20.9	-2.5	28.6	26.2	-2.4	-8.3
222	190	C4102a		342.0	530.2	298	1.0	871.4	866.6	-4.7	-0.5	31.7	29.3	-2.4	-7.6
223	191	C4103a		341.2	520.6	298	1.01	867.5	863.0	-4.5	-0.5	31.5	30.1	-1.4	-4.4
224	192	C4104a		351.6	530.2	298	1.01	880.4	884.5	4.1	0.5	33.5	30.9	-2.6	-7.8
225	193	C4201a		391.1	599.9	298	1.0	1106.3	1090.9	-15.4	-1.4	-	-	-	-
226	194	C4202a		397.1	599.9	298	1.0	1111.8	1112.0	0.2	0.0	40.1	39.5	-0.6	-1.4
227	195	C4203a		427.1	599.9	298	1.0	-	-	-	-	46.4	41.0	-5.4	-11.6
228	196	C5101a		358.8	560.5	298	1.0	859.6	853.8	-5.8	-0.7	-	-	-	-
229	197	C5102a		373.7	560.5	298	1.0	875.0	882.5	7.5	0.9	-	-	-	-
230	198	C5103a		371.7	560.5	298	1.0	870.0	874.8	4.8	0.6	36.2	34.3	-1.9	-5.2
231	199	C5104a		369.7	560.5	298	1.0	866.0	868.4	2.4	0.3	36.0	34.7	-1.3	-3.5
232	200	C5105a		381.5	560.5	298	1.0	877.8	888.5	10.7	1.2	38.2	35.5	-2.7	-7.0
233	201	C5201a		453.1	624.5	298	1.0	1095.6	1099.8	4.2	0.4	51.3	45.3	-6.0	-11.7
234	202	C6101a		408.2	588.0	298	1.0	873.5	890.9	17.3	2.0	42.0	40.0	-2.0	-4.6
235	203	C6201a		-	-	298	1.0	1064.0	1066.2	2.2	0.2	-	-	-	-
236	204	C6202a		-	-	298	1.0	1044.1	1043.8	-0.3	-0.0	-	-	-	-
237	205	C6203a		477.1	647.4	298	1.01	1063.7	1076.4	12.7	1.2	-	-	-	-
238	206	C7101a		433.6	613.5	298	1.0	871.5	892.4	20.9	2.4	47.0	44.7	-2.3	-5.0

Table S.14 – Comparison of experimental and simulated properties using WH combination rules (continued).

n_{sim}	n_{iso}	Code	Outlier	T_m [K]	T_b [K]	T [K]	P [bar]	ρ_{liq}[kg·m^{-3}] exp	sim	dev	err [%]	ΔH_{vap}[kJ·mol^{-1}] exp	sim	dev	err [%]
239	207	C8101a		456.6	637.1	298	1.0	869.4	894.0	24.7	2.8	51.4	49.2	-2.1	-4.2
240	208	C9101a		478.4	659.3	298	1.0	867.4	894.8	27.4	3.2	-	-	-	-
241	209	C0101a		499.0	680.1	298	1.0	865.8	895.9	30.1	3.5	64.0	58.3	-5.7	-8.9
242	210	B1101a		276.7	467.0	273	1.0	1729.8	1671.7	-58.1	-3.4	-	-	-	-
243	210	B1101b		276.7	467.0	298	2.25	1662.1	1607.0	-55.1	-3.3	-	-	-	-
244	210	B1101c		276.7	467.0	281	1.0	-	-	-	-	24.6	22.3	-2.3	-9.5
245	211	B1201a		370.1	615.9	298	0.06	2482.0	2469.4	-12.6	-0.5	37.0	36.9	-0.1	-0.3
246	212	B1301a		422.4	656.4	298	1.0	2877.2	2868.9	-8.3	-0.3	46.1	48.1	2.0	4.3
247	213	B1401a		462.6	706.8	374	1.0	2953.3	2974.9	21.6	0.7	-	-	-	-
248	213	B1401b		462.6	706.8	384	1.0	-	-	-	-	48.2	53.0	4.8	10.0
249	214	B2101a		311.5	503.8	298	1.0	1451.2	1444.1	-7.1	-0.5	-	-	-	-
250	214	B2101b		311.5	503.8	305	1.0	-	-	-	-	27.6	25.8	-1.8	-6.6
251	215	B2201a		381.1	635.8	298	1.0	2091.8	2068.0	-23.8	-1.1	-	-	-	-
252	216	B2202a		404.5	650.1	298	1.0	2169.5	2182.1	12.5	0.6	41.7	40.5	-1.2	-2.8
253	217	B2301a		462.1	671.9	298	1.0	2610.1	2605.3	-4.8	-0.2	-	-	-	-
254	218	B3101a		332.6	532.5	298	1.0	1301.4	1292.8	-8.6	-0.7	30.2	28.5	-1.7	-5.8
255	219	B3102a		344.1	536.9	298	1.0	1345.5	1357.8	12.3	0.9	31.9	30.6	-1.3	-4.2
256	220	B3201a		413.2	654.7	298	1.0	1925.0	1931.0	6.0	0.3	41.7	42.3	0.6	1.4
257	221	B3202a		440.4	654.7	298	1.0	1971.2	1980.0	8.8	0.4	-	-	-	-
258	222	B3301a		464.1	686.8	293	1.0	2298.5	2352.7	54.2	2.4	-	-	-	-
259	223	B3302a		474.1	686.8	293	1.0	2354.8	2358.6	3.8	0.2	-	-	-	-
260	224	B3303a		495.3	686.8	298	1.0	2411.0	2407.6	-3.4	-0.1	-	-	-	-
261	225	B4101a		346.4	557.6	298	1.0	1212.5	1203.8	-8.7	-0.7	31.8	30.2	-1.6	-4.9
262	226	B4102a		364.7	557.6	298	1.0	1257.1	1269.5	12.5	1.0	34.9	33.6	-1.3	-3.7
263	227	B4103a		364.4	557.6	298	1.0	1253.6	1258.1	4.5	0.4	34.8	33.1	-1.7	-4.8
264	228	B4104a		374.8	557.6	298	1.0	1268.6	1290.4	21.8	1.7	36.6	35.2	-1.4	-3.8
265	229	B4201a		448.1	672.7	298	1.0	1799.5	1823.9	24.4	1.4	-	-	-	-
266	230	B4202a		439.5	672.7	298	1.0	1787.0	1808.2	21.2	1.2	45.6	46.7	1.1	2.5
267	231	B4203a		448.1	672.7	293	1.0	1796.0	1801.1	5.1	0.3	-	-	-	-
268	232	B4301a		503.1	701.1	294	1.0	2175.3	2196.9	21.6	1.0	-	-	-	-
269	233	B4302a		493.1	701.1	298	1.0	2180.3	2233.1	52.8	2.4	-	-	-	-
270	234	B5101a		379.1	581.0	298	0.03	1193.5	1217.2	23.7	2.0	-	-	-	-
271	235	B5102a		381.1	581.0	298	0.03	1209.5	1204.2	-5.3	-0.4	-	-	-	-
272	236	B5103a		393.6	581.0	298	1.0	1214.4	1236.4	22.0	1.8	-	-	-	-
273	237	B5104a		391.8	581.0	298	1.0	1205.1	1225.2	20.1	1.7	-	-	-	-
274	238	B5105a		393.6	581.0	298	1.0	1200.7	1223.2	22.5	1.9	-	-	-	-
275	239	B5106a		390.6	581.0	298	1.0	1200.5	1212.2	11.7	1.0	38.5	37.9	-0.6	-1.7
276	240	B5107a		402.7	581.0	298	1.0	1211.4	1241.2	29.8	2.5	40.9	39.8	-1.1	-2.7
277	241	B5201a		453.1	689.9	293	1.0	1669.5	1700.8	31.3	1.9	-	-	-	-
278	242	B6101a		403.1	602.9	293	1.0	1179.2	1200.8	21.6	1.8	-	-	-	-
279	243	B6102a		414.4	602.9	298	1.0	1157.2	1188.1	30.9	2.7	-	-	-	-
280	244	B6103a		428.4	602.9	298	1.0	1168.8	1202.6	33.8	2.9	45.6	44.3	-1.3	-2.8
281	245	B7101a		452.1	623.5	298	1.0	1134.8	1172.4	37.6	3.3	50.4	49.0	-1.4	-2.8
282	246	B8101a		473.9	643.0	298	1.0	1107.7	1147.6	39.9	3.6	55.1	53.5	-1.6	-2.9
283	247	B9101a		494.6	661.6	298	1.0	1084.9	1127.1	42.2	3.9	-	-	-	-
284	248	B0101a		513.8	679.3	298	1.0	1062.5	1109.5	47.0	4.4	-	-	-	-
285	248	B0101b		513.8	679.3	398	1.0	-	-	-	-	56.6	55.7	-0.9	-1.6
286	249	I1101a		315.6	522.4	298	1.0	2264.5	2186.7	-77.8	-3.4	27.5	27.0	-0.5	-1.8

Table S.14 – Comparison of experimental and simulated properties using WH combination rules (continued).

n_{sim}	n_{iso}	Code	Outlier	T_m [K]	T_b [K]	T [K]	P [bar]	ρ_{liq}[kg·m^{-3}] exp	sim	dev	err [%]	ΔH_{vap}[kJ·mol^{-1}] exp	sim	dev	err [%]
287	250	I1201a	×	455.1	697.5	298	1.0	3307.8	3165.9	-141.9	-4.3	49.0	46.8	-2.2	-4.5
288	251	I2101a		345.4	562.2	298	1.0	1924.0	1926.0	2.0	0.1	31.7	31.3	-0.4	-1.3
289	252	I3101a		362.6	583.8	298	1.0	1694.5	1699.0	4.5	0.3	34.1	33.8	-0.3	-0.9
290	253	I3102a		375.6	602.3	298	1.0	1737.2	1770.8	33.5	1.9	36.0	35.8	-0.2	-0.5
291	254	I3201a		496.1	775.2	298	1.0	2565.1	2570.7	5.6	0.2	-	-	-	-
292	254	I3201b		496.1	775.2	369	0.01	-	-	-	-	53.5	51.9	-1.6	-3.1
293	255	I4101a		373.2	596.8	298	0.06	1536.0	1561.2	25.2	1.6	35.7	35.6	-0.1	-0.3
294	256	I4102a		393.6	625.0	298	0.02	1595.1	1628.9	33.8	2.1	38.8	38.9	0.1	0.1
295	257	I4103a		393.1	623.3	298	1.0	1589.0	1616.7	27.7	1.7	38.5	38.4	-0.1	-0.2
296	258	I4104a		403.7	638.8	298	1.0	1606.7	1646.0	39.2	2.4	40.3	40.4	0.1	0.2
297	259	I4201a		477.2	806.4	298	1.0	2349.6	2370.1	20.5	0.9	59.0	59.3	0.3	0.5
298	260	I5101a		407.1	670.9	293	1.0	1494.0	1544.6	50.6	3.4	-	-	-	-
299	261	I5102a		402.1	670.9	298	0.01	1486.6	1527.7	41.1	2.8	-	-	-	-
300	262	I5103a		413.1	670.9	293	1.0	1524.0	1540.2	16.2	1.1	-	-	-	-
301	263	I5104a		418.1	670.9	298	1.0	1505.5	1546.2	40.7	2.7	-	-	-	-
302	264	I5105a		421.4	659.1	298	1.0	1495.2	1535.8	40.6	2.7	42.2	43.8	1.6	3.7
303	265	I5106a		416.1	670.9	293	1.0	1500.9	1535.0	34.1	2.3	-	-	-	-
304	266	I5107a		430.1	671.4	298	1.0	1507.3	1555.6	48.2	3.2	44.4	44.9	0.5	1.2
305	267	I5201a		500.1	843.6	298	1.0	2173.4	2211.1	37.7	1.7	-	-	-	-
306	268	I6101a		441.1	697.2	293	1.0	1443.0	1487.2	44.2	3.1	-	-	-	-
307	269	I6102a		442.1	697.2	293	1.0	1419.3	1469.2	49.9	3.5	-	-	-	-
308	270	I6103a		454.5	704.4	298	1.0	1431.8	1483.8	52.0	3.6	-	-	-	-
309	270	I6103b		454.5	704.4	346	1.0	-	-	-	-	46.2	46.9	0.7	1.5
310	271	I6201a		522.5	831.8	298	1.0	2034.2	2085.9	51.7	2.5	-	-	-	-
311	272	I7101a		477.1	736.7	298	1.0	1371.9	1426.3	54.4	4.0	-	-	-	-
312	272	I7101b		477.1	736.7	357	0.01	-	-	-	-	48.4	50.7	2.2	4.6
313	273	I8101a		498.3	764.8	298	1.0	1326.7	1379.5	52.9	4.0	-	-	-	-
314	273	I8101b		498.3	764.8	374	0.01	-	-	-	-	50.9	53.9	3.0	5.8
315	274	I9101a		518.1	-	298	1.0	1283.6	1340.2	56.6	4.4	-	-	-	-
316	275	I0101a		-	-	298	1.0	1241.2	1294.3	53.1	4.3	-	-	-	-
317	276	I0102a		536.9	-	293	1.0	1256.7	1312.4	55.7	4.4	69.8	67.8	-2.0	-2.9
318	277	O2101a		248.3	400.1	248	1.0	735.0	682.6	-52.4	-7.1	21.6	21.6	0.0	0.2
319	277	O2101b		248.3	400.1	298	9.06	661.9	617.1	-44.8	-6.8	17.8	19.6	1.9	10.5
320	278	O3101a		280.5	437.8	273	1.0	726.0	701.2	-24.8	-3.4	-	-	-	-
321	278	O3101b		280.5	437.8	298	1.82	691.9	672.5	-19.4	-2.8	-	-	-	-
322	278	O3101c		280.5	437.8	280	1.0	-	-	-	-	31.2	24.9	-6.3	-20.3
323	279	O3201a		315.0	480.6	298	0.51	854.1	775.4	-78.7	-9.2	28.9	27.6	-1.3	-4.6
324	280	O4101a		303.9	464.5	298	1.0	709.0	690.6	-18.3	-2.6	26.4	27.0	0.6	2.4
325	281	O4102a		311.7	476.2	298	1.0	719.2	708.9	-10.3	-1.4	27.9	28.6	0.7	2.5
326	282	O4103a		307.6	466.7	298	1.0	707.8	694.9	-13.0	-1.8	27.2	26.8	-0.4	-1.5
327	283	O4201a		337.6	-	293	1.0	851.6	779.9	-71.7	-8.4	36.4	30.6	-5.8	-16.1
328	284	O4202a		357.2	536.1	298	1.01	861.4	821.8	-39.6	-4.6	36.8	37.6	0.8	2.0
329	285	O5101a		328.4	497.1	298	1.0	735.2	712.3	-22.9	-3.1	30.4	29.1	-1.3	-4.4
330	286	O5102a		331.7	500.0	298	1.0	727.2	720.7	-6.5	-0.9	-	-	-	-
331	287	O5103a		332.1	500.0	298	1.0	736.7	727.3	-9.4	-1.3	-	-	-	-
332	288	O5104a		326.1	500.0	298	1.0	717.3	717.6	0.3	0.0	30.0	31.7	1.7	5.7
333	289	O5105a		343.4	512.7	298	1.0	739.4	738.1	-1.3	-0.2	32.5	33.2	0.7	2.3
334	290	O5106a		337.0	500.2	298	1.0	727.0	724.7	-2.3	-0.3	31.4	31.3	-0.1	-0.2

Table S.14 – Comparison of experimental and simulated properties using WH combination rules (continued).

n_{sim}	n_{iso}	Code	Outlier	T_m [K]	T_b [K]	T [K]	P [bar]	ρ_{liq}[kg·m^{-3}] exp	sim	dev	err [%]	ΔH_{vap}[kJ·mol^{-1}] exp	sim	dev	err [%]
335	291	O5201a		356.1	-	298	1.01	845.1	788.2	-56.9	-6.7	37.6	32.5	-5.1	-13.5
336	292	O5202a		375.2	-	298	1.0	846.0	815.5	-30.5	-3.6	39.8	39.9	0.1	0.3
337	293	O5203a		361.1	524.0	298	1.0	825.2	794.8	-30.3	-3.7	35.7	35.2	-0.5	-1.3
338	294	O6101a		359.4	526.0	298	1.0	765.9	751.9	-14.0	-1.8	35.0	33.9	-1.1	-3.2
339	295	O6102a		345.9	526.0	298	1.0	735.2	731.5	-3.7	-0.5	33.1	33.5	0.4	1.3
340	296	O6103a		356.1	526.0	298	1.0	754.2	746.5	-7.7	-1.0	-	-	-	-
341	297	O6104a		341.4	500.1	298	1.0	718.7	726.0	7.3	1.0	32.7	34.2	1.5	4.6
342	298	O6105a		363.1	526.0	298	0.07	746.1	750.7	4.6	0.6	-	-	-	-
343	299	O6106a		363.1	526.0	298	1.0	749.0	750.6	1.5	0.2	-	-	-	-
344	300	O6107a		354.2	526.0	298	1.0	734.0	734.9	0.9	0.1	-	-	-	-
345	301	O6108a		364.1	526.0	298	0.06	749.9	748.3	-1.6	-0.2	-	-	-	-
346	302	O6109a		354.4	526.0	298	1.0	738.3	744.9	6.6	0.9	-	-	-	-
347	303	O6110a		353.1	526.0	298	1.0	732.4	739.3	6.9	0.9	-	-	-	-
348	304	O6111a		372.1	546.5	298	1.0	755.2	758.4	3.2	0.4	-	-	-	-
349	305	O6112a		365.4	526.0	298	1.0	744.7	747.7	3.0	0.4	36.3	35.9	-0.4	-1.0
350	306	O6113a		362.8	530.6	298	1.0	741.9	745.6	3.7	0.5	35.7	35.8	0.1	0.3
351	307	O6201a		-	-	293	1.0	844.6	804.6	-40.0	-4.7	-	-	-	-
352	308	O6202a		376.8	539.7	298	1.01	822.0	797.1	-24.9	-3.0	39.6	39.6	-0.0	-0.1
353	309	O6203a		-	-	298	1.0	852.9	836.7	-16.2	-1.9	-	-	-	-
354	310	O6204a		392.6	637.8	298	1.0	836.2	813.6	-22.6	-2.7	43.2	42.2	-1.0	-2.2
355	311	O6301a		432.9	608.0	298	1.0	939.2	895.2	-44.0	-4.7	48.0	54.0	6.0	12.4
356	312	O7101a		378.7	549.7	298	1.0	736.4	739.3	2.9	0.4	34.5	36.0	1.5	4.4
357	313	O7102a		375.1	546.0	298	1.0	761.8	763.8	2.0	0.3	38.2	38.2	-0.0	-0.0
358	314	O7103a		373.1	549.7	298	1.0	746.7	750.3	3.6	0.5	37.2	37.7	0.5	1.3
359	315	O7104a		371.2	549.7	298	1.0	734.9	745.9	11.0	1.5	-	-	-	-
360	316	O7105a		-	549.7	298	1.0	739.6	749.5	9.9	1.3	-	-	-	-
361	317	O7106a		385.6	549.7	298	1.0	752.1	757.3	5.2	0.7	-	-	-	-
362	318	O7107a		378.1	549.7	298	1.0	744.0	751.6	7.6	1.0	-	-	-	-
363	319	O7108a		-	-	298	1.0	750.1	761.3	11.2	1.5	-	-	-	-
364	320	O7109a		380.1	-	298	1.0	746.0	757.7	11.7	1.6	-	-	-	-
365	321	O7110a		398.1	-	298	1.0	766.3	775.0	8.7	1.1	-	-	-	-
366	322	O7111a		391.1	-	298	1.0	757.2	765.3	8.1	1.1	-	-	-	-
367	323	O7112a		390.1	-	298	1.0	754.2	764.3	10.1	1.3	-	-	-	-
368	324	O7201a		404.6	-	405	1.0	-	-	-	-	34.8	38.8	4.0	11.6
369	325	O7202a		387.1	-	298	1.0	868.8	804.8	-64.0	-7.4	43.9	41.8	-2.1	-4.8
370	326	O7203a		-	-	298	1.0	813.7	798.7	-15.0	-1.8	-	-	-	-
371	327	O7204a		-	-	298	1.0	851.6	842.3	-9.3	-1.1	-	-	-	-
372	328	O7205a		420.1	659.3	298	1.0	-	-	-	-	47.8	48.2	0.4	0.8
373	329	O7206a		-	-	298	1.0	840.3	828.9	-11.4	-1.4	-	-	-	-
374	330	O7207a		-	-	298	1.0	831.2	820.6	-10.6	-1.3	45.9	45.1	-0.8	-1.8
375	331	O7208a		-	-	298	1.0	832.7	820.1	-12.6	-1.5	46.8	46.3	-0.5	-1.2
376	332	O7301a		416.1	-	298	1.0	893.8	845.1	-48.7	-5.5	47.8	46.0	-1.8	-3.8
377	333	O7302a		416.9	-	293	1.0	922.9	888.7	-34.2	-3.7	-	-	-	-
378	334	O8101a		380.4	550.0	298	1.0	757.8	749.0	-8.8	-1.2	37.6	37.5	-0.1	-0.2
379	335	O8102a		439.9	571.4	298	1.0	748.0	755.3	7.3	1.0	40.1	40.3	0.2	0.5
380	336	O8103a		-	-	298	1.0	757.1	760.4	3.3	0.4	-	-	-	-
381	337	O8104a		-	571.4	298	1.0	758.1	765.7	7.6	1.0	42.3	42.1	-0.2	-0.5
382	338	O8105a		395.9	571.4	298	1.0	745.2	755.1	9.9	1.3	41.2	41.5	0.2	0.6

Table S.14 – Comparison of experimental and simulated properties using WH combination rules (continued).

n_{sim}	n_{iso}	Code	Outlier	T_m [K]	T_b [K]	T [K]	P [bar]	ρ_{liq}[kg·m^{-3}] exp	sim	dev	err [%]	ΔH_{vap}[kJ·mol^{-1}] exp	sim	dev	err [%]
383	339	O8106a		-	-	298	1.0	750.8	764.6	13.7	1.8	-	-	-	-
384	340	O8107a		-	-	298	1.0	754.5	765.1	10.6	1.4	-	-	-	-
385	341	O8108a		394.2	571.4	298	1.0	758.8	769.3	10.5	1.4	-	-	-	-
386	342	O8109a		-	-	298	1.0	760.1	771.1	11.0	1.5	-	-	-	-
387	343	O8110a		424.1	571.4	298	1.0	755.2	768.1	12.9	1.7	-	-	-	-
388	344	O8111a		-	-	298	1.0	761.1	775.5	14.4	1.9	-	-	-	-
389	345	O8112a		-	571.4	298	1.0	759.8	772.2	12.4	1.6	-	-	-	-
390	346	O8113a		424.1	-	298	1.0	775.6	787.8	12.2	1.6	-	-	-	-
391	347	O8114a		415.1	-	298	1.0	768.2	779.5	11.3	1.5	-	-	-	-
392	348	O8115a		-	571.4	298	1.0	765.3	778.2	12.9	1.7	-	-	-	-
393	349	O8116a		413.4	584.1	298	1.0	764.1	778.9	14.9	1.9	44.7	45.0	0.3	0.7
394	350	O8201a		478.1	-	298	1.0	825.6	815.0	-10.6	-1.3	-	-	-	-
395	351	O8202a		-	-	298	1.0	851.8	847.6	-4.2	-0.5	-	-	-	-
396	352	O8203a		-	-	298	1.0	843.3	837.6	-5.7	-0.7	-	-	-	-
397	353	O8204a		-	-	298	1.0	840.7	835.9	-4.8	-0.6	-	-	-	-
398	354	O8205a		-	-	298	1.0	833.5	828.6	-4.9	-0.6	-	-	-	-
399	355	O8206a		434.0	-	298	1.0	833.1	828.3	-4.8	-0.6	50.9	50.7	-0.2	-0.3
400	356	O8207a		478.1	-	298	1.0	831.2	825.8	-5.4	-0.7	50.6	50.3	-0.3	-0.6
401	357	O8301a		462.1	624.0	298	1.0	903.3	875.4	-27.9	-3.1	56.4	57.5	1.1	2.0
402	358	O9101a		-	-	298	1.01	792.4	787.3	-5.1	-0.6	-	-	-	-
403	359	O9102a		-	-	298	1.0	761.6	770.1	8.5	1.1	-	-	-	-
404	360	O9103a		-	-	298	1.0	766.4	778.7	12.3	1.6	-	-	-	-
405	361	O9104a		-	-	298	1.0	759.4	775.3	15.9	2.1	-	-	-	-
406	362	O9105a		-	-	298	1.0	764.3	780.6	16.3	2.1	-	-	-	-
407	363	O9106a		-	-	298	1.0	767.6	783.1	15.5	2.0	-	-	-	-
408	364	O9107a		-	-	298	1.0	765.6	780.3	14.7	1.9	-	-	-	-
409	365	O9108a		-	-	298	1.0	802.1	791.1	-10.9	-1.4	-	-	-	-
410	366	O9109a		-	-	298	1.0	768.7	787.7	19.0	2.5	-	-	-	-
411	367	O9110a		-	-	298	1.0	767.8	784.3	16.5	2.1	-	-	-	-
412	368	O9111a		-	591.5	298	1.0	783.0	797.9	14.9	1.9	-	-	-	-
413	369	O9112a		-	-	298	1.0	775.3	791.0	15.7	2.0	-	-	-	-
414	370	O9113a		-	-	298	1.0	773.0	790.0	17.0	2.2	-	-	-	-
415	371	O9114a		-	-	298	1.0	772.7	790.1	17.4	2.3	-	-	-	-
416	372	O9201a		438.6	-	298	1.0	820.3	811.3	-8.9	-1.1	-	-	-	-
417	373	O9202a		-	-	298	1.0	844.0	843.1	-0.9	-0.1	-	-	-	-
418	374	O9203a		-	-	298	1.0	838.6	835.8	-2.8	-0.3	-	-	-	-
419	375	O9204a		-	-	298	1.0	840.6	841.6	1.0	0.1	-	-	-	-
420	376	O9205a		452.4	-	298	1.0	831.5	831.2	-0.3	-0.0	-	-	-	-
421	377	O9206a		-	-	298	1.0	-	-	-	-	54.7	54.7	0.0	0.0
422	378	O0101a		-	-	298	1.0	-	-	-	-	45.3	47.0	1.7	3.7
423	379	O0102a		-	-	298	1.0	-	-	-	-	53.2	51.2	-2.0	-3.8
424	380	O0103a		-	-	293	1.0	784.8	797.2	12.4	1.6	-	-	-	-
425	381	O0104a		445.6	610.4	298	1.0	771.5	787.6	16.1	2.1	51.4	51.4	0.0	0.1
426	382	O0105a		-	-	293	1.0	777.8	793.8	16.0	2.1	-	-	-	-
427	383	O0106a		-	-	298	1.0	775.1	793.7	18.6	2.4	-	-	-	-
428	384	O0107a		-	-	298	1.0	771.2	791.2	20.0	2.6	-	-	-	-
429	385	O0108a		-	-	298	1.0	782.1	797.3	15.2	1.9	-	-	-	-
430	386	O0109a		-	-	293	1.0	787.4	800.6	13.2	1.7	-	-	-	-

Table S.14 – Comparison of experimental and simulated properties using WH combination rules (continued).

n_{sim}	n_{iso}	Code	Outlier	T_m [K]	T_b [K]	T [K]	P [bar]	ρ_{liq}[kg·m^{-3}] exp	sim	dev	err [%]	ΔH_{vap}[kJ·mol^{-1}] exp	sim	dev	err [%]
431	387	O0110a		-	-	298	1.0	775.8	797.2	21.4	2.8	-	-	-	-
432	388	O0111a		-	-	298	1.0	773.6	794.0	20.4	2.6	-	-	-	-
433	389	O0112a		-	-	298	1.0	788.6	806.5	17.9	2.3	-	-	-	-
434	390	O0113a		-	-	298	1.0	782.3	800.6	18.3	2.3	-	-	-	-
435	391	O0114a		-	-	298	1.0	779.8	799.4	19.6	2.5	-	-	-	-
436	392	O0115a		-	-	298	1.0	778.8	799.5	20.7	2.7	53.2	54.1	0.9	1.6
437	393	O0116a		460.1	-	298	1.0	779.2	799.7	20.5	2.6	-	-	-	-
438	394	O0201a		444.4	-	298	1.0	816.8	811.1	-5.7	-0.7	-	-	-	-
439	395	O0202a		415.4	-	298	1.0	829.0	828.9	-0.1	-0.0	57.8	56.6	-1.2	-2.0
440	396	O0203a		-	-	298	1.0	840.9	846.7	5.8	0.7	-	-	-	-
441	397	O0204a		-	-	293	1.0	840.9	842.1	1.2	0.1	-	-	-	-
442	398	O0205a		476.4	715.5	298	1.0	833.7	838.6	4.9	0.6	58.8	59.2	0.4	0.7
443	399	O0301a		-	-	288	1.0	886.9	883.1	-3.8	-0.4	-	-	-	-
444	400	A1101a		254.1	415.2	251	1.36	805.2	832.6	27.4	3.4	-	-	-	-
445	400	A1101b		254.1	415.2	293	1.0	814.0	771.2	-42.8	-5.3	-	-	-	-
446	401	A2101a		293.6	466.0	298	1.0	772.0	739.6	-32.4	-4.2	26.9	26.5	-0.4	-1.3
447	402	A3101a		321.1	504.4	298	1.0	791.2	751.7	-39.5	-5.0	29.6	29.8	0.2	0.8
448	403	A4101a		337.2	540.4	298	1.0	796.6	747.6	-49.0	-6.2	32.3	31.8	-0.5	-1.4
449	404	A4102a		347.9	537.2	298	1.0	796.6	773.4	-23.2	-2.9	33.7	34.1	0.4	1.1
450	405	A5101a		347.1	570.0	298	0.14	783.1	751.4	-31.7	-4.0	-	-	-	-
451	406	A5102a		365.1	570.0	298	0.06	804.1	774.0	-30.1	-3.7	-	-	-	-
452	407	A5103a		365.8	570.0	298	1.0	794.2	774.4	-19.8	-2.5	-	-	-	-
453	408	A5104a		376.1	566.1	298	1.0	806.2	792.7	-13.5	-1.7	38.1	38.5	0.4	1.2
454	409	A6101a		377.1	596.5	298	0.03	801.0	785.4	-15.6	-1.9	-	-	-	-
455	410	A6102a		386.1	596.5	298	1.0	809.7	786.2	-23.5	-2.9	-	-	-	-
456	411	A6103a		389.9	596.5	298	0.02	814.1	790.8	-23.3	-2.9	-	-	-	-
457	412	A6104a		390.1	596.5	298	0.02	808.0	788.7	-19.4	-2.4	-	-	-	-
458	413	A6105a		395.1	596.5	299	0.01	806.6	795.9	-10.7	-1.3	-	-	-	-
459	414	A6106a		401.4	591.0	298	1.01	833.1	805.3	-27.8	-3.3	42.3	42.9	0.6	1.5
460	415	A6201a		435.2	-	292	1.0	1003.0	959.0	-44.0	-4.4	-	-	-	-
461	416	A7101a		-	-	298	1.0	829.5	800.4	-29.1	-3.5	-	-	-	-
462	417	A7102a		-	-	298	1.0	823.1	810.5	-12.6	-1.5	-	-	-	-
463	417	A7102b		-	-	334	1.0	-	-	-	-	42.4	42.1	-0.3	-0.6
464	418	A7103a		416.1	620.4	298	1.0	814.3	807.5	-6.8	-0.8	-	-	-	-
465	418	A7103b		416.1	620.4	329	1.0	-	-	-	-	42.8	43.2	0.4	0.8
466	419	A7104a		-	620.4	298	1.0	824.0	814.6	-9.4	-1.1	-	-	-	-
467	420	A7105a		-	620.4	293	1.0	820.6	810.7	-9.9	-1.2	-	-	-	-
468	421	A7106a		425.9	616.8	298	1.0	813.3	815.6	2.3	0.3	48.0	47.4	-0.6	-1.3
469	422	A8101a		-	-	293	1.0	847.6	809.1	-38.5	-4.5	-	-	-	-
470	423	A8102a		-	-	293	1.0	847.6	813.1	-34.5	-4.1	-	-	-	-
471	424	A8103a		433.8	642.4	298	1.0	815.2	811.8	-3.3	-0.4	-	-	-	-
472	425	A8104a		447.1	638.9	298	1.01	821.1	823.9	2.9	0.3	51.0	51.8	0.8	1.5
473	426	A9101a		-	-	298	1.0	827.0	819.6	-7.4	-0.9	-	-	-	-
474	427	A9102a		-	-	291	1.0	884.3	826.4	-57.9	-6.5	-	-	-	-
475	428	A9103a		-	-	293	1.0	848.3	822.0	-26.3	-3.1	-	-	-	-
476	429	A9104a		-	-	293	1.0	842.3	816.3	-26.0	-3.1	-	-	-	-
477	430	A9105a		468.1	662.7	298	1.01	831.0	830.6	-0.4	-0.0	55.3	56.2	0.9	1.6
478	431	A0101a		-	-	293	1.0	843.0	827.9	-15.1	-1.8	-	-	-	-

Table S.14 – Comparison of experimental and simulated properties using WH combination rules (continued).

n_{sim}	n_{iso}	Code	Outlier	T_m [K]	T_b [K]	T [K]	P [bar]	ρ_{liq}[kg·m^{-3}] exp	sim	dev	err [%]	ΔH_{vap}[kJ·mol^{-1}] exp	sim	dev	err [%]
479	432	A0102a		403.1	656.6	298	1.0	813.4	823.1	9.7	1.2	-	-	-	-
480	433	A0103a		488.1	674.2	298	1.0	824.9	836.4	11.5	1.4	59.5	60.7	1.2	2.0
481	434	K3101a		329.4	508.2	298	1.0	784.4	770.3	-14.1	-1.8	31.3	32.0	0.7	2.2
482	435	K4101a		352.8	535.5	298	1.0	799.9	784.1	-15.8	-2.0	34.5	35.0	0.5	1.5
483	436	K5101a		367.6	553.4	298	1.0	809.4	781.7	-27.6	-3.4	36.8	36.6	-0.2	-0.5
484	437	K5102a		375.1	560.9	298	1.0	809.6	793.6	-16.0	-2.0	38.5	38.2	-0.3	-0.9
485	438	K5103a		375.5	561.1	298	1.0	801.8	795.7	-6.0	-0.8	38.3	39.2	0.9	2.3
486	439	K6101a		379.2	-	298	1.0	804.3	795.3	-9.0	-1.1	38.3	38.4	0.1	0.3
487	440	K6102a		386.6	587.5	298	1.0	806.6	790.4	-16.2	-2.0	39.8	39.9	0.1	0.2
488	441	K6103a		390.6	587.5	298	1.0	808.3	800.8	-7.5	-0.9	39.8	40.9	1.1	2.8
489	442	K6104a		389.6	574.6	298	1.0	796.3	793.8	-2.5	-0.3	41.0	41.1	0.1	0.2
490	443	K6105a		396.6	582.8	298	1.0	811.1	803.3	-7.8	-1.0	40.6	42.4	1.8	4.4
491	444	K6106a		400.9	587.6	298	1.0	807.1	809.4	2.2	0.3	42.2	43.5	1.3	3.1
492	445	K6201a		433.1	629.0	293	1.0	959.0	944.4	-14.6	-1.5	-	-	-	-
493	446	K7101a		403.8	611.4	294	1.0	823.0	825.4	2.4	0.3	-	-	-	-
494	447	K7102a		398.1	611.4	298	1.0	808.4	802.9	-5.5	-0.7	42.3	41.9	-0.4	-0.9
495	448	K7103a		398.1	611.4	298	1.0	801.2	801.7	0.5	0.1	-	-	-	-
496	449	K7104a		397.6	611.4	298	1.0	799.7	786.8	-13.0	-1.6	41.5	41.5	0.0	0.1
497	450	K7105a		405.1	611.4	293	1.0	827.3	813.6	-13.7	-1.7	-	-	-	-
498	451	K7106a		409.1	611.4	298	1.0	824.0	806.7	-17.3	-2.1	-	-	-	-
499	452	K7107a		406.1	611.4	302	0.01	809.5	796.6	-12.8	-1.6	-	-	-	-
500	453	K7108a		411.1	611.4	295	1.0	815.3	812.0	-3.3	-0.4	-	-	-	-
501	454	K7109a		413.1	611.4	298	1.0	828.0	810.8	-17.2	-2.1	-	-	-	-
502	455	K7110a		409.1	611.4	293	1.0	812.0	805.2	-6.8	-0.8	-	-	-	-
503	456	K7111a		412.1	611.4	298	1.0	808.5	812.1	3.6	0.4	-	-	-	-
504	457	K7112a		417.9	611.4	293	1.0	811.6	815.0	3.4	0.4	-	-	-	-
505	458	K7113a		417.1	602.0	298	1.0	811.6	811.2	-0.4	-0.0	-	-	-	-
506	459	K7114a		420.6	606.6	298	1.0	814.6	814.9	0.3	0.0	-	-	-	-
507	460	K7115a		424.1	611.4	298	1.0	811.6	818.5	6.9	0.8	46.1	47.8	1.7	3.8
508	461	K7201a		451.6	645.0	292	1.0	953.1	930.8	-22.3	-2.3	-	-	-	-
509	462	K7202a		449.1	645.0	293	1.0	945.0	932.9	-12.1	-1.3	-	-	-	-
510	463	K8101a		-	-	293	1.0	839.5	842.8	3.3	0.4	-	-	-	-
511	464	K8102a		408.2	633.4	298	1.0	802.3	801.4	-0.9	-0.1	43.3	43.7	0.4	0.8
512	465	K8103a		-	-	293	1.0	826.0	828.0	2.0	0.2	-	-	-	-
513	466	K8104a		426.6	633.4	293	1.0	838.9	843.5	4.6	0.5	-	-	-	-
514	467	K8105a		421.1	633.4	293	1.0	829.8	829.8	-0.0	-0.0	-	-	-	-
515	468	K8106a		420.1	633.4	293	1.0	825.7	832.4	6.7	0.8	-	-	-	-
516	469	K8107a		419.1	633.4	298	1.0	810.5	811.3	0.8	0.1	-	-	-	-
517	470	K8108a		-	-	293	1.0	829.0	829.3	0.3	0.0	-	-	-	-
518	471	K8109a		-	-	293	1.0	812.0	812.5	0.5	0.1	-	-	-	-
519	472	K8110a		420.6	633.4	293	1.0	812.1	803.8	-8.3	-1.0	-	-	-	-
520	473	K8111a		431.1	633.4	295	1.0	829.5	826.7	-2.8	-0.3	-	-	-	-
521	474	K8112a		426.1	633.4	298	1.0	817.0	814.6	-2.4	-0.3	-	-	-	-
522	475	K8113a		-	633.4	298	1.0	820.0	814.9	-5.1	-0.6	-	-	-	-
523	476	K8114a		431.1	633.4	293	1.0	817.5	815.2	-2.3	-0.3	-	-	-	-
524	477	K8115a		435.1	633.4	297	1.0	829.0	817.5	-11.5	-1.4	-	-	-	-
525	478	K8116a		427.1	633.4	295	1.0	813.0	811.6	-1.4	-0.2	-	-	-	-
526	479	K8117a		437.1	633.4	293	1.0	830.4	819.9	-10.5	-1.3	-	-	-	-

Table S.14 – Comparison of experimental and simulated properties using WH combination rules (continued).

n_{sim}	n_{iso}	Code	Outlier	T_m [K]	T_b [K]	T [K]	P [bar]	ρ_{liq}[kg·m^{-3}] exp	sim	dev	err [%]	ΔH_{vap}[kJ·mol^{-1}] exp	sim	dev	err [%]
527	480	K8118a		437.0	633.4	298	1.0	811.0	824.9	13.9	1.7	-	-	-	-
528	481	K8119a		440.1	633.4	298	1.0	810.0	817.9	7.9	1.0	-	-	-	-
529	482	K8120a		436.1	633.4	298	1.0	814.7	820.7	6.0	0.7	-	-	-	-
530	483	K8121a		440.6	633.4	298	1.0	822.0	822.5	0.5	0.1	-	-	-	-
531	484	K8122a		445.8	632.7	298	1.0	815.2	826.3	11.0	1.3	51.8	52.2	0.4	0.7
532	485	K9101a		425.1	653.7	298	1.0	820.2	823.2	3.0	0.4	45.4	46.5	1.1	2.4
533	486	K9102a		-	-	296	1.0	812.0	811.8	-0.2	-0.0	-	-	-	-
534	487	K9103a		-	-	293	1.0	816.8	823.9	7.1	0.9	-	-	-	-
535	488	K9104a		-	-	298	1.0	809.0	815.9	6.9	0.8	-	-	-	-
536	489	K9105a		435.1	653.7	287	1.0	826.0	825.0	-1.0	-0.1	-	-	-	-
537	490	K9106a		445.0	653.7	298	1.0	813.5	812.3	-1.2	-0.2	-	-	-	-
538	491	K9107a		441.4	653.7	298	1.0	802.4	808.6	6.1	0.8	50.9	50.9	0.0	0.1
539	492	K9108a		-	-	293	1.0	833.5	836.4	2.9	0.3	-	-	-	-
540	493	K9109a		-	-	298	1.0	817.0	817.7	0.7	0.1	-	-	-	-
541	494	K9110a		447.1	653.7	287	1.0	829.0	831.2	2.2	0.3	-	-	-	-
542	495	K9111a		456.6	653.7	298	1.0	820.0	823.2	3.2	0.4	-	-	-	-
543	496	K9112a		456.0	653.7	293	1.0	821.2	822.6	1.4	0.2	-	-	-	-
544	497	K9113a		-	-	293	1.0	824.6	829.4	4.8	0.6	-	-	-	-
545	498	K9114a		456.6	653.7	300	1.0	832.0	825.0	-7.0	-0.8	-	-	-	-
546	499	K9115a		-	653.7	298	1.0	815.0	818.1	3.1	0.4	-	-	-	-
547	500	K9116a		451.1	653.7	293	1.0	823.9	825.2	1.3	0.2	-	-	-	-
548	501	K9117a		-	653.7	298	1.0	822.0	830.7	8.7	1.1	-	-	-	-
549	502	K9118a		461.6	640.0	298	1.0	817.8	828.5	10.7	1.3	53.3	55.4	2.1	3.9
550	503	K9119a		460.6	653.7	298	1.0	819.7	827.4	7.7	0.9	-	-	-	-
551	504	K9120a		463.1	653.7	298	1.0	820.4	829.8	9.3	1.1	55.6	55.5	-0.1	-0.2
552	505	K9121a		467.1	652.5	298	1.0	817.8	833.0	15.2	1.9	-	-	-	-
553	506	K0101a		-	-	298	1.0	816.9	816.9	-0.0	-0.0	48.8	50.0	1.2	2.4
554	507	K0102a		-	-	298	1.0	825.2	823.4	-1.8	-0.2	-	-	-	-
555	508	K0103a		-	-	289	1.0	825.2	831.2	6.0	0.7	-	-	-	-
556	509	K0104a		-	-	293	1.0	832.0	830.1	-1.9	-0.2	-	-	-	-
557	510	K0105a		-	-	293	1.0	814.3	827.3	13.0	1.6	-	-	-	-
558	511	K0106a		473.0	672.7	293	1.0	822.6	829.2	6.6	0.8	-	-	-	-
559	512	K0107a		-	-	298	1.0	818.0	824.5	6.5	0.8	-	-	-	-
560	513	K0108a		476.6	672.7	293	1.0	821.3	832.6	11.3	1.4	-	-	-	-
561	514	K0109a		-	-	293	1.0	838.4	839.1	0.7	0.1	-	-	-	-
562	515	K0110a		477.1	672.7	298	1.0	820.5	834.1	13.5	1.6	-	-	-	-
563	516	K0111a		479.6	672.7	294	1.0	822.0	836.8	14.8	1.8	-	-	-	-
564	517	K0112a		476.1	672.7	298	1.0	821.9	835.4	13.4	1.6	-	-	-	-
565	518	K0113a		483.4	672.7	298	1.0	820.1	838.4	18.3	2.2	60.9	60.9	0.0	0.0
566	519	E2201a	vap	304.9	487.2	298	1.0	966.8	-	-	-	28.4	-	-	-
567	520	E3201a		327.5	508.4	298	1.0	915.9	863.8	-52.1	-5.7	31.5	32.4	0.9	2.9
568	521	E3202a		330.1	506.6	298	1.01	927.5	866.3	-61.2	-6.6	32.3	32.0	-0.3	-0.8
569	522	E4201a		341.2	514.9	298	0.17	870.2	827.9	-42.3	-4.9	-	-	-	-
570	523	E4202a		354.0	538.0	298	1.0	899.9	861.7	-38.2	-4.2	36.6	36.3	-0.3	-0.7
571	524	E4203a		352.6	530.6	298	1.0	909.1	860.3	-48.8	-5.4	35.7	35.5	-0.2	-0.5
572	525	E4204a		350.2	523.3	298	1.0	894.3	854.1	-40.1	-4.5	35.1	35.3	0.2	0.6
573	526	E4401a		447.1	-	273	1.0	1193.0	1227.7	34.7	2.9	-	-	-	-
574	527	E5201a		355.9	541.0	298	0.09	871.9	856.2	-15.7	-1.8	-	-	-	-

Table S.14 – Comparison of experimental and simulated properties using WH combination rules (continued).

n_{sim}	n_{iso}	Code	Outlier	T_m [K]	T_b [K]	T [K]	P [bar]	ρ_{liq}[kg·m^{-3}] exp	sim	dev	err [%]	ΔH_{vap}[kJ·mol^{-1}] exp	sim	dev	err [%]
575	528	E5202a		371.2	551.4	298	1.0	875.7	846.5	-29.2	-3.3	-	-	-	-
576	529	E5203a		363.6	541.0	298	0.06	878.6	844.1	-34.5	-3.9	-	-	-	-
577	530	E5204a		379.2	541.0	298	1.0	887.6	869.0	-18.6	-2.1	40.5	41.0	0.5	1.2
578	531	E5205a		365.6	540.7	298	1.0	883.3	842.3	-41.0	-4.6	37.3	37.3	-0.0	-0.1
579	532	E5206a		361.6	532.0	298	1.0	869.0	833.2	-35.8	-4.1	37.0	37.2	0.2	0.5
580	533	E5207a		375.9	554.5	298	1.01	892.5	860.9	-31.6	-3.5	39.8	39.6	-0.2	-0.4
581	534	E5208a		372.2	546.0	298	1.0	884.0	851.2	-32.8	-3.7	39.3	38.8	-0.5	-1.2
582	535	E5209a		374.6	549.7	298	1.01	882.8	857.9	-24.9	-2.8	39.1	39.4	0.3	0.7
583	536	E5401a	×	454.6	-	298	1.0	1146.7	1064.1	-82.6	-7.2	57.5	56.1	-1.4	-2.3
584	537	E5402a		437.6	-	298	1.0	1135.5	1087.2	-48.3	-4.2	56.4	57.8	1.4	2.5
585	538	E6201a		385.6	564.6	298	0.02	883.7	871.3	-12.4	-1.4	-	-	-	-
586	539	E6202a		397.1	564.6	298	1.0	877.0	863.1	-13.9	-1.6	-	-	-	-
587	540	E6203a		406.6	576.0	298	1.0	880.4	871.2	-9.1	-1.0	45.2	45.7	0.5	1.0
588	541	E6204a		374.2	-	293	1.0	850.0	846.0	-4.0	-0.5	38.8	39.2	0.4	1.0
589	542	E6205a		369.1	564.6	298	1.0	861.6	825.3	-36.3	-4.2	38.0	37.3	-0.7	-1.9
590	543	E6206a		-	-	293	1.0	884.7	857.9	-26.8	-3.0	-	-	-	-
591	544	E6207a		383.0	553.1	298	1.0	864.0	837.8	-26.3	-3.0	39.8	40.6	0.8	2.0
592	545	E6208a		389.6	564.6	298	1.0	875.9	849.3	-26.5	-3.0	-	-	-	-
593	546	E6209a		383.1	553.0	298	1.0	860.1	834.6	-25.5	-3.0	-	-	-	-
594	547	E6210a		389.8	560.8	298	1.0	866.3	848.7	-17.6	-2.0	39.5	42.0	2.5	6.3
595	548	E6211a		385.1	564.6	298	1.0	866.0	845.3	-20.8	-2.4	-	-	-	-
596	549	E6212a		400.6	564.6	298	1.0	885.2	865.3	-19.8	-2.2	43.7	44.1	0.4	0.9
597	550	E6213a		394.6	571.0	298	1.0	873.8	854.3	-19.5	-2.2	42.0	43.1	1.1	2.7
598	551	E6214a		395.6	568.6	298	1.0	876.4	855.1	-21.2	-2.4	43.2	42.8	-0.4	-0.9
599	552	E6215a		399.1	575.4	298	1.0	876.4	862.0	-14.3	-1.6	42.7	44.0	1.3	3.1
600	553	E6401a		447.1	-	298	1.0	1093.6	1017.4	-76.2	-7.0	-	-	-	-
601	553	E6401b		447.1	-	293	1.0	-	-	-	-	57.8	56.0	-1.8	-3.1
602	554	E6402a		442.1	635.0	298	1.0	1070.0	1029.3	-40.7	-3.8	59.0	59.2	0.2	0.4
603	555	E6403a		469.6	657.0	298	1.0	1114.0	1041.9	-72.1	-6.5	60.9	61.5	0.6	1.0
604	556	E6404a		463.6	653.0	298	1.0	1098.7	1032.1	-66.6	-6.1	61.0	59.9	-1.1	-1.8
605	557	E7201a		428.6	586.3	298	1.0	874.6	874.7	0.1	0.0	50.0	50.1	0.1	0.3
606	558	E7202a		391.6	-	298	1.0	849.5	837.3	-12.2	-1.4	41.3	42.1	0.8	2.0
607	559	E7203a		-	-	293	1.0	870.0	854.1	-15.9	-1.8	43.9	43.4	-0.5	-1.0
608	560	E7204a		-	586.3	298	1.0	853.9	847.1	-6.8	-0.8	-	-	-	-
609	561	E7205a		-	-	298	1.0	872.5	846.1	-26.4	-3.0	40.3	41.7	1.4	3.5
610	562	E7206a		414.6	-	293	1.0	864.7	833.3	-31.4	-3.6	-	-	-	-
611	563	E7207a		396.1	-	294	1.0	846.7	828.8	-18.0	-2.1	-	-	-	-
612	564	E7208a		401.6	586.3	298	1.0	866.0	847.6	-18.4	-2.1	-	-	-	-
613	565	E7209a		409.1	586.3	293	1.0	879.7	860.9	-18.8	-2.1	-	-	-	-
614	566	E7210a		-	-	293	1.0	876.5	861.3	-15.2	-1.7	-	-	-	-
615	567	E7211a		-	-	293	1.0	867.8	853.0	-14.8	-1.7	44.7	45.2	0.5	1.2
616	568	E7212a		407.1	-	298	1.0	859.5	842.7	-16.8	-2.0	-	-	-	-
617	569	E7213a		408.1	-	298	1.0	861.2	845.8	-15.4	-1.8	-	-	-	-
618	570	E7214a		409.1	592.0	298	1.0	867.5	847.1	-20.4	-2.4	-	-	-	-
619	571	E7215a		406.1	-	298	1.0	861.2	845.2	-16.0	-1.9	-	-	-	-
620	572	E7216a		402.1	-	298	1.0	853.9	838.6	-15.3	-1.8	-	-	-	-
621	573	E7217a		413.1	586.3	293	1.0	867.8	864.4	-3.4	-0.4	-	-	-	-
622	574	E7218a		405.1	586.3	303	0.01	861.5	847.2	-14.3	-1.7	-	-	-	-

Table S.14 – Comparison of experimental and simulated properties using WH combination rules (continued).

n_{sim}	n_{iso}	Code	Outlier	T_m [K]	T_b [K]	T [K]	P [bar]	$\rho_{\text{liq}}[\text{kg}\cdot\text{m}^{-3}]$ exp	sim	dev	err [%]	$\Delta H_{\text{vap}}[\text{kJ}\cdot\text{mol}^{-1}]$ exp	sim	dev	err [%]
623	575	E7219a		414.8	586.1	299	1.0	864.8	856.5	-8.3	-1.0	46.4	47.2	0.8	1.7
624	576	E7220a		406.1	586.3	298	1.0	863.1	849.3	-13.8	-1.6	-	-	-	-
625	577	E7221a		422.6	586.3	298	1.0	880.4	868.4	-12.0	-1.4	47.7	48.6	0.9	1.8
626	578	E7222a		419.2	586.3	298	1.01	869.4	860.0	-9.4	-1.1	47.0	47.7	0.7	1.5
627	579	E7223a		416.4	593.7	298	1.0	868.2	857.2	-11.0	-1.3	-	-	-	-
628	580	E7224a		419.8	594.6	298	1.0	871.5	860.3	-11.2	-1.3	48.5	47.3	-1.2	-2.5
629	581	E7225a		422.1	599.9	298	1.0	872.2	865.8	-6.4	-0.7	48.6	48.6	0.0	0.0
630	582	E7401a		-	-	293	1.0	-	-	-	-	55.6	55.9	0.3	0.5
631	583	E7402a		-	-	298	1.0	1061.4	1000.5	-60.9	-5.7	-	-	-	-
632	584	E7403a		469.1	-	298	1.0	1076.0	1005.8	-70.2	-6.5	-	-	-	-
633	585	E7404a		463.6	-	293	1.0	1059.0	999.3	-59.7	-5.6	-	-	-	-
634	586	E7405a		472.1	653.0	298	1.0	1049.8	1001.0	-48.8	-4.6	58.7	60.7	2.0	3.4
635	587	E7406a		481.4	-	293	1.0	1076.0	1017.8	-58.2	-5.4	-	-	-	-
636	588	E7407a		487.1	-	293	1.01	1087.7	1026.3	-61.4	-5.6	65.7	66.1	0.4	0.6
637	589	E7408a		482.6	-	293	1.0	1054.7	1018.6	-36.1	-3.4	-	-	-	-
638	590	E8201a		451.2	606.5	302	1.0	869.0	870.6	1.6	0.2	53.8	54.0	0.2	0.3
639	591	E8202a		-	-	293	1.0	830.0	830.8	0.8	0.1	-	-	-	-
640	592	E8203a		-	-	298	1.0	874.9	865.5	-9.4	-1.1	-	-	-	-
641	593	E8204a		-	-	277	1.0	883.0	874.6	-8.4	-0.9	-	-	-	-
642	594	E8205a		-	-	293	1.0	860.4	850.8	-9.6	-1.1	-	-	-	-
643	595	E8206a		-	-	293	1.0	867.9	863.7	-4.2	-0.5	-	-	-	-
644	596	E8207a		437.4	606.5	308	0.01	874.7	837.7	-36.9	-4.2	-	-	-	-
645	597	E8208a		426.1	606.5	320	0.01	862.6	831.2	-31.4	-3.6	-	-	-	-
646	598	E8209a		-	-	298	1.0	866.0	851.7	-14.3	-1.6	-	-	-	-
647	599	E8210a		421.1	-	293	1.0	847.0	840.4	-6.6	-0.8	48.5	47.3	-1.2	-2.5
648	600	E8211a		-	606.5	289	1.0	870.0	843.3	-26.7	-3.1	-	-	-	-
649	601	E8212a		420.1	-	298	1.0	846.1	833.0	-13.1	-1.5	-	-	-	-
650	602	E8213a		420.6	-	298	1.0	880.5	846.0	-34.5	-3.9	-	-	-	-
651	603	E8214a		-	-	293	1.0	875.0	863.8	-11.2	-1.3	-	-	-	-
652	604	E8215a		-	-	293	1.0	863.3	856.7	-6.6	-0.8	-	-	-	-
653	605	E8216a		428.3	-	293	1.0	876.5	857.3	-19.2	-2.2	48.4	49.8	1.4	2.9
654	605	E8216b		428.3	-	298	1.0	-	-	-	-	48.4	49.5	1.1	2.3
655	606	E8217a		-	-	293	1.0	869.7	856.3	-13.4	-1.5	-	-	-	-
656	607	E8218a		-	-	298	1.0	857.4	848.7	-8.7	-1.0	-	-	-	-
657	608	E8219a		-	-	293	1.0	878.0	860.7	-17.3	-2.0	-	-	-	-
658	609	E8220a		429.1	-	298	1.0	857.5	849.3	-8.2	-1.0	-	-	-	-
659	610	E8221a		436.1	606.5	293	1.0	870.5	860.9	-9.6	-1.1	-	-	-	-
660	611	E8222a		430.1	611.0	298	1.0	860.6	850.0	-10.6	-1.2	-	-	-	-
661	612	E8223a		446.1	606.5	298	1.0	865.0	856.6	-8.4	-1.0	-	-	-	-
662	613	E8224a		-	-	298	1.0	863.2	848.2	-15.1	-1.7	-	-	-	-
663	614	E8225a		440.2	606.5	298	1.0	861.3	849.0	-12.3	-1.4	-	-	-	-
664	615	E8226a		436.4	606.5	293	1.0	857.9	850.7	-7.2	-0.8	-	-	-	-
665	616	E8227a		435.6	606.5	293	1.0	879.0	871.7	-7.3	-0.8	-	-	-	-
666	617	E8228a		436.1	606.5	298	1.0	869.1	862.8	-6.3	-0.7	-	-	-	-
667	618	E8229a		-	606.5	298	1.0	859.9	855.5	-4.4	-0.5	-	-	-	-
668	619	E8230a		447.1	606.5	298	1.01	875.9	871.0	-4.9	-0.6	51.6	53.0	1.4	2.8
669	620	E8231a		440.1	606.5	298	1.0	866.7	863.2	-3.5	-0.4	50.6	52.1	1.5	3.0
670	621	E8232a		440.6	606.5	298	1.0	865.8	862.8	-2.9	-0.3	-	-	-	-

Table S.14 – Comparison of experimental and simulated properties using WH combination rules (continued).

n_{sim}	n_{iso}	Code	Outlier	T_m [K]	T_b [K]	T [K]	P [bar]	ρ_{liq}[kg·m^{-3}] exp	sim	dev	err [%]	ΔH_{vap}[kJ·mol^{-1}] exp	sim	dev	err [%]
671	622	E8233a		438.1	606.5	298	1.01	869.1	861.5	-7.6	-0.9	-	-	-	-
672	623	E8234a		441.9	606.5	298	1.0	868.1	864.1	-4.0	-0.5	52.2	51.9	-0.3	-0.6
673	624	E8235a		444.6	606.5	298	1.0	868.6	868.6	0.0	0.0	51.9	53.0	1.1	2.0
674	625	E8401a		-	-	298	1.0	1036.9	989.4	-47.6	-4.6	-	-	-	-
675	626	E8402a		474.1	-	298	1.0	1017.4	971.5	-45.9	-4.5	-	-	-	-
676	627	E8403a		489.6	663.0	298	1.0	1035.3	991.1	-44.2	-4.3	64.5	66.1	1.6	2.4
677	628	E8404a		495.1	-	298	1.0	1057.6	1008.8	-48.8	-4.6	69.0	69.5	0.5	0.7
678	629	E8405a		484.1	-	293	1.0	1042.0	994.3	-47.7	-4.6	67.6	65.5	-2.1	-3.1
679	630	E8406a		502.1	-	293	1.0	1046.0	1006.4	-39.6	-3.8	-	-	-	-
680	631	E9201a		471.9	625.3	298	1.0	871.0	876.1	5.1	0.6	58.2	58.8	0.6	1.1
681	632	E9202a		-	-	298	1.0	-	-	-	-	48.4	49.9	1.5	3.1
682	633	E9203a		-	-	298	1.0	-	-	-	-	47.8	47.2	-0.6	-1.2
683	634	E9204a		-	-	298	1.0	-	-	-	-	50.4	50.6	0.2	0.4
684	635	E9205a		460.3	625.3	298	1.0	-	-	-	-	50.3	49.6	-0.7	-1.5
685	636	E9206a		-	-	301	1.0	855.0	848.7	-6.3	-0.7	-	-	-	-
686	637	E9207a		442.1	-	293	1.0	862.7	851.5	-11.2	-1.3	51.7	53.1	1.4	2.7
687	638	E9208a		-	-	298	1.0	869.0	862.0	-7.0	-0.8	-	-	-	-
688	639	E9209a		442.1	-	298	1.0	861.7	843.7	-18.0	-2.1	-	-	-	-
689	640	E9210a		-	-	293	1.0	848.2	845.9	-2.3	-0.3	-	-	-	-
690	641	E9211a		-	-	293	1.0	871.0	866.3	-4.7	-0.5	-	-	-	-
691	642	E9212a		-	-	293	1.0	857.2	859.9	2.7	0.3	-	-	-	-
692	643	E9213a		-	-	293	1.0	868.8	858.6	-10.2	-1.2	-	-	-	-
693	644	E9214a		-	-	293	1.0	866.9	859.4	-7.5	-0.9	-	-	-	-
694	645	E9215a		452.1	-	293	1.0	862.0	860.5	-1.5	-0.2	50.6	53.9	3.3	6.4
695	646	E9216a		-	625.3	293	1.0	872.1	857.8	-14.3	-1.6	-	-	-	-
696	647	E9217a		450.1	625.3	293	1.0	867.9	863.0	-4.9	-0.6	-	-	-	-
697	648	E9218a		456.9	-	298	1.0	856.7	854.0	-2.7	-0.3	-	-	-	-
698	649	E9219a		453.1	625.3	293	1.0	870.8	869.7	-1.1	-0.1	-	-	-	-
699	650	E9220a		452.1	625.3	298	1.0	860.3	858.2	-2.1	-0.2	-	-	-	-
700	651	E9221a		456.1	625.3	298	1.0	853.6	855.9	2.3	0.3	-	-	-	-
701	652	E9222a		455.1	625.3	298	1.0	864.7	851.9	-12.8	-1.5	-	-	-	-
702	653	E9223a		-	-	298	1.0	854.9	854.4	-0.5	-0.1	-	-	-	-
703	654	E9224a		-	-	298	1.0	852.5	850.6	-1.9	-0.2	-	-	-	-
704	655	E9225a		-	625.3	298	1.0	857.0	858.3	1.3	0.2	-	-	-	-
705	656	E9226a		466.1	625.3	298	1.0	873.1	873.5	0.4	0.0	56.4	57.4	1.0	1.8
706	657	E9227a		460.1	625.3	298	1.0	864.7	866.5	1.8	0.2	-	-	-	-
707	658	E9228a		460.1	625.3	298	1.0	863.0	865.0	2.0	0.2	-	-	-	-
708	659	E9229a		459.1	625.3	298	1.0	863.4	865.6	2.2	0.3	-	-	-	-
709	660	E9230a		458.1	625.3	298	1.0	861.9	865.0	3.1	0.4	53.6	56.3	2.7	5.0
710	661	E9231a		463.1	625.3	298	1.0	865.4	867.1	1.7	0.2	57.1	56.3	-0.8	-1.4
711	662	E9232a		465.6	625.3	298	1.0	866.4	871.4	4.9	0.6	56.9	57.4	0.5	0.8
712	663	E9401a		509.2	-	298	1.0	-	-	-	-	63.9	62.4	-1.5	-2.3
713	664	E9402a		481.1	-	298	1.0	1000.7	962.4	-38.3	-3.8	-	-	-	-
714	665	E9403a		502.1	-	298	1.0	-	-	-	-	66.2	67.7	1.5	2.2
715	666	E9404a		509.6	-	293	1.0	1022.0	986.6	-35.4	-3.5	67.0	71.3	4.3	6.4
716	667	E9405a		509.2	-	293	1.0	1039.1	1000.7	-38.4	-3.7	73.5	73.7	0.2	0.3
717	668	E9406a		514.1	-	293	1.0	1029.6	995.7	-33.9	-3.3	-	-	-	-
718	669	E0201a		485.7	643.0	293	1.0	867.0	880.1	13.1	1.5	-	-	-	-

Table S.14 – Comparison of experimental and simulated properties using WH combination rules (continued).

n_{sim}	n_{iso}	Code	Outlier	T_m [K]	T_b [K]	T [K]	P [bar]	ρ_{liq}[kg·m^{-3}] exp	sim	dev	err [%]	ΔH_{vap}[kJ·mol^{-1}] exp	sim	dev	err [%]
719	670	E0202a		-	-	293	1.0	843.1	839.1	-4.0	-0.5	48.9	50.5	1.6	3.4
720	671	E0203a		-	-	298	1.0	-	-	-	-	48.0	48.9	0.9	1.9
721	672	E0204a		461.1	-	273	1.0	872.9	863.6	-9.2	-1.1	-	-	-	-
722	673	E0205a		-	-	289	1.0	883.7	886.5	2.8	0.3	-	-	-	-
723	674	E0206a		-	-	293	1.0	856.8	858.7	1.9	0.2	-	-	-	-
724	675	E0207a		463.1	-	298	1.0	854.1	852.3	-1.8	-0.2	-	-	-	-
725	676	E0208a		460.1	-	293	1.0	847.4	858.1	10.7	1.3	-	-	-	-
726	677	E0209a		-	-	293	1.0	865.9	862.8	-3.1	-0.4	-	-	-	-
727	678	E0210a		-	-	298	1.0	864.4	865.7	1.3	0.1	-	-	-	-
728	679	E0211a		473.7	643.0	298	1.0	858.6	859.5	0.9	0.1	-	-	-	-
729	680	E0212a		-	-	277	1.0	875.9	884.2	8.3	1.0	-	-	-	-
730	681	E0213a		-	-	293	1.0	870.0	860.9	-9.1	-1.1	-	-	-	-
731	682	E0214a		-	643.0	293	1.0	872.6	870.4	-2.2	-0.3	-	-	-	-
732	683	E0215a		466.1	643.0	293	1.0	858.0	866.5	8.5	1.0	-	-	-	-
733	684	E0216a		-	-	298	1.0	857.5	857.2	-0.3	-0.0	-	-	-	-
734	685	E0217a		471.8	642.4	298	1.0	868.8	871.8	3.0	0.3	-	-	-	-
735	686	E0218a		-	643.0	298	1.0	858.1	861.8	3.7	0.4	-	-	-	-
736	687	E0219a		486.6	643.0	298	1.0	870.9	875.7	4.9	0.6	61.6	61.9	0.3	0.6
737	688	E0220a		481.6	643.0	298	1.0	862.9	869.3	6.4	0.7	59.5	61.2	1.7	2.8
738	689	E0221a		481.1	643.0	298	1.0	861.6	867.9	6.3	0.7	-	-	-	-
739	690	E0222a		481.1	643.0	298	1.0	862.3	868.2	5.9	0.7	-	-	-	-
740	691	E0223a		476.9	643.0	298	1.0	860.2	868.6	8.4	1.0	-	-	-	-
741	692	E0224a		479.1	643.0	298	1.01	851.0	867.6	16.6	1.9	-	-	-	-
742	693	E0225a		483.1	643.0	298	1.0	864.0	869.5	5.5	0.6	-	-	-	-
743	694	E0226a		484.4	643.0	298	1.0	864.3	874.0	9.7	1.1	-	-	-	-
744	695	E0401a		488.1	-	293	1.0	996.1	956.1	-40.0	-4.0	-	-	-	-
745	696	E0402a		-	-	298	1.0	980.3	947.0	-33.3	-3.4	70.8	68.7	-2.1	-3.0
746	697	E0403a		494.1	-	298	1.0	982.8	957.0	-25.7	-2.6	-	-	-	-
747	698	E0404a		524.0	-	298	1.0	997.4	973.1	-24.2	-2.4	-	-	-	-
748	699	E0405a		518.1	-	298	1.0	1003.7	974.8	-28.9	-2.9	73.0	74.7	1.7	2.3
749	700	E0406a		541.1	-	298	1.0	1019.2	987.8	-31.4	-3.1	78.1	77.2	-0.9	-1.1
750	701	E0407a		513.1	-	298	1.0	995.3	969.4	-25.9	-2.6	73.2	72.7	-0.5	-0.7
751	702	L1101a	×	337.9	512.6	298	1.01	786.7	698.4	-88.4	-11.2	37.7	37.2	-0.5	-1.4
752	703	L2101a		351.4	513.9	298	1.01	786.6	736.3	-50.3	-6.4	42.3	42.5	0.3	0.6
753	704	L2201a	×	470.4	720.0	298	1.0	1109.9	1002.7	-107.2	-9.7	64.8	64.4	-0.4	-0.6
754	705	L3101a		355.4	508.3	298	1.0	781.2	742.9	-38.3	-4.9	44.4	45.3	0.9	2.0
755	706	L3102a		370.4	536.8	298	1.0	799.8	773.1	-26.7	-3.3	46.6	47.2	0.6	1.3
756	707	L3201a	×	460.8	700.2	298	1.0	1032.5	933.5	-99.0	-9.6	62.2	62.2	-0.0	-0.1
757	708	L3202a	×	487.6	724.0	298	1.0	1050.3	976.9	-73.4	-7.0	69.8	60.1	-9.7	-14.0
758	709	L3301a	×	563.1	850.0	298	1.0	1258.3	1045.3	-213.0	-16.9	-	-	-	-
759	709	L3301b	×	563.1	850.0	308	1.0	-	-	-	-	85.8	72.1	-13.7	-16.0
760	710	L4101a		355.6	506.2	299	1.0	779.5	754.8	-24.7	-3.2	46.2	44.0	-2.2	-4.7
761	711	L4102a		380.8	547.8	298	1.0	797.8	782.9	-14.9	-1.9	50.8	50.1	-0.6	-1.3
762	712	L4103a		372.7	536.0	298	1.01	803.0	780.0	-23.0	-2.9	48.5	50.1	1.6	3.3
763	713	L4104a		390.8	563.0	298	1.0	805.8	793.6	-12.2	-1.5	52.1	52.0	-0.1	-0.2
764	714	L4201a		451.1	728.8	298	1.0	989.6	909.9	-79.7	-8.1	-	-	-	-
765	715	L4202a	×	455.1	728.8	298	1.0	999.8	902.7	-97.1	-9.7	-	-	-	-
766	716	L4203a	×	487.1	728.8	293	1.0	1009.0	948.7	-60.3	-6.0	71.3	62.5	-8.8	-12.3

Table S.14 – Comparison of experimental and simulated properties using WH combination rules (continued).

n_{sim}	n_{iso}	Code	Outlier	T_m [K]	T_b [K]	T [K]	P [bar]	ρ_{liq}[kg·m^{-3}] exp	sim	dev	err [%]	ΔH_{vap}[kJ·mol^{-1}] exp	sim	dev	err [%]
767	717	L4204a		464.1	680.0	298	1.0	999.2	927.7	-71.5	-7.2	-	-	-	-
768	718	L4205a	×	480.1	676.0	298	1.0	1000.2	932.1	-68.1	-6.8	72.6	60.1	-12.5	-17.2
769	719	L4206a		501.1	728.8	298	1.0	1015.4	967.5	-47.9	-4.7	76.6	77.3	0.7	0.9
770	720	L4301a	×	-	697.0	298	1.0	1184.0	1058.6	-125.4	-10.6	-	-	-	-
771	721	L5101a		375.1	543.7	298	1.0	804.7	786.0	-18.8	-2.3	50.1	47.9	-2.2	-4.5
772	722	L5102a		384.6	556.1	298	1.0	815.0	799.4	-15.6	-1.9	-	-	-	-
773	723	L5103a		401.9	575.4	298	1.0	815.2	807.1	-8.1	-1.0	54.1	54.8	0.7	1.4
774	724	L5104a		388.4	559.6	298	1.0	815.4	802.1	-13.3	-1.6	52.9	54.4	1.5	2.8
775	725	L5105a		404.4	577.2	298	1.0	806.9	797.4	-9.5	-1.2	55.3	55.5	0.2	0.3
776	726	L5106a		392.1	560.3	298	1.01	805.3	793.0	-12.3	-1.5	53.6	54.4	0.8	1.6
777	727	L5107a		410.9	588.1	298	1.0	811.3	809.2	-2.2	-0.3	56.9	56.5	-0.4	-0.7
778	728	L5201a		447.1	754.5	298	1.0	968.8	908.6	-60.2	-6.2	-	-	-	-
779	729	L5202a		472.1	754.5	293	1.0	964.5	909.4	-55.1	-5.7	-	-	-	-
780	730	L5203a		473.1	754.5	293	1.0	991.7	921.2	-70.5	-7.1	-	-	-	-
781	731	L5204a		473.1	754.5	295	1.0	984.2	926.1	-58.1	-5.9	-	-	-	-
782	732	L5205a		460.6	754.5	292	1.0	979.8	917.3	-62.5	-6.4	-	-	-	-
783	733	L5206a	×	472.1	754.5	298	1.0	956.0	884.7	-71.3	-7.5	72.5	61.6	-10.9	-15.0
784	734	L5207a		475.2	754.5	293	1.0	997.0	939.2	-57.8	-5.8	-	-	-	-
785	735	L5208a		494.1	754.5	293	1.0	981.0	934.8	-46.2	-4.7	-	-	-	-
786	736	L5209a		482.1	754.5	297	1.0	969.1	919.0	-50.1	-5.2	74.6	71.8	-2.8	-3.7
787	737	L5210a		497.0	754.5	293	1.0	989.5	945.0	-44.5	-4.5	-	-	-	-
788	738	L5211a		512.1	754.5	298	1.0	989.7	957.5	-32.2	-3.3	86.8	83.8	-3.0	-3.5
789	739	L5301a		460.6	-	298	1.0	1103.6	1053.3	-50.3	-4.6	-	-	-	-
790	740	L6101a		393.1	596.0	298	0.01	813.9	814.4	0.4	0.1	53.8	53.2	-0.6	-1.0
791	741	L6102a		391.8	596.0	298	1.0	818.6	808.5	-10.1	-1.2	54.0	50.9	-3.1	-5.8
792	742	L6103a		409.9	596.0	298	1.0	824.5	824.0	-0.5	-0.1	-	-	-	-
793	743	L6104a		395.6	575.6	298	1.0	823.8	811.8	-12.0	-1.5	-	-	-	-
794	743	L6104b		395.6	575.6	337	1.0	-	-	-	-	40.1	46.3	6.2	15.4
795	744	L6105a		416.1	596.0	298	1.0	809.7	807.6	-2.1	-0.3	58.0	58.3	0.3	0.4
796	745	L6106a		394.6	559.5	298	1.0	809.5	795.6	-13.8	-1.7	54.7	52.5	-2.2	-3.9
797	746	L6107a		422.1	596.0	298	1.0	823.7	819.2	-4.5	-0.5	-	-	-	-
798	747	L6108a		407.4	596.0	298	1.0	824.7	815.9	-8.8	-1.1	58.2	57.0	-1.2	-2.1
799	748	L6109a		399.7	596.0	298	1.0	820.1	810.0	-10.1	-1.2	56.0	56.5	0.5	0.9
800	749	L6110a		404.9	574.4	298	1.0	803.0	798.1	-4.9	-0.6	-	-	-	-
801	749	L6110b		404.9	574.4	308	1.0	-	-	-	-	49.6	57.4	7.8	15.7
802	750	L6111a		419.6	596.0	298	1.0	829.3	824.5	-4.8	-0.6	60.3	59.5	-0.8	-1.3
803	751	L6112a		425.6	596.0	298	1.0	820.5	818.0	-2.5	-0.3	61.7	60.2	-1.5	-2.4
804	752	L6113a		421.1	604.4	298	1.0	820.6	817.6	-3.0	-0.4	59.4	59.5	0.1	0.2
805	753	L6114a		408.6	596.0	298	1.0	814.5	812.5	-2.0	-0.2	58.6	58.9	0.3	0.5
806	754	L6115a		424.9	603.5	298	1.0	809.7	812.0	2.3	0.3	-	-	-	-
807	755	L6116a		413.0	585.9	298	1.0	810.3	807.8	-2.6	-0.3	58.3	59.0	0.7	1.1
808	756	L6117a		430.1	610.3	298	1.0	815.5	820.8	5.2	0.6	59.6	61.1	1.5	2.6
809	757	L6201a		445.9	777.8	316	0.002	970.4	908.7	-61.7	-6.4	-	-	-	-
810	758	L6202a		505.8	777.8	298	1.0	963.8	914.9	-48.9	-5.1	-	-	-	-
811	759	L6203a		478.6	777.8	323	1.0	940.0	900.1	-39.9	-4.2	-	-	-	-
812	760	L6204a		480.0	777.8	298	1.0	964.5	910.8	-53.7	-5.6	-	-	-	-
813	761	L6205a		505.8	777.8	293	1.0	962.7	903.1	-59.6	-6.2	-	-	-	-
814	762	L6206a		470.6	777.8	298	1.0	918.5	869.1	-49.4	-5.4	68.6	61.2	-7.4	-10.8

Table S.14 – Comparison of experimental and simulated properties using WH combination rules (continued).

n_{sim}	n_{iso}	Code	Outlier	T_m [K]	T_b [K]	T [K]	P [bar]	ρ_{liq}[kg·m^{-3}] exp	sim	dev	err [%]	ΔH_{vap}[kJ·mol^{-1}] exp	sim	dev	err [%]
815	763	L6207a	×	484.6	777.8	287	1.0	990.6	903.2	-87.4	-8.8	-	-	-	-
816	764	L6208a		498.9	777.8	323	1.0	958.2	909.1	-49.1	-5.1	-	-	-	-
817	765	L6209a		480.0	777.8	277	1.0	996.0	964.2	-31.8	-3.2	-	-	-	-
818	766	L6210a		483.0	777.8	293	1.0	969.0	922.8	-46.2	-4.8	-	-	-	-
819	767	L6211a		494.4	777.8	293	1.0	964.5	933.0	-31.5	-3.3	-	-	-	-
820	768	L6212a		496.9	777.8	293	1.0	976.8	923.6	-53.2	-5.4	-	-	-	-
821	769	L6213a		480.0	777.8	298	1.0	967.7	908.9	-58.8	-6.1	-	-	-	-
822	770	L6214a		-	-	293	1.0	977.1	955.5	-21.6	-2.2	-	-	-	-
823	771	L6215a		493.4	777.8	295	1.0	973.7	915.5	-58.2	-6.0	-	-	-	-
824	772	L6216a		484.1	777.8	294	1.0	951.6	896.7	-54.9	-5.8	-	-	-	-
825	773	L6217a		493.9	777.8	323	1.0	939.8	908.2	-31.6	-3.4	-	-	-	-
826	774	L6218a		474.2	777.8	298	1.0	963.6	925.7	-37.9	-3.9	-	-	-	-
827	775	L6219a		521.5	777.8	293	1.0	972.6	950.6	-22.0	-2.3	-	-	-	-
828	776	L6220a		496.9	777.8	293	1.0	971.9	950.0	-21.9	-2.3	-	-	-	-
829	777	L6221a		508.1	777.8	295	1.0	958.0	925.1	-32.9	-3.4	-	-	-	-
830	778	L6222a		529.1	777.8	289	1.0	982.0	946.0	-36.0	-3.7	-	-	-	-
831	779	L6223a		497.1	777.8	385	0.01	917.4	837.8	-79.7	-8.7	-	-	-	-
832	780	L6224a		510.1	777.8	298	1.0	964.0	934.5	-29.5	-3.1	-	-	-	-
833	781	L6225a		516.1	777.8	318	1.0	968.3	936.8	-31.5	-3.3	-	-	-	-
834	781	L6225b		516.1	777.8	342	1.0	-	-	-	-	87.0	84.5	-2.5	-2.9
835	782	L6301a		-	-	293	1.0	1104.1	1049.1	-55.0	-5.0	-	-	-	-
836	783	L6302a		472.5	-	298	1.0	1100.0	1029.9	-70.1	-6.4	-	-	-	-
837	784	L7101a		404.1	619.7	298	1.0	833.5	838.1	4.6	0.5	-	-	-	-
838	784	L7101b		404.1	619.7	313	1.0	-	-	-	-	48.7	51.4	2.7	5.5
839	785	L7102a		430.1	619.7	293	1.0	846.6	849.3	2.7	0.3	-	-	-	-
840	786	L7103a		420.1	619.7	293	1.0	827.0	841.5	14.5	1.8	-	-	-	-
841	787	L7104a		413.1	619.7	298	1.0	837.3	831.9	-5.3	-0.6	-	-	-	-
842	788	L7105a		433.1	619.7	298	1.0	823.8	836.7	12.9	1.6	-	-	-	-
843	789	L7106a		409.1	619.7	298	1.0	822.4	825.0	2.6	0.3	-	-	-	-
844	790	L7107a		412.1	619.7	298	1.0	828.5	824.1	-4.4	-0.5	-	-	-	-
845	791	L7108a		411.1	619.7	293	1.0	811.9	815.4	3.5	0.4	-	-	-	-
846	792	L7109a		406.1	619.7	298	1.0	810.0	801.7	-8.3	-1.0	-	-	-	-
847	793	L7110a		426.1	619.7	294	1.0	836.0	827.6	-8.4	-1.0	-	-	-	-
848	794	L7111a		411.9	619.7	298	1.0	824.9	819.3	-5.6	-0.7	-	-	-	-
849	794	L7111b		411.9	619.7	322	1.0	-	-	-	-	53.6	54.5	0.9	1.7
850	795	L7112a		430.1	619.7	293	1.0	828.2	850.6	22.4	2.7	-	-	-	-
851	796	L7113a		415.6	619.7	298	1.0	839.6	833.4	-6.2	-0.7	57.3	54.4	-2.9	-5.1
852	797	L7114a		438.1	619.7	293	1.0	832.0	838.0	6.0	0.7	-	-	-	-
853	798	L7115a		426.1	619.7	293	1.0	837.9	833.8	-4.1	-0.5	-	-	-	-
854	799	L7116a		415.9	619.7	298	1.0	820.2	818.5	-1.7	-0.2	-	-	-	-
855	800	L7117a		433.1	619.7	293	1.0	815.1	824.1	9.0	1.1	-	-	-	-
856	801	L7118a		415.9	619.7	298	1.0	809.8	807.6	-2.2	-0.3	58.6	57.0	-1.6	-2.7
857	802	L7119a		435.1	619.7	298	1.0	832.7	832.8	0.1	0.0	-	-	-	-
858	803	L7120a		425.1	619.7	298	1.0	833.3	824.0	-9.3	-1.1	-	-	-	-
859	804	L7121a		437.1	619.7	296	1.0	836.0	837.1	1.1	0.1	-	-	-	-
860	805	L7122a		438.1	619.7	297	1.0	819.0	830.5	11.5	1.4	-	-	-	-
861	806	L7123a		425.1	619.7	298	1.0	822.0	822.2	0.2	0.0	-	-	-	-
862	807	L7124a		418.1	619.7	293	1.0	823.9	821.7	-2.2	-0.3	-	-	-	-

Table S.14 – Comparison of experimental and simulated properties using WH combination rules (continued).

n_{sim}	n_{iso}	Code	Outlier	T_m [K]	T_b [K]	T [K]	P [bar]	ρ_{liq}[kg·m^{-3}] exp	sim	dev	err [%]	ΔH_{vap}[kJ·mol^{-1}] exp	sim	dev	err [%]
863	808	L7125a		432.1	619.7	298	1.0	816.0	819.0	3.0	0.4	-	-	-	-
864	809	L7126a		424.1	619.7	298	1.0	817.7	816.3	-1.4	-0.2	-	-	-	-
865	810	L7127a		421.1	619.7	293	1.0	833.1	817.1	-16.0	-1.9	59.8	62.6	2.8	4.7
866	811	L7128a		424.1	619.7	293	1.0	813.1	813.1	0.0	0.0	-	-	-	-
867	812	L7129a		439.1	619.7	298	1.0	828.8	831.9	3.1	0.4	-	-	-	-
868	813	L7130a		445.1	619.7	298	1.0	824.5	826.8	2.3	0.3	-	-	-	-
869	814	L7131a		446.1	619.7	297	1.0	821.0	828.4	7.4	0.9	-	-	-	-
870	815	L7132a		436.1	619.7	293	1.0	827.0	831.8	4.8	0.6	-	-	-	-
871	816	L7133a		427.9	619.7	298	1.0	815.6	820.2	4.6	0.6	62.4	63.5	1.1	1.7
872	817	L7134a		429.9	619.7	298	1.0	816.5	820.4	3.9	0.5	-	-	-	-
873	818	L7135a		445.1	619.7	298	1.0	819.2	821.7	2.5	0.3	-	-	-	-
874	819	L7136a		432.4	608.3	298	1.0	813.4	816.4	3.0	0.4	62.1	63.3	1.2	1.9
875	820	L7137a		449.4	632.6	298	1.0	819.2	830.1	11.0	1.3	66.5	65.8	-0.7	-1.1
876	821	L7201a		525.9	799.3	293	1.0	961.3	940.6	-20.7	-2.2	-	-	-	-
877	822	L7202a		517.0	799.3	293	1.0	952.8	948.7	-4.1	-0.4	-	-	-	-
878	823	L7203a		517.0	799.3	293	1.0	929.6	886.5	-43.1	-4.6	-	-	-	-
879	824	L7204a		517.0	799.3	291	1.0	932.1	878.3	-53.8	-5.8	-	-	-	-
880	825	L7205a		507.1	799.3	334	1.0	949.0	906.8	-42.2	-4.4	-	-	-	-
881	826	L7206a		494.2	799.3	295	1.0	967.0	928.3	-38.7	-4.0	-	-	-	-
882	827	L7207a		508.2	799.3	293	1.0	967.2	947.5	-19.7	-2.0	-	-	-	-
883	828	L7208a		508.2	799.3	298	1.0	926.0	889.2	-36.8	-4.0	-	-	-	-
884	829	L7209a		485.4	799.3	293	1.0	962.5	945.3	-17.2	-1.8	-	-	-	-
885	830	L7210a		485.4	799.3	293	1.0	967.9	948.7	-19.2	-2.0	-	-	-	-
886	831	L7211a		515.1	799.3	298	1.0	950.4	937.0	-13.4	-1.4	-	-	-	-
887	832	L7212a		489.8	799.3	293	1.0	970.5	939.6	-30.9	-3.2	-	-	-	-
888	833	L7213a		485.4	799.3	298	1.0	962.0	929.8	-32.2	-3.3	-	-	-	-
889	834	L7214a		535.1	799.3	298	1.0	952.2	946.5	-5.8	-0.6	96.5	95.0	-1.5	-1.5
890	835	L7301a		416.9	-	291	1.0	1075.0	1037.9	-37.1	-3.5	-	-	-	-
891	836	L8101a		433.1	641.4	298	1.0	815.1	857.5	42.4	5.2	-	-	-	-
892	837	L8102a		425.1	641.4	298	1.0	842.9	856.3	13.4	1.6	-	-	-	-
893	838	L8103a		419.6	641.4	293	1.0	823.7	818.3	-5.4	-0.7	-	-	-	-
894	839	L8104a		430.1	641.4	293	1.0	849.2	852.0	2.8	0.3	-	-	-	-
895	840	L8105a		431.1	641.4	293	1.0	840.8	842.6	1.8	0.2	-	-	-	-
896	841	L8106a		424.1	641.4	293	1.0	832.4	835.9	3.5	0.4	-	-	-	-
897	842	L8107a		431.1	641.4	293	1.0	808.0	837.4	29.4	3.6	-	-	-	-
898	843	L8108a		433.0	641.4	293	1.0	857.6	860.4	2.8	0.3	-	-	-	-
899	844	L8109a		433.1	641.4	293	1.0	829.5	851.6	22.1	2.7	-	-	-	-
900	845	L8110a		432.1	641.4	293	1.0	834.1	850.1	16.0	1.9	-	-	-	-
901	846	L8111a		433.0	641.4	293	1.0	845.7	846.5	0.8	0.1	-	-	-	-
902	847	L8112a		425.1	641.4	298	1.0	834.5	842.7	8.2	1.0	-	-	-	-
903	848	L8113a		431.4	641.4	293	1.0	837.1	840.2	3.1	0.4	-	-	-	-
904	849	L8114a		446.0	641.4	298	1.0	842.5	850.1	7.6	0.9	-	-	-	-
905	850	L8115a		429.2	641.4	293	1.0	834.2	837.2	3.0	0.4	-	-	-	-
906	851	L8116a		430.9	641.4	298	1.0	834.6	830.6	-4.0	-0.5	-	-	-	-
907	852	L8117a		433.2	641.4	298	1.0	831.0	830.9	-0.1	-0.0	-	-	-	-
908	853	L8118a		441.4	641.4	293	1.0	838.4	834.9	-3.5	-0.4	-	-	-	-
909	853	L8118b		441.4	641.4	348	1.0	-	-	-	-	54.7	56.7	2.0	3.7
910	854	L8119a		425.1	641.4	298	1.0	827.0	822.5	-4.5	-0.5	-	-	-	-

Table S.14 – Comparison of experimental and simulated properties using WH combination rules (continued).

n_{sim}	n_{iso}	Code	Outlier	T_m	T_b	T	P	ρ_{liq}[kg·m^{-3}]				ΔH_{vap}[kJ·mol^{-1}]			
				[K]	[K]	[K]	[bar]	exp	sim	dev	err [%]	exp	sim	dev	err [%]
911	855	L8120a		444.1	641.4	293	1.0	833.0	830.7	-2.3	-0.3	-	-	-	-
912	856	L8121a		426.1	641.4	293	1.0	833.9	825.9	-8.0	-1.0	-	-	-	-
913	857	L8122a		423.9	641.4	301	1.0	806.5	815.3	8.8	1.1	-	-	-	-
914	858	L8123a		425.6	641.4	298	1.0	811.5	810.2	-1.3	-0.2	-	-	-	-
915	859	L8124a		446.1	641.4	298	1.0	842.5	843.8	1.3	0.2	-	-	-	-
916	860	L8125a		456.1	641.4	293	1.0	849.8	848.7	-1.1	-0.1	-	-	-	-
917	861	L8126a		432.1	641.4	298	1.0	813.5	820.0	6.5	0.8	-	-	-	-
918	862	L8127a		432.1	641.4	298	1.0	834.2	838.3	4.1	0.5	-	-	-	-
919	863	L8128a		454.0	641.4	293	1.0	839.0	844.0	5.0	0.6	-	-	-	-
920	864	L8129a		445.6	641.4	293	1.0	826.5	842.7	16.2	2.0	-	-	-	-
921	865	L8130a		434.2	641.4	298	1.0	820.2	825.2	5.0	0.6	-	-	-	-
922	865	L8130b		434.2	641.4	345	1.0	-	-	-	-	54.8	54.2	-0.6	-1.1
923	866	L8131a		434.1	641.4	298	1.0	824.9	827.9	3.0	0.4	-	-	-	-
924	866	L8131b		434.1	641.4	353	1.0	-	-	-	-	54.7	52.6	-2.1	-3.8
925	867	L8132a		429.9	641.4	298	1.0	805.0	815.8	10.8	1.3	-	-	-	-
926	867	L8132b		429.9	641.4	358	1.0	-	-	-	-	53.1	52.8	-0.3	-0.6
927	868	L8133a		437.1	641.4	332	0.01	812.9	807.2	-5.7	-0.7	-	-	-	-
928	869	L8134a		428.6	641.4	298	1.0	794.2	830.7	36.5	4.6	-	-	-	-
929	869	L8134b	×	428.6	641.4	319	0.01	-	-	-	-	43.5	63.0	19.5	44.8
930	870	L8135a		437.9	641.4	298	1.0	833.5	830.1	-3.4	-0.4	-	-	-	-
931	870	L8135b	×	437.9	641.4	355	1.0	-	-	-	-	48.0	57.4	9.4	19.6
932	871	L8136a		439.2	641.4	298	1.0	817.6	829.4	11.8	1.4	-	-	-	-
933	871	L8136b	×	439.2	641.4	356	1.0	-	-	-	-	48.0	57.8	9.8	20.5
934	872	L8137a		440.8	641.4	298	1.0	821.0	825.4	4.4	0.5	-	-	-	-
935	872	L8137b		440.8	641.4	364	1.0	-	-	-	-	54.8	55.4	0.6	1.1
936	873	L8138a		449.6	641.4	293	1.0	827.3	834.6	7.3	0.9	-	-	-	-
937	873	L8138b		449.6	641.4	365	1.0	-	-	-	-	53.3	58.5	5.2	9.8
938	874	L8139a		426.8	641.4	298	1.0	814.1	826.5	12.3	1.5	-	-	-	-
939	875	L8140a		456.1	641.4	293	1.0	828.2	830.6	2.4	0.3	-	-	-	-
940	876	L8141a		444.8	641.4	298	1.0	799.0	821.6	22.6	2.8	-	-	-	-
941	877	L8142a		452.1	641.4	298	1.0	824.5	827.4	2.8	0.3	-	-	-	-
942	878	L8143a		445.1	641.4	298	1.0	810.0	823.2	13.2	1.6	-	-	-	-
943	879	L8144a		439.2	641.4	298	1.0	809.8	817.6	7.8	1.0	-	-	-	-
944	880	L8145a		433.0	641.4	298	1.0	776.6	819.9	43.3	5.6	-	-	-	-
945	881	L8146a		445.1	641.4	298	1.0	803.4	817.0	13.6	1.7	-	-	-	-
946	882	L8147a		453.0	641.4	301	1.0	829.0	835.9	6.9	0.8	-	-	-	-
947	883	L8148a		457.8	640.6	298	1.0	828.7	838.5	9.8	1.2	68.5	68.3	-0.2	-0.3
948	884	L8149a		456.4	641.4	298	1.0	806.0	834.6	28.6	3.6	-	-	-	-
949	885	L8150a		459.1	641.4	298	1.0	784.5	835.0	50.5	6.4	-	-	-	-
950	886	L8151a		459.8	641.4	298	1.0	815.2	835.0	19.8	2.4	-	-	-	-
951	887	L8152a		448.8	641.4	298	1.0	798.7	834.4	35.7	4.5	-	-	-	-
952	888	L8153a		449.8	641.4	298	1.0	815.9	825.9	10.0	1.2	67.2	67.5	0.3	0.5
953	889	L8154a		447.9	641.4	298	1.0	817.0	826.8	9.8	1.2	-	-	-	-
954	890	L8155a		460.9	641.4	298	1.0	817.6	829.2	11.7	1.4	-	-	-	-
955	891	L8156a		452.9	629.6	298	1.0	817.0	825.1	8.1	1.0	67.9	67.7	-0.2	-0.2
956	892	L8157a		468.4	652.5	298	1.0	821.8	837.3	15.5	1.9	70.1	70.2	0.1	0.2
957	893	L8201a		-	-	298	1.0	928.5	901.1	-27.4	-3.0	-	-	-	-
958	894	L8202a		-	-	293	1.0	917.2	875.4	-41.8	-4.6	-	-	-	-

Table S.14 – Comparison of experimental and simulated properties using WH combination rules (continued).

n_{sim}	n_{iso}	Code	Outlier	T_m [K]	T_b [K]	T [K]	P [bar]	ρ_{liq}[kg·m^{-3}] exp	sim	dev	err [%]	ΔH_{vap}[kJ·mol^{-1}] exp	sim	dev	err [%]
959	895	L8203a		-	-	298	1.0	929.0	898.5	-30.5	-3.3	-	-	-	-
960	896	L8204a		-	-	292	1.0	970.4	950.3	-20.1	-2.1	-	-	-	-
961	897	L8205a		-	-	298	1.0	959.0	926.9	-32.1	-3.3	-	-	-	-
962	898	L8206a		517.1	819.3	295	1.0	932.5	912.4	-20.1	-2.2	-	-	-	-
963	899	L8207a		478.1	819.3	298	1.0	918.0	890.4	-27.6	-3.0	-	-	-	-
964	900	L8208a		-	-	298	1.0	949.0	931.8	-17.2	-1.8	-	-	-	-
965	901	L8209a		-	-	298	1.0	943.0	927.8	-15.2	-1.6	-	-	-	-
966	902	L8210a	×	544.1	819.3	356	1.0	-	-	-	-	101.0	90.9	-10.1	-10.0
967	903	L9101a		447.1	661.5	298	1.0	852.6	855.9	3.2	0.4	-	-	-	-
968	904	L9102a		439.1	661.5	294	1.0	832.3	877.4	45.1	5.4	-	-	-	-
969	905	L9103a		446.2	661.5	293	1.0	846.2	864.9	18.7	2.2	-	-	-	-
970	906	L9104a		466.0	661.5	293	1.0	847.5	838.4	-9.1	-1.1	-	-	-	-
971	907	L9105a		439.0	661.5	293	1.0	835.0	833.2	-1.8	-0.2	-	-	-	-
972	908	L9106a		451.1	661.5	298	1.0	854.9	849.8	-5.2	-0.6	-	-	-	-
973	909	L9107a		444.1	661.5	293	1.0	848.8	855.3	6.5	0.8	-	-	-	-
974	910	L9108a		439.0	661.5	293	1.0	825.6	839.6	14.0	1.7	-	-	-	-
975	911	L9109a		466.0	661.5	288	1.0	835.3	853.5	18.2	2.2	-	-	-	-
976	912	L9110a		442.0	661.5	293	1.0	825.0	833.2	8.2	1.0	-	-	-	-
977	913	L9111a		452.0	661.5	288	1.0	861.0	866.6	5.6	0.6	-	-	-	-
978	914	L9112a		457.2	661.5	298	1.0	844.5	850.9	6.4	0.8	-	-	-	-
979	915	L9113a		452.0	661.5	298	1.0	899.4	848.4	-51.0	-5.7	-	-	-	-
980	916	L9114a		447.1	661.5	294	1.0	838.3	846.6	8.3	1.0	-	-	-	-
981	917	L9115a		475.1	661.5	293	1.0	846.0	855.2	9.2	1.1	-	-	-	-
982	918	L9116a		451.0	661.5	298	1.0	823.7	837.8	14.1	1.7	-	-	-	-
983	919	L9117a		451.1	661.5	298	1.0	833.4	837.4	4.0	0.5	-	-	-	-
984	920	L9118a		461.1	661.5	298	1.0	837.0	847.1	10.1	1.2	-	-	-	-
985	921	L9119a		445.1	661.5	295	1.0	839.6	837.2	-2.4	-0.3	-	-	-	-
986	922	L9120a		450.0	661.5	298	1.0	821.8	834.8	13.0	1.6	-	-	-	-
987	923	L9121a		444.6	661.5	293	1.0	824.2	832.7	8.5	1.0	-	-	-	-
988	924	L9122a		446.1	661.5	289	1.0	828.5	834.8	6.3	0.8	-	-	-	-
989	925	L9123a		466.1	661.5	298	1.0	823.6	833.4	9.8	1.2	67.9	70.1	2.2	3.2
990	926	L9124a		455.0	661.5	298	1.0	828.0	824.2	-3.8	-0.5	-	-	-	-
991	927	L9125a		-	-	295	1.0	830.0	825.7	-4.3	-0.5	-	-	-	-
992	928	L9126a		444.1	661.5	293	1.0	818.6	820.6	2.0	0.2	-	-	-	-
993	929	L9127a		452.0	661.5	293	1.0	827.5	844.8	17.3	2.1	-	-	-	-
994	930	L9128a		460.1	661.5	293	1.0	859.2	846.1	-13.1	-1.5	-	-	-	-
995	931	L9129a		448.1	661.5	293	1.0	814.8	828.7	13.9	1.7	-	-	-	-
996	932	L9130a		467.1	661.5	273	1.0	878.7	841.4	-37.3	-4.2	-	-	-	-
997	933	L9131a		451.0	661.5	298	1.0	806.4	817.9	11.4	1.4	65.2	70.0	4.8	7.3
998	934	L9132a		452.1	661.5	298	1.0	829.9	840.5	10.6	1.3	-	-	-	-
999	935	L9133a		455.4	661.5	298	1.0	829.9	843.7	13.8	1.7	-	-	-	-
1000	936	L9134a		454.1	661.5	298	1.0	824.6	833.7	9.1	1.1	-	-	-	-
1001	937	L9135a		-	-	298	1.0	827.5	834.0	6.5	0.8	-	-	-	-
1002	938	L9136a		455.0	661.5	293	1.0	843.9	838.9	-5.0	-0.6	-	-	-	-
1003	939	L9137a		451.1	661.5	298	1.0	813.4	824.3	10.9	1.3	-	-	-	-
1004	940	L9138a		466.1	661.5	298	1.0	835.8	848.7	12.9	1.5	-	-	-	-
1005	941	L9139a		455.0	661.5	298	1.0	815.6	836.2	20.6	2.5	-	-	-	-
1006	942	L9140a		459.0	661.5	298	1.0	843.7	837.2	-6.5	-0.8	-	-	-	-

Table S.14 – Comparison of experimental and simulated properties using WH combination rules (continued).

n_{sim}	n_{iso}	Code	Outlier	T_m [K]	T_b [K]	T [K]	P [bar]	$\rho_{\mathrm{liq}}[\mathrm{kg \cdot m^{-3}}]$ exp	sim	dev	err [%]	$\Delta H_{\mathrm{vap}}[\mathrm{kJ \cdot mol^{-1}}]$ exp	sim	dev	err [%]
1007	943	L9141a		453.1	661.5	291	1.0	834.0	840.5	6.5	0.8	-	-	-	-
1008	944	L9142a		454.0	661.5	300	1.0	831.0	834.2	3.2	0.4	-	-	-	-
1009	945	L9143a		457.1	661.5	293	1.0	827.0	835.5	8.5	1.0	-	-	-	-
1010	946	L9144a		468.1	661.5	293	1.0	828.8	845.0	16.2	2.0	-	-	-	-
1011	947	L9145a		465.1	661.5	293	1.0	825.6	837.1	11.5	1.4	-	-	-	-
1012	948	L9146a		466.0	661.5	298	1.0	820.8	836.4	15.6	1.9	-	-	-	-
1013	949	L9147a		455.0	661.5	296	1.0	822.0	832.0	10.0	1.2	-	-	-	-
1014	950	L9148a		459.0	661.5	301	1.0	832.0	829.6	-2.4	-0.3	-	-	-	-
1015	951	L9149a		-	-	300	1.0	823.0	832.0	9.0	1.1	-	-	-	-
1016	952	L9150a		454.0	661.5	298	1.0	821.0	829.0	8.0	1.0	-	-	-	-
1017	953	L9151a		457.1	661.5	298	1.0	815.0	826.1	11.1	1.4	-	-	-	-
1018	954	L9152a		455.0	661.5	293	1.0	813.6	830.0	16.4	2.0	-	-	-	-
1019	955	L9153a		459.0	661.5	285	1.0	840.2	837.3	-2.9	-0.4	-	-	-	-
1020	956	L9154a		480.1	661.5	296	1.0	834.0	845.2	11.2	1.3	-	-	-	-
1021	957	L9155a		472.0	661.5	298	1.0	848.0	844.8	-3.2	-0.4	-	-	-	-
1022	958	L9156a		473.0	661.5	301	1.0	820.0	839.2	19.2	2.3	-	-	-	-
1023	959	L9157a		473.0	661.5	297	1.0	828.0	841.9	13.9	1.7	-	-	-	-
1024	960	L9158a		473.0	661.5	297	1.0	827.0	841.3	14.3	1.7	-	-	-	-
1025	961	L9159a		473.0	661.5	277	1.0	841.8	854.4	12.6	1.5	-	-	-	-
1026	962	L9160a		468.2	661.5	298	1.0	818.3	832.1	13.8	1.7	71.4	71.8	0.4	0.6
1027	963	L9161a		466.1	661.5	293	1.0	826.3	835.5	9.2	1.1	71.5	72.5	1.0	1.4
1028	964	L9162a		467.9	661.5	298	1.0	823.5	834.3	10.7	1.3	70.9	72.1	1.2	1.7
1029	965	L9163a		479.1	661.5	298	1.0	826.0	836.4	10.4	1.3	-	-	-	-
1030	966	L9164a		471.6	649.6	298	1.0	819.4	832.0	12.7	1.5	72.9	72.1	-0.8	-1.0
1031	967	L9165a		486.2	670.7	298	1.0	824.6	843.1	18.5	2.2	72.2	74.9	2.7	3.7
1032	968	L9201a		-	-	301	1.0	920.7	879.2	-41.5	-4.5	-	-	-	-
1033	969	L9202a		-	-	290	1.0	913.8	881.5	-32.3	-3.5	-	-	-	-
1034	970	L9203a		-	-	291	1.0	902.0	873.7	-28.3	-3.1	-	-	-	-
1035	971	L9204a		535.1	837.9	323	1.0	929.0	909.1	-19.9	-2.1	-	-	-	-
1036	971	L9204b		535.1	837.9	460	1.0	-	-	-	-	67.2	62.5	-4.7	-7.0
1037	972	L9205a		-	-	291	1.0	958.0	947.7	-10.3	-1.1	-	-	-	-
1038	973	L9206a		-	-	293	1.0	941.6	932.9	-8.7	-0.9	-	-	-	-
1039	974	L9207a		-	-	293	1.0	937.0	930.3	-6.7	-0.7	-	-	-	-
1040	975	L9208a		571.1	837.9	293	1.0	929.5	931.5	2.0	0.2	-	-	-	-
1041	976	L9209a		-	-	298	1.0	944.0	931.5	-12.5	-1.3	-	-	-	-
1042	977	L9210a	×	558.1	837.9	323	1.0	-	-	-	-	110.0	99.2	-10.8	-9.8
1043	978	L0101a		464.1	680.4	293	1.0	862.4	865.0	2.6	0.3	-	-	-	-
1044	979	L0102a		475.1	680.4	293	1.0	874.2	886.9	12.7	1.4	-	-	-	-
1045	980	L0103a		474.1	680.4	293	1.0	874.5	880.8	6.3	0.7	-	-	-	-
1046	981	L0104a		465.1	680.4	293	1.0	858.4	872.4	14.0	1.6	-	-	-	-
1047	982	L0105a		513.5	680.4	293	1.0	839.3	850.9	11.6	1.4	-	-	-	-
1048	983	L0106a		463.1	680.4	293	1.0	854.9	874.7	19.8	2.3	-	-	-	-
1049	984	L0107a		468.1	680.4	293	1.0	862.3	871.6	9.3	1.1	-	-	-	-
1050	985	L0108a		475.0	680.4	293	1.0	837.8	841.7	3.9	0.5	-	-	-	-
1051	986	L0109a		467.6	680.4	298	1.0	859.1	856.8	-2.3	-0.3	-	-	-	-
1052	987	L0110a		459.1	680.4	293	1.0	848.7	866.1	17.4	2.0	-	-	-	-
1053	988	L0111a		460.0	680.4	293	1.0	842.8	842.2	-0.6	-0.1	-	-	-	-
1054	989	L0112a		468.8	680.4	293	1.0	854.3	850.9	-3.4	-0.4	-	-	-	-

Table S.14 – Comparison of experimental and simulated properties using WH combination rules (continued).

n_{sim}	n_{iso}	Code	Outlier	T_m [K]	T_b [K]	T [K]	P [bar]	ρ_{liq}[kg·m^{-3}] exp	sim	dev	err [%]	ΔH_{vap}[kJ·mol^{-1}] exp	sim	dev	err [%]
1055	990	L0113a		503.4	687.3	298	1.0	-	-	-	-	80.9	79.4	-1.5	-1.8
1056	991	L0114a		454.1	680.4	293	1.0	833.0	839.0	6.0	0.7	-	-	-	-
1057	992	L0115a		465.1	680.4	293	1.0	853.7	856.6	2.9	0.3	-	-	-	-
1058	993	L0116a		460.0	680.4	293	1.0	860.6	871.5	10.9	1.3	-	-	-	-
1059	994	L0117a		463.0	680.4	293	1.0	839.8	846.4	6.6	0.8	-	-	-	-
1060	995	L0118a		460.1	680.4	293	1.0	833.9	855.1	21.2	2.5	-	-	-	-
1061	996	L0119a		463.0	680.4	293	1.0	823.6	840.5	16.9	2.0	-	-	-	-
1062	997	L0120a		455.1	680.4	294	1.0	823.0	829.6	6.6	0.8	-	-	-	-
1063	998	L0121a		466.0	680.4	293	1.0	826.5	833.9	7.4	0.9	-	-	-	-
1064	999	L0122a		466.1	680.4	291	1.0	833.0	835.1	2.1	0.3	-	-	-	-
1065	1000	L0123a		463.1	680.4	298	1.0	841.3	853.1	11.7	1.4	-	-	-	-
1066	1001	L0124a		466.1	680.4	293	1.0	845.5	858.9	13.4	1.6	-	-	-	-
1067	1002	L0125a		462.2	680.4	298	1.0	824.9	847.4	22.5	2.7	-	-	-	-
1068	1003	L0126a		464.0	680.4	301	1.0	825.8	837.0	11.2	1.4	-	-	-	-
1069	1004	L0127a		466.0	680.4	298	1.0	837.0	839.8	2.8	0.3	-	-	-	-
1070	1005	L0128a		464.0	680.4	293	1.0	823.2	838.6	15.4	1.9	-	-	-	-
1071	1006	L0129a		465.1	680.4	295	1.0	834.7	840.8	6.1	0.7	-	-	-	-
1072	1007	L0130a		465.1	680.4	273	1.0	842.1	851.9	9.8	1.2	-	-	-	-
1073	1008	L0131a		469.1	680.4	298	1.0	826.0	832.9	6.9	0.8	-	-	-	-
1074	1009	L0132a		463.1	680.4	293	1.0	821.2	836.7	15.5	1.9	-	-	-	-
1075	1010	L0133a		485.0	680.4	293	1.0	825.7	835.3	9.6	1.2	-	-	-	-
1076	1011	L0134a		485.0	680.4	293	1.0	827.3	831.9	4.6	0.6	-	-	-	-
1077	1012	L0135a		485.0	680.4	298	1.0	821.1	825.5	4.4	0.5	-	-	-	-
1078	1013	L0136a		466.0	680.4	290	1.0	865.0	854.3	-10.7	-1.2	-	-	-	-
1079	1014	L0137a		486.1	680.4	293	1.0	832.2	843.9	11.7	1.4	-	-	-	-
1080	1015	L0138a		464.0	680.4	298	1.0	821.5	837.2	15.7	1.9	-	-	-	-
1081	1016	L0139a		485.0	680.4	293	1.0	829.1	837.2	8.1	1.0	-	-	-	-
1082	1017	L0140a		467.1	680.4	293	1.0	815.2	836.1	20.9	2.6	-	-	-	-
1083	1018	L0141a		477.1	680.4	277	1.0	846.0	848.5	2.5	0.3	-	-	-	-
1084	1019	L0142a		475.1	680.4	293	1.0	814.0	830.3	16.3	2.0	-	-	-	-
1085	1020	L0143a		467.1	680.4	298	1.0	828.3	844.5	16.2	2.0	-	-	-	-
1086	1021	L0144a		466.0	680.4	298	1.0	826.8	846.9	20.1	2.4	-	-	-	-
1087	1022	L0145a		472.1	680.4	298	1.0	836.1	847.2	11.1	1.3	-	-	-	-
1088	1023	L0146a		481.1	680.4	293	1.0	830.0	851.9	21.9	2.6	-	-	-	-
1089	1024	L0147a		475.1	680.4	298	1.0	825.4	840.0	14.6	1.8	-	-	-	-
1090	1025	L0148a		482.0	680.4	298	1.0	827.1	837.9	10.8	1.3	-	-	-	-
1091	1026	L0149a		482.0	680.4	298	1.0	826.5	840.4	14.0	1.7	-	-	-	-
1092	1027	L0150a		487.0	680.4	298	1.0	807.8	830.7	22.9	2.8	-	-	-	-
1093	1028	L0151a		488.4	680.4	303	1.0	834.1	844.0	9.9	1.2	-	-	-	-
1094	1029	L0152a		482.0	680.4	300	1.0	826.0	840.9	14.9	1.8	-	-	-	-
1095	1030	L0153a		487.0	680.4	295	1.0	845.0	842.2	-2.8	-0.3	-	-	-	-
1096	1031	L0154a		481.6	680.4	298	1.0	824.5	838.1	13.6	1.6	-	-	-	-
1097	1032	L0155a		481.1	680.4	293	1.0	828.6	849.2	20.6	2.5	-	-	-	-
1098	1033	L0156a		472.0	680.4	298	1.0	839.6	840.3	0.7	0.1	-	-	-	-
1099	1034	L0157a		483.0	680.4	291	1.0	830.0	847.7	17.7	2.1	-	-	-	-
1100	1035	L0158a		485.1	680.4	298	1.0	832.0	838.8	6.8	0.8	-	-	-	-
1101	1036	L0159a		-	-	288	1.0	830.3	846.9	16.6	2.0	-	-	-	-
1102	1037	L0160a		487.0	680.4	293	1.0	833.2	838.7	5.5	0.7	-	-	-	-

Table S.14 – Comparison of experimental and simulated properties using WH combination rules (continued).

n_{sim}	n_{iso}	Code	Outlier	T_m [K]	T_b [K]	T [K]	P [bar]	$\rho_{\text{liq}}[\text{kg}\cdot\text{m}^{-3}]$ exp	sim	dev	err [%]	$\Delta H_{\text{vap}}[\text{kJ}\cdot\text{mol}^{-1}]$ exp	sim	dev	err [%]
1103	1038	L0161a		482.0	680.4	298	1.0	820.0	830.8	10.8	1.3	-	-	-	-
1104	1039	L0162a		482.0	680.4	293	1.0	821.5	835.1	13.6	1.7	-	-	-	-
1105	1040	L0163a		-	-	289	1.0	836.0	854.6	18.6	2.2	-	-	-	-
1106	1041	L0164a		491.1	680.4	293	1.0	832.2	849.7	17.5	2.1	-	-	-	-
1107	1042	L0165a		487.0	680.4	297	1.0	831.0	847.0	16.0	1.9	-	-	-	-
1108	1043	L0166a		489.1	680.4	300	1.0	826.0	843.9	17.9	2.2	-	-	-	-
1109	1044	L0167a		487.0	680.4	296	1.0	837.0	847.9	10.9	1.3	-	-	-	-
1110	1045	L0168a		487.0	680.4	298	1.0	828.2	846.1	17.9	2.2	-	-	-	-
1111	1046	L0169a		495.1	680.4	288	1.0	833.6	852.9	19.2	2.3	-	-	-	-
1112	1047	L0170a		474.1	680.4	298	1.0	820.6	836.9	16.3	2.0	-	-	-	-
1113	1048	L0171a		483.1	680.4	293	1.0	825.0	841.2	16.2	2.0	-	-	-	-
1114	1049	L0172a		483.0	680.4	298	1.0	822.9	839.1	16.2	2.0	-	-	-	-
1115	1050	L0201a		-	-	288	1.0	910.2	877.3	-32.9	-3.6	-	-	-	-
1116	1051	L0202a		503.1	855.5	298	1.0	943.5	932.8	-10.7	-1.1	-	-	-	-
1117	1052	L0203a		-	-	285	1.0	911.8	887.2	-24.6	-2.7	-	-	-	-
1118	1053	L0204a		-	-	293	1.0	916.5	901.3	-15.2	-1.7	-	-	-	-
1119	1054	L0205a		538.1	855.5	293	1.0	926.0	920.7	-5.3	-0.6	-	-	-	-
1120	1055	L0206a		-	-	293	1.0	948.1	926.4	-21.7	-2.3	-	-	-	-
1121	1056	L0207a		-	-	293	1.0	936.2	939.6	3.4	0.4	-	-	-	-
1122	1057	L0208a		-	-	301	1.0	915.5	903.1	-12.4	-1.4	-	-	-	-
1123	1058	L0209a		-	-	298	1.0	930.7	934.6	3.9	0.4	-	-	-	-
1124	1059	L0210a		572.1	855.5	353	1.0	883.0	897.7	14.7	1.7	-	-	-	-
1125	1060	D1201a		373.7	588.0	298	1.0	1213.6	1279.5	65.9	5.4	46.3	45.9	-0.4	-0.8
1126	1061	D2201a		391.1	592.0	298	1.01	1043.5	1085.0	41.5	4.0	50.3	51.6	1.3	2.7
1127	1062	D3201a		414.3	600.8	298	1.01	988.1	996.0	7.9	0.8	54.9	55.5	0.6	1.2
1128	1063	D4201a		427.6	-	298	1.0	943.9	945.0	1.1	0.1	56.3	58.4	2.1	3.8
1129	1064	D4202a		436.9	615.7	298	1.0	952.9	966.4	13.5	1.4	58.2	60.4	2.2	3.9
1130	1065	D5201a		436.9	631.0	310	1.0	907.5	919.1	11.6	1.3	-	-	-	-
1131	1065	D5201b		436.9	631.0	320	1.0	-	-	-	-	57.6	58.4	0.8	1.4
1132	1066	D5202a		450.1	631.0	298	1.0	934.7	939.0	4.3	0.5	-	-	-	-
1133	1067	D5203a		448.2	629.1	298	1.0	924.1	936.2	12.2	1.3	61.2	62.9	1.7	2.8
1134	1068	D5204a		458.6	639.2	298	1.0	934.4	954.4	20.0	2.1	63.0	65.1	2.1	3.3
1135	1069	D6201a		459.1	654.6	293	1.0	927.5	941.6	14.2	1.5	-	-	-	-
1136	1069	D6201b		459.1	654.6	370	1.0	-	-	-	-	59.4	58.6	-0.8	-1.3
1137	1070	D6202a		457.1	654.6	298	1.0	908.0	926.0	18.0	2.0	64.0	64.3	0.3	0.5
1138	1071	D6203a		464.9	654.6	293	1.0	927.5	934.6	7.2	0.8	-	-	-	-
1139	1072	D6204a		466.1	654.6	298	1.0	923.5	931.5	8.0	0.9	-	-	-	-
1140	1073	D6205a		-	654.6	298	1.0	918.2	930.5	12.3	1.3	-	-	-	-
1141	1074	D6206a		-	654.6	298	1.0	923.0	936.6	13.6	1.5	-	-	-	-
1142	1075	D6207a		473.6	654.6	298	1.0	917.0	935.2	18.2	2.0	-	-	-	-
1143	1076	D6208a		478.9	660.2	298	1.0	922.9	944.3	21.4	2.3	69.2	69.6	0.4	0.6
1144	1077	D7201a		-	676.3	293	1.0	885.6	933.9	48.3	5.5	-	-	-	-
1145	1078	D7202a		-	-	298	1.0	934.8	937.3	2.5	0.3	-	-	-	-
1146	1079	D7203a		-	-	298	1.0	910.0	918.3	8.3	0.9	-	-	-	-
1147	1080	D7204a		-	-	298	1.0	923.9	932.5	8.6	0.9	-	-	-	-
1148	1081	D7205a		-	676.3	298	1.0	909.8	925.1	15.3	1.7	-	-	-	-
1149	1082	D7206a		482.6	-	298	1.0	909.0	926.7	17.7	1.9	-	-	-	-
1150	1083	D7207a		486.1	676.3	293	1.0	918.7	932.9	14.2	1.5	-	-	-	-

Table S.14 – Comparison of experimental and simulated properties using WH combination rules (continued).

n_{sim}	n_{iso}	Code	Outlier	T_m [K]	T_b [K]	T [K]	P [bar]	ρ_{liq}[kg·m^{-3}] exp	sim	dev	err [%]	ΔH_{vap}[kJ·mol^{-1}] exp	sim	dev	err [%]
1151	1084	D7208a		489.1	676.3	293	1.0	916.3	931.9	15.6	1.7	-	-	-	-
1152	1085	D7209a		496.1	677.3	298	1.0	913.5	937.8	24.3	2.7	72.9	74.2	1.3	1.8
1153	1086	D8201a		-	-	298	1.0	919.9	937.7	17.8	1.9	-	-	-	-
1154	1087	D8202a		-	-	293	1.0	929.0	934.0	5.0	0.5	-	-	-	-
1155	1088	D8203a		-	-	298	1.0	900.9	931.0	30.1	3.3	-	-	-	-
1156	1089	D8204a		-	-	298	1.0	902.0	915.9	13.9	1.5	-	-	-	-
1157	1090	D8205a		494.1	696.5	298	1.0	904.0	920.1	16.1	1.8	74.8	75.3	0.5	0.7
1158	1091	D8206a		501.1	673.2	298	1.0	903.0	922.4	19.3	2.1	75.6	75.3	-0.3	-0.4
1159	1092	D8207a		-	-	293	1.0	910.5	933.4	22.9	2.5	-	-	-	-
1160	1093	D8208a		513.0	694.3	298	1.01	906.0	932.8	26.8	3.0	81.2	78.8	-2.4	-3.0
1161	1094	D9201a		-	-	298	1.0	910.2	926.8	16.6	1.8	-	-	-	-
1162	1095	D9202a		456.9	715.3	298	1.0	893.5	919.2	25.7	2.9	-	-	-	-
1163	1096	D9203a		456.9	715.3	277	1.0	909.7	937.1	27.4	3.0	-	-	-	-
1164	1097	D9204a		456.9	715.3	296	1.0	899.0	923.5	24.5	2.7	-	-	-	-
1165	1098	D9205a		528.8	710.7	298	1.0	901.6	929.8	28.2	3.1	-	-	-	-
1166	1098	D9205b		528.8	710.7	304	1.0	-	-	-	-	85.3	82.9	-2.4	-2.8
1167	1099	D0201a		473.7	733.0	293	1.0	902.2	924.1	21.9	2.4	-	-	-	-
1168	1100	D0202a		-	-	298	1.0	912.9	925.8	12.9	1.4	-	-	-	-
1169	1101	D0203a		-	-	291	1.0	897.0	920.5	23.5	2.6	-	-	-	-
1170	1102	D0204a		-	-	292	1.0	897.8	920.7	22.9	2.6	-	-	-	-
1171	1103	D0205a		-	733.0	293	1.0	848.1	920.8	72.7	8.6	-	-	-	-
1172	1104	D0206a		-	733.0	298	1.0	893.0	917.4	24.4	2.7	-	-	-	-
1173	1105	D0207a		473.7	733.0	293	1.0	898.3	924.2	25.9	2.9	-	-	-	-
1174	1106	D0208a		-	733.0	293	1.0	908.6	931.2	22.6	2.5	-	-	-	-
1175	1107	D0209a		543.1	722.1	313	1.0	881.7	916.4	34.7	3.9	88.6	86.4	-2.2	-2.5
1176	1108	N1101a		266.8	430.1	267	1.0	694.2	684.0	-10.2	-1.5	26.1	24.9	-1.2	-4.5
1177	1108	N1101b		266.8	430.1	298	3.52	655.0	605.3	-49.7	-7.6	-	-	-	-
1178	1109	N2101a		289.7	456.1	288	1.0	688.6	708.2	19.7	2.9	27.4	28.8	1.4	5.2
1179	1110	N2102a		280.0	437.2	283	1.0	667.9	733.7	65.8	9.9	27.2	32.4	5.2	19.2
1180	1110	N2102b		280.0	437.2	298	1.96	650.0	692.6	42.6	6.6	-	-	-	-
1181	1111	N2201a	vap	390.4	593.0	298	0.02	892.8	-	-	-	45.0	-	-	-
1182	1112	N3101a		305.6	471.9	298	1.01	683.9	691.4	7.4	1.1	28.4	28.8	0.4	1.3
1183	1113	N3102a		321.6	496.9	298	1.01	712.8	730.1	17.3	2.4	31.3	31.1	-0.2	-0.7
1184	1114	N3103a		276.0	433.2	273	1.0	655.7	675.9	20.2	3.1	22.9	23.9	1.0	4.3
1185	1114	N3103b		276.0	433.2	298	2.33	628.9	648.2	19.3	3.1	-	-	-	-
1186	1115	N3201a		388.1	-	298	1.0	841.0	904.2	63.2	7.5	-	-	-	-
1187	1116	N3202a	vap	392.4	585.0	298	1.0	0.0	-	-	-	44.2	-	-	-
1188	1117	N3203a		412.9	608.0	298	1.0	884.0	951.3	67.3	7.6	50.2	45.6	-4.6	-9.2
1189	1118	N4101a		317.6	483.9	298	1.0	690.1	691.8	1.8	0.3	29.6	28.0	-1.6	-5.5
1190	1119	N4102a		336.1	514.3	298	1.01	717.8	726.6	8.8	1.2	32.6	31.7	-0.9	-2.7
1191	1120	N4103a		340.9	522.4	298	1.0	729.1	734.0	4.9	0.7	33.8	32.3	-1.5	-4.4
1192	1121	N4104a		350.6	531.9	298	1.0	736.8	761.2	24.3	3.3	35.6	35.9	0.3	0.8
1193	1122	N4105a		323.6	482.4	288	1.0	740.0	716.6	-23.4	-3.2	30.7	30.7	0.0	0.0
1194	1123	N4106a		328.6	496.6	298	1.0	701.2	724.2	23.0	3.3	31.2	31.9	0.7	2.3
1195	1124	N4107a		336.1	482.4	298	0.2	711.9	721.4	9.5	1.3	-	-	-	-
1196	1125	N4108a		310.1	500.0	298	1.0	669.4	691.7	22.3	3.3	-	-	-	-
1197	1126	N4201a		378.6	-	298	1.0	803.0	849.5	46.5	5.8	-	-	-	-
1198	1127	N4202a		393.1	-	288	1.0	828.0	889.8	61.8	7.5	-	-	-	-

Table S.14 – Comparison of experimental and simulated properties using WH combination rules (continued).

n_{sim}	n_{iso}	Code	Outlier	T_m [K]	T_b [K]	T [K]	P [bar]	ρ_{liq}[kg·m^{-3}] exp	sim	dev	err [%]	ΔH_{vap}[kJ·mol^{-1}] exp	sim	dev	err [%]
1199	1128	N4203a	vap	396.1	-	298	1.0	841.0	-	-	-	45.8	-	-	-
1200	1129	N4204a		402.1	-	298	1.0	837.0	880.0	43.0	5.1	-	-	-	-
1201	1130	N4205a	×	413.1	-	298	1.0	-	-	-	-	53.1	41.8	-11.3	-21.2
1202	1131	N4206a		-	-	298	1.0	-	-	-	-	46.9	46.7	-0.2	-0.4
1203	1132	N4207a		431.6	-	298	1.0	877.0	955.5	78.5	8.9	55.2	54.6	-0.6	-1.0
1204	1133	N5101a		350.1	557.7	298	0.11	727.6	731.2	3.6	0.5	-	-	-	-
1205	1134	N5102a		350.1	557.7	298	0.1	728.1	742.1	14.0	1.9	-	-	-	-
1206	1135	N5103a		357.1	557.7	298	0.08	753.0	742.8	-10.2	-1.4	-	-	-	-
1207	1136	N5104a		362.1	557.7	291	1.0	711.3	757.1	45.8	6.4	-	-	-	-
1208	1137	N5105a		368.6	557.7	298	1.0	750.5	768.1	17.6	2.3	-	-	-	-
1209	1138	N5106a		370.1	557.7	298	1.0	744.3	772.8	28.5	3.8	-	-	-	-
1210	1139	N5107a		377.6	557.7	298	0.04	751.0	782.1	31.1	4.1	40.1	40.4	0.3	0.8
1211	1140	N5108a		350.1	517.7	298	1.0	727.0	770.5	43.5	6.0	32.3	33.9	1.6	5.0
1212	1141	N5109a		351.6	517.7	298	0.1	720.0	728.5	8.6	1.2	-	-	-	-
1213	1142	N5110a		346.1	517.7	298	0.12	730.0	718.5	-11.5	-1.6	-	-	-	-
1214	1143	N5111a		349.1	517.7	298	1.0	-	-	-	-	33.1	32.1	-1.0	-2.9
1215	1144	N5112a		353.4	517.7	298	0.11	726.7	736.1	9.5	1.3	-	-	-	-
1216	1145	N5113a		364.2	517.7	298	0.05	728.1	747.7	19.6	2.7	-	-	-	-
1217	1146	N5114a		339.2	517.7	298	1.0	710.6	716.9	6.3	0.9	31.9	31.0	-0.9	-3.0
1218	1147	N5115a		339.1	517.7	298	1.0	701.5	722.6	21.0	3.0	31.8	31.8	0.0	0.1
1219	1148	N5116a		339.1	517.7	298	0.21	695.6	719.8	24.2	3.5	-	-	-	-
1220	1149	N5201a		356.1	-	291	1.0	749.1	783.0	33.9	4.5	-	-	-	-
1221	1149	N5201b		356.1	-	298	1.0	-	-	-	-	33.1	36.1	3.0	9.2
1222	1150	N5202a		406.6	-	293	1.0	827.2	857.3	30.1	3.6	42.2	46.2	4.0	9.4
1223	1151	N5203a		437.1	-	298	1.0	-	-	-	-	54.9	47.6	-7.3	-13.2
1224	1152	N5204a		452.1	-	298	1.0	873.0	944.7	71.7	8.2	58.7	59.2	0.5	0.8
1225	1153	N6101a		375.1	589.1	298	0.04	760.1	763.4	3.3	0.4	-	-	-	-
1226	1154	N6102a		381.6	589.1	298	0.02	745.7	761.6	15.9	2.1	-	-	-	-
1227	1155	N6103a		404.6	589.1	298	1.0	763.0	798.1	35.1	4.6	45.0	45.0	0.0	0.0
1228	1156	N6104a		351.1	549.1	298	1.0	716.1	776.4	60.3	8.4	-	-	-	-
1229	1157	N6105a		357.1	523.1	298	1.0	712.3	726.2	13.9	2.0	34.5	33.7	-0.8	-2.3
1230	1158	N6106a		371.1	549.1	298	0.04	729.8	739.7	10.0	1.4	-	-	-	-
1231	1159	N6107a		371.4	549.1	298	0.04	723.2	736.4	13.3	1.8	37.3	35.4	-1.9	-5.1
1232	1160	N6108a		-	-	295	1.0	739.0	763.3	24.3	3.3	-	-	-	-
1233	1161	N6109a		382.0	550.0	298	1.0	734.9	748.3	13.4	1.8	40.0	37.3	-2.7	-6.7
1234	1162	N6110a		381.1	549.1	298	0.02	735.4	755.4	20.0	2.7	40.2	38.9	-1.3	-3.3
1235	1163	N6111a		390.6	549.1	298	0.02	743.0	765.8	22.8	3.1	-	-	-	-
1236	1164	N6112a		363.1	549.1	298	1.0	737.6	747.4	9.8	1.3	34.8	34.8	0.0	0.0
1237	1165	N6113a		367.1	549.1	298	0.05	733.9	745.8	11.8	1.6	-	-	-	-
1238	1166	N6114a		364.6	549.1	298	0.06	721.5	739.1	17.6	2.4	-	-	-	-
1239	1167	N6115a		354.1	549.1	298	0.1	720.0	728.7	8.7	1.2	-	-	-	-
1240	1168	N6116a		361.9	535.1	298	1.01	723.0	743.9	20.9	2.9	34.9	35.9	1.0	2.9
1241	1169	N6117a		364.6	549.1	298	0.06	718.0	742.6	24.6	3.4	-	-	-	-
1242	1170	N6118a		367.1	549.1	298	0.06	716.0	744.7	28.7	4.0	-	-	-	-
1243	1171	N6201a		394.1	-	298	1.0	770.0	805.2	35.2	4.6	41.4	42.8	1.4	3.3
1244	1172	N6202a		407.6	-	298	1.0	738.0	817.5	79.5	10.8	-	-	-	-
1245	1173	N6203a		417.1	-	293	1.0	828.0	861.5	33.5	4.0	-	-	-	-
1246	1174	N6204a		419.1	-	293	1.0	828.0	851.0	23.0	2.8	-	-	-	-

Table S.14 – Comparison of experimental and simulated properties using WH combination rules (continued).

n_{sim}	n_{iso}	Code	Outlier	T_m [K]	T_b [K]	T [K]	P [bar]	ρ_{liq}[kg·m^{-3}] exp	sim	dev	err [%]	ΔH_{vap}[kJ·mol^{-1}] exp	sim	dev	err [%]
1247	1175	N6205a		466.1	-	298	1.0	-	-	-	-	60.9	60.1	-0.8	-1.3
1248	1176	N6206a		475.0	-	298	1.0	-	-	-	-	63.1	63.0	-0.1	-0.2
1249	1177	N7101a		-	-	298	1.0	761.5	773.6	12.1	1.6	-	-	-	-
1250	1178	N7102a		395.1	617.7	298	0.01	768.2	759.0	-9.2	-1.2	-	-	-	-
1251	1179	N7103a		-	-	293	1.0	788.2	773.0	-15.2	-1.9	-	-	-	-
1252	1180	N7104a		405.6	617.7	307	0.01	755.5	774.8	19.3	2.6	-	-	-	-
1253	1181	N7105a		412.6	617.7	312	0.01	752.7	766.3	13.6	1.8	-	-	-	-
1254	1182	N7106a		422.1	617.7	320	0.01	756.1	784.0	27.9	3.7	-	-	-	-
1255	1183	N7107a		415.1	617.7	293	1.0	762.9	791.4	28.5	3.7	-	-	-	-
1256	1184	N7108a		430.1	617.7	298	1.0	771.3	809.9	38.6	5.0	49.9	49.4	-0.5	-0.9
1257	1185	N7109a		-	-	298	1.0	-	-	-	-	42.1	39.8	-2.3	-5.4
1258	1186	N7110a		415.1	577.7	293	1.0	778.7	785.8	7.1	0.9	-	-	-	-
1259	1187	N7111a		383.1	577.7	298	0.02	744.5	763.6	19.0	2.6	-	-	-	-
1260	1188	N7112a		385.1	577.7	298	0.02	749.5	752.4	2.9	0.4	-	-	-	-
1261	1189	N7113a		380.1	577.7	298	0.03	729.5	747.8	18.3	2.5	-	-	-	-
1262	1190	N7114a		385.1	577.7	298	0.02	738.0	759.4	21.4	2.9	-	-	-	-
1263	1191	N7201a		417.1	-	298	1.0	-	-	-	-	45.3	46.9	1.6	3.6
1264	1192	N7202a		441.6	-	293	1.0	-	-	-	-	52.4	53.1	0.7	1.3
1265	1193	N7203a		497.1	-	298	1.0	-	-	-	-	67.1	67.1	-0.0	-0.1
1266	1194	N8101a		438.1	643.9	293	1.0	772.0	803.4	31.4	4.1	-	-	-	-
1267	1195	N8102a		452.8	643.9	298	1.01	780.0	819.8	39.8	5.1	55.1	54.0	-1.1	-2.0
1268	1196	N8103a		407.1	603.9	298	1.0	749.0	758.8	9.8	1.3	-	-	-	-
1269	1196	N8103b		407.1	603.9	308	0.01	-	-	-	-	46.3	40.6	-5.7	-12.3
1270	1197	N8104a		412.2	603.9	298	1.0	740.7	745.3	4.5	0.6	-	-	-	-
1271	1198	N8105a		432.0	602.3	298	1.0	755.7	779.2	23.5	3.1	49.4	46.6	-2.8	-5.6
1272	1199	N8106a		438.1	603.9	273	1.0	771.2	817.4	46.2	6.0	-	-	-	-
1273	1200	N8107a		399.6	603.9	313	1.0	749.0	745.9	-3.1	-0.4	-	-	-	-
1274	1201	N8108a		409.1	603.9	293	1.0	742.0	778.4	36.4	4.9	-	-	-	-
1275	1202	N8109a		-	-	298	1.0	742.0	774.2	32.2	4.3	-	-	-	-
1276	1203	N8201a		441.1	-	288	1.0	794.2	829.7	35.5	4.5	-	-	-	-
1277	1204	N8202a		457.1	-	288	1.0	848.5	871.7	23.2	2.7	-	-	-	-
1278	1205	N9101a		-	-	293	1.0	771.9	795.5	23.6	3.1	-	-	-	-
1279	1206	N9102a		-	-	298	1.0	789.2	821.3	32.1	4.1	-	-	-	-
1280	1207	N9103a		475.4	668.2	293	1.0	788.6	832.0	43.4	5.5	-	-	-	-
1281	1208	N9104a		453.9	628.2	298	1.0	767.5	793.5	25.9	3.4	-	-	-	-
1282	1209	N9105a		459.9	628.2	293	1.0	782.4	806.4	24.0	3.1	-	-	-	-
1283	1210	N9106a		429.6	628.2	298	1.0	752.9	782.2	29.4	3.9	46.2	48.7	2.5	5.4
1284	1211	N9107a		429.1	628.2	293	1.0	766.3	790.0	23.7	3.1	-	-	-	-
1285	1212	N9108a		432.8	628.2	293	1.0	759.3	790.9	31.6	4.2	-	-	-	-
1286	1213	N9109a		445.1	628.2	293	1.0	758.0	791.8	33.8	4.5	-	-	-	-
1287	1214	N9201a		438.9	-	293	1.0	800.0	821.1	21.1	2.6	-	-	-	-
1288	1215	N9202a		474.1	-	293	1.0	814.0	850.8	36.8	4.5	-	-	-	-
1289	1216	N9203a		531.6	-	298	1.0	-	-	-	-	75.5	75.5	-0.0	-0.0
1290	1217	N0101a		493.6	690.9	298	1.0	789.0	833.5	44.5	5.6	64.9	62.9	-2.0	-3.1
1291	1218	N0102a		461.1	650.9	298	1.0	766.9	790.1	23.2	3.0	-	-	-	-
1292	1219	N0103a		476.1	650.9	298	1.0	773.2	798.2	24.9	3.2	61.2	55.4	-5.8	-9.5
1293	1220	N0104a		475.1	650.9	298	1.0	774.3	802.1	27.8	3.6	-	-	-	-
1294	1221	N0105a		480.1	650.9	292	1.0	784.0	815.2	31.2	4.0	-	-	-	-

Table S.14 – Comparison of experimental and simulated properties using WH combination rules (continued).

n_{sim}	n_{iso}	Code	Outlier	T_m [K]	T_b [K]	T [K]	P [bar]	ρ_{liq}[kg·m^{-3}] exp	sim	dev	err [%]	ΔH_{vap}[kJ·mol^{-1}] exp	sim	dev	err [%]
1295	1222	N0106a		-	-	293	1.0	768.6	797.4	28.8	3.7	-	-	-	-
1296	1223	N0107a		464.1	650.9	293	1.0	768.7	801.4	32.7	4.3	-	-	-	-
1297	1224	N0201a		485.0	-	345	1.0	-	-	-	-	73.6	69.3	-4.3	-5.8
1298	1225	M2201a		494.3	761.0	354	0.004	1002.0	981.9	-20.1	-2.0	-	-	-	-
1299	1226	M3201a		478.1	718.0	308	1.01	949.7	929.4	-20.4	-2.1	-	-	-	-
1300	1227	M3202a		486.1	-	356	1.01	963.2	932.8	-30.4	-3.2	-	-	-	-
1301	1227	M3202b		486.1	-	390	1.0	-	-	-	-	63.9	57.2	-6.7	-10.5
1302	1228	M4201a		439.2	658.0	298	1.01	936.7	904.9	-31.8	-3.4	50.7	50.1	-0.6	-1.1
1303	1229	M4202a		421.1	-	298	1.0	930.5	916.7	-13.8	-1.5	66.6	65.9	-0.7	-1.0
1304	1230	M4203a		478.1	-	277	1.0	942.0	947.9	5.9	0.6	-	-	-	-
1305	1230	M4203b		478.1	-	298	1.0	-	-	-	-	64.9	67.5	2.6	4.0
1306	1231	M4204a		489.1	-	393	1.0	885.0	877.9	-7.1	-0.8	-	-	-	-
1307	1232	M5201a		444.6	-	298	1.01	920.3	896.5	-23.8	-2.6	-	-	-	-
1308	1233	M5202a		-	-	298	1.0	-	-	-	-	67.1	68.0	0.9	1.3
1309	1234	M5203a		-	-	298	1.0	-	-	-	-	66.4	63.0	-3.4	-5.2
1310	1235	M5204a		-	-	298	1.0	-	-	-	-	69.8	70.7	0.9	1.3
1311	1236	M5205a	×	427.0	-	427	1.0	-	-	-	-	36.9	53.6	16.7	45.2
1312	1237	M6201a		459.1	-	298	1.0	906.4	892.7	-13.7	-1.5	-	-	-	-
1313	1238	M6202a		458.6	700.0	290	1.0	913.0	898.4	-14.6	-1.6	53.7	55.0	1.3	2.4
1314	1239	M6203a		502.1	700.0	298	1.0	896.0	911.8	15.8	1.8	76.1	70.4	-5.7	-7.5
1315	1240	M7201a		464.1	-	293	1.0	897.2	890.5	-6.7	-0.7	-	-	-	-
1316	1241	M7202a		458.8	-	298	1.0	896.2	892.4	-3.8	-0.4	-	-	-	-
1317	1242	M8201a		482.6	700.0	290	1.0	899.2	892.9	-6.3	-0.7	-	-	-	-
1318	1243	M9201a		484.1	-	293	1.0	876.4	881.6	5.2	0.6	-	-	-	-
1319	1244	S1201a		341.2	557.0	298	1.01	1924.9	1932.8	8.0	0.4	-	-	-	-
1320	1245	S1202a		264.1	411.5	260	1.0	1282.1	1211.5	-70.6	-5.5	21.9	24.9	3.0	13.7
1321	1246	S1301a		257.6	412.0	298	1.0	1775.5	1786.3	10.8	0.6	-	-	-	-
1322	1246	S1301b	×	257.6	412.0	244	1.0	-	-	-	-	24.0	32.7	8.7	36.2
1323	1247	S1302a	vap	232.3	369.3	233	1.0	1408.0	-	-	-	20.2	-	-	-
1324	1247	S1302b		232.3	369.3	298	10.77	1193.0	1212.9	19.9	1.7	-	-	-	-
1325	1248	S1303a		282.1	451.6	298	1.83	1367.0	1400.0	33.1	2.4	-	-	-	-
1326	1248	S1303b		282.1	451.6	267	1.0	-	-	-	-	26.1	31.8	5.6	21.6
1327	1249	S1304a	×	373.1	-	295	1.0	3196.9	3024.0	-172.9	-5.4	-	-	-	-
1328	1250	S1305a		363.1	-	298	1.0	1983.5	2028.9	45.4	2.3	-	-	-	-
1329	1251	S1401a	×	378.1	606.0	298	1.01	2002.1	2058.3	56.1	2.8	36.1	44.4	8.3	22.9
1330	1252	S1402a	×	215.3	340.1	215	1.0	1989.9	1749.4	-240.5	-12.1	17.5	27.3	9.8	55.7
1331	1252	S1402b		215.3	340.1	298	16.64	1536.1	1532.7	-3.4	-0.2	-	-	-	-
1332	1253	S1403a	×	295.9	478.0	298	1.0	2251.2	2252.5	1.3	0.1	25.0	37.9	12.9	51.7
1333	1254	S1404a	×	191.7	301.8	190	1.0	1527.8	1667.0	139.2	9.1	15.4	23.5	8.1	52.6
1334	1254	S1404b	×	191.7	301.8	298	36.66	840.9	1139.8	298.8	35.5	-	-	-	-
1335	1255	S1405a		243.4	384.9	243	1.0	1488.0	1441.1	-46.9	-3.2	20.1	26.1	5.9	29.6
1336	1255	S1405b		243.4	384.9	298	6.74	1307.0	1320.0	12.9	1.0	-	-	-	-
1337	1256	S1406a		297.0	471.2	298	1.06	1477.0	1500.7	23.7	1.6	24.9	31.3	6.5	26.1
1338	1257	S1407a	×	250.7	-	298	20.0	2046.8	1909.5	-137.4	-6.7	-	-	-	-
1339	1258	S1408a	×	269.1	426.1	269	1.0	1899.6	1871.5	-28.2	-1.5	23.1	32.6	9.5	41.1
1340	1258	S1408b		269.1	426.1	298	2.6	1810.0	1804.0	-6.0	-0.3	-	-	-	-
1341	1259	S2201a		356.1	-	283	1.0	1667.0	1660.6	-6.4	-0.4	-	-	-	-
1342	1260	S2202a		380.1	-	298	1.01	1727.0	1724.2	-2.8	-0.2	-	-	-	-

Table S.14 – Comparison of experimental and simulated properties using WH combination rules (continued).

n_{sim}	n_{iso}	Code	Outlier	T_m	T_b	T	P	$\rho_{\text{liq}}[\text{kg}\cdot\text{m}^{-3}]$				$\Delta H_{\text{vap}}[\text{kJ}\cdot\text{mol}^{-1}]$			
				[K]	[K]	[K]	[bar]	exp	sim	dev	err [%]	exp	sim	dev	err [%]
1343	1260	S2202b		380.1	-	308	1.0	-	-	-	-	37.6	35.8	-1.8	-4.8
1344	1261	S2203a	×	344.6	-	298	1.0	1704.4	1598.4	-106.0	-6.2	-	-	-	-
1345	1262	S2204a	×	326.1	497.1	298	0.33	1167.5	1073.7	-93.8	-8.0	-	-	-	-
1346	1263	S2205a		413.1	-	288	1.0	2133.6	2127.2	-6.4	-0.3	-	-	-	-
1347	1264	S2206a		401.8	585.0	298	1.01	1200.9	1139.8	-61.1	-5.1	48.3	48.7	0.4	0.8
1348	1265	S2207a		423.1	-	293	1.0	1762.9	1708.1	-54.8	-3.1	54.1	57.0	2.9	5.3
1349	1266	S2208a	×	376.6	-	293	1.0	1104.0	952.6	-151.4	-13.7	-	-	-	-
1350	1266	S2208b		376.6	-	288	1.0	-	-	-	-	44.1	39.6	-4.5	-10.1
1351	1267	S2209a		449.1	-	293	1.0	2196.7	2144.1	-52.6	-2.4	-	-	-	-
1352	1267	S2209b		449.1	-	288	1.0	-	-	-	-	57.0	62.1	5.1	9.0
1353	1268	S2210a	×	-	-	373	1.0	1366.0	928.2	-437.8	-32.0	-	-	-	-
1354	1269	S2211a		444.1	678.2	298	1.01	1012.1	1003.7	-8.4	-0.8	59.6	60.5	0.9	1.5
1355	1270	S2301a	×	263.1	410.3	265	1.07	1188.8	1044.8	-144.0	-12.1	22.7	21.5	-1.2	-5.2
1356	1270	S2301b	×	263.1	410.3	298	3.61	1107.7	967.6	-140.1	-12.7	19.7	20.0	0.2	1.2
1357	1271	S2302a		304.9	478.9	298	1.0	1233.7	1168.6	-65.1	-5.3	26.0	26.9	0.9	3.5
1358	1272	S2303a		346.9	523.6	298	1.0	1369.2	1320.1	-49.1	-3.6	-	-	-	-
1359	1273	S2304a	vap	362.0	555.0	298	0.07	1433.0	-	-	-	0.0	-	-	-
1360	1274	S2305a		462.5	686.0	352	0.01	1354.2	1352.5	-1.7	-0.1	-	-	-	-
1361	1275	S2306a		481.1	-	323	1.0	1933.5	1915.7	-17.8	-0.9	-	-	-	-
1362	1276	S2307a	×	441.1	-	309	1.0	1369.3	1251.7	-117.6	-8.6	-	-	-	-
1363	1277	S2308a		419.1	-	298	1.0	1404.0	1382.0	-22.0	-1.6	-	-	-	-
1364	1278	S2309a	vap	-	-	571	1.0	1607.0	-	-	-	0.0	-	-	-
1365	1279	S2401a	×	365.6	-	293	1.0	2223.8	2115.7	-108.1	-4.9	-	-	-	-
1366	1280	S2402a	×	319.8	479.1	298	1.0	1406.6	1304.9	-101.7	-7.2	-	-	-	-
1367	1281	S2403a	×	-	-	283	1.0	1874.0	1713.1	-160.9	-8.6	-	-	-	-
1368	1282	S2404a	×	279.2	426.2	285	1.0	1353.9	1143.1	-210.8	-15.6	-	-	-	-
1369	1283	S2405a		361.1	535.0	298	1.0	1482.6	1449.8	-32.8	-2.2	-	-	-	-
1370	1284	S2406a		375.1	555.6	298	1.0	1529.8	1516.0	-13.8	-0.9	-	-	-	-
1371	1285	S2407a	×	327.6	-	298	1.0	2130.0	1853.9	-276.1	-13.0	-	-	-	-
1372	1286	S2408a	vap	370.9	565.0	298	0.06	1499.1	-	-	-	0.0	-	-	-
1373	1287	S2409a	vap	447.1	-	298	1.0	2664.9	-	-	-	0.0	-	-	-
1374	1288	S2410a		467.1	686.0	356	0.01	1473.5	1540.0	66.5	4.5	-	-	-	-
1375	1289	S2411a	×	406.1	-	298	1.0	1526.0	1357.3	-168.7	-11.1	-	-	-	-
1376	1290	S2412a	×	347.1	-	298	1.01	1382.4	1058.4	-324.0	-23.4	-	-	-	-
1377	1290	S2412b		347.1	-	289	1.0	-	-	-	-	45.9	39.3	-6.6	-14.3
1378	1291	S2501a		469.6	688.0	337	1.0	1612.6	1663.8	51.2	3.2	-	-	-	-
1379	1292	S2502a	×	344.9	491.2	298	1.0	1535.1	1316.5	-218.6	-14.2	35.9	45.7	9.8	27.3
1380	1293	S3201a		368.1	-	293	1.0	1495.0	1460.7	-34.3	-2.3	-	-	-	-
1381	1294	S3202a		390.1	-	293	1.0	1537.0	1547.9	10.9	0.7	-	-	-	-
1382	1295	S3203a		391.1	-	293	1.0	1531.0	1554.6	23.6	1.5	-	-	-	-
1383	1296	S3204a		416.4	-	293	1.0	1596.9	1595.5	-1.4	-0.1	-	-	-	-
1384	1297	S3205a	×	-	515.4	293	1.0	998.2	899.0	-99.2	-9.9	-	-	-	-
1385	1298	S3206a		-	-	293	1.0	1086.0	1006.6	-79.4	-7.3	-	-	-	-
1386	1299	S3207a		444.1	-	293	1.0	1904.0	1935.9	31.9	1.7	-	-	-	-
1387	1300	S3208a	vap	359.1	-	288	1.0	1182.0	-	-	-	0.0	-	-	-
1388	1301	S3209a		392.1	-	293	1.0	1150.0	1115.2	-34.8	-3.0	-	-	-	-
1389	1302	S3210a		411.1	-	296	1.0	1634.0	1568.9	-65.1	-4.0	-	-	-	-
1390	1303	S3211a		350.1	-	293	1.0	1028.8	970.2	-58.6	-5.7	-	-	-	-

Table S.14 – Comparison of experimental and simulated properties using WH combination rules (continued).

n_{sim}	n_{iso}	Code	Outlier	T_m [K]	T_b [K]	T [K]	P [bar]	ρ_{liq}[kg·m^{-3}] exp	sim	dev	err [%]	ΔH_{vap}[kJ·mol^{-1}] exp	sim	dev	err [%]
1391	1304	S3212a	×	-	-	288	1.0	2170.0	1953.5	-216.5	-10.0	-	-	-	-
1392	1305	S3213a		400.1	-	293	1.0	1113.0	1069.2	-43.8	-3.9	-	-	-	-
1393	1306	S3214a		406.6	-	293	1.0	1103.0	1063.7	-39.3	-3.6	-	-	-	-
1394	1307	S3215a		438.1	-	293	1.0	1130.9	1102.6	-28.3	-2.5	-	-	-	-
1395	1308	S3216a		419.6	-	303	1.0	1558.5	1517.2	-41.3	-2.6	-	-	-	-
1396	1309	S3217a		395.3	-	293	1.0	1537.4	1564.7	27.3	1.8	-	-	-	-
1397	1310	S3218a		499.1	-	499	1.0	-	-	-	-	43.8	45.5	1.7	3.9
1398	1311	S3219a		365.6	-	293	1.0	1034.5	987.2	-47.3	-4.6	-	-	-	-
1399	1312	S3220a		383.1	-	293	1.0	1462.3	1419.4	-42.9	-2.9	-	-	-	-
1400	1313	S3221a	×	418.6	596.0	298	1.0	1074.3	974.3	-100.0	-9.3	44.2	56.5	12.3	27.8
1401	1314	S3222a		431.1	717.6	293	1.0	937.0	902.4	-34.6	-3.7	57.8	55.0	-2.8	-4.8
1402	1315	S3223a		432.6	717.6	298	1.01	956.5	920.8	-35.7	-3.7	-	-	-	-
1403	1316	S3224a		460.6	717.6	298	1.01	987.4	942.1	-45.3	-4.6	-	-	-	-
1404	1317	S3225a	×	365.1	-	298	1.0	1005.0	869.9	-135.1	-13.4	-	-	-	-
1405	1318	S3226a		397.6	564.0	298	1.01	960.2	881.9	-78.4	-8.2	45.2	45.6	0.4	0.9
1406	1319	S3301a		-	-	293	1.0	1708.4	1723.7	15.3	0.9	-	-	-	-
1407	1320	S3302a		469.1	-	287	1.0	2093.0	2097.9	4.9	0.2	-	-	-	-
1408	1321	S3303a		361.8	536.5	298	0.07	1255.2	1182.4	-72.9	-5.8	-	-	-	-
1409	1322	S3304a		458.1	-	293	1.0	1258.5	1265.8	7.3	0.6	-	-	-	-
1410	1323	S3305a		403.0	600.0	293	1.0	1236.0	1164.8	-71.2	-5.8	46.7	43.6	-3.1	-6.7
1411	1324	S3306a		405.1	-	293	1.0	1635.0	1573.4	-61.6	-3.8	-	-	-	-
1412	1325	S3307a		377.6	-	288	1.0	-	-	-	-	42.7	39.5	-3.2	-7.5
1413	1326	S3308a		393.1	-	291	1.0	1304.0	1326.9	22.9	1.8	-	-	-	-
1414	1327	S3309a		446.6	-	319	1.0	1382.6	1349.3	-33.3	-2.4	-	-	-	-
1415	1328	S3310a		449.1	633.0	298	0.87	1356.5	1319.2	-37.2	-2.7	-	-	-	-
1416	1328	S3310b		449.1	633.0	316	1.0	-	-	-	-	50.4	53.5	3.1	6.1
1417	1329	S3311a		457.1	647.0	293	1.0	1360.7	1321.4	-39.3	-2.9	-	-	-	-
1418	1330	S3312a		492.1	-	293	1.0	2136.4	2106.5	-29.9	-1.4	-	-	-	-
1419	1331	S3313a		492.1	-	293	1.0	2120.0	2100.5	-19.5	-0.9	-	-	-	-
1420	1332	S3314a	×	400.1	-	298	1.0	1240.0	1046.9	-193.1	-15.6	-	-	-	-
1421	1333	S3315a	×	489.1	-	293	1.0	1321.9	1238.8	-83.1	-6.3	-	-	-	-
1422	1334	S3316a	×	486.1	648.0	298	1.0	1315.9	1228.4	-87.4	-6.6	-	-	-	-
1423	1335	S3317a		-	-	303	1.0	-	-	-	-	31.8	30.8	-1.1	-3.3
1424	1336	S3318a	×	422.1	-	291	1.0	1167.7	1057.0	-110.7	-9.5	-	-	-	-
1425	1337	S3319a	×	478.3	691.0	293	1.0	1176.8	1092.9	-83.9	-7.1	-	-	-	-
1426	1338	S3401a		-	-	293	1.0	2077.2	2116.0	38.8	1.9	-	-	-	-
1427	1339	S3402a		460.6	-	285	1.0	1389.0	1447.2	58.2	4.2	-	-	-	-
1428	1340	S3403a		416.1	-	293	1.0	1377.4	1347.2	-30.2	-2.2	47.7	51.4	3.7	7.7
1429	1341	S3404a		358.5	-	288	1.0	-	-	-	-	41.9	39.2	-2.7	-6.5
1430	1342	S3405a		422.1	-	293	1.0	1435.0	1460.6	25.6	1.8	-	-	-	-
1431	1343	S3406a	×	295.1	-	298	1.0	1252.0	1055.1	-196.9	-15.7	-	-	-	-
1432	1344	S3407a		348.1	-	298	1.0	-	-	-	-	44.8	39.0	-5.8	-13.0
1433	1345	S3501a		426.9	-	293	1.0	1487.4	1455.9	-31.5	-2.1	48.3	52.4	4.1	8.4
1434	1346	S3502a	×	316.6	-	293	1.0	1280.0	1094.5	-185.5	-14.5	-	-	-	-
1435	1347	S3503a	×	382.6	-	302	1.01	1482.8	1165.8	-317.0	-21.4	53.6	38.9	-14.7	-27.4
1436	1348	S4201a		448.1	-	298	1.01	1485.1	1490.6	5.5	0.4	-	-	-	-
1437	1349	S4202a		363.1	-	288	1.0	1053.0	995.4	-57.6	-5.5	-	-	-	-
1438	1350	S4203a		394.6	-	281	1.0	1106.0	1081.8	-24.2	-2.2	-	-	-	-

Table S.14 – Comparison of experimental and simulated properties using WH combination rules (continued).

n_{sim}	n_{iso}	Code	Outlier	T_m [K]	T_b [K]	T [K]	P [bar]	ρ_{liq} [kg·m^{-3}] exp	sim	dev	err [%]	ΔH_{vap} [kJ·mol^{-1}] exp	sim	dev	err [%]
1439	1351	S4204a		388.1	-	298	1.0	1055.4	1033.7	-21.7	-2.1	-	-	-	-
1440	1352	S4205a		414.1	-	298	1.0	1068.0	1045.8	-22.2	-2.1	-	-	-	-
1441	1353	S4206a		388.3	-	293	1.0	1088.3	1067.2	-21.1	-1.9	-	-	-	-
1442	1354	S4207a		380.6	-	293	1.0	989.5	959.7	-29.8	-3.0	-	-	-	-
1443	1355	S4208a		400.6	-	273	1.0	1385.2	1354.6	-30.6	-2.2	-	-	-	-
1444	1356	S4209a	×	408.1	620.0	298	1.0	1096.9	919.8	-177.1	-16.1	-	-	-	-
1445	1357	S4210a	×	418.6	-	298	1.0	-	-	-	-	48.7	59.1	10.4	21.4
1446	1358	S4211a		433.1	-	293	1.0	1027.2	954.2	-73.0	-7.1	-	-	-	-
1447	1359	S4212a		455.1	-	293	1.0	1023.3	964.6	-58.7	-5.7	-	-	-	-
1448	1360	S4213a		407.1	-	298	1.01	883.8	867.3	-16.5	-1.9	46.7	54.2	7.5	16.1
1449	1361	S4214a	vap	438.6	-	298	1.01	932.6	-	-	-	0.0	-	-	-
1450	1361	S4214b	vap	438.6	-	308	1.0	0.0	-	-	-	63.3	-	-	-
1451	1362	S4215a		442.6	-	293	1.0	914.0	896.2	-17.8	-2.0	61.0	57.2	-3.8	-6.2
1452	1363	S4216a		478.1	-	285	1.0	967.0	988.3	21.3	2.2	-	-	-	-
1453	1364	S4217a		389.1	-	298	1.0	957.0	879.7	-77.3	-8.1	-	-	-	-
1454	1365	S4218a		403.1	588.8	293	1.0	938.0	865.4	-72.6	-7.7	-	-	-	-
1455	1366	S4219a		392.1	588.8	298	1.01	915.9	850.9	-65.0	-7.1	46.2	42.8	-3.4	-7.4
1456	1367	S4220a		408.1	588.8	298	1.0	925.4	867.4	-57.9	-6.3	48.2	50.6	2.4	4.9
1457	1368	S4301a		-	-	293	1.0	1598.5	1621.7	23.2	1.5	-	-	-	-
1458	1369	S4302a		406.8	-	293	1.0	1179.6	1208.8	29.2	2.5	-	-	-	-
1459	1370	S4303a		406.8	-	293	1.0	1223.6	1236.1	12.5	1.0	-	-	-	-
1460	1371	S4304a		472.1	-	333	1.0	1496.9	1502.2	5.3	0.4	-	-	-	-
1461	1372	S4305a		405.6	-	298	1.0	1075.0	1080.9	5.9	0.5	-	-	-	-
1462	1373	S4306a		417.4	618.0	293	1.0	1158.5	1103.6	-54.9	-4.7	49.5	46.1	-3.4	-6.9
1463	1374	S4307a		418.1	-	293	1.0	1178.0	1104.7	-73.3	-6.2	-	-	-	-
1464	1375	S4308a		441.6	-	293	1.0	1503.2	1456.5	-46.7	-3.1	-	-	-	-
1465	1376	S4309a		435.6	-	293	1.0	1514.0	1463.8	-50.2	-3.3	-	-	-	-
1466	1377	S4310a		-	-	291	1.0	1412.3	1470.6	58.3	4.1	-	-	-	-
1467	1378	S4311a	×	393.1	-	293	1.0	1091.2	998.5	-92.7	-8.5	-	-	-	-
1468	1378	S4311b		393.1	-	288	1.0	-	-	-	-	41.9	41.8	-0.1	-0.3
1469	1379	S4312a		452.1	-	286	1.0	1817.3	1760.4	-56.9	-3.1	-	-	-	-
1470	1380	S4313a	vap	451.6	666.0	333	0.01	1176.5	-	-	-	0.0	-	-	-
1471	1381	S4314a	vap	458.1	-	293	1.0	1845.2	-	-	-	0.0	-	-	-
1472	1382	S4315a		422.1	-	283	1.0	1027.5	999.6	-27.9	-2.7	-	-	-	-
1473	1383	S4316a	×	417.6	584.0	298	1.0	-	-	-	-	44.3	55.6	11.3	25.5
1474	1384	S4317a		433.1	-	296	1.0	1082.6	1009.4	-73.2	-6.8	-	-	-	-
1475	1385	S4318a		461.1	-	288	1.0	1108.0	1031.0	-77.0	-7.0	-	-	-	-
1476	1386	S4319a		452.1	-	336	1.0	-	-	-	-	60.0	56.1	-3.9	-6.6
1477	1387	S4320a		485.1	664.0	356	1.0	1046.1	1038.6	-7.5	-0.7	-	-	-	-
1478	1388	S4321a		479.6	-	293	1.0	1102.1	1041.2	-60.9	-5.5	-	-	-	-
1479	1389	S4322a	×	404.1	-	293	1.0	1051.1	946.6	-104.5	-9.9	-	-	-	-
1480	1390	S4401a		437.1	-	293	1.0	1395.6	1402.3	6.7	0.5	-	-	-	-
1481	1391	S4402a		-	-	293	1.0	1328.2	1296.2	-32.0	-2.4	-	-	-	-
1482	1392	S4403a		428.1	-	293	1.0	1282.7	1264.0	-18.7	-1.5	50.6	53.5	2.9	5.7
1483	1393	S4404a		479.1	-	293	1.0	1933.3	1906.8	-26.5	-1.4	-	-	-	-
1484	1394	S4405a		467.1	-	293	1.0	1899.1	1866.6	-32.5	-1.7	-	-	-	-
1485	1395	S4406a	×	373.1	-	293	1.0	1176.5	1064.8	-111.7	-9.5	-	-	-	-
1486	1396	S4407a		-	-	288	1.0	-	-	-	-	55.1	49.6	-5.5	-9.9

Table S.14 – Comparison of experimental and simulated properties using WH combination rules (continued).

n_{sim}	n_{iso}	Code	Outlier	T_m [K]	T_b [K]	T [K]	P [bar]	ρ_{liq}[kg·m^{-3}] exp	sim	dev	err [%]	ΔH_{vap}[kJ·mol^{-1}] exp	sim	dev	err [%]
1487	1397	S4408a		402.1	-	293	1.0	1225.0	1171.5	-53.5	-4.4	-	-	-	-
1488	1398	S4409a		393.1	-	288	1.0	-	-	-	-	56.4	52.7	-3.7	-6.6
1489	1399	S4501a		440.6	-	293	1.0	1383.6	1370.5	-13.1	-0.9	51.0	55.1	4.1	8.0
1490	1400	S4502a	vap	334.1	-	293	1.0	1194.0	-	-	-	0.0	-	-	-
1491	1401	S5201a		396.5	-	293	1.0	1052.3	1043.3	-9.0	-0.9	-	-	-	-
1492	1402	S5202a		392.6	-	298	1.0	-	-	-	-	43.6	42.3	-1.3	-2.9
1493	1403	S5203a		413.6	-	293	1.0	952.6	906.6	-46.0	-4.8	-	-	-	-
1494	1404	S5204a		482.1	-	293	1.0	1007.1	958.3	-48.8	-4.8	-	-	-	-
1495	1405	S5205a		423.4	-	299	1.0	882.0	875.1	-6.9	-0.8	-	-	-	-
1496	1406	S5206a		397.6	-	298	1.0	837.0	849.0	12.0	1.4	-	-	-	-
1497	1407	S5207a		436.6	-	298	1.01	880.8	872.5	-8.3	-0.9	57.0	57.0	-0.0	-0.0
1498	1408	S5208a		446.1	-	298	1.0	893.2	867.0	-26.2	-2.9	-	-	-	-
1499	1409	S5209a		455.1	-	293	1.0	900.5	889.8	-10.7	-1.2	-	-	-	-
1500	1410	S5210a		408.4	-	293	1.0	916.5	868.5	-48.0	-5.2	-	-	-	-
1501	1411	S5211a		430.1	614.5	296	1.0	923.0	880.1	-42.9	-4.6	-	-	-	-
1502	1412	S5212a		418.1	614.5	298	1.01	899.7	862.7	-36.9	-4.1	50.1	50.1	-0.0	-0.0
1503	1413	S5213a		404.1	614.5	298	1.01	896.6	849.1	-47.5	-5.3	-	-	-	-
1504	1414	S5214a		424.5	615.2	298	1.01	907.9	870.3	-37.6	-4.1	52.1	55.1	3.0	5.8
1505	1415	S5301a	×	-	-	293	1.0	1108.5	1027.5	-81.0	-7.3	-	-	-	-
1506	1416	S5302a	×	503.1	-	298	1.0	1341.6	1180.2	-161.4	-12.0	-	-	-	-
1507	1417	S5303a		423.6	-	293	1.0	1088.8	1049.4	-39.4	-3.6	-	-	-	-
1508	1418	S5304a		420.1	-	293	1.0	1079.3	1045.8	-33.5	-3.1	-	-	-	-
1509	1419	S5305a		434.1	-	293	1.0	1104.0	1073.4	-30.6	-2.8	-	-	-	-
1510	1420	S5306a		435.1	-	293	1.0	1108.6	1072.0	-36.6	-3.3	-	-	-	-
1511	1421	S5307a		447.1	-	293	1.0	1129.3	1087.6	-41.7	-3.7	-	-	-	-
1512	1422	S5308a		441.1	-	293	1.0	1452.8	1374.5	-78.3	-5.4	-	-	-	-
1513	1423	S5309a		433.1	-	293	1.0	1413.5	1349.0	-64.5	-4.6	-	-	-	-
1514	1424	S5310a		452.1	-	291	1.0	1412.3	1383.5	-28.8	-2.0	-	-	-	-
1515	1425	S5311a		459.6	-	298	1.0	1400.0	1394.9	-5.1	-0.4	-	-	-	-
1516	1426	S5312a		-	-	288	1.0	-	-	-	-	44.3	43.2	-1.1	-2.4
1517	1427	S5313a		-	-	288	1.0	-	-	-	-	47.3	47.4	0.1	0.2
1518	1428	S5314a		-	-	293	1.0	-	-	-	-	43.9	45.4	1.5	3.4
1519	1429	S5315a		519.0	738.0	308	1.0	1120.1	1098.8	-21.3	-1.9	-	-	-	-
1520	1430	S5316a		444.1	-	293	1.0	1075.7	1010.4	-65.3	-6.1	-	-	-	-
1521	1431	S5317a		428.1	-	288	1.0	1059.6	1019.7	-39.9	-3.8	-	-	-	-
1522	1432	S5318a	×	444.9	642.0	333	0.01	1035.7	988.2	-47.5	-4.6	45.6	55.8	10.2	22.3
1523	1433	S5319a	×	426.0	588.0	293	1.0	938.4	1045.5	107.1	11.4	-	-	-	-
1524	1434	S5320a		415.6	-	288	1.0	1013.9	942.5	-71.4	-7.0	-	-	-	-
1525	1435	S5321a		416.1	-	292	1.0	1007.4	940.7	-66.7	-6.6	-	-	-	-
1526	1435	S5321b		416.1	-	298	1.0	-	-	-	-	50.3	46.9	-3.4	-6.7
1527	1436	S5401a		456.6	-	293	1.0	1240.1	1229.6	-10.5	-0.8	-	-	-	-
1528	1437	S5402a		487.6	-	293	1.0	1796.6	1777.8	-18.8	-1.0	-	-	-	-
1529	1438	S5403a		-	-	288	1.0	-	-	-	-	54.5	54.0	-0.5	-0.9
1530	1439	S5501a		448.1	-	298	1.0	1291.1	1285.5	-5.6	-0.4	-	-	-	-
1531	1440	S5502a		460.1	-	293	1.0	1322.1	1314.8	-7.3	-0.5	-	-	-	-
1532	1441	S6201a	×	-	-	293	1.0	1250.0	1454.2	204.2	16.3	-	-	-	-
1533	1442	S6202a		431.3	-	293	1.0	1024.1	1027.2	3.1	0.3	-	-	-	-
1534	1443	S6203a		441.0	606.0	293	1.0	938.7	905.8	-32.9	-3.5	-	-	-	-

Table S.14 – Comparison of experimental and simulated properties using WH combination rules (continued).

n_{sim}	n_{iso}	Code	Outlier	T_m [K]	T_b [K]	T [K]	P [bar]	ρ_{liq}[kg·m^{-3}] exp	sim	dev	err [%]	ΔH_{vap}[kJ·mol^{-1}] exp	sim	dev	err [%]
1535	1444	S6204a	×	436.1	-	298	1.01	880.4	871.3	-9.1	-1.0	52.5	63.1	10.6	20.2
1536	1445	S6205a		472.1	-	293	1.0	890.7	890.4	-0.3	-0.0	-	-	-	-
1537	1446	S6206a		410.6	637.8	293	1.0	879.0	844.0	-35.0	-4.0	-	-	-	-
1538	1447	S6207a		423.1	637.8	298	1.01	880.8	853.4	-27.4	-3.1	-	-	-	-
1539	1448	S6208a		433.1	637.8	293	1.0	890.0	865.8	-24.2	-2.7	-	-	-	-
1540	1449	S6209a		444.5	633.9	298	1.01	896.2	874.3	-21.9	-2.4	56.6	59.6	3.0	5.4
1541	1450	S6301a		424.6	-	293	1.0	1031.5	1006.9	-24.6	-2.4	-	-	-	-
1542	1451	S6302a		453.1	-	293	1.0	1065.6	1050.7	-14.9	-1.4	-	-	-	-
1543	1452	S6303a		456.1	-	293	1.0	1070.4	1053.3	-17.1	-1.6	-	-	-	-
1544	1453	S6304a		457.1	-	293	1.0	1075.6	1053.4	-22.2	-2.1	-	-	-	-
1545	1454	S6305a		436.1	-	293	1.0	1326.3	1284.6	-41.7	-3.1	-	-	-	-
1546	1455	S6306a		450.1	-	293	1.0	1327.3	1309.0	-18.3	-1.4	-	-	-	-
1547	1456	S6307a		465.1	-	293	1.0	1354.0	1332.5	-21.5	-1.6	-	-	-	-
1548	1457	S6308a		-	-	288	1.0	-	-	-	-	54.9	52.3	-2.6	-4.7
1549	1458	S6309a		-	-	296	1.0	1086.1	1103.9	17.8	1.6	-	-	-	-
1550	1459	S6310a		460.1	-	293	1.0	1103.0	1099.4	-3.6	-0.3	-	-	-	-
1551	1460	S6311a		489.1	-	293	1.0	1136.0	1119.4	-16.6	-1.5	-	-	-	-
1552	1461	S6312a		440.1	-	293	1.0	998.0	937.6	-60.4	-6.1	-	-	-	-
1553	1462	S6313a	×	450.6	-	298	1.0	-	-	-	-	55.9	64.4	8.5	15.2
1554	1463	S6314a		547.6	-	298	1.0	1090.0	1078.2	-11.8	-1.1	-	-	-	-
1555	1464	S6315a		450.6	-	298	1.0	1021.7	980.8	-40.9	-4.0	-	-	-	-
1556	1465	S6316a		453.9	643.0	283	1.0	1036.8	1000.3	-36.5	-3.5	-	-	-	-
1557	1466	S6317a		469.1	-	293	1.0	1051.1	1004.5	-46.6	-4.4	-	-	-	-
1558	1467	S6318a		431.1	-	293	1.0	970.2	916.5	-53.7	-5.5	-	-	-	-
1559	1468	S6319a		429.4	607.3	293	1.0	974.0	921.4	-52.6	-5.4	52.7	49.4	-3.3	-6.2
1560	1469	S6401a		466.6	-	293	1.0	1182.0	1183.1	1.1	0.1	-	-	-	-
1561	1470	S6402a		495.6	-	293	1.0	1698.7	1695.2	-3.5	-0.2	-	-	-	-
1562	1471	S6403a		482.9	-	293	1.0	1146.6	1104.8	-41.8	-3.6	-	-	-	-
1563	1472	S6404a		482.9	-	298	1.0	1169.0	1111.4	-57.6	-4.9	-	-	-	-
1564	1473	S6501a		461.1	-	293	1.0	1263.6	1257.0	-6.6	-0.5	-	-	-	-
1565	1474	S6502a		477.1	-	293	1.0	1277.8	1276.3	-1.5	-0.1	-	-	-	-
1566	1475	S6503a		375.1	-	295	1.0	1026.8	1020.0	-6.8	-0.7	-	-	-	-
1567	1476	S7201a		430.1	-	298	1.0	-	-	-	-	47.7	49.2	1.5	3.1
1568	1477	S7202a		462.6	-	293	1.0	860.0	878.6	18.6	2.2	-	-	-	-
1569	1478	S7203a		433.1	-	298	1.0	898.0	874.1	-23.9	-2.7	-	-	-	-
1570	1479	S7204a		420.2	659.3	320	0.01	-	-	-	-	47.7	46.8	-0.8	-1.8
1571	1480	S7205a		444.6	659.3	298	1.0	874.6	859.0	-15.6	-1.8	-	-	-	-
1572	1481	S7301a		456.1	-	293	1.0	1025.2	1017.9	-7.3	-0.7	-	-	-	-
1573	1482	S7302a		457.1	-	293	1.0	1025.3	1014.6	-10.7	-1.0	-	-	-	-
1574	1483	S7303a		464.4	-	293	1.0	1032.3	1021.3	-11.0	-1.1	-	-	-	-
1575	1484	S7304a		449.4	-	293	1.0	1037.0	1035.5	-1.5	-0.1	-	-	-	-
1576	1485	S7305a		459.1	-	293	1.0	1276.0	1265.1	-10.9	-0.9	-	-	-	-
1577	1486	S7306a		464.1	-	291	1.0	1226.0	1271.2	45.2	3.7	-	-	-	-
1578	1487	S7307a		499.9	-	293	1.0	1308.5	1290.3	-18.2	-1.4	-	-	-	-
1579	1488	S7308a		-	-	288	1.0	-	-	-	-	54.6	57.0	2.4	4.4
1580	1489	S7309a		-	-	288	1.0	-	-	-	-	56.8	59.3	2.5	4.3
1581	1490	S7310a		455.1	-	287	1.0	995.0	976.3	-18.7	-1.9	-	-	-	-
1582	1491	S7311a		459.1	-	293	1.0	983.5	961.0	-22.5	-2.3	-	-	-	-

Table S.14 – Comparison of experimental and simulated properties using WH combination rules (continued).

n_{sim}	n_{iso}	Code	Outlier	T_m [K]	T_b [K]	T [K]	P [bar]	ρ_{liq}[kg·m^{-3}] exp	sim	dev	err [%]	ΔH_{vap}[kJ·mol^{-1}] exp	sim	dev	err [%]
1583	1492	S7312a		460.1	-	293	1.0	994.1	962.2	-31.9	-3.2	-	-	-	-
1584	1493	S7313a		464.1	-	293	1.0	1012.0	976.4	-35.6	-3.5	-	-	-	-
1585	1494	S7314a		478.9	-	293	1.0	1011.1	980.1	-31.0	-3.1	-	-	-	-
1586	1495	S7315a		438.1	621.0	293	1.0	949.0	912.7	-36.3	-3.8	-	-	-	-
1587	1496	S7316a		442.2	-	298	1.0	-	-	-	-	55.6	52.8	-2.8	-5.0
1588	1497	S7401a		464.2	-	293	1.0	1062.3	1090.5	28.2	2.7	-	-	-	-
1589	1498	S7501a		490.1	-	293	1.0	1231.4	1235.2	3.8	0.3	-	-	-	-
1590	1499	S8201a		458.1	-	289	1.0	910.7	912.8	2.1	0.2	-	-	-	-
1591	1500	S8202a		463.1	-	298	1.0	826.0	856.7	30.7	3.7	-	-	-	-
1592	1501	S8203a		481.1	679.3	293	1.0	887.8	884.0	-3.8	-0.4	-	-	-	-
1593	1502	S8301a		475.6	-	298	1.0	958.9	932.5	-26.4	-2.8	-	-	-	-
1594	1503	S8302a	×	-	-	298	1.0	-	-	-	-	61.9	73.0	11.1	17.9
1595	1504	S8303a		460.1	-	293	1.0	975.6	934.2	-41.4	-4.2	-	-	-	-
1596	1505	S8304a		471.1	-	289	1.0	984.7	956.4	-28.3	-2.9	-	-	-	-
1597	1506	S8305a		446.1	-	298	1.0	980.0	949.9	-30.1	-3.1	-	-	-	-
1598	1507	S8306a		469.0	-	298	1.0	967.1	966.9	-0.2	-0.0	-	-	-	-
1599	1508	S8307a		494.6	-	298	1.0	989.0	966.2	-22.8	-2.3	-	-	-	-
1600	1509	S8308a		465.7	641.2	298	1.0	-	-	-	-	59.5	57.3	-2.2	-3.8
1601	1510	S8401a		495.1	-	293	1.0	1073.2	1059.9	-13.3	-1.2	-	-	-	-
1602	1511	S9301a		-	-	291	1.0	970.0	948.2	-21.8	-2.2	-	-	-	-
1603	1512	S9302a		474.1	-	291	1.0	964.8	942.6	-22.2	-2.3	-	-	-	-
1604	1513	S9303a		497.1	-	293	1.0	966.1	947.5	-18.6	-1.9	-	-	-	-
1605	1514	S9304a		510.6	-	293	1.0	973.5	963.6	-9.9	-1.0	-	-	-	-
1606	1515	S0201a		473.7	-	293	1.0	922.0	911.3	-10.7	-1.2	75.3	78.6	3.3	4.4
1607	1516	S0301a		-	-	297	1.0	1191.2	1252.8	61.6	5.2	-	-	-	-

Code	P	T	GM			LB			WH			
			sim	dev	err [%]	sim	dev	err [%]	sim	dev	err [%]	exp
	[bar]	[K]					[mN·m^{-1}]					
F1101	38.4	298.1	0.1	-2.1	-97.6	0.2	-1.9	-90.3	0.3	-1.8	-86.5	2.1
C1101	1.0	229.0	24.2	-2.2	-8.4	22.1	-4.3	-16.4	23.8	-2.6	-10.0	26.4
C1201	1.0	298.1	28.2	1.0	3.5	27.6	0.4	1.5	29.2	2.0	7.4	27.2
C1301	1.0	298.1	29.2	2.6	9.8	30.5	3.9	14.7	35.1	8.5	31.8	26.6
C1401	1.0	298.1	27.9	1.7	6.6	30.0	3.8	14.5	33.9	7.7	29.2	26.2
C2202	1.0	298.1	32.1	0.3	1.0	30.6	-1.2	-3.9	29.8	-2.0	-6.4	31.8
C2301	1.0	301.4	23.1	-1.7	-6.9	23.2	-1.6	-6.3	25.3	0.5	1.9	24.8
C4101	1.0	298.1	15.5	-3.4	-18.1	15.1	-3.8	-20.0	15.1	-3.8	-20.3	18.9
B1201	1.0	298.1	40.1	1.1	2.8	40.0	1.0	2.5	40.4	1.4	3.6	39.0
B2101	1.0	298.1	21.6	-2.0	-8.5	21.2	-2.4	-10.1	21.9	-1.7	-7.0	23.6
B2202	1.0	298.2	40.1	1.9	4.9	39.4	1.2	3.2	38.1	-0.1	-0.3	38.2
B4101	1.0	298.1	19.2	-1.9	-9.2	18.9	-2.2	-10.6	19.0	-2.1	-9.8	21.1
I1101	1.0	298.1	27.1	-2.7	-9.0	27.2	-2.6	-8.8	27.4	-2.4	-8.0	29.8
I1201	1.0	293.1	48.6	-18.4	-27.5	50.3	-16.7	-25.0	48.8	-18.2	-27.2	67.0
I2101	1.0	293.5	26.9	-1.2	-4.2	27.5	-0.6	-2.2	28.2	0.1	0.3	28.1
I4101	1.0	298.1	16.6	-4.0	-19.6	23.4	2.8	13.6	23.2	2.6	12.8	20.6
I4104	1.0	298.1	28.1	-0.7	-2.4	28.5	-0.3	-1.2	29.2	0.4	1.5	28.8
O2101	1.0	248.2	23.0	4.3	23.2	21.7	3.0	16.1	18.9	0.2	1.0	18.7
O4103	1.0	296.7	15.4	-1.4	-8.6	15.4	-1.4	-8.6	16.5	-0.3	-1.9	16.8
O4202	1.0	298.1	27.2	3.3	13.8	26.5	2.6	10.9	24.6	0.7	2.7	23.9
O5203	1.0	294.2	18.4	-2.7	-13.0	18.0	-3.1	-14.7	20.6	-0.5	-2.3	21.1
O6204	1.0	298.1	21.9	3.3	17.5	22.8	4.1	22.3	22.6	4.0	21.6	18.6
O6301	1.0	298.1	31.7	2.4	8.1	31.2	1.9	6.3	28.9	-0.4	-1.3	29.3
K3101	1.0	298.1	24.1	1.0	4.3	21.9	-1.2	-5.1	25.1	2.0	8.5	23.1
K5103	1.0	293.1	25.5	0.7	2.9	25.2	0.4	1.6	25.7	0.9	3.7	24.8
K6106	1.0	298.1	25.4	0.1	0.2	25.4	0.0	0.2	25.8	0.4	1.8	25.4
K7115	1.0	298.1	26.2	0.1	0.3	26.4	0.3	1.1	26.0	-0.1	-0.3	26.1
K8122	1.0	295.2	27.1	1.5	5.9	27.5	1.9	7.6	26.5	0.9	3.7	25.6
K9121	1.0	293.4	27.9	1.6	6.2	28.5	2.2	8.4	28.3	2.0	7.8	26.3
K0113	1.0	298.1	27.7	2.1	8.1	27.7	2.1	8.3	27.6	2.0	7.9	25.6
E3202	1.0	298.1	23.4	-1.3	-5.4	24.8	0.1	0.6	25.1	0.4	1.7	24.7
E4204	1.0	298.1	22.9	-0.5	-2.0	24.2	0.8	3.5	24.3	0.9	4.0	23.4
E5208	1.0	298.1	23.1	-0.7	-3.1	24.0	0.2	0.6	24.0	0.2	1.0	23.8
E6215	1.0	298.1	25.0	0.7	3.1	26.2	1.9	7.8	26.1	1.8	7.5	24.3
E7225	1.0	298.1	25.7	0.5	2.0	26.6	1.4	5.6	26.4	1.2	4.9	25.2
E8235	1.0	298.1	26.3	-0.1	-0.3	27.5	1.1	4.3	27.0	0.6	2.4	26.4
E0226	1.0	298.1	27.3	1.8	7.1	28.7	3.2	12.4	27.7	2.2	8.5	25.5
L1101	1.0	298.1	14.0	-8.1	-36.5	14.4	-7.7	-35.0	16.0	-6.1	-27.8	22.1
L2101	1.0	298.1	16.9	-5.1	-23.1	17.1	-4.9	-22.2	18.0	-4.0	-18.1	22.0
L2201	1.0	298.1	37.9	-10.4	-21.5	30.0	-18.2	-37.8	29.1	-19.1	-39.7	48.2
L3101	1.0	298.1	16.9	-4.0	-18.9	17.3	-3.6	-17.3	18.3	-2.6	-12.4	20.9
L3102	1.0	298.1	19.5	-3.9	-16.7	19.5	-3.9	-16.7	20.5	-2.9	-12.3	23.4
L3201	1.0	298.1	32.0	-3.7	-10.4	31.6	-4.2	-11.6	24.6	-11.1	-31.1	35.7
L3202	1.0	298.1	31.6	-21.5	-40.5	32.2	-20.9	-39.3	35.9	-17.2	-32.5	53.1
L3301	1.0	298.1	42.4	-20.1	-32.2	30.2	-32.3	-51.6	25.1	-37.4	-59.9	62.5
L4101	1.0	308.1	15.2	-4.1	-21.1	14.7	-4.6	-23.6	16.9	-2.4	-12.7	19.3
L4104	1.0	298.1	20.7	-3.2	-13.5	21.0	-2.9	-12.0	22.6	-1.3	-5.3	23.9
L4205	1.0	298.1	27.1	-10.7	-28.4	26.5	-11.3	-29.9	27.9	-9.9	-26.3	37.8
L4206	1.0	298.1	34.4	-11.1	-24.5	34.1	-11.4	-25.1	37.0	-8.5	-18.8	45.5
L5104	1.0	298.1	20.5	-3.1	-13.1	20.5	-3.1	-13.1	21.4	-2.2	-9.4	23.6
L5105	1.0	298.1	21.8	-2.0	-8.2	21.9	-1.9	-7.8	22.6	-1.2	-5.2	23.8
L5106	1.0	298.1	20.8	-2.5	-10.6	20.7	-2.6	-11.3	22.2	-1.1	-4.5	23.3
L5107	1.0	298.1	22.8	-2.3	-9.3	23.2	-1.9	-7.5	23.8	-1.3	-5.2	25.1
L6117	1.0	298.1	24.2	-1.2	-4.9	24.2	-1.2	-4.6	24.8	-0.6	-2.2	25.4
L6206	1.0	293.1	21.9	-11.2	-33.8	22.1	-11.0	-33.2	22.4	-10.7	-32.4	33.1
L7137	1.0	298.2	25.0	-1.8	-6.9	24.7	-2.1	-8.0	25.7	-1.1	-4.2	26.8
L8157	1.0	298.1	25.5	-1.6	-5.8	26.1	-1.0	-3.6	25.4	-1.7	-6.1	27.1
D2201	1.0	298.1	39.3	12.3	45.5	38.3	11.3	41.9	39.0	12.0	44.5	27.0
D4201	1.0	296.6	30.0	5.2	20.8	28.5	3.7	14.8	26.8	2.0	7.9	24.8
D8208	1.0	293.1	28.0	-1.2	-4.0	29.0	-0.2	-0.6	30.0	0.8	2.7	29.2
D9205	1.0	306.6	30.2	-6.0	-16.7	29.7	-6.5	-17.9	29.0	-7.2	-19.8	36.2
M3201	1.0	320.0	33.9	2.6	8.2	33.1	1.8	5.8	32.9	1.6	5.2	31.3
M4201	1.0	298.1	39.3	6.2	18.6	37.9	4.8	14.6	37.4	4.3	12.9	33.1
S1302	1.0	232.3	20.6	1.9	10.4	22.1	3.4	18.3	28.1	9.4	50.3	18.7
S1405	1.0	243.2	17.2	1.3	8.4	18.0	2.1	13.4	22.6	6.7	42.4	15.9
S1406	1.0	297.0	19.2	1.1	6.0	20.9	2.8	15.3	24.9	6.8	37.5	18.1

Table S.15: Experimental and simulated values for the surface-tension coefficient γ.

				GM			LB			WH		
Code	P	T	sim	dev	err [%]	sim	dev	err [%]	sim	dev	err [%]	exp
F1101	1.0	193.7	18.8	-6.3	-25.1	19.2	-5.9	-23.5	18.4	-6.7	-26.7	25.1
C1101	1.0	247.2	9.9	-3.1	-23.8	5.5	-7.5	-57.7	3.9	-9.1	-70.0	13.0
C1201	1.0	298.1	9.4	0.6	6.8	5.9	-2.9	-33.0	3.0	-5.8	-65.9	8.8
C1301	1.0	298.1	4.2	-0.5	-10.6	3.3	-1.4	-29.8	3.8	-0.9	-19.1	4.7
C1401	1.0	298.1	1.0	-1.2	-54.5	1.0	-1.2	-54.5	1.0	-1.2	-54.5	2.2
C2202	1.0	298.2	3.0	-7.2	-70.6	5.4	-4.8	-47.1	3.1	-7.1	-69.6	10.2
C2301	1.0	298.1	3.5	-3.6	-50.7	2.0	-5.1	-71.8	2.0	-5.1	-71.8	7.1
C4101	1.0	298.2	5.4	-4.2	-43.7	2.5	-7.1	-74.0	2.7	-6.9	-71.9	9.6
B1201	1.0	298.1	3.7	-3.5	-48.6	2.9	-4.3	-59.7	1.1	-6.1	-84.7	7.2
B2101	1.0	298.1	3.3	-5.7	-63.3	2.7	-6.3	-70.0	1.1	-7.9	-87.8	9.0
B2202	1.0	298.2	1.8	-3.0	-62.5	3.6	-1.2	-25.0	1.1	-3.7	-77.1	4.8
B4101	1.0	293.1	2.4	-8.6	-78.2	1.5	-9.5	-86.4	1.0	-10.0	-90.9	11.0
I1101	1.0	298.2	1.1	-5.8	-84.1	1.0	-5.9	-85.5	1.0	-5.9	-85.5	6.9
I1201	1.0	298.1	1.0	-4.3	-81.1	1.2	-4.1	-77.4	1.0	-4.3	-81.1	5.3
I2101	1.0	298.2	1.0	-6.6	-86.8	1.2	-6.4	-84.2	1.0	-6.6	-86.8	7.6
I4101	1.0	298.1	1.0	-5.4	-84.4	1.0	-5.4	-84.4	1.0	-5.4	-84.4	6.4
I4104	1.0	298.1	1.0	-5.2	-83.9	1.1	-5.1	-82.3	1.0	-5.2	-83.9	6.2
O2101	1.0	246.6	22.7	16.1	243.9	23.4	16.8	254.5	7.7	1.1	16.7	6.6
O4103	1.0	298.1	5.1	0.9	21.4	6.0	1.8	42.9	2.3	-1.9	-45.2	4.2
O4202	1.0	298.1	11.9	4.7	65.3	16.1	8.9	123.6	7.2	0.0	0.0	7.2
O5203	1.0	293.1	3.3	0.8	32.0	3.5	1.0	40.0	1.8	-0.7	-28.0	2.5
O6204	1.0	298.1	9.8	5.9	151.3	12.5	8.6	220.5	4.7	0.8	20.5	3.9
O6301	1.0	298.2	13.6	6.3	86.3	21.6	14.3	195.9	8.9	1.6	21.9	7.3
K3101	1.0	298.2	6.8	-13.8	-67.0	1.1	-19.5	-94.7	10.8	-9.8	-47.6	20.6
K5103	1.0	298.1	5.0	-10.2	-67.1	1.1	-14.1	-92.8	8.0	-7.2	-47.4	15.2
K6106	1.0	303.1	4.3	-9.7	-69.3	1.1	-12.9	-92.1	6.8	-7.2	-51.4	14.0
K7115	1.0	298.2	3.9	-7.8	-66.7	1.1	-10.6	-90.6	6.2	-5.5	-47.0	11.7
K8122	1.0	298.2	3.6	-5.9	-62.1	1.1	-8.4	-88.4	5.7	-3.8	-40.0	9.5
K9121	1.0	295.2	3.3	-5.8	-63.7	1.1	-8.0	-87.9	5.2	-3.9	-42.9	9.1
K0113	1.0	287.1	3.2	-5.1	-61.4	1.1	-7.2	-86.7	5.0	-3.3	-39.8	8.3
E3202	1.0	298.1	4.6	-2.1	-31.3	3.6	-3.1	-46.3	4.3	-2.4	-35.8	6.7
E4204	1.0	298.1	3.0	-3.0	-50.0	2.2	-3.8	-63.3	2.9	-3.1	-51.7	6.0
E5208	1.0	298.1	2.7	-3.0	-52.6	2.0	-3.7	-64.9	2.7	-3.0	-52.6	5.7
E6215	1.0	298.1	2.4	-2.6	-52.0	1.9	-3.1	-62.0	2.5	-2.5	-50.0	5.0
E7225	1.0	298.1	2.2	-2.5	-53.2	1.8	-2.9	-61.7	2.2	-2.5	-53.2	4.7
E8235	1.0	303.1	2.1	-2.3	-52.3	1.7	-2.7	-61.4	2.1	-2.3	-52.3	4.4
E0226	1.0	298.1	1.9	-2.2	-53.7	1.6	-2.5	-61.0	1.9	-2.2	-53.7	4.1
L1101	1.0	298.1	17.1	-15.5	-47.5	16.8	-15.8	-48.5	19.9	-12.7	-39.0	32.6
L2101	1.0	298.1	13.5	-11.4	-45.8	13.4	-11.5	-46.2	15.5	-9.4	-37.8	24.9
L2201	1.0	298.1	23.8	-16.8	-41.4	37.7	-2.9	-7.1	71.6	31.0	76.4	40.6
L3101	1.0	298.1	11.6	-7.7	-39.9	10.7	-8.6	-44.6	13.2	-6.1	-31.6	19.3
L3102	1.0	298.1	12.0	-8.5	-41.5	11.2	-9.3	-45.4	12.7	-7.8	-38.0	20.5
L3201	1.0	298.1	17.3	-11.4	-39.7	2.5	-26.2	-91.3	20.0	-8.7	-30.3	28.7
L3202	1.0	298.1	19.7	-14.6	-42.6	19.8	-14.5	-42.3	23.3	-11.0	-32.1	34.3
L3301	1.0	298.1	29.0	-15.1	-34.2	46.7	2.6	5.9	49.0	4.9	11.1	44.1
L4101	1.0	299.2	6.8	-5.5	-44.7	3.2	-9.1	-74.0	7.0	-5.3	-43.1	12.3
L4104	1.0	298.1	10.0	-7.4	-42.5	9.4	-8.0	-46.0	11.5	-5.9	-33.9	17.4
L4205	1.0	298.1	18.5	-10.2	-35.5	17.8	-10.9	-38.0	19.5	-9.2	-32.1	28.7
L4206	1.0	298.1	16.8	-14.8	-46.8	15.9	-15.7	-49.7	19.2	-12.4	-39.2	31.6
L5104	1.0	298.1	8.3	-4.9	-37.1	4.0	-9.2	-69.7	10.3	-2.9	-22.0	13.2
L5105	1.0	298.1	9.5	-5.4	-36.2	9.1	-5.8	-38.9	10.8	-4.1	-27.5	14.9
L5106	1.0	298.2	8.7	-4.5	-34.1	6.7	-6.5	-49.2	9.2	-4.0	-30.3	13.2
L5107	1.0	298.1	8.9	-6.1	-40.7	8.3	-6.7	-44.7	10.3	-4.7	-31.3	15.0
L6117	1.0	298.2	7.8	-5.1	-39.5	7.1	-5.8	-45.0	9.1	-3.8	-29.5	12.9
L6206	1.0	298.1	14.3	-10.8	-43.0	12.8	-12.3	-49.0	17.0	-8.1	-32.3	25.1
L7137	1.0	298.1	6.1	-5.4	-47.0	6.7	-4.8	-41.7	7.7	-3.8	-33.0	11.5
L8157	1.0	298.1	5.9	-4.1	-41.0	4.9	-5.1	-51.0	7.5	-2.5	-25.0	10.0
D2201	1.0	303.1	14.7	8.6	141.0	4.4	-1.7	-27.9	3.7	-2.4	-39.3	6.1
D4201	1.0	283.2	7.5	4.8	177.8	1.7	-1.0	-37.0	1.9	-0.8	-29.6	2.7
D8208	1.0	300.3	6.7	4.2	168.0	1.5	-1.0	-40.0	1.6	-0.9	-36.0	2.5
D9205	1.0	298.1	4.7	2.2	88.0	1.3	-1.2	-48.0	1.6	-0.9	-36.0	2.5
M3201	1.0	313.1	24.4	-139.4	-85.1	11.1	-152.7	-93.2	22.3	-141.5	-86.4	163.8
M4201	1.0	298.1	23.1	-16.3	-41.4	20.8	-18.6	-47.2	26.2	-13.2	-33.5	39.4
S1302	1.0	130.0	1.1	-23.8	-95.6	1.1	-23.8	-95.6	1.1	-23.8	-95.6	24.9
S1405	1.0	223.2	1.1	-1.4	-56.0	2.0	-0.5	-20.0	3.2	0.7	28.0	2.5
S1406	1.0	293.2	1.1	-1.9	-63.3	1.4	-1.6	-53.3	1.9	-1.1	-36.7	3.0

Table S.16: Experimental and simulated values for the static relative dielectric permittivity ϵ.

Code	P	T		GM			LB			WH		
			sim	dev	err [%]	sim	dev	err [%]	sim	dev	err [%]	exp
	[bar]	[K]					$[10^{-9}\ m^2{\cdot}s^{-1}]$					
F1101	2.0	178.0	3.164	-0.036	-1.1	3.117	-0.083	-2.6	3.023	-0.177	-5.5	3.200
C1101	1.0	233.0	3.932	0.532	15.6	4.390	0.990	29.1	4.291	0.891	26.2	3.400
C1201	1.0	298.2	3.229	-0.571	-15.0	3.494	-0.306	-8.1	3.356	-0.444	-11.7	3.800
C1301	1.0	298.2	2.011	-1.029	-33.8	1.893	-1.147	-37.7	1.592	-1.448	-47.6	3.040
C1401	1.0	298.2	1.311	-0.029	-2.2	1.214	-0.126	-9.4	0.974	-0.366	-27.3	1.340
C2202	1.0	298.2	1.728	0.838	94.2	2.130	1.240	139.3	2.635	1.745	196.1	0.890
C2301	1.0	293.2	1.619	0.229	16.5	1.627	0.237	17.1	1.550	0.160	11.5	1.390
C4101	1.0	298.2	3.726	2.126	132.9	3.831	2.231	139.4	3.597	1.997	124.8	1.600
B1201	1.0	297.8	1.514	-0.012	-0.8	1.633	0.107	7.0	1.629	0.103	6.7	1.526
B2101	1.0	295.7	3.997	0.197	5.2	4.169	0.369	9.7	4.076	0.276	7.3	3.800
B2202	1.0	294.7	1.028	0.248	31.8	1.165	0.385	49.4	1.273	0.493	63.2	0.780
B4101	1.0	300.1	2.478	0.968	64.1	2.463	0.953	63.1	2.450	0.940	62.3	1.510
I1101	1.0	298.2	3.438	-0.062	-1.8	3.279	-0.221	-6.3	3.236	-0.264	-7.5	3.500
I1201	1.0	297.1	0.865	0.333	62.6	0.813	0.281	52.8	0.852	0.320	60.2	0.532
I2101	1.0	292.5	2.695	0.483	21.8	2.670	0.458	20.7	2.651	0.439	19.8	2.212
I4101	1.0	296.1	1.611	0.671	71.4	1.566	0.626	66.6	1.601	0.661	70.3	0.940
I4104	1.0	292.5	1.800	0.453	33.6	1.767	0.420	31.2	1.810	0.463	34.4	1.347
O2101	19.7	235.5	3.785	1.625	75.2	3.941	1.781	82.5	4.649	2.489	115.2	2.160
O4103	1.0	287.4	5.510	-0.790	-12.5	5.312	-0.988	-15.7	5.446	-0.854	-13.6	6.300
O4202	1.0	292.9	1.832	-1.168	-38.9	2.014	-0.986	-32.9	2.360	-0.640	-21.3	3.000
O5203	1.0	294.7	3.797	0.597	18.7	3.651	0.451	14.1	3.385	0.185	5.8	3.200
O6204	1.0	303.2	2.433	0.233	10.6	2.162	-0.038	-1.7	2.640	0.440	20.0	2.200
O6301	1.0	303.2	0.879	-0.201	-18.6	0.846	-0.234	-21.7	1.268	0.188	17.4	1.080
K3101	1.0	298.2	4.126	-0.404	-8.9	4.971	0.441	9.7	3.911	-0.619	-13.7	4.530
K5103	1.0	298.2	2.918	-0.052	-1.8	3.307	0.337	11.3	2.717	-0.253	-8.5	2.970
K6106	1.0	298.2	2.487	0.427	20.7	2.652	0.592	28.7	2.194	0.134	6.5	2.060
K7115	1.0	298.2	1.896	0.286	17.8	2.254	0.644	40.0	1.806	0.196	12.2	1.610
K8122	1.0	298.2	1.589	0.409	34.7	1.795	0.615	52.1	1.451	0.271	23.0	1.180
K9121	1.0	298.2	1.313	0.373	39.7	1.428	0.488	51.9	1.211	0.271	28.8	0.940
K0113	1.0	298.2	1.087	0.337	44.9	1.183	0.433	57.7	0.963	0.213	28.4	0.750
E3202	1.0	298.2	4.277	0.997	30.4	3.992	0.712	21.7	3.544	0.264	8.0	3.280
E4204	1.0	298.2	3.552	0.782	28.2	3.393	0.623	22.5	3.262	0.492	17.8	2.770
E5208	1.0	303.2	3.025	0.525	21.0	3.153	0.653	26.1	3.102	0.602	24.1	2.500
E6215	1.0	303.2	2.407	0.697	40.8	2.149	0.439	25.7	2.272	0.562	32.9	1.710
E7225	1.0	303.2	1.837	0.477	35.1	1.721	0.361	26.5	1.720	0.360	26.5	1.360
E8235	1.0	303.2	1.480	0.390	35.8	1.387	0.297	27.2	1.441	0.351	32.2	1.090
E0226	1.0	303.2	1.114	0.394	54.7	0.987	0.267	37.1	1.012	0.292	40.6	0.720
L1101	1.0	298.2	2.383	-0.567	-19.2	2.239	-0.711	-24.1	2.428	-0.522	-17.7	2.950
L2101	1.0	298.2	1.226	0.066	5.7	1.191	0.031	2.7	1.295	0.135	11.6	1.160
L2201	1.0	298.2	0.088	-0.012	-12.0	0.005	-0.095	-95.0	0.098	-0.002	-2.0	0.100
L3101	1.0	298.2	0.568	-0.012	-2.1	0.602	0.022	3.8	0.536	-0.044	-7.6	0.580
L3102	1.0	298.2	0.647	0.057	9.7	0.682	0.092	15.6	0.692	0.102	17.3	0.590
L3201	1.0	304.0	0.051	-0.009	-15.0	0.002	-0.058	-96.7	0.041	-0.019	-31.7	0.060
L3202	1.0	304.0	0.069	0.009	15.0	0.095	0.035	58.3	0.104	0.044	73.3	0.060
L3301	1.0	298.1	0.007	0.005	250.0	0.009	0.007	350.0	0.090	0.088	4400.0	0.002
L4101	1.0	299.4	0.707	0.397	128.1	0.837	0.527	170.0	0.428	0.118	38.1	0.310
L4104	1.0	298.2	0.432	-0.028	-6.1	0.462	0.002	0.4	0.458	-0.002	-0.4	0.460
L4205	1.0	297.4	0.039	-0.071	-64.5	0.091	-0.019	-17.3	0.035	-0.075	-68.2	0.110
L4206	1.0	304.0	0.041	0.011	36.7	0.031	0.001	3.3	0.054	0.024	80.0	0.030
L5104	4.9	297.2	0.244	0.104	74.3	0.402	0.262	187.1	0.177	0.037	26.4	0.140
L5105	1.0	296.2	0.277	0.007	2.6	0.246	-0.024	-8.9	0.301	0.031	11.5	0.270
L5106	9.9	296.9	0.274	0.154	128.3	0.347	0.227	189.2	0.246	0.126	105.0	0.120
L5107	1.0	298.2	0.308	0.008	2.7	0.361	0.061	20.3	0.370	0.070	23.3	0.300
L6117	1.0	298.2	0.244	0.024	10.9	0.263	0.043	19.5	0.263	0.043	19.5	0.220
L6206	1.0	297.4	0.197	-0.033	-14.3	0.175	-0.055	-23.9	0.119	-0.111	-48.3	0.230
L7137	1.0	298.2	0.194	0.024	14.1	0.201	0.031	18.2	0.213	0.043	25.3	0.170
L8157	1.0	298.2	0.159	0.019	13.6	0.178	0.038	27.1	0.166	0.026	18.6	0.140
D2201	1.0	298.2	0.083	-1.107	-93.0	0.370	-0.820	-68.9	0.044	-1.146	-96.3	1.190
D4201	1.0	299.5	0.056	-0.774	-93.3	0.424	-0.406	-48.9	0.284	-0.546	-65.8	0.830
D8208	1.0	323.2	0.148	-0.242	-62.1	0.366	-0.024	-6.2	0.226	-0.164	-42.1	0.390
D9205	1.0	298.2	0.042	-0.118	-73.7	0.138	-0.022	-13.7	0.079	-0.081	-50.6	0.160
M3201	1.0	305.5	0.414	0.044	11.9	0.552	0.182	49.2	0.526	0.156	42.2	0.370
M4201	9.9	298.0	1.164	0.374	47.3	1.260	0.470	59.5	1.206	0.416	52.7	0.790
S1302	1.0	232.0	2.257	-1.053	-31.8	2.229	-1.081	-32.7	1.310	-2.000	-60.4	3.310
S1405	1.0	293.2	4.496	0.396	9.7	4.236	0.136	3.3	2.795	-1.305	-31.8	4.100
S1406	1.1	379.0	5.955	-0.065	-1.1	5.597	-0.423	-7.0	4.220	-1.800	-29.9	6.020

Table S.17: Experimental and simulated values for the self-diffusion coefficient D.

S.11 Outliers

In this section, we provide more information on the simulation results considered as outliers in the comparison between experimental and simulated properties, namely 114, 68, and 101 simulations concerning 105, 61, and 91 molecules for GM, LB, and WH, respectively. These are marked in Tabs. S.12, S.13, and S.14. They are selected based on errors larger than 80.0 $kg{\cdot}m^{-3}$ for ρ_{liq} and/or larger than 8.0 $kJ{\cdot}mol^{-1}$ for ΔH_{vap}. The corresponding structures are shown in Figs. S.8, S.9, and S.10.

Figure S.8: Structure of the 105 compounds considered as outliers with the GM combination rule.

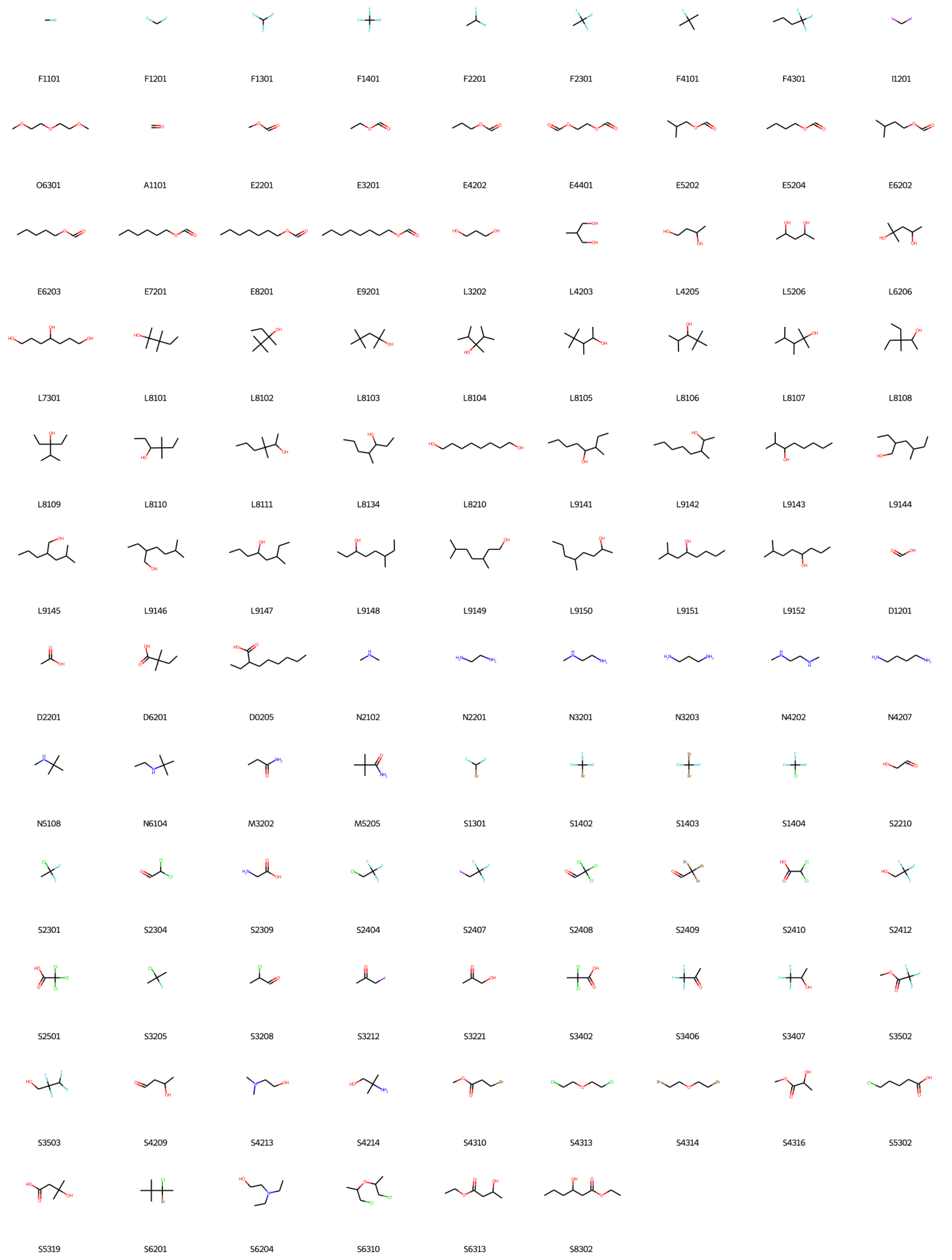

Figure S.9: Structure of the 61 compounds considered as outliers with the LB combination rule.

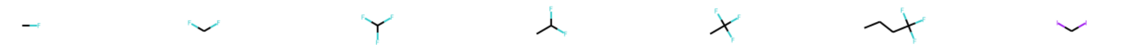

F1101 F1201 F1301 F2201 F2301 F4301 I1201

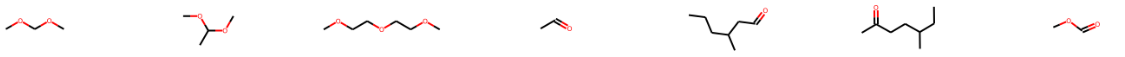

O3201 O4201 O6301 A2101 A7103 K8118 E2201

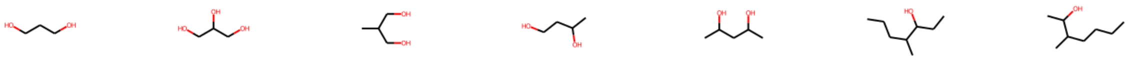

L3202 L3301 L4203 L4205 L5206 L8134 L8136

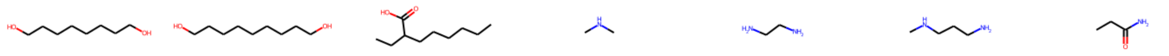

L8210 L9210 D0205 N2102 N2201 N4205 M3202

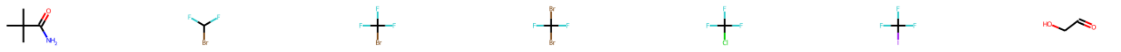

M5205 S1301 S1402 S1403 S1404 S1407 S2210

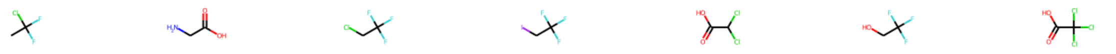

S2301 S2309 S2404 S2407 S2410 S2412 S2501

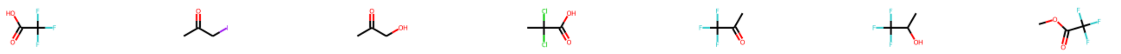

S2502 S3212 S3221 S3402 S3406 S3407 S3502

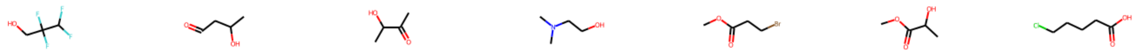

S3503 S4209 S4210 S4213 S4310 S4316 S5302

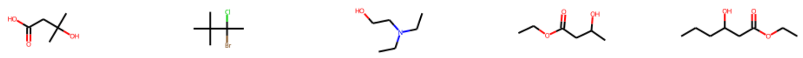

S5319 S6201 S6204 S6313 S8302

Figure S.10: Structure of the 91 compounds considered as outliers with the WH combination rule.

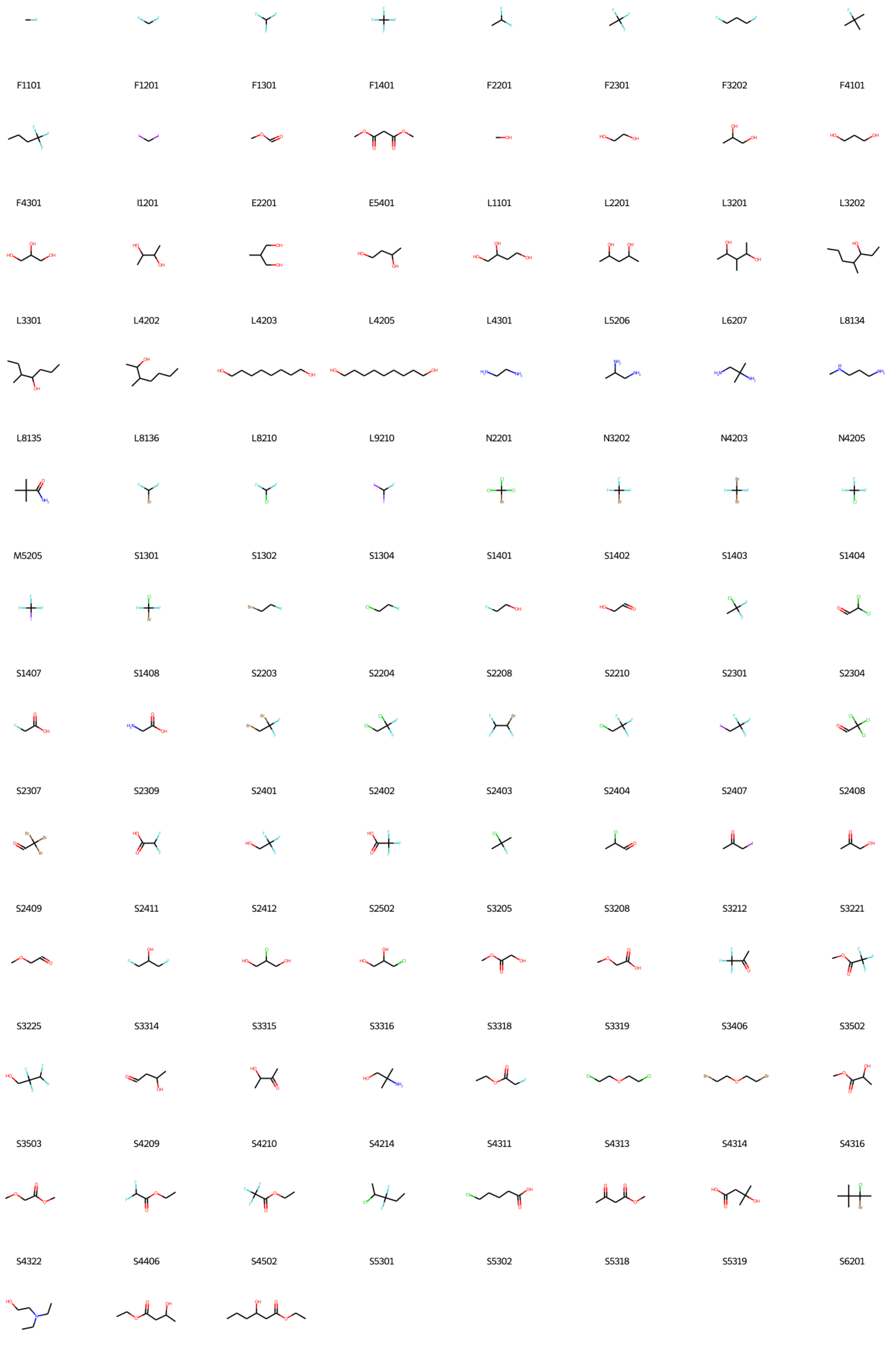

S.12 Effect on Interchanging Combination Rules

The matrices with the statistics concerning ρ_{liq} and ΔH_{vap} for simulations carried out with parameters optimized with a given combination rule but using the two other rules are shown in Fig. S.11.

HAL		ρ [kg m^{-3}]			ΔH_{vap} [kJ mol^{-1}]		
		GM	LB	WH	GM	LB	WH
RMSD	GM	39.0	50.4	53.0	1.5	1.9	3.6
RMSD	LB	36.5	41.6	54.6	1.5	1.8	3.5
RMSD	WH	110.3	86.3	41.5	4.6	4.1	2.4
AVED	GM	0.5	2.6	31.6	0.1	0.1	1.6
AVED	LB	-3.8	1.4	35.9	-0.4	-0.3	1.3
AVED	WH	-71.1	-58.5	3.8	-3.4	-3.1	-0.6

MIX		ρ [kg m^{-3}]			ΔH_{vap} [kJ mol^{-1}]		
		GM	LB	WH	GM	LB	WH
RMSD	GM	43.4	47.9	61.0	4.7	5.5	9.1
RMSD	LB	46.8	44.4	59.5	5.0	4.9	8.8
RMSD	WH	151.5	129.8	65.0	10.4	8.7	4.9
AVED	GM	-1.7	1.8	24.1	1.4	2.4	7.3
AVED	LB	-12.5	-4.6	23.3	-0.2	1.4	6.9
AVED	WH	-119.0	-105.7	-33.3	-8.5	-6.9	1.7

NHB		ρ [kg m^{-3}]			ΔH_{vap} [kJ mol^{-1}]		
		GM	LB	WH	GM	LB	WH
RMSD	GM	23.7	17.5	27.5	2.1	2.2	5.3
RMSD	LB	13.1	13.8	27.5	2.0	2.1	5.4
RMSD	WH	65.9	60.9	21.9	7.4	7.1	1.5
AVED	GM	-1.0	-6.5	18.0	0.5	-0.4	4.5
AVED	LB	-2.9	-4.6	20.9	0.5	0.0	4.6
AVED	WH	-54.8	-53.7	-9.7	-6.2	-6.2	0.3

HBD		ρ [kg m^{-3}]			ΔH_{vap} [kJ mol^{-1}]		
		GM	LB	WH	GM	LB	WH
RMSD	GM	27.8	21.0	36.2	4.0	3.8	5.9
RMSD	LB	31.2	22.0	37.8	5.0	4.0	5.6
RMSD	WH	75.2	56.5	29.7	11.3	9.0	4.2
AVED	GM	5.1	5.5	24.2	-0.7	0.1	4.0
AVED	LB	-0.6	3.7	26.7	-2.0	-0.7	3.6
AVED	WH	-53.9	-42.3	1.3	-9.0	-7.2	-0.4

ALL		ρ_{liq} [kg m^{-3}]			ΔH_{vap} [kJ mol^{-1}]		
		GM	LB	WH	GM	LB	WH
RMSD	GM	30.0	29.4	40.3	2.8	2.9	5.2
RMSD	LB	29.8	26.9	41.0	3.2	2.8	5.0
RMSD	WH	90.7	75.4	36.7	8.0	6.8	2.9
AVED	GM	2.0	0.9	23.1	0.1	0.1	3.4
AVED	LB	-2.7	0.0	25.6	-0.5	-0.1	3.3
AVED	WH	-63.1	-54.7	-5.6	-6.0	-5.2	0.0

Figure S.11: Statistics concerning the discrepancies between simulated and experimental properties considering the interchange of combination rules. Each entry corresponds to the results obtained with the combination rule specified in the given row and the parameters optimized using the combination rule specified in the given column. The root-mean-square (RMSD) and average (AVED) deviations are reported in terms of ρ_{liq} (left) and ΔH_{vap} (right) for the common set of 1447 molecules. The codes in the first column refer to molecules with two or more distinct functional groups (MIX), halogens (HAL), non-hydrogen-bonding (NHB), hydrogen-bonding (HBD), and the entire set (ALL) of compounds.

S.13 Results with Alternative Replica Sets

The results for the alternative replicas are shown here in terms of the final non-bonded parameters (Sec. S.13.1), the evolution of the non-bonded parameters (Sec. S.13.2), the charge distribution for the different LJ types (Sec. S.13.3), and the statistics concerning the discrepancies between simulated and experimental properties (Sec. S.13.4).

S.13.1 Final Values of the Non-Bonded Interaction Parameters

LJ type	GM					LB					WH				
	σ	$\tilde{\sigma}$	σ^*	ϵ	ϵ^*	σ	$\tilde{\sigma}$	σ^*	ϵ	ϵ^*	σ	$\tilde{\sigma}$	σ^*	ϵ	ϵ^*
		[nm]		[kJ·mol^{-1}]			[nm]		[kJ·mol^{-1}]			[nm]		[kJ·mol^{-1}]	
Carbon															
CH0	0.656	-	0.336	0.006	0.406	0.614	-	0.492	0.005	0.004	0.571	-	0.457	0.007	0.006
CH1	0.483	-	0.330	0.091	0.567	0.467	-	0.373	0.095	0.076	0.448	-	0.359	0.088	0.070
CH2	0.402	-	0.316	0.404	1.176	0.400	-	0.320	0.413	0.330	0.394	-	0.316	0.410	0.328
CH3	0.378	-	0.310	0.867	1.947	0.382	-	0.306	0.849	0.679	0.377	-	0.301	0.933	0.746
CH4	0.361	-	0.301	1.115	0.746	0.449	-	0.359	1.234	0.987	0.428	-	0.343	0.998	0.798
C=O	0.360	-	0.336	0.365	0.406	0.367	-	0.294	0.443	0.354	0.358	-	0.287	0.483	0.386
Halogen															
F	0.274	-	0.287	0.722	0.386	0.250	-	0.200	0.906	0.725	0.292	-	0.234	1.092	0.874
Cl	0.330	-	0.234	1.684	0.874	0.332	-	0.266	1.640	1.312	0.329	-	0.263	2.064	1.651
Br	0.349	-	0.263	2.387	1.651	0.354	-	0.283	2.331	1.865	0.354	-	0.283	2.504	2.004
I	0.383	-	0.283	3.178	2.004	0.386	-	0.309	3.162	2.529	0.387	-	0.310	3.122	2.498
Oxygen															
OC	0.307	0.352	0.287	0.707	1.011	0.311	0.338	0.248	0.508	0.407	0.334	0.377	0.267	0.520	0.416
O=C	0.274	0.304	0.263	1.122	1.725	0.252	0.280	0.201	1.087	0.869	0.252	0.286	0.201	1.387	1.109
OH	0.276	0.304	0.287	1.067	1.011	0.287	0.303	0.230	0.913	0.730	0.333	0.352	0.267	0.916	0.733
Nitrogen															
N_amn	0.288	0.281	0.298	0.482	0.877	0.302	0.294	0.242	0.514	0.411	0.249	0.254	0.199	0.519	0.415
N_amd	0.297	0.305	0.298	0.560	0.877	0.315	0.314	0.252	0.385	0.308	0.299	0.292	0.239	0.443	0.354
Hydrogen															
HC	0.222	-	0.239	0.143	0.354	0.221	-	0.177	0.140	0.112	0.219	-	0.176	0.099	0.080
HB	0.000	-	0.176	0.000	0.080	0.000	-	0.000	0.000	0.000	0.000	-	0.000	0.000	0.000

Table S.18: Final values of the LJ parameters of the force fields calibrated using GM, LB, and WH combination rules (considering the alternative calibration replicas). See also Tab. S.20 for the third replica with WH.

Atom type (EE-type)	LJ-type	GM		LB		WH	
		η [e^{-1}·V]	χ [V]	η [e^{-1}·V]	χ [V]	η [e^{-1}·V]	χ [V]
Aliphatic carbon (united-)atoms							
CH0	CH0	-	-	-	-	-	-
CH1	CH1	-	-	-	-	-	-
CH2	CH2	-	-	-	-	-	-
CH3	CH3	-	-	-	-	-	-
CH4	CH4	-	-	-	-	-	-
Halogen							
F_hal	F	29.987	17.177	33.898	17.879	36.749	19.991
Cl_hal	Cl	23.398	12.971	25.561	15.048	27.848	12.603
Br_hal	Br	22.867	10.218	26.856	13.721	25.651	9.576
I_hal	I	21.506	5.717	30.749	7.416	29.042	8.746
CH0_hal	CH0	10.632	8.287	8.434	8.436	10.945	8.161
CH1_hal	CH1	10.306	6.203	11.262	6.881	10.357	6.739
CH2_hal	CH2	10.410	7.165	8.972	9.454	7.429	9.510
CH3_hal	CH3	10.823	8.098	9.949	7.528	8.089	8.369
Ether							
O_eth	OC	10.651	10.958	13.986	12.453	13.282	10.914
CH0_O_eth	CH0	8.893	7.953	10.098	6.924	10.018	6.559
CH1_O_eth	CH1	11.681	6.574	9.470	6.426	10.238	7.318
CH2_O_eth	CH2	9.445	8.950	10.564	8.440	11.524	7.785
CH3_O_eth	CH3	10.076	8.201	12.611	5.821	10.906	6.868
Aldehyde							
H_CO_ald	HC	19.062	6.533	15.720	6.182	17.771	5.345
C_ald	C=O	9.488	6.136	8.755	8.904	9.609	8.775
O_ald	O=C	14.065	9.942	10.228	8.999	12.300	9.988
Ketone							
C_ket	C=O	7.065	7.863	8.757	5.050	6.691	5.917
O_ket	O=C	12.049	10.232	16.910	9.527	13.466	9.440
Ester							
H_CO_est	HC	21.171	6.693	16.638	7.079	15.422	5.979
C_est	C=O	15.936	6.430	12.559	7.298	10.876	6.678
O_est	O=C	17.093	8.884	13.942	8.834	15.465	9.629
O_C_est	OC	11.361	8.076	14.259	9.592	11.136	9.268
CH0_O_est	CH0	9.622	7.282	7.858	9.072	9.011	6.807
CH1_O_est	CH1	11.949	8.492	14.282	7.937	10.123	8.991
CH2_O_est	CH2	10.369	8.180	13.418	9.352	11.690	8.891
CH3_O_est	CH3	10.911	7.518	9.393	9.569	8.554	8.687
Alcohol							
H_ol	HB	13.753	4.937	13.222	5.335	14.498	4.746
O_ol	OH	13.606	10.324	13.910	10.972	12.420	11.633
CH0_O_ol	CH0	12.079	9.457	10.541	9.159	9.457	7.772
CH1_O_ol	CH1	10.813	8.560	14.588	7.225	11.985	7.076
CH2_O_ol	CH2	8.822	8.046	10.716	7.085	10.409	7.370
CH3_O_ol	CH3	11.954	7.184	8.201	6.542	11.162	6.719
Carboxylic acid							
H_CO_acd	HC	14.254	5.804	17.141	6.069	17.290	6.891
C_acd	C=O	9.321	8.218	10.028	6.015	9.611	6.532
O_acd	O=C	15.773	8.525	15.286	11.318	13.900	10.033
H_O_acd	HB	15.268	5.395	12.241	6.368	13.241	5.138
O_H_acd	OH	11.982	10.719	15.682	10.603	11.182	11.190
H_N_amn	HB	16.169	4.416	16.470	4.507	13.745	5.292
Amine							
N_amn	N_amn	12.927	7.420	10.893	9.257	11.264	8.725
CH0_N_amn	CH0	9.303	8.628	10.238	8.512	9.943	8.670
CH1_N_amn	CH1	12.154	9.445	10.958	8.337	10.635	8.827
CH2_N_amn	CH2	9.009	8.209	11.481	7.552	12.443	7.488
CH3_N_amn	CH3	12.297	8.367	13.343	6.872	14.281	5.588
Amide							
H_N_amd	HC	15.123	6.345	16.448	6.361	17.339	5.384
C_amd	C=O	10.209	5.513	10.112	5.622	8.995	5.804
O_amd	O=C	12.205	10.900	11.869	10.209	12.474	10.071
N_amd	N_amd	10.441	9.690	10.406	10.797	9.581	9.748
CH0_N_amd	CH0	-	-	-	-	-	-
CH1_N_amd	CH1	8.259	5.912	9.866	7.695	9.329	6.104
CH2_N_amd	CH2	10.024	6.908	9.038	7.551	9.371	6.909
CH3_N_amd	CH3	10.787	6.866	11.426	6.904	9.098	6.949

Table S.19: Final values of the EE parameters of the force fields calibrated using the GM, LB, and WH combination rules (considering the alternative calibration replicas). See also Tab. S.21 for the third replica with WH.

LJ type	WH				
	σ	$\tilde{\sigma}$	σ^*	ϵ	ϵ^*
	[nm]			[kJ·mol^{-1}]	
Carbon					
CH0	0.541	-	0.433	0.004	0.003
CH1	0.375	-	0.300	0.136	0.109
CH2	0.396	-	0.317	0.417	0.334
CH3	0.386	-	0.309	0.901	0.721
CH4	0.450	-	0.360	0.919	0.735
C=O	0.351	-	0.281	0.701	0.561
Halogen					
F	0.311	-	0.249	0.911	0.729
Cl	0.341	-	0.272	1.577	1.261
Br	0.363	-	0.291	2.366	1.893
I	0.388	-	0.310	3.060	2.448
Oxygen					
OC	0.321	0.353	0.257	0.696	0.557
O=C	0.205	0.223	0.164	1.233	0.986
OH	0.185	0.190	0.148	0.599	0.479
Nitrogen					
N_amn	0.283	0.270	0.226	0.477	0.382
N_amd	0.342	0.342	0.273	0.311	0.249
Hydrogen					
HC	0.172	-	0.138	0.114	0.091
HB	0.000	-	0.000	0.000	0.000

Table S.20: Final values of the LJ parameters of the force field calibrated using the WH combination rule (considering the second alternative run).

Atom type (EE-type)	LJ-type	WH η $[e^{-1}\cdot V]$	WH χ [V]
Aliphatic carbon (united-)atoms			
CH0	CH0	-	-
CH1	CH1	-	-
CH2	CH2	-	-
CH3	CH3	-	-
CH4	CH4	-	-
Halogen			
F_hal	F	42.471	17.468
Cl_hal	Cl	31.373	15.022
Br_hal	Br	22.067	8.611
I_hal	I	27.897	9.043
CH0_hal	CH0	18.940	4.909
CH1_hal	CH1	10.652	8.896
CH2_hal	CH2	7.280	6.200
CH3_hal	CH3	10.229	13.725
Ether			
O_eth	OC	24.480	6.187
CH0_O_eth	CH0	11.161	6.320
CH1_O_eth	CH1	15.960	7.333
CH2_O_eth	CH2	8.108	5.565
CH3_O_eth	CH3	6.841	4.420
Aldehyde			
H_CO_ald	HC	9.425	4.655
C_ald	C=O	11.343	4.640
O_ald	O=C	11.538	6.968
Ketone			
C_ket	C=O	7.821	6.489
O_ket	O=C	7.858	5.104
Ester			
H_CO_est	HC	30.235	7.273
C_est	C=O	10.617	9.583
O_est	O=C	10.098	10.987
O_C_est	OC	10.127	10.514
CH0_O_est	CH0	10.578	9.441
CH1_O_est	CH1	10.581	9.172
CH2_O_est	CH2	10.641	9.505
CH3_O_est	CH3	10.583	9.340
Alcohol			
H_ol	HB	17.881	6.641
O_ol	OH	14.265	10.028
CH0_O_ol	CH0	9.652	7.590
CH1_O_ol	CH1	9.932	6.502
CH2_O_ol	CH2	10.351	6.092
CH3_O_ol	CH3	10.887	7.376
Carboxylic acid			
H_CO_acd	HC	18.214	6.720
C_acd	C=O	6.862	8.515
O_acd	O=C	15.162	7.641
H_O_acd	HB	8.954	7.171
O_H_acd	OH	9.779	7.900
H_N_amn	HB	11.261	9.746
Amine			
N_amn	N_amn	13.728	8.737
CH0_N_amn	CH0	10.059	12.577
CH1_N_amn	CH1	7.387	7.541
CH2_N_amn	CH2	7.461	7.351
CH3_N_amn	CH3	19.114	8.292
Amide			
H_N_amd	HC	11.119	8.510
C_amd	C=O	11.168	4.733
O_amd	O=C	9.118	8.672
N_amd	N_amd	9.656	10.880
CH0_N_amd	CH0	-	-
CH1_N_amd	CH1	14.142	6.534
CH2_N_amd	CH2	8.529	8.006
CH3_N_amd	CH3	9.635	7.488

Table S.21: Final values of the EE parameters of the force field calibration using the WH combination rule (considering the second alternative run).

S.13.2 Evolution of the Non-Bonded Interaction Parameters

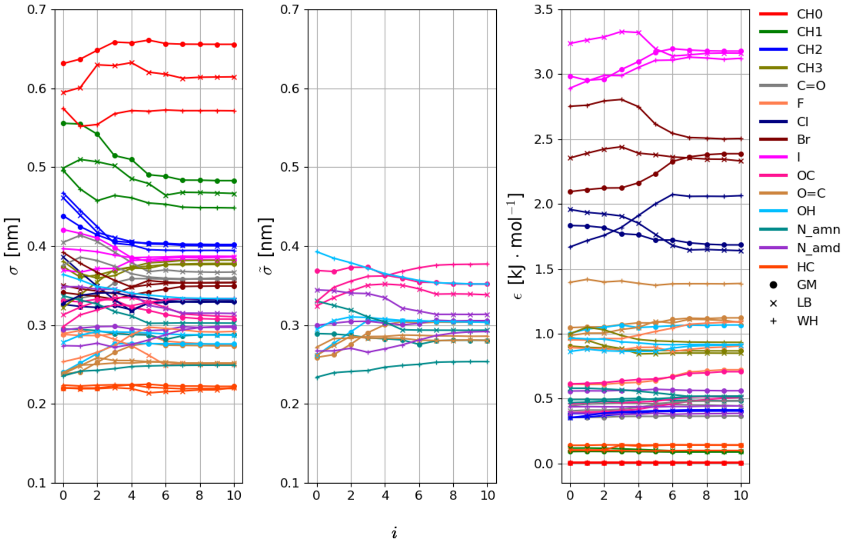

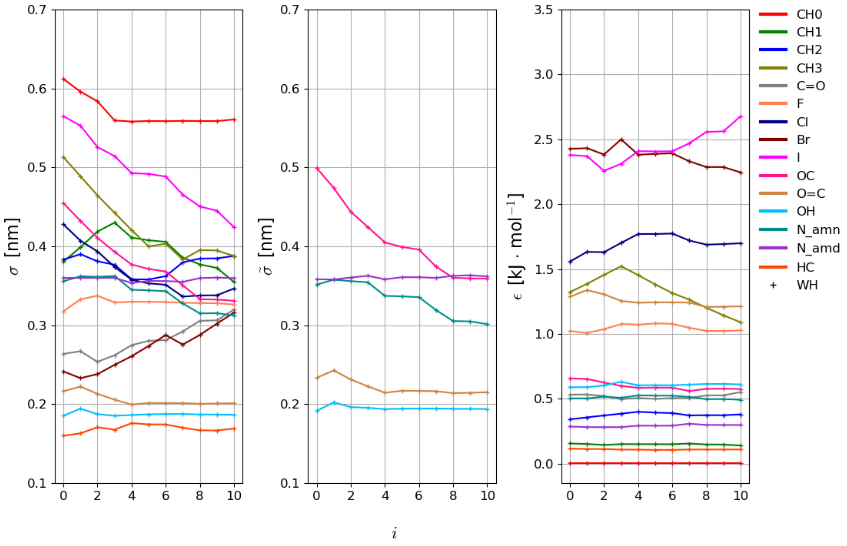

Figure S.12: Evolution of the 37 LJ parameters against the iteration number i along the force-field parameter optimization for the geometric-mean (GM), Lorentz-Berthelot (LB), and Waldman-Hagler (WH) combination rules for the additional runs. The main results reported in the Main Article are shown in Fig. 4. The parameters considered are the LJ collision diameter σ or $\tilde{\sigma}$ (the latter for hydrogen-bonding types), and the well depth ϵ. The $N_{\mathrm{att}}^{\mathrm{LJ}} = 17$ LJ-types are listed in Tab. 4. The final parameter values are reported numerically in Tabs. S.18 and S.20. Note that the parameters $\tilde{\sigma}$ are only relevant for potentially hydrogen-bonding LJ-types (5 types), and the LJ-type HB is omitted from the graph (σ and ϵ set to zero). The top graph shows the calibration replicas for the three combination rules, and the bottom graph corresponds to the second replica for WH. Note that the lines have no physical meaning and are intended only as a guide to the eye.

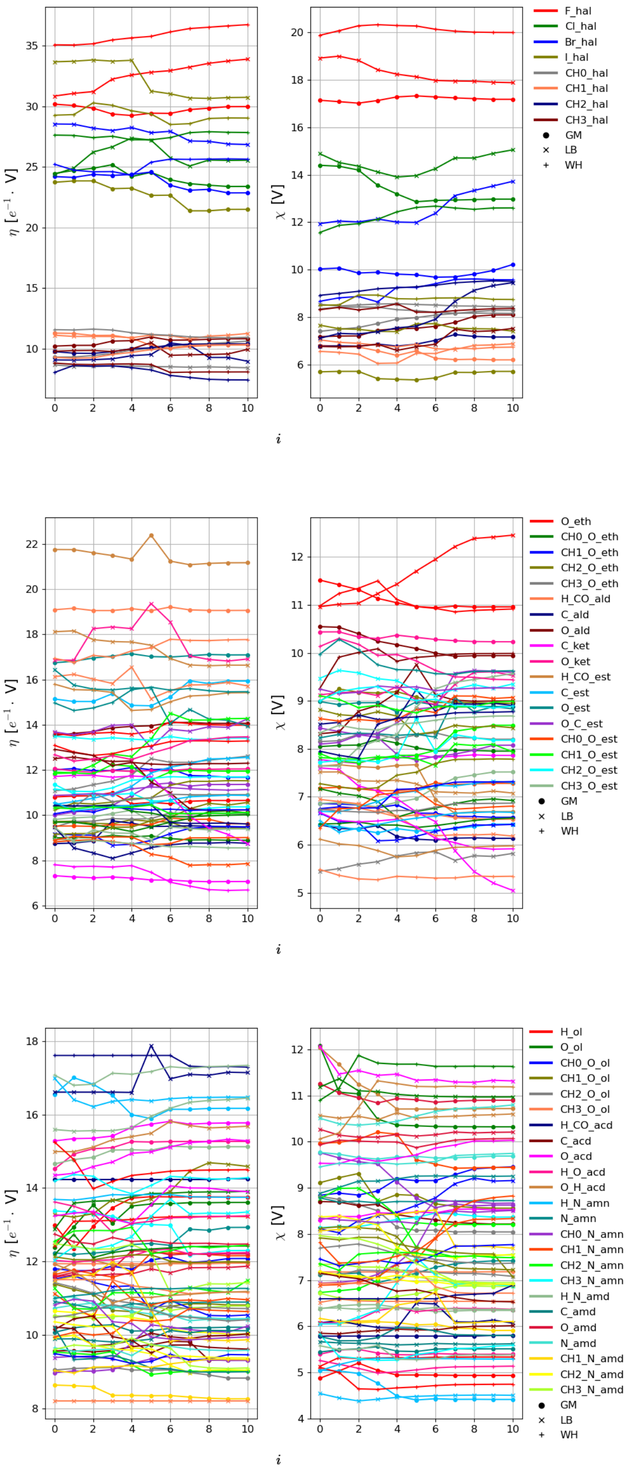

Figure S.13: Evolution of the 100 EE interaction parameters against the iteration number i along the force-field parameter optimization for the geometric-mean (GM), Lorentz-Berthelot (LB), and Waldman-Hagler (WH) combination rules for the additional runs. The parameters considered are the electrostatic hardness η and electronegativity χ. The $N_{\text{att}}^{\text{EE}} = 56$ EE-types are listed in Tab. 3. The final parameter values are reported numerically in Tabs. S.19 and S.21. Note that the aliphatic united-atom EE-types are omitted from the graph (5 types with zero charge) as well as the EE-type CH0_N_amd (no representative molecule for calibration). These graphs show the calibration replicas for the three combination rules. The second replica for WH is shown in Fig. S.14. Note that the lines have no physical meaning and are intended only as a guide to the eye.

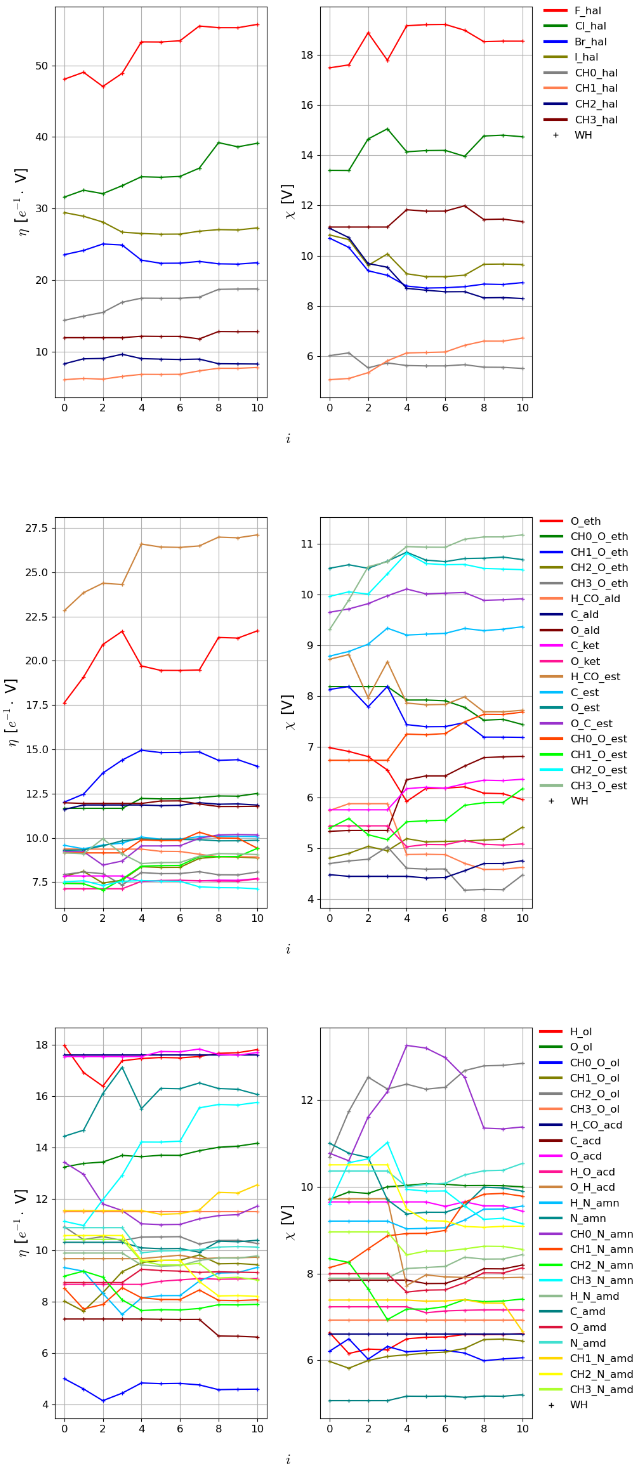

Figure S.14: Evolution of the 100 EE interaction parameters against the iteration number i along the force-field parameter optimization for the third run using the Waldman-Hagler (WH) combination rules. The parameters considered are the electrostatic hardness η and electronegativity χ. The $N_{\text{att}}^{\text{EE}} = 56$ EE-types are listed in Tab. 3. The final parameter values are reported numerically in Suppl. Mat. Tab. S.7. Note that the aliphatic united-atom EE-types are omitted from the graph (5 types with zero charge) as well as the EE-type CH0_N_amd (no representative molecule for calibration). The first replicas for the three combination rules are shown in Fig. S.13. Note that the lines have no physical meaning and are intended only as a guide to the eye.

S.13.3 Charge Distribution

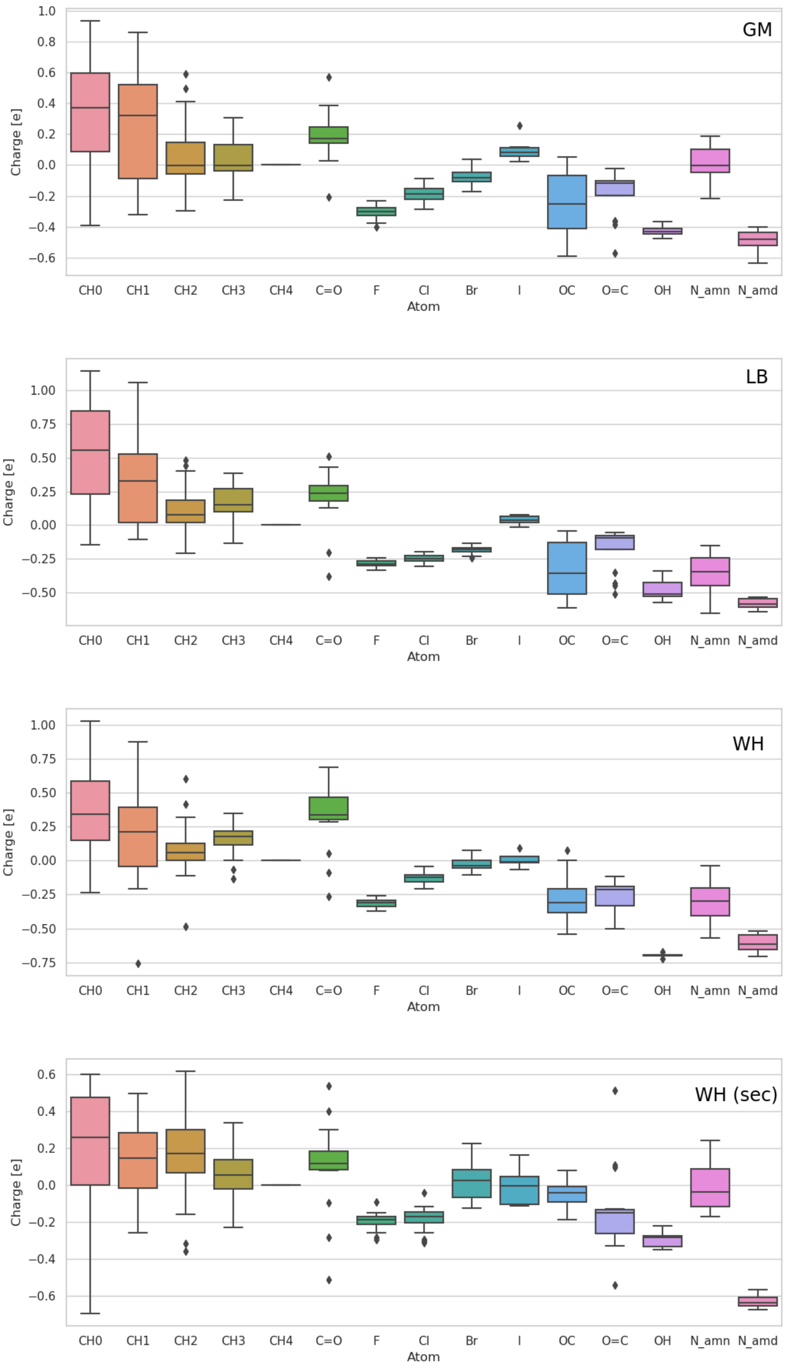

Figure S.15: Distribution of the possible EE-derived atomic partial charges for the different LJ types: GM (top), LB (middle) and WH (bottom). The boxes show the minimum, first quartile, median, third quartile, maximum, and outlier values of the distribution. Each possible value of the charge is only counted once in the distribution (irrespective of the number of molecules in which the particular charge occurs). The $N_{\text{att}}^{\text{LJ}} = 15$ LJ-types are listed in Tab. 4. The different graphs show the results for the different replicas.

S.13.4 Statistics per Combining Rule

He, we provide the statistics concerning the discrepancies between simulated and experimental properties based on the optimized force field for each combination rule and for the entire set of molecules considering the different replicas (Tabs. S.22-S.25). For selected groups of molecules (Tab. 2) and number m of occurrences of the functional group in the molecule, the number N_{ρ}^{cal} of experimental ρ_{liq} values and the number $N_{\Delta H}^{\text{cal}}$ of experimental ΔH_{vap} values are reported, along with the root-mean-square deviation (RMSD) and the average deviation (AVED) between simulation and experiment for both properties. The last five lines refer to molecules with two or more distinct functional groups (MIX), halogens (HAL), non-hydrogen-bonding (NHB), hydrogen-bonding (HBD), and the entire set (ALL) of molecules.

Group	m	$N_{\mathrm{iso}}^{\mathrm{sim}}$	N_{ρ}^{cal}	RMSD	AVED	MAD	AVG	$N_{\Delta H}^{\mathrm{cal}}$	RMSD	AVED	MAD	AVG
ALK	-	149	109	10.2	7.9	8.3	695.6	150	0.9	-0.1	0.6	41.8
HAL	-	126	132	50.6	-3.8	22.7	1330.9	73	1.8	-0.4	1.4	37.9
HAL-F	1	16	19	41.1	-15.4	26.0	759.7	6	1.0	0.9	0.9	32.0
HAL-F	2	6	8	78.4	-19.6	62.1	973.5	3	1.4	-0.4	1.3	22.9
HAL-F	3	3	3	268.3	-116.9	254.7	1211.9	2	6.0	-1.4	5.9	17.9
HAL-F	4	1	1	6.1	-6.1	6.1	1605.2	1	2.1	-2.1	2.1	12.3
HAL-F	1-4	26	31	97.9	-26.0	56.8	885.9	12	2.7	-0.0	1.9	25.7
HAL-Cl	1	17	18	8.1	-4.9	5.7	878.4	12	1.6	-1.2	1.2	38.7
HAL-Cl	2	12	11	9.9	0.3	6.7	1140.8	8	0.7	-0.3	0.5	38.5
HAL-Cl	3	3	3	20.4	12.7	16.0	1413.9	3	2.0	1.3	1.9	34.5
HAL-Cl	4	1	1	34.2	34.2	34.2	1584.3	1	2.4	2.4	2.4	32.4
HAL-Cl	1-4	33	33	11.9	-0.4	7.8	1035.9	24	1.5	-0.5	1.1	37.9
HAL-Br	1	22	23	8.9	-1.6	7.4	1252.7	14	1.8	-1.7	1.7	38.5
HAL-Br	2	9	9	13.4	3.9	10.1	1965.7	4	1.3	0.0	1.3	41.5
HAL-Br	3	7	7	36.7	22.9	28.6	2415.3	1	0.3	0.3	0.3	46.1
HAL-Br	4	1	1	88.7	88.7	88.7	2953.3	1	3.0	3.0	3.0	48.2
HAL-Br	1-4	39	40	22.8	6.2	13.7	1659.1	20	1.8	-1.0	1.6	40.0
HAL-I	1	23	23	14.2	10.0	11.6	1531.9	14	1.3	0.2	0.9	41.8
HAL-I	2	5	5	46.1	-31.6	31.6	2486.0	3	1.6	-0.9	1.5	53.8
HAL-I	1-2	28	28	23.3	2.6	15.2	1702.3	17	1.3	0.1	1.0	43.9
ROR	1	82	82	6.3	-4.2	4.8	754.3	27	1.8	0.4	1.1	35.9
ROR	2	36	33	24.2	-21.3	21.3	838.6	18	1.7	0.2	1.1	43.9
ROR	3	5	5	33.2	-31.6	31.6	909.2	3	3.6	2.5	2.9	50.7
ROR	1-3	123	120	15.3	-10.1	10.4	784.0	48	1.9	0.5	1.2	39.8
RCOH	1	33	34	19.1	-0.2	11.8	817.8	12	0.9	0.1	0.7	41.8
RCOH	2	1	1	13.8	-13.8	13.8	1003.0	0	-	-	-	-
RCOH	1-2	34	35	19.0	-0.5	11.9	823.1	12	0.9	0.1	0.7	41.8
RCOR	1	82	82	9.1	2.1	7.6	817.2	22	1.0	-0.6	0.8	43.7
RCOR	2	3	3	10.8	10.0	10.0	952.4	0	-	-	-	-
RCOR	1-2	85	85	9.2	2.4	7.7	822.0	22	1.0	-0.6	0.8	43.7
RCOOR	1	149	144	8.1	-1.2	6.1	868.6	54	1.6	1.1	1.3	45.7
RCOOR	2	34	31	20.7	-18.5	18.6	1052.2	20	2.2	-0.4	1.9	65.0
RCOOR	1-2	183	175	11.4	-4.2	8.3	901.1	74	1.8	0.7	1.4	50.9
ROH	1	262	261	13.3	6.7	9.8	827.4	56	3.8	0.3	2.5	57.4
ROH	2	88	86	21.0	-13.4	16.7	958.9	15	6.0	-4.5	4.8	78.8
ROH	3	6	6	22.0	-17.6	17.6	1137.5	1	6.7	-6.7	6.7	85.8
ROH	1-3	356	353	15.7	1.4	11.6	864.7	72	4.4	-0.8	3.1	62.3
RCOOH	1	48	48	48.0	44.4	44.4	922.1	17	3.7	-1.6	2.3	65.8
RN	1	87	88	40.0	16.5	19.3	736.7	32	2.0	-0.7	1.5	37.7
RN	2	30	19	72.7	55.1	55.8	824.9	19	3.9	-1.8	2.5	53.1
RN	1-2	117	107	47.5	23.4	25.8	752.4	51	2.8	-1.1	1.9	43.5
RCON	1	19	15	19.5	10.4	12.8	920.9	10	6.3	-0.5	4.1	61.6
MIX	-	272	254	85.7	-3.1	39.8	1274.1	71	5.2	1.3	3.8	45.1
NHB	-	425	415	13.1	-4.3	9.1	844.5	156	1.7	0.4	1.2	45.8
HBD	-	540	523	29.2	10.1	17.6	848.6	150	4.0	-1.0	2.6	56.2
ALL	-	1512	1433	43.7	2.2	18.8	955.6	600	2.9	-0.1	1.7	46.4

Table S.22: Statistics concerning the discrepancies between simulated and experimental properties with the GM combination rule with the alternative replicas.

Group	m	$N_{\mathrm{iso}}^{\mathrm{sim}}$	N_{ρ}^{cal}	RMSD	AVED	MAD	AVG	$N_{\Delta H}^{\mathrm{cal}}$	RMSD	AVED	MAD	AVG
ALK	-	148	107	9.3	6.1	8.0	700.2	148	0.7	-0.1	0.5	42.3
HAL	-	124	130	44.1	0.3	22.4	1318.4	72	1.6	-0.4	1.3	37.7
HAL-F	1	16	19	45.4	-10.4	31.6	759.7	6	0.9	-0.1	0.6	32.0
HAL-F	2	5	7	66.1	-22.0	50.8	973.0	3	2.5	-2.4	2.4	22.9
HAL-F	3	3	3	223.7	124.1	210.7	1211.9	2	1.8	0.5	1.7	17.9
HAL-F	4	1	1	6.7	-6.7	6.7	1605.2	1	3.5	-3.5	3.5	12.3
HAL-F	1-4	25	30	85.6	0.5	53.1	882.9	12	1.9	-0.8	1.5	25.7
HAL-Cl	1	17	18	13.7	-8.0	10.2	878.4	12	1.8	-1.4	1.4	38.7
HAL-Cl	2	12	11	9.3	-5.0	7.1	1140.8	8	1.8	-1.1	1.3	38.5
HAL-Cl	3	3	3	16.6	7.7	14.6	1413.9	3	2.1	1.9	1.9	34.5
HAL-Cl	4	1	1	8.9	8.9	8.9	1584.3	1	1.7	1.7	1.7	32.4
HAL-Cl	1-4	33	33	12.6	-5.1	9.5	1035.9	24	1.8	-0.8	1.5	37.9
HAL-Br	1	22	23	13.1	-3.5	10.9	1252.7	14	1.2	-1.0	1.1	38.5
HAL-Br	2	9	9	15.6	-1.1	12.8	1965.7	4	1.9	1.2	1.5	41.5
HAL-Br	3	7	7	35.2	23.6	25.1	2415.3	1	1.6	1.6	1.6	46.1
HAL-Br	4	1	1	23.0	23.0	23.0	2953.3	1	2.6	2.6	2.6	48.2
HAL-Br	1-4	39	40	19.6	2.5	14.1	1659.1	20	1.5	-0.3	1.3	40.0
HAL-I	1	23	23	22.1	5.3	16.6	1531.9	14	1.3	0.2	1.0	41.8
HAL-I	2	4	4	14.8	-8.2	12.5	2280.6	2	1.0	-0.2	1.0	56.2
HAL-I	1-2	27	27	21.2	3.3	16.0	1642.9	16	1.3	0.2	1.0	43.6
ROR	1	82	82	8.6	-5.5	6.3	754.3	27	2.1	0.8	1.5	35.9
ROR	2	36	33	28.8	-22.5	22.8	838.6	18	3.2	0.2	2.2	43.9
ROR	3	5	5	35.8	-32.3	32.3	909.2	3	6.6	3.5	5.4	50.7
ROR	1-3	123	120	18.2	-11.3	12.0	784.0	48	3.0	0.7	2.0	39.8
RCOH	1	33	34	22.8	3.9	14.0	817.8	12	1.2	-0.6	1.0	41.8
RCOH	2	1	1	1.6	1.6	1.6	1003.0	0	-	-	-	-
RCOH	1-2	34	35	22.4	3.9	13.6	823.1	12	1.2	-0.6	1.0	41.8
RCOR	1	82	82	8.8	5.0	7.4	817.2	22	1.0	-0.4	0.8	43.7
RCOR	2	3	3	18.4	18.0	18.0	952.4	0	-	-	-	-
RCOR	1-2	85	85	9.3	5.5	7.8	822.0	22	1.0	-0.4	0.8	43.7
RCOOR	1	149	144	9.0	-1.6	7.0	868.6	54	1.4	0.9	1.1	45.7
RCOOR	2	34	31	16.7	-14.5	14.8	1052.2	20	2.3	-0.7	1.8	65.0
RCOOR	1-2	183	175	10.8	-3.9	8.4	901.1	74	1.7	0.4	1.3	50.9
ROH	1	262	261	11.5	1.3	7.8	827.4	56	3.6	1.1	2.0	57.4
ROH	2	89	87	35.1	-27.9	28.7	959.2	15	7.4	-5.3	5.4	78.8
ROH	3	6	6	81.9	-69.5	69.5	1137.5	1	10.0	-10.0	10.0	85.8
ROH	1-3	357	354	22.7	-7.1	14.0	865.1	72	4.8	-0.3	2.8	62.3
RCOOH	1	48	48	35.8	33.9	33.9	922.1	17	1.6	-0.6	1.2	65.8
RN	1	87	88	18.6	9.2	12.9	736.7	32	2.2	-0.7	1.5	37.7
RN	2	30	19	50.2	41.6	41.6	824.9	19	3.3	-0.2	2.4	53.1
RN	1-2	117	107	27.1	14.9	18.0	752.4	51	2.6	-0.5	1.8	43.5
RCON	1	18	15	16.9	7.9	10.4	920.9	9	2.0	-0.5	1.4	64.4
MIX	-	267	247	103.3	-0.2	43.1	1264.9	72	5.0	0.5	3.8	45.1
NHB	-	425	415	14.3	-3.5	9.7	844.5	156	2.1	0.3	1.4	45.8
HBD	-	540	524	24.9	1.6	16.5	848.9	149	3.7	-0.4	2.2	56.4
ALL	-	1504	1423	48.2	0.0	19.1	951.5	597	2.8	-0.0	1.7	46.5

Table S.23: Statistics concerning the discrepancies between simulated and experimental properties with the LB combination rule with the alternative replicas.

Group	m	$N_{\text{iso}}^{\text{sim}}$	N_{ρ}^{cal}	RMSD	AVED	MAD	AVG	$N_{\Delta H}^{\text{cal}}$	RMSD	AVED	MAD	AVG
ALK	-	148	107	26.6	26.2	26.2	700.2	148	0.8	0.0	0.6	42.3
HAL	-	126	133	91.2	6.4	39.3	1324.4	72	2.5	-0.5	1.8	37.8
HAL-F	1	16	19	52.2	-17.9	40.6	759.7	6	1.4	0.4	0.9	32.0
HAL-F	2	7	9	92.8	-69.1	84.2	977.1	3	2.0	0.9	1.4	22.9
HAL-F	3	3	4	465.9	123.8	348.1	1068.1	2	6.4	3.0	5.7	17.9
HAL-F	4	1	1	161.3	-161.3	161.3	1605.2	1	3.0	3.0	3.0	12.3
HAL-F	1-4	27	33	176.1	-19.0	93.5	882.0	12	3.1	1.1	2.0	25.7
HAL-Cl	1	17	18	10.7	4.4	8.9	878.4	12	2.7	-2.4	2.4	38.7
HAL-Cl	2	11	10	13.3	-2.3	9.6	1136.9	7	3.3	-2.1	2.3	38.3
HAL-Cl	3	3	3	40.8	23.3	27.9	1413.9	3	3.4	2.7	2.7	34.5
HAL-Cl	4	1	1	46.6	46.6	46.6	1584.3	1	6.8	6.8	6.8	32.4
HAL-Cl	1-4	32	32	18.5	5.4	12.1	1031.4	23	3.2	-1.3	2.6	37.8
HAL-Br	1	22	23	21.5	18.3	18.9	1252.7	14	1.5	-1.4	1.4	38.5
HAL-Br	2	9	9	15.9	8.3	11.9	1965.7	4	0.8	-0.1	0.6	41.5
HAL-Br	3	7	7	27.1	12.6	21.3	2415.3	1	1.5	1.5	1.5	46.1
HAL-Br	4	1	1	4.9	4.9	4.9	2953.3	1	3.9	3.9	3.9	48.2
HAL-Br	1-4	39	40	21.3	14.7	17.4	1659.1	20	1.6	-0.8	1.4	40.0
HAL-I	1	23	23	38.4	34.9	37.4	1531.9	14	1.2	0.2	0.8	41.8
HAL-I	2	5	5	63.2	-16.8	40.0	2486.0	3	1.9	-1.6	1.6	53.8
HAL-I	1-2	28	28	43.9	25.7	37.8	1702.3	17	1.3	-0.1	0.9	43.9
ROR	1	82	82	10.7	6.8	9.7	754.3	27	1.6	0.4	1.1	35.9
ROR	2	36	33	23.6	-17.3	17.3	838.6	18	2.0	-0.5	1.2	43.9
ROR	3	5	5	36.1	-33.8	33.8	909.2	3	3.1	1.6	2.4	50.7
ROR	1-3	123	120	16.9	-1.5	12.8	784.0	48	1.9	0.1	1.2	39.8
RCOH	1	33	34	22.7	-7.8	16.1	817.8	12	0.6	0.2	0.5	41.8
RCOH	2	1	1	25.0	-25.0	25.0	1003.0	0	-	-	-	-
RCOH	1-2	34	35	22.8	-8.3	16.4	823.1	12	0.6	0.2	0.5	41.8
RCOR	1	82	82	8.0	4.1	6.8	817.2	22	0.8	-0.0	0.7	43.7
RCOR	2	3	3	3.0	-1.3	2.9	952.4	0	-	-	-	-
RCOR	1-2	85	85	7.9	3.9	6.6	822.0	22	0.8	-0.0	0.7	43.7
RCOOR	1	148	143	13.4	-3.3	8.8	867.9	53	1.0	0.4	0.8	46.0
RCOOR	2	34	31	32.2	-27.0	30.2	1052.2	20	1.6	0.5	1.3	65.0
RCOOR	1-2	182	174	18.2	-7.6	12.6	900.7	73	1.2	0.4	0.9	51.2
ROH	1	263	262	13.8	2.8	9.6	827.3	57	3.8	1.3	2.0	57.1
ROH	2	89	87	56.4	-49.3	49.3	959.2	15	4.9	-2.8	3.5	78.8
ROH	3	6	6	161.8	-142.9	142.9	1137.5	1	15.1	-15.1	15.1	85.8
ROH	1-3	358	355	36.9	-12.4	21.6	864.9	73	4.4	0.2	2.5	61.9
RCOOH	1	48	48	23.7	18.7	18.7	922.1	17	2.4	-0.3	2.0	65.8
RN	1	87	87	23.7	8.7	18.8	737.6	32	3.6	-2.1	2.6	37.7
RN	2	23	14	39.6	29.3	33.4	816.7	15	4.3	-2.0	3.0	55.1
RN	1-2	110	101	26.5	11.6	20.9	748.6	47	3.9	-2.1	2.7	43.3
RCON	1	19	15	21.7	3.7	16.6	920.9	10	6.8	0.5	3.8	61.6
MIX	-	262	242	84.5	-40.2	54.0	1263.8	72	5.9	1.8	4.5	45.1
NHB	-	424	414	16.7	-3.5	11.7	844.2	155	1.4	0.2	1.0	45.9
HBD	-	535	519	33.7	-4.4	21.0	849.2	147	4.3	-0.6	2.6	56.4
ALL	-	1495	1415	50.5	-6.9	26.1	952.0	594	3.2	0.1	1.8	46.5

Table S.24: Statistics concerning the discrepancies between simulated and experimental properties with the WH combination rule with the first alternative replicas.

Group	m	$N_{\mathrm{iso}}^{\mathrm{sim}}$	N_{ρ}^{cal}	RMSD	AVED	MAD	AVG	$N_{\Delta H}^{\mathrm{cal}}$	RMSD	AVED	MAD	AVG
ALK	-	148	107	18.2	16.2	16.7	700.2	148	1.1	0.1	0.8	42.3
HAL	-	127	133	84.2	-10.1	42.8	1330.0	74	2.4	0.2	1.4	37.5
HAL-F	1	16	17	67.0	-52.7	52.7	784.3	6	1.8	-0.8	1.1	32.0
HAL-F	2	7	9	98.4	-86.2	86.2	977.1	3	1.4	-0.0	1.3	22.9
HAL-F	3	3	5	357.0	147.4	285.0	987.8	3	5.6	4.4	4.4	16.3
HAL-F	4	1	1	1.9	1.9	1.9	1605.2	1	10.8	10.8	10.8	12.3
HAL-F	1-4	27	32	158.2	-29.1	96.8	895.9	13	4.3	1.5	2.6	24.8
HAL-Cl	1	17	18	37.7	-17.3	21.2	878.4	12	1.6	-0.9	1.1	38.7
HAL-Cl	2	12	11	15.4	-12.1	12.1	1140.8	8	2.1	0.2	1.4	38.5
HAL-Cl	3	3	3	52.5	6.8	48.5	1413.9	3	2.0	0.7	2.0	34.5
HAL-Cl	4	1	1	31.5	-31.5	31.5	1584.3	1	1.1	1.1	1.1	32.4
HAL-Cl	1-4	33	33	33.7	-13.8	21.0	1035.9	24	1.8	-0.2	1.3	37.9
HAL-Br	1	22	23	29.3	-7.2	19.4	1252.7	14	0.9	-0.7	0.8	38.5
HAL-Br	2	9	9	50.0	-22.7	33.4	1965.7	4	1.2	0.6	1.0	41.5
HAL-Br	3	7	7	24.3	10.0	18.7	2415.3	1	3.8	3.8	3.8	46.1
HAL-Br	4	1	1	45.2	-45.2	45.2	2953.3	1	5.3	5.3	5.3	48.2
HAL-Br	1-4	39	40	34.8	-8.7	23.1	1659.1	20	1.7	0.1	1.2	40.0
HAL-I	1	23	23	37.5	23.1	33.7	1531.9	14	1.2	0.5	0.8	41.8
HAL-I	2	5	5	70.1	-28.1	41.6	2486.0	3	2.0	-1.6	1.6	53.8
HAL-I	1-2	28	28	45.1	14.0	35.1	1702.3	17	1.4	0.1	1.0	43.9
ROR	1	82	82	16.1	-0.5	10.1	754.3	27	1.7	0.8	1.4	35.9
ROR	2	36	33	29.5	-23.9	24.9	838.6	18	1.7	0.0	1.2	43.9
ROR	3	5	5	50.2	-48.2	48.2	909.2	3	2.4	0.4	2.0	50.7
ROR	1-3	123	120	22.8	-8.9	15.8	784.0	48	1.8	0.5	1.4	39.8
RCOH	1	33	34	24.5	-8.0	14.4	817.8	12	0.8	0.2	0.7	41.8
RCOH	2	1	1	15.8	-15.8	15.8	1003.0	0	-	-	-	-
RCOH	1-2	34	35	24.3	-8.2	14.5	823.1	12	0.8	0.2	0.7	41.8
RCOR	1	82	82	8.4	-0.7	6.5	817.2	22	0.7	0.1	0.6	43.7
RCOR	2	3	3	2.8	-0.2	2.3	952.4	0	-	-	-	-
RCOR	1-2	85	85	8.2	-0.7	6.3	822.0	22	0.7	0.1	0.6	43.7
RCOOR	1	149	144	36.2	-0.0	20.0	868.6	54	1.9	-0.2	1.6	45.7
RCOOR	2	34	31	45.7	-34.8	41.4	1052.2	20	5.5	-5.2	5.2	65.0
RCOOR	1-2	183	175	38.0	-6.2	23.8	901.1	74	3.3	-1.6	2.6	50.9
ROH	1	259	258	56.5	-44.2	46.4	827.9	53	21.9	-15.6	17.1	58.1
ROH	2	85	83	152.3	-140.0	140.0	955.4	12	55.7	-53.2	53.2	81.3
ROH	3	6	6	282.8	-264.5	264.5	1137.5	1	45.0	-45.0	45.0	85.8
ROH	1-3	350	347	96.4	-70.9	72.5	863.7	66	31.3	-22.9	24.1	62.8
RCOOH	1	48	48	31.6	24.8	29.0	922.1	17	2.3	-0.7	1.6	65.8
RN	1	77	77	33.6	14.6	20.7	736.5	31	3.1	0.5	2.2	38.1
RN	2	26	16	45.5	27.7	32.2	822.7	17	4.3	-1.7	2.7	53.5
RN	1-2	103	93	35.9	16.9	22.7	751.4	48	3.6	-0.3	2.3	43.6
RCON	1	19	15	26.7	-22.3	23.5	920.9	10	7.3	1.7	3.3	61.6
MIX	-	259	242	100.1	-49.7	66.7	1287.5	68	14.9	-4.9	11.1	44.5
NHB	-	425	415	28.7	-6.0	17.1	844.5	156	2.5	-0.6	1.8	45.8
HBD	-	520	503	82.3	-44.1	57.7	850.2	141	21.6	-10.8	12.5	56.5
T	-	1479	1400	71.5	-25.9	42.7	958.2	587	11.9	-3.3	5.1	46.3

Table S.25: Statistics concerning the discrepancies between simulated and experimental properties with the WH combination rule with the second alternative replicas.
